# Suppressors of Break-Induced Replication in Human Cells

**DOI:** 10.3390/genes14020398

**Published:** 2023-02-03

**Authors:** Stanley Dean Rider, French J. Damewood, Rujuta Yashodhan Gadgil, David C. Hitch, Venicia Alhawach, Resha Shrestha, Matilyn Shanahan, Nathen Zavada, Michael Leffak

**Affiliations:** Department of Biochemistry and Molecular Biology, Wright State University, Dayton, OH 45435, USA

**Keywords:** break-induced replication, DNA replication, DNA repair, COPS2, repeat-induced mutagenesis

## Abstract

Short tandem DNA repeats are drivers of genome instability. To identify suppressors of break-induced mutagenesis human cells, unbiased genetic screens were conducted using a lentiviral shRNA library. The recipient cells possessed fragile non-B DNA that could induce DNA double-strand breaks (DSBs), integrated at an ectopic chromosomal site adjacent to a thymidine kinase marker gene. Mutagenesis of the thymidine kinase gene rendered cells resistant to the nucleoside analog ganciclovir (GCV). The screen identified genes that have established roles in DNA replication and repair, chromatin modification, responses to ionizing radiation, and genes encoding proteins enriched at replication forks. Novel loci implicated in BIR included olfactory receptors, the G0S2 oncogene/tumor suppressor axis, the EIF3H-METTL3 translational regulator, and the SUDS3 subunit of the Sin3A corepressor. Consistent with a role in suppressing BIR, siRNA knockdown of selected candidates increased the frequency of the GCV^r^ phenotype and increased DNA rearrangements near the ectopic non-B DNA. Inverse PCR and DNA sequence analyses showed that hits identified in the screen increased genome instability. Further analysis quantitated repeat-induced hypermutagenesis at the ectopic site and showed that knockdown of a primary hit, COPS2, induced mutagenic hotspots, remodeled the replication fork, and increased nonallelic chromosome template switches.

## 1. Introduction

DNA microsatellites are a diverse collection of short (1–9 bp) sequences each tandemly repeated ~10–40 times, which cumulatively represent about 3% of the human genome [1,2,3]. Microsatellite instability is responsible for recurrent hypermutation and gross chromosomal rearrangements (GCRs) in more than forty neuromuscular diseases [4,5,6,7,8] and multiple cancers [9,10,11,12]. These short sequence repeats are hotspots of DSBs, indels, and GCRs [13,14,15,16,17,18,19].

Replication-induced DSBs have been attributed to (CNG)_n_, (GAA)_n_, and AT-rich repeats at chromosome fragile sites [15,20,21,22,23,24,25,26]. There is also strong evidence of replication-dependent microsatellite recombination at GCR junctions in model organisms and tumors [12,13,21,27,28,29]. Indeed, the majority of cytogenetic lesions resulting from impediments in replication fork progression occur at non-random sites [22,30,31,32,33]. The formation of non-B DNA secondary structures (hairpins, triplexes (H-DNA), guanine quadruplexes (G4), and AT-rich non-B DNA structures) by microsatellites causes fork stalling and replication-dependent genome instability [12,13,17,34,35,36,37,38,39,40,41]. Nevertheless, the mechanisms by which microsatellite structures cause DSBs and subsequent genome instability are incompletely understood [22,30,42,43].

Microsatellite non-B DNA structures blocking DNA replication can be repaired by homology-dependent repair mechanisms [44,45], including break-induced DNA replication (BIR). BIR is a highly mutagenic homology-based repair pathway first described in yeast [46,47]. In human cells BIR has been shown to be a consequence of replication stress, leading to hypermutagenesis and nonallelic translocations [48,49]. Notwithstanding the rarity of BIR, a variant termed microhomology-mediated BIR (MMBIR), has been implicated as the cause of multiple developmental diseases based on GCRs [10,50,51].

Our laboratory has shown that Mus81-dependent DNA double-strand breaks occur at (CTG)_n_ and at homopurine/homopyrimidine (Pu/Py) repeats, leading to hypermutagenesis and translocations from the upstream and downstream ends of the DSBs [52,53]. A closely related homologous recombination-dependent mechanism (recombination-dependent replication, RDR; homologous recombination restarted replication, HoRReR) has been described for the bypass of the RTS replication fork barrier in the absence of detectable DSBs [54,55,56]. Although RDR has not been demonstrated in *Saccharomyces cerevisiae* or in human cells, it is formally possible that a fraction of replication-dependent sister chromatid exchange mutants might derive from this template-switching mechanism.

In this study, we have used clonal cell lines containing unstable (CTG) and homopurine repeats integrated at a common ectopic site, to screen a genomic shRNA library for targets whose knockdown promotes break-induced replication. As expected, multiple proteins involved in (i) DNA replication, (ii) binding to nascent DNA, (iii) the DNA damage response (DDR), or (iv) sensitivity or resistance to ionizing radiation, are found to protect cells from BIR under unperturbed conditions or under replication stress.

Despite high stringency criteria for positive signals from the screen, we find limited overlap between the shRNAs identified in multiple unstressed cell constructs or under different conditions of replication stress. Nevertheless, many of the candidate genes can be organized into shared pathways, suggesting distinct nodes of stabilizing proteins that function cooperatively to protect DNA. Surprisingly, two groups of proteins not normally associated with replication stabilization were revealed by these screens, namely the olfactory receptor family, and a set of oncogenic proteins encoded by long mRNAs whose translation is controlled by the EIF3H-METTL3 master regulator. Knockdown of several of these candidates has been confirmed by inverse PCR to cause instability at the (CTG)_100_ microsatellite. These results dramatically expand the potential repertoire of proteins involved in replication stabilization and suppression of BIR.

## 2. Materials and Methods

### 2.1. Cell Lines

**Figure 1:**
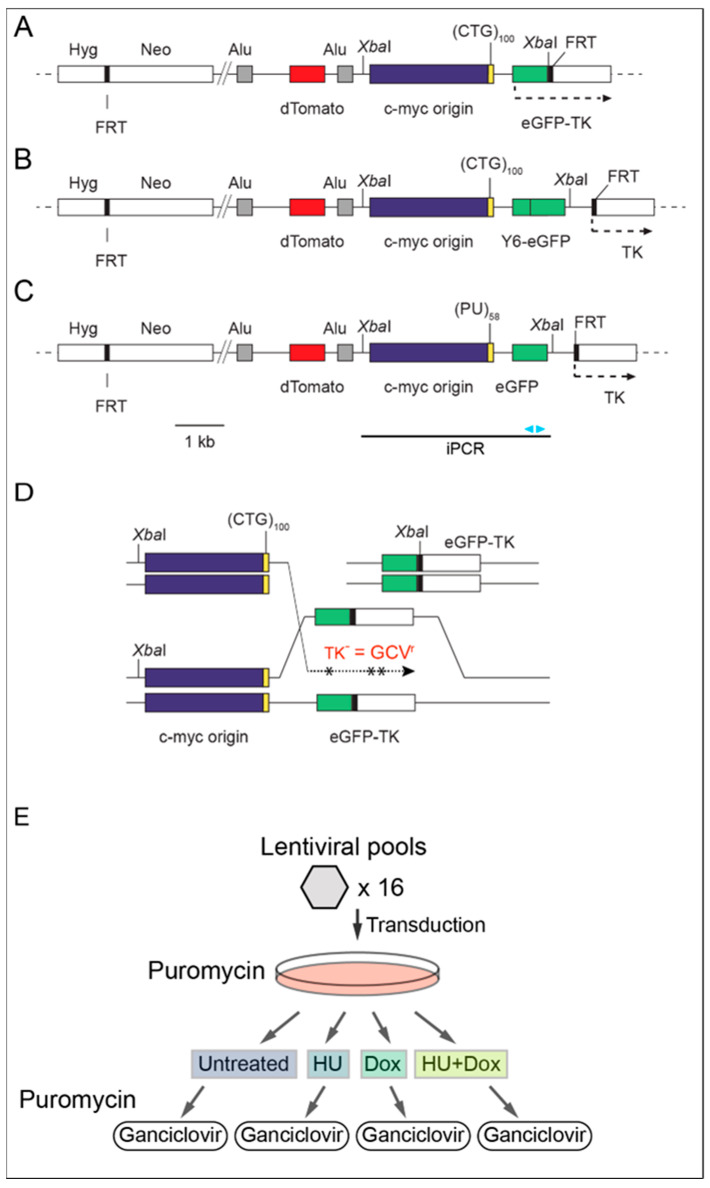
shRNA screen for suppressors of break-induced replication. Maps of the ectopic site constructs integrated into HeLa/406 cells. (**A**) DF/myc(CTG)_100_eGFP/TK contains a hairpin-forming CTG repeat in the lagging strand of DNA synthesis initiated at the c-myc origin. An eGFP-TK fusion gene serves as the marker for negative ganciclovir selection and detection of break-induced replication. (**B**) DF/myc (CTG)_100_/CMV-TK differs principally in that the TK locus is independently driven by the CMV promoter. (**C**) DF/myc(Pu/Py)_58_/CMV-TK possesses a G-quadruplex/triplex forming sequence as the replication barrier and the TK gene is driven by the CMV promoter. The *Xba* I restriction site region used to test for DNA rearrangements by iPCR is indicated and the expected iPCR product is shown at the bottom where arrowheads represent the outward-facing iPCR primers. (**D**) Model of BIR, where mutagenic replication (dashed line with asterisks) leads to TK negative alleles and ganciclovir resistance (GCV^r^). (**E**) Process used for transduction and selection of cells undergoing break-induced replication (BIR) indicating the four treatment groups where doxycycline (Dox) is used to suppress POLD3 through Dox-inducible activation of an shRNA against POLD3 and hydroxyurea (HU) is used to induce replication stress.

A previously described cell line containing the DF/myc(CTG)_100_eGFP/TK ectopic site (Figure 1A) and a doxycycline-inducible shRNA targeting POLD3 was used in the initial screen [52]. A Mus81-dependent DSB occurs at the downstream edge of the (CTG)_100_ replication fork barrier, leading to BIR. BIR results in ganciclovir resistance in cells that undergo mutagenic replication of the eGFP/TK fusion gene downstream of the (CTG)_100_ hairpin-forming microsatellite.

Cells were grown at 37 °C, 5% CO_2_ in DMEM/10% newborn calf serum. GCV (5 μg/mL) resistant colony growth was assayed after 7–14 days of GCV selection as described [57]. Two additional cell lines were made containing replication fork barriers composed of a quadruplex/triplex forming sequence (Pu/Py)_58_ [53] or the hairpin-forming (CTG)_100_, but with the TK gene being driven by a CMV promoter (Figure 1B,C).

### 2.2. Library Screens

DF/myc(CTG)_100_eGFP/TK, DF/myc(Pu/Py)_58_/CMV-TK, and DF/myc (CTG)_100_/CMV-TK cells were transduced with a SMARTvector lentiviral shRNA superpool containing >150,000 different shRNA types, wherein each predicted gene in the human genome was targeted by 5–8 different shRNAs (Horizon Discovery Biosciences Limited). The library also contained 125 different non-targeting controls and 272 different shRNAs that targeted non-essential genes. The initial screen included ~1.2 × 10^6^ viral transduction units at a multiplicity of infection of 0.3. Following expansion under puromycin selection, DF/myc(CTG)_100_eGFP/TK cells were randomly divided into four groups with four replicates of each group. Each group received one of four treatments: (1) untreated (control), (2) hydroxyurea, (3) doxycycline, or (4) hydroxyurea and doxycycline. Doxycycline treatment to induce expression of shPOLD3 was initiated 24 h before beginning hydroxyurea-mediated replication stress. Hydroxyurea and doxycycline treatments lasted for 4 days.

Following treatment, cells were replated at ~30% confluence and challenged with ganciclovir (GCV) for 7–14 days in the presence of puromycin. BIR was scored as the mutagenic repair of the eGFP/TK fusion gene downstream of the (CTG)_100_ hairpin-forming microsatellite, which rendered the cells resistant to GCV (GCV^r^). Culture medium was changed every 2–3 days to remove dead, non-adherent cells and debris. Puromycin was used at a final concentration of 0.5 μg/mL, hydroxyurea was used at a low dose (final concentration) of 0.2 mM, doxycycline was used at a final concentration of 1.0 μg/mL, and ganciclovir at a final concentration of 5.0 μg/mL. The surviving GCV^r^ colonies from each biological replicate yielded sixteen separate populations for DNA extraction. The same transduction methods were used for the DF/myc(Pu/Py)_58_/CMV-TK and DF/myc (CTG)_100_/CMV-TK cell lines. Four biological replicates were completed and the four replicate DF/myc(Pu/Py)_58_/CMV-TK cells were pooled prior to sequence analysis, as were the DF/myc (CTG)_100_/CMV-TK cells.

### 2.3. DNA Extraction and Next Generation Sequencing (NGS)

Genomic DNA was extracted from cells using the E.Z.N.A. Tissue Kit (Omega Bio-Tek, Norcross, GA, USA). One hundred nanograms of genomic DNA was used in PCRs to amplify the region containing the shRNA using HotStar Taq (Qiagen, Germantown, MD, USA). PCR products were cleaned using the Qiaex II gel extraction kit (Qiagen). Amplicons were submitted to GeneWiz (South Plainfield, NJ, USA) for AmpliconEZ sequencing.

### 2.4. Bioinformatics

A local BLAST nucleotide database of guide sequences for the shRNA library was created and used for BLAST with trimmed insert sequences. Forward and reverse reads were counted, and their abundance was converted to the number of reads per million reads based on the total read count. The original shRNA library was provided with counts of the relative abundance of input shRNAs as reads per million reads sequenced. Enrichment of shRNA from the screening process was calculated by dividing the abundance after selection by the value before selection. These fold change values were then evaluated in the non-targeting controls and the candidate shRNAs.

*p*-values (Student’s two-tailed T test) of shRNA hits were calculated for individual replicates from DF/myc(CTG)_100_eGFP/TK cells. The *p*-values were rank ordered and a false discovery rate (FDR) of 0.001 was applied [58,59]. For DF/myc(Pu/Py)_58_/CMV-TK and DF/myc (CTG)_100_/CMV-TK cells hits were considered significant if their enrichment had a Z-score ≥ 2 when compared to the baseline mean and standard deviation of the non-targeting control shRNA sequences. Other considerations for candidate prioritization included the level of enrichment, the presence of multiple shRNA sequences targeting the same locus, the presence of an shRNA in multiple replicates of a given treatment, and the presence of an shRNA in more than one experimental treatment.

Interactivenn (http://www.interactivenn.net/ (accessed on 3 May–21 July 2022)) was used to evaluate the intersections of lists of shRNA clone identifiers and gene loci across treatments. Data were also compared to KEGG pathways (http://www.genome.ad.jp/kegg/ (accessed on 17 July 2022)) for DNA repair, and genes identified in screens for ionizing radiation sensitivity or proteins that are associated with nascent DNA strands. Protein interaction networks were assessed using STRING (https://string-db.org/ (accessed on 13 May–7 July 2022)) while initially limiting the number of interacting partners to the input query proteins and then expanding to a second shell to determine if intermediate interactions could link the query proteins in a network.

### 2.5. siRNA Confirmation

Silencer Select siRNAs were purchased from Ambion (Austin, TX, USA) (Supplementary Table S1. Transfection of siRNA using RNAiMax (Invitrogen, Waltham, MA, USA) was performed according to the manufacturer’s instructions with siRNA between 25–50 nM final concentration. When hydroxyurea was employed, it was added to the culture media 24 h following transfection. A second dose of siRNA was administered at 72 h after the initial dose, and HU treatment (if used) continued for an additional 24 h. At 96 h after the initial transfection, cells were trypsinized, counted using a hemocytometer, and plated in duplicates onto 10 cm dishes without ganciclovir selection, to determine the effects of the siRNA on cell viability, or onto 15 cm dishes with ganciclovir selection to determine the number of ganciclovir resistant cells. Colonies were grown for 1–2 weeks. Colonies were imaged directly or after staining with crystal violet and quantified as the mean grayscale values after background subtraction for viability assays or as individual colonies to determine the number of ganciclovir-resistant cells among viable cells plated. Quantitation was completed using ImageJ. All comparisons were made against samples identically treated with a control non-targeting siRNA or an siRNA targeting a liver glycogen metabolism gene (PYGL).

### 2.6. Inverse PCR

Inverse PCR (iPCR) was performed as described previously [52,53,60] using *Xba*I as the restriction endonuclease prior to circularization of genomic DNA. The primers Gfp-Rev (5′-GTCCATGCCGAGAGTGATC-3′) and Nhe-For (5′-AAGCTTGCCTTGAGTGCTTC-3′) were used with Q5 polymerase mastermix (New England Biolabs) and ~30 ng of circularized genomic DNA.

## 3. Results

To study replication-dependent break-induced DNA damage we analyzed the effects of replication stress on specific non-B DNA forming sequences integrated at a single ectopic FLP recombinase target (FRT) in the HeLa genome [24,53,60,61,62,63,64,65,66]. Repeat-induced mutagenesis occurs via BIR at (CTG)_n_ and homoPu/homoPy non-B microsatellite repeat DNAs leading to a high frequency of nucleotide substitutions, indels, and gross chromosomal rearrangements [52,53,60]. Based on these results, we screened for shRNAs that increase mutagenesis at a herpes virus thymidine kinase (TK) reporter gene 1.5–2 kb away from the non-B DNA. An ultracomplex genomic shRNA library was transduced into the cell line DF/myc(CTG)_100_eGFP/TK (Figure 1A), in which the c-myc core replication origin [67,68,69] is flanked by the hairpin-forming (CTG)_100_ repeat and a constitutively expressed TK gene which renders the cells sensitive to the nucleoside analog ganciclovir (GCV).

To quantitate BIR at the ectopic non-B DNA microsatellites, the initial shRNA screen employed the integrated construct DF/myc(CTG)_100_eGFP/TK shown in Figure 1A, in which the c-myc core replication origin [67,68,69,70,71,72,73,74,75] is adjacent to a hairpin-forming (CTG)_100_ repeat [63,65]. The (CTG)_100_ repeat is in the lagging strand template for replication from the c-myc origin. DF/myc(CTG)_100_eGFP/TK cells also contain an independently integrated, doxycycline-inducible shRNA directed against the DNA polymerase delta POLD3 (POL32) subunit used in metazoan and yeast BIR [52,57,76,77]. DOX treatment reduced POLD3 levels by ~70% and reduced BIR by ~80% in these cells [52]. The c-myc origin and non-B DNA are flanked by AluYA5 element recombination targets, and dual fluorescent (DF) marker genes (dTomato, eGFP), which we used previously as reporters for ectopic site recombination [52,53,78]. Additional screens were performed using a second (CTG)_100_ cell line in which TK was not fused to eGFP (Figure 1B), and a third screening cell line containing part of the asymmetric homoPu/homoPy mirror repeat from the human PKD1 IVS21 locus, capable of forming triplex H-DNA or quadruplex (G4) DNA [53] (Figure 1C). Like the (CTG)_100_ repeats, the homoPu sequence is in the lagging strand replication template from the c-myc origin.

Replication-dependent DSBs map to the ectopic site (CTG) and homoPu/homoPy microsatellite repeats in these non-B DNA reporter cell lines [52,53,60]. Moreover, non-allelic translocations and hypermutagenesis have been detected both upstream and downstream of the DSBs in these cells [52,53]. The eGFP/TK fusion gene contains approximately 150 codons which can be converted to translation stop signals by single base mutation during BIR, resulting in ganciclovir resistance. In screens using the DF/myc(CTG)_100_/CMV-TK [52] or DF/myc(Pu/Py)_58_/CMV-TK [53] cell lines, the TK gene presents a smaller mutational target (~75 codons which can be converted to translation stop signals by single base mutation). All non-B DNA constructs were integrated at the same ectopic site FLP recombinase target (FRT) in HeLa/406 cells [60,61,63,66,69].

The screening strategy is outlined in Figure 1. DF/myc(CTG)_100_eGFP/TK cells were treated in quadruplicate under each of four protocols, viz. untreated, hydroxyurea (HU) treated, doxycycline (DOX) treated, and HU plus DOX treated. Over one million sequence reads were generated from PCR-amplified lentiviral shRNA sequences from GCV^r^ cells. These sequences covered the sixteen samples of GCV^r^ cells in the four different experimental protocols. Each of the four replicates from each of the four treatments was assessed to determine the level of enrichment for shRNA in the screen. Enrichment of shRNA was based on the value of reads per million (RPM) reads sequenced in the original shRNA pool and after GCV selection.

The non-targeting control shRNAs (NTCs) should not have had any effect on BIR in our cell lines, thus the NTCs and other irrelevant gene-targeting shRNAs should have been depleted or absent after selection. Nevertheless, cells harboring NTCs and other irrelevant shRNAs were present at low numbers at the end of the selection process because a minority of these negative control shRNAs were detected after selection. Inasmuch as the non-B DNA reporter cells show significant levels of ectopic site instability under unperturbed conditions [52,53,63,65], these false positive reads may have come from transduced cells that experienced spontaneous BIR before or during the transduction screen, transduced cells that harbored multiple shRNAs, or GCV^s^ cells that persisted through senescence.

**Figure 2:**
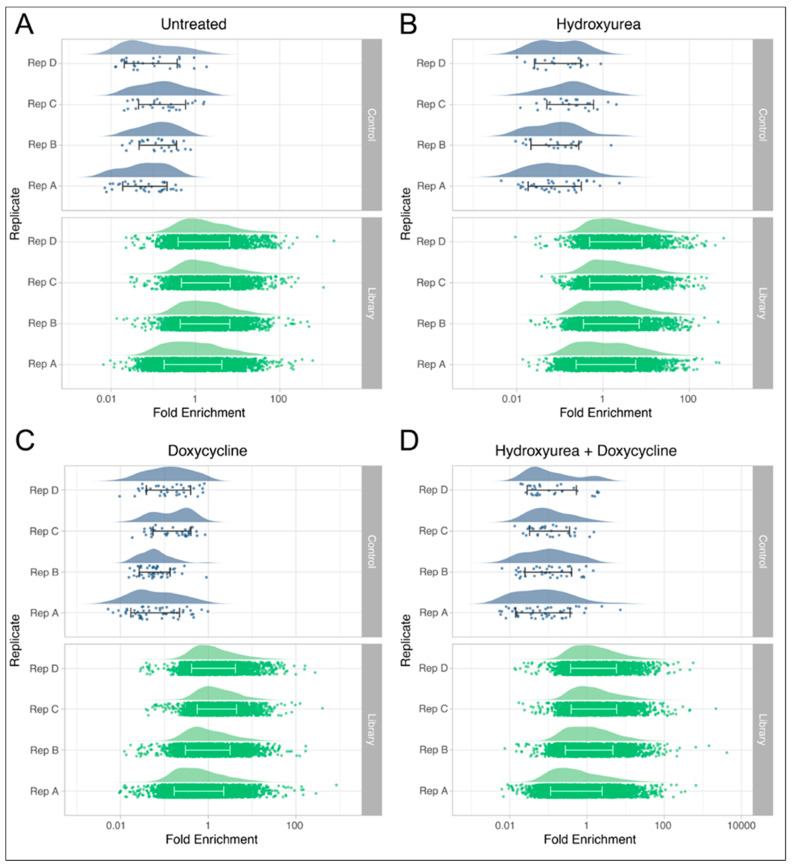
shRNA enrichment. shRNA enrichment over input for each replicate and each treatment following selection for ganciclovir resistance in DF/myc(CTG)_100_eGFP/TK cells, including non-targeting shRNA controls (upper panels) and the gene-targeting shRNA library (lower panels). The distribution of data for each replicate sample is shown above the bee swarm of individual data points. Within the swarm, the bars span from one standard deviation below to one standard deviation above the mean values for fold enrichment. (**A**) Control (untreated); (**B**) hydroxyurea; (**C**) doxycycline; and (**D**) hydroxyurea plus doxycycline treatments are as indicated.

To estimate the level of background noise in the system and eliminate irrelevant shRNAs that were detectable after selection, we used information from the NTCs. As expected, despite being detectable, 96.8% of the NTCs had enrichment values below 1.0, indicating a relative reduction in their presence compared to the original input virus pools. The gene-targeting shRNA enrichment levels were compared against the NTC enrichment levels to identify those that were significantly above the mean values calculated for the NTCs. The shRNA *p*-values were ranked and a false discovery rate (FDR) ≤ 0.001 [58,59] was applied to represent a significant level of enrichment above the background observed for the NTCs. This approach produced a list of over 1000 candidate loci from each treatment (Supplementary Table S2). The distributions of the NTCs and the gene-targeting shRNAs in each sample are depicted in Figure 2. Some shRNA types were listed by the manufacturer as not present in the pools before selection. The thirty-four shRNAs in this group were not included in subsequent quantitative analyses.

**Figure 3:**
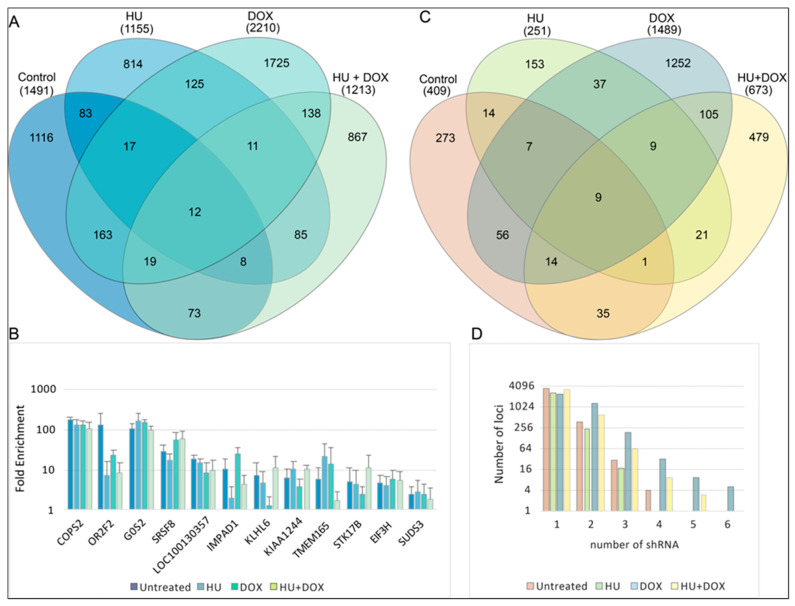
Treatment group comparisons in DF/myc(CTG)_100_eGFP/TK cells. The list of genes targeted by the lentiviral shRNAs was investigated. (**A**) Genes targeted and identified with a false discovery rate (FDR) = 0.001 [58,59] (see also Supplementary Tables S2 and S3). (**B**) Enrichment levels based on the sequence read counts before and after selection for the 12 genes found to be common to all treatments. (**C**) Genes targeted by two or more different shRNAs in a given treatment (see also Supplementary Table S4). (**D**) Number of loci with one or more shRNAs targeting each locus within each treatment category.

A total of 5200 unique shRNA hits are represented in Figure 3, representing approximately 3.4% of the input shRNAs. The candidate loci identified in each of the four types of treatment were compared (Figure 3A) (tabulated in Supplementary Table S3). While there was some overlap across treatments, the great majority of the loci that were targeted by the shRNAs were unique to each treatment. This not only suggested that there were few off-target effects in the screen but also indicated that each treatment represented a novel biological context and that different genes were required to prevent break-induced replication in each scenario. Despite the uniqueness of each treatment, twelve target genes were common across all four treatments (Figure 3B).

If multiple shRNAs targeted a given locus, this could serve as corroboration that the locus is an important suppressor of BIR in the context of a particular treatment. At a further enhanced level of stringency where two or more different shRNA sequences were represented in the sequence reads (independent of enrichment levels), nine of these twelve loci overlapped those targeted in the different treatments (Figure 3A,C). Nevertheless, a majority of loci were represented by a single shRNA sequence, even though each locus was potentially targeted by up to eight different shRNA sequences (Figure 3D). Fewer than 20% of the total candidate loci possessed multiple shRNA types whose enrichment levels met the FDR ≤ 0.001 criteria or had a Z-score ≥ 2 (Supplementary Table S4).

**Figure 4:**
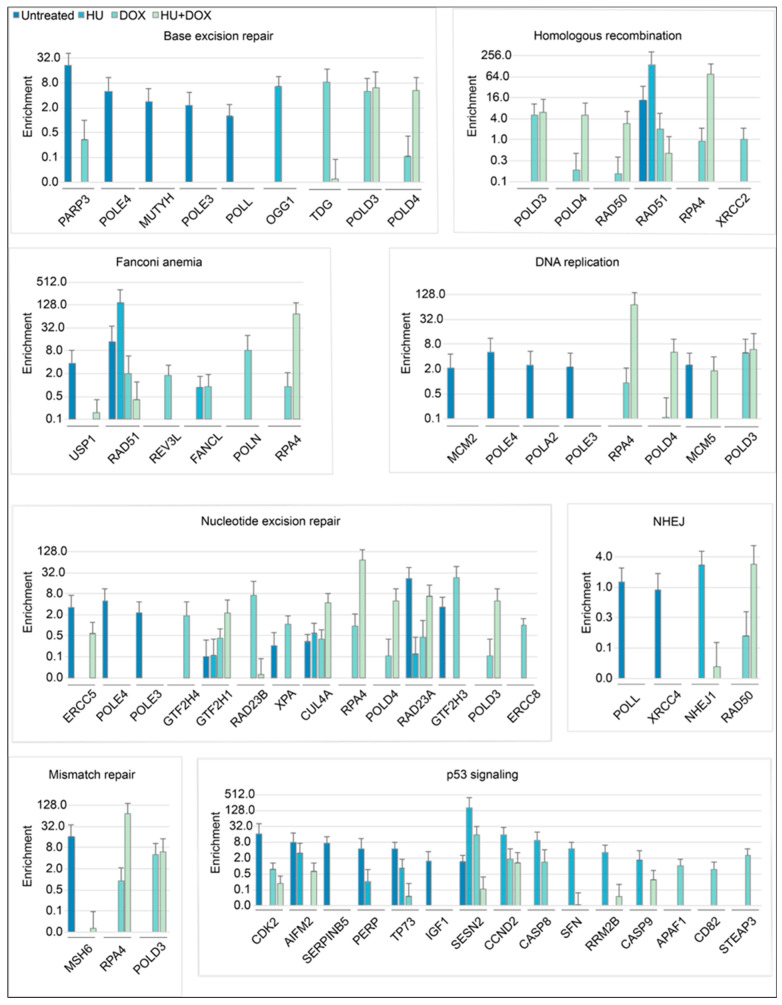
KEGG pathway loci involved in DNA replication, repair, and signaling that were detected in the shRNA screen. If more than one shRNA targeting the same locus was identified in a given treatment, the one with the highest enrichment is presented. The legend for the graphs is at the top left. (n = 4 replicates per treatment, FDR ≤ 0.001).

The candidate hits were examined to determine whether they were involved in pathways for DNA replication, recombination, or repair found in KEGG pathway datasets. Fifty-six unique loci were identified in the screen that corresponded to genes known in those pathways (Figure 4).

Hundreds of loci were identified in the screen that were not represented in the curated KEGG pathways that were examined. This suggested that many of the loci identified in the BIR screen may not be known to be involved in the regulation of DNA damage responses. To assess whether any of the candidate BIR loci could be further implicated in DNA damage and repair, we examined the same candidate loci by comparing them to genes identified in other screens for responses to ionizing radiation (IR, which can cause DSBs) and for proteins present at the sites of newly replicated (nascent) DNA. While there was some overlap between data sets for responses to ionizing radiation, or to proteins identified as being enriched at nascent DNA versus mature chromatin, the number of loci in common with those screens was fewer than ten percent of the total number of loci.

**Figure 5:**
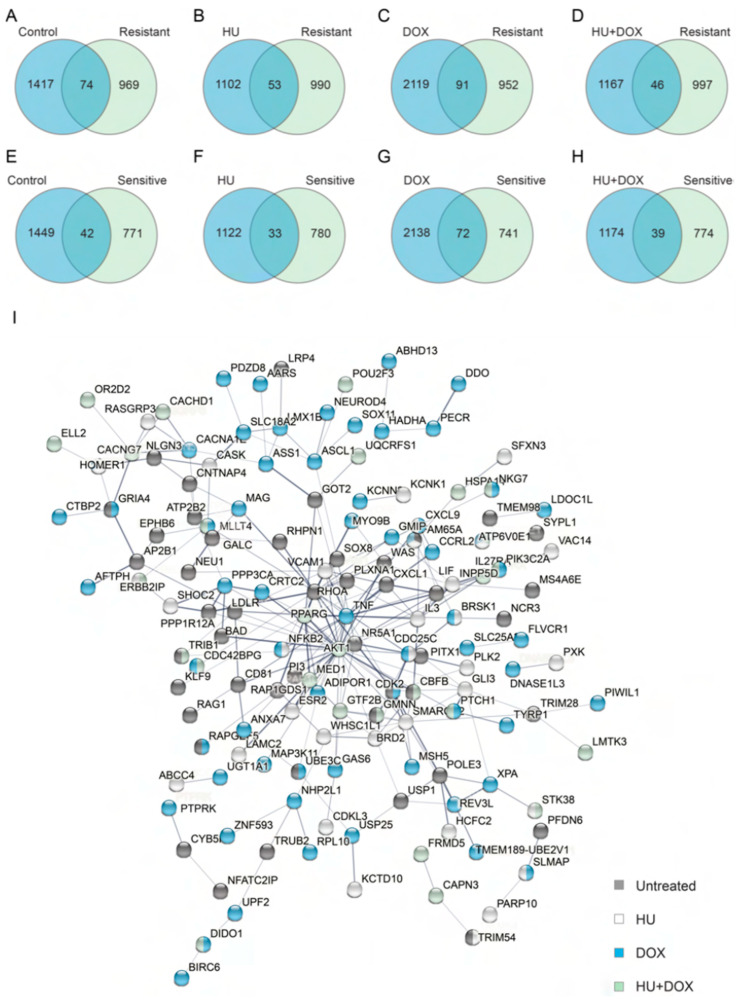

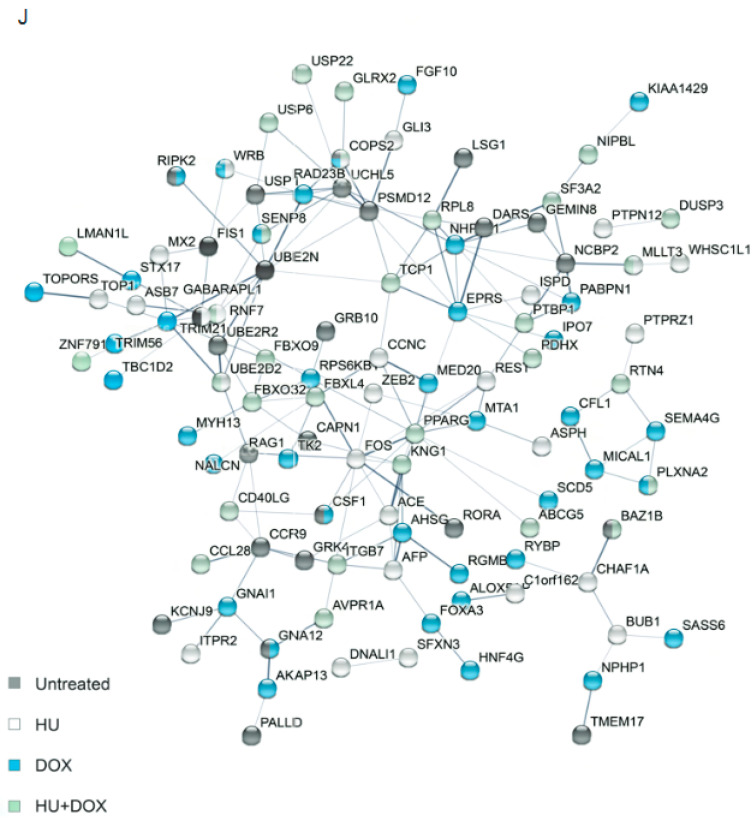
Comparison of loci in the BIR screen with loci identified in screens for ionizing radiation responses. (**A**–**D**) Overlaps between positive hits and loci that promote sensitivity to radiation; knockdown leads to cells becoming resistant to radiation. (**E**–**H**) Overlaps between positive hits and loci that block sensitivity to radiation; knockdown causes cells to become sensitive to radiation. ((**A**–**H**), see also Supplementary Table S5). (**I**) Networks formed from loci that promote sensitivity to radiation and were at the intersections of the four treatments. (**J**) Networks formed from loci that block sensitivity to radiation and were at the intersections of the four treatments.

Network analyses were performed on the sets of genes that overlapped with each of the treatments and loci from the screens on ionizing radiation sensitivity mentioned above (Figure 5A–D, Supplementary Table S5). Within a given treatment, 51–82% of the loci that overlapped with IR sensitivity genes were not directly connected to any other locus through known physical or genetic interactions. If the network was extended to a second shell to include indirect interactions, most loci were still without direct partners. However, if the loci from all four treatments were combined and analyzed as a group, a larger and more connected network was generated and a lower percentage (39–42%) of the loci remained unconnected (Figure 5E,F). In this context, AKT1, RHOA, and TNF were present at highly connected nodes within the network related to genes that promote sensitivity to ionizing radiation while NHP2L1, EPRS, FOS, UBE2N, and RAD23B were present at nodes within the network of genes that block sensitivity to ionizing radiation.

**Figure 6:**
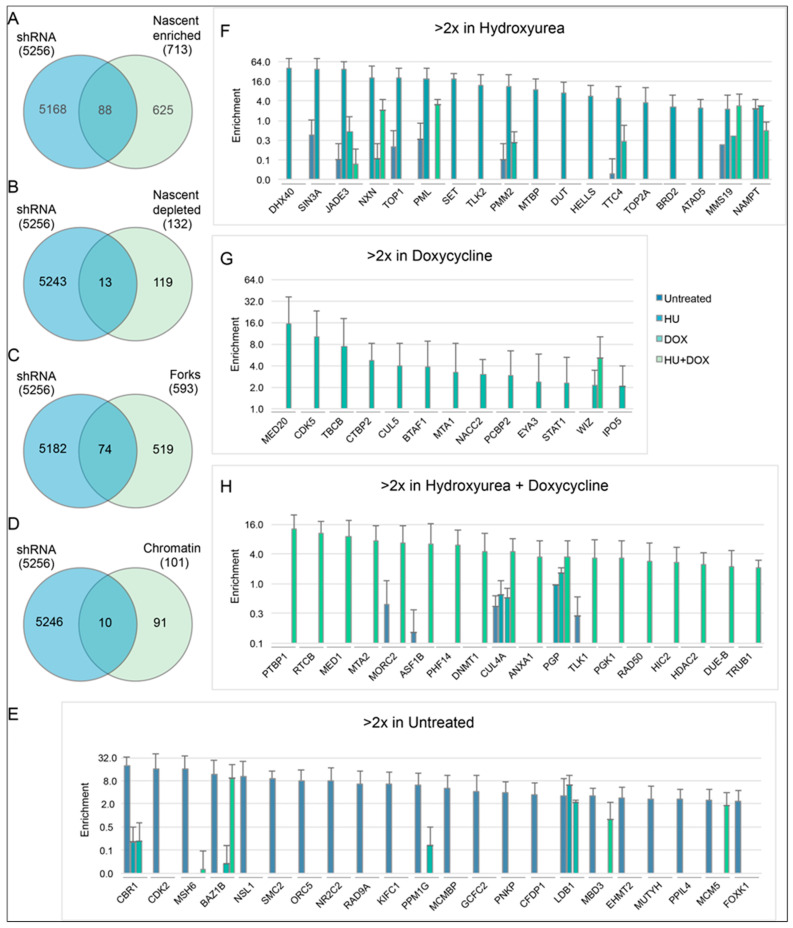
Loci corresponding to proteins present on nascent or mature DNA. (**A**–**D**) Venn diagrams for all shRNAs from all treatments in the screen compared with loci for proteins enriched or depleted at nascent DNA sites, enriched at replication forks, or enriched at mature chromatin. (**E**–**H**) Enrichment values of shRNAs with >2-fold enrichment in different treatments that correspond to proteins reported to be significantly enriched at the sites of nascent DNA. ((**A**–**H**), see also Supplementary Table S6). Most shRNAs were enriched only in one treatment. The legend for the graphs is to the right of panel (**G**).

**Figure 7:**
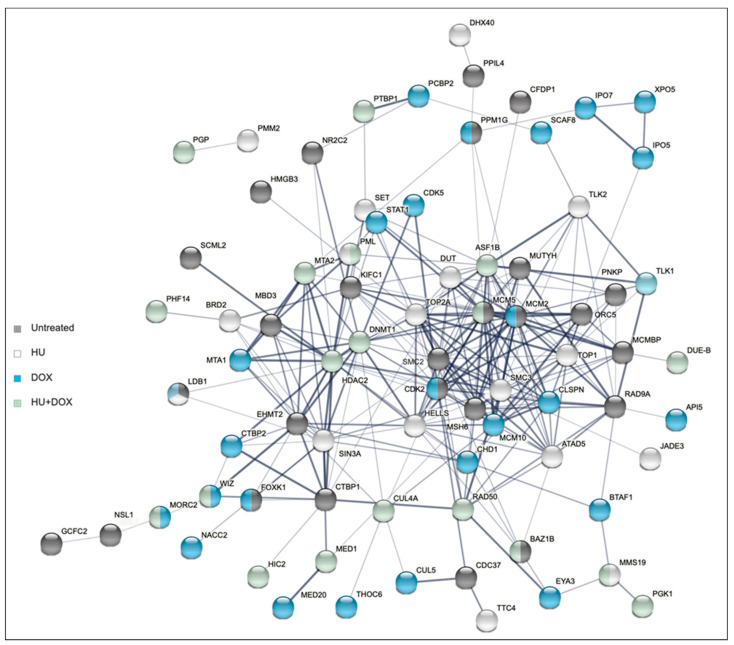
Network analysis for all positive hit loci with >2-fold shRNA enrichment whose proteins are enriched at sites of nascent DNA. Nodes are color coded by treatment. Edge thickness indicates the relative confidence in an interaction. Virtually 100% of the positive hit loci were part of an interconnected network but enriched in only one treatment.

Analyses of all hits showed only a slight enrichment for proteins present at sites of nascent DNA or replication forks (12%) [79,80] vs. those depleted from nascent DNA or bound to mature chromatin (10%) (Figure 6A–D, Supplementary Table S6). Loci that were enriched above two-fold and encoded proteins bound to nascent DNA were almost exclusively enriched in only one treatment (Figure 6E–H). Nevertheless, virtually all of the loci enriched >2-fold from all four treatments which overlapped with proteins bound to nascent DNA generated a highly connected network (Figure 7). Here, the histone chaperone ASF1B, the corepressors HDAC2 and SIN3A, the methylase of nascent DNA DNMT1, the replisome proteins MCM2, MCM5, and Claspin, and the structural maintenance of chromosomes proteins SMC2 and SMC3 were at network nodes. These comparisons suggested that while each of the treatments represented a different biological environment, each treatment elicited responses from the same networks associated with DNA lesions (intersecting with proteins in the IR response) or DNA replication (intersecting with the proteins on nascent DNA).

**Figure 8:**
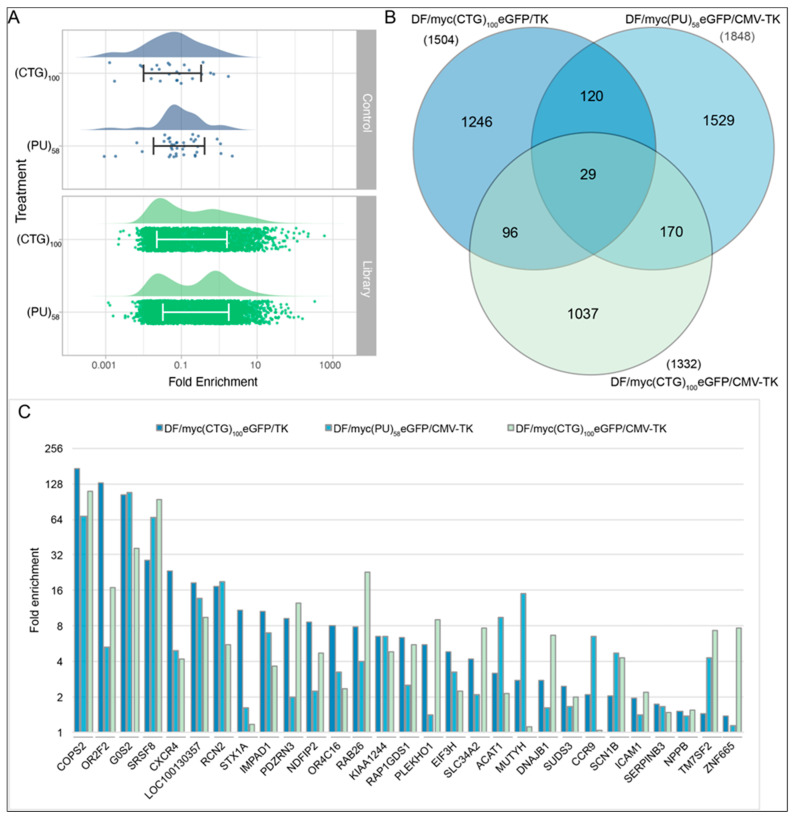
Enrichment of non-targeting shRNA controls and the gene-targeting shRNA library following selection for ganciclovir resistance in DF/myc(CTG)_100_eGFP/CMV-TK and DF/myc(PU)_58_eGFP/CMV-TK cell lines. (**A**) The distribution of data for each sample lies above the bee swarm of individual data points. Within the swarm, the bars span from one standard deviation below to one standard deviation above the mean values for fold enrichment. (**B**) Hits identified with a false discovery rate (FDR) = 0.001 (*p* < 0.001) in untreated cells from each of the three indicated cell lines. See also Supplementary Table S7. (**C**) Enrichment levels based on the sequence read counts before and after selection for the 29 genes found to be common to the three cell lines.

**Figure 9:**
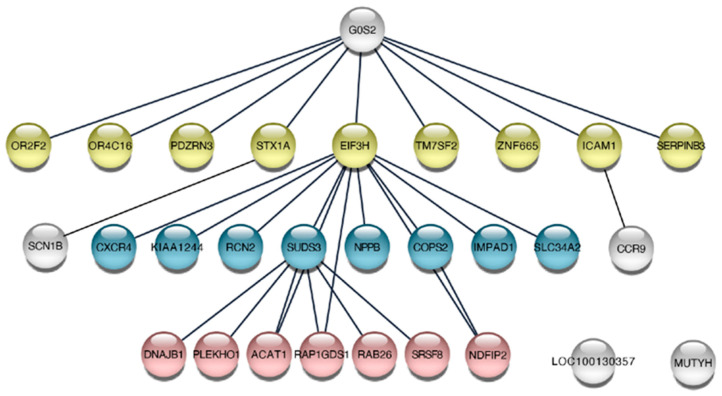
G0S2, EIF3H, SUDS3 master regulators. Regulatory hierarchy of twenty-nine shRNA hits common to DF/myc(CTG)_100_eGFP/TK, DF/myc(CTG)_100_eGFP/CMV-TK, and DF/myc(PU)_58_eGFP/CMV-TK cell lines.

Two additional non-B DNA cell lines (DF/myc(CTG)_100_/CMV-TK [52] and DF/myc(Pu/Py)_58_/CMV-TK [53]) were engineered and screened for suppressors of BIR in the untreated condition. The cell lines each possessed a different type of non-B DNA replication barrier ((Pu)_58_, (CTG)_100_) in the lagging strand template of DNA replication, in addition, the TK gene was driven by its own promoter instead of being part of a fusion protein. Again, non-targeting controls were present at low abundance after the selection process (Figure 8A). High stringency candidates (Z ≥ 2 in at least 3 replicates) identified in these two cell lines were compared with those identified in the untreated cells in the screen described above (Supplementary Table S7). While there were significant differences in the non-B DNA ectopic sites in these cell lines, there were similarities in the loci identified (Figure 8B). Most (>62%) of the BIR suppressors that were common to all three untreated cell lines had shRNA enrichment levels >2 fold in each cell line (Figure 8C). Network analysis of the twenty-nine hits common to the three non-B DNA cell lines showed that twenty-seven of the twenty-nine hits could be organized into a transcriptional (G0S2 [81,82], SUDS3 [83]) or translational (EIF3H-METTL3 [83]) regulatory hierarchy (Figure 9).

**Figure 10:**
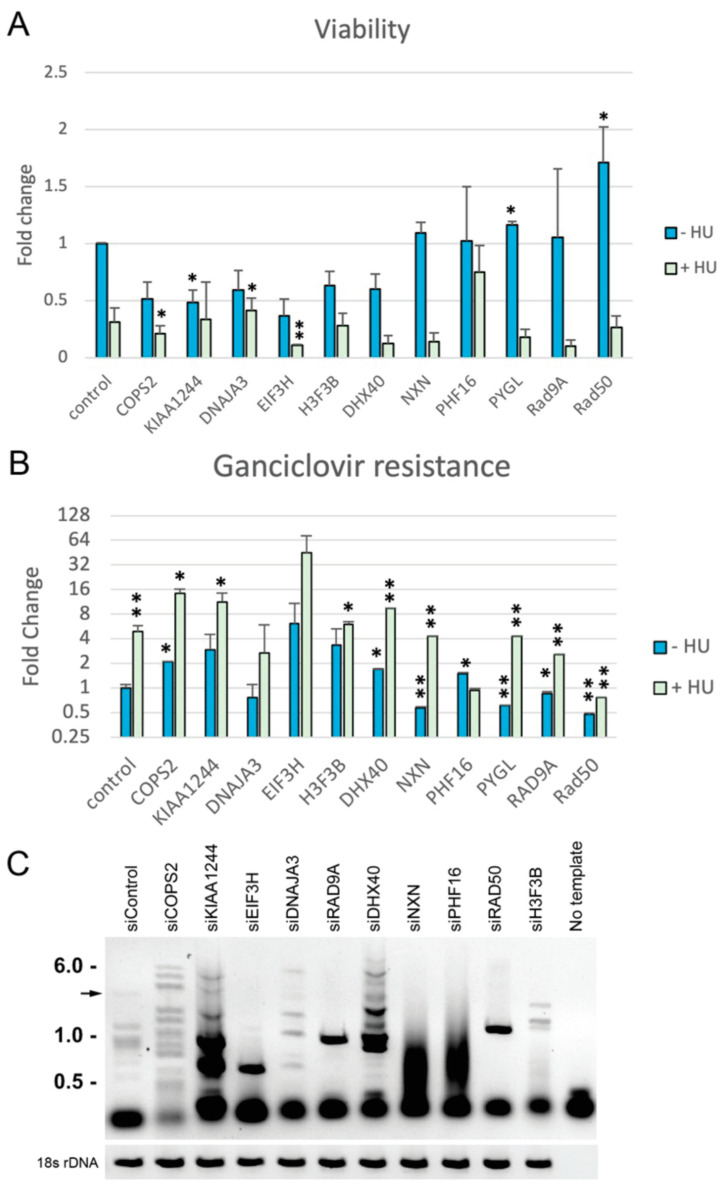
Effects of knockdown hits on cell viability, GCV^r^, and ectopic site stability. (**A**) Relative viability of cells treated with the listed siRNA and hydroxyurea (HU) compared to a non-targeting control in the absence of HU (see also Supplementary Table S1). * *p* < 0.05, ** *p* < 0.01, n = 2. (**B**) Ganciclovir resistant colony abundance (fold change) in siRNA-treated cells relative to cells treated with a non-targeting siRNA control in the absence of HU * *p* < 0.05, ** *p* < 0.01, n = 2. (**C**) iPCR of genomic DNA from cells treated with the listed siRNA. The arrow indicates the position of the expected 4 kb iPCR band from genomic DNA that has not undergone a rearrangement. The 18S rDNA was amplified separately as a test of the quality of each template for PCR and did not show rearrangements.

A number of candidate genes identified in the screen were selected for follow-up tests using individual siRNA knockdowns (Figure 10). Three candidates (COPS2, KIAA1244, EIF3H) were selected because they were identified in all four treatments from DF/myc(CTG)_100_eGFP/TK screens as well as in the DF/myc(CTG)_100_/CMV-TK and DF/myc(Pu/Py)_58_/CMV-TK cell lines, suggesting a core influence on BIR that is independent of context. The COPS2 signalosome subunit was highly enriched in GCV^r^ colonies in DF/myc(CTG)_100_eGFP/TK, as well as in the twenty-nine overlapping hits between DF/myc(CTG)_100_eGFP/TK, DF/myc(CTG)_100_/CMV-TK and DF/myc(Pu/Py)_58_/CMV-TK cells (Figure 8), and as a gene that blocks sensitivity to ionizing radiation (Figure 5). The CSN signalosome is implicated in multiple DNA repair pathways through deneddylation of CRL4^CDT2^ [84,85,86,87,88]. Histone H3F3B was chosen because this locus had at least two different shRNA types present in every treatment of the DF/myc(CTG)_100_eGFP/TK cells. RAD9A and DNAJA3 were chosen because they were present in every replicate of untreated DF/myc(CTG)_100_eGFP/TK cells, and DHX40 and PHF16/JADE3 were present in every replicate of HU treated DF/myc(CTG)_100_eGFP/TK cells. RAD50 was present in every replicate of HU + DOX treated cells and was identified as a protein enriched on nascent DNA.

Cells were treated with siRNA for each of these loci to determine their effects on cell viability and GCV resistance. Most of the siRNAs caused a reduction in cell viability under control conditions (Figure 10A), and all of these siRNAs decreased viability in the presence of replication stress induced by HU. Among the cells that survived, ganciclovir-resistant colonies were increased more than two-fold in untreated cells exposed to siRNA targeting COPS2, KIAA1244, EIF3H, and H3F3B. In all cases, HU treatment increased ganciclovir resistance in cells treated with a non-targeting control siRNA or targeting siRNA. In the presence of HU, 4 of 10 siRNAs caused a ≥2-fold increase over control siRNA in GCV^r^ colonies (Figure 10B), consistent with the selected siRNAs knocking down suppressors of BIR.

Inverse PCR initiating at the non-B DNA ectopic site was used as an orthogonal assay of DNA instability. iPCR has been used to demonstrate that rearrangements and other mutations occur at the ectopic site in DF/myc(CTG)_100_eGFP/TK and related cells that have undergone BIR [52,53,60]. Genomic DNA was isolated and cleaved with XbaI. Following dilution and circularization of the DNA, iPCR of the DNA from cells treated with the non-targeting control siRNA showed the anticipated 4 kb fragment as well as a few additional bands which reflect the intrinsic instability of the (CTG)_100_ repeat under unperturbed growth conditions (Figure 10C). siRNAs targeting the selected loci showed changes from the pattern seen in non-targeting control siRNA-treated cells. Consistent with an increase in genomic rearrangements caused by BIR, multiple additional bands above a diffuse background of iPCR products were observed in cells treated with siRNA targeting COPS2, KIAA1244, DNAJA3, DHX40, and H3F3B. siRNA targeting EIF3H, RAD9A, and RAD50 produced a single prominent band, while the expected genomic DNA fragment corresponding to the unrearranged ectopic site was not detected. siRNA targeting NXN and PHF16/JADE3 did not produce distinct bands; instead, each induced a collection of lower molecular weight recombination products. The distinct arrays of iPCR products suggest that each of the siRNAs alters the structure of the replication fork towards a different pattern of BIR recurrent recombinations in the DF/myc(CTG)_100_eGFP/TK cells.

We further tested the effects of knockdown of COPS2 on another unstable microsatellite, (ATTCT), derived from the ATXN10 locus. Expansion of this pentanucleotide leads to spinocerebellar ataxia type 10 (OMIM #603516), and replication-dependent instability [61]. The (ATTCT)_47_ microsatellite was integrated into the same chromosomal ectopic site as previously, in the DF/myc(ATTCT)_47_eGFP/TK cells, which were treated with control or COPS2 siRNA. DNA was prepared for iPCR and the products were subjected to long-read HiFi PAC Bio sequencing.

**Figure 11:**
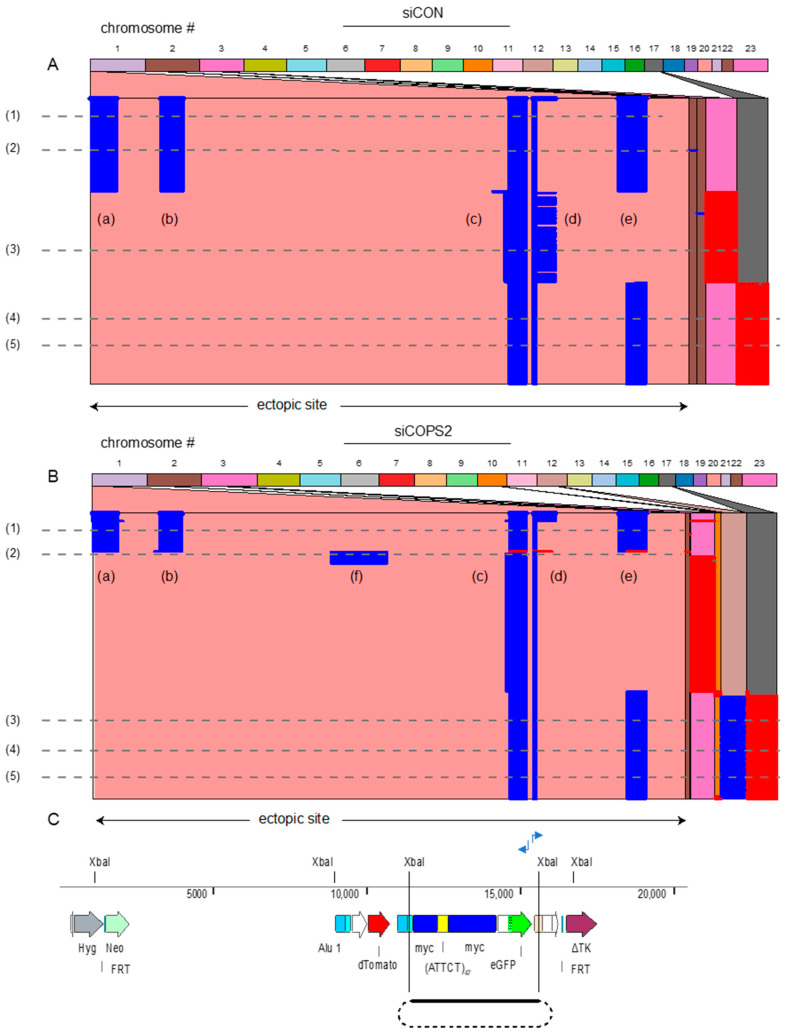
COPS2 knockdown increases genomic instability at the (ATTCT)_47_ ectopic site. Panel (**A**) contains 14,070 unique deduplicated reads derived from cells treated with siCON; panel (**B**) contains 13,501 unique deduplicated reads derived from cells treated with siCOPS2. Letters (a)–(f) refer to template switching domains. (**C**) schematic of the ectopic site aligned with the Ribbon [89] maps of (**A**,**B**). Domain (d) comprises sequences initiated at the forward iPCR primer; domain (**C**) contains the reverse complement of sequences initiated at the reverse iPCR primer. Dashed lines (1)–(5) are reads that are detailed in Supplementary Figures S1–S3. The positions of the forward and reverse iPCR primers are shown. Read segments in red are in the reverse orientation to the standard short-long arm chromosome orientation. The ectopic site is shown below the alignment; nonallelic chromosome templates are shown above the alignment. (**C**) Schematic of the ectopic site aligned with the Ribbon maps of (**A**,**B**); including the predicted ectopic site XbaI-XbaI circle template for iPCR.

In order to avoid reads generated by off-target binding of the iPCR primers, the raw data were filtered to select for reads containing the forward primer and reverse primer complement sequences at the ends. To filter out multiple reads from the same template due to iPCR, the raw sequences were deduplicated, thus each read displays a unique set of mutations. Figure 11 shows the analysis of the sequencing reads using Ribbon [89], a visualization tool which shows how alignments are positioned within genomic/ectopic site reference and read contexts, to enable an understanding of complex structural variants.

iPCR of DF/myc(ATTCT)_47_eGFP/TK cell DNA yielded 14,070 unique reads which aligned with five well-defined domains of the ectopic site (Figure 11A, (a)–(e)). Remarkably, domains (a), (b), and (e) lie outside the circularized Xba1 fragment template (aligned in Figure 11C). This indicates that the template-switching events are complex, but not random. Approximately two-thirds of the reads from DF/myc(ATTCT)_47_eGFP/TK cells treated with siCON show template switching ranging from 250 bp to 1 kbp, to chromosome 2, 3, or 17, before returning to the ectopic site. Detailed views of the rearrangements in several reads (Figure 11, lines (1)–(5)) are shown in Supplementary Figure S1. Although a large percentage of the reads share the same pattern of template switching, each read exhibits a unique pattern of indels and base substitutions (Supplementary Figure S2).

**Figure 12:**
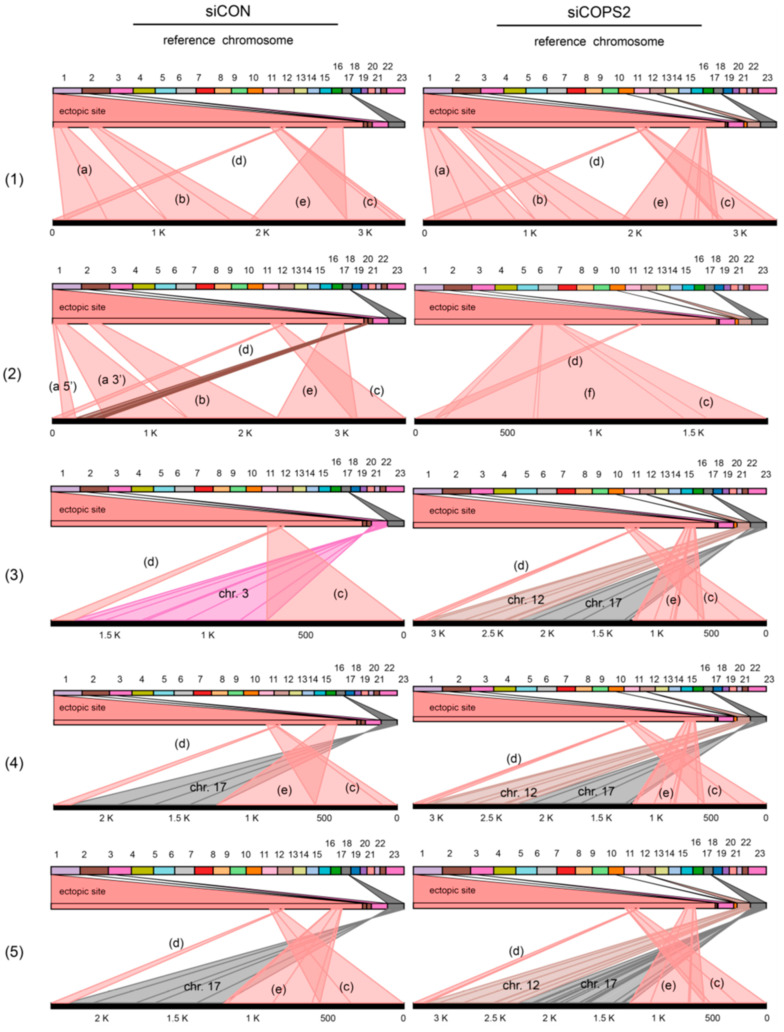
COPS2 knockdown increases template switching. Template switching in DF/myc(ATTCT)_47_ cells treated with siCON (left) or siCOPS2 (right). Reference chromosomes are shown at the top. Segments of the ectopic site engaged in template switching extend from the wedge below the reference chromosomes. Template switching domains (a)–(f) are as in Figure 11. Darker lines within a domain indicate indels. The black bar at the base of each diagram represents the sequence read. Lines (1)–(5) in Figure 12 refer to lines (1)–(5) in Figure 11.

In cells treated with siCOPS2 (Figure 11B) domains (a)–(e) persisted as regions of preferential template switching. However, in contrast to reads from control cells, domain (d) was narrower in approximately one-third of the reads, and a new domain of template switching (domain (f)) appeared. Approximately 67% of reads contained single template switches to chromosomes 2, 3, or 17 in cells treated with siCON, whereas approximately 87% of cells knocked down for COPS2 showed template switches to these chromosomes; in addition, COPS2 knockdown-induced template switching to chromosome 12 (Figure 12). Remarkably, all of the reads showing template switching to chromosome 12 also showed a second template switch to chromosome 17, whereas none of the template switches in siCON treated cells involved multiple chromosomes.

The number of insertion mutations that had accumulated in cells treated with siCON or siCOPS2 was extremely high (~1 × 10^−3^/bp (Supplementary Table S8)). Although siCOPS knockdown increased the number of deleted nucleotides by 21% (to 1.15 × 10^−3^/bp), and the number of mismatch mutations by 12% (to 2 × 10^−4^/bp), differences in the levels of indels or mismatches between siCON and siCOPS2 knockdowns did not achieve statistical significance.

However, it is important to recognize that these totals do not distinguish between the high background of mutations that occurred before siCOPS2 treatment or prior to deletion of the (ATTCT) repeat vs. mutations that occurred due to COPS2 knockdown.

Inspection of the mutational signatures in COSMIC (https://cancer.sanger.ac.uk/signatures/sbs/ (accessed on 14 January 2023)) following siCON and siCOPS2 treatment (Supplementary Figure S4) showed a significant increase in C>A mutations (SBS36), characteristic of depletion of the MUTYH glycosylase which removes oxidized G residues and repairs A:G mismatches (see Discussion).

In addition, siCOPS2 treatment resulted in the appearance of mutagenic hotspots within 50 bp of specific d(T)_29_ and G4 consensus sequences (Supplementary Figure S3), although not all d(T) repeats or G4 consensus sequences were hotspots. In contrast, COPS2 knockdown revealed a new domain of template switching (domain (f)) and increased nonallelic template switching, particularly double template switching within a single read. Considered together, these data suggest that the knockdown of COPS2 has effects on replisome stability, fork remodeling, and replisome mutagenesis.

## 4. Discussion

We screened an ultracomplex genomic shRNA lentivirus library to identify suppressors of break-induced replication. Three cell lines that contain non-B forming DNA at a single ectopic site were transduced with the library, followed by ganciclovir selection for mutagenesis of the resident viral thymidine kinase gene. We have previously shown that DSBs and BIR induce local mutagenesis and gross chromosomal rearrangements at the ectopic sites dependent on the presence of non-B DNA forming microsatellite repeats [52,53,60].

The primary cell line used in the screen, DF/myc(CTG)_100_eGFP/TK, has been shown to form hairpin structures in vivo [63]. DF/myc(CTG)_100_eGFP/TK cells were transduced with the shRNA library in the absence of additional replication stress (untreated), or in the presence of low-dose hydroxyurea (HU). These cells also contain an integrated shRNA targeting the third subunit of DNA polymerase delta (POLD3) under the control of a doxycycline-inducible promoter [52] and were transduced with the library in the presence of doxycycline (DOX) and in the presence of doxycycline plus low dose hydroxyurea (DOX + HU). Each of the four treatment conditions was performed in quadruplicate.

*p*-values were calculated for the enrichment over the input of more than one million shRNA reads from the biological replicates for each treatment. The *p*-values were stratified and a false discovery rate (FDR) ≤ 0.001 [58,59] was chosen as the threshold of significance. This threshold equated closely to shRNAs whose enrichment exceeded the mean enrichment of NTC shRNAs by ≥2 standard deviations (Z ≥ 2) in at least three of four biological replicates for a single treatment.

More than 1400 shRNA-targeted loci met the threshold criteria under untreated (control) conditions, with fewer than 10% of loci targeted by more than one shRNA. Similarly, large numbers of hits (1000–2000) were observed under HU, DOX, and HU + DOX conditions, with ~10–20% overlap between conditions (Figure 3). These results suggest that an unexpectedly large number of loci act to mitigate repeat-induced mutagenesis and replication stress or induce replication stress when knocked down and that beyond a core of stabilizing pathways, each treatment condition represented a unique environment of replication stress. These results also suggest that BIR-like repair can occur when POLD3 has been depleted.

The twelve hits that overlapped and showed consistent enrichment across all treatments (Figure 3B) included COPS2, a subunit of the COP9 (CSN) signalosome that regulates DNA repair and translesion polymerase binding to PCNA through deneddylation of CRL4^CDT2^ [84]; G0S2, a tumor suppressor [82,90] and oncogene [91] which blocks PIK3/mTOR signaling, oncogene-induced transformation and the anti-apoptotic function of the Bcl-2/Bax complex [81,82,92]; SRSF8, which binds to the ATM (Ataxia Telangiectasia Mutated) kinase [93]; SUDS3, a subunit of the Sin3/HDAC corepressor complex [94]; and the translation regulator EIF3H, which binds to METTL3 to promote translation of a large subset of oncogenic mRNAs [95]. More than 18% of the shRNA hits are upregulated >2× by EIF3H-METTL3 at the level of translation, including SUDS3, SRSF8, COPS2, IMPAD1, STK178, TMEM165, and KLHL6. Network analysis of the hits from the four treatments emphasizes the hierarchy of regulation by G0S2, EIF3H, and SUDS3 (Figure 10) and the connectivity between the hit loci, despite the modest overlap between hits from different treatments.

Several of the hits from the screen could be directly assigned to KEGG pathways for DNA metabolism and DNA damage signaling downstream of p53 (Figure 4) (p53 itself is inactivated by HPV18 E6 in the HeLa non-B DNA reporter cells [96]), and a large number of the screen hits have been implicated by others in sensitization or resistance to DNA damage by ionizing radiation (Figure 5). Interestingly, the total shRNA hits are enriched for loci coding for proteins bound to nascent DNA or replication forks vs. proteins depleted at nascent chromatin or bound to mature chromatin [79,80] (Figure 6A–D), in line with the view that the shRNA hits are involved in replisome stabilization or DNA damage signaling. Moreover, when the hits from individual treatments were compared to the proteins bound to nascent DNA, virtually no hits/proteins appeared from other treatments (Figure 6E–H). Although this analysis shows a limited number of hits coding for proteins bound to nascent DNA or replication forks which are identical between treatments, network analysis of the total shRNA hits that code for proteins bound to nascent DNA (Figure 7) reveals a matrix of candidates that protect against replication stress. This observation reinforces the conclusion that each treatment reflects a unique replication stress environment.

We repeated the lentivirus shRNA screen in quadruplicate under untreated conditions on two additional cell lines that were independently derived. The DF/myc(PU)_58_eGFP/CMV -TK line contains part of the asymmetric Pu/Py repeat from the PKD1 IVS21 locus [53], and the DF/myc(CTG)_100_eGFP/CMV-TK line places the HSV-TK gene under the CMV promoter, separate from the eGFP fusion gene. As these lines were independently derived and expanded after clonal isolation, they are expected to display idiotypic patterns of ectopic site instability [24,52,53,60,61,65,78] and responses to replication stress. Nevertheless, the overlap between these cell lines was approximately double the overlap between the four different treatments of the DF/myc(CTG)_100_eGFP/TK line (Figure 8). We note as well that twenty-seven of twenty-nine hits that overlap between untreated DF/myc(CTG)_100_eGFP/TK, DF/myc(CTG)_100_eGFP/CMV-TK, and DF/myc(PU)_58_eGFP/CMV-TK cells (Figure 8C) encode genes in the G0S2-EIF3H-SUDS3 regulatory hierarchy (Figure 9).

In order to test the validity of the shRNA screen, individual siRNAs were designed against shRNA loci that showed consistent enrichment in various treatments and cell lines. Consistent with these loci encoding suppressors of BIR, iPCR across the ectopic site of DF/myc(CTG)_100_eGFP/TK cells [52,53], each of the cognate siRNAs generated different forms of instability. These data strongly suggest that the knockdown of the corresponding gene products identified in the shRNA screen resulted in ectopic site mutagenesis. We stress that each of the siRNA knockdowns produced a distinct pattern of bands, from which we conclude that different perturbations of the replication fork result in distinct but reproducible patterns of recurrent recombination.

It has been estimated that no fewer than 450–600 human proteins respond to replication stress via the S- phase DNA replication checkpoint (DRC) and the DNA damage response (DDR) pathways [79,97]. Our data suggest that as many as 5000–6000 genes may impinge on diverse aspects of the replication process. While the depletion of different proteins sensitizes cells to different forms of stress, these candidates comprise a diffuse network directed toward replication stabilization.

We have shown that recombination of the CTG and homoPu/homoPy repeats is dependent on MUS81 nuclease, and that mutagenesis is bidirectional from the microsatellites [52,53], which distinguishes our model from alternative homology-dependent repair pathways. Nevertheless, the large number of hits raises the possibility that our screen has detected variants of other pathways that could result in TK mutagenesis. Indeed, recombination-dependent replication [56,98], single-strand annealing, and synthesis-dependent strand annealing have been modeled to repair replication-dependent DSBs [44,45].

When the effect of the knockdown of several candidates was tested by iPCR, it was consistently observed that specific bands were generated instead of a completely random smear of product sizes. This suggests that under control or perturbed conditions, there are recurrent template switches that lead to recombination and apparent chromosome translocations during repeat-mediated instability in DF/myc(CTG)_100_ cells. This was confirmed in the case of control or COPS2 knockdown in DF/myc(ATTCT)_47_ cells where single molecular analysis showed distinct domains of template switching. Moreover, each domain exhibited high levels of mutagenesis for their common template-switching domains A–E, although the level of mutagenesis between siCON and siCOPS2 did not reach statistical significance.

However, siCOPS2 led to a significant increase in the C>A mutational signature (SBS36) of MUTYH depletion. Interestingly, access of MUTYH to A:G mispairs is regulated by the CUL4B ubiquitin ligase [99,100], that is in turn inactivated by the COP9 deneddylase.

COPS2 knockdown also led to a new, domain of template switching (domain (f)), increased nonallelic template switching, and double template switching within a single event. From this, we suggest that COPS2 knockdown leads the perturbed replication fork to adopt alternative folding configurations, alters the stability of the repair replisome, and modifies BIR mutagenesis.

## Data Availability

Primary data are available in the or upon request from the authors.

## Supplementary Materials

The following supporting information can be downloaded at: https://www.mdpi.com/article/10.3390/genes14020398/s1, Figure S1: COPS2 Western blot, sequence alignments; Figure S2: Mutation quantitation maps; Figure S3: Schematics of rearrangements; Figure S4: Mutation signatures. Table S1: siRNAs; Table S2: Candidate p values; Table S3: Hits in each of the 4 treatments; Table S4: Unique/Multiple Hits.; Table S5: Overlap hits between treatments and radiation resistance or sensitivity; Table S6: Overlap between hits and proteins bound or depleted at nascent chromatin; Table S7: Overlapping Hits between Cell Lines; Table S8: Mutation quantitation.
