# Supplementary material for: Suppressors of Break-Induced Replication in Human Cells
Source: Genes (Basel). 2023 Feb 3;14(2):398. doi: 10.3390/genes14020398 (PMC9956954; doi:10.3390/genes14020398)
Supplement: Supplementary file 1 [file genes-14-00398-s001.zip › Supplementary TableS1, S2, S4-S8; Figures S1-S4.pdf]

Supplementary Figure S1: COPS2 Western blot, sequence alignments

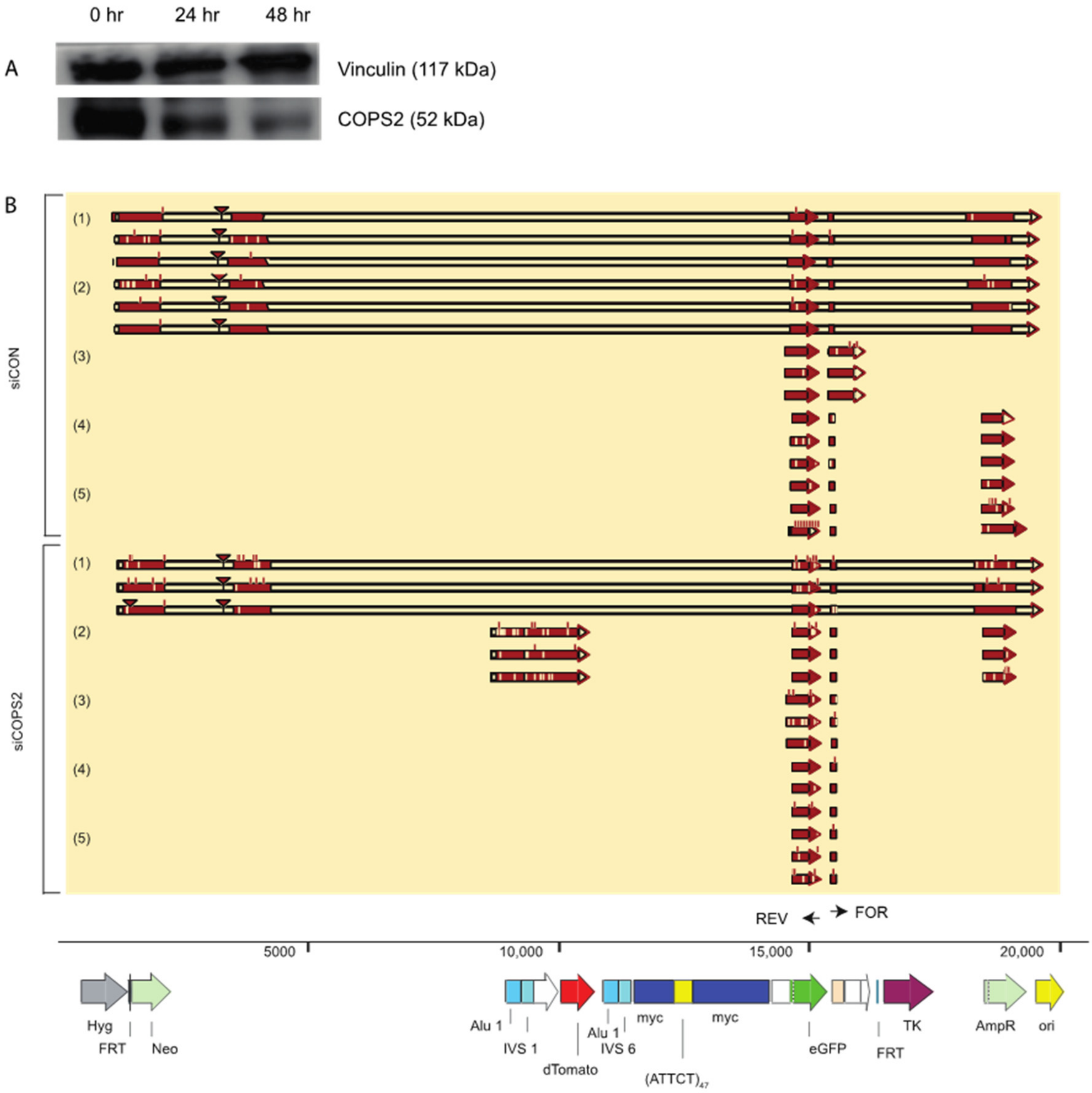

Supplementary Figure S2: Mutation quantitation maps.

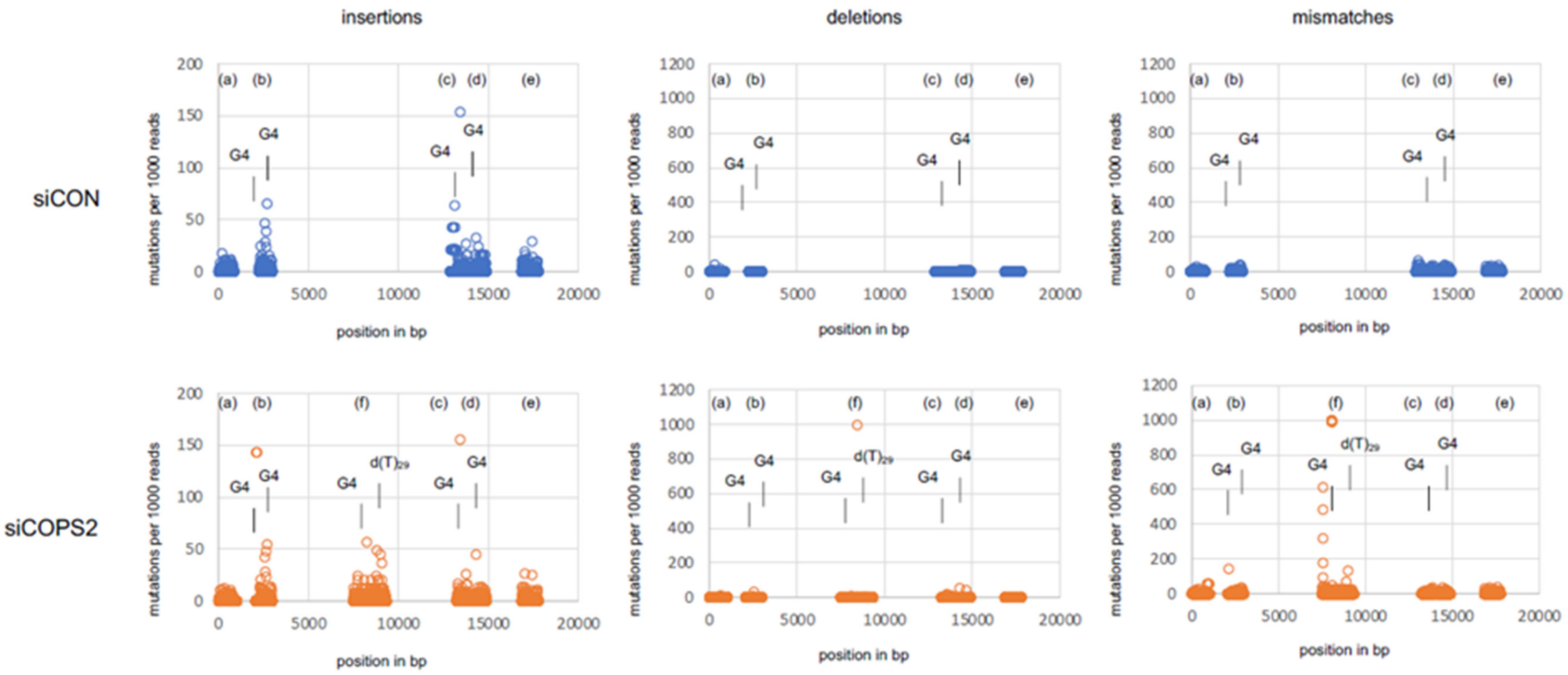

Supplementary Figure S3: Schematics of rearrangements.

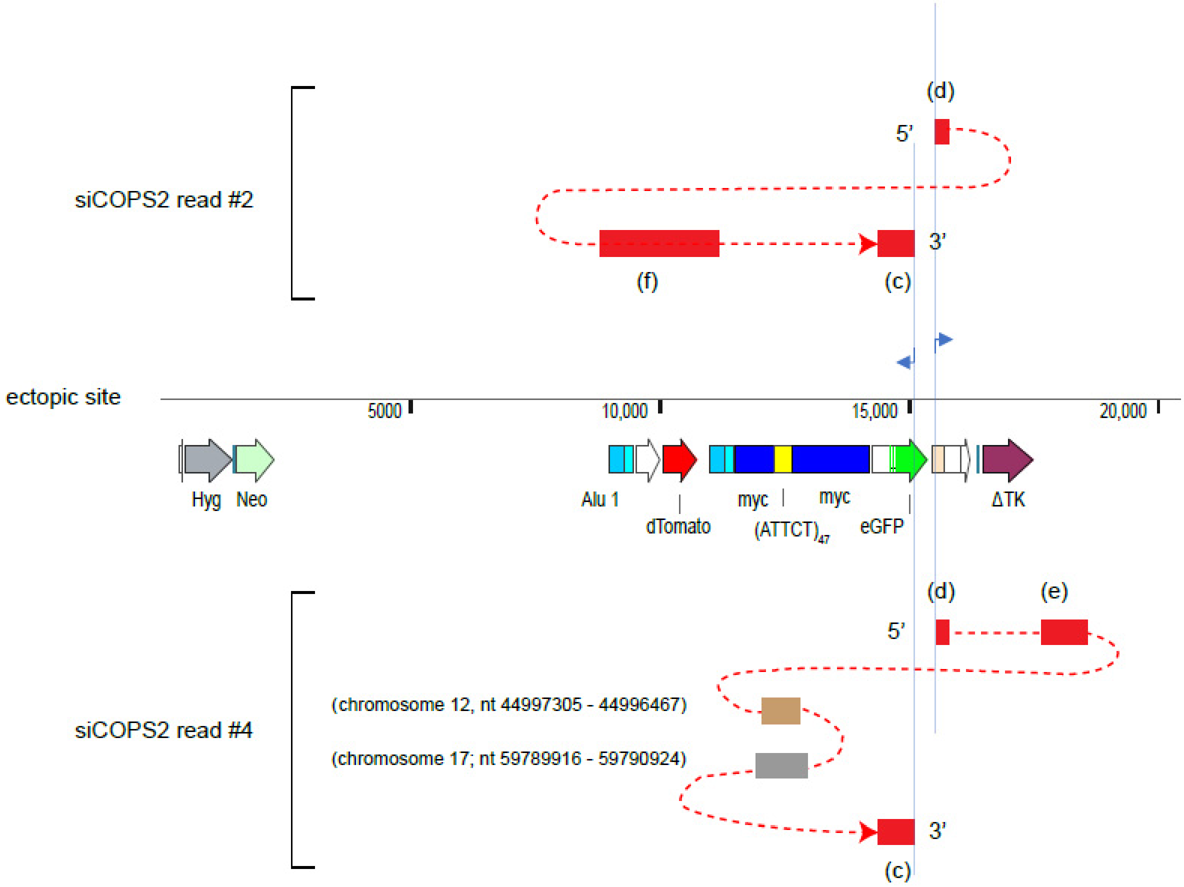

Supplementary Figure S4: Mutation signatures. Analysis of transitions and transversions from cells treated with siCON or siCOPS2 are indicated. Error bars indicate SD. *p* values were derived using Student's t-test tailed.

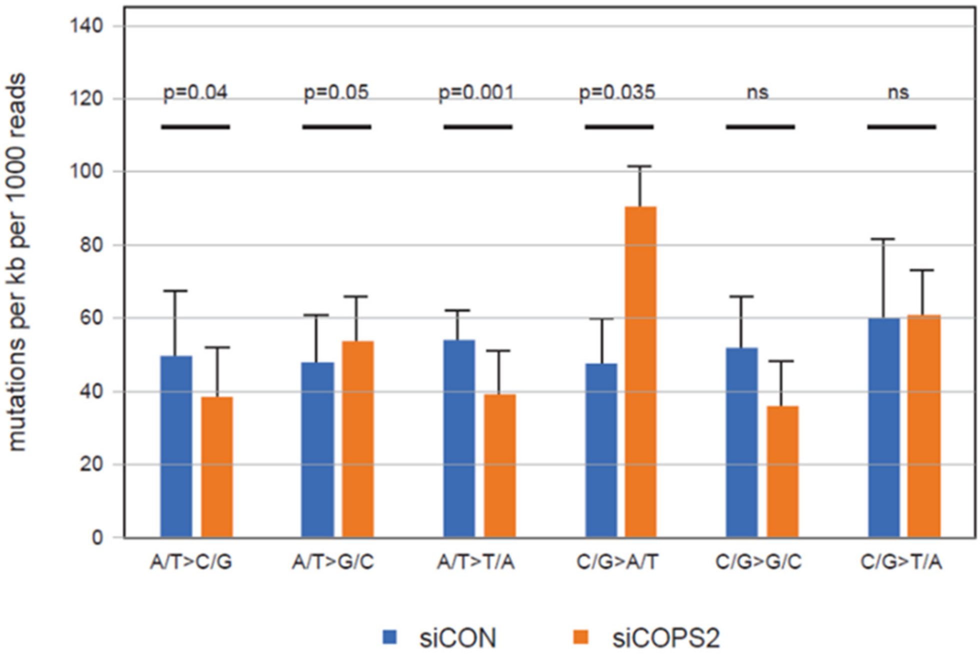

Supplementary Table S1: siRNAs

siRNAs re- Figure 10

Locus	siRNA	Provider	Sequence
COPS2	s17803	Ambion	CGAUAUACUGCACUAGAUATT
KIAA1244	s32912	Ambion	CCAGGUCUUUGCUAAUGCATT
DNAJA3	s17346	Ambion	GAAAGCCUAUUAUCAGCUUTT
EIF3H	s16508	Ambion	GAGGAAUACUAGUAAACAATT
H3F3B	s6424	Ambion	GACUGCACUUGUUCUCAUATT
RAD9A	a11721	Ambion	GACAUUGACUCUUACAUGATT
PYGL	s534288	Ambion	GGUACGAGGCAAAAGAAUATT
PHF16	s18860	Ambion	GGUCAAUGUAUAGGAUCATT
NXN	s34657	Ambion	CAACAUUCCAUCACUAAUATT
DHX40	s36047	Ambion	GAACUAAUCAGGAAGCUUATT
Rad50	s792	Ambion	GGAAUAGACUUAGAUCGAATT
control #1	4398043	Ambion	(undisclosed)

Supplementary Table S2: Candidate p values

		Benjamini-Hochberg FDR = 0.001 Adjusted p-value cut-offs								
		Control	HU	Dox	HU+Dox					
		7.972E-04	7.619E-04	8.124E-04	5.624E-04					
		adjusted p values					shRNA candidates p < FDR			
Clone ID	Gene ID	Control	HU	Dox	HU+Dox		Control	HU	Dox	HU+Dox
V3SVHSHC_9290615	COPS2	6.050E-88	5.015E-54	2.387E-91	3.412E-73		COPS2	COPS2	COPS2	COPS2
V3SVHSHC_9153236	LOC100130357	9.336E-60	8.387E-53	5.484E-38	8.807E-30		LOC100130357	LOC100130357	LOC100130357	LOC100130357
V3SVHSHC_10728788	OR4M2	2.000E-57					OR4M2			
V3SVHSHC_4664345	DCUN1D2	5.163E-52		1.261E-13			DCUN1D2		DCUN1D2	
V3SVHSHC_5641277	NLRP2	5.583E-50					NLRP2			
V3SVHSHC_6017675	SRSF8	7.426E-49	1.189E-36	2.120E-57	8.409E-49		SRSF8	SRSF8	SRSF8	SRSF8
V3SVHSHC_5416877	G0S2	2.128E-47	8.097E-34	2.845E-111	5.503E-86		G0S2	G0S2	G0S2	G0S2
V3SVHSHC_9074597	DALRD3	9.136E-47					DALRD3			
V3SVHSHC_7846700	RPUSD1	3.133E-42					RPUSD1			
V3SVHSHC_9595172	FOXG1	3.576E-41					FOXG1			
V3SVHSHC_8315234	MROH5	5.446E-41					MROH5			
V3SVHSHC_5401961	SMC2	1.172E-37					SMC2			
V3SVHSHC_6748823	ZNF418	2.457E-37					ZNF418			
V3SVHSHC_9494588	FAT3	1.027E-36					FAT3			
V3SVHSHC_4804892	LOC101927859	2.902E-36					LOC101927859			
V3SVHSHC_6205709	MTNR1B	5.678E-36					MTNR1B			
V3SVHSHC_10036910	LCP2	1.713E-35					LCP2			
V3SVHSHC_8882504	AQP11	5.627E-35					AQP11			
V3SVHSHC_5471657	PIGY	8.546E-34					PIGY			
V3SVHSHC_7556234	DND1	1.837E-33					DND1			
V3SVHSHC_10661006	SMARCD2	4.078E-33					SMARCD2			
V3SVHSHC_9611969	RHOF	7.252E-33					RHOF			
V3SVHSHC_10773371	ANGPTL4	2.134E-32					ANGPTL4			
V3SVHSHC_7307810	C1QL3	2.512E-32					C1QL3			
V3SVHSHC_9720176	MAGEB1	3.678E-32					MAGEB1			
V3SVHSHC_8914448	CCL14	9.762E-32					CCL14			
V3SVHSHC_7638899	GLTSCR1L	1.763E-31		3.358E-07			GLTSCR1L		GLTSCR1L	
V3SVHSHC_5420639	KCNE2	2.051E-31					KCNE2			
V3SVHSHC_5428757	CCDC64	3.210E-31					CCDC64			
V3SVHSHC_5567852	HYPK	4.191E-31					HYPK			
V3SVHSHC_8724698	KIAA1244	6.857E-31	1.266E-32	2.106E-40	2.197E-50		KIAA1244	KIAA1244	KIAA1244	KIAA1244
V3SVHSHC_6011372	PCYT1A	8.094E-31					PCYT1A			
V3SVHSHC_6506372	CBR1	1.080E-30					CBR1			
V3SVHSHC_10706018	BDH2	1.105E-30					BDH2			
V3SVHSHC_8309459	LRP4	2.061E-30					LRP4			
V3SVHSHC_6582041	LOC101930355	3.102E-30					LOC101930355			
V3SVHSHC_5116709	LOC101928268	4.366E-30					LOC101928268			
V3SVHSHC_10463171	CCL25	5.689E-30					CCL25			
V3SVHSHC_8269628	PIK3C3	1.083E-29					PIK3C3			
V3SVHSHC_6909401	FAM71E2	1.385E-29					FAM71E2			

V3SVHSHC_7076447	CCDC64	1.542E-29	2.053E-20				CCDC64	CCDC64		
V3SVHSHC_6979394	LOC389831	1.604E-29					LOC389831			
V3SVHSHC_10138649	FDCSP	1.670E-29					FDCSP			
V3SVHSHC_6381236	Ms4a15	2.116E-29					Ms4a15			
V3SVHSHC_9167921	IQGAP2	3.255E-29					IQGAP2			
V3SVHSHC_7326983	PMM1	3.326E-29					PMM1			
V3SVHSHC_9562139	EIF3H	4.219E-29	3.468E-20	9.411E-43	8.537E-23		EIF3H	EIF3H	EIF3H	EIF3H
V3SVHSHC_9081725	ZNF300	6.133E-29					ZNF300			
V3SVHSHC_6237356	VPS11	6.357E-29					VPS11			
V3SVHSHC_10124063	LCN12	6.393E-29					LCN12			
V3SVHSHC_5580194	ETFDH	1.129E-28					ETFDH			
V3SVHSHC_9383543	SGCB	1.577E-28					SGCB			
V3SVHSHC_8800664	ZNF202	1.662E-28					ZNF202			
V3SVHSHC_7877720	ZNF468	2.586E-28					ZNF468			
V3SVHSHC_10702025	MTRNR2L10	7.468E-28					MTRNR2L10			
V3SVHSHC_8433638	FAM24A	8.702E-28					FAM24A			
V3SVHSHC_5984873	RAP1GDS1	9.620E-28					RAP1GDS1			
V3SVHSHC_7504028	C20ORF166	1.581E-27	7.678E-10				C20ORF166	C20ORF1<br>66		
V3SVHSHC_9773405	PAGE2B	1.823E-27					PAGE2B			
V3SVHSHC_7670018	OR9Q2	1.845E-27					OR9Q2			
V3SVHSHC_6376022	TBC1D26	2.663E-27					TBC1D26			
V3SVHSHC_10666781	CALCOCO1	2.717E-27					CALCOCO1			
V3SVHSHC_4728035	NNAT	2.778E-27					NNAT			
V3SVHSHC_10637147	C12orf49	2.989E-27					C12orf49			
V3SVHSHC_6060806	WDR96	3.488E-27					WDR96			
V3SVHSHC_7725260	FGF18	4.012E-27					FGF18			
V3SVHSHC_8109908	CCDC86	5.503E-27					CCDC86			
V3SVHSHC_7974311	ANKRD13D	6.211E-27					ANKRD13D			
V3SVHSHC_6251612	C15orf60	2.750E-26					C15orf60			
V3SVHSHC_6459875	DCAF15	2.859E-26					DCAF15			
V3SVHSHC_10733573	PALLD	3.416E-26					PALLD			
V3SVHSHC_9550391	CORO2B	3.897E-26		7.181E-07			CORO2B		CORO2B	
V3SVHSHC_6818222	PSMG2	4.101E-26					PSMG2			
V3SVHSHC_9555605	GOLGB1	4.554E-26					GOLGB1			
V3SVHSHC_9096740	GOT2	7.409E-26					GOT2			
V3SVHSHC_4645766	SPTSSA	7.864E-26					SPTSSA			
V3SVHSHC_9589496	NTF3	9.968E-26					NTF3			
V3SVHSHC_6508385	RHBDL2	1.136E-25					RHBDL2			
V3SVHSHC_9812609	SUOX	1.147E-25					SUOX			
V3SVHSHC_8691830	FGF19	1.260E-25					FGF19			
V3SVHSHC_7132085	JADE1	1.541E-25					JADE1			
V3SVHSHC_6580589	LIPI	2.149E-25					LIPI			
V3SVHSHC_10254941	CXCR4	2.295E-25			2.875E-06		CXCR4			CXCR4
V3SVHSHC_4859705	SERPINB5	4.941E-25					SERPINB5			
V3SVHSHC_9673448	RTL1	5.068E-25		1.969E-01			RTL1			
V3SVHSHC_7867655	ZBTB46	5.113E-25					ZBTB46			
V3SVHSHC_8283620	KLF9	5.528E-25					KLF9			
V3SVHSHC_10164290	PCCB	6.694E-25					PCCB			
V3SVHSHC_4915673	HIPK2	6.965E-25					HIPK2			
V3SVHSHC_6983420	SRCIN1	7.995E-25					SRCIN1			
V3SVHSHC_5656688	ADAMTS2	8.939E-25					ADAMTS2			
V3SVHSHC_10725125	GABBR1	1.024E-24					GABBR1			
V3SVHSHC_5962004	F3	1.180E-24					F3			
V3SVHSHC_6928376	TNFRSF13C	1.409E-24					TNFRSF13C			

V3SVHSHC_10430831	IL36A	1.598E-24					IL36A			
V3SVHSHC_5799050	IMPAD1	1.890E-24	1.667E-09	2.701E-53	3.227E-16		IMPAD1	IMPAD1	IMPAD1	IMPAD1
V3SVHSHC_9866300	NUSAP1	2.999E-24					NUSAP1			
V3SVHSHC_4971443	SOX8	3.481E-24					SOX8			
V3SVHSHC_8428160	GIP	4.121E-24					GIP			
V3SVHSHC_10098653	SUGCT	5.851E-24					SUGCT			
V3SVHSHC_6910424	CDR2L	6.474E-24					CDR2L			
V3SVHSHC_10101656	CST7	6.812E-24					CST7			
V3SVHSHC_4751696	SDAD1	8.710E-24					SDAD1			
V3SVHSHC_10679057	MAP4K3	1.115E-23					MAP4K3			
V3SVHSHC_7958273	FIS1	1.327E-23					FIS1			
V3SVHSHC_9656783	EXT2	1.429E-23					EXT2			
V3SVHSHC_8003252	SH3BP2	1.438E-23					SH3BP2			
V3SVHSHC_6505877	SOX13	1.496E-23					SOX13			
V3SVHSHC_8235242	SOX8	1.790E-23					SOX8			
V3SVHSHC_7263524	LDLR	1.950E-23					LDLR			
V3SVHSHC_9647378	IDUA	2.053E-23					IDUA			
V3SVHSHC_5493965	DSC1	2.343E-23					DSC1			
V3SVHSHC_6514952	LRRC48	2.393E-23					LRRC48			
V3SVHSHC_9010148	ELAVL1	2.451E-23					ELAVL1			
V3SVHSHC_8292761	TRAF6	2.469E-23					TRAF6			
V3SVHSHC_9855146	EPHA3	3.016E-23					EPHA3			
V3SVHSHC_10788221	C1RL	3.671E-23		5.017E-06			C1RL		C1RL	
V3SVHSHC_5452550	KIAA1279	4.166E-23					KIAA1279			
V3SVHSHC_5306195	KLF17	4.831E-23					KLF17			
V3SVHSHC_10169339	HYPK	5.265E-23					HYPK			
V3SVHSHC_8100272	ACCSL	5.331E-23					ACCSL			
V3SVHSHC_9821651	C3orf36	8.486E-23					C3orf36			
V3SVHSHC_6923195	CX3CR1	9.195E-23					CX3CR1			
V3SVHSHC_10663580	PPM1J	9.288E-23					PPM1J			
V3SVHSHC_6034703	SDF2	9.358E-23					SDF2			
V3SVHSHC_7188548	GDPD1	1.246E-22					GDPD1			
V3SVHSHC_5017841	EGFR	1.322E-22					EGFR			
V3SVHSHC_8152280	SARS2	1.322E-22					SARS2			
V3SVHSHC_5059256	CRAT	1.335E-22					CRAT			
V3SVHSHC_10263389	NECAP2	1.437E-22					NECAP2			
V3SVHSHC_7238840	AK8	2.482E-22					AK8			
V3SVHSHC_9561182	EBPL	2.550E-22			5.303E-03		EBPL			
V3SVHSHC_6356024	IDUA	2.560E-22					IDUA			
V3SVHSHC_9403706	RAB12	2.617E-22					RAB12			
V3SVHSHC_7718726	ANXA2R	3.002E-22					ANXA2R			
V3SVHSHC_5044439	PIP5K1C	3.144E-22					PIP5K1C			
V3SVHSHC_5801525	AMMECR1L	3.321E-22					AMMECR1L			
V3SVHSHC_6717440	FXYD6	3.467E-22					FXYD6			
V3SVHSHC_5611082	UBE2R2	3.589E-22					UBE2R2			
V3SVHSHC_8284808	MBD3	3.711E-22					MBD3			
V3SVHSHC_9026681	OR6F1	3.905E-22					OR6F1			
V3SVHSHC_5219801	CNIH1	4.142E-22					CNIH1			
V3SVHSHC_10619987	C5orf24	6.307E-22					C5orf24			
V3SVHSHC_7573262	ISL1	7.091E-22					ISL1			
V3SVHSHC_5092619	CXorf57	7.110E-22					CXorf57			
V3SVHSHC_6307217	LOC101928764	7.841E-22					LOC10192876 4			
V3SVHSHC_7073708	KCTD14	7.956E-22					KCTD14			
V3SVHSHC_6335399	CBWD7	7.962E-22					CBWD7			

V3SVHSHC_7398791	RNF8	8.106E-22					RNF8			
V3SVHSHC_9645497	Ermn	9.458E-22			4.241E-18		Ermn			Ermn
V3SVHSHC_9713642	SPATS2L	9.608E-22					SPATS2L			
V3SVHSHC_5893199	CYB5D2	9.914E-22					CYB5D2			
V3SVHSHC_6174227	NADK2	1.066E-21					NADK2			
V3SVHSHC_10230125	TRIM45	1.147E-21					TRIM45			
V3SVHSHC_10541282	TPD52L3	1.262E-21					TPD52L3			
V3SVHSHC_5095721	HERPUD1	1.462E-21					HERPUD1			
V3SVHSHC_5179178	WDR55	1.515E-21					WDR55			
V3SVHSHC_6361370	TP73	1.518E-21					TP73			
V3SVHSHC_4754303	ARPC5L	1.664E-21					ARPC5L			
V3SVHSHC_6407669	TMED3	1.787E-21					TMED3			
V3SVHSHC_6044900	PRAME	2.071E-21					PRAME			
V3SVHSHC_7277549	CDC20B	2.138E-21					CDC20B			
V3SVHSHC_5383910	OR2F2	2.163E-21	2.380E-12	2.185E-71	2.320E-26		OR2F2	OR2F2	OR2F2	OR2F2
V3SVHSHC_7417634	SRRD	2.294E-21					SRRD			
V3SVHSHC_7272500	MAP1B	2.512E-21					MAP1B			
V3SVHSHC_8619989	RABL2A	2.661E-21					RABL2A			
V3SVHSHC_8974277	LETMD1	2.901E-21					LETMD1			
V3SVHSHC_4662167	GEMIN8	3.369E-21					GEMIN8			
V3SVHSHC_10063937	FTMT	3.438E-21					FTMT			
V3SVHSHC_9169472	IMPA1	3.492E-21					IMPA1			
V3SVHSHC_7011965	ORC5	3.904E-21					ORC5			
V3SVHSHC_7049057	COL26A1	4.510E-21			8.510E-14		COL26A1			COL26A1
V3SVHSHC_9169802	OR7A5	4.641E-21					OR7A5			
V3SVHSHC_9502574	SLC7A3	6.125E-21					SLC7A3			
V3SVHSHC_6715988	REP15	6.360E-21					REP15			
V3SVHSHC_9706118	OR2W1	9.664E-21					OR2W1			
V3SVHSHC_9042422	ZNF132	1.031E-20			1.082E-01		ZNF132			
V3SVHSHC_7113242	MACF1	1.414E-20					MACF1			
V3SVHSHC_5049356	ARHGEF19	1.488E-20					ARHGEF19			
V3SVHSHC_6654278	HK2	1.692E-20					HK2			
V3SVHSHC_5516471	PARP3	2.073E-20					PARP3			
V3SVHSHC_5168321	VPS39	2.165E-20					VPS39			
V3SVHSHC_10112513	Ubash3b	2.187E-20					Ubash3b			
V3SVHSHC_8927912	ROR2	2.323E-20					ROR2			
V3SVHSHC_7731728	NRIP2	2.526E-20					NRIP2			
V3SVHSHC_5456906	EHBP1L1	3.013E-20					EHBP1L1			
V3SVHSHC_6807761	RXFP1	3.258E-20					RXFP1			
V3SVHSHC_9074894	EVA1B	3.272E-20					EVA1B			
V3SVHSHC_5606363	RNASE1	3.404E-20					RNASE1			
V3SVHSHC_9045524	ZDHHC3	3.427E-20					ZDHHC3			
V3SVHSHC_8182178	TAB2	3.716E-20					TAB2			
V3SVHSHC_8819903	PARS2	3.983E-20					PARS2			
V3SVHSHC_9925238	NKAIN3	4.090E-20					NKAIN3			
V3SVHSHC_4883564	SFRP1	4.432E-20					SFRP1			
V3SVHSHC_7366088	ZNF20	5.196E-20					ZNF20			
V3SVHSHC_5520959	SYPL1	5.285E-20					SYPL1			
V3SVHSHC_7395260	RILPL1	5.608E-20					RILPL1			
V3SVHSHC_6705989	TRIM28	5.655E-20					TRIM28			
V3SVHSHC_9417830	LARP1B	5.835E-20					LARP1B			
V3SVHSHC_8485679	LOC101929070	6.012E-20					LOC101929070			
V3SVHSHC_8054798	IL36G	7.665E-20			3.919E-01		IL36G			
V3SVHSHC_5133275	LOC101059915	7.812E-20					LOC101059915			

V3SVHSHC_9356417	NXPH3	7.854E-20					NXPH3			
V3SVHSHC_6698861	HS2ST1	7.879E-20		9.048E-17			HS2ST1		HS2ST1	
V3SVHSHC_5704340	PRAMEF25	9.774E-20					PRAMEF25			
V3SVHSHC_5496836	TMPRSS12	1.071E-19					TMPRSS12			
V3SVHSHC_9840461	GAGE4	1.102E-19					GAGE4			
V3SVHSHC_5665466	C1orf105	1.107E-19					C1orf105			
V3SVHSHC_6438062	SDC4	1.114E-19					SDC4			
V3SVHSHC_6189176	MTUS1	1.120E-19					MTUS1			
V3SVHSHC_4938872	EFHB	1.125E-19					EFHB			
V3SVHSHC_4927850	MYBL1	1.297E-19					MYBL1			
V3SVHSHC_5972069	NR1H4	1.584E-19					NR1H4			
V3SVHSHC_9049484	KDM6B	1.726E-19					KDM6B			
V3SVHSHC_5626955	THEMIS	2.138E-19					THEMIS			
V3SVHSHC_7846502	FAM177A1	2.464E-19					FAM177A1			
V3SVHSHC_8193431	PDE1A	2.482E-19					PDE1A			
V3SVHSHC_6230426	IL17B	2.572E-19					IL17B			
V3SVHSHC_8904713	ACTN2	3.229E-19					ACTN2			
V3SVHSHC_9546563	SUDS3	3.414E-19	5.247E-13	7.036E-27	8.122E-05		SUDS3	SUDS3	SUDS3	SUDS3
V3SVHSHC_10111061	RASSF4	4.038E-19					RASSF4			
V3SVHSHC_8521352	IFNL4	4.165E-19					IFNL4			
V3SVHSHC_6729815	NUDT12	4.536E-19					NUDT12			
V3SVHSHC_7605338	TMEM150B	4.627E-19					TMEM150B			
V3SVHSHC_4990979	AGPAT9	5.047E-19					AGPAT9			
V3SVHSHC_9260321	C5orf55	5.065E-19					C5orf55			
V3SVHSHC_6794594	TMEM165	5.146E-19					TMEM165			
V3SVHSHC_8453471	SPERT	5.197E-19					SPERT			
V3SVHSHC_8482115	HSF4	5.202E-19					HSF4			
V3SVHSHC_9866003	STX1A	5.439E-19					STX1A			
V3SVHSHC_9077897	GPR113	5.615E-19					GPR113			
V3SVHSHC_7488749	KCNK16	5.622E-19					KCNK16			
V3SVHSHC_9731693	ZNF20	5.639E-19					ZNF20			
V3SVHSHC_8033909	ETV4	6.401E-19					ETV4			
V3SVHSHC_5459348	LOC101930637	6.627E-19					LOC10193063<br>7			
V3SVHSHC_10853990	SLC25A53	6.918E-19					SLC25A53			
V3SVHSHC_6746117	ANKRD66	7.336E-19					ANKRD66			
V3SVHSHC_5123936	GIP	8.101E-19					GIP			
V3SVHSHC_5083115	FHL2	8.317E-19					FHL2			
V3SVHSHC_9932960	SGCE	8.862E-19					SGCE			
V3SVHSHC_8076776	PIWIL4	9.663E-19					PIWIL4			
V3SVHSHC_8989457	STK19	9.670E-19					STK19			
V3SVHSHC_9381464	RABEP2	9.921E-19					RABEP2			
V3SVHSHC_9776342	VPS11	1.003E-18					VPS11			
V3SVHSHC_5551451	OR1G1	1.015E-18					OR1G1			
V3SVHSHC_5337710	ASPDH	1.048E-18					ASPDH			
V3SVHSHC_5169311	ACTN1	1.060E-18		1.149E-27			ACTN1		ACTN1	
V3SVHSHC_5061995	KCNS2	1.109E-18					KCNS2			
V3SVHSHC_6949034	WDR64	1.110E-18					WDR64			
V3SVHSHC_9481883	CRLF3	1.280E-18					CRLF3			
V3SVHSHC_7891217	C1orf194	1.290E-18					C1orf194			
V3SVHSHC_9988235	PLXNA1	1.295E-18					PLXNA1			
V3SVHSHC_9404927	PDHB	1.308E-18					PDHB			
V3SVHSHC_8183201	ADORA2A	1.315E-18					ADORA2A			
V3SVHSHC_9142148	UBE2V2	1.344E-18					UBE2V2			
V3SVHSHC_9732353	ATP7A	1.467E-18					ATP7A			

V3SVHSHC_5567588	FLAD1	1.676E-18		3.044E-35			FLAD1		FLAD1	
V3SVHSHC_9751295	NBPF19	1.830E-18					NBPF19			
V3SVHSHC_10539269	NRTN	1.894E-18					NRTN			
V3SVHSHC_6554915	TFDP3	1.912E-18		2.888E-15			TFDP3		TFDP3	
V3SVHSHC_7977083	GNA12	1.955E-18					GNA12			
V3SVHSHC_6849506	DNAJA3	1.998E-18					DNAJA3			
V3SVHSHC_5423312	CLDN10	2.038E-18					CLDN10			
V3SVHSHC_9123767	HTR3B	2.080E-18					HTR3B			
V3SVHSHC_4714604	ZNF740	2.401E-18					ZNF740			
V3SVHSHC_10832573	PSMG3	2.403E-18					PSMG3			
V3SVHSHC_5964446	PAQR3	2.500E-18					PAQR3			
V3SVHSHC_5857856	UBQLN2	2.861E-18					UBQLN2			
V3SVHSHC_8986949	MSANTD1	2.916E-18					MSANTD1			
V3SVHSHC_8112020	BPIFA2	3.065E-18					BPIFA2			
V3SVHSHC_4913363	ROBO4	3.494E-18					ROBO4			
V3SVHSHC_7137893	SYT12	3.566E-18					SYT12			
V3SVHSHC_8780138	SSBP2	3.583E-18					SSBP2			
V3SVHSHC_9705821	ORM2	3.677E-18					ORM2			
V3SVHSHC_5887226	SEPT1	3.816E-18					SEPT1			
V3SVHSHC_7854125	ZSCAN31	4.009E-18					ZSCAN31			
V3SVHSHC_9892997	SLC1A3	4.148E-18					SLC1A3			
V3SVHSHC_10092350	GDF2	4.289E-18					GDF2			
V3SVHSHC_5577653	SYNPR	4.347E-18					SYNPR			
V3SVHSHC_7370147	THBS2	4.599E-18					THBS2			
V3SVHSHC_9507689	SNX14	4.635E-18					SNX14			
V3SVHSHC_5158388	CD69	4.792E-18					CD69			
V3SVHSHC_5523236	IDH3G	4.815E-18					IDH3G			
V3SVHSHC_6889469	ATP2B2	4.924E-18					ATP2B2			
V3SVHSHC_4653356	CAV1	4.935E-18					CAV1			
V3SVHSHC_8256593	EPS8L3	5.015E-18					EPS8L3			
V3SVHSHC_7956095	KCTD21	5.362E-18					KCTD21			
V3SVHSHC_7963421	PLEKHG7	5.620E-18					PLEKHG7			
V3SVHSHC_7835414	CHMP3	5.680E-18					CHMP3			
V3SVHSHC_9202604	NSG1	5.730E-18					NSG1			
V3SVHSHC_8908607	DERA	6.385E-18					DERA			
V3SVHSHC_7768160	ARMC5	6.452E-18					ARMC5			
V3SVHSHC_7022162	LOC101928380	6.814E-18					LOC10192838<br>0			
V3SVHSHC_6567488	MAS1	7.067E-18					MAS1			
V3SVHSHC_8046053	ATPAF1	7.967E-18					ATPAF1			
V3SVHSHC_4792946	MLLT1	8.615E-18					MLLT1			
V3SVHSHC_7450766	RPS12	8.711E-18					RPS12			
V3SVHSHC_6355496	SLAMF6	9.824E-18					SLAMF6			
V3SVHSHC_4661210	SERPINA6	1.038E-17					SERPINA6			
V3SVHSHC_5785553	KLHL13	1.137E-17					KLHL13			
V3SVHSHC_7400474	LOC101930512	1.138E-17					LOC10193051<br>2			
V3SVHSHC_6148421	PLA2G4F	1.177E-17					PLA2G4F			
V3SVHSHC_7429646	Slc22a20	1.178E-17					Slc22a20			
V3SVHSHC_5187692	COL28A1	1.180E-17					COL28A1			
V3SVHSHC_9512540	HTR3D	1.199E-17					HTR3D			
V3SVHSHC_7957481	OR6K2	1.248E-17					OR6K2			
V3SVHSHC_6895013	OAT	1.279E-17					OAT			
V3SVHSHC_8744630	OR2C1	1.302E-17					OR2C1			
V3SVHSHC_10817492	TRIM54	1.353E-17					TRIM54			
V3SVHSHC_9366119	RPL3L	1.493E-17					RPL3L			

V3SVHSHC_8297843	ADRA2C	1.535E-17					ADRA2C			
V3SVHSHC_9491585	AMELY	1.645E-17					AMELY			
V3SVHSHC_7408625	OR4K13	1.671E-17					OR4K13			
V3SVHSHC_9209303	ATE1	1.672E-17					ATE1			
V3SVHSHC_5054834	HOXD11	1.870E-17					HOXD11			
V3SVHSHC_5545412	INF2	1.972E-17	4.969E-01				INF2			
V3SVHSHC_9610154	AP5S1	2.025E-17					AP5S1			
V3SVHSHC_6069056	KIDINS220	2.084E-17					KIDINS220			
V3SVHSHC_9916064	DBH	2.120E-17					DBH			
V3SVHSHC_9625367	HSPB7	2.182E-17					HSPB7			
V3SVHSHC_9074498	TRPS1	2.287E-17					TRPS1			
V3SVHSHC_6844688	PSMD9	2.310E-17					PSMD9			
V3SVHSHC_7085390	ACTR1B	2.406E-17					ACTR1B			
V3SVHSHC_7746974	LOC101930546	2.435E-17					LOC101930546			
V3SVHSHC_5418329	TIMM9	2.444E-17					TIMM9			
V3SVHSHC_5689358	TBCE	2.493E-17					TBCE			
V3SVHSHC_9786011	CLIP1	2.680E-17			1.929E-09		CLIP1			CLIP1
V3SVHSHC_8511617	NKAIN2	2.699E-17					NKAIN2			
V3SVHSHC_6307085	DQX1	2.807E-17					DQX1			
V3SVHSHC_9788321	ZNF215	3.204E-17					ZNF215			
V3SVHSHC_5293754	KRTAP13-3	3.211E-17					KRTAP13-3			
V3SVHSHC_7881713	HSPA1A	3.330E-17					HSPA1A			
V3SVHSHC_10729085	EIF1	3.355E-17					EIF1			
V3SVHSHC_9112217	PRPF4	3.562E-17					PRPF4			
V3SVHSHC_5113541	GPR31	3.563E-17	2.703E-02		3.467E-01		GPR31			
V3SVHSHC_5109416	FAM72A	3.609E-17					FAM72A			
V3SVHSHC_5936198	PPM1G	3.627E-17					PPM1G			
V3SVHSHC_10575140	CASZ1	3.974E-17					CASZ1			
V3SVHSHC_9638600	C6orf7	4.185E-17					C6orf7			
V3SVHSHC_6925967	VSTM2L	4.325E-17					VSTM2L			
V3SVHSHC_9956159	ZNF747	4.417E-17					ZNF747			
V3SVHSHC_6330185	C10orf113	4.471E-17					C10orf113			
V3SVHSHC_10257416	Camsap3	4.582E-17					Camsap3			
V3SVHSHC_6314873	SHCBP1	4.707E-17					SHCBP1			
V3SVHSHC_7933886	TMEM98	4.750E-17					TMEM98			
V3SVHSHC_5955932	CPE	4.783E-17					CPE			
V3SVHSHC_7550228	HRH1	5.156E-17					HRH1			
V3SVHSHC_6176108	PATE3	5.184E-17					PATE3			
V3SVHSHC_6272402	RAB15	5.359E-17					RAB15			
V3SVHSHC_10047074	LIMA1	5.428E-17					LIMA1			
V3SVHSHC_4801922	FAM8A1	5.951E-17					FAM8A1			
V3SVHSHC_8308931	CLCN2	6.033E-17					CLCN2			
V3SVHSHC_5181158	RASA2	6.196E-17					RASA2			
V3SVHSHC_5574848	CASP7	6.225E-17					CASP7			
V3SVHSHC_6844523	TRADD	6.371E-17					TRADD			
V3SVHSHC_6076877	PNKP	6.946E-17					PNKP			
V3SVHSHC_9045689	MAPK3	7.915E-17					MAPK3			
V3SVHSHC_9665891	UQCRC1	9.004E-17					UQCRC1			
V3SVHSHC_6432650	TMOD2	9.473E-17					TMOD2			
V3SVHSHC_5312960	NAA11	1.008E-16					NAA11			
V3SVHSHC_6791723	ARL11	1.090E-16					ARL11			
V3SVHSHC_6390113	TRPM2	1.112E-16					TRPM2			
V3SVHSHC_5429087	MOGAT2	1.189E-16					MOGAT2			
V3SVHSHC_10834058	PRSS57	1.262E-16					PRSS57			

V3SVHSHC_7504061	AQP8	1.370E-16					AQP8			
V3SVHSHC_9724895	CAMK2N1	1.407E-16					CAMK2N1			
V3SVHSHC_6063314	ANKFN1	1.467E-16					ANKFN1			
V3SVHSHC_6278408	KIAA1033	1.507E-16					KIAA1033			
V3SVHSHC_5148422	C17orf112	1.521E-16					C17orf112			
V3SVHSHC_7688102	SEC13	1.591E-16					SEC13			
V3SVHSHC_5297747	METRNL	1.594E-16					METRNL			
V3SVHSHC_10432184	GLRA3	1.600E-16					GLRA3			
V3SVHSHC_5279168	CNNM1	1.681E-16					CNNM1			
V3SVHSHC_8721596	H2AFJ	1.754E-16					H2AFJ			
V3SVHSHC_9416972	ATF6	1.757E-16					ATF6			
V3SVHSHC_7365164	SYCE1	1.767E-16					SYCE1			
V3SVHSHC_9828185	WNT10B	1.835E-16					WNT10B			
V3SVHSHC_9455054	TP53TG3D	1.972E-16					TP53TG3D			
V3SVHSHC_7437467	SLC26A5	2.010E-16					SLC26A5			
V3SVHSHC_10008926	LOC101930318	2.062E-16					LOC101930318			
V3SVHSHC_9647708	SLC34A2	2.108E-16		3.898E-03			SLC34A2			
V3SVHSHC_9377702	ZNF569	2.193E-16					ZNF569			
V3SVHSHC_4644875	CCL5	2.238E-16					CCL5			
V3SVHSHC_4980584	NR2C2	2.411E-16					NR2C2			
V3SVHSHC_6420143	KLHL6	2.559E-16	1.479E-14	6.762E-15	7.291E-24		KLHL6	KLHL6	KLHL6	KLHL6
V3SVHSHC_4977515	VAMP8	2.787E-16	1.910E-06				VAMP8	VAMP8		
V3SVHSHC_6791624	PATE1	2.837E-16					PATE1			
V3SVHSHC_9969392	OR7A5	2.881E-16					OR7A5			
V3SVHSHC_8324540	MAPK15	2.906E-16					MAPK15			
V3SVHSHC_9512903	LAGE3	3.009E-16					LAGE3			
V3SVHSHC_10001963	MSH6	3.161E-16					MSH6			
V3SVHSHC_10348826	PPP2R2B	3.278E-16					PPP2R2B			
V3SVHSHC_4976723	IQCC	3.285E-16					IQCC			
V3SVHSHC_6296855	SNAP23	3.317E-16					SNAP23			
V3SVHSHC_9794426	HLA-DRA	3.540E-16					HLA-DRA			
V3SVHSHC_5397407	NRG3	3.605E-16					NRG3			
V3SVHSHC_7857194	TRIM38	3.606E-16					TRIM38			
V3SVHSHC_10354073	LIN28B	3.650E-16					LIN28B			
V3SVHSHC_8590850	CCL20	3.748E-16					CCL20			
V3SVHSHC_4829609	TPRKB	3.956E-16					TPRKB			
V3SVHSHC_9097565	NDFIP2	4.307E-16					NDFIP2			
V3SVHSHC_8843366	RASSF1	4.612E-16					RASSF1			
V3SVHSHC_5142284	TMEM173	4.622E-16					TMEM173			
V3SVHSHC_6878282	ACOT1	4.916E-16					ACOT1			
V3SVHSHC_9581807	SCAF11	5.488E-16					SCAF11			
V3SVHSHC_9920189	CRADD	5.515E-16					CRADD			
V3SVHSHC_9571808	LOC100144595	5.646E-16					LOC100144595			
V3SVHSHC_9416543	SLC7A11	5.655E-16					SLC7A11			
V3SVHSHC_8873264	ARHGDIG	5.735E-16					ARHGDIG			
V3SVHSHC_6300089	SPIN1	6.166E-16					SPIN1			
V3SVHSHC_9199040	PNLIPRP2	6.349E-16					PNLIPRP2			
V3SVHSHC_7951145	MFAP2	6.425E-16					MFAP2			
V3SVHSHC_9920321	OR51B5	6.726E-16					OR51B5			
V3SVHSHC_8548973	GARS	6.787E-16					GARS			
V3SVHSHC_6577982	EEF1G	6.799E-16					EEF1G			
V3SVHSHC_10411625	THAP3	6.880E-16					THAP3			
V3SVHSHC_5879669	GFRAL	6.917E-16					GFRAL			
V3SVHSHC_9656057	Izumo2	7.186E-16					Izumo2			

V3SVHSHC_6961772	B3GNT6	7.188E-16					B3GNT6			
V3SVHSHC_8447927	TLX2	7.848E-16					TLX2			
V3SVHSHC_6386186	TXNDC15	7.930E-16					TXNDC15			
V3SVHSHC_5703284	SLC38A10	8.059E-16					SLC38A10			
V3SVHSHC_7765718	NKX2-2	8.339E-16					NKX2-2			
V3SVHSHC_9646586	LDLR	8.424E-16					LDLR			
V3SVHSHC_9665627	ARL4D	8.539E-16					ARL4D			
V3SVHSHC_9862571	TSPAN5	8.731E-16					TSPAN5			
V3SVHSHC_8975630	FADS2	9.013E-16					FADS2			
V3SVHSHC_9734465	C8orf82	9.218E-16					C8orf82			
V3SVHSHC_8633321	LOC101929072	1.002E-15					LOC10192907<br>2			
V3SVHSHC_7233692	IPO4	1.028E-15					IPO4			
V3SVHSHC_8362391	C12ORF5	1.040E-15					C12ORF5			
V3SVHSHC_7172972	GYG1	1.048E-15					GYG1			
V3SVHSHC_8056217	ZMIZ2	1.106E-15					ZMIZ2			
V3SVHSHC_8108357	RAG1	1.113E-15					RAG1			
V3SVHSHC_8917286	LIN7A	1.162E-15					LIN7A			
V3SVHSHC_9704930	TLX3	1.227E-15					TLX3			
V3SVHSHC_8746082	SIK2	1.284E-15					SIK2			
V3SVHSHC_10419875	PIK3C2B	1.305E-15					PIK3C2B			
V3SVHSHC_10337837	CPLX3	1.361E-15					CPLX3			
V3SVHSHC_6885377	LOC100996758	1.372E-15					LOC10099675<br>8			
V3SVHSHC_8539469	FAF2	1.476E-15					FAF2			
V3SVHSHC_7887818	SLC8A1	1.488E-15					SLC8A1			
V3SVHSHC_7448126	DEFB118	1.533E-15					DEFB118			
V3SVHSHC_10577549	PLEKHO1	1.569E-15		2.426E-02			PLEKHO1			
V3SVHSHC_5101067	SLC6A1	1.602E-15					SLC6A1			
V3SVHSHC_7673516	EHMT2	1.613E-15					EHMT2			
V3SVHSHC_9445880	GNAT1	1.692E-15					GNAT1			
V3SVHSHC_8429777	KCNAB3	1.728E-15					KCNAB3			
V3SVHSHC_8390243	SLC13A5	1.729E-15					SLC13A5			
V3SVHSHC_6493271	OR7G1	1.768E-15					OR7G1			
V3SVHSHC_5174723	STXBP6	1.792E-15					STXBP6			
V3SVHSHC_7417997	POP1	1.811E-15					POP1			
V3SVHSHC_4749287	ELAVL3	1.827E-15					ELAVL3			
V3SVHSHC_9094133	FOSL1	1.859E-15					FOSL1			
V3SVHSHC_8893493	RYR1	1.898E-15					RYR1			
V3SVHSHC_9343613	SALL1	1.898E-15					SALL1			
V3SVHSHC_8227223	CRELD2	1.935E-15					CRELD2			
V3SVHSHC_9627809	GAGE8	1.960E-15					GAGE8			
V3SVHSHC_9277877	PDCD4	1.994E-15		4.809E-18			PDCD4		PDCD4	
V3SVHSHC_6846734	DEFB128	2.132E-15					DEFB128			
V3SVHSHC_4814924	LOC100505549	2.225E-15					LOC10050554<br>9			
V3SVHSHC_5697773	ECI1	2.250E-15					ECI1			
V3SVHSHC_6559172	KIFC1	2.280E-15					KIFC1			
V3SVHSHC_10669223	STAT5B	2.410E-15					STAT5B			
V3SVHSHC_6640253	OTOF	2.555E-15					OTOF			
V3SVHSHC_6078857	GPR83	2.627E-15					GPR83			
V3SVHSHC_5699423	MTR	2.663E-15					MTR			
V3SVHSHC_9017540	PPIL4	2.690E-15					PPIL4			
V3SVHSHC_10727864	EXD1	2.862E-15					EXD1			
V3SVHSHC_9667277	SPINK7	2.878E-15					SPINK7			
V3SVHSHC_7435157	CCDC87	2.895E-15					CCDC87			
V3SVHSHC_7581149	PPP2R3C	3.035E-15					PPP2R3C			

V3SVHSHC_6949496	CCK	3.161E-15					CCK			
V3SVHSHC_10620284	LRRC58	3.260E-15					LRRC58			
V3SVHSHC_9182045	Wdr38	3.355E-15					Wdr38			
V3SVHSHC_7027244	RAB26	3.377E-15					RAB26			
V3SVHSHC_6329129	SNAI1	3.484E-15					SNAI1			
V3SVHSHC_8397899	PFDN6	3.503E-15					PFDN6			
V3SVHSHC_8513663	C2orf54	4.024E-15					C2orf54			
V3SVHSHC_6286262	AGBL2	4.313E-15					AGBL2			
V3SVHSHC_8532506	IL2RB	4.380E-15					IL2RB			
V3SVHSHC_5468753	VPS4B	4.589E-15			3.492E-01		VPS4B			
V3SVHSHC_8937020	MYL6	4.758E-15					MYL6			
V3SVHSHC_5713349	OR4C6	4.799E-15					OR4C6			
V3SVHSHC_9325067	MYL7	4.907E-15	3.627E-05				MYL7	MYL7		
V3SVHSHC_7270355	GABARAPL1	4.920E-15					GABARAPL1			
V3SVHSHC_10558541	RAD23A	5.041E-15					RAD23A			
V3SVHSHC_10227089	P4HTM	5.089E-15					P4HTM			
V3SVHSHC_7307843	DMTN	5.091E-15					DMTN			
V3SVHSHC_6361832	JAG2	5.408E-15					JAG2			
V3SVHSHC_4673288	CAPN9	5.642E-15					CAPN9			
V3SVHSHC_7567190	APOA1BP	6.071E-15					APOA1BP			
V3SVHSHC_9527555	PRAP1	6.343E-15					PRAP1			
V3SVHSHC_7933820	NDUFV3	6.429E-15					NDUFV3			
V3SVHSHC_8270057	TAS2R13	6.932E-15					TAS2R13			
V3SVHSHC_9863231	RAD9A	7.023E-15					RAD9A			
V3SVHSHC_5066351	PLEKHA3	7.030E-15					PLEKHA3			
V3SVHSHC_9312659	PARK2	7.137E-15					PARK2			
V3SVHSHC_7576727	FCF1	7.316E-15					FCF1			
V3SVHSHC_7460171	Ankle1	7.392E-15					Ankle1			
V3SVHSHC_9053774	COL28A1	7.438E-15					COL28A1			
V3SVHSHC_10463831	EFCAB4B	7.592E-15					EFCAB4B			
V3SVHSHC_9527984	FREM3	7.963E-15					FREM3			
V3SVHSHC_7039454	PHLPP1	8.058E-15					PHLPP1			
V3SVHSHC_7034900	NRF1	8.416E-15					NRF1			
V3SVHSHC_9823004	OR6C75	8.487E-15					OR6C75			
V3SVHSHC_8147099	NAT16	8.512E-15					NAT16			
V3SVHSHC_5733017	C12orf45	8.879E-15					C12orf45			
V3SVHSHC_10743605	DSCC1	9.044E-15					DSCC1			
V3SVHSHC_5724272	ANP32C	9.089E-15					ANP32C			
V3SVHSHC_6028367	MECOM	9.452E-15					MECOM			
V3SVHSHC_5823569	PLA2G1B	9.692E-15					PLA2G1B			
V3SVHSHC_6159872	ANKRD7	9.760E-15					ANKRD7			
V3SVHSHC_5264516	OR52B4	1.084E-14					OR52B4			
V3SVHSHC_9952958	OR1Q1	1.106E-14					OR1Q1			
V3SVHSHC_7040774	OR5M1	1.133E-14					OR5M1			
V3SVHSHC_9176732	LILRA2	1.138E-14					LILRA2			
V3SVHSHC_7509671	SRP72	1.139E-14					SRP72			
V3SVHSHC_8751164	OR5B12	1.144E-14					OR5B12			
V3SVHSHC_10446902	NEK4	1.145E-14					NEK4			
V3SVHSHC_8427797	TRAF3IP2	1.166E-14					TRAF3IP2			
V3SVHSHC_5824724	USF2	1.184E-14					USF2			
V3SVHSHC_5723084	GTF2H3	1.184E-14					GTF2H3			
V3SVHSHC_7750472	Pabpc1l2b	1.186E-14					Pabpc1l2b			
V3SVHSHC_5163008	TMEM241	1.198E-14					TMEM241			
V3SVHSHC_8654243	REXO1L1P	1.218E-14					REXO1L1P			
V3SVHSHC_9805382	NCALD	1.242E-14					NCALD			

V3SVHSHC_8744069	GCM1	1.270E-14					GCM1			
V3SVHSHC_9272201	AQP4	1.273E-14		1.452E-08			AQP4		AQP4	
V3SVHSHC_10109675	NPTX1	1.273E-14					NPTX1			
V3SVHSHC_6925307	SPIB	1.290E-14					SPIB			
V3SVHSHC_9086246	ACTRT3	1.308E-14					ACTRT3			
V3SVHSHC_7632233	CST6	1.315E-14					CST6			
V3SVHSHC_6619925	ARF3	1.320E-14					ARF3			
V3SVHSHC_8927450	CD302	1.340E-14					CD302			
V3SVHSHC_9659423	EED	1.355E-14					EED			
V3SVHSHC_5111066	GPR171	1.386E-14					GPR171			
V3SVHSHC_9389054	SND1	1.406E-14					SND1			
V3SVHSHC_4978076	GABRG2	1.734E-14					GABRG2			
V3SVHSHC_9864452	C1orf234	1.742E-14					C1orf234			
V3SVHSHC_10516631	PDZRN3	1.767E-14					PDZRN3			
V3SVHSHC_5116775	RGL2	1.773E-14					RGL2			
V3SVHSHC_10810793	EEF1E1	1.907E-14					EEF1E1			
V3SVHSHC_4793276	FAM65A	1.929E-14					FAM65A			
V3SVHSHC_8260355	PDSS2	1.945E-14					PDSS2			
V3SVHSHC_9650381	LRRC39	1.951E-14		3.904E-12			LRRC39		LRRC39	
V3SVHSHC_6067868	CEP250	1.990E-14					CEP250			
V3SVHSHC_4791395	PEG10	2.018E-14					PEG10			
V3SVHSHC_5904848	RHOV	2.043E-14					RHOV			
V3SVHSHC_7410836	GPR126	2.218E-14	8.403E-17				GPR126	GPR126		
V3SVHSHC_7169771	SLC30A9	2.245E-14					SLC30A9			
V3SVHSHC_10172111	REM2	2.256E-14					REM2			
V3SVHSHC_5720015	SP2	2.313E-14					SP2			
V3SVHSHC_5855909	DGUOK	2.496E-14					DGUOK			
V3SVHSHC_10422944	LRFN1	2.517E-14					LRFN1			
V3SVHSHC_7294709	FOXN1	2.588E-14					FOXN1			
V3SVHSHC_9430106	TMEM63C	2.669E-14					TMEM63C			
V3SVHSHC_4794134	RMND5A	2.743E-14					RMND5A			
V3SVHSHC_8270288	C9orf163	2.752E-14					C9orf163			
V3SVHSHC_5674607	POLE4	2.836E-14					POLE4			
V3SVHSHC_5228612	SYTL2	2.860E-14					SYTL2			
V3SVHSHC_9221282	AGBL1	2.984E-14					AGBL1			
V3SVHSHC_4887260	Bend3	3.059E-14					Bend3			
V3SVHSHC_8759678	RXRA	3.081E-14					RXRA			
V3SVHSHC_10503794	ATE1	3.131E-14					ATE1			
V3SVHSHC_7678169	TFE3	3.219E-14					TFE3			
V3SVHSHC_9312263	GS1-259H13.2	3.297E-14					GS1-259H13.2			
V3SVHSHC_7994705	ALX3	3.345E-14					ALX3			
V3SVHSHC_6706451	MX1	3.345E-14					MX1			
V3SVHSHC_6308108	ALDH6A1	3.431E-14					ALDH6A1			
V3SVHSHC_9751394	ESM1	3.462E-14					ESM1			
V3SVHSHC_6218546	LCE2B	3.539E-14					LCE2B			
V3SVHSHC_5623952	CA1	3.544E-14					CA1			
V3SVHSHC_5115455	PDZRN3	3.912E-14					PDZRN3			
V3SVHSHC_9106178	Pabpc1l	4.259E-14					Pabpc1l			
V3SVHSHC_9309392	VPS72	4.419E-14					VPS72			
V3SVHSHC_7787366	FAM19A3	4.458E-14					FAM19A3			
V3SVHSHC_9419084	OR6C1	4.814E-14					OR6C1			
V3SVHSHC_6742025	NELFE	4.927E-14					NELFE			
V3SVHSHC_8941211	AKR1C1	5.089E-14					AKR1C1			
V3SVHSHC_9226364	ARL8B	5.119E-14					ARL8B			
V3SVHSHC_8575241	CTAG1B	5.344E-14					CTAG1B			

V3SVHSHC_9456374	TRIM32	5.351E-14	9.425E-17				TRIM32	TRIM32		
V3SVHSHC_6824492	CTNNA2	5.378E-14					CTNNA2			
V3SVHSHC_9911807	RAPGEF5	6.058E-14					RAPGEF5			
V3SVHSHC_4921250	CHD6	6.099E-14					CHD6			
V3SVHSHC_10814786	CCDC79	6.183E-14					CCDC79			
V3SVHSHC_9819143	IRAK1	6.370E-14					IRAK1			
V3SVHSHC_9786341	SHD	6.553E-14					SHD			
V3SVHSHC_5688401	RFESD	6.614E-14					RFESD			
V3SVHSHC_8192738	PAM	6.784E-14					PAM			
V3SVHSHC_7157528	MRPS26	7.024E-14					MRPS26			
V3SVHSHC_4776380	TOR3A	7.270E-14					TOR3A			
V3SVHSHC_6195380	CSNK1G2	7.482E-14					CSNK1G2			
V3SVHSHC_7301540	GJA4	7.503E-14					GJA4			
V3SVHSHC_5723810	DRD5	7.521E-14					DRD5			
V3SVHSHC_5517791	HSF1	7.820E-14					HSF1			
V3SVHSHC_9656618	CRCP	7.864E-14					CRCP			
V3SVHSHC_6615206	NPY2R	7.866E-14					NPY2R			
V3SVHSHC_9138056	ADCK1	7.919E-14					ADCK1			
V3SVHSHC_7204157	UBE3C	8.034E-14					UBE3C			
V3SVHSHC_7685627	RCN2	8.552E-14		2.846E-50	1.571E-10		RCN2		RCN2	RCN2
V3SVHSHC_5049950	PRTN3	8.615E-14					PRTN3			
V3SVHSHC_5834954	AIFM2	9.151E-14					AIFM2			
V3SVHSHC_5412257	DSCAML1	9.335E-14					DSCAML1			
V3SVHSHC_8814425	ACSM2A	9.807E-14					ACSM2A			
V3SVHSHC_9492410	NOB1	9.967E-14					NOB1			
V3SVHSHC_7038266	SBF2	1.026E-13					SBF2			
V3SVHSHC_9101987	ACVR2B	1.069E-13					ACVR2B			
V3SVHSHC_5425688	C6ORF15	1.072E-13					C6ORF15			
V3SVHSHC_9863726	GNS	1.073E-13					GNS			
V3SVHSHC_5843897	DARS	1.076E-13					DARS			
V3SVHSHC_6897752	OR10V1	1.090E-13					OR10V1			
V3SVHSHC_6280322	USP1	1.133E-13					USP1			
V3SVHSHC_8481323	ATPAF1	1.135E-13					ATPAF1			
V3SVHSHC_5377013	F3	1.177E-13					F3			
V3SVHSHC_5128193	C19ORF12	1.191E-13					C19ORF12			
V3SVHSHC_7149740	Cul9	1.202E-13					Cul9			
V3SVHSHC_9328928	NVL	1.230E-13					NVL			
V3SVHSHC_10779575	PKLR	1.261E-13					PKLR			
V3SVHSHC_8689025	TMEM191B	1.266E-13					TMEM191B			
V3SVHSHC_10695128	NCEH1	1.312E-13					NCEH1			
V3SVHSHC_6225806	UBQLNL	1.339E-13					UBQLNL			
V3SVHSHC_6244451	LOC101927562	1.399E-13					LOC101927562			
V3SVHSHC_7454693	SCN1B	1.420E-13					SCN1B			
V3SVHSHC_6567719	MYH10	1.469E-13					MYH10			
V3SVHSHC_5841554	LINC00684	1.475E-13					LINC00684			
V3SVHSHC_9000446	RICTOR	1.510E-13					RICTOR			
V3SVHSHC_10637345	INSR	1.555E-13					INSR			
V3SVHSHC_10330841	CDR2	1.667E-13	9.296E-12				CDR2	CDR2		
V3SVHSHC_4822151	APOC4	1.697E-13					APOC4			
V3SVHSHC_8846303	MAL2	1.699E-13					MAL2			
V3SVHSHC_4801031	CAPN1	1.751E-13					CAPN1			
V3SVHSHC_10230917	CHML	1.761E-13					CHML			
V3SVHSHC_8881844	CYB561D1	1.776E-13					CYB561D1			
V3SVHSHC_7417436	FNDC9	1.813E-13					FNDC9			

V3SVHSHC_9233657	OR4C16	1.830E-13					OR4C16			
V3SVHSHC_5732126	NR5A1	1.881E-13					NR5A1			
V3SVHSHC_8851979	WFIKKN2	1.898E-13					WFIKKN2			
V3SVHSHC_5765720	GSS	1.972E-13					GSS			
V3SVHSHC_6134891	ITGAD	2.017E-13					ITGAD			
V3SVHSHC_7586561	SLC22A2	2.038E-13					SLC22A2			
V3SVHSHC_9484457	UBE2N	2.113E-13					UBE2N			
V3SVHSHC_8451062	SPDL1	2.131E-13					SPDL1			
V3SVHSHC_10038560	SPATA22	2.133E-13					SPATA22			
V3SVHSHC_8167031	CFDP1	2.159E-13					CFDP1			
V3SVHSHC_6824294	NDUFS3	2.162E-13					NDUFS3			
V3SVHSHC_10734332	PDHB	2.174E-13					PDHB			
V3SVHSHC_8538380	LGSN	2.184E-13					LGSN			
V3SVHSHC_10850294	TSPAN2	2.235E-13		1.184E-29			TSPAN2		TSPAN2	
V3SVHSHC_6178187	SCAF11	2.252E-13					SCAF11			
V3SVHSHC_6470666	NAGS	2.333E-13					NAGS			
V3SVHSHC_5993123	DENND6B	2.391E-13					DENND6B			
V3SVHSHC_8120897	DLX3	2.432E-13					DLX3			
V3SVHSHC_9420998	PPP1R7	2.761E-13					PPP1R7			
V3SVHSHC_4745294	SMIM15	2.771E-13					SMIM15			
V3SVHSHC_5457995	FKBP14	2.813E-13					FKBP14			
V3SVHSHC_7952234	OR10H1	2.837E-13					OR10H1			
V3SVHSHC_6026882	SLC37A2	3.014E-13		3.565E-11			SLC37A2		SLC37A2	
V3SVHSHC_8835116	NDUFA4L2	3.111E-13	1.679E-09				NDUFA4L2	NDUFA4L2		
V3SVHSHC_9556727	FOXR2	3.149E-13					FOXR2			
V3SVHSHC_6166967	SLC7A14	3.236E-13					SLC7A14			
V3SVHSHC_8423870	IVL	3.364E-13					IVL			
V3SVHSHC_9391265	LOC100996707	3.453E-13					LOC100996707			
V3SVHSHC_5991044	HMGN2	3.599E-13					HMGN2			
V3SVHSHC_5504987	CCL24	3.727E-13					CCL24			
V3SVHSHC_7197161	PTPN3	3.738E-13					PTPN3			
V3SVHSHC_10590716	HIST1H1C	3.824E-13					HIST1H1C			
V3SVHSHC_5465915	NDST2	4.043E-13					NDST2			
V3SVHSHC_10804226	AMN1	4.547E-13					AMN1			
V3SVHSHC_4783508	LYZ	4.633E-13					LYZ			
V3SVHSHC_7780007	BMP6	4.832E-13					BMP6			
V3SVHSHC_4929632	EPHX3	4.880E-13					EPHX3			
V3SVHSHC_10512242	PMP22	4.924E-13					PMP22			
V3SVHSHC_6742784	AXDND1	5.041E-13					AXDND1			
V3SVHSHC_7634015	ABCG2	5.112E-13					ABCG2			
V3SVHSHC_10587383	FOXO3	5.172E-13					FOXO3			
V3SVHSHC_7832246	KCNAB3	5.174E-13					KCNAB3			
V3SVHSHC_7228280	GRIA4	5.219E-13		2.529E-29			GRIA4		GRIA4	
V3SVHSHC_7904087	DEFB104A	5.233E-13					DEFB104A			
V3SVHSHC_8362853	MCOLN2	5.249E-13					MCOLN2			
V3SVHSHC_8267384	DDX47	5.252E-13					DDX47			
V3SVHSHC_8972264	VPS41	5.281E-13					VPS41			
V3SVHSHC_7014902	ARHGAP15	5.292E-13					ARHGAP15			
V3SVHSHC_7075061	ING1	5.482E-13					ING1			
V3SVHSHC_6496571	TOMM6	5.754E-13					TOMM6			
V3SVHSHC_9270584	OMA1	5.791E-13					OMA1			
V3SVHSHC_9081263	CCDC92	5.815E-13					CCDC92			
V3SVHSHC_9011864	CCNG2	5.949E-13					CCNG2			
V3SVHSHC_9101591	DIAPH3	6.007E-13					DIAPH3			

V3SVHSHC_9452711	CHRFAM7A	6.062E-13					CHRFAM7A			
V3SVHSHC_9482246	KIR3DL1	6.144E-13					KIR3DL1			
V3SVHSHC_9605072	B4GALT1	6.326E-13					B4GALT1			
V3SVHSHC_6965138	ZNF555	6.390E-13		1.043E-10			ZNF555		ZNF555	
V3SVHSHC_5820335	AP2B1	6.589E-13					AP2B1			
V3SVHSHC_6143075	FITM1	6.598E-13					FITM1			
V3SVHSHC_10722848	SPATA33	6.702E-13					SPATA33			
V3SVHSHC_9620285	BAZ1B	6.710E-13					BAZ1B			
V3SVHSHC_7420175	PTPN13	6.717E-13					PTPN13			
V3SVHSHC_8606789	NPAS1	6.735E-13					NPAS1			
V3SVHSHC_6895046	MCM5	6.828E-13			2.141E-04		MCM5			MCM5
V3SVHSHC_5959463	GRIN3A	7.045E-13					GRIN3A			
V3SVHSHC_7471688	ARF5	7.384E-13					ARF5			
V3SVHSHC_9932894	FAM69C	7.464E-13					FAM69C			
V3SVHSHC_7823006	Tti2	7.476E-13	1.561E-12				Tti2	Tti2		
V3SVHSHC_8643221	OSCP1	7.802E-13					OSCP1			
V3SVHSHC_7551119	HTR1D	7.842E-13					HTR1D			
V3SVHSHC_10263884	OR8G2	7.884E-13					OR8G2			
V3SVHSHC_9701267	IL34	8.127E-13					IL34			
V3SVHSHC_6090077	GABPB1	8.144E-13					GABPB1			
V3SVHSHC_5154164	PDE10A	8.246E-13					PDE10A			
V3SVHSHC_7852772	MCMBP	8.570E-13					MCMBP			
V3SVHSHC_4650188	ATP1B4	9.191E-13					ATP1B4			
V3SVHSHC_9054929	ASTN2	9.286E-13					ASTN2			
V3SVHSHC_6163964	CYP2A7	9.608E-13					CYP2A7			
V3SVHSHC_8570093	MYLPF	9.667E-13					MYLPF			
V3SVHSHC_6938474	PITPNB	9.769E-13					PITPNB			
V3SVHSHC_6331307	HCN1	1.004E-12					HCN1			
V3SVHSHC_7594118	CCNB2	1.009E-12					CCNB2			
V3SVHSHC_10688528	LAMC3	1.012E-12					LAMC3			
V3SVHSHC_4940654	ZNF517	1.026E-12					ZNF517			
V3SVHSHC_10526366	AGMAT	1.040E-12					AGMAT			
V3SVHSHC_7905935	ZNF585B	1.054E-12					ZNF585B			
V3SVHSHC_7015793	RCN2	1.091E-12					RCN2			
V3SVHSHC_7361435	PRDM15	1.102E-12					PRDM15			
V3SVHSHC_5511323	ZBTB20	1.110E-12		5.343E-23			ZBTB20		ZBTB20	
V3SVHSHC_10004405	ESYT3	1.222E-12			3.485E-07		ESYT3			ESYT3
V3SVHSHC_8726084	ZNF607	1.301E-12					ZNF607			
V3SVHSHC_5808323	OR9G1	1.326E-12					OR9G1			
V3SVHSHC_7337378	CT45A2	1.338E-12					CT45A2			
V3SVHSHC_9128024	AIG1	1.380E-12					AIG1			
V3SVHSHC_6743081	FNIP2	1.407E-12					FNIP2			
V3SVHSHC_10390967	FCAMR	1.420E-12					FCAMR			
V3SVHSHC_7054337	RFPL2	1.454E-12					RFPL2			
V3SVHSHC_8138981	NOXA1	1.463E-12					NOXA1			
V3SVHSHC_5415656	NCCRP1	1.473E-12					NCCRP1			
V3SVHSHC_9105353	DYNLRB2	1.528E-12					DYNLRB2			
V3SVHSHC_10160858	PVRL2	1.558E-12					PVRL2			
V3SVHSHC_7796210	PDE1A	1.560E-12					PDE1A			
V3SVHSHC_6263129	CYB5R4	1.622E-12					CYB5R4			
V3SVHSHC_9792974	TAS2R60	1.625E-12					TAS2R60			
V3SVHSHC_9792314	KRTAP5-6	1.670E-12					KRTAP5-6			
V3SVHSHC_6788489	DEFB4B	1.692E-12					DEFB4B			
V3SVHSHC_6382589	ACBD4	1.721E-12					ACBD4			
V3SVHSHC_5439119	WBP2	1.746E-12					WBP2			

V3SVHSHC_9381398	PCDH19	1.746E-12	1.043E-04	5.146E-12	3.454E-02		PCDH19	PCDH19	PCDH19	
V3SVHSHC_6420242	KIAA0408	1.751E-12					KIAA0408			
V3SVHSHC_8480267	SLC37A3	1.764E-12					SLC37A3			
V3SVHSHC_9885902	PLA2G1B	1.784E-12					PLA2G1B			
V3SVHSHC_8723312	TAC1	1.802E-12					TAC1			
V3SVHSHC_8853167	SLC9B1	1.849E-12					SLC9B1			
V3SVHSHC_5934614	STK17B	1.864E-12	1.729E-11	3.915E-36	1.344E-22		STK17B	STK17B	STK17B	STK17B
V3SVHSHC_6744500	DIP2B	1.897E-12					DIP2B			
V3SVHSHC_4735658	CDK11A	1.903E-12					CDK11A			
V3SVHSHC_4829675	ZNF230	1.962E-12					ZNF230			
V3SVHSHC_8529107	DCDC5	2.045E-12					DCDC5			
V3SVHSHC_6834788	DCPS	2.059E-12					DCPS			
V3SVHSHC_9931211	LOC100294033	2.060E-12					LOC100294033			
V3SVHSHC_8359553	PEX11G	2.073E-12	9.856E-17				PEX11G	PEX11G		
V3SVHSHC_8864882	Dpy30	2.097E-12					Dpy30			
V3SVHSHC_8724467	TGM3	2.100E-12		2.420E-41	1.309E-01		TGM3		TGM3	
V3SVHSHC_10206464	TMCO2	2.107E-12					TMCO2			
V3SVHSHC_7949198	HJURP	2.226E-12					HJURP			
V3SVHSHC_4943690	CDK2	2.313E-12					CDK2			
V3SVHSHC_9168119	ADH4	2.343E-12					ADH4			
V3SVHSHC_9759809	NAA38	2.410E-12					NAA38			
V3SVHSHC_9185609	TRIM10	2.458E-12	2.999E-03				TRIM10			
V3SVHSHC_6272336	FAM26E	2.516E-12			4.630E-01		FAM26E			
V3SVHSHC_5590325	EPHB6	2.577E-12					EPHB6			
V3SVHSHC_5118722	NECAB2	2.667E-12	4.263E-13				NECAB2	NECAB2		
V3SVHSHC_8164622	ATP1B4	2.669E-12					ATP1B4			
V3SVHSHC_7308206	ZNF583	2.694E-12					ZNF583			
V3SVHSHC_7856072	LOC100133128	2.741E-12					LOC100133128			
V3SVHSHC_8717768	CCL24	2.764E-12					CCL24			
V3SVHSHC_8758556	SPACA4	2.806E-12					SPACA4			
V3SVHSHC_6337247	LRRN2	2.822E-12					LRRN2			
V3SVHSHC_8599529	MECOM	2.886E-12					MECOM			
V3SVHSHC_10824488	IL31RA	2.992E-12					IL31RA			
V3SVHSHC_9254348	LBP	3.027E-12		1.699E-02			LBP			
V3SVHSHC_6236201	AGA	3.116E-12					AGA			
V3SVHSHC_7268045	ZCCHC10	3.127E-12					ZCCHC10			
V3SVHSHC_6897521	CRCP	3.216E-12					CRCP			
V3SVHSHC_10570685	C9orf92	3.285E-12					C9orf92			
V3SVHSHC_8516006	OR4K5	3.454E-12					OR4K5			
V3SVHSHC_7056911	TRIB1	3.558E-12					TRIB1			
V3SVHSHC_8747963	PBX1	3.574E-12					PBX1			
V3SVHSHC_8325101	SQLE	3.645E-12					SQLE			
V3SVHSHC_10161815	PPM1J	3.648E-12			3.676E-01		PPM1J			
V3SVHSHC_8068955	GMEB2	3.664E-12		4.702E-05			GMEB2		GMEB2	
V3SVHSHC_8913029	GRXCR2	3.740E-12					GRXCR2			
V3SVHSHC_9795350	PROZ	3.802E-12					PROZ			
V3SVHSHC_4993190	CA3	4.069E-12					CA3			
V3SVHSHC_8564912	MBLAC2	4.185E-12		5.780E-09			MBLAC2		MBLAC2	
V3SVHSHC_9327905	SCML1	4.249E-12					SCML1			
V3SVHSHC_8457299	LGI2	4.496E-12					LGI2			
V3SVHSHC_4845119	FRS3	4.514E-12					FRS3			
V3SVHSHC_5204390	PI16	4.532E-12					PI16			
V3SVHSHC_4833206	PLA2G2E	4.797E-12					PLA2G2E			
V3SVHSHC_10541183	SSMEM1	4.804E-12					SSMEM1			

V3SVHSHC_5919104	SLC38A11	4.876E-12					SLC38A11			
V3SVHSHC_8619593	PPARA	4.894E-12					PPARA			
V3SVHSHC_7812842	CHIC2	4.997E-12					CHIC2			
V3SVHSHC_5363978	NFATC2IP	5.318E-12					NFATC2IP			
V3SVHSHC_10168481	ZNF727P	5.319E-12					ZNF727P			
V3SVHSHC_5017148	MCEE	5.567E-12					MCEE			
V3SVHSHC_9601541	DIABLO	5.617E-12					DIABLO			
V3SVHSHC_8165282	LOC101929578	5.672E-12					LOC101929578			
V3SVHSHC_5141525	SPAG17	5.935E-12					SPAG17			
V3SVHSHC_7837592	HOXC13	6.245E-12					HOXC13			
V3SVHSHC_9388526	CLTA	6.250E-12					CLTA			
V3SVHSHC_9505907	FOXK1	6.477E-12					FOXK1			
V3SVHSHC_9218774	ELF1	6.567E-12					ELF1			
V3SVHSHC_8698496	TLR6	6.574E-12	4.312E-08				TLR6	TLR6		
V3SVHSHC_10006550	TLR5	6.783E-12					TLR5			
V3SVHSHC_7661636	UQCC2	7.020E-12					UQCC2			
V3SVHSHC_6600587	FMO5	7.258E-12					FMO5			
V3SVHSHC_6920489	MUTYH	7.441E-12					MUTYH			
V3SVHSHC_7429217	MICA	7.781E-12					MICA			
V3SVHSHC_8104100	UBTFL1	7.797E-12					UBTFL1			
V3SVHSHC_9150926	ADAM15	8.119E-12					ADAM15			
V3SVHSHC_5116643	TSHZ3	8.122E-12					TSHZ3			
V3SVHSHC_6721070	CYB5D2	8.345E-12					CYB5D2			
V3SVHSHC_8508713	DIS3L2	8.537E-12					DIS3L2			
V3SVHSHC_8614676	PEX7	8.812E-12					PEX7			
V3SVHSHC_5184788	NYNRIN	9.357E-12					NYNRIN			
V3SVHSHC_9933257	BMP2	9.917E-12					BMP2			
V3SVHSHC_9105089	CIRBP	9.998E-12					CIRBP			
V3SVHSHC_10368494	MTNR1B	1.038E-11	4.312E-14				MTNR1B	MTNR1B		
V3SVHSHC_10524353	FAT2	1.040E-11					FAT2			
V3SVHSHC_7194224	GBP2	1.051E-11					GBP2			
V3SVHSHC_5169707	TMTC3	1.074E-11					TMTC3			
V3SVHSHC_7313420	OR5D18	1.082E-11					OR5D18			
V3SVHSHC_9042455	ERO1LB	1.155E-11					ERO1LB			
V3SVHSHC_5341340	NCBP2	1.170E-11					NCBP2			
V3SVHSHC_6649790	MYH9	1.191E-11					MYH9			
V3SVHSHC_9653714	ATP10A	1.211E-11					ATP10A			
V3SVHSHC_10400537	ERCC5	1.222E-11					ERCC5			
V3SVHSHC_5644841	WAS	1.232E-11					WAS			
V3SVHSHC_10641437	CSF1	1.270E-11		2.265E-01	3.802E-01		CSF1			
V3SVHSHC_8439644	PTPN20B	1.296E-11					PTPN20B			
V3SVHSHC_8486174	PAFAH2	1.309E-11					PAFAH2			
V3SVHSHC_8882075	SNED1	1.349E-11					SNED1			
V3SVHSHC_9066116	ZNF19	1.386E-11					ZNF19			
V3SVHSHC_5663387	ACTC1	1.435E-11					ACTC1			
V3SVHSHC_10208939	DGKI	1.464E-11		3.824E-12			DGKI		DGKI	
V3SVHSHC_9805547	PAK4	1.477E-11					PAK4			
V3SVHSHC_7213925	Pbrm1	1.508E-11					Pbrm1			
V3SVHSHC_5632763	Iqcf5	1.514E-11					Iqcf5			
V3SVHSHC_8253227	TMED7	1.548E-11					TMED7			
V3SVHSHC_8648072	LACTB	1.580E-11					LACTB			
V3SVHSHC_7444397	LTK	1.631E-11					LTK			
V3SVHSHC_5785223	OASL	1.641E-11					OASL			

V3SVHSHC_5226104	LOC101928291	1.692E-11	9.056E-05				LOC101928291	LOC101928291		
V3SVHSHC_7038992	ISL2	1.697E-11					ISL2			
V3SVHSHC_9498977	SLC10A4	1.715E-11					SLC10A4			
V3SVHSHC_5865446	FAM101B	1.740E-11					FAM101B			
V3SVHSHC_7733510	WDR78	1.747E-11					WDR78			
V3SVHSHC_8722388	POMGNT1	1.784E-11					POMGNT1			
V3SVHSHC_7945403	CLDN6	1.816E-11					CLDN6			
V3SVHSHC_9981470	ZNF565	1.951E-11					ZNF565			
V3SVHSHC_5122583	PLEKHH2	2.024E-11					PLEKHH2			
V3SVHSHC_5104565	ZFYVE20	2.030E-11					ZFYVE20			
V3SVHSHC_8323121	SLC35B3	2.090E-11					SLC35B3			
V3SVHSHC_5828024	FAM175A	2.177E-11					FAM175A			
V3SVHSHC_8952365	HEATR5B	2.272E-11					HEATR5B			
V3SVHSHC_7036682	PRDM8	2.369E-11					PRDM8			
V3SVHSHC_4987019	PRR11	2.375E-11					PRR11			
V3SVHSHC_6399650	KCNH3	2.497E-11					KCNH3			
V3SVHSHC_7440074	TRABD2B	2.592E-11					TRABD2B			
V3SVHSHC_8087072	ZNF436	2.597E-11					ZNF436			
V3SVHSHC_8581049	P2RY14	2.628E-11					P2RY14			
V3SVHSHC_10703378	GOLGA5	2.648E-11					GOLGA5			
V3SVHSHC_7578740	LDHA	2.650E-11					LDHA			
V3SVHSHC_7937351	EDDM3B	2.718E-11					EDDM3B			
V3SVHSHC_5537327	ADRBK1	2.810E-11					ADRBK1			
V3SVHSHC_9493961	RGAG1	2.828E-11					RGAG1			
V3SVHSHC_6355265	INHBB	2.880E-11					INHBB			
V3SVHSHC_7322462	ARHGAP44	2.910E-11					ARHGAP44			
V3SVHSHC_9211052	RFPL4AL1	2.962E-11	6.879E-02				RFPL4AL1			
V3SVHSHC_6944612	CCL13	2.975E-11					CCL13			
V3SVHSHC_7373777	SMCO4	2.981E-11					SMCO4			
V3SVHSHC_8477891	OR10A6	2.996E-11					OR10A6			
V3SVHSHC_4923659	SHANK1	3.166E-11					SHANK1			
V3SVHSHC_9183926	FBXO31	3.169E-11					FBXO31			
V3SVHSHC_8444660	KIAA0947	3.283E-11					KIAA0947			
V3SVHSHC_10756607	PANX1	3.340E-11					PANX1			
V3SVHSHC_4923758	LRRC4	3.366E-11					LRRC4			
V3SVHSHC_5833733	TNK1	3.441E-11					TNK1			
V3SVHSHC_7738658	SUN2	3.541E-11					SUN2			
V3SVHSHC_9154853	AURKC	3.644E-11					AURKC			
V3SVHSHC_10284113	NGDN	3.652E-11					NGDN			
V3SVHSHC_7816934	TIMM44	3.655E-11					TIMM44			
V3SVHSHC_5102651	HK3	3.761E-11					HK3			
V3SVHSHC_7328996	ZNF445	3.871E-11					ZNF445			
V3SVHSHC_6375230	VPREB1	3.932E-11					VPREB1			
V3SVHSHC_7355066	LTBP2	3.936E-11					LTBP2			
V3SVHSHC_8499737	ING5	3.972E-11					ING5			
V3SVHSHC_8015759	OR10H1	4.011E-11					OR10H1			
V3SVHSHC_6824261	CSRNP3	4.065E-11					CSRNP3			
V3SVHSHC_5642036	HTN3	4.089E-11					HTN3			
V3SVHSHC_7354637	BRINP1	4.162E-11					BRINP1			
V3SVHSHC_5062622	PFKFB1	4.207E-11					PFKFB1			
V3SVHSHC_6331274	FAM50B	4.346E-11					FAM50B			
V3SVHSHC_6011702	MS4A5	4.494E-11					MS4A5			
V3SVHSHC_6501653	SLTM	4.537E-11					SLTM			
V3SVHSHC_5979956	GPT2	4.571E-11					GPT2			

V3SVHSHC_9223592	UNC119	4.592E-11					UNC119			
V3SVHSHC_5982629	C15orf48	4.610E-11					C15orf48			
V3SVHSHC_9117365	CCK	4.611E-11					CCK			
V3SVHSHC_10017077	MDK	5.022E-11					MDK			
V3SVHSHC_7721069	TSFM	5.036E-11			7.268E-20		TSFM			TSFM
V3SVHSHC_9312824	C14ORF166	5.117E-11					C14ORF166			
V3SVHSHC_7170398	PREX1	5.282E-11					PREX1			
V3SVHSHC_7738097	SNX1	5.584E-11					SNX1			
V3SVHSHC_5209043	HEY2	5.644E-11					HEY2			
V3SVHSHC_5845679	FAM90A1	5.667E-11					FAM90A1			
V3SVHSHC_8009093	TRIQK	5.671E-11					TRIQK			
V3SVHSHC_6492215	NSL1	5.749E-11					NSL1			
V3SVHSHC_8536499	PI15	5.865E-11					PI15			
V3SVHSHC_8126408	TRIM60	5.924E-11					TRIM60			
V3SVHSHC_5335664	MACC1	6.374E-11					MACC1			
V3SVHSHC_6726614	Tti2	6.398E-11					Tti2			
V3SVHSHC_6680480	C1orf123	6.399E-11					C1orf123			
V3SVHSHC_10298930	DIO3	6.454E-11					DIO3			
V3SVHSHC_8480333	NPBWR2	6.580E-11					NPBWR2			
V3SVHSHC_6078923	AHSG	6.633E-11					AHSG			
V3SVHSHC_8450369	PTPN22	6.657E-11					PTPN22			
V3SVHSHC_5769581	MYO1B	6.714E-11					MYO1B			
V3SVHSHC_6955469	FSD2	7.087E-11					FSD2			
V3SVHSHC_7873199	KRT5	7.131E-11					KRT5			
V3SVHSHC_5192873	LOC646862	7.550E-11					LOC646862			
V3SVHSHC_9842672	DZANK1	7.579E-11					DZANK1			
V3SVHSHC_4968605	NPAS1	7.633E-11			3.166E-01		NPAS1			
V3SVHSHC_9656552	NEU1	7.782E-11					NEU1			
V3SVHSHC_7424828	COX19	8.584E-11					COX19			
V3SVHSHC_10441490	ACAT1	8.591E-11			1.167E-01		ACAT1			
V3SVHSHC_4738859	FERD3L	8.801E-11					FERD3L			
V3SVHSHC_9805349	KLHL33	8.854E-11					KLHL33			
V3SVHSHC_5195909	ACOT9	8.914E-11					ACOT9			
V3SVHSHC_6938045	LOC100129083	9.039E-11		1.226E-14			LOC10012908 3		LOC10012 9083	
V3SVHSHC_7572437	OR4F17	9.169E-11					OR4F17			
V3SVHSHC_8757302	C20ORF85	9.198E-11					C20ORF85			
V3SVHSHC_9827459	VGF	9.486E-11					VGF			
V3SVHSHC_9593093	LEMD1	9.647E-11					LEMD1			
V3SVHSHC_7429679	VSTM2B	9.761E-11					VSTM2B			
V3SVHSHC_5579138	RTKN	9.763E-11					RTKN			
V3SVHSHC_10462511	CALCOCO2	9.880E-11					CALCOCO2			
V3SVHSHC_9007970	DDX58	1.096E-10					DDX58			
V3SVHSHC_5334014	PI4K2B	1.102E-10					PI4K2B			
V3SVHSHC_5646392	GPRIN1	1.113E-10					GPRIN1			
V3SVHSHC_7199438	RPL36A-HNRNPH2	1.147E-10					RPL36A-HNRNPH2			
V3SVHSHC_4796543	DNAJB1	1.171E-10		2.842E-19			DNAJB1		DNAJB1	
V3SVHSHC_6113540	POLE3	1.171E-10					POLE3			
V3SVHSHC_9623024	ZNF233	1.240E-10					ZNF233			
V3SVHSHC_6437666	Trappc10	1.244E-10					Trappc10			
V3SVHSHC_9079316	HIRIP3	1.281E-10					HIRIP3			
V3SVHSHC_10354337	IL22RA2	1.287E-10					IL22RA2			
V3SVHSHC_10503167	CAMK2N2	1.293E-10					CAMK2N2			
V3SVHSHC_5192081	SLCO5A1	1.347E-10					SLCO5A1			

V3SVHSHC_6802613	ZNF551	1.425E-10					ZNF551			
V3SVHSHC_5729321	QRICH2	1.469E-10					QRICH2			
V3SVHSHC_7685297	TMEM240	1.473E-10					TMEM240			
V3SVHSHC_9008432	HCAR2	1.495E-10					HCAR2			
V3SVHSHC_6995927	ZYX	1.549E-10					ZYX			
V3SVHSHC_7435124	TFEC	1.563E-10					TFEC			
V3SVHSHC_9213131	RTFDC1	1.598E-10					RTFDC1			
V3SVHSHC_10558739	SMIM13	1.672E-10					SMIM13			
V3SVHSHC_5478059	CATIP	1.676E-10			1.570E-01		CATIP			
V3SVHSHC_7039586	CACNG3	1.759E-10					CACNG3			
V3SVHSHC_9821057	TSTA3	1.802E-10					TSTA3			
V3SVHSHC_10463567	CNTNAP4	1.873E-10					CNTNAP4			
V3SVHSHC_8517227	OR6K3	1.906E-10					OR6K3			
V3SVHSHC_7900523	DTWD2	1.958E-10					DTWD2			
V3SVHSHC_9225275	PAPL	1.991E-10					PAPL			
V3SVHSHC_9378923	LGALSL	2.024E-10					LGALSL			
V3SVHSHC_7350413	ZNF263	2.081E-10					ZNF263			
V3SVHSHC_7486736	ACTG1	2.115E-10					ACTG1			
V3SVHSHC_8606261	ARID2	2.194E-10					ARID2			
V3SVHSHC_8722586	SPTBN1	2.290E-10					SPTBN1			
V3SVHSHC_10298897	CSNK2A2	2.315E-10					CSNK2A2			
V3SVHSHC_5488223	CCDC104	2.411E-10					CCDC104			
V3SVHSHC_5012396	CPNE8	2.444E-10					CPNE8			
V3SVHSHC_5210891	HAS1	2.480E-10					HAS1			
V3SVHSHC_9216134	WDR17	2.567E-10					WDR17			
V3SVHSHC_6753410	PCDHA13	2.599E-10			2.894E-03		PCDHA13			
V3SVHSHC_6606758	MAGEC3	2.768E-10					MAGEC3			
V3SVHSHC_6339425	MS4A6E	2.804E-10					MS4A6E			
V3SVHSHC_5490038	LITAF	2.866E-10					LITAF			
V3SVHSHC_9330776	NLK	2.871E-10					NLK			
V3SVHSHC_9974243	CT47A9	2.887E-10					CT47A9			
V3SVHSHC_8070011	B4GALT7	2.888E-10					B4GALT7			
V3SVHSHC_9749018	NDUFB5	2.955E-10					NDUFB5			
V3SVHSHC_10652459	PJA2	3.004E-10					PJA2			
V3SVHSHC_6513764	AMTN	3.131E-10					AMTN			
V3SVHSHC_7776014	SERPINB3	3.185E-10					SERPINB3			
V3SVHSHC_5881748	ORM2	3.312E-10					ORM2			
V3SVHSHC_6192872	POLA2	3.314E-10					POLA2			
V3SVHSHC_7206434	PERP	3.318E-10					PERP			
V3SVHSHC_9861086	MBOAT4	3.396E-10					MBOAT4			
V3SVHSHC_7110965	TTC18	3.437E-10					TTC18			
V3SVHSHC_8380772	Tarm1	3.447E-10		2.263E-03			Tarm1			
V3SVHSHC_8219963	NEMF	3.477E-10					NEMF			
V3SVHSHC_9073937	KIAA1045	3.511E-10					KIAA1045			
V3SVHSHC_5809676	DISP1	3.560E-10					DISP1			
V3SVHSHC_9077468	NEK9	3.576E-10					NEK9			
V3SVHSHC_5075228	MCM2	3.618E-10					MCM2			
V3SVHSHC_9521318	PEX11B	3.775E-10					PEX11B			
V3SVHSHC_10332722	VPS37C	3.880E-10					VPS37C			
V3SVHSHC_5334839	ACTN4	4.101E-10					ACTN4			
V3SVHSHC_7788125	GMNN	4.170E-10					GMNN			
V3SVHSHC_6270554	PLCB4	4.188E-10					PLCB4			
V3SVHSHC_10092713	GPBP1	4.213E-10					GPBP1			
V3SVHSHC_9651932	TRHDE	4.354E-10					TRHDE			
V3SVHSHC_7671833	PALMD	4.382E-10					PALMD			

V3SVHSHC_8557454	DDX51	4.390E-10					DDX51			
V3SVHSHC_8546663	RORA	4.616E-10					RORA			
V3SVHSHC_7356551	PKD1L2	5.073E-10					PKD1L2			
V3SVHSHC_5844458	LOC101929702	5.109E-10					LOC101929702			
V3SVHSHC_9346055	ZC3H3	5.294E-10					ZC3H3			
V3SVHSHC_6237686	Dcaf17	5.737E-10					Dcaf17			
V3SVHSHC_8443868	MAP7	6.498E-10					MAP7			
V3SVHSHC_9438488	CCDC149	6.546E-10					CCDC149			
V3SVHSHC_5979692	SEPT14	6.549E-10					SEPT14			
V3SVHSHC_8784824	NNAT	6.809E-10					NNAT			
V3SVHSHC_7336817	FUT5	6.817E-10					FUT5			
V3SVHSHC_9291044	LOC100294341	6.826E-10					LOC100294341			
V3SVHSHC_9387371	MAMDC2	7.112E-10					MAMDC2			
V3SVHSHC_7219634	LSG1	7.486E-10					LSG1			
V3SVHSHC_10764362	ELP2	7.590E-10					ELP2			
V3SVHSHC_7745984	HAVCR1	7.612E-10					HAVCR1			
V3SVHSHC_5877458	AQP7	7.803E-10					AQP7			
V3SVHSHC_8352359	OR52W1	7.844E-10					OR52W1			
V3SVHSHC_9624047	KIAA0922	8.367E-10					KIAA0922			
V3SVHSHC_9691037	GPR83	8.442E-10					GPR83			
V3SVHSHC_10049516	GYS1	8.701E-10					GYS1			
V3SVHSHC_5526800	CBFB	8.811E-10					CBFB			
V3SVHSHC_9198875	PPM1N	8.855E-10					PPM1N			
V3SVHSHC_9264710	CCR9	9.027E-10					CCR9			
V3SVHSHC_9354635	MAMDC4	9.087E-10					MAMDC4			
V3SVHSHC_10825775	NRXN2	9.494E-10					NRXN2			
V3SVHSHC_6798719	TAAR9	9.879E-10					TAAR9			
V3SVHSHC_10223855	C1orf229	1.004E-09					C1orf229			
V3SVHSHC_4731434	SLC30A1	1.043E-09					SLC30A1			
V3SVHSHC_7656719	DBR1	1.049E-09					DBR1			
V3SVHSHC_4788458	RAB11FIP3	1.073E-09					RAB11FIP3			
V3SVHSHC_9298403	LYVE1	1.136E-09					LYVE1			
V3SVHSHC_4654709	TYW1	1.140E-09					TYW1			
V3SVHSHC_6737999	NUMB	1.143E-09					NUMB			
V3SVHSHC_7303751	LOC101060521	1.168E-09					LOC101060521			
V3SVHSHC_9477131	LZTS1	1.176E-09					LZTS1			
V3SVHSHC_7712720	LILRB5	1.179E-09					LILRB5			
V3SVHSHC_9955532	TPD52	1.184E-09					TPD52			
V3SVHSHC_6806375	SCRT2	1.197E-09					SCRT2			
V3SVHSHC_5315303	ADH1A	1.211E-09					ADH1A			
V3SVHSHC_4710248	BTF3	1.235E-09					BTF3			
V3SVHSHC_6129149	SDHB	1.249E-09					SDHB			
V3SVHSHC_10019981	MRPS30	1.262E-09					MRPS30			
V3SVHSHC_7946789	TEX30	1.281E-09					TEX30			
V3SVHSHC_10296818	SYN1	1.341E-09					SYN1			
V3SVHSHC_6875213	STRADA	1.345E-09					STRADA			
V3SVHSHC_10306058	MMRN2	1.369E-09					MMRN2			
V3SVHSHC_4791329	FYN	1.402E-09					FYN			
V3SVHSHC_6445982	ANKRD54	1.415E-09					ANKRD54			
V3SVHSHC_6942203	ACAA2	1.446E-09					ACAA2			
V3SVHSHC_7616591	AKAP14	1.486E-09					AKAP14			
V3SVHSHC_5724734	FRS3	1.509E-09					FRS3			
V3SVHSHC_10301504	FAAH2	1.520E-09			2.864E-10		FAAH2			FAAH2
V3SVHSHC_5955041	SEPT8	1.522E-09					SEPT8			

V3SVHSHC_5633291	AQP4	1.562E-09					AQP4			
V3SVHSHC_6062720	WBP4	1.639E-09					WBP4			
V3SVHSHC_6956921	NAPRT1	1.679E-09					NAPRT1			
V3SVHSHC_9977873	LRRC8D	1.770E-09					LRRC8D			
V3SVHSHC_7636754	NWD1	1.823E-09					NWD1			
V3SVHSHC_9919595	MYH3	1.842E-09					MYH3			
V3SVHSHC_6622730	CLPP	1.885E-09					CLPP			
V3SVHSHC_7506470	TTC7A	1.901E-09					TTC7A			
V3SVHSHC_7955567	OR7A10	1.936E-09					OR7A10			
V3SVHSHC_9638501	GNL3L	2.026E-09					GNL3L			
V3SVHSHC_9617018	LYG1	2.052E-09					LYG1			
V3SVHSHC_9675560	LOC101929065	2.069E-09					LOC101929065			
V3SVHSHC_5048630	SESN2	2.146E-09					SESN2			
V3SVHSHC_5216930	C21ORF62	2.156E-09					C21ORF62			
V3SVHSHC_10763801	TM7SF2	2.170E-09					TM7SF2			
V3SVHSHC_6918245	EML2	2.183E-09					EML2			
V3SVHSHC_8160860	GYPC	2.249E-09					GYPC			
V3SVHSHC_9077501	IDO2	2.254E-09					IDO2			
V3SVHSHC_8916791	BAD	2.276E-09					BAD			
V3SVHSHC_9004868	OR52B6	2.348E-09					OR52B6			
V3SVHSHC_5275010	KCNH5	2.402E-09					KCNH5			
V3SVHSHC_8789081	CAMK2B	2.515E-09					CAMK2B			
V3SVHSHC_8998466	OCM	2.606E-09		5.737E-52	1.058E-03		OCM		OCM	
V3SVHSHC_9652493	LIF	2.798E-09					LIF			
V3SVHSHC_9389318	PDCD6IP	2.842E-09					PDCD6IP			
V3SVHSHC_5982827	TRUB2	2.887E-09					TRUB2			
V3SVHSHC_5978867	ZMPSTE24	2.901E-09					ZMPSTE24			
V3SVHSHC_9609494	SDHC	2.978E-09					SDHC			
V3SVHSHC_10297874	WBSCR28	3.035E-09		1.413E-10			WBSCR28		WBSCR28	
V3SVHSHC_6734006	SMPX	3.088E-09					SMPX			
V3SVHSHC_9058691	ICAM1	3.195E-09					ICAM1			
V3SVHSHC_7529372	PI3	3.249E-09					PI3			
V3SVHSHC_10783733	ANKRD55	3.266E-09					ANKRD55			
V3SVHSHC_8316983	BRSK2	3.380E-09					BRSK2			
V3SVHSHC_8046152	KCNB2	3.493E-09					KCNB2			
V3SVHSHC_9414794	PEX5L	3.501E-09					PEX5L			
V3SVHSHC_10136867	KIAA1211	3.518E-09					KIAA1211			
V3SVHSHC_6474098	CDH26	3.553E-09					CDH26			
V3SVHSHC_7504919	CCDC30	3.560E-09					CCDC30			
V3SVHSHC_10792808	ACTR8	3.618E-09					ACTR8			
V3SVHSHC_5356982	PIGW	3.626E-09			3.581E-10		PIGW			PIGW
V3SVHSHC_5847824	KCNJ9	3.645E-09					KCNJ9			
V3SVHSHC_8808155	FOXJ1	3.663E-09					FOXJ1			
V3SVHSHC_10546430	LST1	4.095E-09					LST1			
V3SVHSHC_10655330	ZNF606	4.117E-09					ZNF606			
V3SVHSHC_7115321	S100A7	4.118E-09					S100A7			
V3SVHSHC_6162380	ZNF311	4.122E-09					ZNF311			
V3SVHSHC_5639891	FABP2	4.323E-09					FABP2			
V3SVHSHC_6956789	RIPK2	4.498E-09					RIPK2			
V3SVHSHC_10831550	PGC	4.516E-09					PGC			
V3SVHSHC_5491127	ANKRD34B	4.588E-09					ANKRD34B			
V3SVHSHC_7770173	PKN3	4.899E-09					PKN3			
V3SVHSHC_7251116	ALG12	5.035E-09					ALG12			
V3SVHSHC_9758918	GCFC2	5.488E-09					GCFC2			

V3SVHSHC_10587185	FAM118B	5.596E-09					FAM118B			
V3SVHSHC_8346254	ALG14	5.614E-09					ALG14			
V3SVHSHC_7671602	TP53BP2	5.789E-09					TP53BP2			
V3SVHSHC_9554021	AIM1L	6.135E-09					AIM1L			
V3SVHSHC_10644407	FCHSD2	6.210E-09					FCHSD2			
V3SVHSHC_5058992	APOL5	6.265E-09					APOL5			
V3SVHSHC_10652426	GALC	6.424E-09					GALC			
V3SVHSHC_7462547	ATP6V1A	6.527E-09					ATP6V1A			
V3SVHSHC_9769709	SIRT1	6.731E-09					SIRT1			
V3SVHSHC_7948505	ERGIC2	6.755E-09					ERGIC2			
V3SVHSHC_6872243	HOMER1	6.977E-09					HOMER1			
V3SVHSHC_9588341	LCN12	7.013E-09					LCN12			
V3SVHSHC_6469181	EPS15L1	7.060E-09					EPS15L1			
V3SVHSHC_5847296	FAM109A	7.325E-09					FAM109A			
V3SVHSHC_10290482	RLTPR	7.335E-09					RLTPR			
V3SVHSHC_8922302	Phax	7.855E-09					Phax			
V3SVHSHC_9961637	ZDHHC15	8.337E-09					ZDHHC15			
V3SVHSHC_5768393	CD70	8.358E-09					CD70			
V3SVHSHC_8391563	GRXCR1	8.492E-09					GRXCR1			
V3SVHSHC_8189174	PIH1D1	9.049E-09					PIH1D1			
V3SVHSHC_5019623	NPPB	9.077E-09					NPPB			
V3SVHSHC_5715065	ZNF554	9.081E-09					ZNF554			
V3SVHSHC_6304841	CRTAP	9.446E-09					CRTAP			
V3SVHSHC_6924350	EBI3	1.022E-08					EBI3			
V3SVHSHC_8494325	CD83	1.027E-08					CD83			
V3SVHSHC_8206862	UPRT	1.046E-08					UPRT			
V3SVHSHC_8349950	GRK4	1.066E-08					GRK4			
V3SVHSHC_8399747	SCAMP3	1.175E-08					SCAMP3			
V3SVHSHC_7697870	OR52K2	1.206E-08		1.082E-18			OR52K2		OR52K2	
V3SVHSHC_8089877	PDLIM2	1.223E-08					PDLIM2			
V3SVHSHC_9550226	ETFA	1.237E-08					ETFA			
V3SVHSHC_6758393	RNFT2	1.256E-08					RNFT2			
V3SVHSHC_5286560	MRPS6	1.307E-08					MRPS6			
V3SVHSHC_7938044	HEATR2	1.376E-08					HEATR2			
V3SVHSHC_5128721	BAAT	1.379E-08					BAAT			
V3SVHSHC_8128421	WBP11	1.396E-08					WBP11			
V3SVHSHC_6943919	PRKCD	1.478E-08					PRKCD			
V3SVHSHC_7411496	TMEM243	1.618E-08					TMEM243			
V3SVHSHC_7896332	PAPOLB	1.657E-08					PAPOLB			
V3SVHSHC_6426248	PHKA1	1.713E-08					PHKA1			
V3SVHSHC_9541481	ZMYM2	1.755E-08					ZMYM2			
V3SVHSHC_4874489	SETDB2	1.774E-08					SETDB2			
V3SVHSHC_8459048	ZNF555	1.776E-08			4.878E-01		ZNF555			
V3SVHSHC_7253030	C1orf127	1.789E-08					C1orf127			
V3SVHSHC_10538180	EDF1	1.819E-08					EDF1			
V3SVHSHC_6808157	LOC101929264	1.908E-08					LOC10192926<br>4			
V3SVHSHC_8284907	LOC100996713	1.979E-08					LOC10099671<br>3			
V3SVHSHC_10061033	NUP62CL	1.995E-08					NUP62CL			
V3SVHSHC_6856898	YBX3	2.081E-08					YBX3			
V3SVHSHC_10706744	COX6B2	2.085E-08					COX6B2			
V3SVHSHC_10283948	DAGLB	2.229E-08					DAGLB			
V3SVHSHC_9418358	SAA4	2.232E-08			7.352E-03		SAA4			
V3SVHSHC_9048560	CASP12	2.251E-08					CASP12			
V3SVHSHC_9366317	FOSB	2.451E-08					FOSB			

V3SVHSHC_6852740	MTCP1	2.474E-08					MTCP1			
V3SVHSHC_7169573	SBK1	2.512E-08					SBK1			
V3SVHSHC_9302726	NPAP1	2.547E-08					NPAP1			
V3SVHSHC_10466009	CTBP1	2.694E-08					CTBP1			
V3SVHSHC_5652596	STPG1	2.707E-08					STPG1			
V3SVHSHC_4638869	CACNA1C	2.777E-08					CACNA1C			
V3SVHSHC_6762056	CLEC16A	2.863E-08					CLEC16A			
V3SVHSHC_9825875	FAM20C	2.863E-08			4.429E-13		FAM20C			FAM20C
V3SVHSHC_6088988	OR4F15	2.868E-08					OR4F15			
V3SVHSHC_10242236	Gpank1	2.884E-08					Gpank1			
V3SVHSHC_9601112	Pstk	2.973E-08					Pstk			
V3SVHSHC_6334079	C9ORF9	3.076E-08					C9ORF9			
V3SVHSHC_8169968	SH2D1B	3.240E-08					SH2D1B			
V3SVHSHC_10759907	LOC646862	3.354E-08		4.990E-17			LOC646862		LOC646862	
V3SVHSHC_5898545	S100B	3.416E-08					S100B			
V3SVHSHC_9455582	TMEM91	3.513E-08					TMEM91			
V3SVHSHC_8178020	SYS1	3.556E-08			3.420E-01		SYS1			
V3SVHSHC_9644111	LOC101929936	3.596E-08					LOC101929936			
V3SVHSHC_9820496	CRIP2	3.635E-08					CRIP2			
V3SVHSHC_7017938	CCDC127	3.672E-08					CCDC127			
V3SVHSHC_6160763	ZNF786	3.749E-08					ZNF786			
V3SVHSHC_5792747	ARHGAP44	3.836E-08					ARHGAP44			
V3SVHSHC_6953291	IL11	4.127E-08					IL11			
V3SVHSHC_4748297	IQCD	4.384E-08					IQCD			
V3SVHSHC_9571214	PDCD7	4.426E-08					PDCD7			
V3SVHSHC_6685958	NLGN3	4.461E-08					NLGN3			
V3SVHSHC_5187164	PKP1	4.642E-08					PKP1			
V3SVHSHC_9338795	APLN	4.765E-08					APLN			
V3SVHSHC_10507226	NAT8B	4.852E-08					NAT8B			
V3SVHSHC_8122085	LOC101927375	5.152E-08					LOC101927375			
V3SVHSHC_5949299	PPP1R17	5.432E-08					PPP1R17			
V3SVHSHC_7007642	RBBP5	5.469E-08					RBBP5			
V3SVHSHC_9911048	CD81	5.490E-08					CD81			
V3SVHSHC_7449545	OR10A3	5.673E-08					OR10A3			
V3SVHSHC_10024898	NUPL1	5.736E-08					NUPL1			
V3SVHSHC_8369255	Aspg	5.766E-08					Aspg			
V3SVHSHC_10520888	FSCN1	5.798E-08					FSCN1			
V3SVHSHC_5461526	SERINC4	6.094E-08					SERINC4			
V3SVHSHC_5931380	AFF1	6.258E-08					AFF1			
V3SVHSHC_7760900	BCL6	6.429E-08					BCL6			
V3SVHSHC_8131193	DIAPH2	6.917E-08					DIAPH2			
V3SVHSHC_7543232	VPS51	7.133E-08					VPS51			
V3SVHSHC_9188942	DSP	7.288E-08		1.059E-12			DSP		DSP	
V3SVHSHC_6954017	OR4K14	8.370E-08					OR4K14			
V3SVHSHC_7871054	TMED10	8.543E-08					TMED10			
V3SVHSHC_8940881	CREB3L2	8.543E-08					CREB3L2			
V3SVHSHC_5538020	SRP68	8.745E-08					SRP68			
V3SVHSHC_9161354	PLAGL2	8.851E-08					PLAGL2			
V3SVHSHC_10442843	RUFY3	9.107E-08					RUFY3			
V3SVHSHC_10713707	NIN	9.362E-08					NIN			
V3SVHSHC_8203067	SLC35G3	9.424E-08					SLC35G3			
V3SVHSHC_10606655	PRRG4	9.954E-08					PRRG4			
V3SVHSHC_7288307	Ercc6l	1.001E-07					Ercc6l			

V3SVHSHC_8224682	RHOA	1.023E-07					RHOA			
V3SVHSHC_4672298	VIMP	1.069E-07					VIMP			
V3SVHSHC_9496832	LDB1	1.080E-07					LDB1			
V3SVHSHC_6088823	NLRP7	1.093E-07					NLRP7			
V3SVHSHC_8036714	MINK1	1.153E-07					MINK1			
V3SVHSHC_9672524	ZCCHC10	1.166E-07					ZCCHC10			
V3SVHSHC_7452779	TEP1	1.209E-07					TEP1			
V3SVHSHC_4661177	GABRP	1.225E-07					GABRP			
V3SVHSHC_6874718	CNTROB	1.263E-07					CNTROB			
V3SVHSHC_7780502	MME	1.342E-07					MME			
V3SVHSHC_7895408	POLR3G	1.402E-07					POLR3G			
V3SVHSHC_9652691	LIN28B	1.468E-07					LIN28B			
V3SVHSHC_10670345	SLC25A46	1.470E-07		3.887E-19			SLC25A46		SLC25A46	
V3SVHSHC_8239532	LY6E	1.588E-07					LY6E			
V3SVHSHC_10555274	Tmco5a	1.597E-07					Tmco5a			
V3SVHSHC_4896698	IGSF5	1.622E-07					IGSF5			
V3SVHSHC_5995565	ZNF682	1.631E-07					ZNF682			
V3SVHSHC_8445617	ESRP2	1.673E-07					ESRP2			
V3SVHSHC_6294479	SPCS3	1.692E-07					SPCS3			
V3SVHSHC_9305861	SPARCL1	1.699E-07					SPARCL1			
V3SVHSHC_5269334	IFNA2	1.704E-07		1.590E-22			IFNA2		IFNA2	
V3SVHSHC_6747536	TMX4	1.734E-07					TMX4			
V3SVHSHC_10344866	PITX1	1.802E-07					PITX1			
V3SVHSHC_10371233	LOC441239	1.850E-07					LOC441239			
V3SVHSHC_8281706	EBF4	1.980E-07					EBF4			
V3SVHSHC_6935174	RBP4	1.995E-07			3.276E-02		RBP4			
V3SVHSHC_8579762	AAAS	2.068E-07					AAAS			
V3SVHSHC_7921247	PDP1	2.072E-07					PDP1			
V3SVHSHC_8295995	EARS2	2.096E-07					EARS2			
V3SVHSHC_9067568	NHLRC4	2.127E-07					NHLRC4			
V3SVHSHC_7417304	ANXA8L1	2.159E-07					ANXA8L1			
V3SVHSHC_9388097	CST3	2.199E-07					CST3			
V3SVHSHC_5037212	LOC101928058	2.229E-07					LOC10192805<br>8			
V3SVHSHC_8741957	PTRH1	2.242E-07					PTRH1			
V3SVHSHC_8100041	APOL6	2.312E-07					APOL6			
V3SVHSHC_8366054	POTEA	2.408E-07					POTEA			
V3SVHSHC_8825975	RBM34	2.471E-07					RBM34			
V3SVHSHC_10447133	RNF169	2.495E-07					RNF169			
V3SVHSHC_6472118	SULT1E1	2.505E-07					SULT1E1			
V3SVHSHC_8921048	IGF1	2.511E-07					IGF1			
V3SVHSHC_9014075	NOL7	2.532E-07					NOL7			
V3SVHSHC_5593031	RNF219	2.533E-07					RNF219			
V3SVHSHC_8790368	MRPL39	2.557E-07					MRPL39			
V3SVHSHC_8304905	NPM3	2.626E-07					NPM3			
V3SVHSHC_9996386	C3orf27	2.634E-07					C3orf27			
V3SVHSHC_9560324	SOX30	2.653E-07					SOX30			
V3SVHSHC_9026120	OXSR1	2.749E-07					OXSR1			
V3SVHSHC_9600848	AREL1	2.755E-07		2.134E-07			AREL1		AREL1	
V3SVHSHC_8953388	PABPC4L	2.830E-07					PABPC4L			
V3SVHSHC_6525776	RNF133	2.869E-07					RNF133			
V3SVHSHC_8916395	ATG16L2	3.129E-07					ATG16L2			
V3SVHSHC_5441693	ALLC	3.321E-07					ALLC			
V3SVHSHC_7423112	PPP1R2	3.475E-07					PPP1R2			
V3SVHSHC_7340645	DIRC2	3.550E-07					DIRC2			

V3SVHSHC_10269362	DNAJB12	3.665E-07					DNAJB12		
V3SVHSHC_4774268	MRPS36	3.743E-07					MRPS36		
V3SVHSHC_10769906	PPP1R12A	3.822E-07					PPP1R12A		
V3SVHSHC_4821392	GPR137B	3.844E-07					GPR137B		
V3SVHSHC_6621542	POU5F2	3.927E-07					POU5F2		
V3SVHSHC_8885111	PRR32	4.036E-07					PRR32		
V3SVHSHC_8572172	HMGB3	4.142E-07					HMGB3		
V3SVHSHC_9085355	LOC101928291	4.398E-07					LOC10192829<br>1		
V3SVHSHC_9433769	UQCRQ	4.476E-07					UQCRQ		
V3SVHSHC_10047470	SLC39A8	4.575E-07		3.454E-13			SLC39A8		SLC39A8	
V3SVHSHC_8891942	ARMCX5	4.646E-07	3.231E-11				ARMCX5	ARMCX5		
V3SVHSHC_7324046	RSRC1	4.739E-07		1.573E-17			RSRC1		RSRC1	
V3SVHSHC_4688138	ZNF550	4.962E-07			4.316E-01		ZNF550			
V3SVHSHC_6449150	KCNG4	5.126E-07					KCNG4			
V3SVHSHC_5836967	MYT1	5.282E-07			3.354E-08		MYT1			MYT1
V3SVHSHC_9972131	DEFB115	5.957E-07					DEFB115			
V3SVHSHC_6253031	BPIFB2	5.981E-07					BPIFB2			
V3SVHSHC_9179042	MEGF10	6.066E-07					MEGF10			
V3SVHSHC_10815446	EDDM3A	6.084E-07					EDDM3A			
V3SVHSHC_5779448	FBLN2	6.090E-07					FBLN2			
V3SVHSHC_9768950	RAB33A	6.148E-07					RAB33A			
V3SVHSHC_8717966	ADAMTS19	6.365E-07					ADAMTS19			
V3SVHSHC_5894585	ZNF701	6.919E-07					ZNF701			
V3SVHSHC_9458255	MTRF1L	6.991E-07					MTRF1L			
V3SVHSHC_8927978	PRG4	7.009E-07					PRG4			
V3SVHSHC_8416808	POLL	7.024E-07					POLL			
V3SVHSHC_7748162	JAM3	7.037E-07		7.948E-08			JAM3		JAM3	
V3SVHSHC_4784300	SMG9	7.081E-07					SMG9			
V3SVHSHC_9879137	SHBG	7.356E-07			9.545E-03		SHBG			
V3SVHSHC_9736313	OR4N2	7.367E-07					OR4N2			
V3SVHSHC_10384070	RUFY3	8.069E-07			3.848E-01		RUFY3			
V3SVHSHC_5266034	PAGE1	8.293E-07					PAGE1			
V3SVHSHC_6556598	TRIM34	8.787E-07					TRIM34			
V3SVHSHC_4862411	DSG2	8.833E-07					DSG2			
V3SVHSHC_7916462	LPL	9.422E-07					LPL			
V3SVHSHC_8350049	CHRDL2	9.795E-07		1.536E-12			CHRDL2		CHRDL2	
V3SVHSHC_7490498	ZNF665	9.796E-07					ZNF665			
V3SVHSHC_7187426	WDR72	9.797E-07					WDR72			
V3SVHSHC_9752681	FAM47A	1.079E-06					FAM47A			
V3SVHSHC_6415688	CDH19	1.142E-06					CDH19			
V3SVHSHC_5502512	HLA-F	1.254E-06					HLA-F			
V3SVHSHC_7377572	SLC38A9	1.256E-06					SLC38A9			
V3SVHSHC_9364634	CLK4	1.295E-06					CLK4			
V3SVHSHC_7715393	TMEM17	1.324E-06					TMEM17			
V3SVHSHC_5047871	GSTM5	1.387E-06					GSTM5			
V3SVHSHC_6700577	SKIV2L2	1.401E-06					SKIV2L2			
V3SVHSHC_6312134	ZNF572	1.405E-06					ZNF572			
V3SVHSHC_10044038	CNNM3	1.419E-06					CNNM3			
V3SVHSHC_10450103	MIR205HG	1.420E-06					MIR205HG			
V3SVHSHC_5391104	TROAP	1.505E-06					TROAP			
V3SVHSHC_5244749	SGSH	1.514E-06					SGSH			
V3SVHSHC_8626292	ZCCHC10	1.537E-06					ZCCHC10			
V3SVHSHC_8544518	MFI2	1.651E-06	2.232E-23				MFI2	MFI2		
V3SVHSHC_10458551	ZNF615	1.698E-06					ZNF615			

V3SVHSHC_10079909	ING1	1.706E-06					ING1			
V3SVHSHC_5010020	CNOT11	1.834E-06					CNOT11			
V3SVHSHC_9989192	PVALB	1.994E-06					PVALB			
V3SVHSHC_9152840	Snrnp70	2.065E-06					Snrnp70			
V3SVHSHC_5906399	GPRC5B	2.111E-06					GPRC5B			
V3SVHSHC_7416446	MTHFD2L	2.162E-06					MTHFD2L			
V3SVHSHC_5269301	PLBD1	2.200E-06					PLBD1			
V3SVHSHC_6703151	PIGT	2.219E-06					PIGT			
V3SVHSHC_5228183	CXCL1	2.238E-06					CXCL1			
V3SVHSHC_7835249	OR5AS1	2.259E-06					OR5AS1			
V3SVHSHC_10160099	SMAD1	2.322E-06					SMAD1			
V3SVHSHC_4713614	CDC37	2.367E-06					CDC37			
V3SVHSHC_10646618	P4HB	2.452E-06					P4HB			
V3SVHSHC_6600389	LGI1	2.456E-06					LGI1			
V3SVHSHC_9082352	NOMO2	2.511E-06					NOMO2			
V3SVHSHC_5909105	NDUFB11	2.597E-06					NDUFB11			
V3SVHSHC_6587420	RASSF10	2.673E-06					RASSF10			
V3SVHSHC_9860459	MNDA	2.703E-06					MNDA			
V3SVHSHC_5647844	RHPN1	2.780E-06					RHPN1			
V3SVHSHC_9540128	PRMT9	2.950E-06			5.828E-19		PRMT9			PRMT9
V3SVHSHC_8705129	SGSH	2.962E-06					SGSH			
V3SVHSHC_9736280	LRRC8E	3.040E-06					LRRC8E			
V3SVHSHC_5187428	SHANK1	3.049E-06					SHANK1			
V3SVHSHC_9962990	PPP1R14D	3.125E-06					PPP1R14D			
V3SVHSHC_4702328	LCN2	3.164E-06					LCN2			
V3SVHSHC_5259467	MAFG	3.184E-06					MAFG			
V3SVHSHC_6472184	LRP10	3.237E-06					LRP10			
V3SVHSHC_9966026	RNF150	3.542E-06					RNF150			
V3SVHSHC_6072587	MILR1	3.584E-06					MILR1			
V3SVHSHC_8976125	IRX5	3.668E-06					IRX5			
V3SVHSHC_7144262	HIST2H2AA4	3.822E-06					HIST2H2AA4			
V3SVHSHC_10432778	PALMD	3.902E-06	2.370E-04				PALMD	PALMD		
V3SVHSHC_5902901	SYT13	3.909E-06					SYT13			
V3SVHSHC_7629560	COL3A1	4.001E-06		2.279E-06			COL3A1		COL3A1	
V3SVHSHC_9163136	USP51	4.116E-06					USP51			
V3SVHSHC_10427432	LACE1	4.201E-06					LACE1			
V3SVHSHC_8489078	UCHL5	4.241E-06					UCHL5			
V3SVHSHC_9740999	DAXX	4.378E-06					DAXX			
V3SVHSHC_5506340	ADD3	4.437E-06					ADD3			
V3SVHSHC_9435881	WDR62	4.788E-06					WDR62			
V3SVHSHC_8327279	PSMD12	5.380E-06					PSMD12			
V3SVHSHC_6035132	FGF13	5.521E-06					FGF13			
V3SVHSHC_8300351	NPPB	5.579E-06					NPPB			
V3SVHSHC_7828649	C17orf67	5.673E-06					C17orf67			
V3SVHSHC_10592696	ODAM	5.938E-06					ODAM			
V3SVHSHC_4978241	FAM96B	6.135E-06					FAM96B			
V3SVHSHC_9820859	POLR2K	6.373E-06					POLR2K			
V3SVHSHC_9001370	LOC101929748	6.410E-06					LOC101929748			
V3SVHSHC_6244187	SRRM4	6.674E-06					SRRM4			
V3SVHSHC_5156606	Ccdc39	6.800E-06					Ccdc39			
V3SVHSHC_9083243	GABRA6	6.821E-06					GABRA6			
V3SVHSHC_7112483	IFNA10	7.073E-06					IFNA10			
V3SVHSHC_6799346	ABR	7.504E-06					ABR			
V3SVHSHC_7818782	NAP1L1	7.607E-06					NAP1L1			

V3SVHSHC_9996155	LSM5	7.696E-06					LSM5			
V3SVHSHC_5732456	AKTIP	7.754E-06					AKTIP			
V3SVHSHC_7495811	C12orf57	8.011E-06					C12orf57			
V3SVHSHC_9036944	TBC1D4	8.196E-06					TBC1D4			
V3SVHSHC_5406614	IGLL5	8.287E-06					IGLL5			
V3SVHSHC_5159807	SPOCD1	9.107E-06					SPOCD1			
V3SVHSHC_10681268	CHST5	1.010E-05					CHST5			
V3SVHSHC_6538712	ZSCAN32	1.049E-05					ZSCAN32			
V3SVHSHC_7117136	CD160	1.117E-05					CD160			
V3SVHSHC_6492743	LOC101927989	1.123E-05					LOC101927989			
V3SVHSHC_6433178	SLC10A2	1.165E-05					SLC10A2			
V3SVHSHC_7712291	MANSC1	1.199E-05					MANSC1			
V3SVHSHC_6261182	DYNAP	1.209E-05					DYNAP			
V3SVHSHC_6094070	GYG1	1.276E-05					GYG1			
V3SVHSHC_9078887	TBPL2	1.278E-05					TBPL2			
V3SVHSHC_10422416	SLC12A1	1.299E-05					SLC12A1			
V3SVHSHC_7600454	CCDC178	1.323E-05					CCDC178			
V3SVHSHC_5602172	BSND	1.336E-05					BSND			
V3SVHSHC_9629657	FKBP3	1.391E-05					FKBP3			
V3SVHSHC_9211250	CCDC140	1.437E-05					CCDC140			
V3SVHSHC_10085354	IRF2	1.522E-05					IRF2			
V3SVHSHC_6677477	PFAS	1.535E-05					PFAS			
V3SVHSHC_5855480	GABRP	1.573E-05					GABRP			
V3SVHSHC_10388129	MANEA	1.636E-05					MANEA			
V3SVHSHC_5109746	F8A3	1.677E-05					F8A3			
V3SVHSHC_8932235	VWA5B1	1.857E-05					VWA5B1			
V3SVHSHC_6260159	HRASLS5	1.960E-05					HRASLS5			
V3SVHSHC_9006617	LOC101060604	2.021E-05					LOC101060604			
V3SVHSHC_10785779	GPR132	2.061E-05					GPR132			
V3SVHSHC_8259794	GJC1	2.075E-05					GJC1			
V3SVHSHC_8112977	TWF2	2.093E-05					TWF2			
V3SVHSHC_8100767	Llph	2.109E-05					Llph			
V3SVHSHC_9291473	DCTN2	2.325E-05					DCTN2			
V3SVHSHC_10091096	DNM3	2.427E-05					DNM3			
V3SVHSHC_4756910	CDH18	2.438E-05					CDH18			
V3SVHSHC_5480171	USP37	2.438E-05					USP37			
V3SVHSHC_9119708	BSN	2.468E-05					BSN			
V3SVHSHC_4953128	LOC101929725	2.499E-05	2.356E-10		6.488E-07		LOC101929725	LOC101929725		LOC101929725
V3SVHSHC_10590650	Efcab9	2.655E-05					Efcab9			
V3SVHSHC_5126840	ETNK1	2.701E-05					ETNK1			
V3SVHSHC_10328729	TRAPPC9	2.917E-05					TRAPPC9			
V3SVHSHC_5897984	C17orf62	3.019E-05					C17orf62			
V3SVHSHC_9770831	KIAA1755	3.056E-05					KIAA1755			
V3SVHSHC_9500825	OR2M3	3.459E-05					OR2M3			
V3SVHSHC_10374368	ZNF8	3.582E-05		6.330E-06			ZNF8		ZNF8	
V3SVHSHC_9568871	PPARD	3.596E-05					PPARD			
V3SVHSHC_7839110	TULP4	3.600E-05	4.828E-06				TULP4	TULP4		
V3SVHSHC_10774097	HRH3	3.704E-05					HRH3			
V3SVHSHC_10164917	ZDHHC2	3.801E-05					ZDHHC2			
V3SVHSHC_5227259	SNX8	3.875E-05					SNX8			
V3SVHSHC_9845048	PLP1	4.223E-05					PLP1			
V3SVHSHC_5466179	MTG2	4.229E-05					MTG2			
V3SVHSHC_10238210	OR52L1	4.406E-05					OR52L1			

V3SVHSHC_5609102	SMARCD3	4.412E-05					SMARCD3			
V3SVHSHC_8333153	C19orf38	4.439E-05					C19orf38			
V3SVHSHC_5733182	NCR3	4.512E-05					NCR3			
V3SVHSHC_8368859	MAGEA1	4.544E-05					MAGEA1			
V3SVHSHC_9261047	DMD	4.685E-05					DMD			
V3SVHSHC_4662827	SLC2A11	5.118E-05					SLC2A11			
V3SVHSHC_10619096	S100A7	5.322E-05					S100A7			
V3SVHSHC_9939329	FGFBP2	5.334E-05					FGFBP2			
V3SVHSHC_4698104	KCNAB3	5.403E-05					KCNAB3			
V3SVHSHC_6923492	RPL6	5.644E-05					RPL6			
V3SVHSHC_4863896	LOC101060341	5.682E-05					LOC101060341			
V3SVHSHC_10376282	PODXL	5.715E-05					PODXL			
V3SVHSHC_5362295	ZGRF1	6.140E-05					ZGRF1			
V3SVHSHC_9736643	Apobr	6.228E-05					Apobr			
V3SVHSHC_9775517	ZMAT2	6.311E-05					ZMAT2			
V3SVHSHC_6866039	SCML2	6.784E-05					SCML2			
V3SVHSHC_7299263	GDF6	6.904E-05					GDF6			
V3SVHSHC_5230955	PSMD10	6.978E-05					PSMD10			
V3SVHSHC_8836007	RNF182	7.041E-05					RNF182			
V3SVHSHC_10041134	GRIN2B	7.097E-05					GRIN2B			
V3SVHSHC_6641012	TNFAIP8L2-SCNM1	7.599E-05					TNFAIP8L2-SCNM1			
V3SVHSHC_5996852	ZBED3	7.819E-05		9.356E-15			ZBED3		ZBED3	
V3SVHSHC_9693677	DAGLA	7.831E-05					DAGLA			
V3SVHSHC_9209666	C1orf198	8.072E-05					C1orf198			
V3SVHSHC_9663614	FNDC3A	8.200E-05					FNDC3A			
V3SVHSHC_9554681	IL23A	8.208E-05	3.676E-07				IL23A	IL23A		
V3SVHSHC_5578115	OAS1	8.230E-05					OAS1			
V3SVHSHC_7386284	MPO	9.330E-05	1.204E-06				MPO	MPO		
V3SVHSHC_6855809	APMAP	9.605E-05					APMAP			
V3SVHSHC_10763636	C17orf112	9.774E-05					C17orf112			
V3SVHSHC_6261776	SPATA31D1	9.846E-05			4.018E-01		SPATA31D1			
V3SVHSHC_5126213	GRB10	1.013E-04					GRB10			
V3SVHSHC_9609131	PIGC	1.020E-04					PIGC			
V3SVHSHC_5091233	APBB1	1.048E-04					APBB1			
V3SVHSHC_6617285	FIGNL1	1.094E-04					FIGNL1			
V3SVHSHC_10820891	LCE5A	1.167E-04					LCE5A			
V3SVHSHC_9192242	C22orf24	1.226E-04					C22orf24			
V3SVHSHC_9486932	LOC100288966	1.256E-04					LOC100288966			
V3SVHSHC_7091891	C1QTNF4	1.293E-04					C1QTNF4			
V3SVHSHC_8585999	PTGES3L	1.294E-04					PTGES3L			
V3SVHSHC_5044241	SAFB	1.327E-04					SAFB			
V3SVHSHC_10634507	Spns1	1.415E-04					Spns1			
V3SVHSHC_10405751	TIMM17B	1.461E-04					TIMM17B			
V3SVHSHC_7144592	NR1H4	1.497E-04					NR1H4			
V3SVHSHC_5925869	FOXA1	1.501E-04					FOXA1			
V3SVHSHC_7157924	KDR	1.543E-04					KDR			
V3SVHSHC_8914646	TMCO4	1.640E-04					TMCO4			
V3SVHSHC_8668301	MIEN1	1.675E-04					MIEN1			
V3SVHSHC_10382981	CNTNAP4	1.766E-04					CNTNAP4			
V3SVHSHC_7806044	SUN3	1.789E-04					SUN3			
V3SVHSHC_8195279	OR4D9	1.894E-04					OR4D9			
V3SVHSHC_7776245	ADAMTS3	1.906E-04					ADAMTS3			
V3SVHSHC_9482642	NBPF14	1.912E-04					NBPF14			

V3SVHSHC_10582334	LCAT	1.960E-04					LCAT			
V3SVHSHC_9760964	GSG1	1.974E-04					GSG1			
V3SVHSHC_10333151	ATIC	1.978E-04					ATIC			
V3SVHSHC_10232600	FREM1	2.028E-04					FREM1			
V3SVHSHC_5996654	CD68	2.143E-04					CD68			
V3SVHSHC_10060373	TMEM147	2.157E-04					TMEM147			
V3SVHSHC_7306028	FABP5	2.310E-04					FABP5			
V3SVHSHC_6490697	ZBTB7A	2.512E-04					ZBTB7A			
V3SVHSHC_6507659	HIST1H2BA	2.579E-04					HIST1H2BA			
V3SVHSHC_10276787	PLCB4	2.753E-04					PLCB4			
V3SVHSHC_5156375	PPIP5K2	2.851E-04					PPIP5K2			
V3SVHSHC_10375094	TP53I11	2.909E-04					TP53I11			
V3SVHSHC_6396548	TSEN34	2.964E-04					TSEN34			
V3SVHSHC_8950946	SLC22A24	2.979E-04					SLC22A24			
V3SVHSHC_10708163	ADSS	3.007E-04					ADSS			
V3SVHSHC_5069354	Hrct1	3.051E-04					Hrct1			
V3SVHSHC_9433076	REG1A	3.333E-04					REG1A			
V3SVHSHC_9871283	ANAPC2	3.435E-04					ANAPC2			
V3SVHSHC_5496605	DOPEY1	3.481E-04					DOPEY1			
V3SVHSHC_8493401	BRINP2	3.517E-04			2.301E-01		BRINP2			
V3SVHSHC_7055393	COA6	3.585E-04					COA6			
V3SVHSHC_6218909	PSME4	3.694E-04					PSME4			
V3SVHSHC_10451819	IFNA1	3.739E-04					IFNA1			
V3SVHSHC_10528511	GNL3	3.794E-04					GNL3			
V3SVHSHC_5164196	OR5AP2	3.859E-04					OR5AP2			
V3SVHSHC_8456969	GUCA1B	3.863E-04					GUCA1B			
V3SVHSHC_8005100	EMD	3.952E-04					EMD			
V3SVHSHC_4772552	OR2AG2	4.210E-04					OR2AG2			
V3SVHSHC_6115190	MTERFD1	4.272E-04		2.036E-16			MTERFD1		MTERFD1	
V3SVHSHC_9418259	KIAA0513	4.302E-04					KIAA0513			
V3SVHSHC_4634018	OR52L1	4.448E-04					OR52L1			
V3SVHSHC_7599959	CD37	4.536E-04					CD37			
V3SVHSHC_10217717	MXRA8	4.642E-04					MXRA8			
V3SVHSHC_4728827	FST	5.169E-04					FST			
V3SVHSHC_7332758	PPP3CB	5.313E-04					PPP3CB			
V3SVHSHC_9087137	XRCC4	5.996E-04					XRCC4			
V3SVHSHC_9402551	GPATCH2L	6.008E-04		4.958E-11			GPATCH2L		GPATCH2 L	
V3SVHSHC_7872176	F7	6.191E-04			2.945E-01		F7			
V3SVHSHC_6823271	BRINP1	6.291E-04					BRINP1			
V3SVHSHC_10091591	WFDC8	6.482E-04					WFDC8			
V3SVHSHC_6035396	MTERFD2	6.505E-04					MTERFD2			
V3SVHSHC_8443934	ZNF658	6.535E-04					ZNF658			
V3SVHSHC_4801658	CECR2	6.600E-04	9.594E-12				CECR2	CECR2		
V3SVHSHC_9989159	LIPT2	6.624E-04					LIPT2			
V3SVHSHC_9761591	ZSCAN16	6.749E-04					ZSCAN16			
V3SVHSHC_8257682	KIF2B	6.946E-04					KIF2B			
V3SVHSHC_8605601	STRIP2	7.069E-04		3.175E-03	1.680E-02		STRIP2			
V3SVHSHC_6847097	STARD6	7.223E-04					STARD6			
V3SVHSHC_5012495	SRPK3	7.241E-04					SRPK3			
V3SVHSHC_8759480	TOR3A	7.490E-04					TOR3A			
V3SVHSHC_8112152	ADAL	7.677E-04					ADAL			
V3SVHSHC_10636949	RPL39L	7.753E-04					RPL39L			
V3SVHSHC_10260386	TWF1	7.757E-04					TWF1			
V3SVHSHC_7457267	F2	7.869E-04		1.162E-10	4.604E-01		F2		F2	

V3SVHSHC_5156177	BRDT	7.972E-04					BRDT			
V3SVHSHC_10242401	RIN3	8.028E-04								
V3SVHSHC_8209205	CD276	8.113E-04								
V3SVHSHC_9584150	PDGFRB	8.208E-04								
V3SVHSHC_6103607	TMEM30B	8.650E-04			1.501E-01					
V3SVHSHC_7521947	SEC63	8.884E-04								
V3SVHSHC_8811818	NCAM1	9.209E-04								
V3SVHSHC_8678960	MYCBP2	9.318E-04								
V3SVHSHC_6082388	ASB15	9.339E-04								
V3SVHSHC_10717502	OLFML2A	9.380E-04								
V3SVHSHC_6704801	TBC1D10C	1.032E-03	1.446E-06					TBC1D10C		
V3SVHSHC_8275997	PAOX	1.037E-03								
V3SVHSHC_4761596	APOOL	1.061E-03								
V3SVHSHC_9805613	ESX1	1.080E-03								
V3SVHSHC_7053842	DEFB123	1.083E-03								
V3SVHSHC_8767796	KHK	1.120E-03	2.499E-01	2.305E-15	7.973E-02				KHK	
V3SVHSHC_4691867	RPS3	1.138E-03								
V3SVHSHC_5848781	LOC100996634	1.233E-03								
V3SVHSHC_10696085	ZNF304	1.249E-03		4.339E-07					ZNF304	
V3SVHSHC_9770666	LOC100653225	1.281E-03								
V3SVHSHC_4871585	C16ORF3	1.355E-03								
V3SVHSHC_8064467	ZNF34	1.360E-03								
V3SVHSHC_4964447	ZBTB9	1.387E-03								
V3SVHSHC_5624348	KRT27	1.458E-03								
V3SVHSHC_9197654	SHE	1.486E-03								
V3SVHSHC_8320646	DCLK2	1.502E-03								
V3SVHSHC_5578973	LIPA	1.507E-03								
V3SVHSHC_9044435	CD22	1.625E-03								
V3SVHSHC_5417768	CAMK2D	1.642E-03								
V3SVHSHC_6741266	ACSM2A	1.648E-03								
V3SVHSHC_10550324	CD1E	1.657E-03								
V3SVHSHC_7261709	RPL10A	1.665E-03								
V3SVHSHC_8937581	USP9X	1.708E-03								
V3SVHSHC_10021037	OR7D4	1.727E-03								
V3SVHSHC_9060803	MORN4	1.748E-03								
V3SVHSHC_7584713	TMEM181	1.772E-03								
V3SVHSHC_5099285	SLITRK5	1.819E-03								
V3SVHSHC_5452121	SLC2A9	1.835E-03								
V3SVHSHC_9613982	FAM76B	1.839E-03								
V3SVHSHC_10637675	STK11	1.843E-03								
V3SVHSHC_7358663	OVOS	1.976E-03								
V3SVHSHC_7429514	TSPAN12	1.999E-03								
V3SVHSHC_8474855	DERL2	2.006E-03		2.433E-10					DERL2	
V3SVHSHC_7983485	TIMM21	2.105E-03								
V3SVHSHC_7154756	GRPEL2	2.135E-03								
V3SVHSHC_9351236	STOM	2.165E-03								
V3SVHSHC_6770999	OR9Q2	2.173E-03								
V3SVHSHC_9026450	LOC101928282	2.174E-03								
V3SVHSHC_9191846	USP17L15	2.282E-03	1.141E-04					USP17L15		
V3SVHSHC_5603459	IGFL4	2.292E-03								
V3SVHSHC_9460070	NUDCD3	2.318E-03								
V3SVHSHC_10113767	TFAP2B	2.334E-03								
V3SVHSHC_4837001	SLC25A34	2.386E-03								
V3SVHSHC_6228578	ALG14	2.412E-03								

V3SVHSHC_6029753	MST1L	2.439E-03								
V3SVHSHC_6449942	ADAMTS8	2.482E-03								
V3SVHSHC_9342458	OR52A5	2.537E-03								
V3SVHSHC_10836731	RRP15	2.550E-03								
V3SVHSHC_8262368	CCR9	2.610E-03								
V3SVHSHC_10724993	DYM	2.632E-03								
V3SVHSHC_10512044	CCDC121	2.693E-03								
V3SVHSHC_6033086	LYSMD1	2.809E-03								
V3SVHSHC_5179970	CENPO	2.937E-03								
V3SVHSHC_7498979	PAPLN	2.937E-03		2.690E-01						
V3SVHSHC_7955006	TRAIP	2.976E-03			2.812E-01					
V3SVHSHC_6419252	SS18	3.037E-03								
V3SVHSHC_6278837	ZNF322	3.071E-03								
V3SVHSHC_10322360	RNF212	3.123E-03								
V3SVHSHC_4920458	AURKA	3.135E-03		3.853E-01						
V3SVHSHC_7039520	TOMM20	3.179E-03								
V3SVHSHC_9391595	CHKB	3.222E-03								
V3SVHSHC_8212604	AKR1E2	3.284E-03								
V3SVHSHC_9164456	MRPL53	3.320E-03								
V3SVHSHC_5591777	DUSP21	3.508E-03								
V3SVHSHC_6563990	SERPINA6	3.554E-03		2.380E-13	5.139E-06				SERPINA6	SERPINA6
V3SVHSHC_10475018	EEF1G	3.682E-03								
V3SVHSHC_10551710	SLC30A1	3.718E-03								
V3SVHSHC_6352064	ULBP3	3.776E-03								
V3SVHSHC_6148652	GPR20	3.883E-03								
V3SVHSHC_7675133	TUBG2	3.887E-03								
V3SVHSHC_5824196	PCBP4	3.933E-03								
V3SVHSHC_7597946	NXF5	4.090E-03								
V3SVHSHC_6954644	PHLPP1	4.176E-03								
V3SVHSHC_5485847	REL	4.315E-03								
V3SVHSHC_6832874	OR4S2	4.448E-03								
V3SVHSHC_10249628	KCNJ9	4.679E-03								
V3SVHSHC_7120700	PRG2	4.832E-03	4.481E-01							
V3SVHSHC_6367607	SNX22	5.046E-03								
V3SVHSHC_4889537	NCOA5	5.121E-03								
V3SVHSHC_7699685	NCAM1	5.270E-03								
V3SVHSHC_8699552	SST	5.354E-03			3.068E-18					SST
V3SVHSHC_6929036	Phax	5.633E-03								
V3SVHSHC_6291674	MPP6	5.726E-03								
V3SVHSHC_10716446	RNASE12	5.952E-03								
V3SVHSHC_5693582	ZNF132	6.224E-03								
V3SVHSHC_7764794	GUCY1A3	6.256E-03								
V3SVHSHC_7567751	ZNF709	6.488E-03								
V3SVHSHC_10767068	PIBF1	6.746E-03								
V3SVHSHC_6167594	WDR83	6.762E-03								
V3SVHSHC_10353908	THAP10	6.932E-03								
V3SVHSHC_8749943	IRGC	7.117E-03								
V3SVHSHC_6458489	FAM153A	7.164E-03								
V3SVHSHC_7373645	VAMP4	7.171E-03								
V3SVHSHC_4813901	PDGFD	7.461E-03								
V3SVHSHC_4819082	NPR1	7.646E-03								
V3SVHSHC_5252669	LOC100130357	7.884E-03								
V3SVHSHC_10325594	C9ORF40	7.964E-03								
V3SVHSHC_8589860	CDX1	8.150E-03								
V3SVHSHC_6307811	Vsig10	8.171E-03								

V3SVHSHC_9247418	OR4Q3	8.231E-03								
V3SVHSHC_6872078	TFF3	8.659E-03								
V3SVHSHC_4847330	RCC1	8.670E-03	7.947E-02							
V3SVHSHC_5435720	PRKAG3	8.864E-03								
V3SVHSHC_4992530	CSMD3	9.357E-03								
V3SVHSHC_9685262	SCUBE1	9.664E-03								
V3SVHSHC_4748759	THSD7B	9.966E-03		2.889E-22					THSD7B	
V3SVHSHC_10679783	SMR3B	1.006E-02								
V3SVHSHC_10416245	ATF1	1.012E-02								
V3SVHSHC_6872837	FCGR1B	1.019E-02								
V3SVHSHC_8614082	TBPL1	1.027E-02								
V3SVHSHC_5442716	ZNF473	1.035E-02								
V3SVHSHC_8656586	SUV39H1	1.090E-02								
V3SVHSHC_10751855	PRTN3	1.092E-02								
V3SVHSHC_8767070	OR52N4	1.094E-02								
V3SVHSHC_7803305	C1QTNF9	1.100E-02								
V3SVHSHC_9359057	ZXDA	1.139E-02								
V3SVHSHC_8871350	UTRN	1.141E-02			2.608E-04					UTRN
V3SVHSHC_8055458	VPS36	1.173E-02								
V3SVHSHC_7350314	FGFR1	1.189E-02								
V3SVHSHC_8777267	TMEM132B	1.315E-02								
V3SVHSHC_6965831	ETNK1	1.326E-02								
V3SVHSHC_7409747	ODF1	1.344E-02								
V3SVHSHC_7370939	C1orf54	1.361E-02								
V3SVHSHC_5026223	ZBTB14	1.407E-02								
V3SVHSHC_6156473	ZNF711	1.410E-02								
V3SVHSHC_9796802	FGFR1	1.477E-02								
V3SVHSHC_7389749	OR6C75	1.504E-02								
V3SVHSHC_9443372	MYSM1	1.506E-02								
V3SVHSHC_7607879	YTHDF2	1.508E-02								
V3SVHSHC_6214652	C10orf54	1.532E-02								
V3SVHSHC_6585143	LAMP5	1.538E-02		7.521E-36					LAMP5	
V3SVHSHC_7460501	NOS2	1.541E-02								
V3SVHSHC_7558973	GRIN3A	1.574E-02								
V3SVHSHC_5696222	ZNF398	1.592E-02								
V3SVHSHC_9532769	TMEM182	1.606E-02								
V3SVHSHC_7036979	PMCH	1.634E-02								
V3SVHSHC_8130302	CT45A6	1.689E-02								
V3SVHSHC_5210231	PTCHD3	1.692E-02								
V3SVHSHC_10572599	FAM195A	1.708E-02								
V3SVHSHC_8600585	TMPRSS6	1.785E-02	4.777E-17					TMPRSS6		
V3SVHSHC_4967747	PLXNA4	1.816E-02								
V3SVHSHC_8408162	TMEM101	1.820E-02								
V3SVHSHC_6372260	FBXW4	1.855E-02								
V3SVHSHC_9189305	C20orf202	1.855E-02								
V3SVHSHC_6708101	PROSER2	1.858E-02								
V3SVHSHC_6894188	HSPA12B	1.895E-02								
V3SVHSHC_9832904	C1QB	1.933E-02								
V3SVHSHC_5325830	SERPINB1	1.993E-02								
V3SVHSHC_5662991	ZFP82	2.010E-02								
V3SVHSHC_5792417	ABR	2.029E-02								
V3SVHSHC_6867359	GSTA2	2.174E-02								
V3SVHSHC_5882672	SLC23A2	2.244E-02								
V3SVHSHC_6521453	SDAD1	2.279E-02								
V3SVHSHC_7372754	CCDC37	2.313E-02								

V3SVHSHC_4824461	MARVELD2	2.383E-02								
V3SVHSHC_6895739	RNASE7	2.419E-02								
V3SVHSHC_6869108	C16orf47	2.514E-02								
V3SVHSHC_10087697	DDX46	2.530E-02								
V3SVHSHC_5460107	RESP18	2.608E-02								
V3SVHSHC_7355231	CPB1	2.631E-02								
V3SVHSHC_10829405	FAM43B	2.758E-02								
V3SVHSHC_8508746	OR14J1	2.769E-02								
V3SVHSHC_9010874	FEZ2	2.892E-02								
V3SVHSHC_10038164	DCDC2	2.903E-02								
V3SVHSHC_8032985	OCIAD1	3.043E-02								
V3SVHSHC_7512245	SPRYD4	3.117E-02								
V3SVHSHC_6417371	INSM2	3.157E-02								
V3SVHSHC_7600355	ARHGAP15	3.159E-02								
V3SVHSHC_4952798	OR5P2	3.281E-02								
V3SVHSHC_9498317	TG	3.523E-02								
V3SVHSHC_10025756	ZSWIM7	3.695E-02								
V3SVHSHC_9978368	SLC25A25	3.747E-02								
V3SVHSHC_6368267	SLC35G1	3.867E-02								
V3SVHSHC_9319589	XAGE1B	4.139E-02								
V3SVHSHC_7728494	ACOXL	4.177E-02								
V3SVHSHC_8872967	NCOA3	4.180E-02								
V3SVHSHC_5028995	LAMP5	4.238E-02								
V3SVHSHC_8194685	MCC	4.460E-02								
V3SVHSHC_8555705	PSG11	4.598E-02								
V3SVHSHC_6056054	TMEM216	4.683E-02	4.302E-04					TMEM216		
V3SVHSHC_5696783	ZC3H3	4.786E-02								
V3SVHSHC_4939301	ZIC5	4.802E-02								
V3SVHSHC_9982130	PIBF1	4.809E-02								
V3SVHSHC_4773245	CFB	5.269E-02								
V3SVHSHC_6902174	PF4V1	5.297E-02								
V3SVHSHC_5141459	NUAK1	5.323E-02								
V3SVHSHC_9635762	VMO1	5.379E-02								
V3SVHSHC_10022489	TMEM218	5.453E-02								
V3SVHSHC_5517131	SYCE3	5.466E-02								
V3SVHSHC_4912439	DMC1	5.530E-02								
V3SVHSHC_5614316	TSHZ2	5.591E-02								
V3SVHSHC_5071763	PPM1D	5.592E-02								
V3SVHSHC_4793738	TRIM54	5.610E-02	3.535E-14					TRIM54		
V3SVHSHC_8022953	MFAP3L	5.737E-02								
V3SVHSHC_5544356	TMEM81	5.826E-02								
V3SVHSHC_10247417	ZNF561	5.885E-02								
V3SVHSHC_10676813	NSUN6	5.927E-02								
V3SVHSHC_5526404	STK3	6.069E-02								
V3SVHSHC_6583196	RBM44	6.268E-02								
V3SVHSHC_9575999	USP6	6.508E-02			2.938E-08					USP6
V3SVHSHC_7877489	CLCN1	6.617E-02								
V3SVHSHC_9865211	SLC6A18	6.623E-02	4.547E-01		3.909E-01					
V3SVHSHC_5386814	TOMM70A	6.761E-02								
V3SVHSHC_6777599	HIST1H4F	7.016E-02								
V3SVHSHC_9445418	C11orf94	7.018E-02			3.567E-01					
V3SVHSHC_9257582	ADCY3	7.197E-02								
V3SVHSHC_7525577	NDUFA12	7.281E-02								
V3SVHSHC_5302202	SIT1	7.336E-02		4.957E-23					SIT1	
V3SVHSHC_10127792	CLEC2L	7.376E-02								

V3SVHSHC_6740936	CD33	7.816E-02								
V3SVHSHC_8082419	ALPK3	7.834E-02								
V3SVHSHC_9639557	FOLR1	7.854E-02								
V3SVHSHC_10567352	AIG1	8.093E-02		3.781E-24					AIG1	
V3SVHSHC_8940287	PPIAL4C	8.096E-02								
V3SVHSHC_7578146	DNAH7	8.218E-02								
V3SVHSHC_10394366	ANP32A	8.425E-02								
V3SVHSHC_9522440	PSEN2	8.464E-02								
V3SVHSHC_6387605	PPAN	8.688E-02		1.637E-01						
V3SVHSHC_10207190	HSPB2	8.726E-02								
V3SVHSHC_4685894	OSGIN2	9.232E-02		7.185E-03						
V3SVHSHC_8060177	VPS16	9.360E-02								
V3SVHSHC_9314474	FFAR1	9.381E-02								
V3SVHSHC_8961803	CCDC59	9.447E-02								
V3SVHSHC_7012922	CTNNA1	9.522E-02								
V3SVHSHC_9900554	DNAJB13	9.570E-02								
V3SVHSHC_4762718	MTX2	9.695E-02								
V3SVHSHC_8072915	FOXRED2	9.759E-02								
V3SVHSHC_4935506	KHDRBS2	9.812E-02								
V3SVHSHC_9317576	ABHD17C	9.916E-02								
V3SVHSHC_6145451	B9D2	9.951E-02								
V3SVHSHC_4732127	CDK1	1.032E-01								
V3SVHSHC_5440934	SCEL	1.033E-01								
V3SVHSHC_6050312	DYNC1H1	1.036E-01		1.465E-01	2.915E-01					
V3SVHSHC_7246463	Gramd1b	1.043E-01								
V3SVHSHC_8186402	CEP250	1.049E-01								
V3SVHSHC_7931972	FAM65B	1.052E-01								
V3SVHSHC_4841555	TRAF1	1.056E-01								
V3SVHSHC_7144130	ART5	1.082E-01								
V3SVHSHC_10117232	RBM47	1.110E-01								
V3SVHSHC_7401926	SEC24B	1.116E-01								
V3SVHSHC_5154857	DHX57	1.140E-01								
V3SVHSHC_9644342	GAGE7	1.150E-01		9.931E-07					GAGE7	
V3SVHSHC_9132776	APOL5	1.171E-01								
V3SVHSHC_5862443	CARD14	1.218E-01								
V3SVHSHC_9333482	CDK1	1.228E-01								
V3SVHSHC_8570984	IL18	1.238E-01								
V3SVHSHC_10140563	NUP62CL	1.283E-01	2.673E-13		4.734E-01			NUP62CL		
V3SVHSHC_8670248	YAP1	1.308E-01		2.086E-01						
V3SVHSHC_7713380	PPP2R1A	1.309E-01								
V3SVHSHC_9360311	OR2L3	1.339E-01								
V3SVHSHC_6685859	GNAT1	1.342E-01								
V3SVHSHC_5739584	Ntn5	1.345E-01								
V3SVHSHC_5445158	OLAH	1.363E-01								
V3SVHSHC_8819408	CAPNS1	1.373E-01								
V3SVHSHC_8034701	SPRR2G	1.381E-01								
V3SVHSHC_8526533	AUNIP	1.386E-01								
V3SVHSHC_8143205	SMS	1.399E-01								
V3SVHSHC_7849901	ICA1L	1.435E-01								
V3SVHSHC_7080704	C2orf44	1.488E-01								
V3SVHSHC_6770570	ZBTB20	1.508E-01								
V3SVHSHC_5388464	TNFRSF17	1.560E-01								
V3SVHSHC_7750703	ACTB	1.598E-01			4.459E-01					
V3SVHSHC_8670875	PSMD6	1.599E-01								
V3SVHSHC_4803572	GPR65	1.606E-01			7.456E-06					GPR65

V3SVHSHC_5615075	ZNF703	1.635E-01								
V3SVHSHC_7182113	NLRP9	1.635E-01								
V3SVHSHC_9483038	Sycp2l	1.671E-01								
V3SVHSHC_7530527	GRXCR2	1.679E-01								
V3SVHSHC_7395788	IFNL3	1.727E-01								
V3SVHSHC_5404634	TOMM34	1.743E-01								
V3SVHSHC_8327609	MFSD6	1.748E-01								
V3SVHSHC_5946725	COPB2	1.752E-01								
V3SVHSHC_9873758	FUT5	1.770E-01								
V3SVHSHC_6482579	SCRIB	1.788E-01								
V3SVHSHC_8044436	KIT	1.811E-01								
V3SVHSHC_9543758	KLRF1	1.835E-01		1.919E-54					KLRF1	
V3SVHSHC_6750341	KCNB1	1.844E-01								
V3SVHSHC_10322690	APOB	1.845E-01								
V3SVHSHC_6771989	PFDN5	1.865E-01								
V3SVHSHC_6422123	C12orf60	1.916E-01								
V3SVHSHC_8213495	TMEM192	1.919E-01								
V3SVHSHC_9824522	NUDT5	2.019E-01								
V3SVHSHC_6772286	LAMB1	2.046E-01								
V3SVHSHC_8541020	CHST14	2.172E-01								
V3SVHSHC_9745487	CYP24A1	2.181E-01								
V3SVHSHC_8113538	CASC1	2.192E-01								
V3SVHSHC_8811422	LOC101929849	2.210E-01								
V3SVHSHC_9088853	DIRAS2	2.249E-01			1.254E-33					DIRAS2
V3SVHSHC_5624777	POLR1A	2.307E-01	4.617E-01		2.303E-01					
V3SVHSHC_7414004	TGFBR3L	2.322E-01								
V3SVHSHC_9315662	SRMS	2.386E-01								
V3SVHSHC_7542143	BLOC1S1	2.462E-01								
V3SVHSHC_7343912	AURKA	2.488E-01								
V3SVHSHC_8777564	HPS4	2.499E-01								
V3SVHSHC_10021103	EPHX1	2.499E-01								
V3SVHSHC_5751662	EPN2	2.581E-01								
V3SVHSHC_7233098	DGCR2	2.595E-01								
V3SVHSHC_9143534	GPATCH8	2.604E-01			3.886E-01					
V3SVHSHC_4833734	HSD17B11	2.662E-01								
V3SVHSHC_7873298	PPIB	2.695E-01			2.377E-01					
V3SVHSHC_8632661	C19orf80	2.733E-01								
V3SVHSHC_7594646	ACTB	2.757E-01								
V3SVHSHC_9694898	RNF123	2.757E-01	8.542E-06					RNF123		
V3SVHSHC_8117267	OST4	2.774E-01								
V3SVHSHC_5416217	CUL4A	2.778E-01	5.554E-02	2.288E-02	2.925E-15					CUL4A
V3SVHSHC_9448190	APLF	2.824E-01								
V3SVHSHC_8265404	PKD2L1	2.835E-01								
V3SVHSHC_5837759	Apobr	2.843E-01								
V3SVHSHC_7795121	SLC10A3	2.866E-01								
V3SVHSHC_5776247	ZFAND4	2.873E-01								
V3SVHSHC_7424432	TOMM34	2.891E-01	4.541E-01	1.201E-01	3.206E-01					
V3SVHSHC_10142180	DAK	2.932E-01								
V3SVHSHC_9800729	RPN2	2.963E-01								
V3SVHSHC_9853529	SBSPON	2.978E-01								
V3SVHSHC_6304973	C15orf56	3.001E-01		1.437E-06					C15orf56	
V3SVHSHC_7690181	PTPRT	3.043E-01								
V3SVHSHC_9811355	ZNF396	3.090E-01								
V3SVHSHC_9962264	TCERG1L	3.150E-01								
V3SVHSHC_8439281	PTPRN	3.227E-01								

V3SVHSHC_7803008	DTX3	3.232E-01								
V3SVHSHC_5529209	H3F3B	3.236E-01			3.954E-01					
V3SVHSHC_9368330	IL1RAPL2	3.240E-01								
V3SVHSHC_10068854	NOTUM	3.351E-01								
V3SVHSHC_6790139	ACACB	3.352E-01								
V3SVHSHC_5498288	HSF1	3.398E-01								
V3SVHSHC_10635332	SERTAD3	3.432E-01								
V3SVHSHC_7112582	BAG4	3.435E-01								
V3SVHSHC_8860130	OCM	3.454E-01								
V3SVHSHC_10134722	SLITRK6	3.470E-01								
V3SVHSHC_8219435	NOVA2	3.495E-01								
V3SVHSHC_5975534	DIRAS1	3.556E-01								
V3SVHSHC_5009063	SUPT20HL2	3.583E-01								
V3SVHSHC_8622926	C3orf67	3.589E-01	2.892E-01							
V3SVHSHC_7203959	LOC101929051	3.652E-01								
V3SVHSHC_7420076	TMEM55A	3.685E-01								
V3SVHSHC_6190958	SNX31	3.693E-01								
V3SVHSHC_9617843	PRKCG	3.697E-01								
V3SVHSHC_6443276	GPBP1L1	3.725E-01								
V3SVHSHC_7534190	PLK1	3.735E-01								
V3SVHSHC_8815778	SLC29A2	3.855E-01								
V3SVHSHC_7874453	TTLL12	3.892E-01								
V3SVHSHC_6601973	LOC101060301	3.897E-01								
V3SVHSHC_4868846	DSC1	3.903E-01								
V3SVHSHC_7902833	NIP7	3.971E-01			2.943E-01					
V3SVHSHC_7101692	CDC42EP3	4.028E-01								
V3SVHSHC_10143962	WWTR1	4.042E-01								
V3SVHSHC_6437105	SLC35E2B	4.090E-01			2.836E-08					SLC35E2B
V3SVHSHC_6942368	TMBIM6	4.115E-01								
V3SVHSHC_9362423	UBB	4.126E-01								
V3SVHSHC_10693412	ZNF19	4.143E-01								
V3SVHSHC_6641441	TNFRSF17	4.172E-01								
V3SVHSHC_6887126	ACTB	4.229E-01								
V3SVHSHC_4662893	ACVR1C	4.249E-01								
V3SVHSHC_5138489	SSPN	4.256E-01								
V3SVHSHC_7840199	UTS2B	4.310E-01								
V3SVHSHC_7966325	PDF	4.355E-01								
V3SVHSHC_7543199	RHBDD1	4.356E-01								
V3SVHSHC_4676324	DENR	4.407E-01								
V3SVHSHC_8953190	ZFP28	4.423E-01								
V3SVHSHC_6356222	HSBP1L1	4.438E-01			4.010E-01					
V3SVHSHC_7700543	GRHL2	4.466E-01								
V3SVHSHC_6237092	DCAF15	4.489E-01								
V3SVHSHC_10133534	FCGRT	4.611E-01								
V3SVHSHC_5435951	TBC1D22A	4.628E-01								
V3SVHSHC_5605439	FGFR1	4.659E-01		1.133E-01						
V3SVHSHC_8335562	PABPC4	4.705E-01		1.374E-08					PABPC4	
V3SVHSHC_9742847	BAI2	4.712E-01			5.282E-16					BAI2
V3SVHSHC_8058164	TPP1	4.734E-01								
V3SVHSHC_8420999	TRIM16L	4.756E-01								
V3SVHSHC_10092581	FAM163A	4.785E-01								
V3SVHSHC_10202108	PNLIPRP1	4.826E-01		1.599E-01						
V3SVHSHC_7367342	SPTBN2	4.926E-01								
V3SVHSHC_8494358	CALN1	4.969E-01								
V3SVHSHC_8746676	IL18RAP	4.980E-01								

V3SVHSHC_4744931	SAP130	4.994E-01								
V3SVHSHC_9258506	CD8B		3.666E-42					CD8B		
V3SVHSHC_5619629	SET		2.128E-36					SET		
V3SVHSHC_7442285	COX6B1		5.857E-36					COX6B1		
V3SVHSHC_9148418	GABARAPL1		6.733E-36					GABARAPL1		
V3SVHSHC_10183727	TJP2		5.144E-35					TJP2		
V3SVHSHC_6484658	TMEM170A		2.310E-33					TMEM170A		
V3SVHSHC_8377835	C1QL4		6.105E-32					C1QL4		
V3SVHSHC_7726448	HIST1H1T		1.866E-29					HIST1H1T		
V3SVHSHC_10345163	SMIM23		3.728E-29					SMIM23		
V3SVHSHC_8197952	Chpf2		2.674E-27					Chpf2		
V3SVHSHC_5177825	PRODH		3.686E-27					PRODH		
V3SVHSHC_6457796	GALNT8		1.427E-26					GALNT8		
V3SVHSHC_7710938	GOLPH3		2.424E-26					GOLPH3		
V3SVHSHC_10142213	FAM69C		7.109E-26					FAM69C		
V3SVHSHC_5006291	SIRT5		8.241E-26					SIRT5		
V3SVHSHC_10712684	ATXN2L		1.102E-25					ATXN2L		
V3SVHSHC_10310810	CLUL1		1.472E-25					CLUL1		
V3SVHSHC_8798552	IFNL3		3.392E-25					IFNL3		
V3SVHSHC_6929927	PTRH2		8.596E-25					PTRH2		
V3SVHSHC_10543955	JADE3 (PHF16)		8.771E-25					JADE3 (PHF16)		
V3SVHSHC_7754696	SIGLEC8		1.217E-24					SIGLEC8		
V3SVHSHC_5588708	ABCA13		1.415E-24					ABCA13		
V3SVHSHC_7615469	SNAP25		3.678E-24					SNAP25		
V3SVHSHC_9120962	TMPRSS11F		4.086E-24		7.203E-16			TMPRSS11F		TMPRSS11F
V3SVHSHC_4655072	GRPEL2		1.945E-23					GRPEL2		
V3SVHSHC_10419809	EGFR		3.271E-23					EGFR		
V3SVHSHC_9749810	GM2A		4.526E-23					GM2A		
V3SVHSHC_10825412	ANGPTL4		4.907E-23					ANGPTL4		
V3SVHSHC_5900228	PNPLA5		8.111E-23					PNPLA5		
V3SVHSHC_10297181	Greb1l		1.894E-22					Greb1l		
V3SVHSHC_5757800	PKHD1		2.060E-22					PKHD1		
V3SVHSHC_5136311	TAF1L		3.053E-22					TAF1L		
V3SVHSHC_7378034	ISX		3.999E-22					ISX		
V3SVHSHC_6924218	GSTM3		7.221E-22					GSTM3		
V3SVHSHC_6785552	RNF26		3.363E-21					RNF26		
V3SVHSHC_7754663	FAM127A		4.270E-21					FAM127A		
V3SVHSHC_8921411	OGG1		4.899E-21					OGG1		
V3SVHSHC_8211416	PLIN1		5.020E-21					PLIN1		
V3SVHSHC_5786444	LDB1		6.608E-21					LDB1		
V3SVHSHC_8513366	TRIM39-RPP21		7.407E-21					TRIM39-RPP21		
V3SVHSHC_5100935	RHPN2		7.527E-21					RHPN2		
V3SVHSHC_9284807	KLHL13		7.531E-21					KLHL13		
V3SVHSHC_8837591	OR5K4		9.238E-21					OR5K4		
V3SVHSHC_10633286	SUB1		2.164E-20					SUB1		
V3SVHSHC_8363777	CLEC6A		2.468E-20					CLEC6A		
V3SVHSHC_10017374	DENND3		3.218E-20					DENND3		
V3SVHSHC_5037344	SC5D		3.300E-20					SC5D		
V3SVHSHC_4808027	SLC34A1		3.460E-20					SLC34A1		
V3SVHSHC_9741362	GSPT1		3.609E-20		1.394E-05			GSPT1		GSPT1

V3SVHSHC_6438656	USP20		4.665E-20					USP20		
V3SVHSHC_10689848	PTDSS1		9.862E-20					PTDSS1		
V3SVHSHC_5433641	TAF9B		1.016E-19					TAF9B		
V3SVHSHC_5101232	ROM1		1.245E-19					ROM1		
V3SVHSHC_7216796	CELSR2		1.437E-19					CELSR2		
V3SVHSHC_6392423	AFP		1.478E-19					AFP		
V3SVHSHC_7463075	TGIF2LY		2.214E-19					TGIF2LY		
V3SVHSHC_10478351	CHIC2		2.247E-19					CHIC2		
V3SVHSHC_9348662	SLC12A4		2.656E-19					SLC12A4		
V3SVHSHC_7256231	KLF12		2.673E-19					KLF12		
V3SVHSHC_5884784	GABBR1		3.624E-19					GABBR1		
V3SVHSHC_10758422	CCDC17		3.650E-19					CCDC17		
V3SVHSHC_4830368	STXBP6		3.809E-19					STXBP6		
V3SVHSHC_8541449	IL5		3.944E-19					IL5		
V3SVHSHC_6734831	GNG13		4.006E-19					GNG13		
V3SVHSHC_6870494	VCAM1		4.905E-19					VCAM1		
V3SVHSHC_6732653	HTN1		5.121E-19					HTN1		
V3SVHSHC_8599199	HIBCH		5.223E-19					HIBCH		
V3SVHSHC_8833664	ATP13A4		5.601E-19					ATP13A4		
V3SVHSHC_10203461	C2orf61		5.635E-19					C2orf61		
V3SVHSHC_8686220	ELAVL4		5.883E-19					ELAVL4		
V3SVHSHC_8270024	SYK		5.984E-19					SYK		
V3SVHSHC_5392787	KRTAP4-11		7.011E-19					KRTAP4-11		
V3SVHSHC_9947744	MRPL48		8.339E-19					MRPL48		
V3SVHSHC_9054467	SHROOM3		8.365E-19					SHROOM3		
V3SVHSHC_5602832	Prss53		8.460E-19					Prss53		
V3SVHSHC_8881250	SPINK9		8.954E-19					SPINK9		
V3SVHSHC_5435192	FCRL4		9.171E-19					FCRL4		
V3SVHSHC_5242868	EBAG9		9.399E-19					EBAG9		
V3SVHSHC_6975137	CCND2		9.883E-19					CCND2		
V3SVHSHC_10749974	CEP164		1.001E-18					CEP164		
V3SVHSHC_8985167	CXCL6		1.038E-18					CXCL6		
V3SVHSHC_6806771	Sycp2l		1.073E-18					Sycp2l		
V3SVHSHC_9449444	C3AR1		1.225E-18					C3AR1		
V3SVHSHC_10609526	IGF2BP3		1.250E-18					IGF2BP3		
V3SVHSHC_6906431	DNAJB7		1.250E-18					DNAJB7		
V3SVHSHC_7281047	ULBP1		1.258E-18					ULBP1		
V3SVHSHC_10854254	C10orf62		1.377E-18					C10orf62		
V3SVHSHC_5827463	C17orf98		1.419E-18					C17orf98		
V3SVHSHC_7595240	BGLAP		1.493E-18					BGLAP		
V3SVHSHC_5104400	POM121L2		1.617E-18					POM121L2		
V3SVHSHC_10173860	VAMP3		1.712E-18					VAMP3		
V3SVHSHC_10356977	DDX47		1.714E-18					DDX47		
V3SVHSHC_4879505	STK17A		1.761E-18					STK17A		
V3SVHSHC_6204554	TEX28		2.014E-18					TEX28		
V3SVHSHC_10608932	WDFY1		2.029E-18					WDFY1		
V3SVHSHC_4692560	MRPL10		2.133E-18					MRPL10		
V3SVHSHC_7765883	ADAMTS19		2.348E-18					ADAMTS19		
V3SVHSHC_8091131	SLC39A2		2.601E-18					SLC39A2		
V3SVHSHC_10668629	Dnajc30		2.908E-18					Dnajc30		
V3SVHSHC_6047540	REEP4		3.183E-18					REEP4		
V3SVHSHC_8583887	OR9A4		3.499E-18					OR9A4		

V3SVHSHC_6895805	UBL4B		3.860E-18					UBL4B		
V3SVHSHC_6434003	SHOC2		4.006E-18					SHOC2		
V3SVHSHC_9839570	BTNL2		5.137E-18					BTNL2		
V3SVHSHC_7667807	PRAME		5.195E-18	8.231E-19				PRAME	PRAME	
V3SVHSHC_8542010	GPR55		5.695E-18					GPR55		
V3SVHSHC_5460602	ARHGEF7		5.986E-18					ARHGEF7		
V3SVHSHC_9593720	KCNA6		5.990E-18					KCNA6		
V3SVHSHC_5741564	TNFAIP1		6.518E-18					TNFAIP1		
V3SVHSHC_8673911	EREG		7.222E-18					EREG		
V3SVHSHC_4659230	NECAB2		7.265E-18					NECAB2		
V3SVHSHC_6965501	CAPN6		7.784E-18					CAPN6		
V3SVHSHC_8340347	AJAP1		9.312E-18					AJAP1		
V3SVHSHC_7826141	THNSL1		9.319E-18					THNSL1		
V3SVHSHC_8863562	POLR2K		9.394E-18					POLR2K		
V3SVHSHC_9816767	UBE2D2		1.256E-17					UBE2D2		
V3SVHSHC_10393805	NFATC1		1.331E-17					NFATC1		
V3SVHSHC_8275667	SCFD2		1.406E-17					SCFD2		
V3SVHSHC_7830827	APOBEC3F		1.433E-17					APOBEC3F		
V3SVHSHC_7260290	SOCS7		1.457E-17					SOCS7		
V3SVHSHC_10601012	FEZF2		1.476E-17					FEZF2		
V3SVHSHC_5195315	GRB7		1.582E-17	5.394E-05				GRB7	GRB7	
V3SVHSHC_6375065	GJA5		1.620E-17					GJA5		
V3SVHSHC_7049453	CTTNBP2NL		1.696E-17					CTTNBP2NL		
V3SVHSHC_9014471	C10ORF53		1.891E-17					C10ORF53		
V3SVHSHC_8437004	BLZF1		1.950E-17					BLZF1		
V3SVHSHC_10828316	CRY1		2.160E-17					CRY1		
V3SVHSHC_10647872	PLEKHF2		2.302E-17					PLEKHF2		
V3SVHSHC_8565011	SDPR		2.476E-17					SDPR		
V3SVHSHC_9184487	SUSD1		2.561E-17					SUSD1		
V3SVHSHC_7568477	KRTAP9-1		2.646E-17					KRTAP9-1		
V3SVHSHC_7771922	PTP4A2		2.679E-17					PTP4A2		
V3SVHSHC_7262336	AMIGO2		3.092E-17					AMIGO2		
V3SVHSHC_5656424	DNASE1L1		3.428E-17					DNASE1L1		
V3SVHSHC_8980448	RBM45		3.438E-17					RBM45		
V3SVHSHC_8801522	KDELC1		3.475E-17					KDELC1		
V3SVHSHC_8934083	PKD1		3.694E-17					PKD1		
V3SVHSHC_10287446	KIFAP3		3.839E-17					KIFAP3		
V3SVHSHC_7702259	FOXI1		4.053E-17					FOXI1		
V3SVHSHC_7089581	OR4C6		4.136E-17					OR4C6		
V3SVHSHC_7842311	MTMR9		4.562E-17					MTMR9		
V3SVHSHC_6826208	ZNF816-ZNF321P		4.571E-17					ZNF816-ZNF321P		
V3SVHSHC_7111460	LOC101059976		4.595E-17					LOC101059976		
V3SVHSHC_6264383	GPR20		4.914E-17					GPR20		
V3SVHSHC_8226827	STARD13		5.048E-17					STARD13		
V3SVHSHC_5385098	GPR137		5.408E-17					GPR137		
V3SVHSHC_7189241	TOP1		5.677E-17					TOP1		
V3SVHSHC_7391828	HOXD8		6.033E-17					HOXD8		
V3SVHSHC_5341406	ANKRD18A		6.048E-17					ANKRD18A		
V3SVHSHC_5489873	FAM83D		6.105E-17					FAM83D		

V3SVHSHC_6516503	OR6B2		6.370E-17					OR6B2		
V3SVHSHC_7309526	CGB1		6.534E-17					CGB1		
V3SVHSHC_7738922	CDH6		6.546E-17					CDH6		
V3SVHSHC_10845707	PCGF2		6.963E-17					PCGF2		
V3SVHSHC_8681666	AIM2		7.067E-17					AIM2		
V3SVHSHC_6923261	NUP37		7.152E-17					NUP37		
V3SVHSHC_8511122	SLC43A2		7.699E-17					SLC43A2		
V3SVHSHC_9521945	SLC2A14		8.138E-17					SLC2A14		
V3SVHSHC_7325927	Mst4		8.215E-17	5.813E-06				Mst4	Mst4	
V3SVHSHC_8987378	LCMT2		8.378E-17					LCMT2		
V3SVHSHC_10011137	OR4E2		8.979E-17					OR4E2		
V3SVHSHC_9934874	CITED2		9.147E-17					CITED2		
V3SVHSHC_10568507	FKBP15		9.309E-17		4.428E-21			FKBP15		FKBP15
V3SVHSHC_4772453	MS4A12		9.384E-17					MS4A12		
V3SVHSHC_6470567	DEFB131		9.721E-17					DEFB131		
V3SVHSHC_4905212	LOC101930085		9.830E-17					LOC10193 0085		
V3SVHSHC_10599494	PDP1		9.993E-17	3.726E-03				PDP1		
V3SVHSHC_5364473	SIK3		1.101E-16					SIK3		
V3SVHSHC_6226697	SP110		1.169E-16					SP110		
V3SVHSHC_8529866	TMEM165		1.257E-16	2.931E-13				TMEM165	TMEM165	
V3SVHSHC_6293324	ZNF665		1.275E-16					ZNF665		
V3SVHSHC_10034303	TRAM1		1.398E-16					TRAM1		
V3SVHSHC_9449279	LOXL1		1.451E-16	2.568E-10				LOXL1	LOXL1	
V3SVHSHC_8652956	SLC12A7		1.494E-16					SLC12A7		
V3SVHSHC_6095357	TRAFD1		1.494E-16					TRAFD1		
V3SVHSHC_6707738	NGRN		1.504E-16					NGRN		
V3SVHSHC_9114692	CCNC		1.542E-16					CCNC		
V3SVHSHC_6078494	PRIMA1		1.578E-16					PRIMA1		
V3SVHSHC_9627677	SH3PXD2B		1.620E-16					SH3PXD2 B		
V3SVHSHC_7859669	SFN		1.688E-16					SFN		
V3SVHSHC_8440700	ZNF500		1.880E-16					ZNF500		
V3SVHSHC_8482841	C9orf169		2.000E-16	2.881E-17				C9orf169	C9orf169	
V3SVHSHC_7229435	EPT1		2.099E-16					EPT1		
V3SVHSHC_10054202	GLI3		2.166E-16					GLI3		
V3SVHSHC_10661534	FAM65A		2.242E-16					FAM65A		
V3SVHSHC_10197224	PKN1		2.332E-16					PKN1		
V3SVHSHC_8451260	GCA		2.557E-16					GCA		
V3SVHSHC_8852705	SIN3A		2.559E-16					SIN3A		
V3SVHSHC_9980744	MTBP		2.570E-16					MTBP		
V3SVHSHC_10028693	ASMTL		2.631E-16					ASMTL		
V3SVHSHC_7826405	ZNF433		2.948E-16					ZNF433		
V3SVHSHC_6127928	TRPT1		3.113E-16					TRPT1		
V3SVHSHC_7245308	COQ7		3.180E-16					COQ7		
V3SVHSHC_6177032	HSPB8		3.245E-16					HSPB8		
V3SVHSHC_5608046	Lrit1		3.264E-16					Lrit1		
V3SVHSHC_5353055	CRYBA4		3.325E-16					CRYBA4		
V3SVHSHC_7399517	C15orf57		3.367E-16					C15orf57		
V3SVHSHC_9728690	SMAD3		3.675E-16					SMAD3		
V3SVHSHC_5803142	Ly6g6f		3.868E-16					Ly6g6f		
V3SVHSHC_6933656	CD79B		4.078E-16					CD79B		
V3SVHSHC_10220060	Tbck		4.144E-16					Tbck		
V3SVHSHC_8543891	SYCE1L		4.259E-16					SYCE1L		
V3SVHSHC_6732521	NFKB2		4.561E-16					NFKB2		

V3SVHSHC_6402488	FRMD8		4.627E-16					FRMD8		
V3SVHSHC_6455948	LHCGR		4.802E-16					LHCGR		
V3SVHSHC_4931249	AKAP3		4.944E-16					AKAP3		
V3SVHSHC_4849013	PTP4A1		5.151E-16					PTP4A1		
V3SVHSHC_9942035	SOCS1		5.228E-16					SOCS1		
V3SVHSHC_8322362	DHTKD1		5.819E-16					DHTKD1		
V3SVHSHC_7642232	LAMB4		5.842E-16					LAMB4		
V3SVHSHC_7477100	PAM		6.021E-16					PAM		
V3SVHSHC_6728693	Syce2		6.159E-16					Syce2		
V3SVHSHC_7714568	TMEM56		6.188E-16					TMEM56		
V3SVHSHC_9345593	PHF2		6.756E-16					PHF2		
V3SVHSHC_8930948	ST20-MTHFS		6.810E-16					ST20-MTHFS		
V3SVHSHC_7742651	C15orf32		7.123E-16					C15orf32		
V3SVHSHC_6723644	HSD17B4		7.547E-16					HSD17B4		
V3SVHSHC_6723908	CMTM6		7.778E-16					CMTM6		
V3SVHSHC_4647779	GSPT2		8.061E-16		4.201E-01			GSPT2		
V3SVHSHC_6417140	C6ORF89		8.419E-16					C6ORF89		
V3SVHSHC_4936595	PVRL2		8.546E-16					PVRL2		
V3SVHSHC_8811092	Tctex1d2		9.044E-16					Tctex1d2		
V3SVHSHC_7496702	RABGGTB		9.049E-16					RABGGTB		
V3SVHSHC_4909436	JPH3		9.225E-16					JPH3		
V3SVHSHC_10245866	CACNB3		9.307E-16					CACNB3		
V3SVHSHC_9904547	PALM		9.890E-16					PALM		
V3SVHSHC_9605930	CYP7B1		1.017E-15					CYP7B1		
V3SVHSHC_10783700	DHX40		1.023E-15					DHX40		
V3SVHSHC_5624909	KCTD10		1.070E-15					KCTD10		
V3SVHSHC_7543067	VIPR2		1.089E-15					VIPR2		
V3SVHSHC_7168781	GPR133		1.173E-15					GPR133		
V3SVHSHC_10792280	CLEC6A		1.193E-15					CLEC6A		
V3SVHSHC_9542603	SIX6		1.226E-15					SIX6		
V3SVHSHC_9951407	VWA8		1.253E-15					VWA8		
V3SVHSHC_7844126	CLCNKA		1.263E-15					CLCNKA		
V3SVHSHC_6978272	CASP8		1.287E-15					CASP8		
V3SVHSHC_7757336	TNKS2		1.316E-15					TNKS2		
V3SVHSHC_6363977	DNALI1		1.331E-15					DNALI1		
V3SVHSHC_5663288	KIAA1683		1.342E-15					KIAA1683		
V3SVHSHC_8258606	ZNF131		1.350E-15					ZNF131		
V3SVHSHC_5945240	CSNK2A3		1.446E-15					CSNK2A3		
V3SVHSHC_10580288	ZWINT		1.520E-15					ZWINT		
V3SVHSHC_8521781	PIP5KL1		1.549E-15					PIP5KL1		
V3SVHSHC_7408856	CYB5RL		1.644E-15					CYB5RL		
V3SVHSHC_4942700	SYNJ2		1.705E-15					SYNJ2		
V3SVHSHC_8082683	KLRC4-KLRK1		1.740E-15					KLRC4-KLRK1		
V3SVHSHC_7764926	C8orf88		1.813E-15					C8orf88		
V3SVHSHC_6445586	Nprl2		1.820E-15					Nprl2		
V3SVHSHC_7021568	CDC25C		1.892E-15					CDC25C		
V3SVHSHC_10755155	FAM86C1		1.916E-15					FAM86C1		
V3SVHSHC_10376447	ANKLE2		1.970E-15	1.114E-16				ANKLE2	ANKLE2	
V3SVHSHC_9715391	C11orf95		2.052E-15					C11orf95		
V3SVHSHC_8639096	DET1		2.120E-15					DET1		
V3SVHSHC_9532175	LOC100996720		2.142E-15					LOC100996720		

V3SVHSHC_8893790	LRRC14B		2.208E-15					LRRC14B			
V3SVHSHC_5052920	CD86		2.265E-15					CD86			
V3SVHSHC_4941149	ERBB2IP		2.367E-15					ERBB2IP			
V3SVHSHC_6286229	FEM1C		2.511E-15					FEM1C			
V3SVHSHC_7961837	PLEKHA4		2.544E-15		1.578E-04			PLEKHA4		PLEKHA4
V3SVHSHC_6185546	KCMF1		2.553E-15					KCMF1		
V3SVHSHC_7545014	EML3		2.710E-15					EML3		
V3SVHSHC_10116044	OR1J2		2.720E-15					OR1J2		
V3SVHSHC_7560986	OR4F6		2.783E-15					OR4F6		
V3SVHSHC_9439874	OTP		2.799E-15					OTP		
V3SVHSHC_7272434	RERE		2.807E-15					RERE		
V3SVHSHC_6692294	KLRG2		3.027E-15					KLRG2		
V3SVHSHC_4850498	ZNF80		3.092E-15					ZNF80		
V3SVHSHC_10218443	CADM3		3.166E-15					CADM3		
V3SVHSHC_8927681	KRAS		3.342E-15					KRAS		
V3SVHSHC_8417105	VNN1		3.350E-15					VNN1		
V3SVHSHC_7480829	OR6C70		3.484E-15					OR6C70		
V3SVHSHC_6490895	MAD1L1		3.652E-15					MAD1L1		
V3SVHSHC_5724074	LOC101929829		3.769E-15					LOC10192 9829		
V3SVHSHC_9848810	SLC9A5		3.820E-15					SLC9A5		
V3SVHSHC_8423441	RSPH10B		3.871E-15					RSPH10B		
V3SVHSHC_8319689	DDA1		3.937E-15					DDA1		
V3SVHSHC_9523958	OR11H12		4.278E-15					OR11H12		
V3SVHSHC_5643752	KANSL1L		4.313E-15					KANSL1L		
V3SVHSHC_6294248	PLCB4		4.362E-15					PLCB4		
V3SVHSHC_9328532	PML		4.479E-15					PML		
V3SVHSHC_9000479	KLRD1		4.495E-15					KLRD1		
V3SVHSHC_9666782	MMP16		4.513E-15					MMP16		
V3SVHSHC_4775060	TEX26		4.625E-15					TEX26		
V3SVHSHC_10470695	PQBP1		4.988E-15					PQBP1		
V3SVHSHC_9421625	CACNG1		5.192E-15					CACNG1		
V3SVHSHC_9403112	FAM127A		5.196E-15					FAM127A		
V3SVHSHC_9842441	LOC101060861		5.220E-15					LOC10106 0861		
V3SVHSHC_7695890	ZNF185		5.231E-15					ZNF185		
V3SVHSHC_7984673	PPFIA2		5.383E-15					PPFIA2		
V3SVHSHC_9082781	ENTPD8		5.590E-15					ENTPD8		
V3SVHSHC_7592666	TTC34		6.213E-15					TTC34		
V3SVHSHC_5399354	DAGLA		6.343E-15					DAGLA		
V3SVHSHC_9040145	CCDC107		6.422E-15					CCDC107		
V3SVHSHC_4809446	ATP6V0E1		7.285E-15	1.719E-18				ATP6V0E1	ATP6V0E1	
V3SVHSHC_6086711	OPTC		7.487E-15					OPTC		
V3SVHSHC_6564320	RALGPS1		7.841E-15					RALGPS1		
V3SVHSHC_5432585	C2cd4a		8.267E-15					C2cd4a		
V3SVHSHC_9921641	UPP2		8.328E-15					UPP2		
V3SVHSHC_9517160	TMEM51		8.599E-15					TMEM51		
V3SVHSHC_9426146	REM1		9.101E-15					REM1		
V3SVHSHC_5438591	EHD1		1.068E-14					EHD1		
V3SVHSHC_6429119	IRX5		1.077E-14					IRX5		
V3SVHSHC_10672622	PCTP		1.123E-14					PCTP		
V3SVHSHC_4872311	KRTAP2-2		1.129E-14					KRTAP2-2		
V3SVHSHC_4960256	LYPD4		1.217E-14					LYPD4		
V3SVHSHC_5376188	SAG		1.225E-14					SAG		
V3SVHSHC_5534423	Tmem237		1.245E-14					Tmem237		

V3SVHSHC_10655363	KCNK1		1.279E-14					KCNK1		
V3SVHSHC_9966356	ZNF148		1.346E-14					ZNF148		
V3SVHSHC_10767431	ARHGAP40		1.354E-14					ARHGAP4<br>0		
V3SVHSHC_8435882	H2BFM		1.369E-14					H2BFM		
V3SVHSHC_7124627	RFPL4AL1		1.384E-14					RFPL4AL1		
V3SVHSHC_9726578	FBXL12		1.528E-14					FBXL12		
V3SVHSHC_7559798	ATP11AUN		1.573E-14					ATP11AU<br>N		
V3SVHSHC_10178909	GATSL3		1.701E-14					GATSL3		
V3SVHSHC_5480765	GFI1		1.751E-14					GFI1		
V3SVHSHC_6851750	LRRTM3		1.766E-14					LRRTM3		
V3SVHSHC_5855942	KCNH2		1.785E-14					KCNH2		
V3SVHSHC_9895010	LTB4R2		1.788E-14					LTB4R2		
V3SVHSHC_8439380	HCAR1		1.789E-14					HCAR1		
V3SVHSHC_9620021	CCL19		1.807E-14					CCL19		
V3SVHSHC_6475484	ZEB2		1.819E-14					ZEB2		
V3SVHSHC_9469838	TLK2		1.886E-14					TLK2		
V3SVHSHC_6718958	PPP1R16B		1.891E-14					PPP1R16<br>B		
V3SVHSHC_7273721	CNOT7		1.993E-14					CNOT7		
V3SVHSHC_8920454	SYT14		2.065E-14					SYT14		
V3SVHSHC_6428195	PTPN18		2.074E-14					PTPN18		
V3SVHSHC_5009228	APPBP2		2.121E-14					APPBP2		
V3SVHSHC_5173007	TMEM198		2.178E-14					TMEM198		
V3SVHSHC_6626525	ASB9		2.194E-14					ASB9		
V3SVHSHC_9597185	KRTAP5-5		2.379E-14					KRTAP5-5		
V3SVHSHC_7747931	C4ORF6		2.448E-14					C4ORF6		
V3SVHSHC_5976557	DLG1		2.451E-14					DLG1		
V3SVHSHC_6053777	GPS2		2.471E-14					GPS2		
V3SVHSHC_9542141	TNFRSF13C		2.485E-14					TNFRSF1<br>3C		
V3SVHSHC_7008104	PRSS21		2.619E-14					PRSS21		
V3SVHSHC_6583922	COL9A2		2.629E-14					COL9A2		
V3SVHSHC_7796441	KLHL1		2.731E-14					KLHL1		
V3SVHSHC_5000747	OR7C2		2.740E-14					OR7C2		
V3SVHSHC_5501753	KDSR		2.743E-14					KDSR		
V3SVHSHC_7175315	CTRC		2.850E-14					CTRC		
V3SVHSHC_10535276	GFPT2		2.901E-14					GFPT2		
V3SVHSHC_6365759	DGKG		2.976E-14					DGKG		
V3SVHSHC_5646029	SFXN3		3.082E-14					SFXN3		
V3SVHSHC_8371400	PSAPL1		3.135E-14					PSAPL1		
V3SVHSHC_5081828	LMO2		3.176E-14					LMO2		
V3SVHSHC_9761063	LOC101929451		3.333E-14					LOC10192<br>9451		
V3SVHSHC_5645435	PPRC1		3.481E-14					PPRC1		
V3SVHSHC_10838513	S100A4		3.535E-14					S100A4		
V3SVHSHC_7766840	FUT6		3.555E-14					FUT6		
V3SVHSHC_7419218	CCDC129		3.646E-14					CCDC129		
V3SVHSHC_8204453	RNF125		3.949E-14					RNF125		
V3SVHSHC_8610617	OR13C3		3.983E-14					OR13C3		
V3SVHSHC_7016255	NUS1		4.006E-14					NUS1		
V3SVHSHC_7653089	TCN2		4.033E-14					TCN2		
V3SVHSHC_7763375	TMTC1		4.214E-14					TMTC1		
V3SVHSHC_7391300	VRK3		4.332E-14					VRK3		
V3SVHSHC_8329523	CDPF1		4.380E-14					CDPF1		

V3SVHSHC_4685267	BPIFB3		4.523E-14					BPIFB3		
V3SVHSHC_5546930	VSTM2B		4.719E-14					VSTM2B		
V3SVHSHC_6980714	MYO5B		4.911E-14					MYO5B		
V3SVHSHC_7020908	SLC27A3		5.012E-14					SLC27A3		
V3SVHSHC_9296654	RPUSD1		5.074E-14					RPUSD1		
V3SVHSHC_7053083	FHL5		5.165E-14					FHL5		
V3SVHSHC_8180264	SLMAP		5.683E-14					SLMAP		
V3SVHSHC_10313384	CXorf57		5.783E-14					CXorf57		
V3SVHSHC_10610351	DUT		5.803E-14					DUT		
V3SVHSHC_5537525	LOC645202		5.973E-14					LOC64520 2		
V3SVHSHC_5040941	ADD1		6.111E-14	3.168E-09				ADD1	ADD1	
V3SVHSHC_6861419	RIPK1		6.138E-14					RIPK1		
V3SVHSHC_6279431	EHD4		6.194E-14					EHD4		
V3SVHSHC_10752812	C1orf56		6.215E-14					C1orf56		
V3SVHSHC_10209566	RANBP17		6.229E-14					RANBP17		
V3SVHSHC_6122351	LRCH4		6.282E-14					LRCH4		
V3SVHSHC_9724367	ANAPC7		6.411E-14					ANAPC7		
V3SVHSHC_6586067	RSPH4A		6.496E-14					RSPH4A		
V3SVHSHC_8719517	HS3ST6		6.770E-14					HS3ST6		
V3SVHSHC_6033284	STK38		6.845E-14		2.018E-06			STK38		STK38
V3SVHSHC_7002593	CCDC80		6.906E-14					CCDC80		
V3SVHSHC_8198744	GCA		7.106E-14					GCA		
V3SVHSHC_9608867	PAN2		7.291E-14					PAN2		
V3SVHSHC_5906597	LURAP1L		7.431E-14					LURAP1L		
V3SVHSHC_8204618	RBMY1J		7.637E-14					RBMY1J		
V3SVHSHC_10350179	MOSPD1		7.732E-14					MOSPD1		
V3SVHSHC_7216763	CABLES1		7.908E-14					CABLES1		
V3SVHSHC_8390738	DPCR1		8.664E-14					DPCR1		
V3SVHSHC_7801358	HNRNPD		8.752E-14					HNRNPD		
V3SVHSHC_7144856	IDH3G		9.281E-14					IDH3G		
V3SVHSHC_10030310	LHPP		9.510E-14					LHPP		
V3SVHSHC_6090176	UXT		1.013E-13					UXT		
V3SVHSHC_5958572	LRRC10B		1.023E-13					LRRC10B		
V3SVHSHC_10224152	OR56B4		1.069E-13					OR56B4		
V3SVHSHC_8224781	OR12D3		1.103E-13					OR12D3		
V3SVHSHC_9809243	SEPT8		1.145E-13					SEPT8		
V3SVHSHC_8008598	PMM2		1.157E-13					PMM2		
V3SVHSHC_6696452	EPS15		1.163E-13					EPS15		
V3SVHSHC_5914451	SENP2		1.173E-13					SENP2		
V3SVHSHC_6197855	NETO2		1.302E-13					NETO2		
V3SVHSHC_10112579	NTSR2		1.317E-13					NTSR2		
V3SVHSHC_6162710	ZNF500		1.448E-13					ZNF500		
V3SVHSHC_5633126	ZNF267		1.570E-13					ZNF267		
V3SVHSHC_9147131	TMEM30A		1.600E-13					TMEM30A		
V3SVHSHC_10688627	SYTL1		1.638E-13					SYTL1		
V3SVHSHC_7262897	ADAM8		1.673E-13					ADAM8		
V3SVHSHC_8753078	COL25A1		1.740E-13					COL25A1		
V3SVHSHC_7857722	MLLT3		1.799E-13					MLLT3		
V3SVHSHC_4870100	MARS2		1.842E-13					MARS2		
V3SVHSHC_9068822	PADI1		1.947E-13		7.765E-12			PADI1		PADI1
V3SVHSHC_10765913	HPGDS		1.949E-13					HPGDS		
V3SVHSHC_8334011	LHFPL3		1.998E-13					LHFPL3		
V3SVHSHC_5326424	ADPRHL2		2.001E-13					ADPRHL2		
V3SVHSHC_4916069	BIRC7		2.049E-13					BIRC7		

V3SVHSHC_5284415	GAL3ST1		2.069E-13					GAL3ST1		
V3SVHSHC_6310913	ANXA8L1		2.130E-13					ANXA8L1		
V3SVHSHC_10764395	GJA4		2.183E-13					GJA4		
V3SVHSHC_9555374	TCTEX1D1		2.234E-13					TCTEX1D1		
V3SVHSHC_5636624	ACTC1		2.330E-13					ACTC1		
V3SVHSHC_7832114	PDE4A		2.356E-13					PDE4A		
V3SVHSHC_5117732	CCNJ		2.357E-13					CCNJ		
V3SVHSHC_7690148	WTAP		2.363E-13					WTAP		
V3SVHSHC_4719686	TRAM1		2.430E-13					TRAM1		
V3SVHSHC_6607220	SERPINB10		2.460E-13					SERPINB10		
V3SVHSHC_8516699	TMC6		2.675E-13					TMC6		
V3SVHSHC_6500333	THBS4		2.731E-13					THBS4		
V3SVHSHC_10206134	BRF2		2.793E-13					BRF2		
V3SVHSHC_9658598	IL3		2.824E-13					IL3		
V3SVHSHC_10091492	GALNT1		2.903E-13					GALNT1		
V3SVHSHC_8251148	ATP5G2		2.934E-13					ATP5G2		
V3SVHSHC_5531684	TEAD1		2.945E-13					TEAD1		
V3SVHSHC_10012325	MCTP1		3.011E-13					MCTP1		
V3SVHSHC_6961343	TP53TG3D		3.019E-13					TP53TG3D		
V3SVHSHC_9803798	DEFA4		3.032E-13					DEFA4		
V3SVHSHC_7073378	ACTR3		3.079E-13					ACTR3		
V3SVHSHC_8777828	ALOXE3		3.383E-13					ALOXE3		
V3SVHSHC_7976885	MCC		3.444E-13					MCC		
V3SVHSHC_9746642	SCAMP5		3.492E-13					SCAMP5		
V3SVHSHC_8418326	PCDHGA11		3.533E-13					PCDHGA11		
V3SVHSHC_9006419	PRSS56		3.545E-13					PRSS56		
V3SVHSHC_7314179	C5orf66		3.584E-13					C5orf66		
V3SVHSHC_6083741	NLRP3		3.691E-13					NLRP3		
V3SVHSHC_4962071	C9orf16		3.856E-13					C9orf16		
V3SVHSHC_10037075	Haus8		4.094E-13					Haus8		
V3SVHSHC_7652660	PARP6		4.231E-13					PARP6		
V3SVHSHC_8037176	OR4M2		4.359E-13					OR4M2		
V3SVHSHC_9390572	RAET1E		4.576E-13		5.242E-03			RAET1E		
V3SVHSHC_6459809	FAM134B		4.754E-13					FAM134B		
V3SVHSHC_8380904	KBTBD6		4.856E-13					KBTBD6		
V3SVHSHC_6840497	ACSL5		4.949E-13					ACSL5		
V3SVHSHC_4923461	POFUT1		5.229E-13					POFUT1		
V3SVHSHC_8670050	SLC2A12		5.252E-13					SLC2A12		
V3SVHSHC_10478978	AEBP1		5.365E-13					AEBP1		
V3SVHSHC_7065458	RGS4		5.566E-13					RGS4		
V3SVHSHC_10152410	TPI1		5.707E-13					TPI1		
V3SVHSHC_8390606	LAMC2		5.725E-13					LAMC2		
V3SVHSHC_6797894	KIAA1377		5.832E-13					KIAA1377		
V3SVHSHC_10214747	ANKS1A		6.280E-13					ANKS1A		
V3SVHSHC_8172344	PRNP		6.345E-13					PRNP		
V3SVHSHC_10814588	FOXC1		6.394E-13					FOXC1		
V3SVHSHC_10777298	TCEA3		6.441E-13					TCEA3		
V3SVHSHC_7308800	FILIP1L		6.496E-13					FILIP1L		
V3SVHSHC_9000314	KLHDC2		6.784E-13					KLHDC2		
V3SVHSHC_9818747	ADPRHL1		7.401E-13					ADPRHL1		
V3SVHSHC_8762450	CD300C		7.803E-13					CD300C		

V3SVHSHC_10482971	ELP5		8.192E-13					ELP5		
V3SVHSHC_8702027	FAM198B		8.390E-13					FAM198B		
V3SVHSHC_8690576	PKD1L2		8.411E-13					PKD1L2		
V3SVHSHC_5452616	SFXN4		8.731E-13					SFXN4		
V3SVHSHC_5359589	RAD51		8.860E-13					RAD51		
V3SVHSHC_8302463	SUCLA2		8.910E-13					SUCLA2		
V3SVHSHC_10271903	KIAA1324L		8.944E-13					KIAA1324L		
V3SVHSHC_5324147	ARSA		9.040E-13					ARSA		
V3SVHSHC_9607184	ARSF		9.437E-13					ARSF		
V3SVHSHC_8642924	PDE1A		9.554E-13					PDE1A		
V3SVHSHC_10663217	SIDT1		9.559E-13					SIDT1		
V3SVHSHC_8766641	NEUROG2		9.775E-13					NEUROG2		
V3SVHSHC_5321738	MYO7B		1.056E-12	6.683E-14				MYO7B	MYO7B	
V3SVHSHC_6789083	AICDA		1.067E-12					AICDA		
V3SVHSHC_5782253	LOC101927989		1.108E-12					LOC101927989		
V3SVHSHC_9367406	LOC730159		1.169E-12					LOC730159		
V3SVHSHC_5472614	TMEM151B		1.189E-12					TMEM151B		
V3SVHSHC_9960845	SHPRH		1.265E-12					SHPRH		
V3SVHSHC_5943425	WISP3		1.285E-12					WISP3		
V3SVHSHC_9185774	USF1		1.287E-12					USF1		
V3SVHSHC_6602072	PCDHGB4		1.311E-12					PCDHGB4		
V3SVHSHC_9394565	HNRNPU		1.322E-12					HNRNPU		
V3SVHSHC_7883231	PMM2		1.345E-12					PMM2		
V3SVHSHC_6605372	KAT6A		1.364E-12					KAT6A		
V3SVHSHC_7586198	ZNF273		1.396E-12					ZNF273		
V3SVHSHC_7427171	GSPT2		1.478E-12					GSPT2		
V3SVHSHC_7883858	C6ORF165		1.537E-12					C6ORF165		
V3SVHSHC_7165811	LOC101930300		1.609E-12					LOC101930300		
V3SVHSHC_6582965	KRTAP5-9		1.687E-12					KRTAP5-9		
V3SVHSHC_8145515	LOC100288562		1.777E-12					LOC100288562		
V3SVHSHC_8279561	CTSG		1.790E-12					CTSG		
V3SVHSHC_7569566	PPP2R5D		1.800E-12					PPP2R5D		
V3SVHSHC_7027310	PTGR2		1.850E-12					PTGR2		
V3SVHSHC_6363482	GRP		1.866E-12					GRP		
V3SVHSHC_9867455	PTGES3		2.169E-12					PTGES3		
V3SVHSHC_7124033	TAOK1		2.190E-12					TAOK1		
V3SVHSHC_10246592	RRM2B		2.220E-12					RRM2B		
V3SVHSHC_8387438	SPRN		2.260E-12					SPRN		
V3SVHSHC_8158913	LOC651959		2.333E-12					LOC651959		
V3SVHSHC_6465683	SFRP5		2.461E-12					SFRP5		
V3SVHSHC_6534686	SHFM1		2.492E-12					SHFM1		
V3SVHSHC_7907783	KRTAP10-7		2.550E-12	5.157E-04				KRTAP10-7	KRTAP10-7	
V3SVHSHC_9265304	OLFML1		2.781E-12					OLFML1		
V3SVHSHC_4855514	EZH1		2.868E-12					EZH1		
V3SVHSHC_5104103	CALY		2.912E-12					CALY		
V3SVHSHC_10644737	TCERG1L		2.929E-12					TCERG1L		
V3SVHSHC_10277843	SFTPC		3.004E-12					SFTPC		
V3SVHSHC_8796836	LMOD3		3.021E-12	1.256E-30				LMOD3	LMOD3	

V3SVHSHC_8985563	HES2		3.173E-12					HES2		
V3SVHSHC_9574052	KDM5C		3.290E-12					KDM5C		
V3SVHSHC_8126936	NHEJ1		3.318E-12					NHEJ1		
V3SVHSHC_7369091	SMYD5		3.354E-12					SMYD5		
V3SVHSHC_9555407	RGS21		3.625E-12					RGS21		
V3SVHSHC_5774663	TVP23B		3.646E-12					TVP23B		
V3SVHSHC_9758324	TRPM4		3.647E-12					TRPM4		
V3SVHSHC_6525413	VAC14		3.847E-12					VAC14		
V3SVHSHC_10202174	PAQR6		4.076E-12	9.916E-28				PAQR6	PAQR6	
V3SVHSHC_7043909	EBI3		4.181E-12					EBI3		
V3SVHSHC_10561412	ZNF92		4.312E-12					ZNF92		
V3SVHSHC_8697176	KRTAP7-1		4.349E-12					KRTAP7-1		
V3SVHSHC_9695657	NMUR1		4.374E-12					NMUR1		
V3SVHSHC_7793966	GRXCR1		4.418E-12					GRXCR1		
V3SVHSHC_7070573	GLIS2		4.473E-12					GLIS2		
V3SVHSHC_8653550	GSTT2B		4.484E-12					GSTT2B		
V3SVHSHC_6478751	ZNF737		4.546E-12					ZNF737		
V3SVHSHC_7122548	ZNF284		4.556E-12					ZNF284		
V3SVHSHC_10708691	LSMEM1		4.562E-12					LSMEM1		
V3SVHSHC_6426710	ZNF177		4.597E-12					ZNF177		
V3SVHSHC_6160829	PCDHA1		4.655E-12					PCDHA1		
V3SVHSHC_5901350	TOMM7		4.680E-12	5.960E-22				TOMM7	TOMM7	
V3SVHSHC_5925902	SLC6A5		4.722E-12					SLC6A5		
V3SVHSHC_9480035	Fam181b		4.726E-12					Fam181b		
V3SVHSHC_7029290	TRPC5OS		5.141E-12					TRPC5OS		
V3SVHSHC_5722688	TENM3		5.236E-12					TENM3		
V3SVHSHC_5467367	PRC1		5.674E-12		5.081E-12			PRC1		PRC1
V3SVHSHC_6752420	AMER2		5.690E-12					AMER2		
V3SVHSHC_4727012	EXOC1		5.817E-12					EXOC1		
V3SVHSHC_6664277	SNAP23		5.877E-12					SNAP23		
V3SVHSHC_7268936	FOSL2		5.900E-12					FOSL2		
V3SVHSHC_4990484	MYO1C		6.301E-12					MYO1C		
V3SVHSHC_8004572	PAK1IP1		6.419E-12					PAK1IP1		
V3SVHSHC_5705462	GGT6		6.610E-12					GGT6		
V3SVHSHC_6086942	NXN		6.759E-12					NXN		
V3SVHSHC_9465779	BANF1		7.328E-12					BANF1		
V3SVHSHC_8132282	Mob1b		7.647E-12					Mob1b		
V3SVHSHC_7987874	UGT2B17		8.088E-12					UGT2B17		
V3SVHSHC_9618338	DEFB107A		8.096E-12					DEFB107A		
V3SVHSHC_7033250	SMARCA2		8.694E-12					SMARCA2		
V3SVHSHC_6055130	RRAS		8.975E-12					RRAS		
V3SVHSHC_7260323	KIAA1377		9.643E-12					KIAA1377		
V3SVHSHC_4842644	CARM1		1.002E-11					CARM1		
V3SVHSHC_5592767	CPNE4		1.028E-11					CPNE4		
V3SVHSHC_5584979	HELLS		1.039E-11					HELLS		
V3SVHSHC_9145382	LRRC43		1.046E-11					LRRC43		
V3SVHSHC_7213232	XPNPEP1		1.065E-11					XPNPEP1		
V3SVHSHC_10371563	TMEM216		1.102E-11					TMEM216		
V3SVHSHC_6894683	VAMP1		1.172E-11					VAMP1		
V3SVHSHC_6945503	GATS		1.201E-11					GATS		
V3SVHSHC_5649791	SMTNL1		1.261E-11					SMTNL1		
V3SVHSHC_5898149	FAM160B2		1.318E-11					FAM160B2		
V3SVHSHC_5751233	ARHGAP9		1.323E-11					ARHGAP9		
V3SVHSHC_9398657	LOC100132731		1.344E-11	1.342E-07				LOC10013 2731	LOC10013 2731	

V3SVHSHC_9387107	POLR2K		1.368E-11					POLR2K		
V3SVHSHC_6629660	DNM3		1.369E-11					DNM3		
V3SVHSHC_7510661	PARP16		1.377E-11					PARP16		
V3SVHSHC_10131653	CDKL3		1.395E-11					CDKL3		
V3SVHSHC_6212111	LAT2		1.551E-11		1.643E-15			LAT2		LAT2
V3SVHSHC_7209569	TCP11L1		1.566E-11					TCP11L1		
V3SVHSHC_8656157	ASPH		1.569E-11					ASPH		
V3SVHSHC_10746707	TMEM206		1.576E-11					TMEM206		
V3SVHSHC_6279233	ZNF155		1.594E-11					ZNF155		
V3SVHSHC_10657904	VPS37B		1.628E-11					VPS37B		
V3SVHSHC_8107730	PQBP1		1.733E-11					PQBP1		
V3SVHSHC_7943324	HIAT1		1.801E-11					HIAT1		
V3SVHSHC_4996820	PHIP		1.874E-11					PHIP		
V3SVHSHC_9099446	SOS2		1.903E-11					SOS2		
V3SVHSHC_9656189	GAGE12J		2.030E-11					GAGE12J		
V3SVHSHC_10785977	OTUD6B		2.113E-11					OTUD6B		
V3SVHSHC_7479707	RAB3GAP2		2.141E-11					RAB3GAP<br>2		
V3SVHSHC_4654148	Krt222		2.154E-11					Krt222		
V3SVHSHC_5616362	IL1RL1		2.181E-11					IL1RL1		
V3SVHSHC_5215115	PDCD1		2.338E-11	1.936E-10				PDCD1	PDCD1	
V3SVHSHC_9940979	PAQR6		2.371E-11					PAQR6		
V3SVHSHC_8431295	C1orf68		2.414E-11					C1orf68		
V3SVHSHC_6534257	C8orf33		2.444E-11					C8orf33		
V3SVHSHC_5632565	KRT35		2.476E-11					KRT35		
V3SVHSHC_6307943	SLC16A4		2.583E-11					SLC16A4		
V3SVHSHC_10126736	FOS		2.648E-11					FOS		
V3SVHSHC_7188515	OR6C74		2.680E-11					OR6C74		
V3SVHSHC_6068396	C1orf162		2.708E-11					C1orf162		
V3SVHSHC_6054833	ATAD5		2.753E-11					ATAD5		
V3SVHSHC_9005528	SLC25A40		2.840E-11					SLC25A40		
V3SVHSHC_8554616	C5orf38		2.912E-11					C5orf38		
V3SVHSHC_8765915	KIT		2.940E-11					KIT		
V3SVHSHC_10557518	ISM2		2.947E-11					ISM2		
V3SVHSHC_9280583	ITPR2		3.067E-11					ITPR2		
V3SVHSHC_4647680	OR2L8		3.145E-11					OR2L8		
V3SVHSHC_10094957	SFTA2		3.226E-11					SFTA2		
V3SVHSHC_6771593	RAD54L2		3.516E-11					RAD54L2		
V3SVHSHC_6370445	SETD7		3.623E-11					SETD7		
V3SVHSHC_5615141	N6AMT1		3.632E-11					N6AMT1		
V3SVHSHC_4788293	KIAA1024L		3.780E-11					KIAA1024<br>L		
V3SVHSHC_9310646	NFKBIB		3.879E-11					NFKBIB		
V3SVHSHC_6420605	LOC100652807		3.943E-11					LOC10065<br>2807		
V3SVHSHC_10185905	SLC22A24		3.987E-11					SLC22A24		
V3SVHSHC_9036713	AARSD1		4.047E-11					AARSD1		
V3SVHSHC_6368795	SEC31B		4.166E-11					SEC31B		
V3SVHSHC_10195310	SLC35B1		4.290E-11					SLC35B1		
V3SVHSHC_9751064	CGB8		4.644E-11					CGB8		
V3SVHSHC_8118158	TCF12		4.736E-11					TCF12		
V3SVHSHC_6057341	RAP2B		4.746E-11					RAP2B		
V3SVHSHC_8465912	TPSD1		4.773E-11					TPSD1		
V3SVHSHC_9613586	C9orf163		4.939E-11					C9orf163		
V3SVHSHC_10330478	TACC2		5.176E-11					TACC2		

V3SVHSHC_9358100	GAPDHS		5.196E-11					GAPDHS		
V3SVHSHC_8444462	ZCCHC4		5.339E-11					ZCCHC4		
V3SVHSHC_9170759	C19orf48		5.358E-11					C19orf48		
V3SVHSHC_8697011	SESN2		5.428E-11					SESN2		
V3SVHSHC_10762844	RNF7		5.556E-11					RNF7		
V3SVHSHC_4846736	Snrnp25		5.955E-11		3.774E-03			Snrnp25		
V3SVHSHC_6536600	GIN1		5.995E-11					GIN1		
V3SVHSHC_10109345	TNFRSF6B		6.316E-11					TNFRSF6<br>B		
V3SVHSHC_9065951	AQP1		6.367E-11					AQP1		
V3SVHSHC_6793406	DLX1		6.520E-11	8.107E-38				DLX1	DLX1	
V3SVHSHC_9441557	CD74		6.520E-11					CD74		
V3SVHSHC_6906530	PDHA1		6.530E-11					PDHA1		
V3SVHSHC_5582207	FABP5		6.590E-11					FABP5		
V3SVHSHC_8291243	NDUFA11		6.728E-11					NDUFA11		
V3SVHSHC_6156440	TTC4		7.733E-11					TTC4		
V3SVHSHC_4771793	VBP1		7.759E-11					VBP1		
V3SVHSHC_4838024	SCARA5		7.784E-11					SCARA5		
V3SVHSHC_7727207	GIF		8.179E-11					GIF		
V3SVHSHC_10719812	DCDC2C		8.242E-11					DCDC2C		
V3SVHSHC_10597118	LOC101928951		8.326E-11					LOC10192<br>8951		
V3SVHSHC_10389515	EEF2		8.367E-11					EEF2		
V3SVHSHC_7829804	MSC		9.020E-11					MSC		
V3SVHSHC_7213364	KDR		9.642E-11					KDR		
V3SVHSHC_8691302	FUCA1		9.968E-11					FUCA1		
V3SVHSHC_8232074	ZNF518B		1.003E-10					ZNF518B		
V3SVHSHC_9555440	CELSR1		1.092E-10					CELSR1		
V3SVHSHC_6901118	OR13D1		1.160E-10					OR13D1		
V3SVHSHC_9975233	PCDHGA1		1.219E-10					PCDHGA1		
V3SVHSHC_9379781	TRIM42		1.252E-10					TRIM42		
V3SVHSHC_7330646	EPN2		1.264E-10					EPN2		
V3SVHSHC_9497756	PCDHGB4		1.309E-10					PCDHGB4		
V3SVHSHC_10601507	TYMP		1.310E-10					TYMP		
V3SVHSHC_6522938	FAM151A		1.326E-10					FAM151A		
V3SVHSHC_10610714	PAOX		1.398E-10					PAOX		
V3SVHSHC_5705990	Slc46a2		1.422E-10					Slc46a2		
V3SVHSHC_8435750	DZANK1		1.449E-10					DZANK1		
V3SVHSHC_7640450	EDN3		1.563E-10					EDN3		
V3SVHSHC_10756937	MTRNR2L4		1.587E-10					MTRNR2L<br>4		
V3SVHSHC_7864322	SH2B3		1.649E-10					SH2B3		
V3SVHSHC_8505050	PADI1		1.718E-10					PADI1		
V3SVHSHC_8994209	NAMPT		1.725E-10		3.123E-01			NAMPT		
V3SVHSHC_5580755	ZSCAN23		1.761E-10					ZSCAN23		
V3SVHSHC_9677507	CCL11		1.841E-10					CCL11		
V3SVHSHC_10663547	MYO3A		1.881E-10					MYO3A		
V3SVHSHC_8817230	TMEM9		1.901E-10					TMEM9		
V3SVHSHC_5820038	TRIM62		1.957E-10					TRIM62		
V3SVHSHC_7497857	KLRK1		2.006E-10					KLRK1		
V3SVHSHC_4721171	C2ORF16		2.120E-10					C2ORF16		
V3SVHSHC_6693416	KRT75		2.199E-10					KRT75		
V3SVHSHC_6281114	RASGRP3		2.249E-10					RASGRP3		
V3SVHSHC_8996024	PCDHGC4		2.350E-10					PCDHGC4		

V3SVHSHC_8209271	LOC101929950		2.484E-10					LOC101929950		
V3SVHSHC_6799280	NXPE2		2.571E-10					NXPE2		
V3SVHSHC_8907782	OR52N2		2.694E-10	1.773E-25				OR52N2	OR52N2	
V3SVHSHC_8979227	TMEM67		2.840E-10					TMEM67		
V3SVHSHC_7188878	ZBTB8B		2.856E-10					ZBTB8B		
V3SVHSHC_9008795	BACE2		2.921E-10					BACE2		
V3SVHSHC_8405984	C22orf46		2.921E-10					C22orf46		
V3SVHSHC_10252004	SHISA4		3.046E-10					SHISA4		
V3SVHSHC_6927650	WHSC1L1		3.095E-10					WHSC1L1		
V3SVHSHC_7443308	LSM11		3.097E-10					LSM11		
V3SVHSHC_6986423	SMAD2		3.362E-10					SMAD2		
V3SVHSHC_7433837	DUSP26		3.460E-10					DUSP26		
V3SVHSHC_6313652	FXYD4		3.569E-10					FXYD4		
V3SVHSHC_10794293	CPO		3.707E-10	1.277E-10				CPO	CPO	
V3SVHSHC_6737570	Krtdap		3.751E-10					Krtdap		
V3SVHSHC_8994011	TMEM165		3.828E-10		2.560E-04			TMEM165		TMEM165
V3SVHSHC_5206337	FAM107B		3.975E-10					FAM107B		
V3SVHSHC_7812248	OR11H4		4.043E-10					OR11H4		
V3SVHSHC_9705953	B3GAT2		4.158E-10					B3GAT2		
V3SVHSHC_10734068	INPP5D		4.173E-10					INPP5D		
V3SVHSHC_5137994	TRPV1		4.323E-10					TRPV1		
V3SVHSHC_9934247	ZNF845		5.070E-10					ZNF845		
V3SVHSHC_8576330	NAB1		5.075E-10		1.153E-14			NAB1		NAB1
V3SVHSHC_9830660	VN1R2		5.123E-10					VN1R2		
V3SVHSHC_9236198	ABCC4		5.178E-10					ABCC4		
V3SVHSHC_7874882	SLC10A5		5.237E-10					SLC10A5		
V3SVHSHC_5612501	LOC100996906		5.237E-10					LOC100996906		
V3SVHSHC_8547851	GRPEL2		5.345E-10					GRPEL2		
V3SVHSHC_5123639	BNIP2		5.519E-10					BNIP2		
V3SVHSHC_5253857	SCAMP2		5.633E-10					SCAMP2		
V3SVHSHC_6123803	FAM195B		6.021E-10	4.889E-28				FAM195B	FAM195B	
V3SVHSHC_5821523	CHRNA9		6.023E-10					CHRNA9		
V3SVHSHC_8328500	ZNF793		6.157E-10					ZNF793		
V3SVHSHC_8278208	SLC2A2		6.349E-10					SLC2A2		
V3SVHSHC_10138979	SECISBP2		6.357E-10					SECISBP2		
V3SVHSHC_9188084	GNA14		6.527E-10					GNA14		
V3SVHSHC_10406741	CHAF1A		6.548E-10					CHAF1A		
V3SVHSHC_9432515	Haus7		6.782E-10					Haus7		
V3SVHSHC_4654247	LOC101928892		6.824E-10	7.027E-18				LOC101928892	LOC101928892	
V3SVHSHC_4896500	CTNNB1		6.827E-10					CTNNB1		
V3SVHSHC_5336819	GK		6.920E-10					GK		
V3SVHSHC_7317644	CDK19		6.945E-10					CDK19		
V3SVHSHC_10668398	RFT1		7.196E-10					RFT1		
V3SVHSHC_8954345	SLC9B1		7.271E-10					SLC9B1		
V3SVHSHC_5266991	C21ORF33		7.404E-10					C21ORF33		
V3SVHSHC_8434430	SLC10A1		7.462E-10					SLC10A1		
V3SVHSHC_4941710	S1PR5		7.905E-10					S1PR5		
V3SVHSHC_10798550	MUC3B		7.952E-10					MUC3B		
V3SVHSHC_6451328	PARP10		8.037E-10					PARP10		
V3SVHSHC_6093938	ROPN1L		8.412E-10					ROPN1L		
V3SVHSHC_8707703	LOC101930400		8.790E-10		3.100E-16			LOC101930400		LOC101930400

V3SVHSHC_10162376	NLRP14		8.954E-10					NLRP14		
V3SVHSHC_9393311	BCKDHA		9.182E-10					BCKDHA		
V3SVHSHC_7682657	ASB4		9.713E-10					ASB4		
V3SVHSHC_9391067	TEDDM1		9.784E-10					TEDDM1		
V3SVHSHC_8649194	LOC101930432		1.010E-09					LOC10193<br>0432		
V3SVHSHC_7574549	HLA-DOB		1.026E-09					HLA-DOB		
V3SVHSHC_9518381	LOC729458		1.035E-09					LOC72945<br>8		
V3SVHSHC_8273456	FASTKD5		1.053E-09					FASTKD5		
V3SVHSHC_10527950	KLHL26		1.119E-09					KLHL26		
V3SVHSHC_8400506	C6orf183		1.120E-09					C6orf183		
V3SVHSHC_10474787	CASK		1.205E-09					CASK		
V3SVHSHC_8131985	SMARCD1		1.205E-09					SMARCD1		
V3SVHSHC_6242339	ZSCAN12		1.329E-09					ZSCAN12		
V3SVHSHC_5327249	C19orf68		1.333E-09					C19orf68		
V3SVHSHC_7604579	CAMK2N1		1.352E-09					CAMK2N1		
V3SVHSHC_8996717	C1orf194		1.380E-09					C1orf194		
V3SVHSHC_7112747	PPM1J		1.478E-09					PPM1J		
V3SVHSHC_10686053	LEMD3		1.539E-09					LEMD3		
V3SVHSHC_10687241	SLIT2		1.552E-09					SLIT2		
V3SVHSHC_4917059	IGFALS		1.553E-09					IGFALS		
V3SVHSHC_9632495	DYDC1		1.618E-09					DYDC1		
V3SVHSHC_10011896	RANBP17		1.643E-09					RANBP17		
V3SVHSHC_7131656	FAM189A2		1.652E-09					FAM189A2		
V3SVHSHC_6182180	Hm13		1.685E-09					Hm13		
V3SVHSHC_9920783	DEFB104A		1.730E-09					DEFB104A		
V3SVHSHC_6629627	TRPM2		1.739E-09					TRPM2		
V3SVHSHC_8241941	DGKK		1.882E-09		3.172E-04			DGKK		DGKK
V3SVHSHC_8130401	FAM189B		1.890E-09					FAM189B		
V3SVHSHC_8183663	PTGES3L		1.896E-09					PTGES3L		
V3SVHSHC_9131093	SLC36A3		2.084E-09					SLC36A3		
V3SVHSHC_6445883	WDR45B		2.116E-09					WDR45B		
V3SVHSHC_5357246	BFSP1		2.123E-09					BFSP1		
V3SVHSHC_4836242	FAM166A		2.204E-09					FAM166A		
V3SVHSHC_10564712	LRRC37B		2.236E-09					LRRC37B		
V3SVHSHC_7101461	ZNF282		2.244E-09					ZNF282		
V3SVHSHC_5131427	CT83		2.343E-09					CT83		
V3SVHSHC_8419811	S100A14		2.364E-09					S100A14		
V3SVHSHC_5110736	TRIO		2.395E-09					TRIO		
V3SVHSHC_6109151	RESP18		2.400E-09					RESP18		
V3SVHSHC_9562502	VCY		2.442E-09					VCY		
V3SVHSHC_6993815	FRRS1L		2.596E-09					FRRS1L		
V3SVHSHC_9731858	TSN		2.785E-09		6.810E-20			TSN		TSN
V3SVHSHC_7029719	CLPTM1L		2.877E-09					CLPTM1L		
V3SVHSHC_7344770	HOXA7		2.939E-09					HOXA7		
V3SVHSHC_8326586	AIFM2		3.085E-09		2.093E-01			AIFM2		
V3SVHSHC_9753407	GAS7		3.254E-09					GAS7		
V3SVHSHC_10014074	GPR137B		3.292E-09					GPR137B		
V3SVHSHC_8370080	AGBL4		3.360E-09					AGBL4		
V3SVHSHC_10751030	GPR68		3.386E-09					GPR68		
V3SVHSHC_9945368	ALPI		3.411E-09					ALPI		
V3SVHSHC_5489972	LOC101060588		3.487E-09					LOC10106<br>0588		
V3SVHSHC_7998302	CNOT7		3.670E-09					CNOT7		

V3SVHSHC_6968900	ASB7		3.733E-09					ASB7		
V3SVHSHC_7626821	SPI1		4.285E-09					SPI1		
V3SVHSHC_5107403	BRD8		4.353E-09					BRD8		
V3SVHSHC_10334900	SDC3		4.416E-09					SDC3		
V3SVHSHC_10279097	OR10Z1		4.524E-09					OR10Z1		
V3SVHSHC_6238940	Zdbf2		4.862E-09					Zdbf2		
V3SVHSHC_5305172	OBSCN		4.965E-09					OBSCN		
V3SVHSHC_7245275	NFE2L1		5.039E-09					NFE2L1		
V3SVHSHC_9629327	TRIML1		5.107E-09					TRIML1		
V3SVHSHC_8634641	FAM46A		5.601E-09					FAM46A		
V3SVHSHC_7686254	PLK2		5.665E-09					PLK2		
V3SVHSHC_6165812	TCP11X1		5.853E-09					TCP11X1		
V3SVHSHC_5851586	POC5		5.924E-09					POC5		
V3SVHSHC_7406480	ACTRT1		6.208E-09					ACTRT1		
V3SVHSHC_9241181	VPS4A		6.464E-09					VPS4A		
V3SVHSHC_7722653	CLIP1		6.553E-09		2.745E-16			CLIP1		CLIP1
V3SVHSHC_5207063	HEATR3		6.554E-09					HEATR3		
V3SVHSHC_10335527	KRTAP2-3		6.606E-09					KRTAP2-3		
V3SVHSHC_7235969	ARFIP1		7.598E-09					ARFIP1		
V3SVHSHC_7564748	NUDT6		7.812E-09					NUDT6		
V3SVHSHC_8760404	MEAF6		7.898E-09		3.328E-08			MEAF6		MEAF6
V3SVHSHC_7498649	PJA1		8.405E-09					PJA1		
V3SVHSHC_9698660	ZNF200		8.480E-09		9.248E-03			ZNF200		
V3SVHSHC_6111362	SRRD		8.581E-09					SRRD		
V3SVHSHC_10245437	HPDL		8.888E-09					HPDL		
V3SVHSHC_4859639	BOLA2B		8.923E-09					BOLA2B		
V3SVHSHC_4643588	CNTF		9.113E-09					CNTF		
V3SVHSHC_8178977	SLC39A12		9.209E-09					SLC39A12		
V3SVHSHC_5916134	PCDH1		9.521E-09					PCDH1		
V3SVHSHC_8499671	PHKA1		9.585E-09					PHKA1		
V3SVHSHC_10461851	ZFP36		1.006E-08					ZFP36		
V3SVHSHC_5452187	FOXL1		1.055E-08					FOXL1		
V3SVHSHC_4672397	PNMA1		1.102E-08	1.289E-20				PNMA1	PNMA1	
V3SVHSHC_6654971	UBA52		1.109E-08					UBA52		
V3SVHSHC_8492015	LGALS7		1.112E-08					LGALS7		
V3SVHSHC_10291835	EFCAB13		1.130E-08					EFCAB13		
V3SVHSHC_6951311	NPFF		1.165E-08					NPFF		
V3SVHSHC_5583593	FKBP14		1.198E-08					FKBP14		
V3SVHSHC_10543328	GJC2		1.211E-08					GJC2		
V3SVHSHC_6500102	PPP1R14D		1.268E-08					PPP1R14D		
V3SVHSHC_7456112	SLC7A13		1.284E-08					SLC7A13		
V3SVHSHC_10245800	SPATA5		1.295E-08					SPATA5		
V3SVHSHC_9502607	KLHL6		1.298E-08					KLHL6		
V3SVHSHC_7528382	MANSC1		1.326E-08					MANSC1		
V3SVHSHC_5221979	CNDP2		1.418E-08					CNDP2		
V3SVHSHC_6576827	WDR53		1.491E-08	4.845E-12				WDR53	WDR53	
V3SVHSHC_6285305	IL17D		1.507E-08					IL17D		
V3SVHSHC_8986883	KCNRG		1.548E-08					KCNRG		
V3SVHSHC_8816966	EIF3F		1.580E-08					EIF3F		
V3SVHSHC_7609430	TUBGCP5		1.616E-08					TUBGCP5		
V3SVHSHC_5506472	ZNF732		1.620E-08		4.891E-01			ZNF732		
V3SVHSHC_6130733	WAC		1.649E-08					WAC		
V3SVHSHC_5919302	TBX19		1.741E-08					TBX19		
V3SVHSHC_5810831	PLA2G4E		1.800E-08					PLA2G4E		

V3SVHSHC_7881482	TOR1B		1.854E-08					TOR1B		
V3SVHSHC_8403410	DUOXA2		1.905E-08					DUOXA2		
V3SVHSHC_5562440	KIAA1462		1.961E-08					KIAA1462		
V3SVHSHC_9121391	TLR2		2.003E-08					TLR2		
V3SVHSHC_8338928	FRAT2		2.005E-08		4.877E-01			FRAT2		
V3SVHSHC_9511913	Ttc39a		2.057E-08					Ttc39a		
V3SVHSHC_7201154	LOC645177		2.220E-08					LOC645177		
V3SVHSHC_10734266	SIKE1		2.227E-08					SIKE1		
V3SVHSHC_9883163	STOML1		2.247E-08					STOML1		
V3SVHSHC_10175741	ACE		2.329E-08					ACE		
V3SVHSHC_7065590	NANOS1		2.357E-08					NANOS1		
V3SVHSHC_7659491	SPDL1		2.370E-08					SPDL1		
V3SVHSHC_10150727	SGPP1		2.443E-08					SGPP1		
V3SVHSHC_7899467	PTGIS		2.498E-08		4.463E-23			PTGIS		PTGIS
V3SVHSHC_4642598	MCCD1		2.536E-08					MCCD1		
V3SVHSHC_6211022	RNF219		2.604E-08					RNF219		
V3SVHSHC_7687475	YBEY		2.746E-08					YBEY		
V3SVHSHC_6261380	PTPRZ1		2.803E-08					PTPRZ1		
V3SVHSHC_9711959	Ttc39a		2.834E-08					Ttc39a		
V3SVHSHC_7071167	NDUFA13		2.923E-08					NDUFA13		
V3SVHSHC_4632302	HOMER1		3.024E-08					HOMER1		
V3SVHSHC_10046216	MAP4K1		3.052E-08					MAP4K1		
V3SVHSHC_8830793	FAM217A		3.068E-08		1.959E-10			FAM217A		FAM217A
V3SVHSHC_8943983	TSR3		3.102E-08					TSR3		
V3SVHSHC_6837395	NDUFA11		3.102E-08					NDUFA11		
V3SVHSHC_7317512	SKI		3.150E-08					SKI		
V3SVHSHC_10556495	NEUROG2		3.159E-08					NEUROG2		
V3SVHSHC_7900721	MYH10		3.231E-08					MYH10		
V3SVHSHC_10820033	DDA1		3.365E-08					DDA1		
V3SVHSHC_9959162	FBLN7		3.699E-08					FBLN7		
V3SVHSHC_8352656	ZNF606		3.807E-08					ZNF606		
V3SVHSHC_8182640	RPUSD4		3.861E-08					RPUSD4		
V3SVHSHC_9242963	HELLS		3.982E-08					HELLS		
V3SVHSHC_9735158	UNCX		4.083E-08	1.668E-07				UNCX	UNCX	
V3SVHSHC_7681172	WRB		4.154E-08	1.370E-09				WRB	WRB	
V3SVHSHC_10022588	OPCML		4.550E-08					OPCML		
V3SVHSHC_6370544	TNIP1		4.924E-08					TNIP1		
V3SVHSHC_5083445	GPN2		5.089E-08					GPN2		
V3SVHSHC_5920853	SLC22A7		5.151E-08					SLC22A7		
V3SVHSHC_6452912	IKZF4		5.172E-08	6.119E-03				IKZF4		
V3SVHSHC_9541052	KATNAL2		5.372E-08					KATNAL2		
V3SVHSHC_8488022	Cul9		5.729E-08					Cul9		
V3SVHSHC_7515710	FLNC		5.843E-08					FLNC		
V3SVHSHC_9360212	C12orf49		5.916E-08					C12orf49		
V3SVHSHC_5055857	PTGR2		6.064E-08					PTGR2		
V3SVHSHC_6042557	SLC2A14		6.078E-08					SLC2A14		
V3SVHSHC_10489505	SLC25A51		6.136E-08					SLC25A51		
V3SVHSHC_9938768	RSPH10B2		6.147E-08					RSPH10B2		
V3SVHSHC_6579698	ST5		6.318E-08					ST5		
V3SVHSHC_10279130	ANKRD37		6.512E-08					ANKRD37		
V3SVHSHC_8624411	PHKG1		6.758E-08					PHKG1		
V3SVHSHC_9880325	CLCN6		7.457E-08					CLCN6		
V3SVHSHC_6210593	BUB1		7.636E-08					BUB1		

V3SVHSHC_6898346	ZNF705D		7.824E-08					ZNF705D		
V3SVHSHC_10818878	DEFB123		7.824E-08					DEFB123		
V3SVHSHC_8864684	CLEC9A		7.885E-08					CLEC9A		
V3SVHSHC_5807795	NKAPL		7.965E-08					NKAPL		
V3SVHSHC_10139111	LRP2		8.058E-08					LRP2		
V3SVHSHC_9619889	MPC1		8.229E-08					MPC1		
V3SVHSHC_8360477	OR4B1		8.325E-08					OR4B1		
V3SVHSHC_6432782	ESR2		8.462E-08					ESR2		
V3SVHSHC_8847689	HIF1AN		8.511E-08	4.278E-09				HIF1AN	HIF1AN	
V3SVHSHC_5229041	H2AFY		8.786E-08					H2AFY		
V3SVHSHC_10023611	BEST4		8.924E-08					BEST4		
V3SVHSHC_10473467	PTPN12		9.375E-08					PTPN12		
V3SVHSHC_5649857	RARA		9.601E-08					RARA		
V3SVHSHC_6082487	SLC29A1		9.624E-08					SLC29A1		
V3SVHSHC_5653322	OR1L6		9.922E-08					OR1L6		
V3SVHSHC_7381070	CUEDC2		1.011E-07					CUEDC2		
V3SVHSHC_9897584	CACNG8		1.065E-07					CACNG8		
V3SVHSHC_7642265	Mex3b		1.076E-07					Mex3b		
V3SVHSHC_8046317	COL25A1		1.123E-07		6.608E-06			COL25A1		COL25A1
V3SVHSHC_7291112	FGR		1.146E-07					FGR		
V3SVHSHC_10599395	SEPW1		1.197E-07					SEPW1		
V3SVHSHC_7935107	RAVER1		1.269E-07					RAVER1		
V3SVHSHC_8028827	TNFSF9		1.334E-07	2.757E-03				TNFSF9		
V3SVHSHC_6780569	PALM2-AKAP2		1.339E-07					PALM2-<br>AKAP2		
V3SVHSHC_7892339	ISPD		1.368E-07					ISPD		
V3SVHSHC_5217887	PSAP		1.371E-07					PSAP		
V3SVHSHC_7154690	PBK		1.380E-07					PBK		
V3SVHSHC_10021169	SELPLG		1.431E-07					SELPLG		
V3SVHSHC_7376714	TEKT5		1.455E-07		4.243E-01			TEKT5		
V3SVHSHC_6127763	CLN6		1.576E-07					CLN6		
V3SVHSHC_10689320	PNOC		1.607E-07					PNOC		
V3SVHSHC_8420603	ASPA		1.614E-07					ASPA		
V3SVHSHC_6559997	MRPL36		1.639E-07					MRPL36		
V3SVHSHC_6584516	MUC13		1.774E-07					MUC13		
V3SVHSHC_8737865	FBXW9		1.779E-07					FBXW9		
V3SVHSHC_6197525	ZNF488		1.784E-07	8.688E-05				ZNF488	ZNF488	
V3SVHSHC_7376912	TTC17		1.806E-07					TTC17		
V3SVHSHC_8911676	TARSL2		1.832E-07		2.168E-21			TARSL2		TARSL2
V3SVHSHC_5499410	TTLL11		1.856E-07					TTLL11		
V3SVHSHC_8086940	GDI2		2.048E-07					GDI2		
V3SVHSHC_7460435	CYP2C9		2.085E-07					CYP2C9		
V3SVHSHC_7963289	RUNX1T1		2.125E-07					RUNX1T1		
V3SVHSHC_7620518	GLIPR1L1		2.152E-07					GLIPR1L1		
V3SVHSHC_8946392	NDUFV2		2.157E-07					NDUFV2		
V3SVHSHC_10529072	ZNF727P		2.166E-07					ZNF727P		
V3SVHSHC_8549930	LOC101930592		2.268E-07					LOC10193<br>0592		
V3SVHSHC_9721496	KIAA0408		2.404E-07					KIAA0408		
V3SVHSHC_7468586	CEP57L1		2.481E-07					CEP57L1		
V3SVHSHC_5857229	BTG3		2.535E-07					BTG3		
V3SVHSHC_8782547	C1orf106		2.599E-07					C1orf106		
V3SVHSHC_5964083	HNRNPH1		2.636E-07					HNRNPH1		
V3SVHSHC_7563263	PCDH1		2.740E-07		1.283E-09			PCDH1		PCDH1
V3SVHSHC_10063838	SCN7A		2.776E-07					SCN7A		

V3SVHSHC_6619793	PRDM15		2.885E-07					PRDM15		
V3SVHSHC_10009685	SDHC		3.092E-07					SDHC		
V3SVHSHC_8393312	CYP2D6		3.210E-07					CYP2D6		
V3SVHSHC_9399614	PITPNB		3.243E-07					PITPNB		
V3SVHSHC_8090933	TCEAL1		3.415E-07					TCEAL1		
V3SVHSHC_7810004	FBXL5		3.492E-07					FBXL5		
V3SVHSHC_8500793	ZNF80		3.634E-07					ZNF80		
V3SVHSHC_6101330	TRIM46		3.679E-07					TRIM46		
V3SVHSHC_8959031	GOSR1		3.773E-07	1.345E-03				GOSR1		
V3SVHSHC_6999161	MAPK8IP2		4.313E-07					MAPK8IP2		
V3SVHSHC_7211417	F2RL1		4.445E-07					F2RL1		
V3SVHSHC_5348369	AGXT		4.544E-07					AGXT		
V3SVHSHC_10038362	TET2		4.578E-07					TET2		
V3SVHSHC_9443273	UBASH3A		4.692E-07					UBASH3A		
V3SVHSHC_8310350	ARHGAP9		4.849E-07					ARHGAP9		
V3SVHSHC_5706716	TRIM67		5.367E-07					TRIM67		
V3SVHSHC_9014966	MAP1A		5.654E-07					MAP1A		
V3SVHSHC_7566167	VPS26A		5.679E-07					VPS26A		
V3SVHSHC_9856136	CT47A8		5.759E-07	2.257E-29				CT47A8	CT47A8	
V3SVHSHC_5880956	ZNF408		5.959E-07					ZNF408		
V3SVHSHC_9064598	METTL9		6.190E-07					METTL9		
V3SVHSHC_7466375	PPIAL4D		6.288E-07					PPIAL4D		
V3SVHSHC_9692159	RBP1		6.401E-07					RBP1		
V3SVHSHC_4859738	KCNS2		6.580E-07					KCNS2		
V3SVHSHC_5511356	CHD9		6.790E-07					CHD9		
V3SVHSHC_6183533	AURKAIP1		6.823E-07					AURKAIP1		
V3SVHSHC_8206961	ABCB6		7.184E-07					ABCB6		
V3SVHSHC_10486865	IRS4		7.245E-07					IRS4		
V3SVHSHC_7372292	MAFF		7.257E-07					MAFF		
V3SVHSHC_5534489	MMS19		7.269E-07					MMS19		
V3SVHSHC_4703945	TOP2A		7.506E-07					TOP2A		
V3SVHSHC_7980581	ASIP		7.539E-07					ASIP		
V3SVHSHC_6898907	SH3BGRL2		7.568E-07					SH3BGRL2		
V3SVHSHC_6073247	PRSS45		8.701E-07					PRSS45		
V3SVHSHC_10832342	C1orf194		8.992E-07					C1orf194		
V3SVHSHC_5832776	MLST8		9.075E-07					MLST8		
V3SVHSHC_9263159	ADRBK2		9.095E-07					ADRBK2		
V3SVHSHC_7830497	ZNF302		9.189E-07					ZNF302		
V3SVHSHC_4889207	Fam150a		9.543E-07					Fam150a		
V3SVHSHC_10249364	NT5C1A		9.944E-07					NT5C1A		
V3SVHSHC_7756379	NCAPG		1.031E-06					NCAPG		
V3SVHSHC_6113837	DAPK1		1.074E-06					DAPK1		
V3SVHSHC_6892010	LOC101930553		1.163E-06					LOC101930553		
V3SVHSHC_6952565	MTX2		1.171E-06	2.274E-08				MTX2	MTX2	
V3SVHSHC_6082817	PDXDC1		1.230E-06					PDXDC1		
V3SVHSHC_5021801	ZNF727P		1.236E-06					ZNF727P		
V3SVHSHC_7051400	RNF180		1.340E-06					RNF180		
V3SVHSHC_4910426	HIST2H2BE		1.356E-06					HIST2H2BE		
V3SVHSHC_10115582	KRT71		1.390E-06					KRT71		
V3SVHSHC_10522637	TARSL2		1.406E-06					TARSL2		
V3SVHSHC_8604776	MAN2B2		1.449E-06					MAN2B2		
V3SVHSHC_9164258	INF2		1.453E-06					INF2		

V3SVHSHC_7672427	FGG		1.538E-06					FGG		
V3SVHSHC_9891908	ARRDC1		1.594E-06					ARRDC1		
V3SVHSHC_6392555	Aknad1		1.692E-06					Aknad1		
V3SVHSHC_8222735	FCRL6		1.792E-06					FCRL6		
V3SVHSHC_4846934	SLC4A7		1.819E-06					SLC4A7		
V3SVHSHC_10628666	TM2D2		1.848E-06					TM2D2		
V3SVHSHC_7041929	STX10		1.872E-06					STX10		
V3SVHSHC_5119052	CCDC17		1.908E-06					CCDC17		
V3SVHSHC_7068494	C18ORF25		2.036E-06					C18ORF25		
V3SVHSHC_9762317	ZAR1		2.106E-06					ZAR1		
V3SVHSHC_8329985	EML3		2.158E-06					EML3		
V3SVHSHC_9609296	EQTN		2.178E-06					EQTN		
V3SVHSHC_6738362	CACNA2D1		2.265E-06					CACNA2D1		
V3SVHSHC_5752223	BRSK1		2.280E-06					BRSK1		
V3SVHSHC_6896003	MSLN		2.455E-06					MSLN		
V3SVHSHC_8043677	LACTB2		2.514E-06					LACTB2		
V3SVHSHC_7987115	HCFC2		2.576E-06					HCFC2		
V3SVHSHC_6943886	DVL2		2.810E-06	1.529E-04				DVL2	DVL2	
V3SVHSHC_9548246	TNFRSF11B		2.987E-06					TNFRSF11B		
V3SVHSHC_7802876	RHOC		3.005E-06					RHOC		
V3SVHSHC_8083970	SEC24A		3.310E-06					SEC24A		
V3SVHSHC_8658005	ZNF814		3.324E-06					ZNF814		
V3SVHSHC_6288737	ANP32A		3.325E-06					ANP32A		
V3SVHSHC_5253461	GTPBP6		3.435E-06					GTPBP6		
V3SVHSHC_9482213	C4orf33		3.487E-06					C4orf33		
V3SVHSHC_9645992	CSNK1G3		3.627E-06					CSNK1G3		
V3SVHSHC_9936920	GPX8		3.696E-06					GPX8		
V3SVHSHC_7506338	CASP9		3.986E-06					CASP9		
V3SVHSHC_7949495	OR2Z1		4.016E-06					OR2Z1		
V3SVHSHC_5865677	Ntpcr		4.079E-06					Ntpcr		
V3SVHSHC_5699225	USP41		4.762E-06		1.695E-01			USP41		
V3SVHSHC_10240685	FGFBP1		4.869E-06					FGFBP1		
V3SVHSHC_8453339	NPAS2		4.886E-06					NPAS2		
V3SVHSHC_8486933	SCO1		5.008E-06					SCO1		
V3SVHSHC_10324175	CEMIP		5.095E-06					CEMIP		
V3SVHSHC_10155050	MTRNR2L10		5.115E-06					MTRNR2L10		
V3SVHSHC_10079150	BRD2		5.217E-06					BRD2		
V3SVHSHC_9581510	TOM1L2		5.260E-06					TOM1L2		
V3SVHSHC_6646424	OSER1		5.279E-06					OSER1		
V3SVHSHC_6446411	OR13G1		5.317E-06					OR13G1		
V3SVHSHC_7498352	CXCL9		5.505E-06					CXCL9		
V3SVHSHC_9106541	GPR182		5.588E-06					GPR182		
V3SVHSHC_8201747	NUTM2A		5.663E-06					NUTM2A		
V3SVHSHC_9443009	CTIF		6.236E-06					CTIF		
V3SVHSHC_5213498	LRRC52		6.298E-06					LRRC52		
V3SVHSHC_9244481	PLCB2		6.603E-06					PLCB2		
V3SVHSHC_8821289	PPP2R2A		7.661E-06					PPP2R2A		
V3SVHSHC_9349619	TYROBP		8.066E-06					TYROBP		
V3SVHSHC_6557984	KRT25		8.125E-06					KRT25		
V3SVHSHC_7903394	RANBP1		8.155E-06					RANBP1		
V3SVHSHC_9204287	LOC101929264		8.209E-06					LOC101929264		

V3SVHSHC_6325202	GAPT		8.643E-06					GAPT		
V3SVHSHC_9784262	ALDH3A1		8.708E-06					ALDH3A1		
V3SVHSHC_8191319	AGAP5		8.788E-06					AGAP5		
V3SVHSHC_5466014	RNF39		9.483E-06					RNF39		
V3SVHSHC_5048168	SLC6A8		9.816960E-06					SLC6A8		
V3SVHSHC_8912468	KRTAP4-2		1.040E-05					KRTAP4-2		
V3SVHSHC_9108884	BEX5		1.055E-05					BEX5		
V3SVHSHC_9665858	OPN1LW		1.086E-05					OPN1LW		
V3SVHSHC_9074300	URI1		1.099E-05					URI1		
V3SVHSHC_9091394	UQCRC2		1.118E-05					UQCRC2		
V3SVHSHC_9683645	SAC3D1		1.164E-05					SAC3D1		
V3SVHSHC_6419318	SMPDL3A		1.259E-05		2.211E-13			SMPDL3A		SMPDL3A
V3SVHSHC_7408922	HSPG2		1.341E-05					HSPG2		
V3SVHSHC_7157990	CDC42EP3		1.350E-05					CDC42EP3		
V3SVHSHC_5533796	FBXO21		1.376E-05					FBXO21		
V3SVHSHC_5263691	SIDT1		1.408E-05					SIDT1		
V3SVHSHC_6594152	SMARCD2		1.460E-05					SMARCD2		
V3SVHSHC_6692558	CDC34		1.547E-05					CDC34		
V3SVHSHC_9158615	S100A11		1.787E-05					S100A11		
V3SVHSHC_7465088	MAK		1.799E-05					MAK		
V3SVHSHC_5059454	RAD54L		1.925E-05					RAD54L		
V3SVHSHC_7628042	PRR15		1.939E-05					PRR15		
V3SVHSHC_5669624	HAPLN1		2.031E-05					HAPLN1		
V3SVHSHC_5625371	TAS2R16		2.210E-05					TAS2R16		
V3SVHSHC_4799810	DNAJB4		2.417E-05					DNAJB4		
V3SVHSHC_6084896	OR11L1		2.571E-05					OR11L1		
V3SVHSHC_5296394	OR4D11		2.596E-05					OR4D11		
V3SVHSHC_7763540	SNUPN		2.602E-05					SNUPN		
V3SVHSHC_7374371	DOCK1		2.634E-05					DOCK1		
V3SVHSHC_10112678	KIAA1147		2.635E-05					KIAA1147		
V3SVHSHC_10403309	SLPI		2.650E-05					SLPI		
V3SVHSHC_10212998	RAB23		2.739E-05					RAB23		
V3SVHSHC_6505250	TMEM255A		2.817E-05					TMEM255A		
V3SVHSHC_7656290	SLC17A1		2.852E-05					SLC17A1		
V3SVHSHC_6497165	SNTG2		2.989E-05					SNTG2		
V3SVHSHC_7200065	CLEC2L		3.251E-05					CLEC2L		
V3SVHSHC_8263523	B4GALNT4		3.305E-05	1.207E-27				B4GALNT4	B4GALNT4	
V3SVHSHC_8515742	INPP4A		3.314E-05					INPP4A		
V3SVHSHC_6566399	DHCR24		3.380E-05					DHCR24		
V3SVHSHC_8795417	TMEM177		3.582E-05					TMEM177		
V3SVHSHC_6897389	LOC100130705		3.609E-05					LOC100130705		
V3SVHSHC_7949231	CREBZF		3.807E-05					CREBZF		
V3SVHSHC_5426051	CSTF2T		3.976E-05					CSTF2T		
V3SVHSHC_8418161	PXK		4.268E-05					PXK		
V3SVHSHC_7730243	MCTP1		4.393E-05					MCTP1		
V3SVHSHC_7426643	ACVR1B		4.563E-05					ACVR1B		
V3SVHSHC_6701336	COL13A1		4.603E-05					COL13A1		
V3SVHSHC_10031894	PCDHGA1		4.620E-05					PCDHGA1		
V3SVHSHC_10191053	COX7A2L		4.658E-05					COX7A2L		
V3SVHSHC_9646190	IL22		4.963E-05					IL22		
V3SVHSHC_4830698	ST14		5.034E-05					ST14		

V3SVHSHC_5061698	HBG1		5.063E-05					HBG1		
V3SVHSHC_9583754	TSPY3		5.264E-05					TSPY3		
V3SVHSHC_10789376	RNMTL1		5.783E-05					RNMTL1		
V3SVHSHC_9374600	CSTF1		6.713E-05					CSTF1		
V3SVHSHC_8725127	TIMP3		6.801E-05					TIMP3		
V3SVHSHC_10478648	PSMA5		6.802E-05					PSMA5		
V3SVHSHC_10262564	SMC3		6.999E-05					SMC3		
V3SVHSHC_9643715	SPTLC3		7.399E-05					SPTLC3		
V3SVHSHC_6653684	PTRHD1		8.163E-05					PTRHD1		
V3SVHSHC_9691235	CAPZB		9.680E-05					CAPZB		
V3SVHSHC_10785680	LPL		1.011E-04					LPL		
V3SVHSHC_9274511	ZNF282		1.016E-04					ZNF282		
V3SVHSHC_8015396	ENDOG		1.020E-04					ENDOG		
V3SVHSHC_6521288	ZNF880		1.083E-04					ZNF880		
V3SVHSHC_7494689	RANBP6		1.089E-04					RANBP6		
V3SVHSHC_9435353	MRE11A		1.105E-04					MRE11A		
V3SVHSHC_5206370	Ms4a15		1.106E-04					Ms4a15		
V3SVHSHC_6557687	GNLY		1.156E-04					GNLY		
V3SVHSHC_4818521	LOC101060588		1.204E-04					LOC10106<br>0588		
V3SVHSHC_7926461	PREPL		1.227E-04					PREPL		
V3SVHSHC_10477361	C1orf109		1.274E-04					C1orf109		
V3SVHSHC_8885936	KLHL4		1.286E-04					KLHL4		
V3SVHSHC_10085420	GCHFR		1.293E-04					GCHFR		
V3SVHSHC_9834521	C5orf52		1.297E-04					C5orf52		
V3SVHSHC_5537096	KLHDC7A		1.327E-04					KLHDC7A		
V3SVHSHC_7072619	UBQLN4		1.365E-04					UBQLN4		
V3SVHSHC_4861388	GBE1		1.376E-04					GBE1		
V3SVHSHC_8507888	TDGF1		1.439E-04					TDGF1		
V3SVHSHC_10375061	TAC4		1.545E-04					TAC4		
V3SVHSHC_9212306	KIF27		1.585E-04					KIF27		
V3SVHSHC_9585833	CRYGA		1.653E-04					CRYGA		
V3SVHSHC_7501553	CDHR1		1.656E-04					CDHR1		
V3SVHSHC_10578803	NOM1		1.710E-04					NOM1		
V3SVHSHC_10248968	SUMO4		1.730E-04					SUMO4		
V3SVHSHC_6064667	SPX		1.746E-04					SPX		
V3SVHSHC_7428194	FKTN		1.868E-04					FKTN		
V3SVHSHC_8559863	RABGGTB		1.909E-04					RABGGTB		
V3SVHSHC_4996391	KLHL29		1.928E-04					KLHL29		
V3SVHSHC_9093770	ZNF324		1.976E-04					ZNF324		
V3SVHSHC_6268706	ZSCAN4		2.010E-04					ZSCAN4		
V3SVHSHC_9350675	KCNB2		2.041E-04					KCNB2		
V3SVHSHC_9476900	TVP23C		2.162E-04					TVP23C		
V3SVHSHC_6321242	ZNF688		2.898E-04	2.000E-02				ZNF688		
V3SVHSHC_6864884	TMPRSS2		3.029E-04					TMPRSS2		
V3SVHSHC_6514457	MESP2		3.059E-04					MESP2		
V3SVHSHC_6774629	MX2		3.071E-04					MX2		
V3SVHSHC_8029124	CLEC2A		3.083E-04					CLEC2A		
V3SVHSHC_4640288	BAG5		3.170E-04					BAG5		
V3SVHSHC_10854320	HERPUD1		3.321E-04					HERPUD1		
V3SVHSHC_9197390	REST		3.383E-04					REST		
V3SVHSHC_8114495	ZNRF2		3.509E-04					ZNRF2		
V3SVHSHC_6976886	CIB3		3.756E-04					CIB3		
V3SVHSHC_6239006	ERICH2		3.794E-04					ERICH2		

V3SVHSHC_7006553	LOC100505478		4.626E-04					LOC10050 5478		
V3SVHSHC_8131622	RAB3IL1		4.800E-04					RAB3IL1		
V3SVHSHC_9051365	KIAA1432		4.965E-04					KIAA1432		
V3SVHSHC_6209339	ODF3L1		4.998E-04					ODF3L1		
V3SVHSHC_10428620	TNFAIP6		5.165E-04					TNFAIP6		
V3SVHSHC_6710807	VWA2		5.248E-04					VWA2		
V3SVHSHC_7607780	RAPSN		5.263E-04					RAPSN		
V3SVHSHC_9747929	PNLDC1		5.685E-04					PNLDC1		
V3SVHSHC_6091496	MPHOSPH8		6.031E-04					MPHOSP H8		
V3SVHSHC_4974281	ALDH3B1		6.061E-04					ALDH3B1		
V3SVHSHC_7193036	KIF11		6.163E-04					KIF11		
V3SVHSHC_6558116	MLPH		6.567E-04					MLPH		
V3SVHSHC_5021141	BAIAP2		6.708E-04					BAIAP2		
V3SVHSHC_10140662	OR8K3		7.004E-04					OR8K3		
V3SVHSHC_10307411	ROBO3		7.012E-04					ROBO3		
V3SVHSHC_8684108	LEKR1		7.091E-04					LEKR1		
V3SVHSHC_7015859	PCDHGA1		7.176E-04					PCDHGA1		
V3SVHSHC_9838283	Ptpmt1		7.477E-04					Ptpmt1		
V3SVHSHC_4866932	KCNE4		7.619E-04					KCNE4		
V3SVHSHC_6640517	CHRNB2		7.839E-04							
V3SVHSHC_8429513	GPR64		8.642E-04							
V3SVHSHC_7930157	KLHL20		9.416E-04							
V3SVHSHC_8178152	TCEA3		9.647E-04							
V3SVHSHC_5200529	ATP2A2		9.989E-04							
V3SVHSHC_10125680	TSPAN11		1.108E-03							
V3SVHSHC_9235142	ZNF678		1.151E-03							
V3SVHSHC_6680150	HSF2		1.197E-03							
V3SVHSHC_8084696	RBPMS		1.264E-03							
V3SVHSHC_7122977	CNGA3		1.273E-03							
V3SVHSHC_7969658	HIF3A		1.280E-03							
V3SVHSHC_7029917	ZNF300		1.298E-03							
V3SVHSHC_7354736	CCL18		1.312E-03							
V3SVHSHC_7445618	PTGS2		1.322E-03							
V3SVHSHC_9250025	MEGF8		1.344E-03							
V3SVHSHC_6500993	INTS1		1.382E-03							
V3SVHSHC_5971574	POLR2L		1.417E-03							
V3SVHSHC_10369715	C2ORF16		1.419E-03							
V3SVHSHC_9217586	FMO2		1.421E-03							
V3SVHSHC_5305403	MRGPRX4		1.428E-03		2.198E-06					MRGPRX4
V3SVHSHC_6081629	EVX2		1.437E-03							
V3SVHSHC_8297942	TATDN2		1.468E-03							
V3SVHSHC_9484391	DDR1		1.606E-03							
V3SVHSHC_7287152	EFHC1		1.631E-03							
V3SVHSHC_7824854	ZBTB24		1.634E-03							
V3SVHSHC_6077669	RAD18		1.667E-03							
V3SVHSHC_8336123	UQCRHL		1.692E-03							
V3SVHSHC_8398427	MED20		1.745E-03							
V3SVHSHC_7908707	FCN1		1.841E-03							
V3SVHSHC_7873892	KLRC4		1.861E-03							
V3SVHSHC_5926265	LOC101929564		1.987E-03							
V3SVHSHC_10796207	BMP1		2.077E-03							
V3SVHSHC_8425817	EBP		2.180E-03							
V3SVHSHC_9478748	PRSS36		2.185E-03							

V3SVHSHC_4660352	NLRP6		2.214E-03							
V3SVHSHC_9090701	NAA30		2.256E-03							
V3SVHSHC_8989721	MMGT1		2.275E-03							
V3SVHSHC_8351963	ANKRD61		2.349E-03							
V3SVHSHC_10377272	ACCS		2.394E-03							
V3SVHSHC_7556894	CDC14B		2.453E-03							
V3SVHSHC_9454130	PRAMEF20		2.495E-03							
V3SVHSHC_6962927	HEPH		2.502E-03							
V3SVHSHC_8748161	LOC100132174		2.591E-03							
V3SVHSHC_9468749	C1orf159		2.620E-03	4.463E-01						
V3SVHSHC_6649328	PCDHA4		2.740E-03							
V3SVHSHC_9424034	CACNA2D2		2.767E-03							
V3SVHSHC_7240919	DUSP2		2.847E-03		3.558E-10					DUSP2
V3SVHSHC_4816739	DFNA5		2.962E-03							
V3SVHSHC_7492280	NONO		2.980E-03							
V3SVHSHC_8198876	SLC5A7		3.020E-03							
V3SVHSHC_6038399	LOC100653225		3.114E-03							
V3SVHSHC_8922566	TMEM110-MUSTN1		3.128E-03							
V3SVHSHC_9216464	MARCH1		3.164E-03							
V3SVHSHC_4853435	EPC1		3.279E-03							
V3SVHSHC_5385197	TVP23B		3.401E-03							
V3SVHSHC_7096478	STAB1		3.410E-03							
V3SVHSHC_6149741	Cul9		3.516E-03							
V3SVHSHC_7882010	UNC5CL		3.598E-03							
V3SVHSHC_7857689	TRH		3.690E-03							
V3SVHSHC_5641607	UVRAG		3.764E-03							
V3SVHSHC_8018531	ANAPC10		3.766E-03							
V3SVHSHC_10556858	TWISTNB		3.776E-03							
V3SVHSHC_7311506	ZNF687		3.964E-03							
V3SVHSHC_9685493	MRPS18C		4.014E-03							
V3SVHSHC_10025690	FGD5		4.126E-03							
V3SVHSHC_6790271	ATG9A		4.138E-03							
V3SVHSHC_9380408	MTFMT		4.153E-03							
V3SVHSHC_7400573	FANCL		4.202E-03							
V3SVHSHC_9989027	C1QTNF6		4.241E-03							
V3SVHSHC_10794788	ABHD17C		4.445E-03							
V3SVHSHC_9627578	MMRN2		4.478E-03							
V3SVHSHC_7984046	STRBP		4.490E-03							
V3SVHSHC_7208810	OR56A1		4.770E-03							
V3SVHSHC_10220555	ARMC8		5.143E-03							
V3SVHSHC_10237055	HHLA2		5.176E-03							
V3SVHSHC_5855051	IMPG2		5.580E-03							
V3SVHSHC_10678892	RDH13		6.049E-03							
V3SVHSHC_7829375	SLAMF8		6.071E-03							
V3SVHSHC_9820958	SERINC3		6.178E-03							
V3SVHSHC_6284381	SRSF12		6.491E-03							
V3SVHSHC_6237323	COPS4		6.513E-03							
V3SVHSHC_7040477	BPIFA3		6.731E-03							
V3SVHSHC_5315468	PLEKHB1		7.166E-03							
V3SVHSHC_5511092	DCUN1D1		7.613E-03							
V3SVHSHC_10686548	FAM129A		7.697E-03							
V3SVHSHC_4637912	LOC642131		7.818E-03							
V3SVHSHC_8613224	APBA2		8.203E-03							
V3SVHSHC_8565077	HCN3		8.532E-03							
V3SVHSHC_6888941	C3orf72		8.640E-03							

V3SVHSHC_6233693	C9ORF117		8.906E-03							
V3SVHSHC_10566989	PPIB		9.039E-03							
V3SVHSHC_9392684	ZNF502		9.080E-03							
V3SVHSHC_10854386	LOC100287651		9.419E-03							
V3SVHSHC_8812775	OR8B12		9.469E-03							
V3SVHSHC_9274445	RPS16		1.045E-02							
V3SVHSHC_9530888	PPIB		1.053E-02							
V3SVHSHC_8033216	TRPM5		1.157E-02							
V3SVHSHC_10202768	HHIPL2		1.157E-02							
V3SVHSHC_7932830	Eif6		1.177E-02	1.851E-01						
V3SVHSHC_8233031	CORO1B		1.197E-02							
V3SVHSHC_8255504	TMEM115		1.223E-02							
V3SVHSHC_5285009	MLH3		1.268E-02							
V3SVHSHC_10197653	ZNF747		1.270E-02							
V3SVHSHC_8729351	TTC7A		1.286E-02							
V3SVHSHC_10210490	SESN1		1.305E-02							
V3SVHSHC_7779743	C10ORF129		1.315E-02							
V3SVHSHC_9244976	EMR1		1.406E-02							
V3SVHSHC_8420867	TOMM5		1.441E-02							
V3SVHSHC_9807197	SEPHS1		1.452E-02							
V3SVHSHC_8039816	GAL3ST1		1.555E-02							
V3SVHSHC_6213959	SNX13		1.563E-02							
V3SVHSHC_8714006	TM4SF19		1.591E-02							
V3SVHSHC_5766281	CLRN3		1.617E-02							
V3SVHSHC_4720577	RBM11		1.630E-02							
V3SVHSHC_5789513	PRRT1		1.643E-02							
V3SVHSHC_9260849	SPATA31A5		1.654E-02							
V3SVHSHC_5380181	FANCB		1.687E-02							
V3SVHSHC_6742190	SERPINA5		1.712E-02							
V3SVHSHC_8879765	AKR7A2		1.726E-02							
V3SVHSHC_9982526	TYW3		1.799E-02							
V3SVHSHC_9987245	SEMA3A		1.896E-02							
V3SVHSHC_4665632	HIST1H2AG		1.948E-02	1.088E-11					HIST1H2A G	
V3SVHSHC_4682132	KRT72		2.016E-02		5.760E-05					KRT72
V3SVHSHC_7137299	TTBK1		2.059E-02		2.610E-03					
V3SVHSHC_10674536	SLC12A1		2.119E-02							
V3SVHSHC_8931476	AMDHD2		2.137E-02							
V3SVHSHC_8839967	SLCO6A1		2.162E-02							
V3SVHSHC_10220951	ESPN		2.250E-02							
V3SVHSHC_6573923	C3orf35		2.255E-02							
V3SVHSHC_7145483	NF2		2.272E-02							
V3SVHSHC_9698858	SH3TC1		2.312E-02							
V3SVHSHC_5407736	PRR18		2.316E-02							
V3SVHSHC_5477663	NID2		2.333E-02							
V3SVHSHC_5286164	RTP3		2.342E-02							
V3SVHSHC_7165580	PIAS3		2.478E-02							
V3SVHSHC_10091525	MAPRE3		2.486E-02							
V3SVHSHC_8120237	NARS		2.493E-02							
V3SVHSHC_4875941	SLC6A18		2.542E-02							
V3SVHSHC_9266360	SLC39A10		2.593E-02							
V3SVHSHC_8489177	NTF4		2.619E-02							
V3SVHSHC_10352588	DOK7		2.635E-02							
V3SVHSHC_8567981	ZNF483		2.644E-02							
V3SVHSHC_5118689	GEMIN7		2.679E-02							

V3SVHSHC_8023547	NUP93		2.760E-02							
V3SVHSHC_6467663	ESR1		2.800E-02							
V3SVHSHC_8965433	FCN1		2.897E-02							
V3SVHSHC_7503566	PRSS1		3.002E-02							
V3SVHSHC_7513961	UTP23		3.061E-02		5.913E-11					UTP23
V3SVHSHC_9534452	PRPF40B		3.143E-02							
V3SVHSHC_9992096	GRHL2		3.280E-02							
V3SVHSHC_9296357	PNPLA8		3.329E-02							
V3SVHSHC_9019124	PNLIPRP2		3.329E-02							
V3SVHSHC_7369817	ETHE1		3.456E-02							
V3SVHSHC_9660347	UGT1A1		3.505E-02							
V3SVHSHC_5535182	BANF2		3.528E-02							
V3SVHSHC_8313980	ICOSLG		3.554E-02							
V3SVHSHC_6178286	Haus4		3.585E-02							
V3SVHSHC_9575108	NDUFV2		4.102E-02							
V3SVHSHC_4667876	SYNJ2		4.148E-02							
V3SVHSHC_9284510	NANOGNB		4.272E-02							
V3SVHSHC_7027640	KRTAP10-7		4.325E-02							
V3SVHSHC_9341468	STARD3NL		4.329E-02							
V3SVHSHC_9211745	Tmem89		4.335E-02							
V3SVHSHC_5159114	NUDT15		4.419E-02							
V3SVHSHC_5918972	UQCRH		4.562E-02							
V3SVHSHC_5647646	TTC39B		4.898E-02							
V3SVHSHC_8344967	OXCT1		5.049E-02							
V3SVHSHC_7736513	SNAPC4		5.149E-02							
V3SVHSHC_5065922	TSPYL5		5.276E-02							
V3SVHSHC_5004641	SLC2A12		5.814E-02							
V3SVHSHC_9220424	CCL3L3		5.820E-02							
V3SVHSHC_4823108	DRD5		5.848E-02							
V3SVHSHC_8387735	URB1		6.046E-02							
V3SVHSHC_6818882	PREPL		6.154E-02							
V3SVHSHC_6708662	LOC100505498		6.186E-02							
V3SVHSHC_6718628	DCTPP1		6.190E-02							
V3SVHSHC_5578016	CTNNA1		6.389E-02		2.361E-01					
V3SVHSHC_9499439	HSPB8		6.942E-02							
V3SVHSHC_7115024	MCMBP		6.944E-02							
V3SVHSHC_5554619	Adat3		7.148E-02							
V3SVHSHC_8736875	PRR3		7.461E-02							
V3SVHSHC_7032293	SCG2		7.604E-02							
V3SVHSHC_9745091	NOX5		7.766E-02							
V3SVHSHC_8424761	TRIP6		7.911E-02							
V3SVHSHC_6219239	TK2		8.005E-02							
V3SVHSHC_6820268	LMBR1		8.028E-02	5.928E-09					LMBR1	
V3SVHSHC_6000647	TRIM51		8.040E-02							
V3SVHSHC_6688862	SARDH		8.118E-02							
V3SVHSHC_8868809	TICRR		8.462E-02							
V3SVHSHC_6114332	KCNIP1		8.653E-02							
V3SVHSHC_9886166	MALT1		8.828E-02							
V3SVHSHC_9152642	FXYD5		8.864E-02							
V3SVHSHC_6352790	DFFA		8.980E-02							
V3SVHSHC_6310121	LOC646862		9.010E-02							
V3SVHSHC_10200359	LINS		9.202E-02							
V3SVHSHC_5092190	KLF9		9.519E-02							
V3SVHSHC_8354207	EEF2		9.560E-02	5.414E-02						
V3SVHSHC_4894916	PAEP		9.660E-02							

V3SVHSHC_5416184	NDUFS3		9.662E-02							
V3SVHSHC_7080011	ZNF407		9.888E-02							
V3SVHSHC_8587913	CRIP3		9.894E-02							
V3SVHSHC_7340876	GPBP1		1.027E-01		1.282E-02					
V3SVHSHC_5794133	PLGRKT		1.029E-01							
V3SVHSHC_5879735	CD244		1.037E-01							
V3SVHSHC_9844421	NAGPA		1.067E-01							
V3SVHSHC_5536865	POU6F1		1.087E-01							
V3SVHSHC_9062090	FHDC1		1.124E-01							
V3SVHSHC_6249302	MYH10		1.129E-01							
V3SVHSHC_8143370	FOLR2		1.154E-01							
V3SVHSHC_7175414	STAMBP		1.207E-01							
V3SVHSHC_10605401	LOC100506571		1.221E-01							
V3SVHSHC_7140665	HIST1H3J		1.224E-01							
V3SVHSHC_10510427	IL7		1.269E-01							
V3SVHSHC_8812841	LOC101927085		1.318E-01							
V3SVHSHC_5208680	MYB		1.319E-01							
V3SVHSHC_7345364	GAPT		1.344E-01							
V3SVHSHC_10551875	ZNF280C		1.370E-01							
V3SVHSHC_7022789	TXLNB		1.381E-01							
V3SVHSHC_10618502	RECQL		1.409E-01							
V3SVHSHC_6246134	SH3PXD2A		1.410E-01							
V3SVHSHC_9464261	DPY19L2		1.433E-01		2.042E-04					DPY19L2
V3SVHSHC_7020050	PDZD2		1.446E-01							
V3SVHSHC_8804888	MECR		1.480E-01							
V3SVHSHC_5376287	ZNF605		1.480E-01							
V3SVHSHC_5648240	AP2M1		1.485E-01							
V3SVHSHC_5106842	TIMM17A		1.504E-01							
V3SVHSHC_5622698	PCDH18		1.516E-01							
V3SVHSHC_8466011	PFDN4		1.583E-01		2.575E-01					
V3SVHSHC_9242237	HLA-DRB4		1.607E-01							
V3SVHSHC_6707672	ATP11C		1.639E-01							
V3SVHSHC_9739085	EBP		1.671E-01							
V3SVHSHC_6436907	COL4A1		1.722E-01							
V3SVHSHC_6528746	PATL1		1.736E-01							
V3SVHSHC_5295074	PPM1H		1.737E-01							
V3SVHSHC_7539338	YEATS4		1.749E-01							
V3SVHSHC_8795186	TMEM106C		1.755E-01							
V3SVHSHC_9483434	FAM185A		1.767E-01		2.624E-01					
V3SVHSHC_7586594	STON1		1.805E-01							
V3SVHSHC_6498782	ZNF234		1.818E-01							
V3SVHSHC_5057606	ZNF532		1.876E-01							
V3SVHSHC_10847918	TTLL10		1.896E-01							
V3SVHSHC_9033908	ZCCHC6		1.896E-01							
V3SVHSHC_8294246	PCDHA6		1.940E-01	9.291E-05					PCDHA6	
V3SVHSHC_5160137	YAP1		1.999E-01							
V3SVHSHC_9449147	DDX31		2.033E-01							
V3SVHSHC_7547060	PPP2R1A		2.107E-01	1.111E-01						
V3SVHSHC_7922336	NCMAP		2.129E-01							
V3SVHSHC_7507097	DNAJB12		2.157E-01							
V3SVHSHC_9600089	PDLIM2		2.170E-01							
V3SVHSHC_9334142	JTB		2.178E-01	2.786E-02						
V3SVHSHC_6029885	FGFR1		2.235E-01	2.840E-01						
V3SVHSHC_8099447	WDFY3		2.239E-01							
V3SVHSHC_7210955	MRPS26		2.286E-01							

V3SVHSHC_6844886	PPIB		2.351E-01	1.251E-01	4.269E-01					
V3SVHSHC_7863893	GZMM		2.365E-01							
V3SVHSHC_5892869	AURKA		2.366E-01							
V3SVHSHC_7816901	CENPE		2.399E-01	4.091E-02						
V3SVHSHC_9617084	RPL11		2.482E-01							
V3SVHSHC_7285139	OGFRL1		2.522E-01							
V3SVHSHC_9552965	WDR54		2.549E-01							
V3SVHSHC_8130830	TLR4		2.550E-01							
V3SVHSHC_10010246	PPARD		2.560E-01							
V3SVHSHC_6577124	NDUFA3		2.565E-01							
V3SVHSHC_10148681	ZNF625		2.627E-01							
V3SVHSHC_5193599	RAE1		2.764E-01		2.115E-01					
V3SVHSHC_9630614	CTSS		2.780E-01							
V3SVHSHC_4845350	ABCG5		2.790E-01		5.888E-16					ABCG5
V3SVHSHC_5377310	NIP7		2.818E-01	9.163E-02						
V3SVHSHC_9243260	WDR62		2.829E-01							
V3SVHSHC_8861945	NKX2-6		2.840E-01							
V3SVHSHC_6796706	IKZF4		2.845E-01							
V3SVHSHC_8893097	SCAP		2.886E-01							
V3SVHSHC_10743737	ADAM10		2.888E-01							
V3SVHSHC_6618341	GINS3		2.917E-01							
V3SVHSHC_9100634	LOC101928049		3.040E-01							
V3SVHSHC_6336125	FUZ		3.048E-01							
V3SVHSHC_5318273	C11orf44		3.051E-01							
V3SVHSHC_10254446	ITPK1		3.064E-01							
V3SVHSHC_7328765	GTSE1		3.095E-01							
V3SVHSHC_7689059	LRRC3C		3.114E-01							
V3SVHSHC_9450731	Eif6		3.140E-01							
V3SVHSHC_8417996	OR2AG2		3.168E-01		4.776E-01					
V3SVHSHC_10843760	H3F3B		3.271E-01							
V3SVHSHC_5236334	PAK3		3.279E-01							
V3SVHSHC_8069582	PDE6G		3.309E-01							
V3SVHSHC_10634804	ACTB		3.340E-01		4.800E-01					
V3SVHSHC_8688695	POLG2		3.357E-01							
V3SVHSHC_10072385	TERF1		3.370E-01							
V3SVHSHC_10634474	Unc119b		3.433E-01							
V3SVHSHC_5006654	OCM		3.514E-01	2.533E-01	2.461E-01					
V3SVHSHC_6906233	NECAB1		3.528E-01	3.931E-01						
V3SVHSHC_5551814	YEATS4		3.551E-01							
V3SVHSHC_9505841	CSDE1		3.566E-01							
V3SVHSHC_8679422	OR2K2		3.574E-01							
V3SVHSHC_10457693	CCDC50		3.578E-01							
V3SVHSHC_6857987	RNASEH2C		3.601E-01							
V3SVHSHC_7409846	C2orf69		3.628E-01							
V3SVHSHC_9648170	SETD1A		3.629E-01							
V3SVHSHC_10042091	EIF2S2		3.637E-01							
V3SVHSHC_8807000	LEPR		3.652E-01							
V3SVHSHC_9730538	MEIG1		3.712E-01		2.125E-19					MEIG1
V3SVHSHC_9767498	ACTB		3.714E-01							
V3SVHSHC_8863925	CHRND		3.746E-01							
V3SVHSHC_8965961	PFDN4		3.788E-01	4.931E-01	4.668E-01					
V3SVHSHC_7798025	KIF3C		3.862E-01							
V3SVHSHC_6268640	MNAT1		3.869E-01							
V3SVHSHC_6726284	SOWAHC		3.874E-01							
V3SVHSHC_7693877	DPP3		3.884E-01							

V3SVHSHC_8155778	SPAG17		3.896E-01							
V3SVHSHC_4830764	SLC2A11		4.060E-01		1.030E-25					SLC2A11
V3SVHSHC_7766345	LOC101927645		4.067E-01							
V3SVHSHC_9327113	SNAI1		4.102E-01	1.675E-03						
V3SVHSHC_6538613	ATRN		4.168E-01							
V3SVHSHC_8215838	Minos1		4.203E-01							
V3SVHSHC_7335761	RHOBTB3		4.213E-01		4.822E-07					RHOBTB3
V3SVHSHC_9422417	STPG2		4.280E-01							
V3SVHSHC_6910721	FANCD2		4.389E-01							
V3SVHSHC_7343516	PTPN3		4.410E-01							
V3SVHSHC_6347213	KLHL12		4.563E-01							
V3SVHSHC_9512441	ZNF146		4.589E-01							
V3SVHSHC_9726743	ARMS2		4.673E-01							
V3SVHSHC_5086349	MAPK8IP1		4.742E-01							
V3SVHSHC_8473766	SLC45A3		4.846E-01							
V3SVHSHC_10551380	SIRPG		4.888E-01							
V3SVHSHC_7245539	MANSC4			2.785E-57					MANSC4	
V3SVHSHC_5739254	KCNN2			5.522E-57					KCNN2	
V3SVHSHC_8937911	TRMT6			3.680E-54	1.633E-13				TRMT6	TRMT6
V3SVHSHC_5999987	SRSF3			5.686E-54					SRSF3	
V3SVHSHC_8095355	FLT4			1.031E-53					FLT4	
V3SVHSHC_9536333	KCNG3			7.867E-53					KCNG3	
V3SVHSHC_9804128	CRYAB			1.144E-52					CRYAB	
V3SVHSHC_9585635	ENPP3			8.805E-52					ENPP3	
V3SVHSHC_9611771	DERL1			1.699E-51					DERL1	
V3SVHSHC_6791360	LOC101928356			4.827E-51					LOC10192 8356	
V3SVHSHC_8386316	OR2M7			1.814E-50					OR2M7	
V3SVHSHC_10553459	RPS4X			9.087E-50					RPS4X	
V3SVHSHC_9147428	STOML2			1.104E-48					STOML2	
V3SVHSHC_9123932	TOMM70A			2.172E-48					TOMM70A	
V3SVHSHC_7613852	STMN3			2.306E-47					STMN3	
V3SVHSHC_7377242	DNAJB8			3.164E-47					DNAJB8	
V3SVHSHC_9359024	GCSH			1.037E-46					GCSH	
V3SVHSHC_10305035	DMBT1			1.472E-46					DMBT1	
V3SVHSHC_5236598	FCGR3B			4.973E-46					FCGR3B	
V3SVHSHC_10539005	SLC25A16			5.537E-46					SLC25A16	
V3SVHSHC_5846405	LMAN2L			8.118E-46					LMAN2L	
V3SVHSHC_5661242	ENPP3			1.323E-45					ENPP3	
V3SVHSHC_10209302	LSM1			9.369E-45					LSM1	
V3SVHSHC_8365361	BIRC7			2.681E-43					BIRC7	
V3SVHSHC_9610583	AMIGO1			3.055E-43					AMIGO1	
V3SVHSHC_9542108	MED18			3.772E-43					MED18	
V3SVHSHC_9240455	LRRC42			4.031E-43					LRRC42	
V3SVHSHC_7561811	TGIF1			4.929E-43					TGIF1	
V3SVHSHC_6720278	ARHGAP23			5.278E-43					ARHGAP2 3	
V3SVHSHC_9967676	OR2F2			1.836E-42					OR2F2	
V3SVHSHC_9945962	PEX3			1.199E-41					PEX3	
V3SVHSHC_10401362	LDHD			5.150E-41					LDHD	
V3SVHSHC_6240194	THEMIS			5.406E-41					THEMIS	
V3SVHSHC_6744632	FBXO46			7.104E-41					FBXO46	
V3SVHSHC_7774595	STARD4			2.356E-40					STARD4	
V3SVHSHC_6134627	SLC25A12			2.595E-40					SLC25A12	
V3SVHSHC_6515447	C14ORF1			5.184E-40					C14ORF1	

V3SVHSHC_7116179	SFTPC			9.381E-40					SFTPC		
V3SVHSHC_10769180	BIRC6			1.782E-39					BIRC6		
V3SVHSHC_6259301	LDB1			1.920E-39					LDB1		
V3SVHSHC_7231976	ZNF248			2.533E-39					ZNF248		
V3SVHSHC_8362358	CEACAM19			4.870E-39					CEACAM1<br>9		
V3SVHSHC_5596298	BBS10			8.981E-39					BBS10		
V3SVHSHC_9210788	SRPR			9.489E-39					SRPR		
V3SVHSHC_6579830	HNRNPA3			1.300E-38					HNRNPA3		
V3SVHSHC_7427468	DYNLT3			1.391E-38					DYNLT3		
V3SVHSHC_8150564	DPF1			8.075E-38					DPF1		
V3SVHSHC_7016519	CYB561D2			1.412E-37					CYB561D2		
V3SVHSHC_8665727	FEV			1.741E-37					FEV		
V3SVHSHC_7895243	IL32			4.867E-37					IL32		
V3SVHSHC_10483664	SHOX			5.890E-37					SHOX		
V3SVHSHC_7196930	MEGF11			7.240E-37					MEGF11		
V3SVHSHC_5480138	KIAA1429			8.414E-37					KIAA1429		
V3SVHSHC_6246728	DNMT3L			1.376E-36					DNMT3L		
V3SVHSHC_6525578	LCE2B			1.940E-36					LCE2B		
V3SVHSHC_9696713	PATE3			2.100E-36					PATE3		
V3SVHSHC_4778426	TOB1			5.776E-36					TOB1		
V3SVHSHC_7775453	ARID5B			6.039E-36					ARID5B		
V3SVHSHC_9827492	OTOP2			6.385E-36					OTOP2		
V3SVHSHC_9832112	KCNC3			7.680E-36					KCNC3		
V3SVHSHC_10363709	IKZF1			1.317E-35					IKZF1		
V3SVHSHC_9175214	ZNF174			1.507E-35					ZNF174		
V3SVHSHC_9736379	SIN3B			1.944E-35					SIN3B		
V3SVHSHC_9044600	BCL9			2.222E-35					BCL9		
V3SVHSHC_9824258	LYZL1			2.346E-35					LYZL1		
V3SVHSHC_9506699	UGT1A1			2.569E-35					UGT1A1		
V3SVHSHC_5580689	GALNT16			5.974E-35					GALNT16		
V3SVHSHC_6044834	KRT8			8.623E-35					KRT8		
V3SVHSHC_6222176	UBOX5			9.918E-35					UBOX5		
V3SVHSHC_7295072	UBE2V2			1.085E-34					UBE2V2		
V3SVHSHC_4648538	DCLRE1B			3.170E-34					DCLRE1B		
V3SVHSHC_7606427	CARD16			3.241E-34					CARD16		
V3SVHSHC_8649623	NFKB2			3.580E-34					NFKB2		
V3SVHSHC_5307581	SYT2			3.888E-34					SYT2		
V3SVHSHC_8997608	RASGEF1A			8.485E-34					RASGEF1<br>A		
V3SVHSHC_7072520	IFI27L1			8.634E-34					IFI27L1		
V3SVHSHC_7193333	NACC2			2.187E-33					NACC2		
V3SVHSHC_8105750	MYO5C			2.784E-33					MYO5C		
V3SVHSHC_6849539	TIAF1			2.948E-33					TIAF1		
V3SVHSHC_8624939	TMEM189-UBE2V1			3.037E-33					TMEM189-<br>UBE2V1		
V3SVHSHC_8374007	S100A12			4.978E-33					S100A12		
V3SVHSHC_6757667	ZNF19			6.340E-33					ZNF19		
V3SVHSHC_9983384	MRPL51			6.561E-33					MRPL51		
V3SVHSHC_5497826	ATP2B4			7.880E-33					ATP2B4		
V3SVHSHC_5693846	NPM3			1.135E-32					NPM3		
V3SVHSHC_4938971	Izumo2			1.144E-32					Izumo2		
V3SVHSHC_6133406	Carkd			1.473E-32					Carkd		
V3SVHSHC_9952133	SKI			1.681E-32					SKI		
V3SVHSHC_9062552	CTBP2			2.306E-32					CTBP2		

V3SVHSHC_6345068	GOLGA2			2.756E-32					GOLGA2	
V3SVHSHC_10096277	LOC101060399			3.135E-32					LOC101060399	
V3SVHSHC_8039585	MS4A6A			3.386E-32					MS4A6A	
V3SVHSHC_6653552	FERMT1			3.719E-32					FERMT1	
V3SVHSHC_6124397	ZNF93			4.590E-32	1.948E-03				ZNF93	
V3SVHSHC_9898871	CHST12			5.263E-32					CHST12	
V3SVHSHC_9466010	ATP4A			6.484E-32					ATP4A	
V3SVHSHC_5955866	ZNF396			7.082E-32					ZNF396	
V3SVHSHC_6630980	SLC11A1			1.116E-31	4.239E-01				SLC11A1	
V3SVHSHC_5603393	MIA3			1.315E-31					MIA3	
V3SVHSHC_8003450	PILRB			1.457E-31					PILRB	
V3SVHSHC_9816503	PI16			1.755E-31					PI16	
V3SVHSHC_5689655	AGPAT6			3.505E-31					AGPAT6	
V3SVHSHC_6842675	Cisd2			4.349E-31					Cisd2	
V3SVHSHC_5770736	OR11G2			4.508E-31					OR11G2	
V3SVHSHC_5981672	ZSCAN30			4.966E-31					ZSCAN30	
V3SVHSHC_8150267	BRSK1			5.913E-31					BRSK1	
V3SVHSHC_5995136	LMF2			7.111E-31					LMF2	
V3SVHSHC_9354998	ACSS1			7.485E-31					ACSS1	
V3SVHSHC_10734959	SLAMF6			8.494E-31					SLAMF6	
V3SVHSHC_10798451	DUS4L			1.065E-30					DUS4L	
V3SVHSHC_6746876	RBKS			1.227E-30					RBKS	
V3SVHSHC_9743243	FAM200B			1.289E-30					FAM200B	
V3SVHSHC_10784426	ETNK2			1.514E-30					ETNK2	
V3SVHSHC_8017178	CRTC2			1.543E-30					CRTC2	
V3SVHSHC_7808717	KCNA5			1.635E-30					KCNA5	
V3SVHSHC_9415322	LIMCH1			1.681E-30					LIMCH1	
V3SVHSHC_5822579	AKAP8			2.022E-30					AKAP8	
V3SVHSHC_7002923	GYS1			2.146E-30					GYS1	
V3SVHSHC_5976260	OR51A7			3.077E-30					OR51A7	
V3SVHSHC_6765092	MSH5			3.609E-30					MSH5	
V3SVHSHC_6453143	DACT1			3.615E-30					DACT1	
V3SVHSHC_9286292	AMELX			3.755E-30					AMELX	
V3SVHSHC_8173961	ST6GALNAC6			3.866E-30					ST6GALNAC6	
V3SVHSHC_8475482	MARK4			4.087E-30					MARK4	
V3SVHSHC_5908907	ZNF761			4.173E-30					ZNF761	
V3SVHSHC_5627153	ZNF746			4.366E-30					ZNF746	
V3SVHSHC_10678595	PRDM5			5.672E-30					PRDM5	
V3SVHSHC_8925107	SLC25A26			6.190E-30					SLC25A26	
V3SVHSHC_6111725	C2orf49			7.693E-30					C2orf49	
V3SVHSHC_6201353	DGKE			9.660E-30					DGKE	
V3SVHSHC_9277844	SMARCD2			1.062E-29					SMARCD2	
V3SVHSHC_5605406	PCDHB14			1.340E-29					PCDHB14	
V3SVHSHC_4645172	MEX3D			1.845E-29					MEX3D	
V3SVHSHC_6308867	GMIP			1.996E-29					GMIP	
V3SVHSHC_9529832	ZMPSTE24			2.198E-29					ZMPSTE24	
V3SVHSHC_7263326	PAN2			2.778E-29					PAN2	
V3SVHSHC_8766443	CT45A4			2.825E-29					CT45A4	
V3SVHSHC_5232341	PACSIN2			3.068E-29					PACSIN2	
V3SVHSHC_6261050	HGD			3.779E-29					HGD	
V3SVHSHC_7449347	KIF3B			3.782E-29					KIF3B	
V3SVHSHC_7814690	LAMA1			4.427E-29					LAMA1	

V3SVHSHC_7520000	MRPL48			4.523E-29					MRPL48	
V3SVHSHC_6397703	PPP6R1			4.775E-29					PPP6R1	
V3SVHSHC_5527955	R3HDM2			4.874E-29					R3HDM2	
V3SVHSHC_5394899	FAM196B			4.996E-29					FAM196B	
V3SVHSHC_5915672	DEFB104B			6.005E-29					DEFB104B	
V3SVHSHC_4854359	PCSK5			6.488E-29					PCSK5	
V3SVHSHC_9940715	GOLGA6L1			8.515E-29					GOLGA6L1	
V3SVHSHC_10414595	CDH18			1.677E-28					CDH18	
V3SVHSHC_5078495	CLCNKB			1.834E-28					CLCNKB	
V3SVHSHC_9120665	SULT1A3			1.986E-28					SULT1A3	
V3SVHSHC_8008565	SEC61A2			2.449E-28					SEC61A2	
V3SVHSHC_9481487	C21ORF58			2.715E-28					C21ORF58	
V3SVHSHC_8408921	KLHL4			3.161E-28					KLHL4	
V3SVHSHC_9000809	DESI2			3.746E-28					DESI2	
V3SVHSHC_8571644	OR2T29			3.776E-28					OR2T29	
V3SVHSHC_5297021	CD1B			4.812E-28					CD1B	
V3SVHSHC_5196701	LRP11			5.238E-28					LRP11	
V3SVHSHC_6358961	SSR2			5.347E-28					SSR2	
V3SVHSHC_9689519	CD69			5.372E-28					CD69	
V3SVHSHC_9681698	ARSE			6.453E-28					ARSE	
V3SVHSHC_5555675	MAP3K11			1.020E-27					MAP3K11	
V3SVHSHC_8107037	EYS			1.098E-27					EYS	
V3SVHSHC_6786707	NOL11			1.220E-27					NOL11	
V3SVHSHC_5907422	ATP6AP1			1.221E-27					ATP6AP1	
V3SVHSHC_10597712	MAGEA11			1.268E-27					MAGEA11	
V3SVHSHC_5862311	WIZ			1.327E-27					WIZ	
V3SVHSHC_7790963	PTTG1IP			1.733E-27					PTTG1IP	
V3SVHSHC_10363907	HMGN4			1.768E-27					HMGN4	
V3SVHSHC_5971772	SYNE1			1.777E-27					SYNE1	
V3SVHSHC_7012691	NEUROD6			1.856E-27					NEUROD6	
V3SVHSHC_7948769	SYDE2			2.201E-27					SYDE2	
V3SVHSHC_9459410	LOC101927160			2.234E-27					LOC101927160	
V3SVHSHC_7784759	GPR137C			2.252E-27					GPR137C	
V3SVHSHC_6956525	RAB39B			2.284E-27					RAB39B	
V3SVHSHC_7174820	CYB5B			2.522E-27					CYB5B	
V3SVHSHC_8827361	MYH13			2.554E-27					MYH13	
V3SVHSHC_4879043	RAC2			2.813E-27					RAC2	
V3SVHSHC_7190693	Ripply1			2.827E-27					Ripply1	
V3SVHSHC_8550392	TSPAN4			3.256E-27					TSPAN4	
V3SVHSHC_8853695	INADL			3.427E-27					INADL	
V3SVHSHC_8007047	ULK2			3.447E-27					ULK2	
V3SVHSHC_6332066	ZNF747			3.796E-27					ZNF747	
V3SVHSHC_7677179	ZSCAN16			4.114E-27					ZSCAN16	
V3SVHSHC_7843037	TP63			4.310E-27					TP63	
V3SVHSHC_7546631	PIPOX			4.423E-27					PIPOX	
V3SVHSHC_5777402	PBK			4.526E-27					PBK	
V3SVHSHC_10115648	TCF24			5.502E-27					TCF24	
V3SVHSHC_5175680	CA7			5.645E-27					CA7	
V3SVHSHC_7916528	OR4A15			5.711E-27					OR4A15	
V3SVHSHC_7204685	UXS1			5.862E-27					UXS1	
V3SVHSHC_8364371	EFCAB3			6.188E-27					EFCAB3	
V3SVHSHC_6813668	GIMD1			6.623E-27					GIMD1	

V3SVHSHC_6151325	ACAP1			7.012E-27					ACAP1	
V3SVHSHC_5117138	DMTN			7.311E-27					DMTN	
V3SVHSHC_7832048	RPS18			8.308E-27					RPS18	
V3SVHSHC_5244188	M6PR			1.065E-26					M6PR	
V3SVHSHC_4808291	Wfdc2			1.291E-26					Wfdc2	
V3SVHSHC_6483635	TLR5			1.301E-26					TLR5	
V3SVHSHC_5141294	SDR16C5			1.404E-26					SDR16C5	
V3SVHSHC_10840493	TRIML1			1.438E-26					TRIML1	
V3SVHSHC_7662956	XKR6			1.462E-26					XKR6	
V3SVHSHC_7804691	SLC25A12			1.562E-26					SLC25A12	
V3SVHSHC_5754863	ZNF417			1.835E-26					ZNF417	
V3SVHSHC_8477264	IQCF1			1.982E-26					IQCF1	
V3SVHSHC_5172248	ST6GALNAC5			2.071E-26					ST6GALNAC5	
V3SVHSHC_7293290	TSPAN17			2.180E-26					TSPAN17	
V3SVHSHC_7983122	SBF2			2.225E-26					SBF2	
V3SVHSHC_5108129	GGT2			2.335E-26					GGT2	
V3SVHSHC_4916762	IGJ			2.604E-26					IGJ	
V3SVHSHC_10273553	CHI3L2			2.635E-26					CHI3L2	
V3SVHSHC_6744038	MYT1			3.329E-26					MYT1	
V3SVHSHC_9038132	H1FX			3.352E-26					H1FX	
V3SVHSHC_5338832	TMEM247			3.445E-26					TMEM247	
V3SVHSHC_5907851	ADAMTS9			3.528E-26					ADAMTS9	
V3SVHSHC_9141059	KCNMB4			3.627E-26					KCNMB4	
V3SVHSHC_5360018	Spg11			3.775E-26					Spg11	
V3SVHSHC_8419382	PITPNB			3.810E-26					PITPNB	
V3SVHSHC_9664043	MAPK15			5.181E-26	3.992E-01				MAPK15	
V3SVHSHC_8809376	SFI1			5.436E-26					SFI1	
V3SVHSHC_6046022	DHPS			5.613E-26					DHPS	
V3SVHSHC_9575306	PSMA1			6.851E-26					PSMA1	
V3SVHSHC_5510366	AEBP2			8.657E-26					AEBP2	
V3SVHSHC_8069516	LOC101927594			9.084E-26					LOC101927594	
V3SVHSHC_6146507	OR4K5			9.152E-26					OR4K5	
V3SVHSHC_9123272	AGK			1.115E-25					AGK	
V3SVHSHC_7902569	CLSPN			1.117E-25					CLSPN	
V3SVHSHC_6697541	EXOSC3			1.189E-25					EXOSC3	
V3SVHSHC_7433705	CCDC166			1.211E-25					CCDC166	
V3SVHSHC_10657871	EGFL6			1.241E-25					EGFL6	
V3SVHSHC_10165478	C6			1.368E-25					C6	
V3SVHSHC_5626526	RCBTB1			1.572E-25					RCBTB1	
V3SVHSHC_9418292	RBMY1J			1.706E-25					RBMY1J	
V3SVHSHC_9554384	MCFD2			1.948E-25					MCFD2	
V3SVHSHC_10368461	GYPC			1.971E-25					GYPC	
V3SVHSHC_9795548	S100A7			2.427E-25					S100A7	
V3SVHSHC_8220920	KIAA1377			2.471E-25					KIAA1377	
V3SVHSHC_4772321	STAG1			2.573E-25					STAG1	
V3SVHSHC_7569830	ATRX			2.626E-25					ATRX	
V3SVHSHC_7547621	FSCB			2.848E-25					FSCB	
V3SVHSHC_8262797	CCM2L			3.124E-25					CCM2L	
V3SVHSHC_10714565	MFSD6			3.200E-25					MFSD6	
V3SVHSHC_6236663	STK38L			3.286E-25					STK38L	
V3SVHSHC_7801424	FGL2			3.401E-25					FGL2	
V3SVHSHC_7393049	CD1E			3.639E-25					CD1E	
V3SVHSHC_8869106	OR2A5			3.738E-25					OR2A5	

V3SVHSHC_7098128	COX20			3.753E-25					COX20	
V3SVHSHC_10499603	HSD11B1			3.942E-25					HSD11B1	
V3SVHSHC_8358266	MLXIP			3.974E-25					MLXIP	
V3SVHSHC_8317247	CSH2			4.171E-25					CSH2	
V3SVHSHC_5616296	SMYD2			4.966E-25					SMYD2	
V3SVHSHC_4911647	MSC			5.007E-25					MSC	
V3SVHSHC_8328467	KDM1B			5.042E-25					KDM1B	
V3SVHSHC_6231020	ORAI3			5.573E-25					ORAI3	
V3SVHSHC_7610090	PPP2R5A			5.642E-25					PPP2R5A	
V3SVHSHC_6836372	AVL9			5.944E-25					AVL9	
V3SVHSHC_9612926	Krtap21-1			6.100E-25					Krtap21-1	
V3SVHSHC_5404535	ELMOD2			7.348E-25					ELMOD2	
V3SVHSHC_5266199	SMARCA5			7.690E-25					SMARCA5	
V3SVHSHC_10710737	POMC			8.583E-25					POMC	
V3SVHSHC_8602037	BAGE			8.766E-25					BAGE	
V3SVHSHC_7905572	GCNT3			8.861E-25					GCNT3	
V3SVHSHC_9177194	LCN8			9.119E-25					LCN8	
V3SVHSHC_7394798	LOC100506922			9.456E-25					LOC10050<br>6922	
V3SVHSHC_5864819	Asprv1			9.620E-25					Asprv1	
V3SVHSHC_7502576	SLC25A11			1.065E-24					SLC25A11	
V3SVHSHC_9621308	GPATCH11			1.127E-24					GPATCH1<br>1	
V3SVHSHC_10467527	ADCY4			1.164E-24					ADCY4	
V3SVHSHC_9932498	CYP26A1			1.250E-24					CYP26A1	
V3SVHSHC_8642165	LINGO4			1.321E-24					LINGO4	
V3SVHSHC_10600946	ARF4			1.375E-24					ARF4	
V3SVHSHC_8214320	PCDH11Y			1.392E-24					PCDH11Y	
V3SVHSHC_7177823	ZNF461			1.461E-24					ZNF461	
V3SVHSHC_7540955	CARD18			1.536E-24					CARD18	
V3SVHSHC_9427202	C5orf51			1.608E-24					C5orf51	
V3SVHSHC_4812977	ICOSLG			1.616E-24					ICOSLG	
V3SVHSHC_10437860	HK2			1.916E-24					HK2	
V3SVHSHC_5875808	Rnf217			2.006E-24					Rnf217	
V3SVHSHC_8919695	PRAMEF19			2.210E-24					PRAMEF1<br>9	
V3SVHSHC_10712915	SERPINB13			2.380E-24					SERPINB1<br>3	
V3SVHSHC_7799411	TESK2			2.440E-24					TESK2	
V3SVHSHC_8122481	DERA			2.483E-24					DERA	
V3SVHSHC_7534685	HADHA			2.714E-24					HADHA	
V3SVHSHC_8307842	Tmem237			2.900E-24					Tmem237	
V3SVHSHC_5231978	LYRM9			3.096E-24					LYRM9	
V3SVHSHC_9075653	PBXIP1			3.264E-24					PBXIP1	
V3SVHSHC_6796772	SLC2A14			4.074E-24					SLC2A14	
V3SVHSHC_9153566	MISP			4.583E-24					MISP	
V3SVHSHC_4915244	DAPK2			4.605E-24					DAPK2	
V3SVHSHC_8563988	TCF7L2			4.635E-24					TCF7L2	
V3SVHSHC_10711265	VIPAS39			4.665E-24					VIPAS39	
V3SVHSHC_6749285	SLC17A6			5.140E-24					SLC17A6	
V3SVHSHC_8583128	IL4			5.292E-24					IL4	
V3SVHSHC_6986621	LOC388813			5.461E-24					LOC38881<br>3	
V3SVHSHC_5257619	CTBS			6.064E-24					CTBS	
V3SVHSHC_6086480	A4GNT			6.130E-24	6.790E-02				A4GNT	

V3SVHSHC_9601409	EFHC1			6.367E-24					EFHC1	
V3SVHSHC_6819509	MRRF			6.393E-24					MRRF	
V3SVHSHC_9290945	OPLAH			6.600E-24					OPLAH	
V3SVHSHC_7123802	ALDOB			6.645E-24					ALDOB	
V3SVHSHC_6298175	C10ORF11			7.308E-24					C10ORF11	
V3SVHSHC_6002924	GTF2H3			8.207E-24					GTF2H3	
V3SVHSHC_9857819	MAS1			8.356E-24					MAS1	
V3SVHSHC_7113011	BTLA			8.716E-24					BTLA	
V3SVHSHC_7644047	GPR116			9.071E-24					GPR116	
V3SVHSHC_5272139	MADD			9.089E-24					MADD	
V3SVHSHC_5379092	TMEM45B			9.235E-24					TMEM45B	
V3SVHSHC_10222040	PCSK1			9.320E-24					PCSK1	
V3SVHSHC_6672626	GALNT18			9.685E-24					GALNT18	
V3SVHSHC_6718661	CLEC2L			1.026E-23					CLEC2L	
V3SVHSHC_8974772	TIMM9			1.074E-23					TIMM9	
V3SVHSHC_7816043	LOC101929578			1.159E-23					LOC101929578	
V3SVHSHC_7794494	ALKBH5			1.339E-23					ALKBH5	
V3SVHSHC_4775819	STK36			1.367E-23					STK36	
V3SVHSHC_10628105	SLC4A1			1.438E-23					SLC4A1	
V3SVHSHC_5572670	FAT3			1.518E-23					FAT3	
V3SVHSHC_9438521	OR2F2			1.612E-23					OR2F2	
V3SVHSHC_10351829	CIB3			1.736E-23	4.221E-01				CIB3	
V3SVHSHC_10613915	WFIKKN1			1.899E-23					WFIKKN1	
V3SVHSHC_5762189	RBM43			1.906E-23					RBM43	
V3SVHSHC_10686845	SPSB2			1.959E-23					SPSB2	
V3SVHSHC_6672527	RNF125			2.087E-23					RNF125	
V3SVHSHC_5053118	LAMA5			2.216E-23					LAMA5	
V3SVHSHC_8431559	MAST3			2.227E-23					MAST3	
V3SVHSHC_6481985	ABCC12			2.434E-23					ABCC12	
V3SVHSHC_5212739	ARHGAP19			2.567E-23					ARHGAP19	
V3SVHSHC_9703610	Hbq1			2.609E-23					Hbq1	
V3SVHSHC_9565538	PRIMPOL			2.621E-23	7.416E-02				PRIMPOL	
V3SVHSHC_6506471	CPXM2			2.686E-23					CPXM2	
V3SVHSHC_7568708	AKT2			2.713E-23					AKT2	
V3SVHSHC_6114695	ARHGAP6			2.718E-23					ARHGAP6	
V3SVHSHC_6750209	MTG1			2.774E-23					MTG1	
V3SVHSHC_8476208	KIAA0895			2.909E-23	3.242E-17				KIAA0895	KIAA0895
V3SVHSHC_9876134	CHPT1			2.942E-23					CHPT1	
V3SVHSHC_8193134	NOL10			2.953E-23	3.685E-01				NOL10	
V3SVHSHC_9953123	GSG2			3.528E-23					GSG2	
V3SVHSHC_7080968	INMT			3.567E-23					INMT	
V3SVHSHC_6436016	SUMF1			3.673E-23					SUMF1	
V3SVHSHC_5882243	RPL28			4.128E-23					RPL28	
V3SVHSHC_10440500	NCAM2			4.280E-23					NCAM2	
V3SVHSHC_5438525	SEH1L			4.339E-23	1.635E-11				SEH1L	SEH1L
V3SVHSHC_10815578	ATCAY			4.353E-23					ATCAY	
V3SVHSHC_8502113	SESN2			4.937E-23					SESN2	
V3SVHSHC_6374966	CCDC90B			4.966E-23					CCDC90B	
V3SVHSHC_5194490	CLDND1			5.004E-23					CLDND1	
V3SVHSHC_9884054	ANKRD23			5.028E-23					ANKRD23	
V3SVHSHC_7114067	FADS6			5.101E-23					FADS6	
V3SVHSHC_9164357	AADAC			5.121E-23					AADAC	

V3SVHSHC_5434070	COG3			5.147E-23					COG3	
V3SVHSHC_10744298	ARHGEF25			5.962E-23					ARHGEF25	
V3SVHSHC_7861550	OR56A5			6.135E-23					OR56A5	
V3SVHSHC_6347774	USP43			6.332E-23					USP43	
V3SVHSHC_6099185	SULT4A1			6.875E-23	5.202E-20				SULT4A1	SULT4A1
V3SVHSHC_7858019	TFPT			7.032E-23					TFPT	
V3SVHSHC_10685360	FGF10			7.072E-23					FGF10	
V3SVHSHC_7405292	ARRDC3			7.119E-23					ARRDC3	
V3SVHSHC_5492084	FCHSD2			8.179E-23					FCHSD2	
V3SVHSHC_9132116	LOC101930479			8.727E-23					LOC101930479	
V3SVHSHC_6479939	Ptpmt1			8.935E-23					Ptpmt1	
V3SVHSHC_5401268	MTAP			8.960E-23					MTAP	
V3SVHSHC_8032952	PECR			9.228E-23					PECR	
V3SVHSHC_9705623	SAMSN1			9.710E-23					SAMSN1	
V3SVHSHC_4797071	STEAP3			9.994E-23					STEAP3	
V3SVHSHC_8731760	LRRTM2			1.079E-22					LRRTM2	
V3SVHSHC_8399714	ZNF22			1.090E-22					ZNF22	
V3SVHSHC_7409549	NSRP1			1.143E-22					NSRP1	
V3SVHSHC_6049751	ZFP36			1.203E-22					ZFP36	
V3SVHSHC_7812083	CEBPD			1.260E-22					CEBPD	
V3SVHSHC_9572765	ANKRD20A2			1.313E-22					ANKRD20A2	
V3SVHSHC_5458721	OR5AN1			1.457E-22					OR5AN1	
V3SVHSHC_7792712	C9ORF9			1.533E-22					C9ORF9	
V3SVHSHC_6442484	SLC2A3			1.878E-22					SLC2A3	
V3SVHSHC_8345396	THBS2			2.105E-22					THBS2	
V3SVHSHC_9726809	ASXL3			2.110E-22					ASXL3	
V3SVHSHC_10416146	PRDM13			2.181E-22					PRDM13	
V3SVHSHC_7672229	GTF3A			2.298E-22					GTF3A	
V3SVHSHC_6159080	TRAPPC12			2.309E-22					TRAPPC12	
V3SVHSHC_4855745	PIK3R4			2.345E-22					PIK3R4	
V3SVHSHC_6717341	FAM53B			2.369E-22					FAM53B	
V3SVHSHC_5005961	SH3YL1			2.420E-22					SH3YL1	
V3SVHSHC_10816106	AGL			2.596E-22					AGL	
V3SVHSHC_10387073	TRIML1			2.611E-22					TRIML1	
V3SVHSHC_6436280	FMO5			2.730E-22					FMO5	
V3SVHSHC_8277713	C12orf40			2.765E-22	1.008E-12				C12orf40	C12orf40
V3SVHSHC_6175118	C15orf61			3.125E-22					C15orf61	
V3SVHSHC_6460634	SYF2			3.175E-22					SYF2	
V3SVHSHC_7135649	RLN3			3.377E-22					RLN3	
V3SVHSHC_8400902	PRPH			3.734E-22					PRPH	
V3SVHSHC_9789542	PAGE4			3.903E-22					PAGE4	
V3SVHSHC_4739024	MLLT4			3.908E-22					MLLT4	
V3SVHSHC_8362622	THG1L			4.186E-22					THG1L	
V3SVHSHC_8223461	COA1			4.353E-22					COA1	
V3SVHSHC_6169277	GOT1L1			4.353E-22					GOT1L1	
V3SVHSHC_8129510	SMG1			4.671E-22					SMG1	
V3SVHSHC_7550129	INTS10			5.031E-22					INTS10	
V3SVHSHC_9232568	PPP1R9B			5.278E-22					PPP1R9B	
V3SVHSHC_10125845	TWF2			5.382E-22					TWF2	
V3SVHSHC_9102779	TAF10			5.467E-22					TAF10	
V3SVHSHC_5692625	SLC17A8			5.606E-22					SLC17A8	

V3SVHSHC_8551547	TDG			5.642E-22					TDG	
V3SVHSHC_4929434	ADPRH			5.666E-22					ADPRH	
V3SVHSHC_8929001	COL7A1			5.837E-22					COL7A1	
V3SVHSHC_4885808	RGS13			6.739E-22					RGS13	
V3SVHSHC_10035557	TPPP			6.835E-22					TPPP	
V3SVHSHC_10029980	POTEC			6.835E-22					POTEC	
V3SVHSHC_5518418	NHP2L1			7.186E-22					NHP2L1	
V3SVHSHC_5744996	SULT1C2			7.203E-22					SULT1C2	
V3SVHSHC_8148881	CUL5			7.503E-22					CUL5	
V3SVHSHC_9900752	RTN4RL1			7.621E-22					RTN4RL1	
V3SVHSHC_8063147	CSGALNACT2			7.761E-22					CSGALNACT2	
V3SVHSHC_8500100	FAM156A			8.170E-22					FAM156A	
V3SVHSHC_10719713	CLDN19			9.600E-22					CLDN19	
V3SVHSHC_5953721	METTL21B			9.760E-22					METTL21B	
V3SVHSHC_8589662	CECR1			9.877E-22					CECR1	
V3SVHSHC_6724271	SERPINB2			1.069E-21					SERPINB2	
V3SVHSHC_9333350	PCDHGA10			1.094E-21					PCDHGA10	
V3SVHSHC_6748031	RAB9A			1.142E-21					RAB9A	
V3SVHSHC_6453011	NT5M			1.270E-21					NT5M	
V3SVHSHC_9175082	ZNF19			1.280E-21					ZNF19	
V3SVHSHC_8548280	PRKD3			1.293E-21					PRKD3	
V3SVHSHC_10521713	ZNF410			1.297E-21					ZNF410	
V3SVHSHC_10566527	LOC101927375			1.315E-21					LOC101927375	
V3SVHSHC_5470469	NIPSNAP3A			1.338E-21	3.322E-02				NIPSNAP3A	
V3SVHSHC_7374800	SH3GL3			1.421E-21					SH3GL3	
V3SVHSHC_7307777	ZNF276			1.577E-21					ZNF276	
V3SVHSHC_7423739	SLC38A8			1.677E-21					SLC38A8	
V3SVHSHC_6585440	SYNJ2			1.751E-21					SYNJ2	
V3SVHSHC_5375825	MAPK8IP2			1.809E-21					MAPK8IP2	
V3SVHSHC_8332757	CPA3			2.198E-21					CPA3	
V3SVHSHC_7255571	YIF1A			2.265E-21					YIF1A	
V3SVHSHC_9189998	MAMDC4			2.414E-21					MAMDC4	
V3SVHSHC_6369719	MAPK1			2.507E-21					MAPK1	
V3SVHSHC_8182145	SLC33A1			2.517E-21					SLC33A1	
V3SVHSHC_5423510	Bpifb6			2.719E-21					Bpifb6	
V3SVHSHC_6807398	NMS			2.813E-21					NMS	
V3SVHSHC_6737207	EIF4A1			2.937E-21					EIF4A1	
V3SVHSHC_6198878	CYB5D1			2.947E-21					CYB5D1	
V3SVHSHC_6642233	TEAD2			2.970E-21					TEAD2	
V3SVHSHC_5088725	ZNF250			3.009E-21					ZNF250	
V3SVHSHC_5637977	MRPL53			3.216E-21					MRPL53	
V3SVHSHC_6843995	ACSF3			3.412E-21					ACSF3	
V3SVHSHC_8225870	TIFA			3.420E-21					TIFA	
V3SVHSHC_5848484	TRMT13			3.445E-21					TRMT13	
V3SVHSHC_8568806	ZSWIM8			3.727E-21					ZSWIM8	
V3SVHSHC_4780373	Mageb17			3.740E-21					Mageb17	
V3SVHSHC_9490958	PRKAG2			3.815E-21					PRKAG2	
V3SVHSHC_9585206	HOXA13			4.150E-21					HOXA13	
V3SVHSHC_10653152	POLD3			4.757E-21					POLD3	
V3SVHSHC_5618705	CRNN			4.943E-21					CRNN	

V3SVHSHC_10010015	MICAL1			5.146E-21					MICAL1	
V3SVHSHC_6769349	C9ORF84			5.541E-21					C9ORF84	
V3SVHSHC_6859439	CSNK1A1			5.620E-21					CSNK1A1	
V3SVHSHC_7836998	UGT2B10			5.690E-21					UGT2B10	
V3SVHSHC_6658370	GNGT2			6.579E-21					GNGT2	
V3SVHSHC_4721831	TCEB3			6.729E-21					TCEB3	
V3SVHSHC_6252866	C8orf76			6.768E-21					C8orf76	
V3SVHSHC_7712918	VPS53			7.183E-21					VPS53	
V3SVHSHC_6432980	PARVA			7.549E-21					PARVA	
V3SVHSHC_9912203	IQGAP3			7.772E-21					IQGAP3	
V3SVHSHC_7254713	PSMC3			7.893E-21					PSMC3	
V3SVHSHC_4908314	FAM163A			7.927E-21					FAM163A	
V3SVHSHC_5526866	INPP5A			8.298E-21					INPP5A	
V3SVHSHC_4799843	ZAK			8.481E-21					ZAK	
V3SVHSHC_7589003	GHSR			9.183E-21					GHSR	
V3SVHSHC_6044504	DNASE2B			9.258E-21					DNASE2B	
V3SVHSHC_6134825	CFL1			9.537E-21					CFL1	
V3SVHSHC_5847362	DDO			9.546E-21					DDO	
V3SVHSHC_10365722	TMEM144			9.763E-21					TMEM144	
V3SVHSHC_9712025	KIAA1456			1.031E-20					KIAA1456	
V3SVHSHC_7074005	RNASE7			1.068E-20					RNASE7	
V3SVHSHC_4637384	GALNT11			1.082E-20					GALNT11	
V3SVHSHC_5273822	HUS1			1.114E-20	7.343E-02				HUS1	
V3SVHSHC_10669751	IFRD1			1.116E-20					IFRD1	
V3SVHSHC_9363116	SLC36A2			1.148E-20					SLC36A2	
V3SVHSHC_5648735	LPHN1			1.157E-20					LPHN1	
V3SVHSHC_5090342	DGCR14			1.167E-20					DGCR14	
V3SVHSHC_4899008	LRRC8B			1.176E-20					LRRC8B	
V3SVHSHC_7002527	SLC5A4			1.203E-20					SLC5A4	
V3SVHSHC_10594412	C17orf98			1.259E-20					C17orf98	
V3SVHSHC_7209437	UBTD1			1.266E-20					UBTD1	
V3SVHSHC_5695859	MKRN3			1.271E-20					MKRN3	
V3SVHSHC_7711994	DEFB127			1.300E-20					DEFB127	
V3SVHSHC_7144328	SLCO1B1			1.429E-20					SLCO1B1	
V3SVHSHC_7013054	RILPL2			1.507E-20					RILPL2	
V3SVHSHC_6708827	AOC3			1.522E-20					AOC3	
V3SVHSHC_4663553	PPM1H			1.646E-20					PPM1H	
V3SVHSHC_6429416	RPL10L			1.688E-20					RPL10L	
V3SVHSHC_4989527	LIN9			1.736E-20					LIN9	
V3SVHSHC_5538614	AGBL4			1.781E-20					AGBL4	
V3SVHSHC_6708926	OR52H1			1.842E-20					OR52H1	
V3SVHSHC_7873001	CYP2C18			2.008E-20					CYP2C18	
V3SVHSHC_5524820	TMPRSS11B			2.019E-20					TMPRSS11B	
V3SVHSHC_10237022	ASS1			2.028E-20					ASS1	
V3SVHSHC_8436872	ZMYND15			2.037E-20					ZMYND15	
V3SVHSHC_9948437	KRTAP2-3			2.040E-20					KRTAP2-3	
V3SVHSHC_10778255	HSPA1B			2.087E-20					HSPA1B	
V3SVHSHC_7470269	Gen1			2.153E-20					Gen1	
V3SVHSHC_4815320	OR4S2			2.227E-20					OR4S2	
V3SVHSHC_8906396	CASQ1			2.240E-20					CASQ1	
V3SVHSHC_4964282	TMEM79			2.262E-20					TMEM79	
V3SVHSHC_8392256	APH1B			2.332E-20					APH1B	
V3SVHSHC_9353843	STK11			2.369E-20					STK11	
V3SVHSHC_7827593	HMGCL			2.463E-20					HMGCL	

V3SVHSHC_9382190	Adat3			2.565E-20					Adat3	
V3SVHSHC_7940783	LPAR4			2.590E-20					LPAR4	
V3SVHSHC_7679423	MS4A1			2.645E-20					MS4A1	
V3SVHSHC_9662228	NT5C1B			2.765E-20					NT5C1B	
V3SVHSHC_8839538	OR8D1			2.784E-20	5.701E-21				OR8D1	OR8D1
V3SVHSHC_8970548	DNASE1L3			2.804E-20					DNASE1L3	
V3SVHSHC_9246197	SULT2A1			2.893E-20					SULT2A1	
V3SVHSHC_9073640	NTNG2			3.244E-20					NTNG2	
V3SVHSHC_6699653	MUC21			3.318E-20					MUC21	
V3SVHSHC_7808849	RPS6KC1			3.376E-20					RPS6KC1	
V3SVHSHC_8437730	KRTAP12-4			3.633E-20					KRTAP12-4	
V3SVHSHC_7365329	ATOH7			3.662E-20					ATOH7	
V3SVHSHC_9772646	SETD6			3.684E-20					SETD6	
V3SVHSHC_10006088	DAZAP2			3.790E-20					DAZAP2	
V3SVHSHC_9155744	BUB1B			3.873E-20					BUB1B	
V3SVHSHC_7913690	CTSF			3.935E-20					CTSF	
V3SVHSHC_8462282	TARDBP			3.971E-20					TARDBP	
V3SVHSHC_8971010	TBX5			4.149E-20					TBX5	
V3SVHSHC_5100539	CCDC68			4.264E-20					CCDC68	
V3SVHSHC_8835248	CLEC3A			4.360E-20					CLEC3A	
V3SVHSHC_5204126	ZNF292			4.487E-20					ZNF292	
V3SVHSHC_8175677	GOLGA6L3			4.515E-20					GOLGA6L3	
V3SVHSHC_7895870	C1S			4.563E-20					C1S	
V3SVHSHC_10605368	LCTL			4.739E-20					LCTL	
V3SVHSHC_10373807	OR5B12			4.797E-20					OR5B12	
V3SVHSHC_8801786	GUCD1			4.852E-20					GUCD1	
V3SVHSHC_7365692	KIF12			5.024E-20					KIF12	
V3SVHSHC_9141686	PARD6B			5.073E-20					PARD6B	
V3SVHSHC_6988469	SEPT8			5.201E-20					SEPT8	
V3SVHSHC_10769840	C11orf21			5.609E-20					C11orf21	
V3SVHSHC_9208709	NDUFAF6			5.701E-20					NDUFAF6	
V3SVHSHC_8918936	PGBD3			5.721E-20					PGBD3	
V3SVHSHC_9777497	PTPRK			6.160E-20					PTPRK	
V3SVHSHC_9092483	TRMT2B			6.292E-20					TRMT2B	
V3SVHSHC_9859964	FOXO6			6.639E-20					FOXO6	
V3SVHSHC_10573622	TTC30A			6.678E-20					TTC30A	
V3SVHSHC_8825942	TMEM102			6.688E-20					TMEM102	
V3SVHSHC_9629195	TGDS			6.822E-20					TGDS	
V3SVHSHC_4721534	TARP			7.138E-20					TARP	
V3SVHSHC_10164026	SLC25A23			7.294E-20					SLC25A23	
V3SVHSHC_10779080	S100A2			7.622E-20					S100A2	
V3SVHSHC_4650716	CD226			7.715E-20					CD226	
V3SVHSHC_7340744	NPL			7.926E-20					NPL	
V3SVHSHC_8487725	PARD3			8.028E-20					PARD3	
V3SVHSHC_6811028	SH2D7			8.176E-20					SH2D7	
V3SVHSHC_10449344	ANGPT4			8.578E-20					ANGPT4	
V3SVHSHC_7446146	PSMB11			8.734E-20					PSMB11	
V3SVHSHC_7586396	OLFML2A			8.774E-20					OLFML2A	
V3SVHSHC_7291640	C11orf70			8.899E-20					C11orf70	
V3SVHSHC_5706452	CCDC178			9.537E-20					CCDC178	
V3SVHSHC_8773043	TRIM68			9.854E-20					TRIM68	
V3SVHSHC_10047734	MRPL36			1.003E-19					MRPL36	

V3SVHSHC_7134824	RFT1			1.006E-19					RFT1	
V3SVHSHC_9840131	BBOX1			1.043E-19					BBOX1	
V3SVHSHC_8574515	FUT6			1.059E-19					FUT6	
V3SVHSHC_8419613	CLTA			1.069E-19					CLTA	
V3SVHSHC_7921907	GDF5			1.092E-19					GDF5	
V3SVHSHC_5624612	ZBTB32			1.107E-19					ZBTB32	
V3SVHSHC_8634905	AP3B1			1.107E-19					AP3B1	
V3SVHSHC_9990347	BMP4			1.176E-19					BMP4	
V3SVHSHC_8626754	SAA2-SAA4			1.190E-19					SAA2-SAA4	
V3SVHSHC_9921971	LGALS7B			1.192E-19					LGALS7B	
V3SVHSHC_10549730	ANXA13			1.200E-19					ANXA13	
V3SVHSHC_6206171	CASC10			1.240E-19					CASC10	
V3SVHSHC_9093968	LOC101927789			1.268E-19					LOC101927789	
V3SVHSHC_8793206	PTPRN			1.291E-19					PTPRN	
V3SVHSHC_5769713	CCRL2			1.301E-19					CCRL2	
V3SVHSHC_6567752	TRIM31			1.389E-19					TRIM31	
V3SVHSHC_8056514	LMBR1			1.446E-19					LMBR1	
V3SVHSHC_7138982	RPL23A			1.476E-19					RPL23A	
V3SVHSHC_7612004	GSTO1			1.519E-19					GSTO1	
V3SVHSHC_5233694	RELN			1.623E-19					RELN	
V3SVHSHC_9880259	LOC100506127			1.625E-19					LOC100506127	
V3SVHSHC_7635764	CHIA			1.678E-19					CHIA	
V3SVHSHC_5102519	EFCAB13			1.961E-19					EFCAB13	
V3SVHSHC_10216430	CACFD1			1.975E-19					CACFD1	
V3SVHSHC_7234682	TENM3			2.091E-19					TENM3	
V3SVHSHC_6377309	COMP			2.112E-19					COMP	
V3SVHSHC_8907419	MRPL49			2.133E-19					MRPL49	
V3SVHSHC_10536365	TXNRD1			2.153E-19					TXNRD1	
V3SVHSHC_5337050	COX6B1			2.154E-19					COX6B1	
V3SVHSHC_9955829	SPAG5			2.267E-19					SPAG5	
V3SVHSHC_5288639	MTMR4			2.288E-19					MTMR4	
V3SVHSHC_8335661	RERGL			2.291E-19					RERGL	
V3SVHSHC_5570030	NAA25			2.292E-19					NAA25	
V3SVHSHC_5916464	ZNF419			2.342E-19					ZNF419	
V3SVHSHC_5403578	SLC35D3			2.461E-19					SLC35D3	
V3SVHSHC_5708828	FAM9B			2.543E-19					FAM9B	
V3SVHSHC_10006352	CEP57			2.576E-19					CEP57	
V3SVHSHC_6357905	PRIMPOL			2.613E-19					PRIMPOL	
V3SVHSHC_6933557	VCY1B			2.653E-19					VCY1B	
V3SVHSHC_7233296	LYPD5			2.749E-19					LYPD5	
V3SVHSHC_9779906	CNN2			2.759E-19					CNN2	
V3SVHSHC_9891776	ATP6V0A2			2.830E-19					ATP6V0A2	
V3SVHSHC_10508117	HIP1R			2.877E-19					HIP1R	
V3SVHSHC_5167430	SYNGAP1			3.016E-19					SYNGAP1	
V3SVHSHC_9722387	SENP8			3.067E-19					SENP8	
V3SVHSHC_6858350	RASL10A			3.078E-19					RASL10A	
V3SVHSHC_10362785	FPGT			3.084E-19					FPGT	
V3SVHSHC_9108290	ARHGAP19			3.095E-19					ARHGAP19	
V3SVHSHC_8771426	CDK5			3.274E-19					CDK5	
V3SVHSHC_5840861	C6ORF195			3.283E-19					C6ORF195	
V3SVHSHC_8370443	WDR31			3.578E-19					WDR31	

V3SVHSHC_4669757	GCNT1			3.674E-19					GCNT1	
V3SVHSHC_6025265	LHX5			3.873E-19					LHX5	
V3SVHSHC_7279925	GLA			3.911E-19					GLA	
V3SVHSHC_4808390	TAF12			4.386E-19					TAF12	
V3SVHSHC_8473040	TMX3			4.404E-19					TMX3	
V3SVHSHC_6280784	CERCAM			4.505E-19					CERCAM	
V3SVHSHC_5237093	SOX11			4.517E-19					SOX11	
V3SVHSHC_8556728	BAGE2			4.582E-19					BAGE2	
V3SVHSHC_10307213	OR5AN1			4.603E-19					OR5AN1	
V3SVHSHC_10427927	FLVCR1			4.634E-19					FLVCR1	
V3SVHSHC_10591805	MTNR1B			4.770E-19					MTNR1B	
V3SVHSHC_6060344	ERICH1			4.912E-19					ERICH1	
V3SVHSHC_5182577	PRPF38B			5.085E-19					PRPF38B	
V3SVHSHC_9591080	METTL9			5.151E-19					METTL9	
V3SVHSHC_6054701	C1orf159			5.324E-19					C1orf159	
V3SVHSHC_5400311	RASGRP1			5.428E-19					RASGRP1	
V3SVHSHC_7830431	LOC100506922			5.497E-19					LOC10050<br>6922	
V3SVHSHC_9205508	CMTM3			5.732E-19					CMTM3	
V3SVHSHC_10094132	IPO5			5.836E-19					IPO5	
V3SVHSHC_7811390	NXNL1			5.955E-19					NXNL1	
V3SVHSHC_7994672	PRAMEF5			5.981E-19					PRAMEF5	
V3SVHSHC_8541614	IRF2BPL			6.065E-19					IRF2BPL	
V3SVHSHC_9742022	CYP24A1			6.234E-19					CYP24A1	
V3SVHSHC_8989193	OR8J3			6.380E-19					OR8J3	
V3SVHSHC_7914845	PRKCI			7.074E-19					PRKCI	
V3SVHSHC_6870197	SLC1A1			7.142E-19					SLC1A1	
V3SVHSHC_7813964	RND2			7.301E-19					RND2	
V3SVHSHC_10437728	CDC14B			7.311E-19					CDC14B	
V3SVHSHC_4726682	USP28			7.422E-19					USP28	
V3SVHSHC_7684439	LOC199882			7.659E-19					LOC19988<br>2	
V3SVHSHC_5498255	ZNF879			8.155E-19					ZNF879	
V3SVHSHC_7028762	KTI12			8.156E-19					KTI12	
V3SVHSHC_4752983	HK2			8.244E-19					HK2	
V3SVHSHC_6179210	PTP4A1			8.353E-19					PTP4A1	
V3SVHSHC_10671698	SH3RF3			8.451E-19					SH3RF3	
V3SVHSHC_5818190	ERAL1			8.791E-19					ERAL1	
V3SVHSHC_9797099	TERT			8.806E-19					TERT	
V3SVHSHC_9813467	EEA1			8.836E-19					EEA1	
V3SVHSHC_10704929	CTNNB1			9.191E-19					CTNNB1	
V3SVHSHC_4965734	HOXD9			9.315E-19					HOXD9	
V3SVHSHC_6454331	GNAI1			9.935E-19					GNAI1	
V3SVHSHC_5554388	MFNG			1.008E-18					MFNG	
V3SVHSHC_9829406	KLHL36			1.015E-18					KLHL36	
V3SVHSHC_5865710	AP5B1			1.035E-18					AP5B1	
V3SVHSHC_9130796	PPFIA3			1.066E-18					PPFIA3	
V3SVHSHC_5486903	OR4F17			1.069E-18					OR4F17	
V3SVHSHC_8846237	OTUB1			1.105E-18					OTUB1	
V3SVHSHC_5452748	GUCY2D			1.113E-18					GUCY2D	
V3SVHSHC_8658764	CHAMP1			1.142E-18					CHAMP1	
V3SVHSHC_6330746	LOC101928991			1.163E-18					LOC10192<br>8991	
V3SVHSHC_8688761	PLAC1			1.235E-18					PLAC1	
V3SVHSHC_6625931	CGRRF1			1.292E-18					CGRRF1	

V3SVHSHC_6501158	STAT4			1.293E-18					STAT4	
V3SVHSHC_10384532	SSBP4			1.310E-18					SSBP4	
V3SVHSHC_6062654	MXI1			1.350E-18					MXI1	
V3SVHSHC_9928307	HKDC1			1.370E-18					HKDC1	
V3SVHSHC_4852775	MUC7			1.440E-18					MUC7	
V3SVHSHC_10745915	DNAJB7			1.447E-18					DNAJB7	
V3SVHSHC_9293090	SYN1			1.607E-18					SYN1	
V3SVHSHC_5921612	PTPN14			1.658E-18					PTPN14	
V3SVHSHC_10719482	TMED9			1.803E-18					TMED9	
V3SVHSHC_8514686	THRSP			1.834E-18					THRSP	
V3SVHSHC_10041893	AIF1L			1.843E-18					AIF1L	
V3SVHSHC_9330281	TMEM129			1.913E-18					TMEM129	
V3SVHSHC_5288672	TGM5			1.995E-18					TGM5	
V3SVHSHC_7118126	LCNL1			2.003E-18					LCNL1	
V3SVHSHC_8466374	ARMC8			2.054E-18					ARMC8	
V3SVHSHC_8428226	SLC19A2			2.067E-18					SLC19A2	
V3SVHSHC_6237587	IDS			2.144E-18					IDS	
V3SVHSHC_9847061	CNTNAP3B			2.203E-18					CNTNAP3 B	
V3SVHSHC_9265205	PLK3			2.287E-18					PLK3	
V3SVHSHC_10276622	Cers2			2.289E-18					Cers2	
V3SVHSHC_5943194	VCL			2.334E-18					VCL	
V3SVHSHC_6236531	REV3L			2.413E-18					REV3L	
V3SVHSHC_7252370	CUTA			2.428E-18					CUTA	
V3SVHSHC_7948934	TNF			2.467E-18					TNF	
V3SVHSHC_10317080	RHOB			2.607E-18					RHOB	
V3SVHSHC_6709454	F9			2.628E-18					F9	
V3SVHSHC_9642626	FAM151B			2.634E-18					FAM151B	
V3SVHSHC_9591740	TBX18			2.703E-18					TBX18	
V3SVHSHC_8828285	DEFA1B			2.789E-18					DEFA1B	
V3SVHSHC_10317245	ZNF319			2.870E-18					ZNF319	
V3SVHSHC_10443965	C17orf70			2.897E-18					C17orf70	
V3SVHSHC_5411201	LNX2			2.934E-18					LNX2	
V3SVHSHC_6730211	ALDH8A1			3.082E-18					ALDH8A1	
V3SVHSHC_4841621	WDR31			3.082E-18					WDR31	
V3SVHSHC_7776212	TK2			3.132E-18					TK2	
V3SVHSHC_9885308	P2RX4			3.174E-18					P2RX4	
V3SVHSHC_8827823	CBX6			3.190E-18					CBX6	
V3SVHSHC_6362987	ZC3HC1			3.438E-18					ZC3HC1	
V3SVHSHC_7006058	VWF			3.561E-18					VWF	
V3SVHSHC_5891648	TMEM214			3.677E-18					TMEM214	
V3SVHSHC_9819440	GAS6			3.791E-18					GAS6	
V3SVHSHC_10084991	TRIM62			3.946E-18					TRIM62	
V3SVHSHC_8334176	PET117			3.953E-18	4.238E-15				PET117	PET117
V3SVHSHC_8487098	MAP1LC3BP1			4.119E-18					MAP1LC3 BP1	
V3SVHSHC_6927419	LEKR1			4.251E-18					LEKR1	
V3SVHSHC_8932499	SMARCC1			4.266E-18					SMARCC1	
V3SVHSHC_6161159	ECHDC2			4.277E-18					ECHDC2	
V3SVHSHC_4870727	Magt1			4.457E-18					Magt1	
V3SVHSHC_8414036	SMCO4			4.458E-18					SMCO4	
V3SVHSHC_6744467	SEMA4F			4.519E-18					SEMA4F	
V3SVHSHC_7444892	GKN2			4.585E-18					GKN2	
V3SVHSHC_5082719	ABCA3			4.637E-18					ABCA3	
V3SVHSHC_4727309	FGFR3			4.652E-18					FGFR3	

V3SVHSHC_10543988	PAGE2B			4.709E-18					PAGE2B	
V3SVHSHC_7227356	ESAM			4.744E-18					ESAM	
V3SVHSHC_5138423	GOLGA4			4.749E-18					GOLGA4	
V3SVHSHC_6036254	TRIM59			4.970E-18					TRIM59	
V3SVHSHC_4899206	PRRC2C			5.233E-18					PRRC2C	
V3SVHSHC_7005992	DDX51			5.726E-18					DDX51	
V3SVHSHC_10302923	PIK3C2A			5.814E-18	1.164E-10				PIK3C2A	PIK3C2A
V3SVHSHC_9279824	KIF5C			5.839E-18					KIF5C	
V3SVHSHC_5053811	PCBP2			5.928E-18					PCBP2	
V3SVHSHC_9240554	SPATA31A6			6.048E-18					SPATA31A6	
V3SVHSHC_8123768	NPY4R			6.368E-18					NPY4R	
V3SVHSHC_8771756	RHOH			6.577E-18					RHOH	
V3SVHSHC_7046483	PSMC1			6.632E-18					PSMC1	
V3SVHSHC_10420370	MGAT5			6.736E-18					MGAT5	
V3SVHSHC_8940023	CSF2RB			7.068E-18					CSF2RB	
V3SVHSHC_6410903	MMP28			7.318E-18					MMP28	
V3SVHSHC_7012196	PCDH9			7.759E-18					PCDH9	
V3SVHSHC_7028696	ANPEP			7.824E-18					ANPEP	
V3SVHSHC_6754037	LOC391322			7.916E-18					LOC391322	
V3SVHSHC_10059878	VAMP8			7.925E-18					VAMP8	
V3SVHSHC_8165249	C11orf95			8.329E-18					C11orf95	
V3SVHSHC_8184488	LMAN2			8.383E-18					LMAN2	
V3SVHSHC_5358995	WNT3A			8.911E-18					WNT3A	
V3SVHSHC_9435320	B2M			9.027E-18					B2M	
V3SVHSHC_7758227	SLC35A2			9.121E-18					SLC35A2	
V3SVHSHC_10115318	MFI2			9.122E-18					MFI2	
V3SVHSHC_9779675	RFX5			9.168E-18					RFX5	
V3SVHSHC_9456275	PRAMEF7			9.725E-18					PRAMEF7	
V3SVHSHC_7180133	HTR2A			9.813E-18					HTR2A	
V3SVHSHC_6395492	LMAN2			9.882E-18					LMAN2	
V3SVHSHC_5791757	MTRNR2L5			1.012E-17					MTRNR2L5	
V3SVHSHC_10437464	MFAP4			1.017E-17					MFAP4	
V3SVHSHC_4920524	MMP10			1.027E-17					MMP10	
V3SVHSHC_9822740	CCDC120			1.036E-17					CCDC120	
V3SVHSHC_10278800	ZSCAN20			1.052E-17					ZSCAN20	
V3SVHSHC_9565406	MIEF1			1.053E-17	4.672E-01				MIEF1	
V3SVHSHC_5685233	Cela1			1.089E-17					Cela1	
V3SVHSHC_8043380	ENKD1			1.099E-17					ENKD1	
V3SVHSHC_7121756	TP53I11			1.105E-17					TP53I11	
V3SVHSHC_7776179	PPP1CB			1.274E-17					PPP1CB	
V3SVHSHC_6980615	CCBE1			1.313E-17					CCBE1	
V3SVHSHC_6089912	SVIL			1.342E-17					SVIL	
V3SVHSHC_9595601	APOBEC3A			1.443E-17					APOBEC3A	
V3SVHSHC_5284382	COL8A1			1.477E-17					COL8A1	
V3SVHSHC_9677375	MYO9B			1.495E-17					MYO9B	
V3SVHSHC_9460103	RNASE6			1.514E-17					RNASE6	
V3SVHSHC_7694801	NKIRAS2			1.526E-17					NKIRAS2	
V3SVHSHC_4918148	ARMC8			1.559E-17					ARMC8	
V3SVHSHC_5087570	CCDC36			1.658E-17					CCDC36	
V3SVHSHC_10225208	GLIPR1L1			1.769E-17					GLIPR1L1	
V3SVHSHC_7892669	CCNF			1.780E-17					CCNF	

V3SVHSHC_7025363	RBM44			1.799E-17					RBM44	
V3SVHSHC_6024176	Rpl18a			1.851E-17					Rpl18a	
V3SVHSHC_10813136	KRTAP12-2			2.122E-17					KRTAP12-2	
V3SVHSHC_10471190	OPN3			2.147E-17					OPN3	
V3SVHSHC_6049421	POTEI			2.228E-17					POTEI	
V3SVHSHC_7685990	STXBP5			2.233E-17					STXBP5	
V3SVHSHC_6331043	CCDC54			2.236E-17					CCDC54	
V3SVHSHC_8230589	KLHL13			2.422E-17					KLHL13	
V3SVHSHC_7572239	IFNW1			2.466E-17					IFNW1	
V3SVHSHC_7086215	DPPA5			2.516E-17					DPPA5	
V3SVHSHC_5741828	FHAD1			2.532E-17					FHAD1	
V3SVHSHC_4852907	NME2			2.533E-17					NME2	
V3SVHSHC_6654377	ZNF32			2.538E-17					ZNF32	
V3SVHSHC_9714368	LPO			2.585E-17					LPO	
V3SVHSHC_5674376	PRR14L			2.633E-17					PRR14L	
V3SVHSHC_10723310	CRYL1			2.646E-17					CRYL1	
V3SVHSHC_7803437	PTPN18			2.702E-17					PTPN18	
V3SVHSHC_9249332	TRIM49D1			2.705E-17					TRIM49D1	
V3SVHSHC_8085257	TMCO4			2.751E-17					TMCO4	
V3SVHSHC_9780962	TSR2			2.758E-17					TSR2	
V3SVHSHC_8099018	SOX11			2.934E-17					SOX11	
V3SVHSHC_10846829	ZNF720			2.970E-17					ZNF720	
V3SVHSHC_6318206	TAS2R46			2.994E-17					TAS2R46	
V3SVHSHC_5385296	Ttc23l			3.008E-17					Ttc23l	
V3SVHSHC_5458094	GOLGA6L9			3.099E-17					GOLGA6L9	
V3SVHSHC_5888183	LMX1B			3.191E-17					LMX1B	
V3SVHSHC_7437764	S100PBP			3.192E-17					S100PBP	
V3SVHSHC_4864457	DNASE2B			3.194E-17					DNASE2B	
V3SVHSHC_6739583	SEPW1			3.199E-17					SEPW1	
V3SVHSHC_9741626	RAD23B			3.414E-17					RAD23B	
V3SVHSHC_7013945	XKRX			3.442E-17					XKRX	
V3SVHSHC_8951408	BMP7			3.445E-17					BMP7	
V3SVHSHC_6028004	GAGE2E			3.477E-17					GAGE2E	
V3SVHSHC_9472412	DNAJB6			3.563E-17					DNAJB6	
V3SVHSHC_6152480	KLF6			3.680E-17					KLF6	
V3SVHSHC_9629855	PBX2			3.708E-17					PBX2	
V3SVHSHC_5827496	NDUFA9			3.999E-17					NDUFA9	
V3SVHSHC_8922467	RAB21			4.023E-17					RAB21	
V3SVHSHC_6885014	OR2M4			4.039E-17					OR2M4	
V3SVHSHC_5072984	ITM2B			4.058E-17					ITM2B	
V3SVHSHC_4633259	KIAA1244			4.149E-17					KIAA1244	
V3SVHSHC_5646689	LDOC1L			4.220E-17					LDOC1L	
V3SVHSHC_9770468	FAM206A			4.716E-17					FAM206A	
V3SVHSHC_5903660	G3BP2			4.777E-17					G3BP2	
V3SVHSHC_5401070	RIPK2			4.779E-17					RIPK2	
V3SVHSHC_7725425	MAP4K5			4.892E-17					MAP4K5	
V3SVHSHC_8803898	PPP4R1			5.118E-17					PPP4R1	
V3SVHSHC_9455813	CARD14			5.613E-17					CARD14	
V3SVHSHC_9595931	OR11H4			5.670E-17					OR11H4	
V3SVHSHC_9164192	RGPD2			5.816E-17					RGPD2	
V3SVHSHC_7166504	Clec18a			5.888E-17					Clec18a	
V3SVHSHC_6326819	CD151			6.077E-17					CD151	
V3SVHSHC_9865871	FAM229A			6.092E-17					FAM229A	

V3SVHSHC_6464066	CEACAM16			6.457E-17					CEACAM16	
V3SVHSHC_7691633	SCAI			6.470E-17					SCAI	
V3SVHSHC_6581777	Krtdap			6.660E-17					Krtdap	
V3SVHSHC_9504752	WDR75			6.704E-17					WDR75	
V3SVHSHC_8202902	PKIA			6.861E-17					PKIA	
V3SVHSHC_5343254	MMP17			7.112E-17					MMP17	
V3SVHSHC_9150398	TDRD5			7.228E-17					TDRD5	
V3SVHSHC_10022390	ATF7IP2			7.713E-17					ATF7IP2	
V3SVHSHC_6187625	RPS6KA1			8.151E-17					RPS6KA1	
V3SVHSHC_7325168	DEFB121			8.186E-17					DEFB121	
V3SVHSHC_9883724	SPIDR			8.186E-17					SPIDR	
V3SVHSHC_10556363	KLHDC9			8.222E-17					KLHDC9	
V3SVHSHC_4989131	ZNF165			8.274E-17					ZNF165	
V3SVHSHC_4655996	ZMYND11			8.390E-17					ZMYND11	
V3SVHSHC_5995664	CD207			8.434E-17					CD207	
V3SVHSHC_5074469	CCDC104			8.480E-17					CCDC104	
V3SVHSHC_8474657	SLC46A1			8.505E-17					SLC46A1	
V3SVHSHC_4971344	C4orf27			9.331E-17	1.620E-01				C4orf27	
V3SVHSHC_8966390	PITPNM3			9.412E-17					PITPNM3	
V3SVHSHC_7853564	NFU1			9.671E-17					NFU1	
V3SVHSHC_5642366	LAT2			1.004E-16					LAT2	
V3SVHSHC_9076049	ADAT2			1.024E-16					ADAT2	
V3SVHSHC_6663023	MED20			1.026E-16					MED20	
V3SVHSHC_10326056	PCDH18			1.063E-16					PCDH18	
V3SVHSHC_4731203	TMEM231			1.080E-16					TMEM231	
V3SVHSHC_8137232	BRINP1			1.102E-16					BRINP1	
V3SVHSHC_10366415	TMEM255A			1.121E-16					TMEM255A	
V3SVHSHC_9345923	TMEM107			1.161E-16					TMEM107	
V3SVHSHC_5024639	C12orf65			1.180E-16					C12orf65	
V3SVHSHC_6017015	HRNR			1.194E-16					HRNR	
V3SVHSHC_8084168	RANBP9			1.253E-16					RANBP9	
V3SVHSHC_8389781	B3GNT2			1.263E-16					B3GNT2	
V3SVHSHC_7991108	GLUD2			1.270E-16					GLUD2	
V3SVHSHC_9532538	ASB15			1.299E-16					ASB15	
V3SVHSHC_6193268	C9ORF91			1.313E-16					C9ORF91	
V3SVHSHC_7404203	IL2RA			1.324E-16					IL2RA	
V3SVHSHC_5405558	OAZ1			1.329E-16					OAZ1	
V3SVHSHC_9210755	CCL22			1.340E-16					CCL22	
V3SVHSHC_7134032	TBC1D5			1.344E-16					TBC1D5	
V3SVHSHC_5245937	REEP3			1.393E-16					REEP3	
V3SVHSHC_4852346	FEZ2			1.394E-16					FEZ2	
V3SVHSHC_10231841	CHGA			1.401E-16					CHGA	
V3SVHSHC_6964577	LETM1			1.422E-16					LETM1	
V3SVHSHC_8426180	FAM209B			1.464E-16					FAM209B	
V3SVHSHC_9365228	GPR162			1.472E-16					GPR162	
V3SVHSHC_5758031	CRX			1.593E-16					CRX	
V3SVHSHC_5046023	KRT78			1.678E-16					KRT78	
V3SVHSHC_5957153	C11ORF31			1.689E-16					C11ORF31	
V3SVHSHC_8180990	VIPAS39			1.788E-16					VIPAS39	
V3SVHSHC_5686520	LOC101928558			1.829E-16					LOC101928558	
V3SVHSHC_6767798	FAM65A			1.841E-16					FAM65A	
V3SVHSHC_8962595	VAMP7			1.863E-16					VAMP7	

V3SVHSHC_9465581	CNDP2			1.908E-16					CNDP2	
V3SVHSHC_10143500	BDH2			1.940E-16					BDH2	
V3SVHSHC_6511784	RER1			1.975E-16					RER1	
V3SVHSHC_8316257	KIAA1244			1.996E-16					KIAA1244	
V3SVHSHC_5076746	MTRNR2L3			2.103E-16					MTRNR2L 3	
V3SVHSHC_8633123	KIF13A			2.117E-16					KIF13A	
V3SVHSHC_5014838	SNAI3			2.240E-16					SNAI3	
V3SVHSHC_7738856	CCZ1B			2.273E-16					CCZ1B	
V3SVHSHC_8389319	CYBA			2.382E-16					CYBA	
V3SVHSHC_9744035	PON3			2.400E-16					PON3	
V3SVHSHC_10195211	KRT78			2.425E-16					KRT78	
V3SVHSHC_6839837	NAGPA			2.433E-16					NAGPA	
V3SVHSHC_6521090	C1orf112			2.457E-16					C1orf112	
V3SVHSHC_8851484	LPA			2.475E-16					LPA	
V3SVHSHC_5235278	SBK3			2.492E-16					SBK3	
V3SVHSHC_10536893	KCNAB3			2.681E-16					KCNAB3	
V3SVHSHC_10630580	MIB2			2.834E-16					MIB2	
V3SVHSHC_4825682	THRAP3			2.896E-16					THRAP3	
V3SVHSHC_8542373	CAMP			2.915E-16					CAMP	
V3SVHSHC_7729385	TRDN			2.996E-16					TRDN	
V3SVHSHC_9709880	PLEKHJ1			3.001E-16					PLEKHJ1	
V3SVHSHC_6985070	KLC3			3.036E-16					KLC3	
V3SVHSHC_8042852	THSD7B			3.038E-16					THSD7B	
V3SVHSHC_10568870	PRDM4			3.175E-16					PRDM4	
V3SVHSHC_7704338	LOC101929991			3.231E-16					LOC10192 9991	
V3SVHSHC_7354901	YIPF5			3.430E-16					YIPF5	
V3SVHSHC_10267646	TMEM249			3.437E-16					TMEM249	
V3SVHSHC_7369916	PKDCC			3.453E-16					PKDCC	
V3SVHSHC_6944051	GABRP			3.605E-16					GABRP	
V3SVHSHC_9066512	BTAF1			3.779E-16					BTAF1	
V3SVHSHC_5009426	CASQ2			3.814E-16					CASQ2	
V3SVHSHC_8865773	GLYCTK			3.858E-16					GLYCTK	
V3SVHSHC_7546961	KATNA1			3.903E-16					KATNA1	
V3SVHSHC_4661441	RCN3			3.929E-16					RCN3	
V3SVHSHC_5544785	PTBP3			4.064E-16					PTBP3	
V3SVHSHC_5431100	STK10			4.153E-16					STK10	
V3SVHSHC_8668037	FLJ22184			4.174E-16					FLJ22184	
V3SVHSHC_4881584	MAGEB1			4.193E-16					MAGEB1	
V3SVHSHC_6504458	STK4			4.207E-16					STK4	
V3SVHSHC_6538514	HOXA1			4.280E-16					HOXA1	
V3SVHSHC_5114069	RAC1			4.290E-16					RAC1	
V3SVHSHC_10164818	BSX			4.297E-16					BSX	
V3SVHSHC_5645765	SYN1			4.332E-16					SYN1	
V3SVHSHC_6983321	BAIAP2L2			4.400E-16					BAIAP2L2	
V3SVHSHC_9261377	BAGE5			4.546E-16					BAGE5	
V3SVHSHC_7340612	C9ORF78			4.570E-16					C9ORF78	
V3SVHSHC_8048627	UBR7			4.646E-16					UBR7	
V3SVHSHC_8866961	SIPA1L3			4.769E-16					SIPA1L3	
V3SVHSHC_10664108	NDUFA4L2			4.976E-16					NDUFA4L 2	
V3SVHSHC_10630151	ZNF419			5.033E-16					ZNF419	
V3SVHSHC_6806738	VPRBP			5.495E-16					VPRBP	
V3SVHSHC_10761095	HADHB			5.517E-16					HADHB	

V3SVHSHC_10421525	THAP10			5.738E-16					THAP10	
V3SVHSHC_7302794	MLIP			5.867E-16					MLIP	
V3SVHSHC_9997838	LOC101060581			5.900E-16					LOC101060581	
V3SVHSHC_8059583	SMARCAD1			6.356E-16					SMARCAD1	
V3SVHSHC_6847955	PRKCD			6.424E-16					PRKCD	
V3SVHSHC_10838249	TCP11L2			6.427E-16					TCP11L2	
V3SVHSHC_10632164	UBLCP1			6.553E-16					UBLCP1	
V3SVHSHC_8228675	HNF4G			6.863E-16					HNF4G	
V3SVHSHC_7641737	CLSTN2			6.965E-16					CLSTN2	
V3SVHSHC_8296424	SASS6			7.015E-16					SASS6	
V3SVHSHC_9230654	ZNF705A			7.153E-16					ZNF705A	
V3SVHSHC_10102943	TTBK2			7.417E-16					TTBK2	
V3SVHSHC_9842771	IGFL1			7.583E-16					IGFL1	
V3SVHSHC_8840660	EDEM2			7.646E-16					EDEM2	
V3SVHSHC_10311569	RNF113A			7.782E-16					RNF113A	
V3SVHSHC_9570191	XIRP1			7.881E-16					XIRP1	
V3SVHSHC_9452447	LOC101928093			7.889E-16					LOC101928093	
V3SVHSHC_9875639	GYS1			7.967E-16					GYS1	
V3SVHSHC_8111558	TNNI3K			8.049E-16					TNNI3K	
V3SVHSHC_7693514	FGFBP3			8.143E-16					FGFBP3	
V3SVHSHC_8018333	KIF26A			8.194E-16					KIF26A	
V3SVHSHC_7917848	NCSTN			8.216E-16					NCSTN	
V3SVHSHC_9193496	CCDC177			8.236E-16					CCDC177	
V3SVHSHC_5150930	FRK			8.686E-16					FRK	
V3SVHSHC_5425160	GPR133			8.844E-16					GPR133	
V3SVHSHC_6062192	LMOD3			1.015E-15					LMOD3	
V3SVHSHC_9494687	TNFAIP1			1.021E-15					TNFAIP1	
V3SVHSHC_6353285	DDTL			1.038E-15					DDTL	
V3SVHSHC_9969920	C9ORF72			1.040E-15					C9ORF72	
V3SVHSHC_4718102	PDIA5			1.040E-15					PDIA5	
V3SVHSHC_10317971	ZIC5			1.052E-15					ZIC5	
V3SVHSHC_7239533	MOGAT3			1.067E-15					MOGAT3	
V3SVHSHC_5155781	RFPL4B			1.096E-15					RFPL4B	
V3SVHSHC_6981638	DOK1			1.120E-15					DOK1	
V3SVHSHC_7775222	DUOXA2			1.129E-15					DUOXA2	
V3SVHSHC_9978533	ELAC2			1.166E-15					ELAC2	
V3SVHSHC_6055163	XCR1			1.181E-15					XCR1	
V3SVHSHC_6842972	LAT			1.202E-15					LAT	
V3SVHSHC_6427469	METTL17			1.207E-15					METTL17	
V3SVHSHC_9795119	CCDC168			1.264E-15					CCDC168	
V3SVHSHC_10828085	HES4			1.272E-15					HES4	
V3SVHSHC_8733608	PNLIPRP2			1.274E-15					PNLIPRP2	
V3SVHSHC_6668270	PER1			1.288E-15					PER1	
V3SVHSHC_8275238	PCDHGB3			1.319E-15					PCDHGB3	
V3SVHSHC_8034173	CACNA1F			1.365E-15					CACNA1F	
V3SVHSHC_6018467	NDST4			1.366E-15					NDST4	
V3SVHSHC_4973918	FKBP7			1.523E-15					FKBP7	
V3SVHSHC_8087864	DFFB			1.563E-15					DFFB	
V3SVHSHC_7579037	ELMO3			1.579E-15					ELMO3	
V3SVHSHC_7100900	FCRL1			1.632E-15					FCRL1	
V3SVHSHC_7986191	HGFAC			1.635E-15					HGFAC	
V3SVHSHC_9782876	MAPK8IP1			1.704E-15					MAPK8IP1	

V3SVHSHC_7351007	CKM			1.726E-15					CKM		
V3SVHSHC_10407203	RFTN1			1.739E-15					RFTN1		
V3SVHSHC_6908510	NPHP1			1.793E-15					NPHP1		
V3SVHSHC_10844948	NKG7			1.827E-15					NKG7		
V3SVHSHC_9165908	SERPINE3			1.837E-15					SERPINE3		
V3SVHSHC_5318999	FHIT			1.857E-15					FHIT		
V3SVHSHC_10761458	GRAMD2			1.906E-15					GRAMD2		
V3SVHSHC_7088855	GATA6			1.925E-15					GATA6		
V3SVHSHC_8027771	EFCAB3			2.032E-15					EFCAB3		
V3SVHSHC_9457595	PDE9A			2.060E-15					PDE9A		
V3SVHSHC_7079648	GOLGA8M			2.113E-15					GOLGA8M		
V3SVHSHC_10295069	SMIM7			2.148E-15					SMIM7		
V3SVHSHC_10201679	KCNA1			2.175E-15					KCNA1		
V3SVHSHC_9997706	SLC22A2			2.186E-15					SLC22A2		
V3SVHSHC_6335795	SLC44A4			2.191E-15					SLC44A4		
V3SVHSHC_5716781	MRGPRX3			2.208E-15					MRGPRX3		
V3SVHSHC_9351401	BGN			2.248E-15					BGN		
V3SVHSHC_6568973	PDE1B			2.253E-15					PDE1B		
V3SVHSHC_7061795	NUMA1			2.276E-15					NUMA1		
V3SVHSHC_7463669	IFT20			2.322E-15					IFT20		
V3SVHSHC_6955238	FOXA3			2.387E-15					FOXA3		
V3SVHSHC_8046548	RAB3B			2.404E-15					RAB3B		
V3SVHSHC_9075818	KLF1			2.439E-15					KLF1		
V3SVHSHC_5645996	NRSN2			2.520E-15					NRSN2		
V3SVHSHC_10499075	MIEF2			2.551E-15					MIEF2		
V3SVHSHC_8257121	TYRP1			2.593E-15					TYRP1		
V3SVHSHC_7770833	POLN			2.596E-15					POLN		
V3SVHSHC_6395162	TRDMT1			2.611E-15					TRDMT1		
V3SVHSHC_6150962	RGS12			2.675E-15					RGS12		
V3SVHSHC_5339921	CDH10			2.728E-15					CDH10		
V3SVHSHC_10839404	HN1L			2.745E-15					HN1L		
V3SVHSHC_10111721	LRRC70			2.787E-15					LRRC70		
V3SVHSHC_9931178	CDCA7L			2.979E-15					CDCA7L		
V3SVHSHC_7121690	P2RY8			3.069E-15					P2RY8		
V3SVHSHC_6549602	LTBP3			3.069E-15					LTBP3		
V3SVHSHC_9394334	RNF133			3.400E-15					RNF133		
V3SVHSHC_5777369	SCN3B			3.479E-15					SCN3B		
V3SVHSHC_9796142	CCDC59			3.494E-15					CCDC59		
V3SVHSHC_5587454	RASAL3			3.500E-15					RASAL3		
V3SVHSHC_9391430	XPO5			3.519E-15					XPO5		
V3SVHSHC_9366152	KIAA1257			3.520E-15					KIAA1257		
V3SVHSHC_8248574	RBP4			3.633E-15					RBP4		
V3SVHSHC_4908017	ZFAND1			3.690E-15					ZFAND1		
V3SVHSHC_8290088	ZNF410			3.731E-15					ZNF410		
V3SVHSHC_6246497	CACNA1E			3.804E-15					CACNA1E		
V3SVHSHC_8978996	DNAJC17			3.808E-15					DNAJC17		
V3SVHSHC_6834524	APLN			3.821E-15					APLN		
V3SVHSHC_5750804	Tctex1d2			4.071E-15					Tctex1d2		
V3SVHSHC_7659458	SYNJ2			4.137E-15					SYNJ2		
V3SVHSHC_10012061	C1S			4.207E-15					C1S		
V3SVHSHC_5375033	PLD6			4.389E-15					PLD6		
V3SVHSHC_5624975	ANKRD37			4.416E-15					ANKRD37		
V3SVHSHC_6513005	MYOD1			4.446E-15					MYOD1		
V3SVHSHC_8797331	ARL4D			4.491E-15					ARL4D		

V3SVHSHC_8828879	ANKK1			4.503E-15					ANKK1	
V3SVHSHC_10014866	MOXD1			4.647E-15					MOXD1	
V3SVHSHC_9403574	SLCO3A1			4.970E-15					SLCO3A1	
V3SVHSHC_10561775	EFNA3			5.019E-15					EFNA3	
V3SVHSHC_9789773	C11orf82			5.024E-15					C11orf82	
V3SVHSHC_9785417	SLC39A12			5.138E-15					SLC39A12	
V3SVHSHC_10519238	BOD1L2			5.157E-15					BOD1L2	
V3SVHSHC_8295500	NUP133			5.254E-15					NUP133	
V3SVHSHC_5729420	TMEM211			5.339E-15					TMEM211	
V3SVHSHC_7359521	ATIC			5.366E-15					ATIC	
V3SVHSHC_8100008	RYBP			5.481E-15					RYBP	
V3SVHSHC_10119080	IL21			5.579E-15					IL21	
V3SVHSHC_10536266	TCEANC2			5.775E-15					TCEANC2	
V3SVHSHC_9925964	CCDC27			5.886E-15					CCDC27	
V3SVHSHC_10847819	ATF6			6.123E-15					ATF6	
V3SVHSHC_5590952	WDR31			6.172E-15					WDR31	
V3SVHSHC_10034039	ACTR3C			6.257E-15					ACTR3C	
V3SVHSHC_7125617	LPPR2			6.377E-15					LPPR2	
V3SVHSHC_7257749	CBFA2T2			6.524E-15					CBFA2T2	
V3SVHSHC_7046219	EMP1			6.588E-15					EMP1	
V3SVHSHC_5474033	ZC3H12C			6.680E-15					ZC3H12C	
V3SVHSHC_4899107	Hrct1			6.825E-15					Hrct1	
V3SVHSHC_4918346	Rnls			7.007E-15					Rnls	
V3SVHSHC_10538906	ANKRD1			7.047E-15					ANKRD1	
V3SVHSHC_5651639	TDRD1			7.104E-15					TDRD1	
V3SVHSHC_7934282	OCA2			7.119E-15					OCA2	
V3SVHSHC_6708728	TSC22D3			7.619E-15					TSC22D3	
V3SVHSHC_10756442	TBC1D16			7.711E-15					TBC1D16	
V3SVHSHC_6317711	RASA1			8.046E-15					RASA1	
V3SVHSHC_10044896	GNL1			8.351E-15	2.605E-01				GNL1	
V3SVHSHC_8646521	PCDHGB5			8.402E-15					PCDHGB5	
V3SVHSHC_5348072	EFCAB7			8.435E-15					EFCAB7	
V3SVHSHC_7791392	C16orf74			8.545E-15					C16orf74	
V3SVHSHC_8646851	GANAB			8.670E-15					GANAB	
V3SVHSHC_6188879	OFCC1			8.841E-15					OFCC1	
V3SVHSHC_8713577	MFSD4			8.924E-15					MFSD4	
V3SVHSHC_6242141	PIWIL1			8.947E-15					PIWIL1	
V3SVHSHC_6915275	SH3PXD2B			8.989E-15					SH3PXD2<br>B	
V3SVHSHC_9079019	VCY			9.076E-15					VCY	
V3SVHSHC_10426871	Erlec1			9.161E-15					Erlec1	
V3SVHSHC_6993914	GALNT18			9.426E-15					GALNT18	
V3SVHSHC_6764993	KIF20B			1.016E-14					KIF20B	
V3SVHSHC_5358302	TTLL12			1.045E-14					TTLL12	
V3SVHSHC_9848183	LOC101929936			1.064E-14					LOC10192<br>9936	
V3SVHSHC_9252830	KIAA1210			1.100E-14					KIAA1210	
V3SVHSHC_7831058	MS4A7			1.119E-14					MS4A7	
V3SVHSHC_5116082	EMC10			1.141E-14					EMC10	
V3SVHSHC_8302199	AFTPH			1.228E-14					AFTPH	
V3SVHSHC_5906795	PUS10			1.234E-14					PUS10	
V3SVHSHC_5936693	FADS2			1.239E-14					FADS2	
V3SVHSHC_7659755	TNFSF11			1.268E-14					TNFSF11	
V3SVHSHC_9529997	WWC2			1.301E-14					WWC2	

V3SVHSHC_7567520	LOC100131107			1.330E-14					LOC100131107	
V3SVHSHC_8809970	NRP1			1.336E-14					NRP1	
V3SVHSHC_9899663	CDY2A			1.344E-14					CDY2A	
V3SVHSHC_8406446	MAG			1.372E-14					MAG	
V3SVHSHC_10160825	ITGA1			1.412E-14					ITGA1	
V3SVHSHC_6212540	FCER1G			1.417E-14					FCER1G	
V3SVHSHC_8604974	PROSC			1.434E-14					PROSC	
V3SVHSHC_4889900	UNC5B			1.479E-14					UNC5B	
V3SVHSHC_9379847	PSD3			1.504E-14					PSD3	
V3SVHSHC_6370874	DLGAP1			1.531E-14	2.519E-07				DLGAP1	DLGAP1
V3SVHSHC_5348501	CNTN6			1.557E-14					CNTN6	
V3SVHSHC_5339756	PPL			1.558E-14					PPL	
V3SVHSHC_8777069	EPRS			1.561E-14					EPRS	
V3SVHSHC_5744270	LOC101928044			1.633E-14					LOC101928044	
V3SVHSHC_6938408	ZNF485			1.701E-14					ZNF485	
V3SVHSHC_5888249	TRABD2B			1.709E-14					TRABD2B	
V3SVHSHC_4911878	NXF1			1.725E-14					NXF1	
V3SVHSHC_7859768	PLCL2			1.730E-14					PLCL2	
V3SVHSHC_7058462	S100A10			1.784E-14					S100A10	
V3SVHSHC_7934447	RNFT2			1.818E-14					RNFT2	
V3SVHSHC_5455751	TBCB			1.849E-14					TBCB	
V3SVHSHC_4784762	NAV3			1.911E-14					NAV3	
V3SVHSHC_7233923	MTR			1.935E-14					MTR	
V3SVHSHC_6998831	SLITRK2			1.953E-14					SLITRK2	
V3SVHSHC_8986619	PIGR			1.957E-14					PIGR	
V3SVHSHC_5800799	STAT1			1.969E-14					STAT1	
V3SVHSHC_10638203	LHX4			2.010E-14					LHX4	
V3SVHSHC_7837163	NMRK2			2.057E-14					NMRK2	
V3SVHSHC_5275505	AMER2			2.115E-14					AMER2	
V3SVHSHC_9968897	LOC100996318			2.149E-14					LOC100996318	
V3SVHSHC_6789380	ABHD13			2.207E-14					ABHD13	
V3SVHSHC_10360904	CDK10			2.335E-14					CDK10	
V3SVHSHC_8190329	SCGN			2.350E-14					SCGN	
V3SVHSHC_9331139	KPRP			2.356E-14					KPRP	
V3SVHSHC_7799378	COG2			2.364E-14					COG2	
V3SVHSHC_8656025	ZNF443			2.376E-14					ZNF443	
V3SVHSHC_9515939	PCDH12			2.444E-14					PCDH12	
V3SVHSHC_9177293	STK25			2.456E-14					STK25	
V3SVHSHC_7380707	RASSF4			2.537E-14					RASSF4	
V3SVHSHC_6905243	DLL3			2.538E-14					DLL3	
V3SVHSHC_9407303	VEGFA			2.555E-14					VEGFA	
V3SVHSHC_6371963	CXCL9			2.566E-14					CXCL9	
V3SVHSHC_8067371	WFIKKN1			2.606E-14					WFIKKN1	
V3SVHSHC_7129214	OR6M1			2.697E-14					OR6M1	
V3SVHSHC_10437035	ATP9A			2.847E-14					ATP9A	
V3SVHSHC_4988933	SYT6			2.909E-14					SYT6	
V3SVHSHC_9364040	CCL3L3			2.951E-14					CCL3L3	
V3SVHSHC_4957022	SLC32A1			2.998E-14					SLC32A1	
V3SVHSHC_4946363	IL2RA			3.005E-14					IL2RA	
V3SVHSHC_9117068	RAD23B			3.145E-14					RAD23B	
V3SVHSHC_6963521	KRTAP10-8			3.185E-14					KRTAP10-8	

V3SVHSHC_7081826	SORBS2			3.313E-14					SORBS2	
V3SVHSHC_4805288	MSH4			3.360E-14					MSH4	
V3SVHSHC_8856137	SLC2A13			3.383E-14					SLC2A13	
V3SVHSHC_7225706	RAB17			3.425E-14					RAB17	
V3SVHSHC_4814198	CITED2			3.452E-14					CITED2	
V3SVHSHC_9780830	MAP3K6			3.479E-14					MAP3K6	
V3SVHSHC_7680809	OPTN			3.607E-14					OPTN	
V3SVHSHC_9666518	GPSM3			3.629E-14					GPSM3	
V3SVHSHC_10471454	Gpn1			3.717E-14					Gpn1	
V3SVHSHC_10598603	FUT7			3.745E-14					FUT7	
V3SVHSHC_7243130	ZNF331			3.800E-14					ZNF331	
V3SVHSHC_7507427	FAM45A			3.855E-14					FAM45A	
V3SVHSHC_8534651	DUSP8			3.912E-14					DUSP8	
V3SVHSHC_5177495	IDNK			3.959E-14					IDNK	
V3SVHSHC_4900361	PPARGC1B			4.039E-14					PPARGC1B	
V3SVHSHC_7323683	LBX1			4.144E-14					LBX1	
V3SVHSHC_9484523	FAM188B			4.148E-14					FAM188B	
V3SVHSHC_10534418	MCM10			4.253E-14					MCM10	
V3SVHSHC_7202111	NKX2-4			4.295E-14					NKX2-4	
V3SVHSHC_5405327	TBC1D2			4.423E-14					TBC1D2	
V3SVHSHC_5922107	PCIF1			4.433E-14					PCIF1	
V3SVHSHC_8270783	RAPGEF5			4.648E-14					RAPGEF5	
V3SVHSHC_7506866	LPCAT4			4.742E-14					LPCAT4	
V3SVHSHC_6354341	CSF1			4.934E-14					CSF1	
V3SVHSHC_4957319	CIART			5.087E-14					CIART	
V3SVHSHC_10632857	ASB14			5.126E-14					ASB14	
V3SVHSHC_9899069	SLC9A4			5.154E-14					SLC9A4	
V3SVHSHC_6452648	SEZ6L2			5.326E-14					SEZ6L2	
V3SVHSHC_7863662	SPATA8			5.641E-14					SPATA8	
V3SVHSHC_6078989	BAIAP3			5.668E-14					BAIAP3	
V3SVHSHC_4816244	CCM2L			6.026E-14					CCM2L	
V3SVHSHC_5745029	ZBTB32			6.060E-14					ZBTB32	
V3SVHSHC_5079320	C2orf81			6.191E-14					C2orf81	
V3SVHSHC_7787960	C12ORF10			6.380E-14					C12ORF10	
V3SVHSHC_8201351	KLK15			6.938E-14					KLK15	
V3SVHSHC_5961608	TAF7L			6.946E-14					TAF7L	
V3SVHSHC_9703874	REG3G			7.092E-14					REG3G	
V3SVHSHC_7400804	OR5AS1			7.167E-14	9.492E-06				OR5AS1	OR5AS1
V3SVHSHC_7117400	PIK3CA			7.224E-14					PIK3CA	
V3SVHSHC_9378593	ADAM29			7.289E-14					ADAM29	
V3SVHSHC_8265239	FGFR3			7.355E-14					FGFR3	
V3SVHSHC_5231120	RANBP9			7.729E-14					RANBP9	
V3SVHSHC_10339289	MAN2A1			7.756E-14					MAN2A1	
V3SVHSHC_9250454	TRIM43B			7.823E-14					TRIM43B	
V3SVHSHC_8614016	TMEM11			7.921E-14					TMEM11	
V3SVHSHC_6302795	ATF6B			8.081E-14					ATF6B	
V3SVHSHC_10847456	NT5C1A			8.103E-14					NT5C1A	
V3SVHSHC_5036222	GLIS2			8.307E-14					GLIS2	
V3SVHSHC_10694468	THAP7			8.334E-14					THAP7	
V3SVHSHC_7199801	SNURF			8.462E-14					SNURF	
V3SVHSHC_8400605	ZFYVE16			8.584E-14					ZFYVE16	
V3SVHSHC_7685462	IGSF23			8.727E-14					IGSF23	

V3SVHSHC_9487658	LOC101929805			9.338E-14					LOC101929805	
V3SVHSHC_5114201	RNASE12			9.484E-14					RNASE12	
V3SVHSHC_9798947	SAA2-SAA4			9.655E-14					SAA2-SAA4	
V3SVHSHC_9735257	GPA33			9.859E-14					GPA33	
V3SVHSHC_10279295	PIK3IP1			9.885E-14					PIK3IP1	
V3SVHSHC_6396119	TRIM17			1.018E-13					TRIM17	
V3SVHSHC_10546991	TMEM251			1.021E-13					TMEM251	
V3SVHSHC_7576892	NAA60			1.029E-13					NAA60	
V3SVHSHC_6437798	LRRC6			1.036E-13					LRRC6	
V3SVHSHC_6984971	TRMT2A			1.042E-13					TRMT2A	
V3SVHSHC_5200496	SP5			1.095E-13					SP5	
V3SVHSHC_8915009	PTRH2			1.100E-13					PTRH2	
V3SVHSHC_10446407	RIPK2			1.114E-13					RIPK2	
V3SVHSHC_6125453	FAM19A1			1.121E-13					FAM19A1	
V3SVHSHC_6648998	LOC646862			1.164E-13					LOC646862	
V3SVHSHC_7179506	HSF2BP			1.226E-13					HSF2BP	
V3SVHSHC_6808190	C2orf40			1.228E-13					C2orf40	
V3SVHSHC_5726681	CHCHD6			1.259E-13					CHCHD6	
V3SVHSHC_8864354	C14ORF2			1.295E-13					C14ORF2	
V3SVHSHC_8408789	ZFY			1.331E-13					ZFY	
V3SVHSHC_6091430	LPO			1.346E-13					LPO	
V3SVHSHC_8354801	LOC101930295			1.357E-13					LOC101930295	
V3SVHSHC_9101756	C5orf54			1.364E-13					C5orf54	
V3SVHSHC_7356749	ASCL1			1.384E-13					ASCL1	
V3SVHSHC_6922865	RBPMS			1.404E-13					RBPMS	
V3SVHSHC_6030875	GNB4			1.423E-13					GNB4	
V3SVHSHC_9764000	TMEM158			1.497E-13					TMEM158	
V3SVHSHC_7849538	HIGD1B			1.501E-13					HIGD1B	
V3SVHSHC_6273920	TUBGCP6			1.501E-13					TUBGCP6	
V3SVHSHC_7661273	PSD4			1.522E-13					PSD4	
V3SVHSHC_8351039	PCBD2			1.537E-13					PCBD2	
V3SVHSHC_10129376	SRSF3			1.548E-13					SRSF3	
V3SVHSHC_10247153	ANG			1.568E-13					ANG	
V3SVHSHC_8353547	SCRT1			1.601E-13					SCRT1	
V3SVHSHC_7374767	CHRM1			1.699E-13					CHRM1	
V3SVHSHC_10655033	RGMB			1.729E-13					RGMB	
V3SVHSHC_10746047	MBD1			1.729E-13					MBD1	
V3SVHSHC_6132185	ELF3			1.777E-13					ELF3	
V3SVHSHC_7571612	RLN1			1.858E-13					RLN1	
V3SVHSHC_7891844	UBE2E2			1.861E-13					UBE2E2	
V3SVHSHC_6038069	POLR2C			1.870E-13					POLR2C	
V3SVHSHC_6852476	DHDH			1.940E-13					DHDH	
V3SVHSHC_7113209	GJB1			1.940E-13					GJB1	
V3SVHSHC_9637016	CHCHD7			1.977E-13					CHCHD7	
V3SVHSHC_8596592	TNFRSF21			2.003E-13					TNFRSF21	
V3SVHSHC_5821919	ADCY5			2.026E-13					ADCY5	
V3SVHSHC_9888806	TAAR6			2.031E-13					TAAR6	
V3SVHSHC_10019387	STARD10			2.031E-13					STARD10	
V3SVHSHC_10341830	CHRAC1			2.041E-13					CHRAC1	
V3SVHSHC_10436045	GNA11			2.047E-13					GNA11	

V3SVHSHC_6184655	ZNF518A			2.123E-13					ZNF518A	
V3SVHSHC_7148255	HAUS6			2.146E-13					HAUS6	
V3SVHSHC_10113107	ATP6V0E1			2.240E-13					ATP6V0E1	
V3SVHSHC_8409581	SYT13			2.256E-13					SYT13	
V3SVHSHC_9655958	S100A7A			2.272E-13					S100A7A	
V3SVHSHC_9398195	OPTN			2.292E-13					OPTN	
V3SVHSHC_6970253	ZNF17			2.292E-13					ZNF17	
V3SVHSHC_8982197	LOC101060580			2.321E-13					LOC10106 0580	
V3SVHSHC_9962924	PRDM4			2.583E-13					PRDM4	
V3SVHSHC_7669886	QRFPR			2.607E-13					QRFPR	
V3SVHSHC_8455979	CDRT15			2.662E-13					CDRT15	
V3SVHSHC_5195546	TMEM52B			2.740E-13					TMEM52B	
V3SVHSHC_6367079	RPL10			2.742E-13					RPL10	
V3SVHSHC_9194453	CDC42BPG			2.826E-13					CDC42BP G	
V3SVHSHC_4641245	C2orf88			2.849E-13					C2orf88	
V3SVHSHC_6501785	PUM2			2.890E-13	6.852E-10				PUM2	PUM2
V3SVHSHC_7433738	DDX11			2.894E-13					DDX11	
V3SVHSHC_6689324	TPTE			2.956E-13					TPTE	
V3SVHSHC_8302760	PHLDA3			2.992E-13					PHLDA3	
V3SVHSHC_10571543	AARS			3.037E-13					AARS	
V3SVHSHC_5276000	CSNK1D			3.054E-13					CSNK1D	
V3SVHSHC_4763774	OR5M9			3.069E-13					OR5M9	
V3SVHSHC_6823370	CASKIN1			3.077E-13					CASKIN1	
V3SVHSHC_4713647	POLR3F			3.082E-13					POLR3F	
V3SVHSHC_5536469	PPP4R2			3.177E-13					PPP4R2	
V3SVHSHC_8165513	C1orf95			3.201E-13					C1orf95	
V3SVHSHC_5418725	TYROBP			3.209E-13					TYROBP	
V3SVHSHC_5550824	GPA33			3.256E-13					GPA33	
V3SVHSHC_9712751	API5			3.280E-13					API5	
V3SVHSHC_5897918	AMACR			3.293E-13					AMACR	
V3SVHSHC_6270521	VKORC1			3.323E-13	5.039E-02				VKORC1	
V3SVHSHC_5872442	FBN3			3.453E-13					FBN3	
V3SVHSHC_6152084	ZCCHC2			3.583E-13					ZCCHC2	
V3SVHSHC_5190794	SUGP2			3.606E-13					SUGP2	
V3SVHSHC_6326027	OR7D4			3.637E-13					OR7D4	
V3SVHSHC_7435520	ERICH6			3.758E-13					ERICH6	
V3SVHSHC_9130499	ANKS3			3.805E-13					ANKS3	
V3SVHSHC_5747141	ARNT2			3.937E-13					ARNT2	
V3SVHSHC_9464855	TRIOBP			4.096E-13					TRIOBP	
V3SVHSHC_9047042	ANXA7			4.117E-13					ANXA7	
V3SVHSHC_9210656	HOXB4			4.174E-13	3.435E-15				HOXB4	HOXB4
V3SVHSHC_9162410	LOC100652824			4.295E-13					LOC10065 2824	
V3SVHSHC_8245835	LRRC57			4.489E-13					LRRC57	
V3SVHSHC_9901148	ST6GALNAC1			4.656E-13					ST6GALN AC1	
V3SVHSHC_6146771	PRSS50			4.768E-13					PRSS50	
V3SVHSHC_6309791	ZNF526			4.825E-13					ZNF526	
V3SVHSHC_7452020	ZNF19			4.869E-13					ZNF19	
V3SVHSHC_6294941	Ppp6r2			4.945E-13					Ppp6r2	
V3SVHSHC_7983254	DSG2			5.250E-13					DSG2	
V3SVHSHC_7056053	SELPLG			5.399E-13					SELPLG	
V3SVHSHC_6231284	FNDC3A			5.418E-13					FNDC3A	

V3SVHSHC_4744997	RNF157			5.487E-13					RNF157	
V3SVHSHC_6739649	ZFYVE19			5.553E-13					ZFYVE19	
V3SVHSHC_6802877	FRZB			5.557E-13					FRZB	
V3SVHSHC_7802513	P4HTM			5.569E-13					P4HTM	
V3SVHSHC_9410966	PTPRH			5.638E-13					PTPRH	
V3SVHSHC_10687769	NDUFV1			5.691E-13					NDUFV1	
V3SVHSHC_8330150	TNIK			5.780E-13					TNIK	
V3SVHSHC_6083147	MTA1			5.839E-13					MTA1	
V3SVHSHC_7016651	PRSS46			5.904E-13					PRSS46	
V3SVHSHC_4881683	MAP2K2			5.911E-13					MAP2K2	
V3SVHSHC_7428590	CLVS1			5.983E-13					CLVS1	
V3SVHSHC_6656192	TGS1			6.033E-13					TGS1	
V3SVHSHC_6502775	CYP2A7			6.041E-13					CYP2A7	
V3SVHSHC_4778591	FAM78A			6.175E-13					FAM78A	
V3SVHSHC_5136707	FOXQ1			6.375E-13					FOXQ1	
V3SVHSHC_6067505	INPP5J			6.503E-13					INPP5J	
V3SVHSHC_5650451	PRPSAP2			6.638E-13					PRPSAP2	
V3SVHSHC_9055457	ALKBH8			6.723E-13					ALKBH8	
V3SVHSHC_4841423	BCOR			6.771E-13					BCOR	
V3SVHSHC_5597156	GPR156			6.778E-13					GPR156	
V3SVHSHC_8656949	OR51I1			6.795E-13					OR51I1	
V3SVHSHC_7877621	LPPR3			7.120E-13					LPPR3	
V3SVHSHC_8198975	ADORA2A			7.288E-13					ADORA2A	
V3SVHSHC_7769546	UPK1A			7.318E-13					UPK1A	
V3SVHSHC_4910558	TAAR1			7.379E-13					TAAR1	
V3SVHSHC_7095950	POLR3G			7.629E-13					POLR3G	
V3SVHSHC_7572041	MTHFS			7.693E-13					MTHFS	
V3SVHSHC_8709188	LOC101928006			7.731E-13					LOC10192<br>8006	
V3SVHSHC_6862541	LOC101929792			7.826E-13					LOC10192<br>9792	
V3SVHSHC_10750205	NRDE2			7.982E-13	1.915E-01				NRDE2	
V3SVHSHC_7200164	LRRC8E			8.099E-13					LRRC8E	
V3SVHSHC_5263460	FBXO36			8.582E-13					FBXO36	
V3SVHSHC_8690345	ZNF593			8.950E-13					ZNF593	
V3SVHSHC_5452418	IL6ST			9.267E-13					IL6ST	
V3SVHSHC_8067866	SURF6			9.414E-13					SURF6	
V3SVHSHC_10214813	TTC23			9.607E-13					TTC23	
V3SVHSHC_10230950	UBE3C			1.019E-12					UBE3C	
V3SVHSHC_7085324	XPA			1.050E-12					XPA	
V3SVHSHC_10466042	CDK5R1			1.121E-12					CDK5R1	
V3SVHSHC_8455187	RBM6			1.155E-12					RBM6	
V3SVHSHC_7243988	CDC42SE2			1.165E-12					CDC42SE<br>2	
V3SVHSHC_10113371	ARHGAP22			1.182E-12					ARHGAP2<br>2	
V3SVHSHC_6939827	ZNF16			1.257E-12					ZNF16	
V3SVHSHC_8321438	SLCO1A2			1.278E-12					SLCO1A2	
V3SVHSHC_8166569	ANO6			1.323E-12					ANO6	
V3SVHSHC_9159473	PSMF1			1.362E-12					PSMF1	
V3SVHSHC_8936525	CHST2			1.367E-12					CHST2	
V3SVHSHC_5717606	CEP170B			1.386E-12					CEP170B	
V3SVHSHC_5707541	LOC101929829			1.390E-12					LOC10192<br>9829	
V3SVHSHC_7425620	ARHGEF39			1.442E-12					ARHGEF3<br>9	

V3SVHSHC_10048592	TSPAN18			1.444E-12					TSPAN18	
V3SVHSHC_5384702	ARHGAP42			1.464E-12					ARHGAP4 2	
V3SVHSHC_8295830	OR13H1			1.525E-12					OR13H1	
V3SVHSHC_8258573	PRKG2			1.534E-12					PRKG2	
V3SVHSHC_6416315	SURF1			1.554E-12					SURF1	
V3SVHSHC_6356915	ADAM8			1.559E-12					ADAM8	
V3SVHSHC_6739781	RPP40			1.576E-12					RPP40	
V3SVHSHC_9095948	Tab1			1.597E-12					Tab1	
V3SVHSHC_7251743	PASK			1.683E-12					PASK	
V3SVHSHC_8665331	CYP2J2			1.693E-12					CYP2J2	
V3SVHSHC_6604745	TMEM200C			1.699E-12					TMEM200 C	
V3SVHSHC_7871615	WDSUB1			1.718E-12					WDSUB1	
V3SVHSHC_10207883	NUCB2			1.778E-12					NUCB2	
V3SVHSHC_10370045	EFNB2			1.852E-12					EFNB2	
V3SVHSHC_9583919	KCNN2			1.856E-12					KCNN2	
V3SVHSHC_10303583	OR2A5			1.912E-12					OR2A5	
V3SVHSHC_9812378	TMEM210			1.940E-12					TMEM210	
V3SVHSHC_10375028	DHRS4L2			1.991E-12					DHRS4L2	
V3SVHSHC_5854259	MFI2			2.011E-12					MFI2	
V3SVHSHC_8037275	PCDHGB1			2.015E-12					PCDHGB1	
V3SVHSHC_9889499	EYA3			2.046E-12					EYA3	
V3SVHSHC_5485121	ASB16			2.062E-12					ASB16	
V3SVHSHC_5635898	OR1N1			2.094E-12					OR1N1	
V3SVHSHC_9960119	SERPINB11			2.141E-12					SERPINB1 1	
V3SVHSHC_8685494	VMO1			2.160E-12					VMO1	
V3SVHSHC_8903525	USP14			2.163E-12					USP14	
V3SVHSHC_10457099	SSBP2			2.180E-12					SSBP2	
V3SVHSHC_8045624	TXNDC17			2.186E-12					TXNDC17	
V3SVHSHC_6805121	MEPCE			2.208E-12					MEPCE	
V3SVHSHC_9482708	IRF2BP2			2.275E-12					IRF2BP2	
V3SVHSHC_5469050	FMNL1			2.301E-12					FMNL1	
V3SVHSHC_6256496	CD40			2.351E-12					CD40	
V3SVHSHC_9913061	SLC4A9			2.410E-12					SLC4A9	
V3SVHSHC_4685927	CSF1R			2.542E-12					CSF1R	
V3SVHSHC_7242107	CHDC2			2.549E-12					CHDC2	
V3SVHSHC_5347379	RPS6KB1			2.565E-12					RPS6KB1	
V3SVHSHC_7402751	RAB33A			2.566E-12					RAB33A	
V3SVHSHC_8610683	KPNA3			2.588E-12					KPNA3	
V3SVHSHC_5338469	NUP210			2.730E-12					NUP210	
V3SVHSHC_8150465	SMIM14			2.813E-12					SMIM14	
V3SVHSHC_7360247	C1GALT1			2.872E-12					C1GALT1	
V3SVHSHC_7587386	LOC100506248			2.939E-12					LOC10050 6248	
V3SVHSHC_8722817	WDR6			3.005E-12					WDR6	
V3SVHSHC_7721828	APOLD1			3.021E-12					APOLD1	
V3SVHSHC_9131291	CEP128			3.095E-12					CEP128	
V3SVHSHC_5444036	LOC101930098			3.168E-12					LOC10193 0098	
V3SVHSHC_10578836	SLC7A13			3.240E-12					SLC7A13	
V3SVHSHC_7034108	KRT6A			3.300E-12					KRT6A	
V3SVHSHC_8653220	SH3KBP1			3.349E-12					SH3KBP1	
V3SVHSHC_8454296	KCNJ16			3.353E-12					KCNJ16	

V3SVHSHC_9977180	DDO			3.402E-12					DDO	
V3SVHSHC_7975136	CCDC107			3.402E-12					CCDC107	
V3SVHSHC_7975499	TFB2M			3.406E-12					TFB2M	
V3SVHSHC_5439218	ITPR1			3.518E-12					ITPR1	
V3SVHSHC_8868413	NBPF3			3.547E-12					NBPF3	
V3SVHSHC_9537257	TESK1			3.576E-12					TESK1	
V3SVHSHC_6671636	PTK7			3.614E-12					PTK7	
V3SVHSHC_6298439	MYO5B			3.664E-12					MYO5B	
V3SVHSHC_6625964	USP25			3.673E-12					USP25	
V3SVHSHC_7560722	GRM2			3.771E-12					GRM2	
V3SVHSHC_7802942	TOR1AIP1			3.806E-12					TOR1AIP1	
V3SVHSHC_8740208	ZP2			3.824E-12					ZP2	
V3SVHSHC_8942168	MCPH1			3.843E-12					MCPH1	
V3SVHSHC_8613620	FAIM3			4.110E-12					FAIM3	
V3SVHSHC_7597154	PDZD4			4.153E-12					PDZD4	
V3SVHSHC_8874188	LIPA			4.410E-12					LIPA	
V3SVHSHC_8515643	ACCSL			4.552E-12					ACCSL	
V3SVHSHC_10848050	GAB3			4.558E-12					GAB3	
V3SVHSHC_9953915	DCAF12L2			4.941E-12					DCAF12L2	
V3SVHSHC_5742455	SERBP1			5.018E-12					SERBP1	
V3SVHSHC_8750042	FYB			5.084E-12					FYB	
V3SVHSHC_9488450	AKR1C2			5.105E-12					AKR1C2	
V3SVHSHC_10721000	TOPORS			5.112E-12					TOPORS	
V3SVHSHC_6995465	ZRANB3			5.218E-12					ZRANB3	
V3SVHSHC_8786012	GSG1L			5.226E-12					GSG1L	
V3SVHSHC_4817795	USP33			5.308E-12					USP33	
V3SVHSHC_5923724	RNF181			5.329E-12					RNF181	
V3SVHSHC_9509900	CSAG1			5.411E-12					CSAG1	
V3SVHSHC_5810072	COX7A2			5.611E-12					COX7A2	
V3SVHSHC_10513859	RNF212			5.726E-12					RNF212	
V3SVHSHC_4643984	UTS2B			6.145E-12					UTS2B	
V3SVHSHC_5811788	ACOT7			6.182E-12					ACOT7	
V3SVHSHC_8870459	GTF2H4			6.447E-12					GTF2H4	
V3SVHSHC_7583756	SLC7A6			6.473E-12					SLC7A6	
V3SVHSHC_6152546	NAMPT			6.762E-12					NAMPT	
V3SVHSHC_8543429	DAXX			6.950E-12					DAXX	
V3SVHSHC_10285829	LEFTY1			7.003E-12					LEFTY1	
V3SVHSHC_9828911	ZMYND19			7.238E-12					ZMYND19	
V3SVHSHC_6501422	KCNK3			7.242E-12					KCNK3	
V3SVHSHC_7721267	MILR1			7.445E-12					MILR1	
V3SVHSHC_5523170	MSI2			7.886E-12					MSI2	
V3SVHSHC_7603292	TMEM38B			8.116E-12					TMEM38B	
V3SVHSHC_6560261	SPRY4			8.127E-12					SPRY4	
V3SVHSHC_8178251	MUM1L1			8.409E-12					MUM1L1	
V3SVHSHC_5718002	ORMDL1			8.775E-12					ORMDL1	
V3SVHSHC_10231280	OCSTAMP			9.024E-12					OCSTAMP	
V3SVHSHC_4888118	INTS9			9.110E-12					INTS9	
V3SVHSHC_6581381	DISP2			9.298E-12					DISP2	
V3SVHSHC_4850597	SLC29A3			9.419E-12					SLC29A3	
V3SVHSHC_5553728	Arglu1			9.594E-12					Arglu1	
V3SVHSHC_8178350	TES			9.647E-12					TES	
V3SVHSHC_8203628	THOC6			9.728E-12					THOC6	
V3SVHSHC_8376614	POLR2J2			9.930E-12	2.245E-26				POLR2J2	POLR2J2
V3SVHSHC_5453936	HIVEP2			1.020E-11					HIVEP2	

V3SVHSHC_10833035	WDR72			1.036E-11					WDR72	
V3SVHSHC_10027769	FUS			1.041E-11					FUS	
V3SVHSHC_6769646	SLC35G3			1.049E-11					SLC35G3	
V3SVHSHC_7227752	SLC23A3			1.057E-11	1.679E-01				SLC23A3	
V3SVHSHC_6275636	RAD54L			1.144E-11					RAD54L	
V3SVHSHC_7836833	TMEFF1			1.156E-11					TMEFF1	
V3SVHSHC_9178217	SPATA21			1.167E-11					SPATA21	
V3SVHSHC_5006621	MFSD6			1.171E-11					MFSD6	
V3SVHSHC_8974739	SGSM1			1.197E-11					SGSM1	
V3SVHSHC_7368002	TXNRD3			1.206E-11					TXNRD3	
V3SVHSHC_8290385	BBS5			1.235E-11					BBS5	
V3SVHSHC_6054800	C11orf87			1.244E-11					C11orf87	
V3SVHSHC_5358269	ABR			1.250E-11					ABR	
V3SVHSHC_10042916	LOC101930355			1.279E-11					LOC10193<br>0355	
V3SVHSHC_9823499	ARL17B			1.298E-11	3.018E-01				ARL17B	
V3SVHSHC_5406284	OR5AN1			1.317E-11					OR5AN1	
V3SVHSHC_4781198	BRSK2			1.319E-11					BRSK2	
V3SVHSHC_9190196	FAM25A			1.323E-11	3.280E-01				FAM25A	
V3SVHSHC_9633419	C1orf168			1.327E-11					C1orf168	
V3SVHSHC_8171618	RUSC1			1.372E-11					RUSC1	
V3SVHSHC_7667180	SRGAP2D			1.393E-11					SRGAP2D	
V3SVHSHC_8186765	CYP1A2			1.425E-11					CYP1A2	
V3SVHSHC_9294839	VAT1			1.460E-11					VAT1	
V3SVHSHC_9649061	TMSB10			1.460E-11					TMSB10	
V3SVHSHC_10498151	SMAD7			1.521E-11					SMAD7	
V3SVHSHC_7986323	DOPEY1			1.580E-11					DOPEY1	
V3SVHSHC_8825216	ANGPT1			1.598E-11					ANGPT1	
V3SVHSHC_7994012	FAM110A			1.604E-11					FAM110A	
V3SVHSHC_10325528	UTP6			1.611E-11					UTP6	
V3SVHSHC_7972628	PABPN1			1.612E-11					PABPN1	
V3SVHSHC_9450269	OR13C8			1.668E-11					OR13C8	
V3SVHSHC_9030080	MRGPRD			1.698E-11					MRGPRD	
V3SVHSHC_9005627	CD93			1.738E-11					CD93	
V3SVHSHC_6651044	PRDM7			1.761E-11					PRDM7	
V3SVHSHC_7182443	CEP55			1.799E-11					CEP55	
V3SVHSHC_6495251	FBN2			1.811E-11					FBN2	
V3SVHSHC_10213130	NAT2			1.840E-11					NAT2	
V3SVHSHC_9537884	LILRB2			1.846E-11					LILRB2	
V3SVHSHC_5057111	C12orf43			1.907E-11					C12orf43	
V3SVHSHC_7126376	ADRBK1			1.910E-11					ADRBK1	
V3SVHSHC_10496204	SMARCD2			1.912E-11					SMARCD2	
V3SVHSHC_4934813	IFNA10			1.913E-11					IFNA10	
V3SVHSHC_10371464	IER2			1.949E-11					IER2	
V3SVHSHC_6358697	C10ORF91			1.961E-11					C10ORF9<br>1	
V3SVHSHC_9268340	ACTC1			1.997E-11					ACTC1	
V3SVHSHC_5295404	SIRT7			2.075E-11					SIRT7	
V3SVHSHC_6396482	TCTA			2.169E-11					TCTA	
V3SVHSHC_9878477	CRYGB			2.173E-11					CRYGB	
V3SVHSHC_10748654	SRXN1			2.200E-11					SRXN1	
V3SVHSHC_9838349	UGT1A9			2.218E-11					UGT1A9	
V3SVHSHC_5508551	DAPK1			2.272E-11					DAPK1	
V3SVHSHC_7986983	KCTD21			2.356E-11					KCTD21	
V3SVHSHC_5365991	HAO2			2.386E-11					HAO2	

V3SVHSHC_7353251	LOC646730			2.456E-11					LOC64673<br>0	
V3SVHSHC_5790767	NDUFB3			2.492E-11					NDUFB3	
V3SVHSHC_6337511	TMEM38A			2.536E-11					TMEM38A	
V3SVHSHC_7893230	DYNLRB2			2.663E-11					DYNLRB2	
V3SVHSHC_10822871	KCTD5			2.763E-11					KCTD5	
V3SVHSHC_5409221	HVCN1			2.813E-11					HVCN1	
V3SVHSHC_10422020	GALNT2			2.827E-11					GALNT2	
V3SVHSHC_6017873	SMPD1			2.866E-11					SMPD1	
V3SVHSHC_8971538	NOMO1			2.870E-11					NOMO1	
V3SVHSHC_9681467	MAP1LC3B			2.888E-11					MAP1LC3<br>B	
V3SVHSHC_6661307	KLRC1			2.904E-11					KLRC1	
V3SVHSHC_9323384	CSF2RB			2.940E-11					CSF2RB	
V3SVHSHC_7511981	PDXP			2.944E-11					PDXP	
V3SVHSHC_6659624	UNC13D			2.973E-11					UNC13D	
V3SVHSHC_6643949	NCF1			3.144E-11					NCF1	
V3SVHSHC_4722590	TRAPPC13			3.486E-11					TRAPPC1<br>3	
V3SVHSHC_7777367	ZNF808			3.670E-11					ZNF808	
V3SVHSHC_7905605	ATP13A2			3.683E-11					ATP13A2	
V3SVHSHC_10456208	TMEM100			3.693E-11					TMEM100	
V3SVHSHC_6151853	SLC10A5			3.828E-11					SLC10A5	
V3SVHSHC_10461422	PTCH1			3.854E-11					PTCH1	
V3SVHSHC_6048167	Srrm3			4.073E-11					Srrm3	
V3SVHSHC_7375625	LNP1			4.120E-11					LNP1	
V3SVHSHC_6989261	ZNF146			4.135E-11					ZNF146	
V3SVHSHC_9707867	PPIAL4C			4.172E-11					PPIAL4C	
V3SVHSHC_9741428	DAZ2			4.212E-11					DAZ2	
V3SVHSHC_8368826	CLTC			4.214E-11					CLTC	
V3SVHSHC_7480961	GPBP1			4.228E-11					GPBP1	
V3SVHSHC_8658368	SMCO2			4.334E-11					SMCO2	
V3SVHSHC_10450829	CCDC85A			4.394E-11					CCDC85A	
V3SVHSHC_8340611	PRAMEF6			4.428E-11					PRAMEF6	
V3SVHSHC_8439314	RNF4			4.525E-11					RNF4	
V3SVHSHC_5160467	C20orf196			4.549E-11					C20orf196	
V3SVHSHC_7893296	ERICH4			4.791E-11					ERICH4	
V3SVHSHC_8701103	SSX2			4.972E-11					SSX2	
V3SVHSHC_7510760	PPM1M			5.196E-11					PPM1M	
V3SVHSHC_6379223	PARL			5.209E-11					PARL	
V3SVHSHC_6263327	JADE2			5.264E-11					JADE2	
V3SVHSHC_5175746	FILIP1L			5.780E-11					FILIP1L	
V3SVHSHC_8490233	CERKL			6.105E-11					CERKL	
V3SVHSHC_9984638	KCMF1			6.109E-11					KCMF1	
V3SVHSHC_9661172	KRT34			6.366E-11					KRT34	
V3SVHSHC_10834091	HRH2			6.520E-11					HRH2	
V3SVHSHC_4800569	ITGB1BP2			6.568E-11					ITGB1BP2	
V3SVHSHC_10433900	USP25			6.643E-11					USP25	
V3SVHSHC_7584383	TACR2			6.876E-11					TACR2	
V3SVHSHC_8015363	LGALS7			7.144E-11					LGALS7	
V3SVHSHC_5898050	TRPV3			7.168E-11					TRPV3	
V3SVHSHC_9836633	CALHM1			7.273E-11					CALHM1	
V3SVHSHC_10102778	ITGA7			7.312E-11					ITGA7	
V3SVHSHC_10211414	DIDO1			7.396E-11					DIDO1	
V3SVHSHC_5400080	TCN1			7.658E-11					TCN1	

V3SVHSHC_9567485	SRGAP3			7.666E-11					SRGAP3	
V3SVHSHC_5888084	ZDHHC8			8.006E-11					ZDHHC8	
V3SVHSHC_5658437	PARD3B			8.131E-11					PARD3B	
V3SVHSHC_9698297	TMEM117			8.133E-11					TMEM117	
V3SVHSHC_10358891	ADIPOR1			8.398E-11					ADIPOR1	
V3SVHSHC_6774728	DDX43			9.082E-11					DDX43	
V3SVHSHC_9891050	SOCS7			9.194E-11					SOCS7	
V3SVHSHC_6975995	UBE3B			9.223E-11					UBE3B	
V3SVHSHC_6358598	ZNF593			9.490E-11					ZNF593	
V3SVHSHC_5614580	SPINK6			9.503E-11					SPINK6	
V3SVHSHC_10649852	TNFRSF6B			9.641E-11					TNFRSF6 B	
V3SVHSHC_7551581	IGSF23			9.647E-11					IGSF23	
V3SVHSHC_9083012	NPY5R			1.031E-10					NPY5R	
V3SVHSHC_9420173	GRN			1.051E-10					GRN	
V3SVHSHC_4955867	TFB2M			1.087E-10					TFB2M	
V3SVHSHC_9702554	C9ORF89			1.101E-10					C9ORF89	
V3SVHSHC_10552370	COX5A			1.104E-10					COX5A	
V3SVHSHC_9885407	OSBPL7			1.109E-10					OSBPL7	
V3SVHSHC_7596230	C15orf54			1.195E-10					C15orf54	
V3SVHSHC_8853200	RPS6KA1			1.198E-10					RPS6KA1	
V3SVHSHC_8438951	TRIM37			1.203E-10					TRIM37	
V3SVHSHC_8682887	C15orf57			1.205E-10					C15orf57	
V3SVHSHC_6696188	CALML5			1.209E-10					CALML5	
V3SVHSHC_9125912	KRBA2			1.258E-10					KRBA2	
V3SVHSHC_9020807	VPS45			1.299E-10					VPS45	
V3SVHSHC_9152213	C12orf80			1.327E-10					C12orf80	
V3SVHSHC_7089911	DYDC1			1.337E-10					DYDC1	
V3SVHSHC_10829207	C1R			1.360E-10					C1R	
V3SVHSHC_4887458	GAL3ST2			1.378E-10					GAL3ST2	
V3SVHSHC_5265044	EPHA1			1.385E-10					EPHA1	
V3SVHSHC_7434200	CNGB1			1.399E-10					CNGB1	
V3SVHSHC_9278900	NXPH2			1.424E-10					NXPH2	
V3SVHSHC_7678367	ICT1			1.457E-10					ICT1	
V3SVHSHC_6958307	HSPA1B			1.474E-10					HSPA1B	
V3SVHSHC_4893926	RSPO3			1.477E-10					RSPO3	
V3SVHSHC_9098324	APOH			1.500E-10					APOH	
V3SVHSHC_7787036	RETNLB			1.521E-10					RETNLB	
V3SVHSHC_6872771	SLC25A23			1.555E-10					SLC25A23	
V3SVHSHC_5774135	PHKG2			1.616E-10					PHKG2	
V3SVHSHC_10173233	BTN3A3			1.690E-10					BTN3A3	
V3SVHSHC_9603983	GCM2			1.735E-10					GCM2	
V3SVHSHC_5759153	KLRF2			1.756E-10					KLRF2	
V3SVHSHC_10020608	CCDC174			1.865E-10					CCDC174	
V3SVHSHC_8520230	DAZ2			1.897E-10					DAZ2	
V3SVHSHC_9672458	MCM2			1.904E-10					MCM2	
V3SVHSHC_8375129	OSBPL7			1.909E-10					OSBPL7	
V3SVHSHC_6596957	PUSL1			1.915E-10					PUSL1	
V3SVHSHC_10409909	OR51B6			1.973E-10					OR51B6	
V3SVHSHC_7990184	GNA12			1.976E-10					GNA12	
V3SVHSHC_5576795	HOXC6			1.987E-10					HOXC6	
V3SVHSHC_8307413	ZNF324B			2.029E-10					ZNF324B	
V3SVHSHC_8507525	SLC25A24			2.035E-10					SLC25A24	
V3SVHSHC_10804490	SNTG2			2.051E-10					SNTG2	
V3SVHSHC_7918013	KLHL42			2.051E-10					KLHL42	

V3SVHSHC_5391896	SLC1A5			2.082E-10					SLC1A5	
V3SVHSHC_8383247	ZBTB37			2.105E-10					ZBTB37	
V3SVHSHC_6344276	CDK3			2.154E-10					CDK3	
V3SVHSHC_5135915	TRIM21			2.211E-10					TRIM21	
V3SVHSHC_10344899	PRNP			2.222E-10					PRNP	
V3SVHSHC_5502248	DRC1			2.364E-10					DRC1	
V3SVHSHC_9214088	NR5A2			2.438E-10					NR5A2	
V3SVHSHC_8967314	MGAT3			2.472E-10					MGAT3	
V3SVHSHC_4695827	LOC441239			2.473E-10					LOC44123 9	
V3SVHSHC_10346813	DPF3			2.508E-10					DPF3	
V3SVHSHC_9632660	SAFB2			2.548E-10					SAFB2	
V3SVHSHC_8838746	Msl2			2.574E-10					Msl2	
V3SVHSHC_8518217	LRP2BP			2.747E-10					LRP2BP	
V3SVHSHC_6827891	ICAM4			2.781E-10					ICAM4	
V3SVHSHC_6851684	SFXN2			2.825E-10					SFXN2	
V3SVHSHC_8769578	Zc3h18			2.837E-10					Zc3h18	
V3SVHSHC_6200594	Erlec1			2.839E-10					Erlec1	
V3SVHSHC_9843728	ARMC9			2.839E-10					ARMC9	
V3SVHSHC_8453867	APAF1			2.841E-10					APAF1	
V3SVHSHC_8315366	GLP2R			2.859E-10					GLP2R	
V3SVHSHC_5689820	TDP2			2.899E-10					TDP2	
V3SVHSHC_6256760	SCN2A			2.982E-10					SCN2A	
V3SVHSHC_7805978	SPINK2			3.041E-10					SPINK2	
V3SVHSHC_10516598	LRRC23			3.060E-10					LRRC23	
V3SVHSHC_7387142	TNIK			3.079E-10					TNIK	
V3SVHSHC_4861685	NLGN4X			3.183E-10					NLGN4X	
V3SVHSHC_7195346	VPS36			3.206E-10					VPS36	
V3SVHSHC_10693907	PNMAL1			3.240E-10					PNMAL1	
V3SVHSHC_8972759	SVIP			3.250E-10					SVIP	
V3SVHSHC_7464230	TAF7			3.266E-10					TAF7	
V3SVHSHC_10400174	PSD2			3.273E-10					PSD2	
V3SVHSHC_9034832	FLYWCH1			3.346E-10					FLYWCH1	
V3SVHSHC_10381694	CD2AP			3.365E-10					CD2AP	
V3SVHSHC_5813669	FOXK1			3.430E-10					FOXK1	
V3SVHSHC_10373972	KIAA1841			3.447E-10					KIAA1841	
V3SVHSHC_6630617	CLCN4			3.481E-10					CLCN4	
V3SVHSHC_10845773	DNAJA2			3.518E-10					DNAJA2	
V3SVHSHC_6492710	OTUB2			3.545E-10					OTUB2	
V3SVHSHC_7228709	C2cd2			3.556E-10					C2cd2	
V3SVHSHC_10432250	TXNDC11			3.680E-10					TXNDC11	
V3SVHSHC_8472974	ASB17			3.856E-10					ASB17	
V3SVHSHC_9297512	Ddrgk1			3.876E-10					Ddrgk1	
V3SVHSHC_10844420	LRRC38			3.915E-10					LRRC38	
V3SVHSHC_5303159	UPF3A			3.994E-10					UPF3A	
V3SVHSHC_8881448	SMARCA5			4.038E-10					SMARCA5	
V3SVHSHC_6731102	MAGEA9			4.050E-10					MAGEA9	
V3SVHSHC_5615372	TIAF1			4.083E-10					TIAF1	
V3SVHSHC_10685558	CRB1			4.138E-10					CRB1	
V3SVHSHC_5194919	CD4			4.231E-10					CD4	
V3SVHSHC_6176174	PTTG2			4.286E-10					PTTG2	
V3SVHSHC_5397176	TAOK2			4.333E-10					TAOK2	
V3SVHSHC_7299296	AIMP2			4.375E-10					AIMP2	
V3SVHSHC_6209735	KIAA0754			4.395E-10					KIAA0754	
V3SVHSHC_6774332	HEATR4			4.465E-10					HEATR4	

V3SVHSHC_7027772	KIF28P			4.622E-10					KIF28P	
V3SVHSHC_10748357	DNAJA1			5.086E-10					DNAJA1	
V3SVHSHC_5322497	GBP2			5.338E-10					GBP2	
V3SVHSHC_6963224	ZNF668			5.444E-10					ZNF668	
V3SVHSHC_8051630	CDKN3			5.887E-10					CDKN3	
V3SVHSHC_5185217	ADAMTSL2			5.904E-10					ADAMTSL2	
V3SVHSHC_5876633	SYCP2			5.943E-10					SYCP2	
V3SVHSHC_6270422	SLC27A6			5.971E-10					SLC27A6	
V3SVHSHC_7456409	GDE1			5.971E-10					GDE1	
V3SVHSHC_7145516	CPNE5			5.995E-10					CPNE5	
V3SVHSHC_7420439	SEMA7A			6.012E-10					SEMA7A	
V3SVHSHC_6198647	SUN1			6.226E-10					SUN1	
V3SVHSHC_9586130	CD302			6.268E-10					CD302	
V3SVHSHC_8485448	TMEM183A			6.296E-10					TMEM183A	
V3SVHSHC_10267679	Rrp7a			6.367E-10					Rrp7a	
V3SVHSHC_10480298	SEMG2			6.710E-10					SEMG2	
V3SVHSHC_6562505	FOXF2			6.853E-10					FOXF2	
V3SVHSHC_6842213	PDZD3			7.207E-10	6.411E-06				PDZD3	PDZD3
V3SVHSHC_9992261	GSDMA			7.360E-10					GSDMA	
V3SVHSHC_8631803	BTNL10			7.369E-10					BTNL10	
V3SVHSHC_6639791	ING1			7.370E-10					ING1	
V3SVHSHC_8468519	TSPAN31			7.385E-10					TSPAN31	
V3SVHSHC_10404629	KPNA6			7.828E-10					KPNA6	
V3SVHSHC_5219075	CHST10			8.006E-10					CHST10	
V3SVHSHC_8220326	SLMAP			8.320E-10					SLMAP	
V3SVHSHC_4686290	DNAJC22			8.768E-10					DNAJC22	
V3SVHSHC_7823105	ZIK1			8.871E-10					ZIK1	
V3SVHSHC_8028695	ZNF34			9.639E-10					ZNF34	
V3SVHSHC_8888081	CIART			1.072E-09					CIART	
V3SVHSHC_6670448	LOC285500			1.083E-09					LOC285500	
V3SVHSHC_7966589	CXCR4			1.275E-09					CXCR4	
V3SVHSHC_5625470	ROR1			1.284E-09					ROR1	
V3SVHSHC_8473535	POMZP3			1.318E-09					POMZP3	
V3SVHSHC_5250689	MRGPRX1			1.361E-09					MRGPRX1	
V3SVHSHC_6486902	MFAP5			1.366E-09					MFAP5	
V3SVHSHC_6844490	HOXC5			1.369E-09					HOXC5	
V3SVHSHC_10487855	SYNGAP1			1.375E-09					SYNGAP1	
V3SVHSHC_5422058	EIF2AK2			1.384E-09					EIF2AK2	
V3SVHSHC_4782749	KIAA1045			1.423E-09					KIAA1045	
V3SVHSHC_6467498	PRMT8			1.439E-09					PRMT8	
V3SVHSHC_9988895	ZNF600			1.475E-09					ZNF600	
V3SVHSHC_7309922	TSSC4			1.506E-09					TSSC4	
V3SVHSHC_5529242	UQCC1			1.528E-09					UQCC1	
V3SVHSHC_6379685	SLITRK4			1.542E-09					SLITRK4	
V3SVHSHC_5405261	LOC100129361			1.752E-09					LOC100129361	
V3SVHSHC_8774099	TAS2R10			1.762E-09					TAS2R10	
V3SVHSHC_8174357	SEMA3G			1.767E-09					SEMA3G	
V3SVHSHC_7838582	OXSR1			1.770E-09					OXSR1	
V3SVHSHC_10481750	ZXDB			1.850E-09					ZXDB	
V3SVHSHC_9877091	FAM98A			1.947E-09					FAM98A	
V3SVHSHC_4763213	YKT6			2.001E-09					YKT6	

V3SVHSHC_8762978	LRRC24			2.132E-09					LRRC24	
V3SVHSHC_10152278	ISX			2.271E-09					ISX	
V3SVHSHC_7969922	CT45A2			2.299E-09					CT45A2	
V3SVHSHC_6075458	METTL4			2.304E-09					METTL4	
V3SVHSHC_7472216	PLS1			2.307E-09					PLS1	
V3SVHSHC_9862538	CDY2A			2.382E-09					CDY2A	
V3SVHSHC_10824257	TRPM7			2.415E-09					TRPM7	
V3SVHSHC_10776209	LOC101929627			2.509E-09					LOC10192 9627	
V3SVHSHC_7384568	ITGA4			2.605E-09					ITGA4	
V3SVHSHC_4692989	DYNC2H1			2.622E-09					DYNC2H1	
V3SVHSHC_7016849	SLC30A1			2.627E-09					SLC30A1	
V3SVHSHC_6545939	ASB18			2.630E-09					ASB18	
V3SVHSHC_4906433	TJP1			2.714E-09					TJP1	
V3SVHSHC_6869603	SLCO1B7			2.802E-09					SLCO1B7	
V3SVHSHC_5923658	ZNF519			2.942E-09					ZNF519	
V3SVHSHC_9572039	C1QTNF5			3.030E-09					C1QTNF5	
V3SVHSHC_9426047	TMEM54			3.288E-09					TMEM54	
V3SVHSHC_6028301	LOC101929587			3.297E-09					LOC10192 9587	
V3SVHSHC_9587516	RRH			3.677E-09					RRH	
V3SVHSHC_5622071	TTC32			3.688E-09					TTC32	
V3SVHSHC_9733838	TC2N			3.882E-09					TC2N	
V3SVHSHC_4921976	LOC101929571			3.901E-09					LOC10192 9571	
V3SVHSHC_10109114	ACTA1			3.952E-09					ACTA1	
V3SVHSHC_7079252	WASF3			4.053E-09					WASF3	
V3SVHSHC_4642796	ACTR6			4.188E-09					ACTR6	
V3SVHSHC_8933192	CXorf58			4.206E-09					CXorf58	
V3SVHSHC_9500231	DTWD1			4.293E-09					DTWD1	
V3SVHSHC_7643816	SGK2			4.467E-09					SGK2	
V3SVHSHC_8596427	NANS			4.496E-09					NANS	
V3SVHSHC_5069981	LPHN3			4.624E-09					LPHN3	
V3SVHSHC_9914018	PLVAP			4.843E-09					PLVAP	
V3SVHSHC_9437102	CLEC4M			4.988E-09					CLEC4M	
V3SVHSHC_4708532	RBL1			5.012E-09					RBL1	
V3SVHSHC_5813042	ZNF37A			5.209E-09					ZNF37A	
V3SVHSHC_8445155	CIART			5.259E-09	9.598E-14				CIART	CIART
V3SVHSHC_9256922	KCNMB3			5.551E-09					KCNMB3	
V3SVHSHC_6561416	DCLRE1B			5.563E-09					DCLRE1B	
V3SVHSHC_9392717	GJA8			5.575E-09					GJA8	
V3SVHSHC_5458028	CALHM3			5.821E-09					CALHM3	
V3SVHSHC_7844159	IL2RA			5.862E-09					IL2RA	
V3SVHSHC_9764363	LRRC27			6.088E-09					LRRC27	
V3SVHSHC_8226662	ZCRB1			6.159E-09					ZCRB1	
V3SVHSHC_5035397	ULK3			6.214E-09					ULK3	
V3SVHSHC_6962003	ISY1			6.264E-09					ISY1	
V3SVHSHC_9960680	SCNN1D			6.495E-09					SCNN1D	
V3SVHSHC_6477398	GPRASP1			6.566E-09					GPRASP1	
V3SVHSHC_7918838	PYROXD2			6.759E-09					PYROXD2	
V3SVHSHC_9713972	OR5AN1			7.033E-09					OR5AN1	
V3SVHSHC_9824357	PCBP2			7.216E-09					PCBP2	
V3SVHSHC_7384403	DUSP26			7.348E-09					DUSP26	
V3SVHSHC_8244911	AMY1C			7.508E-09					AMY1C	
V3SVHSHC_5573198	CTBS			7.606E-09					CTBS	

V3SVHSHC_9620813	MAK			8.353E-09					MAK	
V3SVHSHC_7029422	NOL9			8.446E-09					NOL9	
V3SVHSHC_9799442	MLLT6			8.699E-09					MLLT6	
V3SVHSHC_8258870	POLN			8.919E-09					POLN	
V3SVHSHC_4712459	MRPL4			8.977E-09					MRPL4	
V3SVHSHC_5561912	NDUFAF3			9.040E-09					NDUFAF3	
V3SVHSHC_4932173	KIAA1429			9.229E-09					KIAA1429	
V3SVHSHC_10832045	SAPCD2			9.514E-09					SAPCD2	
V3SVHSHC_5922239	IFNL4			9.932E-09					IFNL4	
V3SVHSHC_6681965	SAMD12			1.023E-08	4.972E-01				SAMD12	
V3SVHSHC_5880164	HES5			1.062E-08					HES5	
V3SVHSHC_10318499	PRR22			1.071E-08					PRR22	
V3SVHSHC_10351334	PPP6R1			1.089E-08					PPP6R1	
V3SVHSHC_10667771	PRKX			1.091E-08					PRKX	
V3SVHSHC_9087566	TRIM56			1.107E-08					TRIM56	
V3SVHSHC_8571875	C19ORF26			1.153E-08					C19ORF2<br>6	
V3SVHSHC_10222436	OR8G2			1.202E-08					OR8G2	
V3SVHSHC_6525644	MET			1.234E-08					MET	
V3SVHSHC_4703318	TMEM216			1.265E-08					TMEM216	
V3SVHSHC_9137792	OTUD6B			1.276E-08					OTUD6B	
V3SVHSHC_9323483	ERLIN2			1.279E-08					ERLIN2	
V3SVHSHC_8906627	OR4C16			1.332E-08					OR4C16	
V3SVHSHC_8949296	NDUFS3			1.387E-08					NDUFS3	
V3SVHSHC_7376384	OR2Z1			1.394E-08					OR2Z1	
V3SVHSHC_5612798	PDCL2			1.443E-08					PDCL2	
V3SVHSHC_5628605	AAR2			1.447E-08					AAR2	
V3SVHSHC_9561215	LDB2			1.490E-08					LDB2	
V3SVHSHC_8898245	AFF3			1.498E-08					AFF3	
V3SVHSHC_7500068	PIK3R5			1.511E-08					PIK3R5	
V3SVHSHC_6162248	MIPEP			1.562E-08					MIPEP	
V3SVHSHC_8647610	RRP36			1.575E-08					RRP36	
V3SVHSHC_5942567	BEST1			1.771E-08					BEST1	
V3SVHSHC_10382651	NXT2			1.871E-08	2.249E-01				NXT2	
V3SVHSHC_6262601	CRK			1.884E-08					CRK	
V3SVHSHC_6865478	GRXCR1			1.894E-08					GRXCR1	
V3SVHSHC_6593822	ZKSCAN2			1.898E-08					ZKSCAN2	
V3SVHSHC_7785254	SMR3B			2.042E-08					SMR3B	
V3SVHSHC_7759712	ALKBH6			2.045E-08					ALKBH6	
V3SVHSHC_8353052	GAS2L1			2.066E-08					GAS2L1	
V3SVHSHC_7367705	FANCL			2.229E-08					FANCL	
V3SVHSHC_5899205	CT47B1			2.256E-08					CT47B1	
V3SVHSHC_6412256	DCHS1			2.305E-08					DCHS1	
V3SVHSHC_6637844	ZNF485			2.311E-08					ZNF485	
V3SVHSHC_6558347	C3AR1			2.340E-08					C3AR1	
V3SVHSHC_6430835	UNC5D			2.343E-08					UNC5D	
V3SVHSHC_8188514	Carkd			2.476E-08					Carkd	
V3SVHSHC_10301174	CLEC4E			2.493E-08					CLEC4E	
V3SVHSHC_7223561	CORO1B			2.513E-08					CORO1B	
V3SVHSHC_8846501	FCGR3A			2.523E-08					FCGR3A	
V3SVHSHC_6578609	UPF2			2.558E-08					UPF2	
V3SVHSHC_10815974	LMAN2L			2.627E-08					LMAN2L	
V3SVHSHC_6188153	GRXCR2			2.727E-08					GRXCR2	
V3SVHSHC_6405986	SLC35D1			2.782E-08					SLC35D1	
V3SVHSHC_9567716	ZNF300			2.794E-08					ZNF300	

V3SVHSHC_5575277	SMIM3			2.837E-08					SMIM3	
V3SVHSHC_6635435	RPL9			2.844E-08					RPL9	
V3SVHSHC_7028399	AKAP13			2.901E-08					AKAP13	
V3SVHSHC_5432321	CNIH1			3.015E-08					CNIH1	
V3SVHSHC_5354078	BOLA1			3.037E-08					BOLA1	
V3SVHSHC_7995299	ORAI1			3.114E-08					ORAI1	
V3SVHSHC_8242073	OTUD5			3.135E-08	3.589E-01				OTUD5	
V3SVHSHC_8618339	FAM76B			3.237E-08					FAM76B	
V3SVHSHC_7215608	HIF3A			3.242E-08					HIF3A	
V3SVHSHC_9111590	CLCNKB			3.400E-08	1.469E-11				CLCNKB	CLCNKB
V3SVHSHC_5280125	GPATCH11			3.449E-08					GPATCH11	
V3SVHSHC_8651570	LOC100507462			3.457E-08					LOC100507462	
V3SVHSHC_6467102	LOC101928548			3.580E-08					LOC101928548	
V3SVHSHC_4761629	PGAM5			3.593E-08					PGAM5	
V3SVHSHC_8445485	Ccdc138			3.597E-08					Ccdc138	
V3SVHSHC_8562635	CHST8			3.741E-08					CHST8	
V3SVHSHC_7566992	PRDX4			3.844E-08					PRDX4	
V3SVHSHC_9677078	SAMD11			3.884E-08					SAMD11	
V3SVHSHC_7044305	MTRNR2L6			3.991E-08					MTRNR2L6	
V3SVHSHC_5981837	DDX26B			4.037E-08					DDX26B	
V3SVHSHC_7066712	USP14			4.326E-08					USP14	
V3SVHSHC_8346419	ZNF557			4.374E-08					ZNF557	
V3SVHSHC_7768556	C17orf75			4.528E-08					C17orf75	
V3SVHSHC_8423144	RPTOR			4.811E-08					RPTOR	
V3SVHSHC_8116343	KLHL28			4.823E-08					KLHL28	
V3SVHSHC_8214914	Celf4			4.930E-08					Celf4	
V3SVHSHC_7572800	ZNF200			5.708E-08					ZNF200	
V3SVHSHC_9167195	NTF4			5.732E-08					NTF4	
V3SVHSHC_8279264	KDM8			6.241E-08					KDM8	
V3SVHSHC_5258840	BTG2			6.375E-08					BTG2	
V3SVHSHC_10538246	LAMP2			6.445E-08					LAMP2	
V3SVHSHC_5214224	ARPC1B			6.454E-08					ARPC1B	
V3SVHSHC_8250323	TTLL5			7.044E-08					TTLL5	
V3SVHSHC_4988570	STAT5A			7.156E-08					STAT5A	
V3SVHSHC_9658070	NANOG			7.213E-08					NANOG	
V3SVHSHC_4863302	PHYHIP			7.289E-08	1.795E-14				PHYHIP	PHYHIP
V3SVHSHC_9508448	SCD5			7.538E-08					SCD5	
V3SVHSHC_7549964	CSN2			7.642E-08					CSN2	
V3SVHSHC_5013914	HBD			8.280E-08					HBD	
V3SVHSHC_6008402	TTR			8.378E-08					TTR	
V3SVHSHC_5994179	CHRNA6			8.387E-08					CHRNA6	
V3SVHSHC_9062486	ADHFE1			8.554E-08					ADHFE1	
V3SVHSHC_10001105	EIF4E3			8.563E-08					EIF4E3	
V3SVHSHC_8795054	TUSC3			8.596E-08					TUSC3	
V3SVHSHC_7750835	ZSCAN9			8.653E-08					ZSCAN9	
V3SVHSHC_6424796	SLC22A6			8.799E-08					SLC22A6	
V3SVHSHC_4643786	GLT1D1			8.918E-08					GLT1D1	
V3SVHSHC_5749352	SAR1B			9.325E-08					SAR1B	
V3SVHSHC_6152414	TCEAL6			9.372E-08					TCEAL6	
V3SVHSHC_6098063	ALOX5AP			9.688E-08					ALOX5AP	
V3SVHSHC_9383444	ANKRD35			1.012E-07					ANKRD35	

V3SVHSHC_9362852	DRD5			1.014E-07					DRD5	
V3SVHSHC_7505183	ZNF716			1.045E-07					ZNF716	
V3SVHSHC_10564844	SLC28A1			1.064E-07					SLC28A1	
V3SVHSHC_7576364	ZRANB3			1.114E-07					ZRANB3	
V3SVHSHC_5009162	ATP5S			1.132E-07					ATP5S	
V3SVHSHC_8787464	EPO			1.171E-07					EPO	
V3SVHSHC_7522541	LRRC23			1.172E-07					LRRC23	
V3SVHSHC_9061562	NLRP11			1.251E-07					NLRP11	
V3SVHSHC_10309490	OR51M1			1.265E-07					OR51M1	
V3SVHSHC_5656919	KLRC4-KLRK1			1.377E-07					KLRC4-<br>KLRK1	
V3SVHSHC_9992393	CTPS2			1.490E-07					CTPS2	
V3SVHSHC_4946924	RPA4			1.539E-07					RPA4	
V3SVHSHC_10610615	PTGDS			1.560E-07					PTGDS	
V3SVHSHC_7916264	TGM7			1.584E-07					TGM7	
V3SVHSHC_7529141	CYP1B1			1.604E-07					CYP1B1	
V3SVHSHC_4724405	FAM133A			1.613E-07					FAM133A	
V3SVHSHC_5822942	CATSPERG			1.624E-07					CATSPER<br>G	
V3SVHSHC_7534388	INTS3			1.627E-07					INTS3	
V3SVHSHC_5568875	USH1C			1.692E-07					USH1C	
V3SVHSHC_7564616	THBS3			1.749E-07					THBS3	
V3SVHSHC_6360941	BAIAP3			1.783E-07					BAIAP3	
V3SVHSHC_9905438	MFSD2B			1.799E-07					MFSD2B	
V3SVHSHC_7846931	MORC2			1.820E-07					MORC2	
V3SVHSHC_9004142	STRA13			1.829E-07					STRA13	
V3SVHSHC_5730212	RIC3			1.877E-07					RIC3	
V3SVHSHC_9896627	NDUFA7			1.912E-07					NDUFA7	
V3SVHSHC_8139311	CTAGE9			1.946E-07					CTAGE9	
V3SVHSHC_7528547	TM2D1			1.960E-07					TM2D1	
V3SVHSHC_6529010	FUOM			1.973E-07					FUOM	
V3SVHSHC_8011106	LOC441155			2.157E-07					LOC44115<br>5	
V3SVHSHC_10229432	THAP2			2.217E-07					THAP2	
V3SVHSHC_5208020	TEF			2.239E-07					TEF	
V3SVHSHC_10732187	ANGEL2			2.258E-07					ANGEL2	
V3SVHSHC_9395588	FAM110C			2.269E-07					FAM110C	
V3SVHSHC_8367968	REPS2			2.289E-07					REPS2	
V3SVHSHC_10261805	OR13G1			2.348E-07					OR13G1	
V3SVHSHC_8411924	Mfsd10			2.350E-07					Mfsd10	
V3SVHSHC_8384435	TNKS2			2.397E-07					TNKS2	
V3SVHSHC_7287515	ABR			2.498E-07					ABR	
V3SVHSHC_9197621	TMC1			2.586E-07					TMC1	
V3SVHSHC_6021140	P2RX7			2.610E-07					P2RX7	
V3SVHSHC_10582268	LSMEM2			2.628E-07					LSMEM2	
V3SVHSHC_7693712	ACE2			2.629E-07					ACE2	
V3SVHSHC_7807067	LOC101929983			2.731E-07					LOC10192<br>9983	
V3SVHSHC_7710080	OR4E2			2.740E-07					OR4E2	
V3SVHSHC_8287448	FRMD3			2.775E-07					FRMD3	
V3SVHSHC_5676389	DSCAM			2.966E-07					DSCAM	
V3SVHSHC_10717865	PRDM15			2.975E-07					PRDM15	
V3SVHSHC_7907618	VRTN			2.976E-07					VRTN	
V3SVHSHC_9696152	BCL9L			3.097E-07					BCL9L	
V3SVHSHC_6464330	AHSG			3.101E-07					AHSG	

V3SVHSHC_9836534	ATP2C2			3.299E-07					ATP2C2	
V3SVHSHC_6739715	DOCK1			3.387E-07					DOCK1	
V3SVHSHC_6608738	STX17			3.611E-07					STX17	
V3SVHSHC_6761891	DNASE2			3.690E-07					DNASE2	
V3SVHSHC_10080602	GPR88			3.773E-07					GPR88	
V3SVHSHC_7516535	NAT1			3.794E-07					NAT1	
V3SVHSHC_5957384	NEUROD4			3.845E-07					NEUROD4	
V3SVHSHC_6436214	ATP4B			3.861E-07					ATP4B	
V3SVHSHC_10568144	ZNF25			3.918E-07					ZNF25	
V3SVHSHC_5865116	TMEM216			4.001E-07					TMEM216	
V3SVHSHC_5498222	C18orf56			4.156E-07					C18orf56	
V3SVHSHC_7164326	ZNF628			4.338E-07					ZNF628	
V3SVHSHC_4993487	MRPL14			4.372E-07					MRPL14	
V3SVHSHC_9973946	ZMYND10			4.466E-07					ZMYND10	
V3SVHSHC_5404073	CD82			4.473E-07					CD82	
V3SVHSHC_8491124	HEMGN			4.495E-07					HEMGN	
V3SVHSHC_4786379	PDDC1			4.724E-07					PDDC1	
V3SVHSHC_9945863	MROH2A			5.218E-07					MROH2A	
V3SVHSHC_8038397	VWA8			5.368E-07					VWA8	
V3SVHSHC_5429780	EPHB2			5.757E-07					EPHB2	
V3SVHSHC_5514293	TLE4			5.823E-07					TLE4	
V3SVHSHC_4793408	AMFR			5.860E-07					AMFR	
V3SVHSHC_8382752	CCNDBP1			5.992E-07					CCNDBP1	
V3SVHSHC_7670051	NOS1			6.011E-07					NOS1	
V3SVHSHC_7854851	IL15RA			6.142E-07					IL15RA	
V3SVHSHC_6677345	TSPAN12			6.804E-07					TSPAN12	
V3SVHSHC_9523727	RUNX3			7.062E-07					RUNX3	
V3SVHSHC_7383050	CDK2			7.120E-07					CDK2	
V3SVHSHC_10152905	POP5			7.578E-07					POP5	
V3SVHSHC_8771393	F13B			7.902E-07					F13B	
V3SVHSHC_7334309	HHLA3			7.910E-07					HHLA3	
V3SVHSHC_5486144	PLEKHG4B			8.061E-07					PLEKHG4<br>B	
V3SVHSHC_6693251	PROK2			8.269E-07					PROK2	
V3SVHSHC_10552931	LAMA2			8.285E-07					LAMA2	
V3SVHSHC_10285928	UBE2NL			8.725E-07					UBE2NL	
V3SVHSHC_7876565	PPM1G			8.999E-07					PPM1G	
V3SVHSHC_8076281	FAM160A1			9.077E-07					FAM160A1	
V3SVHSHC_8376350	GAB1			9.216E-07					GAB1	
V3SVHSHC_8182541	SPDYE4			9.458E-07					SPDYE4	
V3SVHSHC_8875244	MPRIP			9.939E-07					MPRIP	
V3SVHSHC_9162377	SLC25A47			1.025E-06					SLC25A47	
V3SVHSHC_10328135	DHRS3			1.031E-06					DHRS3	
V3SVHSHC_9486272	MYZAP			1.040E-06					MYZAP	
V3SVHSHC_7271840	ITGA8			1.075E-06					ITGA8	
V3SVHSHC_10615268	PRKX			1.081E-06					PRKX	
V3SVHSHC_5762090	CCNY			1.111E-06					CCNY	
V3SVHSHC_8838779	POLR3GL			1.122E-06					POLR3GL	
V3SVHSHC_5179112	MX1			1.165E-06					MX1	
V3SVHSHC_8663648	GSG1			1.206E-06					GSG1	
V3SVHSHC_5486012	RTFDC1			1.225E-06					RTFDC1	
V3SVHSHC_6218348	LOC101929989			1.244E-06					LOC10192<br>9989	
V3SVHSHC_8999720	KATNAL1			1.300E-06					KATNAL1	
V3SVHSHC_9723674	PIGW			1.385E-06					PIGW	

V3SVHSHC_10817030	RNASE1			1.399E-06					RNASE1	
V3SVHSHC_7549667	TMEM56			1.419E-06					TMEM56	
V3SVHSHC_7492181	LOC101927662			1.420E-06					LOC101927662	
V3SVHSHC_10816601	ERCC8			1.463E-06					ERCC8	
V3SVHSHC_9430139	DENND6A			1.504E-06					DENND6A	
V3SVHSHC_7047275	NPR1			1.547E-06					NPR1	
V3SVHSHC_5645072	C20ORF85			1.586E-06					C20ORF85	
V3SVHSHC_5641805	DEFB107A			1.607E-06					DEFB107A	
V3SVHSHC_5951048	KIAA0100			1.627E-06					KIAA0100	
V3SVHSHC_6887258	C9ORF85			1.636E-06					C9ORF85	
V3SVHSHC_7537556	ST6GALNAC5			1.648E-06					ST6GALNAC5	
V3SVHSHC_7774034	FOXN2			1.667E-06					FOXN2	
V3SVHSHC_5364440	FGF9			1.686E-06					FGF9	
V3SVHSHC_7813370	NAPEPLD			1.767E-06					NAPEPLD	
V3SVHSHC_5391698	NIPAL4			1.792E-06					NIPAL4	
V3SVHSHC_5755622	VPREB3			1.806E-06					VPREB3	
V3SVHSHC_5085326	FAM110D			1.813E-06					FAM110D	
V3SVHSHC_8380838	SCUBE2			1.836E-06					SCUBE2	
V3SVHSHC_9985232	UBE2D3			1.977E-06					UBE2D3	
V3SVHSHC_10165313	FMN2			1.995E-06					FMN2	
V3SVHSHC_8685824	SLC44A1			2.078E-06					SLC44A1	
V3SVHSHC_7523168	PDE1C			2.276E-06					PDE1C	
V3SVHSHC_6388397	TSGA10IP			2.425E-06					TSGA10IP	
V3SVHSHC_9910553	C19orf33			2.545E-06					C19orf33	
V3SVHSHC_8777630	C2orf69			2.588E-06					C2orf69	
V3SVHSHC_6469016	CYP27B1			2.613E-06					CYP27B1	
V3SVHSHC_6697640	SMIM15			2.705E-06					SMIM15	
V3SVHSHC_6519572	VIPR2			2.732E-06					VIPR2	
V3SVHSHC_5785949	ANKS1A			2.789E-06					ANKS1A	
V3SVHSHC_10110071	SMCO2			2.790E-06					SMCO2	
V3SVHSHC_8927549	C5AR2			3.049E-06					C5AR2	
V3SVHSHC_10562204	DLG3			3.074E-06					DLG3	
V3SVHSHC_7258772	RGS5			3.104E-06					RGS5	
V3SVHSHC_10237220	TRIM71			3.289E-06					TRIM71	
V3SVHSHC_9023282	PON1			3.354E-06					PON1	
V3SVHSHC_9736907	ADCY6			3.440E-06					ADCY6	
V3SVHSHC_7494227	SLC12A4			3.497E-06					SLC12A4	
V3SVHSHC_8190626	DNAI1			3.513E-06					DNAI1	
V3SVHSHC_6275801	SLC30A8			3.559E-06					SLC30A8	
V3SVHSHC_10227353	OXT			3.595E-06					OXT	
V3SVHSHC_8048297	C2orf47			3.644E-06					C2orf47	
V3SVHSHC_10362983	SHMT2			3.812E-06					SHMT2	
V3SVHSHC_7799345	CACNB1			3.842E-06	1.051E-13				CACNB1	CACNB1
V3SVHSHC_10162244	SLC25A22			3.929E-06					SLC25A22	
V3SVHSHC_5673155	C17orf78			4.010E-06					C17orf78	
V3SVHSHC_10026185	RBP3			4.025E-06					RBP3	
V3SVHSHC_8598869	HIST1H2BK			4.070E-06					HIST1H2BK	
V3SVHSHC_10096838	IPO7			4.075E-06					IPO7	
V3SVHSHC_6661901	GPR158			4.108E-06					GPR158	
V3SVHSHC_4848716	COX7A2			4.300E-06					COX7A2	
V3SVHSHC_5218910	GRIA4			4.350E-06					GRIA4	
V3SVHSHC_9714401	ACSM3			4.352E-06					ACSM3	

V3SVHSHC_8522177	BLOC1S3			4.360E-06					BLOC1S3	
V3SVHSHC_9415817	CT45A3			4.694E-06					CT45A3	
V3SVHSHC_8218115	CXORF23			4.719E-06					CXORF23	
V3SVHSHC_10078688	RAC1			4.967E-06					RAC1	
V3SVHSHC_7150301	SFT2D1			5.010E-06					SFT2D1	
V3SVHSHC_6962333	NOP10			5.039E-06					NOP10	
V3SVHSHC_8393510	CREB5			5.342E-06					CREB5	
V3SVHSHC_9053180	FAM25A			5.401E-06					FAM25A	
V3SVHSHC_10266425	LOC642441			5.548E-06					LOC64244<br>1	
V3SVHSHC_10713938	PRAME			5.650E-06					PRAME	
V3SVHSHC_5729783	BOK			5.683E-06					BOK	
V3SVHSHC_7854356	SCAF8			5.695E-06					SCAF8	
V3SVHSHC_5012528	TESC			5.724E-06					TESC	
V3SVHSHC_4826210	HUS1			5.734E-06					HUS1	
V3SVHSHC_7867160	GDAP1			5.961E-06					GDAP1	
V3SVHSHC_7718297	PENK			5.962E-06					PENK	
V3SVHSHC_5914583	PRRT3			6.343E-06					PRRT3	
V3SVHSHC_8827790	TNFSF15			6.424E-06					TNFSF15	
V3SVHSHC_10561247	HPSE2			6.503E-06					HPSE2	
V3SVHSHC_5227523	BAK1			6.705E-06					BAK1	
V3SVHSHC_4862939	MYH14			6.796E-06					MYH14	
V3SVHSHC_6101363	AVIL			6.858E-06					AVIL	
V3SVHSHC_8008862	CMAS			7.212E-06					CMAS	
V3SVHSHC_5841917	XRCC2			7.262E-06					XRCC2	
V3SVHSHC_9708890	CTAGE15			7.567E-06					CTAGE15	
V3SVHSHC_4904453	OR6C74			7.869E-06					OR6C74	
V3SVHSHC_6803438	CLDN17			8.036E-06					CLDN17	
V3SVHSHC_10280186	TULP2			8.420E-06					TULP2	
V3SVHSHC_10301405	FSTL3			8.477E-06					FSTL3	
V3SVHSHC_5926100	SLC38A5			8.862E-06					SLC38A5	
V3SVHSHC_4801955	AP5S1			8.961E-06					AP5S1	
V3SVHSHC_5270357	PDZD8			8.962E-06					PDZD8	
V3SVHSHC_10001138	TIMM17A			9.233E-06					TIMM17A	
V3SVHSHC_6546863	NCKAP1L			9.273E-06					NCKAP1L	
V3SVHSHC_5277155	APOB			9.592E-06					APOB	
V3SVHSHC_5043020	SETD8			9.793E-06					SETD8	
V3SVHSHC_9728294	DVL3			1.040E-05					DVL3	
V3SVHSHC_7545575	ING3			1.053E-05					ING3	
V3SVHSHC_6099581	ATP6V0C			1.061E-05					ATP6V0C	
V3SVHSHC_10393772	MYL3			1.075E-05					MYL3	
V3SVHSHC_7024109	LRRIQ1			1.109E-05					LRRIQ1	
V3SVHSHC_8891513	KHDC1L			1.146E-05					KHDC1L	
V3SVHSHC_8756708	CDC25C			1.163E-05					CDC25C	
V3SVHSHC_7387637	Siglec15			1.192E-05	2.442E-06				Siglec15	Siglec15
V3SVHSHC_4771859	ESPN			1.214E-05					ESPN	
V3SVHSHC_5540000	INTU			1.216E-05					INTU	
V3SVHSHC_9723707	SLC13A5			1.256E-05					SLC13A5	
V3SVHSHC_8957678	TRIM32			1.260E-05					TRIM32	
V3SVHSHC_7282070	SEMA4F			1.279E-05					SEMA4F	
V3SVHSHC_9519866	SEZ6L			1.358E-05					SEZ6L	
V3SVHSHC_10579595	HSPBP1			1.420E-05					HSPBP1	
V3SVHSHC_4848452	SLC18A2			1.442E-05					SLC18A2	
V3SVHSHC_5031173	TBC1D21			1.452E-05					TBC1D21	
V3SVHSHC_5432255	FKBP2			1.467E-05					FKBP2	

V3SVHSHC_4857956	C5orf51			1.480E-05					C5orf51	
V3SVHSHC_8230919	ATP4B			1.519E-05					ATP4B	
V3SVHSHC_6508682	FARP2			1.545E-05					FARP2	
V3SVHSHC_4912769	FCGR1B			1.549E-05					FCGR1B	
V3SVHSHC_7978304	FAM83B			1.571E-05					FAM83B	
V3SVHSHC_10204880	CEACAM5			1.619E-05					CEACAM5	
V3SVHSHC_6574682	EMR1			1.646E-05					EMR1	
V3SVHSHC_5224784	DSC1			1.784E-05					DSC1	
V3SVHSHC_7814360	SLC35G1			1.803E-05					SLC35G1	
V3SVHSHC_9760766	C5orf22			1.825E-05					C5orf22	
V3SVHSHC_9941276	GEMIN7			1.840E-05					GEMIN7	
V3SVHSHC_10531250	COL2A1			1.918E-05					COL2A1	
V3SVHSHC_10486931	ZNF385A			1.933E-05					ZNF385A	
V3SVHSHC_10827920	BIRC6			2.034E-05					BIRC6	
V3SVHSHC_10174850	SMR3A			2.054E-05					SMR3A	
V3SVHSHC_6762881	PPP3CA			2.144E-05					PPP3CA	
V3SVHSHC_6855710	IQCB1			2.145E-05					IQCB1	
V3SVHSHC_5427206	Agap1			2.165E-05					Agap1	
V3SVHSHC_5577191	C12orf50			2.246E-05					C12orf50	
V3SVHSHC_7611575	IGFN1			2.309E-05					IGFN1	
V3SVHSHC_5059586	CTSW			2.316E-05					CTSW	
V3SVHSHC_9854123	SCGB3A2			2.382E-05					SCGB3A2	
V3SVHSHC_4984907	SERHL2			2.486E-05					SERHL2	
V3SVHSHC_5135486	WDR81			2.523E-05					WDR81	
V3SVHSHC_8366648	PLB1			2.524E-05					PLB1	
V3SVHSHC_7254218	TMEM167A			2.574E-05					TMEM167 A	
V3SVHSHC_5058662	USP7			2.601E-05					USP7	
V3SVHSHC_5003387	MARCO			2.660E-05					MARCO	
V3SVHSHC_5360645	RFXANK			2.756E-05					RFXANK	
V3SVHSHC_7151423	TMEM229A			2.845E-05					TMEM229 A	
V3SVHSHC_5252933	GJA8			2.872E-05					GJA8	
V3SVHSHC_10691729	OR5M3			2.929E-05					OR5M3	
V3SVHSHC_9233030	FRAT1			2.967E-05					FRAT1	
V3SVHSHC_10552205	ZNF678			3.059E-05					ZNF678	
V3SVHSHC_6999656	SCOC			3.074E-05					SCOC	
V3SVHSHC_6109514	RANBP2			3.104E-05					RANBP2	
V3SVHSHC_9222932	NSUN7			3.130E-05					NSUN7	
V3SVHSHC_9892502	KIAA1328			3.173E-05					KIAA1328	
V3SVHSHC_9218279	HELZ2			3.256E-05					HELZ2	
V3SVHSHC_7051268	EMD			3.468E-05					EMD	
V3SVHSHC_9518348	TRUB1			3.522E-05					TRUB1	
V3SVHSHC_8256857	Sec16a			3.591E-05					Sec16a	
V3SVHSHC_8820365	PTGDR2			3.620E-05					PTGDR2	
V3SVHSHC_10384994	SLC25A28			3.650E-05					SLC25A28	
V3SVHSHC_5648834	CTBS			3.772E-05					CTBS	
V3SVHSHC_7411034	RNF181			3.886E-05					RNF181	
V3SVHSHC_5380280	CNEP1R1			3.976E-05					CNEP1R1	
V3SVHSHC_6675992	SNRNP27			3.995E-05					SNRNP27	
V3SVHSHC_5662232	MS4A4A			4.098E-05					MS4A4A	
V3SVHSHC_9406280	TNNI2			4.150E-05					TNNI2	
V3SVHSHC_5620421	RAB20			4.168E-05					RAB20	
V3SVHSHC_10809836	GTF2E1			4.359E-05					GTF2E1	
V3SVHSHC_5595374	GON4L			4.660E-05					GON4L	

V3SVHSHC_6607286	NUSAP1			4.923E-05					NUSAP1	
V3SVHSHC_6678434	IL5RA			5.194E-05	7.806E-04				IL5RA	
V3SVHSHC_5097668	KIF27			5.315E-05					KIF27	
V3SVHSHC_8600849	NDUFA10			5.503E-05					NDUFA10	
V3SVHSHC_9755288	STRA13			5.568E-05					STRA13	
V3SVHSHC_8587352	TMEM242			5.805E-05					TMEM242	
V3SVHSHC_6033416	DHCR7			5.855E-05					DHCR7	
V3SVHSHC_6003947	KRT10			5.906E-05					KRT10	
V3SVHSHC_9784130	HBEGF			5.963E-05					HBEGF	
V3SVHSHC_8902535	NALCN			6.251E-05					NALCN	
V3SVHSHC_5417537	TINAG			6.267E-05					TINAG	
V3SVHSHC_10692719	GUCD1			6.310E-05					GUCD1	
V3SVHSHC_10597349	MYOM3			6.372E-05	3.790E-16				MYOM3	MYOM3
V3SVHSHC_7878182	SEMA4G			6.735E-05					SEMA4G	
V3SVHSHC_9185939	CCL2			6.793E-05					CCL2	
V3SVHSHC_7613126	OTX2			6.825E-05					OTX2	
V3SVHSHC_8926427	SLC45A2			6.977E-05					SLC45A2	
V3SVHSHC_9505214	GPR149			7.119E-05					GPR149	
V3SVHSHC_8354009	EDEM1			7.134E-05	8.380E-06				EDEM1	EDEM1
V3SVHSHC_9657641	SULT1A2			7.309E-05					SULT1A2	
V3SVHSHC_8813204	CCDC115			7.636E-05					CCDC115	
V3SVHSHC_7124330	C17orf104			7.819E-05					C17orf104	
V3SVHSHC_7202969	RETNLB			7.924E-05					RETNLB	
V3SVHSHC_6537161	MRGPRX4			8.183E-05					MRGPRX4	
V3SVHSHC_6584219	LOC101929766			8.257E-05					LOC10192<br>9766	
V3SVHSHC_9570785	TIMM17A			8.407E-05					TIMM17A	
V3SVHSHC_9038627	ARL2BP			8.515E-05					ARL2BP	
V3SVHSHC_9348134	OR2A1			8.905E-05					OR2A1	
V3SVHSHC_4754831	ADIG			9.101E-05					ADIG	
V3SVHSHC_5234024	WBSCR16			9.139E-05					WBSCR16	
V3SVHSHC_10744562	ZSCAN12			9.535E-05					ZSCAN12	
V3SVHSHC_7636391	LIAS			1.008E-04					LIAS	
V3SVHSHC_7864685	CTLA4			1.008E-04					CTLA4	
V3SVHSHC_9660842	NOTO			1.033E-04					NOTO	
V3SVHSHC_6263921	MTURN			1.063E-04					MTURN	
V3SVHSHC_9865706	SHANK3			1.066E-04					SHANK3	
V3SVHSHC_4994576	KRT84			1.076E-04					KRT84	
V3SVHSHC_5630717	PLIN4			1.081E-04					PLIN4	
V3SVHSHC_5384273	SPG21			1.127E-04					SPG21	
V3SVHSHC_6111956	FAM78B			1.204E-04					FAM78B	
V3SVHSHC_7458554	TMEM161A			1.249E-04					TMEM161<br>A	
V3SVHSHC_10043246	PVR			1.279E-04					PVR	
V3SVHSHC_5421563	RNASE9			1.328E-04					RNASE9	
V3SVHSHC_4895147	UBB			1.422E-04					UBB	
V3SVHSHC_6104993	TRDN			1.482E-04					TRDN	
V3SVHSHC_5764796	TNNC1			1.503E-04					TNNC1	
V3SVHSHC_7707539	LITAF			1.532E-04	2.526E-03				LITAF	
V3SVHSHC_10347770	ZFYVE19			1.718E-04					ZFYVE19	
V3SVHSHC_4866965	RANBP6			1.741E-04					RANBP6	
V3SVHSHC_9955235	PKD1L3			1.781E-04					PKD1L3	
V3SVHSHC_7036880	OR4K5			1.875E-04					OR4K5	
V3SVHSHC_9387602	PFKFB2			1.945E-04					PFKFB2	
V3SVHSHC_10489109	C5orf60			1.954E-04					C5orf60	

V3SVHSHC_7893659	SEMA5A			1.980E-04					SEMA5A	
V3SVHSHC_7069946	WDR87			1.991E-04					WDR87	
V3SVHSHC_10174751	TMEM109			2.036E-04					TMEM109	
V3SVHSHC_8310284	DPP7			2.055E-04					DPP7	
V3SVHSHC_4698401	RCCD1			2.061E-04					RCCD1	
V3SVHSHC_8368001	SH2D4A			2.097E-04					SH2D4A	
V3SVHSHC_6485153	TRPV4			2.152E-04					TRPV4	
V3SVHSHC_4848386	ING2			2.363E-04					ING2	
V3SVHSHC_5708861	KRT27			2.406E-04					KRT27	
V3SVHSHC_10374665	SFTPD			2.416E-04					SFTPD	
V3SVHSHC_5747207	GAGE12J			2.472E-04					GAGE12J	
V3SVHSHC_5150105	SPAST			2.487E-04					SPAST	
V3SVHSHC_6419912	INTS1			2.542E-04					INTS1	
V3SVHSHC_7266263	KIF18B			2.618E-04	6.749E-07				KIF18B	KIF18B
V3SVHSHC_6471920	ATP10D			2.705E-04					ATP10D	
V3SVHSHC_10707173	C17orf80			2.716E-04					C17orf80	
V3SVHSHC_9776111	PATE1			2.817E-04					PATE1	
V3SVHSHC_5330054	CARHSP1			3.019E-04					CARHSP1	
V3SVHSHC_4914221	HNF1B			3.070E-04					HNF1B	
V3SVHSHC_10237583	CHD1			3.077E-04					CHD1	
V3SVHSHC_7786838	BAI1			3.134E-04					BAI1	
V3SVHSHC_7791491	SLC35G6			3.193E-04					SLC35G6	
V3SVHSHC_10345955	LIAS			3.248E-04					LIAS	
V3SVHSHC_4725593	Llph			3.318E-04					Llph	
V3SVHSHC_9320282	SEPT1			3.398E-04					SEPT1	
V3SVHSHC_8824886	GALP			3.431E-04					GALP	
V3SVHSHC_9228245	TLX2			3.576E-04					TLX2	
V3SVHSHC_8133998	PINX1			3.606E-04					PINX1	
V3SVHSHC_8498681	CNR2			3.691E-04					CNR2	
V3SVHSHC_10029749	KLK7			3.699E-04					KLK7	
V3SVHSHC_7682426	HNRNPCP5			3.813E-04					HNRNPCP<br>5	
V3SVHSHC_5096216	HECTD3			3.938E-04					HECTD3	
V3SVHSHC_5018864	OLIG3			4.105E-04					OLIG3	
V3SVHSHC_9039452	TOR3A			4.188E-04					TOR3A	
V3SVHSHC_6530396	PPP3R2			4.190E-04					PPP3R2	
V3SVHSHC_5172578	EBF2			4.285E-04					EBF2	
V3SVHSHC_6404468	SOX10			4.353E-04					SOX10	
V3SVHSHC_5778788	C11orf58			4.576E-04					C11orf58	
V3SVHSHC_7194686	SLCO1B1			4.681E-04					SLCO1B1	
V3SVHSHC_5782187	H1FNT			4.709E-04					H1FNT	
V3SVHSHC_7414499	OCSTAMP			4.714E-04	3.426E-01				OCSTAMP	
V3SVHSHC_8368727	TMPRSS4			5.086E-04					TMPRSS4	
V3SVHSHC_10079975	PROM2			5.230E-04					PROM2	
V3SVHSHC_9751130	SCARA3			5.498E-04					SCARA3	
V3SVHSHC_4716650	FPR2			5.511E-04					FPR2	
V3SVHSHC_7662395	CNR2			5.556E-04					CNR2	
V3SVHSHC_10846928	MARCKSL1			6.249E-04					MARCKSL<br>1	
V3SVHSHC_8468849	MYF6			6.295E-04					MYF6	
V3SVHSHC_8299724	ZBTB32			6.312E-04					ZBTB32	
V3SVHSHC_8828780	USP40			6.623E-04					USP40	
V3SVHSHC_7685693	P4HTM			6.739E-04					P4HTM	
V3SVHSHC_6111527	OTUB1			6.759E-04					OTUB1	

V3SVHSHC_4724735	TMPRSS2			7.009E-04					TMPRSS2	
V3SVHSHC_7898345	C4orf26			7.299E-04					C4orf26	
V3SVHSHC_6317150	PLXNA2			7.325E-04					PLXNA2	
V3SVHSHC_9905834	ICAM3			7.347E-04					ICAM3	
V3SVHSHC_6905507	NCKAP5			7.386E-04					NCKAP5	
V3SVHSHC_10686614	PPP1R35			7.616E-04	2.258E-22				PPP1R35	PPP1R35
V3SVHSHC_7649063	VTI1B			7.759E-04					VTI1B	
V3SVHSHC_9544616	ZFP14			7.801E-04					ZFP14	
V3SVHSHC_9243326	RYBP			7.803E-04					RYBP	
V3SVHSHC_4778657	Msmp			7.910E-04					Msmp	
V3SVHSHC_7435256	CHRM4			8.124E-04					CHRM4	
V3SVHSHC_8994440	MAPK6			8.784E-04						
V3SVHSHC_9499736	CLPTM1L			8.910E-04						
V3SVHSHC_6331406	ANGPT2			9.061E-04						
V3SVHSHC_6715856	SLC25A10			9.433E-04						
V3SVHSHC_7891085	NR2F6			9.655E-04						
V3SVHSHC_7653683	CEP250			9.667E-04						
V3SVHSHC_8223758	ALDH16A1			9.692E-04						
V3SVHSHC_9565901	TMEM144			1.009E-03	3.091E-06					TMEM144
V3SVHSHC_7231646	C8orf59			1.018E-03						
V3SVHSHC_5947847	ACBD6			1.020E-03						
V3SVHSHC_6724667	ZNF81			1.038E-03						
V3SVHSHC_6200825	Stap1			1.067E-03						
V3SVHSHC_6547688	HSD17B10			1.122E-03						
V3SVHSHC_6273194	SYT16			1.136E-03						
V3SVHSHC_10650809	STIP1			1.150E-03						
V3SVHSHC_6680117	ADARB2			1.151E-03						
V3SVHSHC_6537986	TRAPPC6B			1.152E-03						
V3SVHSHC_10306454	UBB			1.212E-03						
V3SVHSHC_6841982	SEC31B			1.220E-03						
V3SVHSHC_9448421	MAD2L1			1.271E-03						
V3SVHSHC_10535177	ATG13			1.362E-03						
V3SVHSHC_7510463	CA5B			1.377E-03						
V3SVHSHC_5276825	ZSCAN9			1.379E-03	4.186E-01					
V3SVHSHC_8040707	C11orf82			1.410E-03						
V3SVHSHC_9378857	EPHX3			1.436E-03						
V3SVHSHC_8104661	LIPE			1.440E-03						
V3SVHSHC_5177990	AKR1B15			1.488E-03						
V3SVHSHC_7919828	RNF183			1.579E-03						
V3SVHSHC_7566926	AMPD2			1.641E-03						
V3SVHSHC_6500828	UTS2			1.656E-03						
V3SVHSHC_5452715	ALOX12			1.769E-03						
V3SVHSHC_5541947	LOC101060275			1.778E-03	2.271E-09					LOC10106<br>0275
V3SVHSHC_5892308	GLIS3			1.813E-03						
V3SVHSHC_7306193	CTAGE1			1.914E-03						
V3SVHSHC_7236959	C16orf97			2.043E-03						
V3SVHSHC_7812215	Carkd			2.052E-03						
V3SVHSHC_8492873	COCH			2.120E-03	4.476E-03					
V3SVHSHC_10396973	SYVN1			2.146E-03	3.218E-01					
V3SVHSHC_6817397	GLCCI1			2.344E-03						
V3SVHSHC_6525875	ARL2			2.344E-03						
V3SVHSHC_8767466	FAM177A1			2.387E-03						
V3SVHSHC_5348534	LOC101929564			2.392E-03	4.059E-01					
V3SVHSHC_8124461	SLITRK3			2.424E-03						

V3SVHSHC_7942037	FBXL8			2.530E-03						
V3SVHSHC_7636226	GLDC			2.546E-03						
V3SVHSHC_8490464	SBSN			2.804E-03						
V3SVHSHC_6947582	ACSF3			2.924E-03						
V3SVHSHC_5263823	HP			2.945E-03						
V3SVHSHC_8231348	PLAT			2.967E-03						
V3SVHSHC_4710875	MMP27			3.247E-03						
V3SVHSHC_6861947	lqcf5			3.424E-03						
V3SVHSHC_6580193	SYF2			3.528E-03						
V3SVHSHC_10518710	IKZF4			3.571E-03						
V3SVHSHC_9092912	PLEKHG6			3.589E-03						
V3SVHSHC_4884851	OR7A17			3.661E-03						
V3SVHSHC_8397206	MGST3			3.717E-03						
V3SVHSHC_6517757	PTPN7			3.733E-03						
V3SVHSHC_8302034	C19ORF12			3.789E-03						
V3SVHSHC_5540033	CCDC114			3.944E-03						
V3SVHSHC_8169044	SPINK1			3.986E-03						
V3SVHSHC_9292232	LNPEP			4.187E-03						
V3SVHSHC_10458650	SHB			4.228E-03						
V3SVHSHC_4862345	DPY19L2			4.467E-03						
V3SVHSHC_4770935	FOXQ1			4.543E-03						
V3SVHSHC_8293058	ALKBH1			4.671E-03						
V3SVHSHC_5169509	ZFC3H1			5.439E-03						
V3SVHSHC_5215973	EDC4			5.704E-03						
V3SVHSHC_10362323	OR52R1			6.155E-03						
V3SVHSHC_5293952	OR6K2			6.239E-03						
V3SVHSHC_8434331	EHMT2			6.318E-03						
V3SVHSHC_4722986	LOC101060445			6.405E-03						
V3SVHSHC_8737337	VPS37A			6.424E-03						
V3SVHSHC_9754529	REPS2			6.490E-03						
V3SVHSHC_6863663	CT83			6.834E-03						
V3SVHSHC_8128784	PDRG1			7.107E-03						
V3SVHSHC_8808584	KIAA0195			7.108E-03						
V3SVHSHC_10820858	IAH1			7.190E-03						
V3SVHSHC_9008960	GTF2H1			7.253E-03						
V3SVHSHC_4757174	ASUN			7.367E-03						
V3SVHSHC_4807895	HAS2			7.840E-03						
V3SVHSHC_8756180	PMF1			8.121E-03						
V3SVHSHC_7440998	STEAP2			8.604E-03						
V3SVHSHC_9400637	OR5H14			8.853E-03						
V3SVHSHC_4681142	ARL3			8.884E-03						
V3SVHSHC_6041501	ALOX15			8.962E-03						
V3SVHSHC_5785355	C11ORF1			9.066E-03	3.476E-01					
V3SVHSHC_5415524	GTSE1			9.070E-03						
V3SVHSHC_5933723	CCL4L2			9.079E-03						
V3SVHSHC_6241019	KDELR3			9.825E-03						
V3SVHSHC_5854556	WBSCR28			1.001E-02						
V3SVHSHC_10211612	HSPA4L			1.034E-02						
V3SVHSHC_6450965	ZRANB2			1.088E-02						
V3SVHSHC_7986719	GNG8			1.099E-02						
V3SVHSHC_5915276	SLC29A1			1.126E-02						
V3SVHSHC_5870429	NIT1			1.139E-02						
V3SVHSHC_10848413	PATL2			1.145E-02						
V3SVHSHC_10510988	KCNA7			1.166E-02						
V3SVHSHC_7266758	SLC45A3			1.175E-02						

V3SVHSHC_8286095	CD164			1.205E-02						
V3SVHSHC_5130107	CES4A			1.245E-02						
V3SVHSHC_4683584	POLR2A			1.248E-02						
V3SVHSHC_5455124	FGF10			1.286E-02						
V3SVHSHC_9959030	UBQLN3			1.289E-02						
V3SVHSHC_5841950	DUSP12			1.336E-02						
V3SVHSHC_5773013	PARP12			1.400E-02						
V3SVHSHC_5164427	NNAT			1.424E-02						
V3SVHSHC_8444726	TOM1L2			1.477E-02						
V3SVHSHC_5696915	TAAR9			1.491E-02	4.226E-07					TAAR9
V3SVHSHC_9234845	PPM1E			1.507E-02	4.741E-01					
V3SVHSHC_8816735	OR5AC2			1.512E-02						
V3SVHSHC_5035826	PTGER3			1.513E-02						
V3SVHSHC_10483499	ZNF592			1.538E-02						
V3SVHSHC_5768657	WBSCR28			1.564E-02						
V3SVHSHC_9678695	CYP4F3			1.627E-02						
V3SVHSHC_10089446	LFNG			1.663E-02						
V3SVHSHC_10387172	DTX4			1.703E-02						
V3SVHSHC_7348994	PPIB			1.733E-02						
V3SVHSHC_5031305	ZNF98			1.748E-02						
V3SVHSHC_9303980	BAG6			1.775E-02						
V3SVHSHC_8103440	PDE2A			1.853E-02						
V3SVHSHC_5146310	IFLTD1			1.934E-02						
V3SVHSHC_5080574	CLDN3			1.956E-02						
V3SVHSHC_6174722	CCDC176			1.970E-02						
V3SVHSHC_6600290	RNF208			2.060E-02						
V3SVHSHC_5393282	BTN3A2			2.072E-02						
V3SVHSHC_10548773	C20orf196			2.072E-02						
V3SVHSHC_9118982	ATF7IP2			2.113E-02						
V3SVHSHC_9640712	GJC3			2.130E-02						
V3SVHSHC_4930094	MZT1			2.155E-02						
V3SVHSHC_8654969	TMEM261			2.192E-02						
V3SVHSHC_9002327	C8orf76			2.245E-02						
V3SVHSHC_6680645	OR4D1			2.253E-02						
V3SVHSHC_7008764	LOC100133128			2.254E-02						
V3SVHSHC_8782217	PRPF40B			2.337E-02						
V3SVHSHC_7785848	ABCC1			2.398E-02						
V3SVHSHC_9647510	UGT3A2			2.481E-02						
V3SVHSHC_7866236	GKAP1			2.579E-02						
V3SVHSHC_5372360	RNF6			2.589E-02						
V3SVHSHC_5897621	TUBB			2.691E-02						
V3SVHSHC_10764131	ZNF560			2.814E-02						
V3SVHSHC_9278867	GOLGA6D			2.839E-02						
V3SVHSHC_6681569	TMCC3			2.875E-02						
V3SVHSHC_5667479	PITPNM3			2.911E-02						
V3SVHSHC_7710542	CFHR2			2.954E-02						
V3SVHSHC_9593159	FGD5			2.967E-02						
V3SVHSHC_5232308	HOXC6			3.000E-02						
V3SVHSHC_7190231	CAPZA2			3.027E-02						
V3SVHSHC_10260881	AGO3			3.067E-02						
V3SVHSHC_10837688	SLC16A14			3.096E-02						
V3SVHSHC_6303950	CD320			3.144E-02						
V3SVHSHC_5190596	ALCAM			3.158E-02						
V3SVHSHC_7757699	TMTC2			3.268E-02						
V3SVHSHC_7791623	ATF7IP			3.268E-02						

V3SVHSHC_9775088	SERTAD2			3.349E-02						
V3SVHSHC_6248741	SLC39A4			3.391E-02						
V3SVHSHC_8722883	HMGN1			3.544E-02						
V3SVHSHC_5256365	PIK3AP1			3.548E-02						
V3SVHSHC_8034140	ALDH7A1			3.549E-02						
V3SVHSHC_5686190	ZNF677			3.566E-02						
V3SVHSHC_10631042	FMO1			3.591E-02						
V3SVHSHC_8947085	SOCS1			3.634E-02						
V3SVHSHC_9426212	CALML5			3.646E-02	7.369E-21					CALML5
V3SVHSHC_4976162	PAK3			3.902E-02						
V3SVHSHC_10145348	HOXB8			3.946E-02						
V3SVHSHC_4877228	LEO1			4.057E-02						
V3SVHSHC_5668535	GABARAPL1			4.221E-02						
V3SVHSHC_7174193	TTC5			4.286E-02						
V3SVHSHC_7667378	LOC101929183			4.484E-02						
V3SVHSHC_10808219	SMN2			4.534E-02	3.725E-16					SMN2
V3SVHSHC_7690643	BRD3			4.557E-02						
V3SVHSHC_9392882	Col6a5			4.593E-02						
V3SVHSHC_10588835	KRBOX4			4.654E-02						
V3SVHSHC_10275731	HPR			5.154E-02						
V3SVHSHC_10319291	LOC285095			5.351E-02						
V3SVHSHC_8240522	PARD6G			5.385E-02						
V3SVHSHC_6502346	FAM160A1			5.559E-02						
V3SVHSHC_9961604	CHCHD6			5.614E-02						
V3SVHSHC_7691072	ALX3			5.807E-02						
V3SVHSHC_8171090	CENPE			6.412E-02						
V3SVHSHC_9973550	RAD51			6.428E-02						
V3SVHSHC_10084727	TRAF3			6.465E-02						
V3SVHSHC_7591016	LIPI			6.668E-02						
V3SVHSHC_5063942	FGFR1			6.726E-02						
V3SVHSHC_8784197	SLC5A6			6.796E-02						
V3SVHSHC_10001237	TM2D3			7.097E-02						
V3SVHSHC_9144161	YIPF5			7.255E-02						
V3SVHSHC_8495711	GIMAP1			7.383E-02						
V3SVHSHC_8033348	PNLDC1			7.417E-02						
V3SVHSHC_7136606	EDDM3A			7.926E-02						
V3SVHSHC_4997777	POLR1A			8.046E-02						
V3SVHSHC_7914119	FAM185A			8.245E-02						
V3SVHSHC_9908573	GAGE2D			8.267E-02						
V3SVHSHC_7951343	ZCCHC7			8.330E-02						
V3SVHSHC_8693084	NARG2			8.337E-02						
V3SVHSHC_8469839	Ak4			8.379E-02						
V3SVHSHC_8432318	JSRP1			8.465E-02						
V3SVHSHC_10083935	DYNC1H1			8.794E-02						
V3SVHSHC_9099248	GSTO2			8.896E-02						
V3SVHSHC_8830661	FBXW5			8.922E-02	9.913E-22					FBXW5
V3SVHSHC_6157562	DHCR7			9.175E-02						
V3SVHSHC_9858941	ERVV-1			9.257E-02						
V3SVHSHC_10227452	ACTG2			9.578E-02						
V3SVHSHC_8379518	LAMB1			9.723E-02						
V3SVHSHC_8715656	PABPC3			9.971E-02						
V3SVHSHC_7219172	METTL23			1.015E-01						
V3SVHSHC_9802841	KLF7			1.023E-01						
V3SVHSHC_5346686	CACNB3			1.035E-01						
V3SVHSHC_6345959	LDB2			1.036E-01						

V3SVHSHC_7827560	CENPE			1.043E-01						
V3SVHSHC_5675861	UBB			1.078E-01	1.842E-02					
V3SVHSHC_6068462	CENPE			1.097E-01						
V3SVHSHC_9812675	OR4F16			1.137E-01						
V3SVHSHC_7766609	FAM214A			1.142E-01						
V3SVHSHC_4639133	IL21R			1.151E-01						
V3SVHSHC_8757467	VEZT			1.167E-01						
V3SVHSHC_6574649	GAPDH			1.204E-01						
V3SVHSHC_10312361	Zdbf2			1.215E-01						
V3SVHSHC_10419677	GIMAP7			1.215E-01						
V3SVHSHC_4705727	SHPK			1.260E-01						
V3SVHSHC_7542506	C16orf46			1.266E-01						
V3SVHSHC_5367047	RC3H2			1.266E-01						
V3SVHSHC_7486439	FGFR1			1.305E-01						
V3SVHSHC_5693483	SLC35G6			1.324E-01						
V3SVHSHC_5933162	RAE1			1.340E-01						
V3SVHSHC_5376650	PSMD6			1.343E-01						
V3SVHSHC_7099382	ACTB			1.366E-01						
V3SVHSHC_6802580	RNF219			1.392E-01						
V3SVHSHC_5502149	HNRNPA1L2			1.395E-01						
V3SVHSHC_5380577	HHIPL2			1.402E-01						
V3SVHSHC_9967181	URB1			1.413E-01	2.419E-01					
V3SVHSHC_6508913	CENPE			1.476E-01						
V3SVHSHC_6694175	LAMB1			1.511E-01						
V3SVHSHC_9938174	PDE8B			1.511E-01						
V3SVHSHC_9899234	NDUFB8			1.521E-01						
V3SVHSHC_7264712	H3F3B			1.537E-01						
V3SVHSHC_6833963	C7orf71			1.554E-01						
V3SVHSHC_5566763	GDPD1			1.583E-01						
V3SVHSHC_8309987	YAP1			1.696E-01						
V3SVHSHC_4757867	GALM			1.723E-01						
V3SVHSHC_8243228	TTC8			1.781E-01						
V3SVHSHC_9928901	POLR2B			1.796E-01						
V3SVHSHC_7045295	OR2D2			1.821E-01						
V3SVHSHC_7291541	FANCD2			1.824E-01						
V3SVHSHC_5721533	C9orf153			1.832E-01						
V3SVHSHC_5937452	CDHR1			1.837E-01						
V3SVHSHC_9837524	LOC101930233			1.844E-01						
V3SVHSHC_5480039	ZNF630			1.855E-01						
V3SVHSHC_8563658	INO80D			1.924E-01						
V3SVHSHC_4855712	PUF60			1.927E-01						
V3SVHSHC_7135913	Eif6			1.928E-01						
V3SVHSHC_7438490	MAT2A			1.942E-01						
V3SVHSHC_9192341	PLK1			1.943E-01						
V3SVHSHC_9513695	DNAJA3			1.979E-01						
V3SVHSHC_5116940	PHOSPHO1			2.019E-01						
V3SVHSHC_9606920	MRGPRX4			2.051E-01						
V3SVHSHC_7845083	DSCR3			2.146E-01						
V3SVHSHC_7315433	C9ORF116			2.183E-01						
V3SVHSHC_6260423	MUC3B			2.185E-01						
V3SVHSHC_5582867	ARID1B			2.189E-01						
V3SVHSHC_9395621	LOR			2.265E-01						
V3SVHSHC_5761694	MAOB			2.273E-01						
V3SVHSHC_6219800	EPT1			2.284E-01						
V3SVHSHC_9932366	VSTM5			2.352E-01						

V3SVHSHC_5886764	POLR2B			2.361E-01						
V3SVHSHC_8800103	CENPE			2.414E-01						
V3SVHSHC_7523927	NRD1			2.481E-01						
V3SVHSHC_9823334	COPB2			2.487E-01						
V3SVHSHC_10091228	LOC100996646			2.524E-01						
V3SVHSHC_8012228	KLRC2			2.576E-01						
V3SVHSHC_7963025	NIP7			2.679E-01						
V3SVHSHC_10340345	EIF2S2			2.689E-01						
V3SVHSHC_6350777	EXT1			2.697E-01						
V3SVHSHC_4877723	LOC285500			2.830E-01						
V3SVHSHC_5824394	NAA30			2.858E-01						
V3SVHSHC_6250391	PRMT2			2.862E-01						
V3SVHSHC_9038198	IMP4			2.864E-01						
V3SVHSHC_8269529	GDPD3			2.890E-01						
V3SVHSHC_8129279	PRSS50			2.911E-01						
V3SVHSHC_10141421	PPP1R14A			2.959E-01						
V3SVHSHC_7115849	YEATS4			3.059E-01						
V3SVHSHC_8268209	PLAGL1			3.092E-01						
V3SVHSHC_6393809	N4BP1			3.111E-01						
V3SVHSHC_5342924	PCDH11X			3.154E-01						
V3SVHSHC_9756476	COL14A1			3.276E-01	2.682E-13					COL14A1
V3SVHSHC_8507030	INTS1			3.303E-01						
V3SVHSHC_5032592	ZNF569			3.398E-01						
V3SVHSHC_7795748	RFESD			3.399E-01						
V3SVHSHC_7468619	SLC10A6			3.415E-01						
V3SVHSHC_6096050	SNRPF			3.422E-01						
V3SVHSHC_9400934	FCHSD2			3.473E-01						
V3SVHSHC_9505808	H3F3B			3.515E-01						
V3SVHSHC_7106015	PPFIA4			3.548E-01						
V3SVHSHC_7962794	NIP7			3.570E-01						
V3SVHSHC_6221582	YAP1			3.571E-01						
V3SVHSHC_10406939	NIP7			3.653E-01						
V3SVHSHC_5148488	SNRPF			3.682E-01						
V3SVHSHC_10786637	GPBP1			3.687E-01						
V3SVHSHC_5169344	HES1			3.776E-01						
V3SVHSHC_5915573	DHTKD1			3.832E-01						
V3SVHSHC_9477197	MMS19			3.859E-01	5.994E-08					MMS19
V3SVHSHC_10006946	PPAN			3.909E-01						
V3SVHSHC_9959789	SNRPF			4.001E-01						
V3SVHSHC_6170168	OXSM			4.052E-01						
V3SVHSHC_9651206	PPAN			4.161E-01						
V3SVHSHC_9017606	PPAN			4.294E-01						
V3SVHSHC_8632925	TCEAL2			4.372E-01						
V3SVHSHC_8346980	NANOS2			4.387E-01						
V3SVHSHC_8861318	POLR1A			4.481E-01	2.350E-01					
V3SVHSHC_4877822	FLOT2			4.485E-01						
V3SVHSHC_7730408	FAM73B			4.503E-01						
V3SVHSHC_7275404	OR2L5			4.655E-01						
V3SVHSHC_7376582	HNRNPF			4.667E-01						
V3SVHSHC_8076611	NT5DC3			4.668E-01						
V3SVHSHC_8916692	AKAP9			4.762E-01						
V3SVHSHC_7655894	LOC100129924			4.835E-01						
V3SVHSHC_8663120	MFN1			4.837E-01						
V3SVHSHC_9560885	APOA1BP				6.502E-80					APOA1BP
V3SVHSHC_8232635	PLEKHS1				9.624E-50					PLEKHS1

V3SVHSHC_9565274	APOC2				5.846E-46					APOC2
V3SVHSHC_7262864	LYPD1				7.749E-46					LYPD1
V3SVHSHC_9597944	AGAP5				8.344E-45					AGAP5
V3SVHSHC_10717238	CDH17				3.832E-43					CDH17
V3SVHSHC_9891512	Ms4a15				1.906E-39					Ms4a15
V3SVHSHC_7003055	LOC101930154				9.225E-39					LOC101930154
V3SVHSHC_6551813	WBSCR27				6.763E-38					WBSCR27
V3SVHSHC_4789877	TRIM16L				1.716E-37					TRIM16L
V3SVHSHC_4659296	LOC101060022				5.580E-37					LOC101060022
V3SVHSHC_10015526	CBX6				2.066E-36					CBX6
V3SVHSHC_7382423	SPATA31A7				1.898E-35					SPATA31A7
V3SVHSHC_8604281	UHMK1				2.632E-35					UHMK1
V3SVHSHC_5686586	CALML6				3.027E-34					CALML6
V3SVHSHC_7751462	RTCB				6.138E-33					RTCB
V3SVHSHC_9223724	AKR1C3				1.880E-31					AKR1C3
V3SVHSHC_9297941	GP9				1.362E-30					GP9
V3SVHSHC_7239236	NBPF6				2.830E-30					NBPF6
V3SVHSHC_6965996	RSPO2				3.983E-30					RSPO2
V3SVHSHC_4965173	EGLN3				8.702E-30					EGLN3
V3SVHSHC_6881384	PRRC1				1.527E-29					PRRC1
V3SVHSHC_8047670	ATF7IP2				1.850E-29					ATF7IP2
V3SVHSHC_5776181	ARNT2				2.792E-29					ARNT2
V3SVHSHC_9047966	ZNF582				3.752E-29					ZNF582
V3SVHSHC_8859503	U2AF1L4				6.382E-29					U2AF1L4
V3SVHSHC_8577815	C19orf35				6.617E-29					C19orf35
V3SVHSHC_4935605	OAZ2				8.139E-29					OAZ2
V3SVHSHC_6761858	HEPHL1				1.228E-28					HEPHL1
V3SVHSHC_9793865	SH2D1A				1.232E-28					SH2D1A
V3SVHSHC_9233426	PTDSS1				2.107E-28					PTDSS1
V3SVHSHC_8774330	TNPO2				2.571E-28					TNPO2
V3SVHSHC_8583491	RPA4				3.502E-28					RPA4
V3SVHSHC_10359122	RNASEK				4.600E-28					RNASEK
V3SVHSHC_7287548	CD79B				6.275E-28					CD79B
V3SVHSHC_9351665	AMIGO2				7.829E-28					AMIGO2
V3SVHSHC_6049289	TREX1				1.789E-27					TREX1
V3SVHSHC_7133537	OR6C6				2.384E-27					OR6C6
V3SVHSHC_5786411	ALDH1A3				3.468E-27					ALDH1A3
V3SVHSHC_8700707	FAM101A				4.975E-27					FAM101A
V3SVHSHC_6665861	FAM43B				5.725E-27					FAM43B
V3SVHSHC_7779875	SLC22A6				8.637E-27					SLC22A6
V3SVHSHC_4938674	QTRT1				9.405E-27					QTRT1
V3SVHSHC_10084463	AHDC1				1.006E-26					AHDC1
V3SVHSHC_7511090	RAB29				1.081E-26					RAB29
V3SVHSHC_9204617	Akip1				1.283E-26					Akip1
V3SVHSHC_8764562	NOG				1.352E-26					NOG
V3SVHSHC_10147922	HGS				1.353E-26					HGS
V3SVHSHC_9619559	KIAA0226L				1.415E-26					KIAA0226L
V3SVHSHC_8047571	MGMT				1.893E-26					MGMT
V3SVHSHC_9601772	HOXB4				3.394E-26					HOXB4
V3SVHSHC_7359422	AP3M2				3.572E-26					AP3M2
V3SVHSHC_10789508	USP9Y				4.062E-26					USP9Y
V3SVHSHC_8598737	CAPS				4.555E-26					CAPS

V3SVHSHC_7683218	CCL3L1				4.717E-26					CCL3L1
V3SVHSHC_5894552	DUSP3				4.797E-26					DUSP3
V3SVHSHC_6413378	DEFB131				5.016E-26					DEFB131
V3SVHSHC_8350907	SPINK5				5.113E-26					SPINK5
V3SVHSHC_8749679	LOC101927594				6.122E-26					LOC101927594
V3SVHSHC_9112151	SF3A2				6.285E-26					SF3A2
V3SVHSHC_8950418	RNASE6				6.782E-26					RNASE6
V3SVHSHC_9772580	SYNJ2BP-COX16				7.153E-26					SYNJ2BP-COX16
V3SVHSHC_8272697	WNT5A				7.461E-26					WNT5A
V3SVHSHC_8092781	ANKRD60				9.452E-26					ANKRD60
V3SVHSHC_6919136	INHA				1.180E-25					INHA
V3SVHSHC_9811058	BNC2				1.764E-25					BNC2
V3SVHSHC_4711997	PATL1				2.986E-25					PATL1
V3SVHSHC_9644936	ADHFE1				3.349E-25					ADHFE1
V3SVHSHC_7827164	RGL2				6.319E-25					RGL2
V3SVHSHC_5346653	DFNA5				7.468E-25					DFNA5
V3SVHSHC_10638038	PTBP1				7.565E-25					PTBP1
V3SVHSHC_6704603	SPIN2B				8.197E-25					SPIN2B
V3SVHSHC_7948637	GOLGA1				1.155E-24					GOLGA1
V3SVHSHC_10591310	RNF213				1.653E-24					RNF213
V3SVHSHC_7003880	CLDN15				2.185E-24					CLDN15
V3SVHSHC_6471788	EEA1				2.913E-24					EEA1
V3SVHSHC_5770538	FAM19A5				3.290E-24					FAM19A5
V3SVHSHC_9448322	CARD14				3.698E-24					CARD14
V3SVHSHC_5500862	A2ML1				4.016E-24					A2ML1
V3SVHSHC_8202176	KCNK2				4.135E-24					KCNK2
V3SVHSHC_5369819	ESPNL				4.700E-24					ESPNL
V3SVHSHC_7952168	STK32A				4.990E-24					STK32A
V3SVHSHC_7217522	ELL2				5.540E-24					ELL2
V3SVHSHC_10542338	C1QTNF6				5.809E-24					C1QTNF6
V3SVHSHC_10673348	CYBA				7.990E-24					CYBA
V3SVHSHC_6120239	PRADC1				9.713E-24					PRADC1
V3SVHSHC_5308637	ADAMTS10				1.100E-23					ADAMTS10
V3SVHSHC_10413044	OLFML1				1.155E-23					OLFML1
V3SVHSHC_6059783	B4GALT5				1.209E-23					B4GALT5
V3SVHSHC_9465944	MRPS18C				1.336E-23					MRPS18C
V3SVHSHC_9908837	VPS26A				1.802E-23					VPS26A
V3SVHSHC_8480003	TMEM183B				1.842E-23					TMEM183B
V3SVHSHC_5389916	ZBTB14				2.169E-23					ZBTB14
V3SVHSHC_8875046	GNPNAT1				2.275E-23					GNPNAT1
V3SVHSHC_7523432	KIAA0319L				2.275E-23					KIAA0319L
V3SVHSHC_8439413	TTC7B				2.277E-23					TTC7B
V3SVHSHC_6833138	HYAL4				2.390E-23					HYAL4
V3SVHSHC_7956887	GSTO1				3.170E-23					GSTO1
V3SVHSHC_9093011	HOXB2				3.402E-23					HOXB2
V3SVHSHC_6635897	CARD9				4.799E-23					CARD9
V3SVHSHC_5980220	C19ORF18				4.993E-23					C19ORF18
V3SVHSHC_9019883	PAPL				5.193E-23					PAPL
V3SVHSHC_7102616	NUDT7				5.341E-23					NUDT7
V3SVHSHC_8718329	IGJ				5.356E-23					IGJ

V3SVHSHC_5827199	ABHD12				5.361E-23					ABHD12
V3SVHSHC_9051530	Hspa13				5.614E-23					Hspa13
V3SVHSHC_7636919	ROCK2				6.902E-23					ROCK2
V3SVHSHC_10232666	AVPR2				9.243E-23					AVPR2
V3SVHSHC_4832150	GLT1D1				1.201E-22					GLT1D1
V3SVHSHC_7112681	MYO3A				1.227E-22					MYO3A
V3SVHSHC_8481059	KLF2				1.432E-22					KLF2
V3SVHSHC_9039287	PTGES3				2.578E-22					PTGES3
V3SVHSHC_9994505	CACNB2				3.384E-22					CACNB2
V3SVHSHC_5490467	SNX4				4.118E-22					SNX4
V3SVHSHC_8265173	VWA5B2				4.722E-22					VWA5B2
V3SVHSHC_9638237	C16orf96				5.215E-22					C16orf96
V3SVHSHC_5243231	LOXL1				5.351E-22					LOXL1
V3SVHSHC_9299426	ALG6				6.601E-22					ALG6
V3SVHSHC_7421000	CA13				6.998E-22					CA13
V3SVHSHC_5039885	MFAP3				7.169E-22					MFAP3
V3SVHSHC_9889268	CLCN6				9.451E-22					CLCN6
V3SVHSHC_10747862	KDELC2				1.092E-21					KDELC2
V3SVHSHC_6728825	KLHDC2				1.359E-21					KLHDC2
V3SVHSHC_5330846	CYP1A1				1.470E-21					CYP1A1
V3SVHSHC_10423637	PTPLAD1				1.527E-21					PTPLAD1
V3SVHSHC_7763177	MRPL27				1.845E-21					MRPL27
V3SVHSHC_10174058	SLC39A13				1.928E-21					SLC39A13
V3SVHSHC_6498650	RET				2.011E-21					RET
V3SVHSHC_6441560	MS4A13				2.267E-21					MS4A13
V3SVHSHC_8985728	PHKA1				2.444E-21					PHKA1
V3SVHSHC_10034666	ATP5O				2.666E-21					ATP5O
V3SVHSHC_10482509	C1GALT1				2.714E-21					C1GALT1
V3SVHSHC_8999225	ITGB7				2.759E-21					ITGB7
V3SVHSHC_8280617	MRPS36				2.797E-21					MRPS36
V3SVHSHC_5046254	MVP				3.151E-21					MVP
V3SVHSHC_8822576	DHCR24				3.539E-21					DHCR24
V3SVHSHC_9292331	STK33				4.026E-21					STK33
V3SVHSHC_6926264	CPO				4.795E-21					CPO
V3SVHSHC_5415722	CARNS1				5.120E-21					CARNS1
V3SVHSHC_6731960	OR4L1				5.317E-21					OR4L1
V3SVHSHC_6875906	FAM49B				5.570E-21					FAM49B
V3SVHSHC_7652627	PCDHA3				6.505E-21					PCDHA3
V3SVHSHC_5494856	BEX1				6.572E-21					BEX1
V3SVHSHC_7928837	LOC100996415				7.004E-21					LOC10099 6415
V3SVHSHC_9275534	OLFM4				7.024E-21					OLFM4
V3SVHSHC_10508777	TBX19				7.416E-21					TBX19
V3SVHSHC_7064402	NCS1				1.189E-20					NCS1
V3SVHSHC_8160431	TMEM41B				1.223E-20					TMEM41B
V3SVHSHC_8927846	IFNA2				1.266E-20					IFNA2
V3SVHSHC_8345891	B3GALT1				1.392E-20					B3GALT1
V3SVHSHC_8072255	PGK2				1.619E-20					PGK2
V3SVHSHC_5994476	CHL1				1.671E-20					CHL1
V3SVHSHC_10171814	S100Z				1.887E-20					S100Z
V3SVHSHC_10623452	CHD6				1.891E-20					CHD6
V3SVHSHC_5156408	BAMBI				1.926E-20					BAMBI
V3SVHSHC_6961871	FYN				2.016E-20					FYN
V3SVHSHC_5514854	UNK				2.087E-20					UNK
V3SVHSHC_9515477	HYI				2.092E-20					HYI

V3SVHSHC_5248841	RASAL3				2.134E-20					RASAL3
V3SVHSHC_9540029	CALCRL				2.263E-20					CALCRL
V3SVHSHC_6294182	LOC100129361				2.319E-20					LOC100129361
V3SVHSHC_5764928	MYEOV				2.392E-20					MYEOV
V3SVHSHC_7435388	HOMER1				2.654E-20					HOMER1
V3SVHSHC_6394172	LOC100996350				2.739E-20					LOC100996350
V3SVHSHC_6131624	ZNF521				2.921E-20					ZNF521
V3SVHSHC_7944809	CSMD1				3.441E-20					CSMD1
V3SVHSHC_7812908	AKT1S1				3.610E-20					AKT1S1
V3SVHSHC_7359620	TSTD3				3.674E-20					TSTD3
V3SVHSHC_10436705	APOA5				3.844E-20					APOA5
V3SVHSHC_9170561	SLC22A9				4.755E-20					SLC22A9
V3SVHSHC_5648471	MARC2				4.791E-20					MARC2
V3SVHSHC_8009456	P4HA1				4.944E-20					P4HA1
V3SVHSHC_5916398	MTA2				4.971E-20					MTA2
V3SVHSHC_6567785	SORCS3				5.016E-20					SORCS3
V3SVHSHC_7495316	FAM120A				5.171E-20					FAM120A
V3SVHSHC_6655070	OR2T1				5.608E-20					OR2T1
V3SVHSHC_10646519	THSD7A				6.618E-20					THSD7A
V3SVHSHC_9009026	SCRN3				7.079E-20					SCRN3
V3SVHSHC_8024174	UNC13D				7.429E-20					UNC13D
V3SVHSHC_6596198	LYPLA2				7.614E-20					LYPLA2
V3SVHSHC_9836237	S100A1				7.842E-20					S100A1
V3SVHSHC_7404038	NAA35				8.319E-20					NAA35
V3SVHSHC_9982394	LYVE1				1.030E-19					LYVE1
V3SVHSHC_5675630	GPR162				1.043E-19					GPR162
V3SVHSHC_9130301	OR5M9				1.101E-19					OR5M9
V3SVHSHC_7926560	ATP6V1F				1.133E-19					ATP6V1F
V3SVHSHC_5190827	RGCC				1.134E-19					RGCC
V3SVHSHC_6568049	ANKRD62				1.863E-19					ANKRD62
V3SVHSHC_8472611	DHRS7C				1.939E-19					DHRS7C
V3SVHSHC_8103638	FFAR4				1.960E-19					FFAR4
V3SVHSHC_9918011	TULP4				2.168E-19					TULP4
V3SVHSHC_7238510	ZNF444				2.233E-19					ZNF444
V3SVHSHC_8873429	CEACAM4				2.257E-19					CEACAM4
V3SVHSHC_6132515	DCT				2.277E-19					DCT
V3SVHSHC_9568475	Tomm20l				2.280E-19					Tomm20l
V3SVHSHC_9391628	Agfg2				2.291E-19					Agfg2
V3SVHSHC_9584249	MED1				2.339E-19					MED1
V3SVHSHC_10148879	TSPAN7				2.371E-19					TSPAN7
V3SVHSHC_6427964	DUSP21				2.451E-19					DUSP21
V3SVHSHC_9962198	LRRC61				2.459E-19					LRRC61
V3SVHSHC_5614745	TTBK1				2.489E-19					TTBK1
V3SVHSHC_9153137	HSFY1				2.550E-19					HSFY1
V3SVHSHC_7560854	HJURP				2.806E-19					HJURP
V3SVHSHC_8723675	PSME4				3.208E-19					PSME4
V3SVHSHC_5010317	OSM				3.266E-19					OSM
V3SVHSHC_6964511	IL12RB2				3.270E-19					IL12RB2
V3SVHSHC_7669193	RAB34				3.422E-19					RAB34
V3SVHSHC_8034965	LY9				3.711E-19					LY9
V3SVHSHC_6728594	VDR				3.935E-19					VDR
V3SVHSHC_5587850	OSTM1				4.421E-19					OSTM1
V3SVHSHC_9316685	C16orf93				4.604E-19					C16orf93

V3SVHSHC_10537157	SDK1				4.940E-19					SDK1
V3SVHSHC_5961839	ZNF578				5.966E-19					ZNF578
V3SVHSHC_6434168	DMC1				6.102E-19					DMC1
V3SVHSHC_9963650	PSAT1				6.109E-19					PSAT1
V3SVHSHC_8991536	ANKRD1				6.417E-19					ANKRD1
V3SVHSHC_8038826	Fam131c				6.704E-19					Fam131c
V3SVHSHC_5265803	ALDH6A1				6.893E-19					ALDH6A1
V3SVHSHC_9837722	HYAL1				6.927E-19					HYAL1
V3SVHSHC_9411131	DEFB103A				6.957E-19					DEFB103A
V3SVHSHC_10313120	C14orf177				7.299E-19					C14orf177
V3SVHSHC_8733278	CNTF				7.453E-19					CNTF
V3SVHSHC_5945174	DCP1B				7.697E-19					DCP1B
V3SVHSHC_9854387	TMEM184C				8.119E-19					TMEM184 C
V3SVHSHC_5852015	CERCAM				8.249E-19					CERCAM
V3SVHSHC_6632267	EGLN2				8.499E-19					EGLN2
V3SVHSHC_4767008	ZDHHC9				9.055E-19					ZDHHC9
V3SVHSHC_8350841	LOC101929804				9.276E-19					LOC10192 9804
V3SVHSHC_9266690	WFIKKN2				1.007E-18					WFIKKN2
V3SVHSHC_5965403	GRINA				1.046E-18					GRINA
V3SVHSHC_5386649	SLC5A4				1.098E-18					SLC5A4
V3SVHSHC_7152116	UNK				1.111E-18					UNK
V3SVHSHC_6942830	PLEKHA1				1.372E-18					PLEKHA1
V3SVHSHC_9468551	CSNK2A1				1.386E-18					CSNK2A1
V3SVHSHC_7543892	LOC100505549				1.430E-18					LOC10050 5549
V3SVHSHC_7982594	CYR61				1.510E-18					CYR61
V3SVHSHC_10770995	MUC5B				1.613E-18					MUC5B
V3SVHSHC_9216002	PTCH1				1.670E-18					PTCH1
V3SVHSHC_7072817	MPHOSPH9				1.766E-18					MPHOSP H9
V3SVHSHC_10040276	USP12				1.850E-18					USP12
V3SVHSHC_9852407	WDTC1				1.875E-18					WDTC1
V3SVHSHC_9892700	KAZN				1.965E-18					KAZN
V3SVHSHC_6723281	C15orf32				1.975E-18					C15orf32
V3SVHSHC_5666489	OR5K1				2.011E-18					OR5K1
V3SVHSHC_5832578	SLC26A11				2.054E-18					SLC26A11
V3SVHSHC_5535545	ASAH2B				2.065E-18					ASAH2B
V3SVHSHC_9306158	ANGPT4				2.148E-18					ANGPT4
V3SVHSHC_10720967	RNF145				2.204E-18					RNF145
V3SVHSHC_8953685	TEX11				2.372E-18					TEX11
V3SVHSHC_8848679	LDHB				2.518E-18					LDHB
V3SVHSHC_6096083	CD9				2.699E-18					CD9
V3SVHSHC_10136438	CLDN18				3.383E-18					CLDN18
V3SVHSHC_4752620	BFSP1				3.582E-18					BFSP1
V3SVHSHC_6926066	CHRM3				3.636E-18					CHRM3
V3SVHSHC_6623489	STH				3.668E-18					STH
V3SVHSHC_6861056	HCAR3				3.673E-18					HCAR3
V3SVHSHC_7386482	ELAC2				3.942E-18					ELAC2
V3SVHSHC_10463105	LOC100996619				3.953E-18					LOC10099 6619
V3SVHSHC_7531418	ERGIC2				4.279E-18					ERGIC2
V3SVHSHC_9548510	PTDSS2				4.389E-18					PTDSS2
V3SVHSHC_5330153	GDAP2				4.613E-18					GDAP2

V3SVHSHC_10667177	ZBED3				4.648E-18					ZBED3
V3SVHSHC_8719451	BLCAP				4.952E-18					BLCAP
V3SVHSHC_7865675	ETV7				5.080E-18					ETV7
V3SVHSHC_9830957	ATP5SL				5.876E-18					ATP5SL
V3SVHSHC_10098950	CLDND1				6.039E-18					CLDND1
V3SVHSHC_10648763	PITPNA				6.465E-18					PITPNA
V3SVHSHC_8352029	PARL				6.488E-18					PARL
V3SVHSHC_8629229	NIPBL				6.636E-18					NIPBL
V3SVHSHC_7698002	IDH3G				7.658E-18					IDH3G
V3SVHSHC_7795583	RAD23A				8.052E-18					RAD23A
V3SVHSHC_10838150	RRP1				8.740E-18					RRP1
V3SVHSHC_8156075	SRCIN1				9.029E-18					SRCIN1
V3SVHSHC_10600517	SENP2				9.853E-18					SENP2
V3SVHSHC_6872144	RIC8A				1.082E-17					RIC8A
V3SVHSHC_8071331	OGFRL1				1.105E-17					OGFRL1
V3SVHSHC_5294447	UPK3A				1.226E-17					UPK3A
V3SVHSHC_7313948	TOP1MT				1.236E-17					TOP1MT
V3SVHSHC_5011373	CLEC2A				1.265E-17					CLEC2A
V3SVHSHC_6738263	PHF14				1.286E-17					PHF14
V3SVHSHC_5843699	IFNL1				1.422E-17					IFNL1
V3SVHSHC_6863630	ZDHHC1				1.507E-17					ZDHHC1
V3SVHSHC_7645763	DMC1				1.685E-17					DMC1
V3SVHSHC_6145550	SOX5				1.801E-17					SOX5
V3SVHSHC_8807033	SUN2				1.843E-17					SUN2
V3SVHSHC_10170164	CDCP1				1.850E-17					CDCP1
V3SVHSHC_9856664	CCDC82				1.853E-17					CCDC82
V3SVHSHC_6404006	HLA-DRB4				1.923E-17					HLA-DRB4
V3SVHSHC_8706416	BPI				1.987E-17					BPI
V3SVHSHC_5543267	FADD				2.039E-17					FADD
V3SVHSHC_5358929	HPS6				2.124E-17					HPS6
V3SVHSHC_9290318	C9ORF37				2.379E-17					C9ORF37
V3SVHSHC_6678995	ZNF718				2.413E-17					ZNF718
V3SVHSHC_7797893	CBWD2				2.581E-17					CBWD2
V3SVHSHC_8575505	Fam154b				2.668E-17					Fam154b
V3SVHSHC_9234251	PPP1R14A				2.743E-17					PPP1R14 A
V3SVHSHC_4695992	RAB9A				3.035E-17					RAB9A
V3SVHSHC_8712785	ADH7				3.183E-17					ADH7
V3SVHSHC_10597613	MCOLN1				3.255E-17					MCOLN1
V3SVHSHC_8948438	BAZ1B				3.285E-17					BAZ1B
V3SVHSHC_7566398	STYK1				3.324E-17					STYK1
V3SVHSHC_5930159	Acap3				3.405E-17					Acap3
V3SVHSHC_6824459	Rrp7a				3.486E-17					Rrp7a
V3SVHSHC_10531118	GPR182				3.527E-17					GPR182
V3SVHSHC_6453110	PDSS1				3.787E-17					PDSS1
V3SVHSHC_6704867	C9orf92				3.936E-17					C9orf92
V3SVHSHC_8291474	CACNG7				4.219E-17					CACNG7
V3SVHSHC_4733216	PTN				4.226E-17					PTN
V3SVHSHC_9720011	GALNT1				4.648E-17					GALNT1
V3SVHSHC_6570986	CACHD1				4.750E-17					CACHD1
V3SVHSHC_7292630	AR				5.066E-17					AR
V3SVHSHC_4669922	CNTN3				5.115E-17					CNTN3
V3SVHSHC_7345727	DUSP26				5.942E-17					DUSP26
V3SVHSHC_5939465	Prss58				6.421E-17					Prss58
V3SVHSHC_5283623	GKAP1				6.746E-17					GKAP1

V3SVHSHC_6225773	RPS4Y2				6.953E-17					RPS4Y2
V3SVHSHC_10384928	ADAMTSL3				6.987E-17					ADAMTSL3
V3SVHSHC_6214850	DNAJB4				6.996E-17					DNAJB4
V3SVHSHC_10519898	CLIC3				7.328E-17					CLIC3
V3SVHSHC_7422617	SLC25A22				7.923E-17					SLC25A22
V3SVHSHC_8398691	COX7A1				8.246E-17					COX7A1
V3SVHSHC_5950289	DZIP1L				9.080E-17					DZIP1L
V3SVHSHC_5422883	FADS3				9.354E-17					FADS3
V3SVHSHC_7720409	TMEM33				9.471E-17					TMEM33
V3SVHSHC_6094268	KRTAP12-4				9.949E-17					KRTAP12-4
V3SVHSHC_9573458	DIS3L				1.006E-16					DIS3L
V3SVHSHC_7739978	LIMA1				1.034E-16					LIMA1
V3SVHSHC_7105850	HDGFRP3				1.063E-16					HDGFRP3
V3SVHSHC_8326355	HMGN2				1.064E-16					HMGN2
V3SVHSHC_7886366	SLC5A4				1.096E-16					SLC5A4
V3SVHSHC_6875477	MYOZ1				1.097E-16					MYOZ1
V3SVHSHC_9036845	DPH1				1.100E-16					DPH1
V3SVHSHC_5104466	LCN2				1.160E-16					LCN2
V3SVHSHC_5205512	SERPINH1				1.231E-16					SERPINH1
V3SVHSHC_10202141	C1orf61				1.294E-16					C1orf61
V3SVHSHC_9313319	ANKH				1.357E-16					ANKH
V3SVHSHC_8129840	RTN4				1.399E-16					RTN4
V3SVHSHC_8788817	NAA60				1.524E-16					NAA60
V3SVHSHC_9361928	F8A1				1.653E-16					F8A1
V3SVHSHC_7198052	ERP44				1.661E-16					ERP44
V3SVHSHC_7494260	PLEKHF1				1.722E-16					PLEKHF1
V3SVHSHC_7154426	FASLG				1.836E-16					FASLG
V3SVHSHC_9454955	HOXA7				1.882E-16					HOXA7
V3SVHSHC_10632032	C1GALT1C1				2.195E-16					C1GALT1C1
V3SVHSHC_8248607	GPR68				2.196E-16					GPR68
V3SVHSHC_8033480	USF2				2.282E-16					USF2
V3SVHSHC_9394037	PDK4				2.414E-16					PDK4
V3SVHSHC_9251807	NT5C1A				2.741E-16					NT5C1A
V3SVHSHC_5996489	COL16A1				2.871E-16					COL16A1
V3SVHSHC_7460369	WIZ				2.879E-16					WIZ
V3SVHSHC_8922599	LHX3				2.932E-16					LHX3
V3SVHSHC_5129579	NUDT1				3.105E-16					NUDT1
V3SVHSHC_8633783	MIER2				3.108E-16					MIER2
V3SVHSHC_5539208	SLC22A23				3.507E-16					SLC22A23
V3SVHSHC_8748920	TDP2				3.529E-16					TDP2
V3SVHSHC_10847720	SMIM9				3.783E-16					SMIM9
V3SVHSHC_8388494	SNUPN				3.950E-16					SNUPN
V3SVHSHC_8105585	OR4P4				4.425E-16					OR4P4
V3SVHSHC_4720445	CPSF4L				4.615E-16					CPSF4L
V3SVHSHC_7886399	MAPKAPK3				5.032E-16					MAPKAPK3
V3SVHSHC_10480793	ZNF576				5.223E-16					ZNF576
V3SVHSHC_6953522	Dlgap5				5.425E-16					Dlgap5
V3SVHSHC_9617942	TARBP2				6.110E-16					TARBP2
V3SVHSHC_5250293	CYP46A1				6.847E-16					CYP46A1
V3SVHSHC_7784561	CXCL14				7.709E-16					CXCL14
V3SVHSHC_6795881	CLEC4G				8.912E-16					CLEC4G

V3SVHSHC_9504224	RB1CC1				9.089E-16					RB1CC1
V3SVHSHC_5074370	HOXB5				9.555E-16					HOXB5
V3SVHSHC_9396908	PAX7				9.831E-16					PAX7
V3SVHSHC_8430338	CEP250				1.043E-15					CEP250
V3SVHSHC_5806640	SPTY2D1				1.083E-15					SPTY2D1
V3SVHSHC_8500166	SLC15A4				1.124E-15					SLC15A4
V3SVHSHC_10586360	CD6				1.340E-15					CD6
V3SVHSHC_7533629	FGF9				1.347E-15					FGF9
V3SVHSHC_8103374	COL24A1				1.359E-15					COL24A1
V3SVHSHC_10715324	MTDH				1.385E-15					MTDH
V3SVHSHC_8334638	XKR3				1.418E-15					XKR3
V3SVHSHC_6441032	CDKL5				1.545E-15					CDKL5
V3SVHSHC_9000380	Stx1b				1.564E-15					Stx1b
V3SVHSHC_7770371	ARPC3				1.751E-15					ARPC3
V3SVHSHC_5991440	GJC3				1.923E-15					GJC3
V3SVHSHC_4832678	PLS3				1.932E-15					PLS3
V3SVHSHC_10389878	VEGFC				2.078E-15					VEGFC
V3SVHSHC_7306523	TBX2				2.123E-15					TBX2
V3SVHSHC_10793402	TUFM				2.126E-15					TUFM
V3SVHSHC_8986388	FAM160B2				2.138E-15					FAM160B2
V3SVHSHC_6930950	OR14I1				2.482E-15					OR14I1
V3SVHSHC_10443767	ARFIP1				2.639E-15					ARFIP1
V3SVHSHC_5955833	LOC100996405				2.918E-15					LOC10099<br>6405
V3SVHSHC_5931347	KCNA5				3.052E-15					KCNA5
V3SVHSHC_5501885	XYLT2				3.069E-15					XYLT2
V3SVHSHC_6242207	HSD11B1				3.346E-15					HSD11B1
V3SVHSHC_7231745	MCAT				3.426E-15					MCAT
V3SVHSHC_10352852	PHF21A				3.851E-15					PHF21A
V3SVHSHC_9460961	OS9				4.336E-15					OS9
V3SVHSHC_10679222	Lrrc16a				4.551E-15					Lrrc16a
V3SVHSHC_10587152	S1PR2				4.733E-15					S1PR2
V3SVHSHC_8190593	COX16				5.276E-15					COX16
V3SVHSHC_6656225	PHOX2A				5.284E-15					PHOX2A
V3SVHSHC_7105619	EGFL6				5.406E-15					EGFL6
V3SVHSHC_9106112	C10ORF76				5.518E-15					C10ORF7<br>6
V3SVHSHC_10561610	HS6ST2				5.763E-15					HS6ST2
V3SVHSHC_6554255	CSF1R				5.809E-15					CSF1R
V3SVHSHC_5210594	COL2A1				5.869E-15					COL2A1
V3SVHSHC_7407305	PPL				5.873E-15					PPL
V3SVHSHC_6561251	LY75-CD302				5.898E-15					LY75-<br>CD302
V3SVHSHC_5799413	RNF141				6.337E-15					RNF141
V3SVHSHC_10473104	MPEG1				6.534E-15					MPEG1
V3SVHSHC_8953223	RHOV				6.559E-15					RHOV
V3SVHSHC_5347610	PDCD2L				6.895E-15					PDCD2L
V3SVHSHC_8452448	GPN3				7.023E-15					GPN3
V3SVHSHC_8332163	OR4M2				8.015E-15					OR4M2
V3SVHSHC_6536897	MYO18B				8.079E-15					MYO18B
V3SVHSHC_6871649	MORC2				8.736E-15					MORC2
V3SVHSHC_7871318	FBXL8				9.143E-15					FBXL8
V3SVHSHC_10188413	FGFR3				9.678E-15					FGFR3
V3SVHSHC_10847687	USP22				1.005E-14					USP22
V3SVHSHC_8811587	ZNF324B				1.033E-14					ZNF324B

V3SVHSHC_5585309	LRRC70				1.050E-14					LRRC70
V3SVHSHC_8433110	PCDHB12				1.061E-14					PCDHB12
V3SVHSHC_8526170	FSCN2				1.082E-14					FSCN2
V3SVHSHC_6810764	PDCD4				1.092E-14					PDCD4
V3SVHSHC_7618637	OSBP				1.116E-14					OSBP
V3SVHSHC_7327280	BAZ1A				1.193E-14					BAZ1A
V3SVHSHC_10213163	FBXO9				1.247E-14					FBXO9
V3SVHSHC_9086477	ZNF501				1.310E-14					ZNF501
V3SVHSHC_5174624	HTRA1				1.332E-14					HTRA1
V3SVHSHC_7329425	ZNF208				1.438E-14					ZNF208
V3SVHSHC_5201453	TESK1				1.547E-14					TESK1
V3SVHSHC_10475810	LRRC49				1.612E-14					LRRC49
V3SVHSHC_6829673	AGGF1				1.797E-14					AGGF1
V3SVHSHC_7067042	POLD4				1.805E-14					POLD4
V3SVHSHC_6849572	C16orf72				1.883E-14					C16orf72
V3SVHSHC_9751526	IL6ST				1.920E-14					IL6ST
V3SVHSHC_8098226	PAFAH1B2				2.151E-14					PAFAH1B 2
V3SVHSHC_5151194	DNAJC10				2.214E-14					DNAJC10
V3SVHSHC_10235174	DSCAM				2.315E-14					DSCAM
V3SVHSHC_7189010	ATRAID				2.475E-14					ATRAID
V3SVHSHC_7754003	RCE1				2.551E-14					RCE1
V3SVHSHC_5189342	PCBP4				3.015E-14					PCBP4
V3SVHSHC_8978666	CLDN16				3.084E-14					CLDN16
V3SVHSHC_4657547	LSMEM1				3.229E-14					LSMEM1
V3SVHSHC_7190264	SCARB2				3.237E-14					SCARB2
V3SVHSHC_8140400	PLEKHO1				3.811E-14					PLEKHO1
V3SVHSHC_9062948	SAMD10				3.821E-14					SAMD10
V3SVHSHC_6255176	TAX1BP1				4.020E-14					TAX1BP1
V3SVHSHC_5556302	EVA1A				4.087E-14					EVA1A
V3SVHSHC_7134461	NRD1				4.112E-14					NRD1
V3SVHSHC_7291145	SLC31A1				4.159E-14					SLC31A1
V3SVHSHC_5494724	MTFR1				4.322E-14					MTFR1
V3SVHSHC_10246262	ZNF710				4.355E-14					ZNF710
V3SVHSHC_5401829	OGN				4.389E-14					OGN
V3SVHSHC_7303850	UGT2B11				4.473E-14					UGT2B11
V3SVHSHC_6716978	EFCC1				4.483E-14					EFCC1
V3SVHSHC_4649132	PRAMEF6				4.561E-14					PRAMEF6
V3SVHSHC_7235045	ITGB5				4.897E-14					ITGB5
V3SVHSHC_10562435	UPK1A				5.026E-14					UPK1A
V3SVHSHC_10676219	FBLN7				5.034E-14					FBLN7
V3SVHSHC_6911876	MLLT3				5.144E-14					MLLT3
V3SVHSHC_8203364	ZNF513				5.327E-14					ZNF513
V3SVHSHC_10553030	ZNF8				5.496E-14					ZNF8
V3SVHSHC_6259466	CD47				6.378E-14					CD47
V3SVHSHC_6389057	VCPIP1				6.964E-14					VCPIP1
V3SVHSHC_8239103	HAVCR2				7.349E-14					HAVCR2
V3SVHSHC_5410640	SUMO4				7.761E-14					SUMO4
V3SVHSHC_7319030	EHD3				8.086E-14					EHD3
V3SVHSHC_8333120	MUC5AC				9.004E-14					MUC5AC
V3SVHSHC_4865414	PDHX				9.693E-14					PDHX
V3SVHSHC_7485614	CDC42BPG				9.753E-14					CDC42BP G
V3SVHSHC_6099053	FHAD1				1.014E-13					FHAD1
V3SVHSHC_9373841	POLD3				1.234E-13					POLD3

V3SVHSHC_6664838	OR8B2				1.341E-13				OR8B2
V3SVHSHC_8705723	ACOT11				1.435E-13				ACOT11
V3SVHSHC_9704171	TSPAN2				1.602E-13				TSPAN2
V3SVHSHC_8109479	PALM				1.708E-13				PALM
V3SVHSHC_9602663	MB				1.780E-13				MB
V3SVHSHC_6839672	SRCAP				1.973E-13				SRCAP
V3SVHSHC_4702229	ATL2				2.011E-13				ATL2
V3SVHSHC_6887720	POLR2F				2.014E-13				POLR2F
V3SVHSHC_8323055	MLLT4				2.213E-13				MLLT4
V3SVHSHC_9189932	FSTL1				2.292E-13				FSTL1
V3SVHSHC_9444494	UBR7				2.631E-13				UBR7
V3SVHSHC_5972762	KAAG1				2.681E-13				KAAG1
V3SVHSHC_7256132	SLC1A7				2.697E-13				SLC1A7
V3SVHSHC_6369059	ASCC3				2.832E-13				ASCC3
V3SVHSHC_6343022	PCYOX1				2.874E-13				PCYOX1
V3SVHSHC_10380638	BCAT2				2.966E-13				BCAT2
V3SVHSHC_5098328	ZNF623				2.992E-13				ZNF623
V3SVHSHC_7049783	UAP1L1				3.212E-13				UAP1L1
V3SVHSHC_10326221	CYB561				3.326E-13				CYB561
V3SVHSHC_9392519	KLK13				3.429E-13				KLK13
V3SVHSHC_9871679	FUT11				3.523E-13				FUT11
V3SVHSHC_7314839	MTERFD3				4.742E-13				MTERFD3
V3SVHSHC_6752651	ENKD1				4.990E-13				ENKD1
V3SVHSHC_6203696	C3orf27				5.072E-13				C3orf27
V3SVHSHC_4648406	OR5AU1				5.126E-13				OR5AU1
V3SVHSHC_5264945	LOC101928498				5.363E-13				LOC10192 8498
V3SVHSHC_6171323	NEU2				5.554E-13				NEU2
V3SVHSHC_10462907	MCHR1				5.735E-13				MCHR1
V3SVHSHC_5542838	TMEM114				6.365E-13				TMEM114
V3SVHSHC_7134857	TMTC2				6.524E-13				TMTC2
V3SVHSHC_10550588	ZP2				7.370E-13				ZP2
V3SVHSHC_7512872	TCTEX1D4				7.909E-13				TCTEX1D 4
V3SVHSHC_7757105	SLC25A33				7.938E-13				SLC25A33
V3SVHSHC_4841654	LCE5A				8.124E-13				LCE5A
V3SVHSHC_6270356	RPL8				8.354E-13				RPL8
V3SVHSHC_6436313	PTPRG				8.368E-13				PTPRG
V3SVHSHC_9946457	ATF7IP2				8.392E-13				ATF7IP2
V3SVHSHC_7440404	ARSE				8.496E-13				ARSE
V3SVHSHC_6703844	CMTM5				8.553E-13				CMTM5
V3SVHSHC_8850923	TPSG1				9.106E-13				TPSG1
V3SVHSHC_10658828	LOC101060400				9.305E-13				LOC10106 0400
V3SVHSHC_6046715	CASP16				9.453E-13				CASP16
V3SVHSHC_7918970	SETD9				9.517E-13				SETD9
V3SVHSHC_5180069	ZNF517				1.004E-12				ZNF517
V3SVHSHC_6238511	PCBP3				1.036E-12				PCBP3
V3SVHSHC_7841486	MTIF2				1.039E-12				MTIF2
V3SVHSHC_8024405	GSK3B				1.086E-12				GSK3B
V3SVHSHC_7164095	RASD1				1.142E-12				RASD1
V3SVHSHC_10097465	CAPN12				1.167E-12				CAPN12
V3SVHSHC_9590585	PIANP				1.320E-12				PIANP
V3SVHSHC_8181617	KRTAP13-1				1.431E-12				KRTAP13-1

V3SVHSHC_6335597	BIK				1.542E-12					BIK
V3SVHSHC_9642329	ASF1B				1.571E-12					ASF1B
V3SVHSHC_9174917	LRIG3				1.635E-12					LRIG3
V3SVHSHC_6028334	LEFTY1				1.779E-12					LEFTY1
V3SVHSHC_4980188	COPRS				1.819E-12					COPRS
V3SVHSHC_5829377	ASNSD1				1.828E-12					ASNSD1
V3SVHSHC_8286623	EDRF1				1.868E-12					EDRF1
V3SVHSHC_5416679	SLC35E2				1.872E-12					SLC35E2
V3SVHSHC_4926068	ARHGEF11				1.930E-12					ARHGEF1 1
V3SVHSHC_8856434	C3orf62				2.101E-12					C3orf62
V3SVHSHC_7940717	SMIM11				2.269E-12					SMIM11
V3SVHSHC_10391231	LHFPL5				2.284E-12					LHFPL5
V3SVHSHC_10703840	ARHGEF3				2.311E-12					ARHGEF3
V3SVHSHC_6427601	TMEM175				2.321E-12					TMEM175
V3SVHSHC_6936692	VNN2				2.334E-12					VNN2
V3SVHSHC_7794956	BBIP1				2.640E-12					BBIP1
V3SVHSHC_10618172	ST7				2.693E-12					ST7
V3SVHSHC_10025426	CXCL3				2.758E-12					CXCL3
V3SVHSHC_8607647	LDHAL6A				2.941E-12					LDHAL6A
V3SVHSHC_6097766	PMP2				3.481E-12					PMP2
V3SVHSHC_9003218	PCSK7				3.494E-12					PCSK7
V3SVHSHC_7695296	SNX25				3.622E-12					SNX25
V3SVHSHC_10808417	CAPN3				3.855E-12					CAPN3
V3SVHSHC_8708231	AGXT				4.411E-12					AGXT
V3SVHSHC_8101922	DNMT1				4.796E-12					DNMT1
V3SVHSHC_6442979	ATP6V0E1				4.854E-12					ATP6V0E1
V3SVHSHC_9917219	KCTD17				5.654E-12					KCTD17
V3SVHSHC_5889173	SLC18A3				5.863E-12					SLC18A3
V3SVHSHC_6912239	RNF7				5.972E-12					RNF7
V3SVHSHC_5821622	LRRC43				6.763E-12					LRRC43
V3SVHSHC_8225243	MYO7B				7.191E-12					MYO7B
V3SVHSHC_9227849	DIDO1				7.613E-12					DIDO1
V3SVHSHC_7503038	GLRA2				7.679E-12					GLRA2
V3SVHSHC_5379323	HSPA6				7.888E-12					HSPA6
V3SVHSHC_7325069	SLC9B2				8.055E-12					SLC9B2
V3SVHSHC_6124496	BMPR2				9.206E-12					BMPR2
V3SVHSHC_5277749	BRSK2				9.770E-12					BRSK2
V3SVHSHC_8981306	JAK3				9.811E-12					JAK3
V3SVHSHC_9995528	PLXNA2				9.944E-12					PLXNA2
V3SVHSHC_8284082	SCAP				1.068E-11					SCAP
V3SVHSHC_5113838	CLPB				1.129E-11					CLPB
V3SVHSHC_5608805	Zcchc18				1.229E-11					Zcchc18
V3SVHSHC_6271016	CSNK1G1				1.251E-11					CSNK1G1
V3SVHSHC_5564618	HERC5				1.255E-11					HERC5
V3SVHSHC_7060871	MAGEA10				1.318E-11					MAGEA10
V3SVHSHC_10267844	FOXJ3				1.379E-11					FOXJ3
V3SVHSHC_4809512	ZMIZ2				1.485E-11					ZMIZ2
V3SVHSHC_10240982	CCDC68				1.563E-11					CCDC68
V3SVHSHC_9608570	PRPH2				1.704E-11					PRPH2
V3SVHSHC_6464462	SHC4				1.889E-11					SHC4
V3SVHSHC_9302000	ZNF891				2.043E-11					ZNF891
V3SVHSHC_6104762	BAGE3				2.047E-11					BAGE3
V3SVHSHC_5359919	CBFB				2.076E-11					CBFB
V3SVHSHC_6553496	Tox2				2.292E-11					Tox2

V3SVHSHC_6638603	ZBTB2				2.354E-11					ZBTB2
V3SVHSHC_5279399	SPATA31A6				2.357E-11					SPATA31 A6
V3SVHSHC_9925931	TMEM63B				2.368E-11					TMEM63B
V3SVHSHC_6630122	GIMAP7				2.510E-11					GIMAP7
V3SVHSHC_6806936	CISD3				2.625E-11					CISD3
V3SVHSHC_8236595	ZFP62				2.763E-11					ZFP62
V3SVHSHC_7635038	RPP25L				2.898E-11					RPP25L
V3SVHSHC_6309956	FBXO32				2.910E-11					FBXO32
V3SVHSHC_6770801	HOPX				2.960E-11					HOPX
V3SVHSHC_9933851	CD300LD				3.020E-11					CD300LD
V3SVHSHC_6894386	CDH2				3.095E-11					CDH2
V3SVHSHC_8815118	RBM42				3.137E-11					RBM42
V3SVHSHC_9424529	ADAMTSL1				3.260E-11					ADAMTSL 1
V3SVHSHC_9222833	SH2D6				3.301E-11					SH2D6
V3SVHSHC_4750640	ZNF747				3.599E-11					ZNF747
V3SVHSHC_5339327	PML				3.648E-11					PML
V3SVHSHC_5596529	NOTCH2				3.794E-11					NOTCH2
V3SVHSHC_9375722	LRRC25				3.847E-11					LRRC25
V3SVHSHC_8439710	NT5DC4				4.133E-11					NT5DC4
V3SVHSHC_6898214	SPATA31A6				4.197E-11					SPATA31 A6
V3SVHSHC_10265369	MTHFR				4.697E-11					MTHFR
V3SVHSHC_9480497	KRIT1				4.814E-11					KRIT1
V3SVHSHC_6283193	ZNF589				5.339E-11					ZNF589
V3SVHSHC_6084995	SLC35A3				5.362E-11					SLC35A3
V3SVHSHC_8579234	FAM155A				5.405E-11					FAM155A
V3SVHSHC_9449774	VIL1				5.487E-11					VIL1
V3SVHSHC_9040904	HSD11B1L				5.817E-11					HSD11B1L
V3SVHSHC_8810003	SCAMP2				5.939E-11					SCAMP2
V3SVHSHC_7605074	TEX101				5.942E-11					TEX101
V3SVHSHC_9003713	NDRG4				6.099E-11					NDRG4
V3SVHSHC_6584384	TRIM39-RPP21				6.638E-11					TRIM39-RPP21
V3SVHSHC_9100007	SSPN				6.714E-11					SSPN
V3SVHSHC_9791621	Prrt4				6.902E-11					Prrt4
V3SVHSHC_9246527	HHLA2				6.994E-11					HHLA2
V3SVHSHC_4953227	SENP8				6.996E-11					SENP8
V3SVHSHC_7184357	LOC101928044				7.120E-11					LOC10192 8044
V3SVHSHC_8038859	ANXA1				7.286E-11					ANXA1
V3SVHSHC_8158880	XCL1				7.319E-11					XCL1
V3SVHSHC_5516966	ELL2				7.436E-11					ELL2
V3SVHSHC_6566828	C18ORF21				7.531E-11					C18ORF2 1
V3SVHSHC_5464133	LSM4				7.865E-11					LSM4
V3SVHSHC_6650582	SOCS5				7.917E-11					SOCS5
V3SVHSHC_8741627	PCDHB11				8.118E-11					PCDHB11
V3SVHSHC_10060142	ZSCAN2				8.371E-11					ZSCAN2
V3SVHSHC_9230819	DHX36				8.705E-11					DHX36
V3SVHSHC_9249827	NKAPL				9.019E-11					NKAPL
V3SVHSHC_7624049	SIGIRR				9.187E-11					SIGIRR
V3SVHSHC_6320483	HSPBAP1				9.271E-11					HSPBAP1
V3SVHSHC_8915735	TGM4				9.837E-11					TGM4
V3SVHSHC_7246166	DAZAP2				1.082E-10					DAZAP2

V3SVHSHC_6899930	KIAA0391				1.116E-10					KIAA0391
V3SVHSHC_6558281	OLA1				1.140E-10					OLA1
V3SVHSHC_4787336	HOXD9				1.183E-10					HOXD9
V3SVHSHC_7437038	ERBB2IP				1.184E-10					ERBB2IP
V3SVHSHC_5407472	FRMD5				1.205E-10					FRMD5
V3SVHSHC_6944942	LMBR1L				1.213E-10					LMBR1L
V3SVHSHC_8707307	TTC21B				1.215E-10					TTC21B
V3SVHSHC_4736516	LOC101929803				1.227E-10					LOC10192 9803
V3SVHSHC_7695263	OTOF				1.233E-10					OTOF
V3SVHSHC_8199536	TRIM63				1.269E-10					TRIM63
V3SVHSHC_8038958	RGS17				1.278E-10					RGS17
V3SVHSHC_7812974	CDHR3				1.451E-10					CDHR3
V3SVHSHC_7048133	SPTBN4				1.480E-10					SPTBN4
V3SVHSHC_8143733	OTOP2				1.551E-10					OTOP2
V3SVHSHC_8818748	SOCS2				1.560E-10					SOCS2
V3SVHSHC_4872740	MMD				1.604E-10					MMD
V3SVHSHC_5964413	WBSCR27				1.624E-10					WBSCR27
V3SVHSHC_5322596	MYOZ3				1.633E-10					MYOZ3
V3SVHSHC_8471060	CCDC28B				1.661E-10					CCDC28B
V3SVHSHC_8576363	FOXS1				1.830E-10					FOXS1
V3SVHSHC_5554223	ARFGAP1				1.914E-10					ARFGAP1
V3SVHSHC_10044071	SCARB2				1.919E-10					SCARB2
V3SVHSHC_10193462	SYN3				2.017E-10					SYN3
V3SVHSHC_4775093	IGFBP7				2.056E-10					IGFBP7
V3SVHSHC_5040380	RNASE2				2.156E-10					RNASE2
V3SVHSHC_6843500	IMMT				2.324E-10					IMMT
V3SVHSHC_5292335	C20ORF27				2.347E-10					C20ORF2 7
V3SVHSHC_5785322	LOC101059976				2.471E-10					LOC10105 9976
V3SVHSHC_5635238	DDX3X				2.476E-10					DDX3X
V3SVHSHC_6931709	SLC10A7				2.486E-10					SLC10A7
V3SVHSHC_10154819	IL27RA				2.497E-10					IL27RA
V3SVHSHC_7889204	AGO4				2.511E-10					AGO4
V3SVHSHC_6227423	PGP				2.670E-10					PGP
V3SVHSHC_8506832	PCTP				2.674E-10					PCTP
V3SVHSHC_10209467	TACSTD2				2.724E-10					TACSTD2
V3SVHSHC_8993714	NUP210				2.729E-10					NUP210
V3SVHSHC_7006355	HLA-DOA				2.869E-10					HLA-DOA
V3SVHSHC_10325627	LOH12CR1				2.931E-10					LOH12CR 1
V3SVHSHC_5734271	FAM103A1				2.995E-10					FAM103A1
V3SVHSHC_5535512	FRYL				3.133E-10					FRYL
V3SVHSHC_9597548	CCDC121				3.238E-10					CCDC121
V3SVHSHC_9062321	EMC4				3.361E-10					EMC4
V3SVHSHC_6704207	PRKAG3				3.380E-10					PRKAG3
V3SVHSHC_6541352	VCPIP1				3.410E-10					VCPIP1
V3SVHSHC_9972032	RNF186				3.552E-10					RNF186
V3SVHSHC_7956722	MYOF				3.591E-10					MYOF
V3SVHSHC_10458386	GCM1				3.682E-10					GCM1
V3SVHSHC_5655302	KIF27				3.686E-10					KIF27
V3SVHSHC_9262037	DENND4A				4.022E-10					DENND4A
V3SVHSHC_9005660	GMNN				4.144E-10					GMNN
V3SVHSHC_9201746	KNG1				4.149E-10					KNG1
V3SVHSHC_7832741	FER1L6				4.229E-10					FER1L6

V3SVHSHC_9922598	DOCK11				4.268E-10					DOCK11
V3SVHSHC_7403939	TNFSF12				4.292E-10					TNFSF12
V3SVHSHC_7395392	PAOX				4.702E-10					PAOX
V3SVHSHC_10480067	PCDHGC4				4.909E-10					PCDHGC4
V3SVHSHC_6040082	SLC25A25				5.286E-10					SLC25A25
V3SVHSHC_7381895	LMTK2				5.427E-10					LMTK2
V3SVHSHC_5243561	NHS				5.476E-10					NHS
V3SVHSHC_7234880	SLC35B4				5.539E-10					SLC35B4
V3SVHSHC_8072057	MGARP				5.827E-10					MGARP
V3SVHSHC_6842147	RAB30				5.960E-10					RAB30
V3SVHSHC_5123672	MMP13				6.808E-10					MMP13
V3SVHSHC_7866830	PTPN13				7.797E-10					PTPN13
V3SVHSHC_8778488	TMEM156				7.843E-10					TMEM156
V3SVHSHC_9001634	FBXL17				8.025E-10					FBXL17
V3SVHSHC_10630877	GPR112				8.229E-10					GPR112
V3SVHSHC_8260619	C2ORF16				8.485E-10					C2ORF16
V3SVHSHC_4782386	BRINP1				8.558E-10					BRINP1
V3SVHSHC_9877619	P2RY8				9.342E-10					P2RY8
V3SVHSHC_10550621	Dcaf8				9.428E-10					Dcaf8
V3SVHSHC_6687806	IRX3				9.614E-10					IRX3
V3SVHSHC_8607713	MYO1F				1.007E-09					MYO1F
V3SVHSHC_5827001	PGK1				1.072E-09					PGK1
V3SVHSHC_8784263	ZNF280B				1.082E-09					ZNF280B
V3SVHSHC_5704736	CNGA2				1.083E-09					CNGA2
V3SVHSHC_6199142	FAM153B				1.116E-09					FAM153B
V3SVHSHC_6224024	GPR65				1.401E-09					GPR65
V3SVHSHC_5560889	SLAMF8				1.482E-09					SLAMF8
V3SVHSHC_10426211	USP51				1.502E-09					USP51
V3SVHSHC_5219669	ZNF600				1.558E-09					ZNF600
V3SVHSHC_9420404	CNST				1.658E-09					CNST
V3SVHSHC_5663090	DENND1C				1.673E-09					DENND1C
V3SVHSHC_10481387	C14ORF79				1.711E-09					C14ORF7 9
V3SVHSHC_9902600	REEP5				1.752E-09					REEP5
V3SVHSHC_5448656	PA2G4				1.767E-09					PA2G4
V3SVHSHC_7906991	CCKBR				1.793E-09					CCKBR
V3SVHSHC_7660613	CDY2B				1.858E-09					CDY2B
V3SVHSHC_8766872	ZNF579				1.994E-09					ZNF579
V3SVHSHC_10753076	ATL1				2.285E-09					ATL1
V3SVHSHC_10577417	Tmem185b				2.368E-09					Tmem185 b
V3SVHSHC_5526008	ABCA13				2.403E-09					ABCA13
V3SVHSHC_9625400	BAGE4				2.674E-09					BAGE4
V3SVHSHC_10528346	NEFL				2.864E-09					NEFL
V3SVHSHC_8016584	SERPINA1				3.002E-09					SERPINA1
V3SVHSHC_6139610	Plac9				3.151E-09					Plac9
V3SVHSHC_6634940	LOC101928044				3.189E-09					LOC10192 8044
V3SVHSHC_6842312	RASAL3				3.195E-09					RASAL3
V3SVHSHC_6151490	NAV3				3.232E-09					NAV3
V3SVHSHC_9022721	MCF2				3.255E-09					MCF2
V3SVHSHC_10442546	ZBTB42				3.265E-09					ZBTB42
V3SVHSHC_7061102	HINFP				3.267E-09					HINFP
V3SVHSHC_8194388	LYRM7				3.310E-09					LYRM7
V3SVHSHC_5705726	NOTCH1				3.438E-09					NOTCH1
V3SVHSHC_6806045	HLA-DPB1				3.483E-09					HLA-DPB1

V3SVHSHC_9122975	ANKRD13B				3.490E-09					ANKRD13B
V3SVHSHC_7998533	ACRBP				3.502E-09					ACRBP
V3SVHSHC_7722092	TEX11				3.652E-09					TEX11
V3SVHSHC_8737040	DUSP1				3.847E-09					DUSP1
V3SVHSHC_8178284	REL				3.946E-09					REL
V3SVHSHC_6682460	RASL11A				4.082E-09					RASL11A
V3SVHSHC_6704240	TLK1				4.098E-09					TLK1
V3SVHSHC_6032954	U2AF2				4.702E-09					U2AF2
V3SVHSHC_7749185	STOM				4.745E-09					STOM
V3SVHSHC_10773668	Dlk2				5.196E-09					Dlk2
V3SVHSHC_7902437	Fam71e1				6.151E-09					Fam71e1
V3SVHSHC_9410669	Tchh				6.169E-09					Tchh
V3SVHSHC_6338138	FAM110B				6.241E-09					FAM110B
V3SVHSHC_4718234	HTR2C				6.594E-09					HTR2C
V3SVHSHC_10784393	CYFIP1				6.860E-09					CYFIP1
V3SVHSHC_5236565	ATF1				7.824E-09					ATF1
V3SVHSHC_6508088	MAN2B2				7.912E-09					MAN2B2
V3SVHSHC_7457036	KIF5B				8.270E-09					KIF5B
V3SVHSHC_9911015	LANCL3				8.369E-09					LANCL3
V3SVHSHC_6698795	ODF3L1				8.988E-09					ODF3L1
V3SVHSHC_10132412	TRIB3				9.050E-09					TRIB3
V3SVHSHC_9178052	CD276				9.275E-09					CD276
V3SVHSHC_8265305	SYBU				9.315E-09					SYBU
V3SVHSHC_5725691	GAS2L3				9.407E-09					GAS2L3
V3SVHSHC_7709024	PTPN22				9.431E-09					PTPN22
V3SVHSHC_5759549	SNX4				1.026E-08					SNX4
V3SVHSHC_8269496	SLC6A5				1.050E-08					SLC6A5
V3SVHSHC_8776607	TULP2				1.067E-08					TULP2
V3SVHSHC_7513763	PAPPA2				1.071E-08					PAPPA2
V3SVHSHC_7518878	HYPK				1.088E-08					HYPK
V3SVHSHC_8433968	KANSL1L				1.118E-08					KANSL1L
V3SVHSHC_8238575	DUSP5				1.178E-08					DUSP5
V3SVHSHC_5116148	PRCP				1.253E-08					PRCP
V3SVHSHC_10199633	TMEM114				1.325E-08					TMEM114
V3SVHSHC_10235966	MRPL24				1.337E-08					MRPL24
V3SVHSHC_7433144	FBXL4				1.344E-08					FBXL4
V3SVHSHC_6142910	ENPEP				1.355E-08					ENPEP
V3SVHSHC_8849900	SENP2				1.455E-08					SENP2
V3SVHSHC_8584415	ELFN2				1.519E-08					ELFN2
V3SVHSHC_8930222	NIPBL				1.625E-08					NIPBL
V3SVHSHC_8042720	GAS2L2				1.708E-08					GAS2L2
V3SVHSHC_6667841	RGS6				1.779E-08					RGS6
V3SVHSHC_7447862	C1QTNF7				1.862E-08					C1QTNF7
V3SVHSHC_10051925	HIC2				1.887E-08					HIC2
V3SVHSHC_5771198	WBP2				1.950E-08					WBP2
V3SVHSHC_8437499	PRKD3				1.967E-08					PRKD3
V3SVHSHC_5228777	OR4K2				1.977E-08					OR4K2
V3SVHSHC_7035989	ACIN1				1.985E-08					ACIN1
V3SVHSHC_9851846	TOM1L1				1.989E-08					TOM1L1
V3SVHSHC_6980879	LOC391722				2.109E-08					LOC391722
V3SVHSHC_8784329	LOC100509091				2.290E-08					LOC100509091
V3SVHSHC_7151489	OPN1SW				2.384E-08					OPN1SW

V3SVHSHC_7163633	LOC100996844				2.438E-08					LOC100996844
V3SVHSHC_4637186	SLC4A7				2.499E-08					SLC4A7
V3SVHSHC_6877358	BUD13				2.589E-08					BUD13
V3SVHSHC_6561119	ZDHHC5				2.610E-08					ZDHHC5
V3SVHSHC_5087207	ULK2				2.649E-08					ULK2
V3SVHSHC_9389714	CAPZA1				2.688E-08					CAPZA1
V3SVHSHC_10048955	LOC101927614				2.709E-08					LOC101927614
V3SVHSHC_6710147	SNRPE				2.748E-08					SNRPE
V3SVHSHC_10438355	SGSM1				2.891E-08					SGSM1
V3SVHSHC_9526235	OR11A1				2.956E-08					OR11A1
V3SVHSHC_9389252	SLC6A6				3.038E-08					SLC6A6
V3SVHSHC_8447960	USP18				3.364E-08					USP18
V3SVHSHC_7722257	GAPDHS				3.426E-08					GAPDHS
V3SVHSHC_4917851	NFYA				3.533E-08					NFYA
V3SVHSHC_8190461	ELOVL7				3.563E-08					ELOVL7
V3SVHSHC_7664837	MYL3				3.572E-08					MYL3
V3SVHSHC_5667677	SVIL				3.588E-08					SVIL
V3SVHSHC_7627085	THEM5				3.760E-08					THEM5
V3SVHSHC_6368234	Kcnu1				3.925E-08					Kcnu1
V3SVHSHC_5010251	PROK1				4.074E-08					PROK1
V3SVHSHC_8860196	SEPT1				4.177E-08					SEPT1
V3SVHSHC_6422915	H2AFX				4.221E-08					H2AFX
V3SVHSHC_5392820	B4GALT6				4.715E-08					B4GALT6
V3SVHSHC_6982265	INPP4B				4.842E-08					INPP4B
V3SVHSHC_10420436	TRIM50				4.934E-08					TRIM50
V3SVHSHC_7628570	STARD7				5.039E-08					STARD7
V3SVHSHC_10487756	NCF1				5.211E-08					NCF1
V3SVHSHC_6524522	MRPL23				5.425E-08					MRPL23
V3SVHSHC_9987113	HBA2				5.560E-08					HBA2
V3SVHSHC_8733212	DNASE2B				5.566E-08					DNASE2B
V3SVHSHC_6120800	F11R				6.119E-08					F11R
V3SVHSHC_10840889	GZMM				6.267E-08					GZMM
V3SVHSHC_6927287	POU2F3				6.622E-08					POU2F3
V3SVHSHC_4988471	KRTAP11-1				6.707E-08					KRTAP11-1
V3SVHSHC_6397571	AKT1				6.767E-08					AKT1
V3SVHSHC_7098590	GGN				7.597E-08					GGN
V3SVHSHC_9615368	PYCR2				8.099E-08					PYCR2
V3SVHSHC_5329031	ARRDC5				8.665E-08					ARRDC5
V3SVHSHC_10058459	FOXD4L5				9.134E-08					FOXD4L5
V3SVHSHC_5416580	TVP23C-CDRT4				9.403E-08					TVP23C-CDRT4
V3SVHSHC_6973520	KAT2B				9.796E-08					KAT2B
V3SVHSHC_4964744	CD248				1.042E-07					CD248
V3SVHSHC_8561117	RARS2				1.156E-07					RARS2
V3SVHSHC_7599860	LOC100292952				1.252E-07					LOC100292952
V3SVHSHC_6052952	ZBTB41				1.256E-07					ZBTB41
V3SVHSHC_10267811	RAD50				1.258E-07					RAD50
V3SVHSHC_4827563	C7orf62				1.269E-07					C7orf62
V3SVHSHC_5847956	ARL14				1.290E-07					ARL14
V3SVHSHC_7274315	HLA-F				1.311E-07					HLA-F

V3SVHSHC_10586030	LOC100132004				1.328E-07					LOC10013 2004
V3SVHSHC_5003024	TTBK2				1.376E-07					TTBK2
V3SVHSHC_9197093	HDAC2				1.379E-07					HDAC2
V3SVHSHC_5691305	ADCK1				1.412E-07					ADCK1
V3SVHSHC_6317447	CFL2				1.448E-07					CFL2
V3SVHSHC_4924682	POU3F2				1.525E-07					POU3F2
V3SVHSHC_7348862	SLC12A3				1.532E-07					SLC12A3
V3SVHSHC_5867624	NTSR2				1.548E-07					NTSR2
V3SVHSHC_5876072	TSTA3				1.617E-07					TSTA3
V3SVHSHC_9402683	UQCRFS1				1.695E-07					UQCRFS1
V3SVHSHC_6918278	CCDC87				1.698E-07					CCDC87
V3SVHSHC_8754002	ARFIP2				1.711E-07					ARFIP2
V3SVHSHC_8286953	AIMP2				1.720E-07					AIMP2
V3SVHSHC_4754699	Eapp				1.744E-07					Eapp
V3SVHSHC_9600650	RNPEP				1.753E-07					RNPEP
V3SVHSHC_9956291	PLCG2				1.756E-07					PLCG2
V3SVHSHC_9794327	HSPA14				1.793E-07					HSPA14
V3SVHSHC_6357806	MAP3K6				1.995E-07					MAP3K6
V3SVHSHC_4980683	STEAP4				2.109E-07					STEAP4
V3SVHSHC_6333023	PNLIPRP3				2.213E-07					PNLIPRP3
V3SVHSHC_6018038	MARK1				2.380E-07					MARK1
V3SVHSHC_5523368	LOC100130301				2.456E-07					LOC10013 0301
V3SVHSHC_10062518	C1orf87				2.501E-07					C1orf87
V3SVHSHC_10819175	PATE2				2.568E-07					PATE2
V3SVHSHC_8967743	GAD2				2.651E-07					GAD2
V3SVHSHC_5732390	TINF2				2.783E-07					TINF2
V3SVHSHC_4911251	KIAA0586				2.848E-07					KIAA0586
V3SVHSHC_10150595	DTWD2				2.879E-07					DTWD2
V3SVHSHC_7653914	PLOD2				2.900E-07					PLOD2
V3SVHSHC_7387109	ASMTL				2.903E-07					ASMTL
V3SVHSHC_6978899	C2orf42				3.222E-07					C2orf42
V3SVHSHC_6485120	RBM8A				3.277E-07					RBM8A
V3SVHSHC_5761463	GTF2B				3.299E-07					GTF2B
V3SVHSHC_6716087	MTRNR2L1				3.425E-07					MTRNR2L 1
V3SVHSHC_10653878	LOC101928728				3.637E-07					LOC10192 8728
V3SVHSHC_9899630	TPP1				3.648E-07					TPP1
V3SVHSHC_9194981	LOC101927848				3.950E-07					LOC10192 7848
V3SVHSHC_8221745	PDP2				3.997E-07					PDP2
V3SVHSHC_5057045	PPARG				4.102E-07					PPARG
V3SVHSHC_7113869	IRAK3				4.155E-07					IRAK3
V3SVHSHC_5759021	ZNF233				4.392E-07					ZNF233
V3SVHSHC_8225408	SHKBP1				4.409E-07					SHKBP1
V3SVHSHC_8277779	EMR1				4.409E-07					EMR1
V3SVHSHC_7922798	TIMM10B				4.501E-07					TIMM10B
V3SVHSHC_8269826	INSIG1				4.540E-07					INSIG1
V3SVHSHC_10128518	MARCH4				4.836E-07					MARCH4
V3SVHSHC_8036252	AXIN1				5.069E-07					AXIN1
V3SVHSHC_7020941	USP3				5.346E-07					USP3
V3SVHSHC_6262205	E2F7				5.449E-07					E2F7
V3SVHSHC_4795025	OR10G4				5.776E-07					OR10G4

V3SVHSHC_8255603	RGS18				6.465E-07					RGS18
V3SVHSHC_6293027	C15orf54				6.580E-07					C15orf54
V3SVHSHC_5133308	SACM1L				6.802E-07					SACM1L
V3SVHSHC_4978274	LDHD				7.275E-07					LDHD
V3SVHSHC_5749814	SUPT20HL2				7.314E-07					SUPT20H L2
V3SVHSHC_6106148	BTC				7.438E-07					BTC
V3SVHSHC_6551384	CLINT1				8.036E-07					CLINT1
V3SVHSHC_9133535	ZFYVE20				8.057E-07					ZFYVE20
V3SVHSHC_9745124	TTC13				8.311E-07					TTC13
V3SVHSHC_4657118	SNAI1				8.518E-07					SNAI1
V3SVHSHC_7165250	TSSK2				8.601E-07					TSSK2
V3SVHSHC_8569532	MAFA				9.070E-07					MAFA
V3SVHSHC_5997380	CACNA1A				9.123E-07					CACNA1A
V3SVHSHC_10036217	LOC101930075				9.615E-07					LOC10193 0075
V3SVHSHC_5679194	SERPINE2				9.723E-07					SERPINE2
V3SVHSHC_9254414	GLRX2				9.912E-07					GLRX2
V3SVHSHC_8208479	SPSB2				1.011E-06					SPSB2
V3SVHSHC_8262335	TNFSF12				1.014E-06					TNFSF12
V3SVHSHC_7375163	ACY3				1.041E-06					ACY3
V3SVHSHC_7253822	RBP3				1.080E-06					RBP3
V3SVHSHC_7324178	CA6				1.112E-06					CA6
V3SVHSHC_9618239	AVPR1A				1.126E-06					AVPR1A
V3SVHSHC_4698368	STRIP2				1.155E-06					STRIP2
V3SVHSHC_6495911	TCP1				1.192E-06					TCP1
V3SVHSHC_5767403	COL1A2				1.234E-06					COL1A2
V3SVHSHC_5658140	GHR				1.252E-06					GHR
V3SVHSHC_9405455	C1QTNF3				1.289E-06					C1QTNF3
V3SVHSHC_9983054	NAPG				1.306E-06					NAPG
V3SVHSHC_7088162	OR10AD1				1.475E-06					OR10AD1
V3SVHSHC_4712393	PTPRCAP				1.556E-06					PTPRCAP
V3SVHSHC_7639592	FBXO48				1.593E-06					FBXO48
V3SVHSHC_5840828	BTBD11				1.628E-06					BTBD11
V3SVHSHC_8255141	CTNNB1				1.631E-06					CTNNB1
V3SVHSHC_7677707	KIAA1024				1.658E-06					KIAA1024
V3SVHSHC_7534916	NKG7				1.675E-06					NKG7
V3SVHSHC_7620452	TMUB1				1.728E-06					TMUB1
V3SVHSHC_7273259	ALDH4A1				1.740E-06					ALDH4A1
V3SVHSHC_7877159	BLVRB				1.866E-06					BLVRB
V3SVHSHC_8206928	HAGHL				1.873E-06					HAGHL
V3SVHSHC_7308536	NAAA				1.976E-06					NAAA
V3SVHSHC_9095189	PTPRR				2.000E-06					PTPRR
V3SVHSHC_7524752	OR2D2				2.075E-06					OR2D2
V3SVHSHC_5113673	ZNF132				2.084E-06					ZNF132
V3SVHSHC_6694109	ELF2				2.147E-06					ELF2
V3SVHSHC_4999031	SDF2L1				2.157E-06					SDF2L1
V3SVHSHC_4818488	C9orf139				2.248E-06					C9orf139
V3SVHSHC_9352391	RUNDC1				2.289E-06					RUNDC1
V3SVHSHC_6975830	GPR34				2.365E-06					GPR34
V3SVHSHC_10374764	GOLGA6L4				2.506E-06					GOLGA6L 4
V3SVHSHC_8904515	LRRC43				2.523E-06					LRRC43
V3SVHSHC_7448951	KAT6B				2.596E-06					KAT6B

V3SVHSHC_7154261	LOC100509091				2.641E-06					LOC10050 9091
V3SVHSHC_9091163	AXDND1				2.777E-06					AXDND1
V3SVHSHC_8566067	ACOXL				3.208E-06					ACOXL
V3SVHSHC_5217689	ARL6IP5				3.224E-06					ARL6IP5
V3SVHSHC_5406713	PDXDC1				3.380E-06					PDXDC1
V3SVHSHC_5037905	TRAPPC2L				3.651E-06					TRAPPC2 L
V3SVHSHC_9088556	RSAD1				3.653E-06					RSAD1
V3SVHSHC_10378460	ANKRD30A				3.817E-06					ANKRD30 A
V3SVHSHC_8839076	SLC35F5				3.882E-06					SLC35F5
V3SVHSHC_5847065	ZNRD1				3.893E-06					ZNRD1
V3SVHSHC_9653153	TAGLN3				3.908E-06					TAGLN3
V3SVHSHC_10422185	TRUB1				4.109E-06					TRUB1
V3SVHSHC_8642264	SLC24A3				4.250E-06					SLC24A3
V3SVHSHC_4825418	C7ORF34				4.515E-06					C7ORF34
V3SVHSHC_8101988	CES3				5.074E-06					CES3
V3SVHSHC_5825549	KLC2				5.081E-06					KLC2
V3SVHSHC_5986358	OR4N4				5.175E-06					OR4N4
V3SVHSHC_5472416	CCL28				5.851E-06					CCL28
V3SVHSHC_6807167	MRPS7				5.852E-06					MRPS7
V3SVHSHC_6246365	NR5A2				6.085E-06					NR5A2
V3SVHSHC_4732094	DTD1				6.143E-06					DTD1
V3SVHSHC_4723349	CACNA1I				6.184E-06					CACNA1I
V3SVHSHC_6785288	C7orf60				6.423E-06					C7orf60
V3SVHSHC_6584021	HRSP12				6.629E-06					HRSP12
V3SVHSHC_6114827	Ankle1				6.703E-06					Ankle1
V3SVHSHC_8607152	ZNF791				6.820E-06					ZNF791
V3SVHSHC_8759513	C6ORF25				6.960E-06					C6ORF25
V3SVHSHC_7904945	LOC101930551				7.205E-06					LOC10193 0551
V3SVHSHC_9907154	TNFRSF21				7.221E-06					TNFRSF2 1
V3SVHSHC_5890724	MS4A12				7.346E-06					MS4A12
V3SVHSHC_4954811	OR10G3				7.437E-06					OR10G3
V3SVHSHC_6208547	ZBTB8B				7.524E-06					ZBTB8B
V3SVHSHC_9604412	OVCA2				7.727E-06					OVCA2
V3SVHSHC_6266066	GTF2H1				8.186E-06					GTF2H1
V3SVHSHC_5794727	UBE2D2				8.478E-06					UBE2D2
V3SVHSHC_9758159	DGKI				8.514E-06					DGKI
V3SVHSHC_9761954	IDO1				8.586E-06					IDO1
V3SVHSHC_9817559	CEP85L				8.661E-06					CEP85L
V3SVHSHC_6387506	SLC22A2				8.970E-06					SLC22A2
V3SVHSHC_6364538	G6PC3				8.984E-06					G6PC3
V3SVHSHC_6556466	EML4				9.014E-06					EML4
V3SVHSHC_8134922	OR5K1				9.141E-06					OR5K1
V3SVHSHC_9956390	CCDC33				9.214E-06					CCDC33
V3SVHSHC_10293320	LOC285500				9.336E-06					LOC28550 0
V3SVHSHC_5009360	OR1F1				9.432E-06					OR1F1
V3SVHSHC_5632796	ZMPSTE24				9.943E-06					ZMPSTE2 4
V3SVHSHC_6269795	RSPH1				1.009E-05					RSPH1
V3SVHSHC_5742884	SERINC1				1.038E-05					SERINC1

V3SVHSHC_8271773	ZCCHC3				1.045E-05					ZCCHC3
V3SVHSHC_8263556	S1PR5				1.053E-05					S1PR5
V3SVHSHC_9169934	NCSTN				1.054E-05					NCSTN
V3SVHSHC_6312266	CENPO				1.141E-05					CENPO
V3SVHSHC_6374900	SNX1				1.159E-05					SNX1
V3SVHSHC_8674142	DRD1				1.168E-05					DRD1
V3SVHSHC_9818582	EHD3				1.207E-05					EHD3
V3SVHSHC_8383082	GRIN2A				1.250E-05					GRIN2A
V3SVHSHC_8866796	VANGL1				1.253E-05					VANGL1
V3SVHSHC_7158155	OFD1				1.265E-05					OFD1
V3SVHSHC_8896925	KRT1				1.327E-05					KRT1
V3SVHSHC_6495680	PLAGL1				1.339E-05					PLAGL1
V3SVHSHC_6125585	HFM1				1.381E-05					HFM1
V3SVHSHC_8709353	ZNF248				1.382E-05					ZNF248
V3SVHSHC_8170925	RPGR				1.391E-05					RPGR
V3SVHSHC_9677342	RARRES2				1.431E-05					RARRES2
V3SVHSHC_10615895	LYPD1				1.467E-05					LYPD1
V3SVHSHC_9533495	LMAN1L				1.520E-05					LMAN1L
V3SVHSHC_5415755	TTBK1				1.619E-05					TTBK1
V3SVHSHC_7242569	FAM209A				1.631E-05					FAM209A
V3SVHSHC_10063706	HDLBP				1.655E-05					HDLBP
V3SVHSHC_8979557	MAP3K1				1.672E-05					MAP3K1
V3SVHSHC_4734371	CARD9				1.693E-05					CARD9
V3SVHSHC_9057173	SNTB1				1.804E-05					SNTB1
V3SVHSHC_6650945	SOX18				1.860E-05					SOX18
V3SVHSHC_8630549	C12orf76				1.875E-05					C12orf76
V3SVHSHC_7581578	NXN				1.908E-05					NXN
V3SVHSHC_6087734	PGM2				1.933E-05					PGM2
V3SVHSHC_6200099	DNAH8				1.945E-05					DNAH8
V3SVHSHC_9833663	RND1				1.981E-05					RND1
V3SVHSHC_7537820	ATP7A				2.173E-05					ATP7A
V3SVHSHC_9493598	LMTK3				2.270E-05					LMTK3
V3SVHSHC_7659854	C15orf61				2.278E-05					C15orf61
V3SVHSHC_9622034	CCDC22				2.336E-05					CCDC22
V3SVHSHC_6080012	TRIB1				2.474E-05					TRIB1
V3SVHSHC_8835083	ANKRD54				2.766E-05					ANKRD54
V3SVHSHC_8752088	UBE2QL1				2.770E-05					UBE2QL1
V3SVHSHC_9837788	KIFAP3				2.840E-05					KIFAP3
V3SVHSHC_9191912	FILIP1L				2.861E-05					FILIP1L
V3SVHSHC_7199339	ABCF2				2.890E-05					ABCF2
V3SVHSHC_10412813	IGFL3				2.909E-05					IGFL3
V3SVHSHC_5450306	PPT2				2.933E-05					PPT2
V3SVHSHC_10655198	TPBGL				2.937E-05					TPBGL
V3SVHSHC_6811952	NDUFS5				3.050E-05					NDUFS5
V3SVHSHC_4689722	GSG1				3.108E-05					GSG1
V3SVHSHC_10521944	WRN				3.232E-05					WRN
V3SVHSHC_7556201	ZNF684				3.366E-05					ZNF684
V3SVHSHC_7300154	GMEB1				3.366E-05					GMEB1
V3SVHSHC_10402154	STAT5A				3.386E-05					STAT5A
V3SVHSHC_10292627	TENM2				3.425E-05					TENM2
V3SVHSHC_4989032	CACNA1B				3.432E-05					CACNA1B
V3SVHSHC_8618735	KCNV2				3.513E-05					KCNV2
V3SVHSHC_9162278	QPCT				3.741E-05					QPCT
V3SVHSHC_10289195	DUOX1				3.883E-05					DUOX1
V3SVHSHC_10781357	GPC1				3.926E-05					GPC1

V3SVHSHC_9525080	PDE6H				4.029E-05					PDE6H
V3SVHSHC_4755524	AKT1S1				4.128E-05					AKT1S1
V3SVHSHC_8341469	LOC100293704				4.325E-05					LOC10029 3704
V3SVHSHC_7665101	WFDC6				4.472E-05					WFDC6
V3SVHSHC_8571314	OSGEPL1				4.503E-05					OSGEPL1
V3SVHSHC_9637280	SKP2				4.535E-05					SKP2
V3SVHSHC_9321965	KPRP				4.718E-05					KPRP
V3SVHSHC_6425555	NDOR1				4.794E-05					NDOR1
V3SVHSHC_5356619	SRI				4.997E-05					SRI
V3SVHSHC_9878906	CAPN10				5.061E-05					CAPN10
V3SVHSHC_9021203	VRK3				5.467E-05					VRK3
V3SVHSHC_5473076	SLC25A2				5.668E-05					SLC25A2
V3SVHSHC_10428521	Aspg				5.847E-05					Aspg
V3SVHSHC_8999654	TSSC4				5.913E-05					TSSC4
V3SVHSHC_8183366	LSM12				5.916E-05					LSM12
V3SVHSHC_5526437	SCFD2				6.048E-05					SCFD2
V3SVHSHC_9715523	ZNF263				6.052E-05					ZNF263
V3SVHSHC_8488946	CCNG1				6.076E-05					CCNG1
V3SVHSHC_7444496	BMP10				6.083E-05					BMP10
V3SVHSHC_8447465	NSUN7				6.099E-05					NSUN7
V3SVHSHC_9732023	LOC100996318				6.349E-05					LOC10099 6318
V3SVHSHC_7668071	EML2				6.457E-05					EML2
V3SVHSHC_7503302	MMADHC				6.614E-05					MMADHC
V3SVHSHC_5389817	OR6M1				6.656E-05					OR6M1
V3SVHSHC_8612234	CTNNB1				6.660E-05					CTNNB1
V3SVHSHC_8285765	UBE3A				6.842E-05					UBE3A
V3SVHSHC_6697739	FGD6				6.859E-05					FGD6
V3SVHSHC_9962759	LAMA1				7.675E-05					LAMA1
V3SVHSHC_7156406	USB1				7.722E-05					USB1
V3SVHSHC_6446180	TMEM178A				7.785E-05					TMEM178 A
V3SVHSHC_10309457	CDADC1				7.886E-05					CDADC1
V3SVHSHC_7409945	MTMR7				8.160E-05					MTMR7
V3SVHSHC_9152114	NPC1				8.194E-05					NPC1
V3SVHSHC_6069254	MCC				8.623E-05					MCC
V3SVHSHC_7617218	CLEC4M				8.695E-05					CLEC4M
V3SVHSHC_6466937	DSCR3				9.018E-05					DSCR3
V3SVHSHC_10212041	THAP2				9.059E-05					THAP2
V3SVHSHC_6668699	OR12D3				9.226E-05					OR12D3
V3SVHSHC_8658335	VN1R5				9.444E-05					VN1R5
V3SVHSHC_5870330	LOC101929702				9.547E-05					LOC10192 9702
V3SVHSHC_9106145	ZPBP2				9.585E-05					ZPBP2
V3SVHSHC_8249498	SPRED1				9.652E-05					SPRED1
V3SVHSHC_10361432	TRERF1				9.662E-05					TRERF1
V3SVHSHC_6406613	AAED1				9.804E-05					AAED1
V3SVHSHC_7921181	SPATA2				9.804E-05					SPATA2
V3SVHSHC_10307609	TMEM45B				9.934E-05					TMEM45B
V3SVHSHC_7480598	PRSS57				1.006E-04					PRSS57
V3SVHSHC_6294116	DIABLO				1.012E-04					DIABLO
V3SVHSHC_6656093	STC1				1.013E-04					STC1
V3SVHSHC_10216595	TMEM253				1.016E-04					TMEM253
V3SVHSHC_6182576	GLB1L3				1.051E-04					GLB1L3

V3SVHSHC_9041597	SMPX				1.061E-04					SMPX
V3SVHSHC_4703879	TBC1D29				1.095E-04					TBC1D29
V3SVHSHC_9112481	ALPP				1.111E-04					ALPP
V3SVHSHC_9174158	SPRY4				1.117E-04					SPRY4
V3SVHSHC_9035987	CLEC17A				1.119E-04					CLEC17A
V3SVHSHC_6022988	C2orf27B				1.163E-04					C2orf27B
V3SVHSHC_8824358	LRRC30				1.164E-04					LRRC30
V3SVHSHC_7362029	AP3B1				1.233E-04					AP3B1
V3SVHSHC_7481753	OR2T8				1.238E-04					OR2T8
V3SVHSHC_6105092	RNF150				1.241E-04					RNF150
V3SVHSHC_9489044	KRT86				1.283E-04					KRT86
V3SVHSHC_7588508	CDC14A				1.342E-04					CDC14A
V3SVHSHC_4980815	SUSD5				1.351E-04					SUSD5
V3SVHSHC_5998931	Ankrd52				1.378E-04					Ankrd52
V3SVHSHC_4760441	LEMD2				1.408E-04					LEMD2
V3SVHSHC_9551612	ELMOD2				1.473E-04					ELMOD2
V3SVHSHC_7839242	C15orf57				1.629E-04					C15orf57
V3SVHSHC_4873928	REPIN1				1.648E-04					REPIN1
V3SVHSHC_4801394	FOXL2				1.723E-04					FOXL2
V3SVHSHC_10518380	TAF6L				1.760E-04					TAF6L
V3SVHSHC_4717574	GCSAML				1.839E-04					GCSAML
V3SVHSHC_6347246	NARFL				1.935E-04					NARFL
V3SVHSHC_6918839	TUBB2A				1.993E-04					TUBB2A
V3SVHSHC_10076642	EIF4ENIF1				2.006E-04					EIF4ENIF1
V3SVHSHC_6807596	OR4P4				2.084E-04					OR4P4
V3SVHSHC_6633653	C15orf53				2.195E-04					C15orf53
V3SVHSHC_9923357	GRM2				2.377E-04					GRM2
V3SVHSHC_6580061	CLDN1				2.482E-04					CLDN1
V3SVHSHC_5489411	MTMR6				2.568E-04					MTMR6
V3SVHSHC_4716683	CD40LG				2.701E-04					CD40LG
V3SVHSHC_7939991	SPON2				2.772E-04					SPON2
V3SVHSHC_6684737	MTUS2				2.785E-04					MTUS2
V3SVHSHC_8087699	RABGEF1				2.835E-04					RABGEF1
V3SVHSHC_5543927	CRISP1				2.912E-04					CRISP1
V3SVHSHC_10449773	CECR6				3.048E-04					CECR6
V3SVHSHC_4788029	PRAMEF10				3.076E-04					PRAMEF1<br>0
V3SVHSHC_9446012	YWHAG				3.196E-04					YWHAG
V3SVHSHC_8085422	TNFRSF1B				3.278E-04					TNFRSF1<br>B
V3SVHSHC_8172179	VPS52				3.307E-04					VPS52
V3SVHSHC_8071694	LRIG3				3.362E-04					LRIG3
V3SVHSHC_4992167	UNC79				3.449E-04					UNC79
V3SVHSHC_5499773	RHOD				3.513E-04					RHOD
V3SVHSHC_4858517	CDK3				3.634E-04					CDK3
V3SVHSHC_5053481	C12orf71				3.877E-04					C12orf71
V3SVHSHC_10739579	AQP5				3.898E-04					AQP5
V3SVHSHC_5510234	DPYSL3				3.959E-04					DPYSL3
V3SVHSHC_10693478	NGB				4.004E-04					NGB
V3SVHSHC_8664737	KIAA0513				4.458E-04					KIAA0513
V3SVHSHC_9552239	STAB1				4.606E-04					STAB1
V3SVHSHC_5789282	KRT6A				4.649E-04					KRT6A
V3SVHSHC_8357111	CIDEC				4.662E-04					CIDEC
V3SVHSHC_7270685	PSPH				4.723E-04					PSPH

V3SVHSHC_7823435	DHRS4				4.747E-04					DHRS4
V3SVHSHC_10606721	YPEL4				4.748E-04					YPEL4
V3SVHSHC_8787662	KRAS				4.908E-04					KRAS
V3SVHSHC_5345762	SMIM4				5.019E-04					SMIM4
V3SVHSHC_9306884	LGMN				5.158E-04					LGMN
V3SVHSHC_4896731	DEFB116				5.163E-04					DEFB116
V3SVHSHC_7671503	Zglp1				5.251E-04					Zglp1
V3SVHSHC_6086051	CLEC2A				5.429E-04					CLEC2A
V3SVHSHC_6582239	PKN1				5.462E-04					PKN1
V3SVHSHC_10721627	IL21R				5.496E-04					IL21R
V3SVHSHC_10632791	ZP1				5.624E-04					ZP1
V3SVHSHC_9353018	SDC2				5.714E-04					
V3SVHSHC_5413181	PDDC1				5.756E-04					
V3SVHSHC_7840430	KRBA2				5.783E-04					
V3SVHSHC_9483632	COPS8				6.024E-04					
V3SVHSHC_9503927	ZNF274				6.166E-04					
V3SVHSHC_6879173	GNG3				6.481E-04					
V3SVHSHC_5860298	DUSP11				6.583E-04					
V3SVHSHC_8016848	LOC100653137				6.627E-04					
V3SVHSHC_5675465	ZG16				6.736E-04					
V3SVHSHC_7182509	LRRC19				6.859E-04					
V3SVHSHC_8366318	SLC12A4				6.962E-04					
V3SVHSHC_7979261	Tmem201				6.990E-04					
V3SVHSHC_10224977	CCNC				7.031E-04					
V3SVHSHC_8920652	INSM1				7.290E-04					
V3SVHSHC_9848612	MSS51				7.505E-04					
V3SVHSHC_7812380	MANEAL				7.932E-04					
V3SVHSHC_5649593	PPIC				8.074E-04					
V3SVHSHC_9985760	OR2L13				8.182E-04					
V3SVHSHC_9727139	UGT2B10				8.217E-04					
V3SVHSHC_5930291	CRISP3				8.236E-04					
V3SVHSHC_5624447	STRN4				8.478E-04					
V3SVHSHC_6569963	FEM1B				8.638E-04					
V3SVHSHC_8703842	ZNF212				8.644E-04					
V3SVHSHC_5771990	MBD4				9.310E-04					
V3SVHSHC_9793172	GRIN3B				9.487E-04					
V3SVHSHC_8544056	CDH6				9.640E-04					
V3SVHSHC_4721105	LMNB1				1.025E-03					
V3SVHSHC_9413540	HLX				1.075E-03					
V3SVHSHC_9942365	FAM129C				1.080E-03					
V3SVHSHC_8332064	GTF2IRD1				1.096E-03					
V3SVHSHC_9710045	LOC101928805				1.146E-03					
V3SVHSHC_8279462	EXOSC10				1.193E-03					
V3SVHSHC_8651471	PIK3C2A				1.197E-03					
V3SVHSHC_7198778	SOX4				1.206E-03					
V3SVHSHC_6161720	DEFB103B				1.207E-03					
V3SVHSHC_5377442	POLB				1.248E-03					
V3SVHSHC_5105588	FOXD4L4				1.253E-03					
V3SVHSHC_4991177	C17orf70				1.256E-03					
V3SVHSHC_7255373	CSTA				1.257E-03					
V3SVHSHC_6575639	AMBP				1.261E-03					
V3SVHSHC_7173269	LOC101928879				1.270E-03					
V3SVHSHC_5119547	E2F5				1.271E-03					
V3SVHSHC_8673482	LANCL1				1.277E-03					
V3SVHSHC_6557093	ZNF174				1.279E-03					

V3SVHSHC_6921974	MAP3K2			1.305E-03						
V3SVHSHC_9828119	CALML3			1.314E-03						
V3SVHSHC_9061727	LOC100132004			1.349E-03						
V3SVHSHC_9627248	RAD51D			1.361E-03						
V3SVHSHC_6904022	FAM98B			1.367E-03						
V3SVHSHC_6344111	ATP8B2			1.372E-03						
V3SVHSHC_7450601	PLAU			1.379E-03						
V3SVHSHC_6544388	GABRQ			1.401E-03						
V3SVHSHC_4785653	C9ORF3			1.466E-03						
V3SVHSHC_10486106	PBX2			1.606E-03						
V3SVHSHC_8766608	CHRAC1			1.677E-03						
V3SVHSHC_6934745	Crybg3			1.683E-03						
V3SVHSHC_10678727	DPEP1			1.753E-03						
V3SVHSHC_4980089	IL7			1.755E-03						
V3SVHSHC_7775519	KLHL35			1.760E-03						
V3SVHSHC_6186173	AADACL2			1.777E-03						
V3SVHSHC_7737899	KIAA0226L			1.879E-03						
V3SVHSHC_6403412	TACR1			1.881E-03						
V3SVHSHC_7897355	EDEM3			1.886E-03						
V3SVHSHC_6807959	GATSL2			1.938E-03						
V3SVHSHC_8722322	C22orf42			2.027E-03						
V3SVHSHC_8483336	FAM72A			2.190E-03						
V3SVHSHC_10162442	NUMA1			2.194E-03						
V3SVHSHC_8591939	RASSF8			2.294E-03						
V3SVHSHC_7982198	CDR2			2.297E-03						
V3SVHSHC_8159309	CCND1			2.482E-03						
V3SVHSHC_9870359	ALG8			2.496E-03						
V3SVHSHC_5658206	MIXL1			2.509E-03						
V3SVHSHC_7394831	SLC22A3			2.539E-03						
V3SVHSHC_10027604	TRAF3IP2			2.647E-03						
V3SVHSHC_5764070	PQBP1			2.668E-03						
V3SVHSHC_10385423	BANK1			2.680E-03						
V3SVHSHC_9988136	APOBEC3A			2.757E-03						
V3SVHSHC_7909367	GINS1			2.816E-03						
V3SVHSHC_7216004	RPUSD3			2.828E-03						
V3SVHSHC_5591315	TCP11			2.931E-03						
V3SVHSHC_4985798	C7			3.090E-03						
V3SVHSHC_10287380	PTCH2			3.190E-03						
V3SVHSHC_10008629	IFNB1			3.282E-03						
V3SVHSHC_5227754	Ahnak2			3.300E-03						
V3SVHSHC_4984874	ZNF425			3.418E-03						
V3SVHSHC_5590985	ZNF862			3.438E-03						
V3SVHSHC_10766705	NKG7			3.444E-03						
V3SVHSHC_5558546	CH25H			3.519E-03						
V3SVHSHC_5436941	CATSPER2			3.528E-03						
V3SVHSHC_6376187	OR6B1			3.532E-03						
V3SVHSHC_9725159	RIMBP3			3.534E-03						
V3SVHSHC_10161518	RENBP			3.561E-03						
V3SVHSHC_5402984	RGS17			3.615E-03						
V3SVHSHC_6986489	DCLRE1A			3.632E-03						
V3SVHSHC_8747303	FAM167A			3.638E-03						
V3SVHSHC_6665993	BTBD6			3.805E-03						
V3SVHSHC_7089383	NR2C1			3.832E-03						
V3SVHSHC_4860167	SIN3B			3.859E-03						
V3SVHSHC_8164853	ZFAND2B			3.882E-03						

V3SVHSHC_6504590	ZNF836				3.944E-03					
V3SVHSHC_10191086	AGPAT1				4.044E-03					
V3SVHSHC_10132346	CREG1				4.200E-03					
V3SVHSHC_6931610	SOCS6				4.321E-03					
V3SVHSHC_6241613	AGPS				4.348E-03					
V3SVHSHC_7430471	SMARCA2				4.433E-03					
V3SVHSHC_9708527	TM4SF4				4.446E-03					
V3SVHSHC_7296359	OLFM2				4.453E-03					
V3SVHSHC_5723876	ABI2				4.454E-03					
V3SVHSHC_7258673	TRIM4				4.569E-03					
V3SVHSHC_7790798	VCL				4.572E-03					
V3SVHSHC_7456310	ENKUR				4.771E-03					
V3SVHSHC_6065360	FAU				4.829E-03					
V3SVHSHC_6274217	DLX5				4.835E-03					
V3SVHSHC_10518446	DTD1				4.927E-03					
V3SVHSHC_4773707	GCDH				4.936E-03					
V3SVHSHC_7044404	HOXC5				4.969E-03					
V3SVHSHC_9047636	UBXN2B				4.972E-03					
V3SVHSHC_9584975	CHRM5				5.050E-03					
V3SVHSHC_4844657	SNX6				5.064E-03					
V3SVHSHC_8432021	ARSI				5.180E-03					
V3SVHSHC_4695002	AGXT2				5.321E-03					
V3SVHSHC_8678597	RNASEH2B				5.469E-03					
V3SVHSHC_5282402	SLC9C1				5.499E-03					
V3SVHSHC_7750967	CCT4				5.647E-03					
V3SVHSHC_7811456	GTF2IRD2B				5.767E-03					
V3SVHSHC_9117101	BRMS1				5.821E-03					
V3SVHSHC_6575177	CHMP5				5.937E-03					
V3SVHSHC_5083709	CCND2				5.948E-03					
V3SVHSHC_9085685	DUS3L				5.986E-03					
V3SVHSHC_4878086	PCDHGA9				6.008E-03					
V3SVHSHC_8455715	OR2B2				6.029E-03					
V3SVHSHC_5288375	DLG5				6.120E-03					
V3SVHSHC_7789181	FEZF1				6.134E-03					
V3SVHSHC_9048923	SLC25A10				6.161E-03					
V3SVHSHC_5981276	TRIM33				6.347E-03					
V3SVHSHC_8083013	LALBA				6.377E-03					
V3SVHSHC_4842215	RPS6KB1				6.491E-03					
V3SVHSHC_7881647	LILRB5				6.545E-03					
V3SVHSHC_7945898	CBR3				6.673E-03					
V3SVHSHC_10479176	CD3D				6.812E-03					
V3SVHSHC_7259993	DDX60L				6.813E-03					
V3SVHSHC_9310283	RNF38				7.007E-03					
V3SVHSHC_4904222	DAAM1				7.143E-03					
V3SVHSHC_5838386	PHF2				7.434E-03					
V3SVHSHC_8721695	COX14				7.520E-03					
V3SVHSHC_9506072	POU4F3				7.526E-03					
V3SVHSHC_4716485	ANKMY1				7.624E-03					
V3SVHSHC_10524584	ZNF445				7.642E-03					
V3SVHSHC_10766078	PACS1				7.663E-03					
V3SVHSHC_4657415	FOXF2				7.714E-03					
V3SVHSHC_9492575	TRIM52				7.719E-03					
V3SVHSHC_7184291	DNAJB7				7.761E-03					
V3SVHSHC_5563595	ABCD2				7.868E-03					
V3SVHSHC_10377635	TMPRSS13				7.975E-03					

V3SVHSHC_8888840	LCN2				7.993E-03					
V3SVHSHC_6963983	IPMK				8.081E-03					
V3SVHSHC_7753871	GPR146				8.132E-03					
V3SVHSHC_8187260	PHKG1				8.254E-03					
V3SVHSHC_7265603	CYP4V2				8.307E-03					
V3SVHSHC_9915338	NEUROD6				8.344E-03					
V3SVHSHC_10624673	SNX1				8.387E-03					
V3SVHSHC_6087470	FAM96A				8.422E-03					
V3SVHSHC_8010809	CFP				8.575E-03					
V3SVHSHC_8114198	TIMM22				8.771E-03					
V3SVHSHC_10681037	AKAP2				8.835E-03					
V3SVHSHC_6224915	ZNF445				8.938E-03					
V3SVHSHC_6966623	REM1				9.044E-03					
V3SVHSHC_8367143	PCDP1				9.129E-03					
V3SVHSHC_5630387	C19orf33				9.146E-03					
V3SVHSHC_8955830	GLTSCR1L				9.186E-03					
V3SVHSHC_7341965	SAP30				9.211E-03					
V3SVHSHC_6547490	NDUFAB1				9.273E-03					
V3SVHSHC_9777695	LOC402269				9.388E-03					
V3SVHSHC_10063079	ADAMTSL1				9.551E-03					
V3SVHSHC_6931313	GGT2				9.601E-03					
V3SVHSHC_5615306	RASIP1				9.748E-03					
V3SVHSHC_5482052	SLC39A10				9.840E-03					
V3SVHSHC_8922830	C7orf71				9.907E-03					
V3SVHSHC_10283651	SMOC1				9.922E-03					
V3SVHSHC_10758686	TMEM35				9.943E-03					
V3SVHSHC_10144193	KCNJ9				1.035E-02					
V3SVHSHC_10042124	SH3BP5				1.052E-02					
V3SVHSHC_9781028	GNG4				1.054E-02					
V3SVHSHC_10415156	SYT1				1.092E-02					
V3SVHSHC_5084402	ZNF391				1.120E-02					
V3SVHSHC_8183993	CIITA				1.165E-02					
V3SVHSHC_10529039	SHISA2				1.172E-02					
V3SVHSHC_8993252	PCOLCE				1.175E-02					
V3SVHSHC_5662892	PCDHGA1				1.177E-02					
V3SVHSHC_9495941	LRRN2				1.187E-02					
V3SVHSHC_7260884	DAB2IP				1.226E-02					
V3SVHSHC_5882309	STXBP3				1.246E-02					
V3SVHSHC_7395953	TLR10				1.274E-02					
V3SVHSHC_6371501	ST6GAL1				1.308E-02					
V3SVHSHC_7784000	ANKAR				1.312E-02					
V3SVHSHC_7866368	AIFM3				1.317E-02					
V3SVHSHC_7895276	VPS45				1.320E-02					
V3SVHSHC_8775980	TMEM259				1.339E-02					
V3SVHSHC_5937683	TXNL4A				1.340E-02					
V3SVHSHC_7515413	CASP2				1.363E-02					
V3SVHSHC_10630052	LARGE				1.372E-02					
V3SVHSHC_8977742	ITGB1BP2				1.405E-02					
V3SVHSHC_8110634	RWDD4				1.433E-02					
V3SVHSHC_8425058	PHF14				1.439E-02					
V3SVHSHC_7885442	RHNO1				1.451E-02					
V3SVHSHC_8950682	IL17A				1.451E-02					
V3SVHSHC_5876369	CR2				1.505E-02					
V3SVHSHC_7645697	KPNA4				1.529E-02					
V3SVHSHC_10429511	LOC101930125				1.538E-02					

V3SVHSHC_6894551	HHIPL2				1.560E-02						
V3SVHSHC_9575702	CDH7				1.581E-02						
V3SVHSHC_8621837	CCDC102A				1.601E-02						
V3SVHSHC_6524555	AIP				1.654E-02						
V3SVHSHC_5652563	GNB5				1.677E-02						
V3SVHSHC_7514984	LHX1				1.683E-02						
V3SVHSHC_10755056	RYK				1.690E-02						
V3SVHSHC_5709554	RBM5				1.752E-02						
V3SVHSHC_8949098	LPL				1.763E-02						
V3SVHSHC_5352494	FAM171A1				1.781E-02						
V3SVHSHC_9271277	TRPM7				1.783E-02						
V3SVHSHC_8538611	MLPH				1.816E-02						
V3SVHSHC_8028299	NAT14				1.825E-02						
V3SVHSHC_7601774	NTN4				1.874E-02						
V3SVHSHC_7774925	ZNF609				1.890E-02						
V3SVHSHC_9386150	DIAPH2				1.906E-02						
V3SVHSHC_6385790	ST3GAL3				1.908E-02						
V3SVHSHC_9487823	ADNP				2.033E-02						
V3SVHSHC_9260981	LOC101927594				2.056E-02						
V3SVHSHC_9632858	PTTG2				2.068E-02						
V3SVHSHC_6553100	RSC1A1				2.155E-02						
V3SVHSHC_10292231	Ucma				2.177E-02						
V3SVHSHC_6079781	C20orf62				2.183E-02						
V3SVHSHC_7549139	FST				2.226E-02						
V3SVHSHC_10233986	GLYR1				2.227E-02						
V3SVHSHC_9153071	TBC1D10A				2.239E-02						
V3SVHSHC_10624079	RNASE8				2.262E-02						
V3SVHSHC_6655895	ATP5L				2.269E-02						
V3SVHSHC_8427731	SERPINH1				2.272E-02						
V3SVHSHC_9956060	STK4				2.298E-02						
V3SVHSHC_10075949	Fam154b				2.302E-02						
V3SVHSHC_8293289	SFTPD				2.382E-02						
V3SVHSHC_4903397	DMP1				2.425E-02						
V3SVHSHC_8945798	NDST2				2.442E-02						
V3SVHSHC_8958569	FAM185A				2.493E-02						
V3SVHSHC_9821255	FAM71E2				2.541E-02						
V3SVHSHC_8645432	POTEB				2.545E-02						
V3SVHSHC_7975994	DMBT1				2.571E-02						
V3SVHSHC_6082652	MAMDC4				2.610E-02						
V3SVHSHC_10183562	ZFY				2.641E-02						
V3SVHSHC_5846273	EIF2AK3				2.677E-02						
V3SVHSHC_10242698	NEDD4				2.698E-02						
V3SVHSHC_6820103	C5orf60				2.746E-02						
V3SVHSHC_7379750	SEPT14				2.752E-02						
V3SVHSHC_10016417	NRD1				2.755E-02						
V3SVHSHC_9503432	KDM5C				2.783E-02						
V3SVHSHC_5110967	NMRAL1				2.823E-02						
V3SVHSHC_6900656	ITGB1BP1				2.853E-02						
V3SVHSHC_9392453	CTGF				2.865E-02						
V3SVHSHC_8997014	OBFC1				2.903E-02						
V3SVHSHC_9027143	FEZF2				2.928E-02						
V3SVHSHC_9111821	SIGMAR1				2.997E-02						
V3SVHSHC_4995302	SLC10A4				3.034E-02						
V3SVHSHC_5111198	ITM2B				3.049E-02						
V3SVHSHC_8159243	CDC42EP3				3.115E-02						

V3SVHSHC_8972099	FAM171A2				3.129E-02					
V3SVHSHC_9299030	F12				3.200E-02					
V3SVHSHC_7932467	NDRG3				3.250E-02					
V3SVHSHC_10114526	WNT6				3.269E-02					
V3SVHSHC_9975266	C6orf183				3.381E-02					
V3SVHSHC_9956753	UQCC2				3.384E-02					
V3SVHSHC_9470465	OR51B4				3.390E-02					
V3SVHSHC_9339455	ATP5G3				3.411E-02					
V3SVHSHC_5080409	TNFAIP2				3.429E-02					
V3SVHSHC_9198809	LOC100287294				3.439E-02					
V3SVHSHC_4747769	PALM2				3.489E-02					
V3SVHSHC_5825879	ADAMTSL1				3.548E-02					
V3SVHSHC_4947947	COX7B				3.584E-02					
V3SVHSHC_5231483	PBX4				3.652E-02					
V3SVHSHC_9431228	MMGT1				3.654E-02					
V3SVHSHC_6488717	CYR61				3.665E-02					
V3SVHSHC_9871118	TEX264				3.672E-02					
V3SVHSHC_9712322	PADI3				3.686E-02					
V3SVHSHC_10077665	PELI2				3.708E-02					
V3SVHSHC_10736114	VWA5A				3.764E-02					
V3SVHSHC_5992760	CDC6				3.776E-02					
V3SVHSHC_6637283	CCDC158				3.824E-02					
V3SVHSHC_9654242	TLE6				3.857E-02					
V3SVHSHC_10464194	LMO4				3.906E-02					
V3SVHSHC_8491652	ZEB2				3.934E-02					
V3SVHSHC_10653944	ACTN3				3.951E-02					
V3SVHSHC_10216331	ZNF280D				3.972E-02					
V3SVHSHC_7007246	TACC1				3.986E-02					
V3SVHSHC_4693550	Krtdap				4.042E-02					
V3SVHSHC_8167856	TCERG1L				4.063E-02					
V3SVHSHC_7246958	DSCR3				4.079E-02					
V3SVHSHC_5839013	ADH1B				4.130E-02					
V3SVHSHC_9602366	PINK1				4.137E-02					
V3SVHSHC_5010911	ART1				4.168E-02					
V3SVHSHC_10559333	CHN2				4.196E-02					
V3SVHSHC_7607252	NEK4				4.213E-02					
V3SVHSHC_9532604	ACO1				4.220E-02					
V3SVHSHC_9634574	TRIOBP				4.252E-02					
V3SVHSHC_6033911	SMIM6				4.266E-02					
V3SVHSHC_5831060	TLR9				4.342E-02					
V3SVHSHC_9750866	SIX1				4.409E-02					
V3SVHSHC_10547750	BRSK2				4.431E-02					
V3SVHSHC_10407170	ZNF136				4.518E-02					
V3SVHSHC_10238540	OR2F1				4.747E-02					
V3SVHSHC_4967087	STK40				4.783E-02					
V3SVHSHC_8049881	RAMP3				4.859E-02					
V3SVHSHC_5209241	ARHGDIG				4.950E-02					
V3SVHSHC_4679723	DEFB132				4.995E-02					
V3SVHSHC_5137202	KAT5				5.032E-02					
V3SVHSHC_9410042	ECI1				5.123E-02					
V3SVHSHC_5732786	ATP6AP2				5.145E-02					
V3SVHSHC_10591343	ALDH1A2				5.231E-02					
V3SVHSHC_10593983	PTGES				5.298E-02					
V3SVHSHC_9073970	RPAP2				5.412E-02					
V3SVHSHC_7751165	CPAMD8				5.442E-02					

V3SVHSHC_7783109	PDE1B				5.476E-02					
V3SVHSHC_7621211	FAM43B				5.491E-02					
V3SVHSHC_8963354	NME1-NME2				5.530E-02					
V3SVHSHC_8918540	USP44				5.536E-02					
V3SVHSHC_8935271	CAMK2A				5.540E-02					
V3SVHSHC_10247813	CMTM5				5.768E-02					
V3SVHSHC_9157295	TGIF2				5.776E-02					
V3SVHSHC_5514755	NDST2				5.882E-02					
V3SVHSHC_8634113	FAM46D				5.907E-02					
V3SVHSHC_10129574	RPL7L1				5.912E-02					
V3SVHSHC_9256625	CLEC11A				5.928E-02					
V3SVHSHC_4697312	KRTAP20-2				6.082E-02					
V3SVHSHC_6975269	LEF1				6.122E-02					
V3SVHSHC_8098028	CORO2B				6.198E-02					
V3SVHSHC_6270323	TRIM71				6.281E-02					
V3SVHSHC_8540855	PLCB2				6.409E-02					
V3SVHSHC_6179078	TBL3				6.523E-02					
V3SVHSHC_9662492	DHX29				6.540E-02					
V3SVHSHC_10311404	ZNF670				6.577E-02					
V3SVHSHC_6447500	OR52M1				6.684E-02					
V3SVHSHC_9919034	CARS				6.695E-02					
V3SVHSHC_7342526	CSNK1E				6.776E-02					
V3SVHSHC_7075193	SEMA4F				6.803E-02					
V3SVHSHC_6936032	PARP6				6.840E-02					
V3SVHSHC_8245373	C11orf57				6.976E-02					
V3SVHSHC_6433079	C1orf137				7.001E-02					
V3SVHSHC_7471853	OR4S1				7.044E-02					
V3SVHSHC_9501287	TTLL2				7.100E-02					
V3SVHSHC_5413049	PSMA3				7.127E-02					
V3SVHSHC_5875082	ZHX1				7.127E-02					
V3SVHSHC_8999786	CWF19L2				7.129E-02					
V3SVHSHC_7541648	CD96				7.220E-02					
V3SVHSHC_7540526	LOC101927910				7.239E-02					
V3SVHSHC_4951280	QPRT				7.273E-02					
V3SVHSHC_6443705	ACN9				7.346E-02					
V3SVHSHC_5905376	CCL20				7.390E-02					
V3SVHSHC_8115947	Crybg3				7.478E-02					
V3SVHSHC_5359853	VWA9				7.512E-02					
V3SVHSHC_6415358	PRPF38A				7.622E-02					
V3SVHSHC_5205479	RLN3				7.638E-02					
V3SVHSHC_7839473	KRT6B				7.741E-02					
V3SVHSHC_9268505	CSDE1				7.752E-02					
V3SVHSHC_7491554	PDCD2L				7.774E-02					
V3SVHSHC_10130168	GATA4				7.784E-02					
V3SVHSHC_9323054	SMCO3				7.831E-02					
V3SVHSHC_6277187	MYC				7.845E-02					
V3SVHSHC_7263161	WASL				7.934E-02					
V3SVHSHC_4886105	AGO2				7.976E-02					
V3SVHSHC_10467296	NDUFS3				8.036E-02					
V3SVHSHC_10781588	BTNL2				8.112E-02					
V3SVHSHC_9998465	Mettl14				8.170E-02					
V3SVHSHC_6972398	TMSB4Y				8.369E-02					
V3SVHSHC_5503700	MYH9				8.370E-02					
V3SVHSHC_10276523	DSTN				8.371E-02					
V3SVHSHC_10834850	SLC25A52				8.484E-02					

V3SVHSHC_7692821	LARP4				8.520E-02					
V3SVHSHC_7114529	H3F3B				8.529E-02					
V3SVHSHC_4759385	PRKD3				8.549E-02					
V3SVHSHC_10141685	GDNF				8.602E-02					
V3SVHSHC_4792187	PITPNB				8.683E-02					
V3SVHSHC_6256232	PAK6				8.697E-02					
V3SVHSHC_8252963	USP9Y				8.715E-02					
V3SVHSHC_4823042	KCNV2				8.733E-02					
V3SVHSHC_6294149	ZNF704				8.803E-02					
V3SVHSHC_4955042	TMEM106C				8.819E-02					
V3SVHSHC_8817593	SCRN2				8.822E-02					
V3SVHSHC_6305402	UBE4B				8.847E-02					
V3SVHSHC_9641372	ADH6				8.934E-02					
V3SVHSHC_9447035	BAG5				8.945E-02					
V3SVHSHC_4875050	LYPD2				8.965E-02					
V3SVHSHC_7842806	ARID3B				8.966E-02					
V3SVHSHC_10389647	TRAPPC9				9.012E-02					
V3SVHSHC_6566762	PAX3				9.022E-02					
V3SVHSHC_7794593	DCSTAMP				9.251E-02					
V3SVHSHC_5228513	TUBB2B				9.429E-02					
V3SVHSHC_6978239	RPL35				9.446E-02					
V3SVHSHC_7372853	F3				9.559E-02					
V3SVHSHC_6003551	RAPSN				9.624E-02					
V3SVHSHC_4728134	POLR3A				9.746E-02					
V3SVHSHC_6674771	CSNK1D				9.816E-02					
V3SVHSHC_6538910	PRDM1				9.873E-02					
V3SVHSHC_5907224	GCM2				9.988E-02					
V3SVHSHC_5859704	C12orf73				1.013E-01					
V3SVHSHC_10370441	LOC101929536				1.025E-01					
V3SVHSHC_10256030	AGXT				1.031E-01					
V3SVHSHC_6967910	TIFAB				1.042E-01					
V3SVHSHC_7487627	RPGR				1.045E-01					
V3SVHSHC_6171686	KPNA7				1.046E-01					
V3SVHSHC_9783635	CXorf30				1.049E-01					
V3SVHSHC_9517391	NDNL2				1.050E-01					
V3SVHSHC_5793176	CLCN6				1.053E-01					
V3SVHSHC_6886070	AFF4				1.053E-01					
V3SVHSHC_4976756	MIER3				1.062E-01					
V3SVHSHC_7786706	KRTAP29-1				1.064E-01					
V3SVHSHC_6409748	RRH				1.073E-01					
V3SVHSHC_7979921	SEPHS1				1.097E-01					
V3SVHSHC_10651667	BMX				1.106E-01					
V3SVHSHC_7376153	TMEM131				1.140E-01					
V3SVHSHC_7518977	TROVE2				1.170E-01					
V3SVHSHC_6989690	MKNK2				1.175E-01					
V3SVHSHC_4980419	TXNRD2				1.180E-01					
V3SVHSHC_6561647	IFITM3				1.182E-01					
V3SVHSHC_5959166	KIF20B				1.182E-01					
V3SVHSHC_7296062	SLC37A1				1.187E-01					
V3SVHSHC_5966723	TMPRSS11D				1.205E-01					
V3SVHSHC_6566564	EPB41L2				1.209E-01					
V3SVHSHC_7756082	VMO1				1.215E-01					
V3SVHSHC_8929694	DLL1				1.221E-01					
V3SVHSHC_6089153	Tomm20l				1.237E-01					
V3SVHSHC_5598047	ATAD5				1.237E-01					

V3SVHSHC_5238017	PDE12				1.258E-01					
V3SVHSHC_5158817	SNX5				1.275E-01					
V3SVHSHC_10804259	USP3				1.286E-01					
V3SVHSHC_7639526	SPACA4				1.304E-01					
V3SVHSHC_5127500	ENTPD1				1.305E-01					
V3SVHSHC_10737665	ENTHD1				1.318E-01					
V3SVHSHC_5582999	MAGEA9				1.319E-01					
V3SVHSHC_7335926	LNX1				1.340E-01					
V3SVHSHC_5685695	COL4A6				1.348E-01					
V3SVHSHC_9853364	VEGFB				1.360E-01					
V3SVHSHC_9576956	AKR1C1				1.360E-01					
V3SVHSHC_9933587	SEC24A				1.369E-01					
V3SVHSHC_8156306	KCNIP3				1.376E-01					
V3SVHSHC_8896793	TSSK4				1.378E-01					
V3SVHSHC_10706942	TGFBR3L				1.381E-01					
V3SVHSHC_6570425	ZNF8				1.410E-01					
V3SVHSHC_9943883	KPNA1				1.413E-01					
V3SVHSHC_5329592	CCDC85A				1.414E-01					
V3SVHSHC_6106247	COTL1				1.415E-01					
V3SVHSHC_7527095	CACNA1E				1.424E-01					
V3SVHSHC_10107497	ZNF623				1.454E-01					
V3SVHSHC_5067242	JUN				1.464E-01					
V3SVHSHC_7868117	GATAD2B				1.476E-01					
V3SVHSHC_9611210	LRRC10B				1.490E-01					
V3SVHSHC_8366021	GOLGA6L1				1.494E-01					
V3SVHSHC_9488978	C12orf55				1.500E-01					
V3SVHSHC_9499043	KRTAP10-9				1.507E-01					
V3SVHSHC_5979197	LOC100293704				1.524E-01					
V3SVHSHC_7290089	MYH14				1.532E-01					
V3SVHSHC_7671998	EPHA5				1.534E-01					
V3SVHSHC_10651007	AOC1				1.563E-01					
V3SVHSHC_6218810	ADAM7				1.569E-01					
V3SVHSHC_5170796	OR2V1				1.577E-01					
V3SVHSHC_6947285	LCE3D				1.578E-01					
V3SVHSHC_6873695	OR10A7				1.596E-01					
V3SVHSHC_9545672	CCDC150				1.597E-01					
V3SVHSHC_6765323	ASIC2				1.600E-01					
V3SVHSHC_6059090	AQP8				1.604E-01					
V3SVHSHC_9057371	LPAR1				1.618E-01					
V3SVHSHC_10144259	ATG2A				1.618E-01					
V3SVHSHC_6611939	POLR2F				1.624E-01					
V3SVHSHC_6377507	OR52W1				1.624E-01					
V3SVHSHC_7557356	RMND5B				1.648E-01					
V3SVHSHC_10100039	DHX40				1.655E-01					
V3SVHSHC_6744896	AKAP10				1.657E-01					
V3SVHSHC_4647944	RGS22				1.659E-01					
V3SVHSHC_5709686	ART5				1.676E-01					
V3SVHSHC_8956589	MOB2				1.682E-01					
V3SVHSHC_7960187	CDH15				1.683E-01					
V3SVHSHC_8833070	CEP162				1.697E-01					
V3SVHSHC_6681404	LOC101927989				1.699E-01					
V3SVHSHC_7536896	SIPA1L2				1.700E-01					
V3SVHSHC_8899400	NEFM				1.701E-01					
V3SVHSHC_5063381	C1orf174				1.714E-01					
V3SVHSHC_7087139	CSNK1D				1.718E-01					

V3SVHSHC_7211681	CCDC173				1.722E-01						
V3SVHSHC_5510168	KIAA0895L				1.730E-01						
V3SVHSHC_7976984	LOC100996906				1.731E-01						
V3SVHSHC_9371993	SGCB				1.733E-01						
V3SVHSHC_7264514	KLF12				1.739E-01						
V3SVHSHC_9268109	ZNF669				1.739E-01						
V3SVHSHC_5470205	SAMD14				1.749E-01						
V3SVHSHC_9233690	TMEM176B				1.787E-01						
V3SVHSHC_6752189	NDOR1				1.809E-01						
V3SVHSHC_4650254	SLC29A1				1.844E-01						
V3SVHSHC_6358334	RBM24				1.844E-01						
V3SVHSHC_8305400	OTUD7B				1.849E-01						
V3SVHSHC_4843370	KRTAP4-11				1.853E-01						
V3SVHSHC_6934580	MIER3				1.853E-01						
V3SVHSHC_5975369	FTH1				1.877E-01						
V3SVHSHC_5161622	LAMB1				1.887E-01						
V3SVHSHC_10314077	SLC12A7				1.888E-01						
V3SVHSHC_8448257	OGDH				1.908E-01						
V3SVHSHC_10035524	TRIM24				1.931E-01						
V3SVHSHC_8508053	LRRC42				1.944E-01						
V3SVHSHC_7921511	GYG2				1.950E-01						
V3SVHSHC_5232902	TRIM55				1.975E-01						
V3SVHSHC_8125583	COPZ2				1.975E-01						
V3SVHSHC_7799675	TFF3				1.986E-01						
V3SVHSHC_9370508	MYO15A				2.013E-01						
V3SVHSHC_7746512	CXorf49				2.025E-01						
V3SVHSHC_7928474	PKNOX1				2.037E-01						
V3SVHSHC_6622136	MSANTD3				2.037E-01						
V3SVHSHC_8648039	ELK4				2.058E-01						
V3SVHSHC_7714634	GZMB				2.068E-01						
V3SVHSHC_6236861	SCGB2A1				2.071E-01						
V3SVHSHC_6777467	CXorf49				2.091E-01						
V3SVHSHC_10704038	PDE5A				2.096E-01						
V3SVHSHC_10645496	BPTF				2.105E-01						
V3SVHSHC_6313883	ITGB1BP2				2.108E-01						
V3SVHSHC_4742720	MAP3K15				2.112E-01						
V3SVHSHC_9581543	LOC100996646				2.129E-01						
V3SVHSHC_6129743	LAMC2				2.132E-01						
V3SVHSHC_10643318	RSRC1				2.152E-01						
V3SVHSHC_10753208	PIGL				2.158E-01						
V3SVHSHC_7087535	SPAST				2.224E-01						
V3SVHSHC_5137499	IRX6				2.246E-01						
V3SVHSHC_5900162	PSAT1				2.255E-01						
V3SVHSHC_4858814	ATG16L1				2.258E-01						
V3SVHSHC_4981409	SDS				2.261E-01						
V3SVHSHC_9644771	ZNF536				2.265E-01						
V3SVHSHC_8159969	GPR63				2.272E-01						
V3SVHSHC_10716215	HIGD1C				2.277E-01						
V3SVHSHC_10199765	DTX3				2.294E-01						
V3SVHSHC_7063247	COPB2				2.295E-01						
V3SVHSHC_4867460	PLK1				2.297E-01						
V3SVHSHC_6410573	FNTB				2.309E-01						
V3SVHSHC_6516470	SULT6B1				2.310E-01						
V3SVHSHC_8484821	SIPA1L3				2.319E-01						
V3SVHSHC_10268306	PPP2R1A				2.334E-01						

V3SVHSHC_7655630	POLR1A				2.361E-01					
V3SVHSHC_10391561	SCAND3				2.364E-01					
V3SVHSHC_10752317	OR13C2				2.367E-01					
V3SVHSHC_10504619	PPIB				2.379E-01					
V3SVHSHC_5807663	RALGDS				2.384E-01					
V3SVHSHC_9939692	GMDS				2.411E-01					
V3SVHSHC_5335169	ARSD				2.442E-01					
V3SVHSHC_7814492	PIAS3				2.451E-01					
V3SVHSHC_7055558	NSUN3				2.460E-01					
V3SVHSHC_8907881	NGFRAP1				2.469E-01					
V3SVHSHC_7973585	ACTB				2.470E-01					
V3SVHSHC_4739948	GUCD1				2.476E-01					
V3SVHSHC_10540985	CASKIN1				2.485E-01					
V3SVHSHC_10594313	SKIL				2.508E-01					
V3SVHSHC_6909236	C21ORF33				2.520E-01					
V3SVHSHC_10737005	TGM6				2.521E-01					
V3SVHSHC_7752122	TRAP1				2.522E-01					
V3SVHSHC_8943389	MESP2				2.536E-01					
V3SVHSHC_7907453	NPY4R				2.544E-01					
V3SVHSHC_5089682	YAP1				2.549E-01					
V3SVHSHC_4986392	HMGCS1				2.579E-01					
V3SVHSHC_4716716	SRSF12				2.595E-01					
V3SVHSHC_5058101	SLC32A1				2.599E-01					
V3SVHSHC_8067041	ASB2				2.607E-01					
V3SVHSHC_6226598	ATP5E				2.641E-01					
V3SVHSHC_6503303	KCNT1				2.648E-01					
V3SVHSHC_10603058	TRAT1				2.656E-01					
V3SVHSHC_10168019	GPIHBP1				2.663E-01					
V3SVHSHC_7721036	DPH6				2.687E-01					
V3SVHSHC_5737439	AADAC				2.695E-01					
V3SVHSHC_4799678	GJD2				2.717E-01					
V3SVHSHC_8123966	KRTAP10-7				2.768E-01					
V3SVHSHC_6624644	CDR1				2.778E-01					
V3SVHSHC_9299261	CLPP				2.797E-01					
V3SVHSHC_10487459	PLK1				2.799E-01					
V3SVHSHC_5875412	MAGI2				2.806E-01					
V3SVHSHC_7226465	PI3				2.830E-01					
V3SVHSHC_10780433	HVCN1				2.848E-01					
V3SVHSHC_9177491	GTF2H2C				2.854E-01					
V3SVHSHC_8161322	ABCG4				2.859E-01					
V3SVHSHC_9651536	RASIP1				2.864E-01					
V3SVHSHC_10748753	TRMT2A				2.868E-01					
V3SVHSHC_7652198	CELF6				2.870E-01					
V3SVHSHC_6784100	ESCO2				2.876E-01					
V3SVHSHC_5151920	GLI2				2.877E-01					
V3SVHSHC_7791887	Prss58				2.889E-01					
V3SVHSHC_8832773	NDUFAF3				2.894E-01					
V3SVHSHC_10223228	LTBP1				2.914E-01					
V3SVHSHC_5580920	KLHL18				2.915E-01					
V3SVHSHC_9252368	ZNF532				2.932E-01					
V3SVHSHC_8911445	RBBP9				2.937E-01					
V3SVHSHC_5554883	LOC100293704				2.945E-01					
V3SVHSHC_9824984	KRT80				2.951E-01					
V3SVHSHC_7135451	TMED7-TICAM2				2.959E-01					
V3SVHSHC_6822182	MORF4L1				2.973E-01					

V3SVHSHC_9501518	THUMPD1				2.981E-01					
V3SVHSHC_8637413	FAM198B				2.998E-01					
V3SVHSHC_6839144	TMCC3				3.032E-01					
V3SVHSHC_10320281	GPT				3.037E-01					
V3SVHSHC_4774532	MAPK12				3.073E-01					
V3SVHSHC_8389022	FAM129C				3.077E-01					
V3SVHSHC_6877754	KRTAP26-1				3.098E-01					
V3SVHSHC_10216958	RXRB				3.106E-01					
V3SVHSHC_9103802	CYC1				3.110E-01					
V3SVHSHC_6534554	IFIT1				3.110E-01					
V3SVHSHC_9077534	PTPLAD2				3.116E-01					
V3SVHSHC_9687176	ELP4				3.121E-01					
V3SVHSHC_8771591	ERAP1				3.126E-01					
V3SVHSHC_6494558	SLC35D3				3.147E-01					
V3SVHSHC_8237420	DOK2				3.150E-01					
V3SVHSHC_5103872	NSUN3				3.158E-01					
V3SVHSHC_4913660	ARF4				3.183E-01					
V3SVHSHC_8047802	Eif6				3.203E-01					
V3SVHSHC_9234350	Eif6				3.220E-01					
V3SVHSHC_6312860	CCDC135				3.233E-01					
V3SVHSHC_6481853	C6ORF118				3.236E-01					
V3SVHSHC_4687313	PPP2R1A				3.243E-01					
V3SVHSHC_8037770	PI4KB				3.244E-01					
V3SVHSHC_4635470	SLFN11				3.246E-01					
V3SVHSHC_7034966	UBTF				3.250E-01					
V3SVHSHC_5629628	FCGR3B				3.258E-01					
V3SVHSHC_5974082	ARRDC3				3.279E-01					
V3SVHSHC_7410110	AURKA				3.294E-01					
V3SVHSHC_5567885	BNC2				3.295E-01					
V3SVHSHC_8788586	CENPU				3.296E-01					
V3SVHSHC_10294541	OR4M1				3.297E-01					
V3SVHSHC_6859736	ZNF286B				3.306E-01					
V3SVHSHC_7224419	C6orf58				3.309E-01					
V3SVHSHC_5820236	GPR88				3.332E-01					
V3SVHSHC_5085821	KRTCAP3				3.347E-01					
V3SVHSHC_10652756	SLC4A10				3.365E-01					
V3SVHSHC_6777434	HUNK				3.375E-01					
V3SVHSHC_6037442	Tmem190				3.384E-01					
V3SVHSHC_9247517	ZNF214				3.404E-01					
V3SVHSHC_10360937	HS6ST1				3.410E-01					
V3SVHSHC_5272832	ERVMER34-1				3.414E-01					
V3SVHSHC_5657216	RND3				3.414E-01					
V3SVHSHC_6498518	LOC101930441				3.422E-01					
V3SVHSHC_7026980	SUPT4H1				3.438E-01					
V3SVHSHC_9372818	IL17RB				3.446E-01					
V3SVHSHC_10780235	UHRF1				3.448E-01					
V3SVHSHC_8232899	IFRD2				3.465E-01					
V3SVHSHC_6662825	FAM217B				3.476E-01					
V3SVHSHC_5627978	ZZEF1				3.482E-01					
V3SVHSHC_9277976	H3F3B				3.491E-01					
V3SVHSHC_4773872	SULT1E1				3.492E-01					
V3SVHSHC_4749782	LRRC31				3.497E-01					
V3SVHSHC_6530957	SCGB1D1				3.502E-01					
V3SVHSHC_5242274	AQR				3.503E-01					
V3SVHSHC_9499967	SLC1A6				3.513E-01					

V3SVHSHC_7192508	AKAP11				3.518E-01					
V3SVHSHC_10145942	AKR1B15				3.521E-01					
V3SVHSHC_8480696	OR4F15				3.526E-01					
V3SVHSHC_7190429	NUP210				3.528E-01					
V3SVHSHC_8234648	TINAG				3.530E-01					
V3SVHSHC_7464923	PLD3				3.535E-01					
V3SVHSHC_7626755	SYNGR4				3.536E-01					
V3SVHSHC_8340941	MTL5				3.541E-01					
V3SVHSHC_7062587	C12orf77				3.562E-01					
V3SVHSHC_9651668	GRPEL1				3.566E-01					
V3SVHSHC_10427399	WTH3DI				3.585E-01					
V3SVHSHC_9654077	FAM109A				3.585E-01					
V3SVHSHC_5590094	LETMD1				3.589E-01					
V3SVHSHC_6308438	R3HDML				3.596E-01					
V3SVHSHC_7520330	H3F3B				3.597E-01					
V3SVHSHC_9313022	ZBTB20				3.600E-01					
V3SVHSHC_5128985	PGM2L1				3.610E-01					
V3SVHSHC_7158287	GDPD3				3.624E-01					
V3SVHSHC_7506998	WWC1				3.640E-01					
V3SVHSHC_10381397	HTR1F				3.654E-01					
V3SVHSHC_10214252	POLR1E				3.662E-01					
V3SVHSHC_6189605	F10				3.670E-01					
V3SVHSHC_9379319	OR5A2				3.672E-01					
V3SVHSHC_6379520	DENND6B				3.682E-01					
V3SVHSHC_7972529	C1orf210				3.684E-01					
V3SVHSHC_6289100	ALG10B				3.689E-01					
V3SVHSHC_10723343	DGKB				3.697E-01					
V3SVHSHC_5318867	DOCK10				3.714E-01					
V3SVHSHC_8292035	Snrnp25				3.715E-01					
V3SVHSHC_9478979	ATP13A5				3.719E-01					
V3SVHSHC_6334376	SIDT2				3.725E-01					
V3SVHSHC_4670747	LOC101928761				3.726E-01					
V3SVHSHC_8211482	TRMT112				3.748E-01					
V3SVHSHC_9731462	STAT6				3.749E-01					
V3SVHSHC_6889073	HDLBP				3.754E-01					
V3SVHSHC_7611905	NARS				3.758E-01					
V3SVHSHC_9899003	ZBTB43				3.771E-01					
V3SVHSHC_5570261	ITGB1				3.771E-01					
V3SVHSHC_9637247	RPN2				3.786E-01					
V3SVHSHC_5555708	EVL				3.792E-01					
V3SVHSHC_5553200	BARD1				3.801E-01					
V3SVHSHC_4762322	ANKRD22				3.812E-01					
V3SVHSHC_7343681	CGNL1				3.821E-01					
V3SVHSHC_9170099	EDNRA				3.822E-01					
V3SVHSHC_5007380	ENOX1				3.823E-01					
V3SVHSHC_5172545	CBLC				3.828E-01					
V3SVHSHC_5997578	HIF3A				3.833E-01					
V3SVHSHC_9810563	LRRC2				3.852E-01					
V3SVHSHC_7234286	SPATA33				3.858E-01					
V3SVHSHC_6816638	THEMIS2				3.859E-01					
V3SVHSHC_6043811	SSX2				3.862E-01					
V3SVHSHC_4979297	C8ORF4				3.870E-01					
V3SVHSHC_8172476	ZNF202				3.884E-01					
V3SVHSHC_9533429	ATP1B3				3.890E-01					
V3SVHSHC_6427667	RBBP9				3.917E-01					

V3SVHSHC_6218216	RPS6KL1				3.930E-01					
V3SVHSHC_10317641	FAM209A				3.934E-01					
V3SVHSHC_7913261	TXK				3.935E-01					
V3SVHSHC_7367804	PSME2				3.937E-01					
V3SVHSHC_4953260	OIT3				3.945E-01					
V3SVHSHC_10705424	KIFC2				3.955E-01					
V3SVHSHC_9331535	DRC1				3.959E-01					
V3SVHSHC_8490596	TFAM				3.966E-01					
V3SVHSHC_6668336	MRPL44				4.007E-01					
V3SVHSHC_9301703	LINC00684				4.007E-01					
V3SVHSHC_10531382	ZBED2				4.011E-01					
V3SVHSHC_7906595	JMJD7-PLA2G4B				4.018E-01					
V3SVHSHC_10401329	NAV1				4.021E-01					
V3SVHSHC_7700180	PXT1				4.022E-01					
V3SVHSHC_5190002	ROBO1				4.026E-01					
V3SVHSHC_5226830	NLRP2				4.028E-01					
V3SVHSHC_7082750	METTL2B				4.041E-01					
V3SVHSHC_10701563	FCER2				4.045E-01					
V3SVHSHC_7810862	CDK2				4.058E-01					
V3SVHSHC_7847855	C12orf65				4.083E-01					
V3SVHSHC_9975497	LONP1				4.087E-01					
V3SVHSHC_6304148	GPR65				4.088E-01					
V3SVHSHC_10074002	LMAN2				4.100E-01					
V3SVHSHC_7683713	AXL				4.101E-01					
V3SVHSHC_8176799	SSR2				4.119E-01					
V3SVHSHC_5200925	PPP1R16A				4.123E-01					
V3SVHSHC_8207753	TPO				4.126E-01					
V3SVHSHC_8407667	ZNF689				4.127E-01					
V3SVHSHC_5149676	HSPBP1				4.135E-01					
V3SVHSHC_5418527	SYNPO				4.142E-01					
V3SVHSHC_8692622	ILVBL				4.143E-01					
V3SVHSHC_7082651	RHBDL1				4.144E-01					
V3SVHSHC_7215377	RAB2A				4.181E-01					
V3SVHSHC_9576263	ANKFN1				4.186E-01					
V3SVHSHC_7598540	LEAP2				4.188E-01					
V3SVHSHC_10658960	AGA				4.193E-01					
V3SVHSHC_6805484	PHOX2A				4.196E-01					
V3SVHSHC_6164063	AQP6				4.205E-01					
V3SVHSHC_9115055	GRIA4				4.219E-01					
V3SVHSHC_10278239	KIR2DL5B				4.221E-01					
V3SVHSHC_10212272	SAMD15				4.232E-01					
V3SVHSHC_8691500	RNASE9				4.234E-01					
V3SVHSHC_4677281	PRLR				4.251E-01					
V3SVHSHC_6237983	FBXO22				4.254E-01					
V3SVHSHC_8331602	LOC100293704				4.257E-01					
V3SVHSHC_6344804	MMP19				4.266E-01					
V3SVHSHC_4711931	PORCN				4.292E-01					
V3SVHSHC_7120139	EDNRB				4.300E-01					
V3SVHSHC_6208712	SAP130				4.302E-01					
V3SVHSHC_7151522	FNDC3B				4.308E-01					
V3SVHSHC_6073907	MEA1				4.311E-01					
V3SVHSHC_5551979	PFDN4				4.314E-01					
V3SVHSHC_7581710	Glipr2				4.326E-01					
V3SVHSHC_8916527	MAPK4				4.326E-01					
V3SVHSHC_10060901	MAD2L1				4.336E-01					

V3SVHSHC_5325896	DENND5A				4.339E-01					
V3SVHSHC_8580389	HCRTR1				4.351E-01					
V3SVHSHC_6442715	TP63				4.356E-01					
V3SVHSHC_9344504	TBC1D32				4.391E-01					
V3SVHSHC_10470530	CYP39A1				4.391E-01					
V3SVHSHC_7268507	RAB24				4.401E-01					
V3SVHSHC_4949366	DYNLT3				4.415E-01					
V3SVHSHC_10034798	GBP1				4.428E-01					
V3SVHSHC_10065125	MMP7				4.433E-01					
V3SVHSHC_9250487	HSD17B6				4.436E-01					
V3SVHSHC_6804065	PRR5-ARHGAP8				4.442E-01					
V3SVHSHC_6243824	EPSTI1				4.443E-01					
V3SVHSHC_9832937	MTX1				4.443E-01					
V3SVHSHC_10317377	ZNF473				4.446E-01					
V3SVHSHC_9962660	MYH15				4.447E-01					
V3SVHSHC_4877393	DYNLL2				4.447E-01					
V3SVHSHC_5491820	MAN1C1				4.448E-01					
V3SVHSHC_6874817	BMP4				4.455E-01					
V3SVHSHC_10047140	C1orf226				4.465E-01					
V3SVHSHC_10593587	VMO1				4.474E-01					
V3SVHSHC_8364470	Mob1b				4.482E-01					
V3SVHSHC_6219371	CCDC107				4.482E-01					
V3SVHSHC_9422318	RYR3				4.483E-01					
V3SVHSHC_5968175	TCTE1				4.486E-01					
V3SVHSHC_9397634	EPHX3				4.486E-01					
V3SVHSHC_7614050	TM9SF2				4.490E-01					
V3SVHSHC_8045888	ECH1				4.496E-01					
V3SVHSHC_7492412	TTLL7				4.498E-01					
V3SVHSHC_7195016	GFI1				4.525E-01					
V3SVHSHC_6899039	SGCG				4.528E-01					
V3SVHSHC_9108257	FAM65A				4.529E-01					
V3SVHSHC_9915734	EEFSEC				4.532E-01					
V3SVHSHC_5400707	DSCR4				4.532E-01					
V3SVHSHC_8367704	LOC729159				4.533E-01					
V3SVHSHC_4908611	RAPGEF6				4.539E-01					
V3SVHSHC_10127891	CLINT1				4.542E-01					
V3SVHSHC_8701433	CNR1				4.543E-01					
V3SVHSHC_7025330	UNC45A				4.549E-01					
V3SVHSHC_9542999	ARHGAP29				4.553E-01					
V3SVHSHC_9572105	GBAS				4.566E-01					
V3SVHSHC_8122613	DHX40				4.571E-01					
V3SVHSHC_5117039	TMEM251				4.576E-01					
V3SVHSHC_10134986	MGEA5				4.581E-01					
V3SVHSHC_9863198	LOC101930432				4.582E-01					
V3SVHSHC_9488615	KAT5				4.583E-01					
V3SVHSHC_8430932	WDR41				4.591E-01					
V3SVHSHC_5283590	ELOVL2				4.597E-01					
V3SVHSHC_5634248	TTLL13				4.597E-01					
V3SVHSHC_9664604	OR2W5				4.598E-01					
V3SVHSHC_5097767	GAB3				4.600E-01					
V3SVHSHC_10226990	LARGE				4.602E-01					
V3SVHSHC_8297711	XPNPEP2				4.617E-01					
V3SVHSHC_4748990	FICD				4.631E-01					
V3SVHSHC_9920024	KCNK13				4.634E-01					
V3SVHSHC_4672925	NDUFAF5				4.638E-01					

V3SVHSHC_8161916	FASLG				4.652E-01						
V3SVHSHC_8808551	NUDT3				4.682E-01						
V3SVHSHC_8461655	SERPINE3				4.687E-01						
V3SVHSHC_8983583	PCDHA12				4.718E-01						
V3SVHSHC_7870328	AGAP10				4.720E-01						
V3SVHSHC_7453736	URB1				4.727E-01						
V3SVHSHC_8133866	G6PC				4.732E-01						
V3SVHSHC_8761691	ACAT1				4.734E-01						
V3SVHSHC_8102351	SHOX				4.757E-01						
V3SVHSHC_8742947	CTNNA2				4.788E-01						
V3SVHSHC_10074662	DPPA3				4.799E-01						
V3SVHSHC_7491059	IVD				4.803E-01						
V3SVHSHC_7479146	SYT16				4.824E-01						
V3SVHSHC_9658565	NQO1				4.825E-01						
V3SVHSHC_9872570	PRR20E				4.829E-01						
V3SVHSHC_10050539	NMNAT2				4.830E-01						
V3SVHSHC_6215015	RAVER2				4.833E-01						
V3SVHSHC_4810073	ADGB				4.833E-01						
V3SVHSHC_7728527	ALG2				4.841E-01						
V3SVHSHC_9576626	CHIC1				4.849E-01						
V3SVHSHC_8595239	NOX3				4.852E-01						
V3SVHSHC_10472312	SIRPD				4.853E-01						
V3SVHSHC_7508615	TMEM185A				4.859E-01						
V3SVHSHC_6263228	LOC100652871				4.864E-01						
V3SVHSHC_10668299	ASPSCR1				4.868E-01						
V3SVHSHC_5614481	CMTM4				4.869E-01						
V3SVHSHC_10412021	EVI2B				4.875E-01						
V3SVHSHC_6228380	KLHL2				4.883E-01						
V3SVHSHC_7042160	CCNK				4.889E-01						
V3SVHSHC_10460300	AKT2				4.893E-01						
V3SVHSHC_7399286	TMEM120B				4.916E-01						
V3SVHSHC_8905670	PPP1R14C				4.924E-01						
V3SVHSHC_9532736	DCSTAMP				4.927E-01						
V3SVHSHC_8382554	IL15				4.930E-01						
V3SVHSHC_7702457	OMG				4.934E-01						
V3SVHSHC_9804821	ZNF565				4.943E-01						
V3SVHSHC_8089217	CIDEB				4.952E-01						
V3SVHSHC_5396648	BDH2				4.973E-01						
V3SVHSHC_7404764	CYP1B1				4.975E-01						
V3SVHSHC_8254415	OR52E2				4.976E-01						
V3SVHSHC_6564089	SOAT1				4.984E-01						
V3SVHSHC_9795185	ATRNL1				4.991E-01						
V3SVHSHC_8352326	RDH13				4.997E-01						

**Control (untreated)**	**HU**	**Dox**	**HU+Dox**
AAAS	AARSD1	A4GNT	A2ML1
ABCG2	ABCA13	AADAC	AAED1
ABR	ABCB6	AAR2	ABCA13
ACAA2	ABCC4	AARS	ABCF2
ACAT1	ACE	ABCA3	ABCG5
ACBD4	ACSL5	ABCC12	ABHD12
ACCSL	ACTC1	ABHD13	Acap3
ACOT1	ACTR3	ABR	ACIN1
ACOT9	ACTRT1	ACAP1	ACOT11
ACSM2A	ACVR1B	ACCSL	ACOXL
ACTC1	ADAM8	ACE2	ACRBP
ACTG1	ADAMTS19	ACOT7	ACY3
ACTN1	ADD1	ACSF3	ADAMTS10
ACTN2	ADPRHL1	ACSM3	ADAMTSL1
ACTN4	ADPRHL2	ACSS1	ADAMTSL3
ACTR1B	ADRBK2	ACTA1	ADCK1
ACTR8	AEBP1	ACTC1	ADH7
ACTRT3	AFP	ACTN1	ADHFE1
ACVR2B	AGAP5	ACTR3C	AGAP5
ADAL	AGBL4	ACTR6	Agfg2
ADAM15	AGXT	ADAM29	AGGF1
ADAMTS19	AICDA	ADAM8	AGO4
ADAMTS2	AIFM2	ADAMTS9	AGXT
ADAMTS3	AIM2	ADAMTSL2	AHDC1
ADCK1	AJAP1	ADAT2	AIMP2
ADD3	AKAP3	Adat3	Akip1
ADH1A	Aknad1	ADCY4	AKR1C3
ADH4	ALDH3A1	ADCY5	AKT1
ADORA2A	ALDH3B1	ADCY6	AKT1S1
ADRA2C	ALOXE3	ADD1	ALDH1A3
ADRBK1	ALPI	ADHFE1	ALDH4A1
ADSS	AMER2	ADIG	ALDH6A1
AFF1	AMIGO2	ADIPOR1	ALG6

AGA	ANAPC7	ADORA2A	ALPP
AGBL1	ANGPTL4	ADPRH	AMIGO2
AGBL2	ANKLE2	ADRBK1	ANGPT4
AGMAT	ANKRD18A	AEBP2	ANKH
AGPAT9	ANKRD37	AFF3	Ankle1
AHSG	ANKS1A	AFTPH	ANKRD1
AIFM2	ANP32A	Agap1	ANKRD13B
AIG1	ANXA8L1	AGBL4	ANKRD30A
AIM1L	APOBEC3F	AGK	Ankrd52
AK8	APPBP2	AGL	ANKRD54
AKAP14	AQP1	AGPAT6	ANKRD60
AKR1C1	ARFIP1	AHSG	ANKRD62
AKTIP	ARHGAP40	AIF1L	ANXA1
ALDH6A1	ARHGAP9	AIG1	AP3B1
ALG12	ARHGEF7	AIMP2	AP3M2
ALG14	ARMCX5	AKAP13	APOA1BP
ALLC	ARRDC1	AKAP8	APOA5
ALX3	ARSA	AKR1C2	APOC2
AMELY	ARSF	AKT2	AQP5
AMMECR1L	ASB4	ALDH8A1	AR
AMN1	ASB7	ALDOB	ARFGAP1
AMTN	ASB9	ALKBH5	ARFIP1
ANAPC2	ASIP	ALKBH6	ARFIP2
ANGPTL4	ASMTL	ALKBH8	ARHGEF11
ANKFN1	ASPA	ALOX5AP	ARHGEF3
Ankle1	ASPH	AMACR	ARL14
ANKRD13D	ATAD5	AMELX	ARL6IP5
ANKRD34B	ATP11AUN	AMER2	ARNT2
ANKRD54	ATP13A4	AMFR	ARPC3
ANKRD55	ATP5G2	AMIGO1	ARRDC5
ANKRD66	ATP6V0E1	AMY1C	ARSE
ANKRD7	ATXN2L	ANG	ASAH2B
ANP32C	AURKAIP1	ANGEL2	ASCC3
ANXA2R	B3GAT2	ANGPT1	ASF1B
ANXA8L1	B4GALNT4	ANGPT4	ASMTL

AP2B1	BACE2	ANKK1	ASNSD1
AP5S1	BAG5	ANKLE2	Aspg
APBB1	BAIAP2	ANKRD1	ATF1
APLN	BANF1	ANKRD20A2	ATF7IP2
APMAP	BCKDHA	ANKRD23	ATL1
APOA1BP	BEST4	ANKRD35	ATL2
Apobr	BEX5	ANKRD37	ATP5O
APOC4	BFSP1	ANKS1A	ATP5SL
APOL5	BGLAP	ANKS3	ATP6V0E1
APOL6	BIRC7	ANO6	ATP6V1F
AQP11	BLZF1	ANPEP	ATP7A
AQP4	BNIP2	ANXA13	ATRAID
AQP7	BOLA2B	ANXA7	AVPR1A
AQP8	BPIFB3	AOC3	AVPR2
AREL1	BRD2	AP3B1	AXDND1
ARF3	BRD8	AP5B1	AXIN1
ARF5	BRF2	AP5S1	B3GALT1
ARHGAP15	BRSK1	APAF1	B4GALT5
ARHGAP44	BTG3	APH1B	B4GALT6
ARHGDIG	BTNL2	API5	BAGE3
ARHGEF19	BUB1	APLN	BAGE4
ARID2	C10ORF53	APOB	BAI2
ARL11	C10orf62	APOBEC3A	BAMBI
ARL4D	C11orf95	APOH	BAZ1A
ARL8B	C12orf49	APOLD1	BAZ1B
ARMC5	C15orf32	AQP4	BBIP1
ARMCX5	C15orf57	AREL1	BCAT2
ARPC5L	C17orf98	ARF4	BEX1
ASPDH	C18ORF25	Arglu1	BFSP1
Aspg	C19orf48	ARHGAP19	BIK
ASTN2	C19orf68	ARHGAP22	BLCAP
ATE1	C1orf106	ARHGAP23	BLVRB
ATF6	C1orf109	ARHGAP42	BMP10
ATG16L2	C1orf162	ARHGAP6	BMPR2
ATIC	C1orf194	ARHGEF25	BNC2

ATP10A	C1orf56	ARHGEF39	BPI
ATP1B4	C1orf68	ARID5B	BRINP1
ATP2B2	C1QL4	ARL17B	BRSK2
ATP6V1A	C20ORF166	ARL2BP	BTBD11
ATP7A	C21ORF33	ARL4D	BTC
ATPAF1	C22orf46	ARMC8	BUD13
AURKC	C2cd4a	ARMC9	C10ORF76
AXDND1	C2ORF16	ARNT2	C12orf40
B3GNT6	C2orf61	ARPC1B	C12orf71
B4GALT1	C3AR1	ARRDC3	C12orf76
B4GALT7	C4orf33	ARSE	C14orf177
BAAT	C4ORF6	ASB14	C14ORF79
BAD	C5orf38	ASB15	C15orf32
BAZ1B	C5orf52	ASB16	C15orf53
BCL6	C5orf66	ASB17	C15orf54
BDH2	C6ORF165	ASB18	C15orf57
Bend3	C6orf183	ASCL1	C15orf61
BMP2	C6ORF89	Asprv1	C16orf72
BMP6	C8orf33	ASS1	C16orf93
BPIFA2	C8orf88	ASXL3	C16orf96
BPIFB2	C9orf16	ATCAY	C18ORF21
BRDT	C9orf163	ATF6	C19ORF18
BRINP1	C9orf169	ATF6B	C19orf35
BRINP2	CABLES1	ATF7IP2	C1GALT1
BRSK2	CACNA2D1	ATIC	C1GALT1C1
BSN	CACNB3	ATOH7	C1orf61
BSND	CACNG1	ATP10D	C1orf87
BTF3	CACNG8	ATP13A2	C1QTNF3
C10orf113	CADM3	ATP2B4	C1QTNF6
C12orf45	CALY	ATP2C2	C1QTNF7
C12orf49	CAMK2N1	ATP4A	C20ORF27
C12ORF5	CAPN6	ATP4B	C2ORF16
C12orf57	CAPZB	ATP5S	C2orf27B
C14ORF166	CARM1	ATP6AP1	C2orf42
C15orf48	CASK	ATP6V0A2	C3orf27

C15orf60	CASP8	ATP6V0C	C3orf62
C17orf112	CASP9	ATP6V0E1	C6ORF25
C17orf62	CCDC107	ATP9A	C7ORF34
C17orf67	CCDC129	ATRX	C7orf60
C19ORF12	CCDC17	AVIL	C7orf62
C19orf38	CCDC64	AVL9	C9orf139
C1orf105	CCDC80	B2M	C9ORF37
C1orf123	CCL11	B3GNT2	C9orf92
C1orf127	CCL19	B4GALNT4	CA13
C1orf194	CCNC	BAGE	CA6
C1orf198	CCND2	BAGE2	CACHD1
C1orf229	CCNJ	BAGE5	CACNA1A
C1orf234	CD300C	BAI1	CACNA1B
C1QL3	CD74	BAIAP2L2	CACNA1I
C1QTNF4	CD79B	BAIAP3	CACNB1
C1RL	CD86	BAK1	CACNB2
C20ORF166	CD8B	BBOX1	CACNG7
C20ORF85	CDC25C	BBS10	CALCRL
C21ORF62	CDC34	BBS5	CALML5
C22orf24	CDC42EP3	BCL9	CALML6
C2orf54	CDH6	BCL9L	CAPN10
C3orf27	CDHR1	BCOR	CAPN12
C3orf36	CDK19	BDH2	CAPN3
C5orf24	CDKL3	BEST1	CAPS
C5orf55	CDPF1	BGN	CAPZA1
C6ORF15	CDR2	BIRC6	CARD14
C6orf7	CECR2	BIRC7	CARD9
C8orf82	CELSR1	BLOC1S3	CARNS1
C9orf163	CELSR2	BMP4	CASP16
C9ORF9	CEMIP	BMP7	CBFB
C9orf92	CEP164	BOD1L2	CBWD2
CA1	CEP57L1	BOK	CBX6
CA3	CGB1	BOLA1	CCDC121
CACNA1C	CGB8	Bpifb6	CCDC22
CACNG3	CHAF1A	BRINP1	CCDC28B

CALCOCO1	CHD9	BRSK1	CCDC33
CALCOCO2	CHIC2	BRSK2	CCDC68
CAMK2B	Chpf2	BSX	CCDC82
CAMK2N1	CHRNA9	BTAF1	CCDC87
CAMK2N2	CIB3	BTG2	CCKBR
Camsap3	CITED2	BTLA	CCL28
CAPN1	CLCN6	BTN3A3	CCL3L1
CAPN9	CLCNKA	BTNL10	CCNG1
CASP12	CLEC2A	BUB1B	CD248
CASP7	CLEC2L	C10ORF11	CD276
CASZ1	CLEC6A	C10ORF91	CD300LD
CATIP	CLEC9A	C11orf21	CD40LG
CAV1	CLIP1	C11ORF31	CD47
CBFB	CLN6	C11orf58	CD6
CBR1	CLPTM1L	C11orf70	CD79B
CBWD7	CLUL1	C11orf82	CD9
CCDC104	CMTM6	C11orf87	CDADC1
CCDC127	CNDP2	C11orf95	CDC14A
CCDC140	CNOT7	C12ORF10	CDC42BPG
CCDC149	CNTF	C12orf40	CDCP1
CCDC178	COL13A1	C12orf43	CDH17
CCDC30	COL25A1	C12orf50	CDH2
Ccdc39	COL9A2	C12orf65	CDHR3
CCDC64	COPS2	C12orf80	CDK3
CCDC79	COQ7	C14ORF1	CDKL5
CCDC86	COX6B1	C14ORF2	CDY2B
CCDC87	COX7A2L	C15orf54	CEACAM4
CCDC92	CPNE4	C15orf56	CECR6
CCK	CPO	C15orf57	CENPO
CCL13	CREBZF	C15orf61	CEP250
CCL14	CRY1	C16orf74	CEP85L
CCL20	CRYBA4	C17orf104	CERCAM
CCL24	CRYGA	C17orf70	CES3
CCL25	CSNK1G3	C17orf75	CFL2
CCL5	CSNK2A3	C17orf78	CHD6

CCNB2	CSTF1	C17orf80	CHL1
CCNG2	CSTF2T	C17orf98	CHRM3
CCR9	CT47A8	C18orf56	CIART
CD160	CT83	C19ORF26	CIDEC
CD302	CTIF	C19orf33	CISD3
CD37	CTNNB1	C1GALT1	CLCN6
CD68	CTRC	C1orf112	CLCNKB
CD69	CTSG	C1orf159	CLDN1
CD70	CTTNBP2NL	C1orf168	CLDN15
CD81	CUEDC2	C1orf95	CLDN16
CD83	Cul9	C1QTNF5	CLDN18
CDC20B	CXCL6	C1R	CLDND1
CDC37	CXCL9	C1RL	CLEC17A
CDH18	CXorf57	C1S	CLEC2A
CDH19	CYB5RL	C20orf196	CLEC4G
CDH26	CYP2C9	C20ORF85	CLEC4M
CDK11A	CYP2D6	C21ORF58	CLIC3
CDK2	CYP7B1	C2cd2	CLINT1
CDR2	DAGLA	C2orf40	CLIP1
CDR2L	DAPK1	C2orf47	CLPB
CECR2	DCDC2C	C2orf49	CMTM5
CEP250	DDA1	C2orf69	CNGA2
CFDP1	DDX47	C2orf81	CNST
CHD6	DEFA4	C2orf88	CNTF
CHIC2	DEFB104A	C3AR1	CNTN3
CHML	DEFB107A	C4orf26	COL14A1
CHMP3	DEFB123	C4orf27	COL16A1
CHRDL2	DEFB131	C5AR2	COL1A2
CHRFAM7A	DENND3	C5orf22	COL24A1
CHST5	DET1	C5orf51	COL25A1
CIRBP	DGKG	C5orf54	COL26A1
CLCN2	DGKK	C5orf60	COL2A1
CLDN10	DHCR24	C6	COPRS
CLDN6	DHTKD1	C6ORF195	COPS2
CLEC16A	DHX40	C8orf76	COX16

CLIP1	DLG1	C9orf169	COX7A1
CLK4	DLX1	C9ORF72	CPO
CLPP	DNAJB4	C9ORF78	CPSF4L
CLTA	DNAJB7	C9ORF84	CRISP1
CNIH1	Dnajc30	C9ORF85	CSF1R
CNNM1	DNALI1	C9ORF89	CSMD1
CNNM3	DNASE1L1	C9ORF9	CSNK1G1
CNOT11	DNM3	C9ORF91	CSNK2A1
CNTNAP4	DOCK1	CA7	CTNNB1
CNTROB	DPCR1	CACFD1	CUL4A
COA6	DUOXA2	CACNA1E	CXCL14
COL26A1	DUSP26	CACNA1F	CXCL3
COL28A1	DUT	CACNB1	CXCR4
COL3A1	DVL2	CALHM1	CYB561
COPS2	DYDC1	CALHM3	CYBA
CORO2B	DZANK1	CALML5	CYFIP1
COX19	EBAG9	CAMP	CYP1A1
COX6B2	EBI3	CARD14	CYP46A1
CPE	EDN3	CARD16	CYR61
CPLX3	EEF2	CARD18	DAZAP2
CPNE8	EFCAB13	CARHSP1	Dcaf8
CRADD	EGFR	Carkd	DCP1B
CRAT	EHD1	CASC10	DCT
CRCP	EHD4	CASKIN1	DDX3X
CREB3L2	EIF3F	CASQ1	DEFB103A
CRELD2	EIF3H	CASQ2	DEFB116
CRIP2	ELAVL4	CATSPERG	DEFB131
CRLF3	ELP5	CBFA2T2	DENND1C
CRTAP	EML3	CBX6	DENND4A
CSF1	ENDOG	CCBE1	DFNA5
CSNK1G2	ENTPD8	CCDC104	DGKI
CSNK2A2	EPN2	CCDC107	DGKK
CSRNP3	EPS15	CCDC115	DHCR24
CST3	EPT1	CCDC120	DHRS4
CST6	EQTN	Ccdc138	DHRS7C

CST7	ERBB2IP	CCDC166	DHX36
CT45A2	EREG	CCDC168	DIABLO
CT47A9	ERICH2	CCDC174	DIDO1
CTAG1B	ESR2	CCDC177	DIRAS2
CTBP1	EXOC1	CCDC178	DIS3L
CTNNA2	EZH1	CCDC27	DLGAP1
Cul9	F2RL1	CCDC36	Dlgap5
CX3CR1	FABP5	CCDC54	Dlk2
CXCL1	FAM107B	CCDC59	DMC1
CXCR4	FAM127A	CCDC68	DNAH8
CXorf57	FAM134B	CCDC85A	DNAJB4
CYB561D1	Fam150a	CCDC90B	DNAJC10
CYB5D2	FAM151A	CCL2	DNASE2B
CYB5R4	FAM160B2	CCL22	DNMT1
CYP2A7	FAM166A	CCL3L3	DOCK11
DAGLA	Fam181b	CCM2L	DPH1
DAGLB	FAM189A2	CCNDBP1	DPY19L2
DALRD3	FAM189B	CCNF	DPYSL3
DARS	FAM195B	CCNY	DRD1
DAXX	FAM198B	CCRL2	DSCAM
DBH	FAM217A	CCZ1B	DSCR3
DBR1	FAM46A	CD151	DTD1
DCAF15	FAM65A	CD1B	DTWD2
Dcaf17	FAM69C	CD1E	DUOX1
DCDC5	FAM83D	CD207	DUSP1
DCPS	FAM86C1	CD226	DUSP2
DCTN2	FASTKD5	CD2AP	DUSP21
DCUN1D2	FBLN7	CD302	DUSP26
DDX47	FBXL12	CD4	DUSP3
DDX51	FBXL5	CD40	DUSP5
DDX58	FBXO21	CD69	DZIP1L
DEFB104A	FBXW9	CD82	E2F7
DEFB115	FCRL4	CD93	Eapp
DEFB118	FCRL6	CDC14B	EDEM1
DEFB128	FEM1C	CDC25C	EDRF1

DEFB4B	FEZF2	CDC42BPG	EEA1
DENND6B	FGFBP1	CDC42SE2	EFCC1
DERA	FGG	CDCA7L	EGFL6
DGKI	FGR	CDH10	EGLN2
DGUOK	FHL5	CDH18	EGLN3
DIABLO	FILIP1L	CDK10	EHD3
DIAPH2	FKBP14	CDK2	EIF3H
DIAPH3	FKBP15	CDK3	EIF4ENIF1
DIO3	FKTN	CDK5	ELAC2
DIP2B	FLNC	CDK5R1	ELF2
DIRC2	FOS	CDKN3	ELFN2
DIS3L2	FOSL2	CDRT15	ELL2
DISP1	FOXC1	CDY2A	ELMOD2
DLX3	FOXI1	CEACAM16	ELOVL7
DMD	FOXL1	CEACAM19	EMC4
DMTN	FRAT2	CEACAM5	EML2
DNAJA3	FRMD8	CEBPD	EML4
DNAJB1	FRRS1L	CECR1	EMR1
DNAJB12	FUCA1	Cela1	ENKD1
DND1	FUT6	Celf4	ENPEP
DNM3	FXYD4	CEP128	ERBB2IP
DOPEY1	G0S2	CEP170B	ERGIC2
Dpy30	GABARAPL1	CEP55	Ermn
DQX1	GABBR1	CEP57	ERP44
DRD5	GAGE12J	CERCAM	ESPNL
DSC1	GAL3ST1	CERKL	ESYT3
DSCAML1	GALNT1	Cers2	ETV7
DSCC1	GALNT8	CFL1	EVA1A
DSG2	GAPDHS	CGRRF1	F11R
DSP	GAPT	CHAMP1	F8A1
DTWD2	GAS7	CHCHD6	FAAH2
DYNAP	GATS	CHCHD7	FADD
DYNLRB2	GATSL3	CHD1	FADS3
DZANK1	GBE1	CHDC2	FAM101A
EARS2	GCA	CHGA	FAM103A1

EBF4	GCHFR	CHI3L2	FAM110B
EBI3	GDI2	CHIA	FAM120A
EBPL	GFI1	CHPT1	Fam131c
ECI1	GFPT2	CHRAC1	FAM153B
EDDM3A	GGT6	CHRDL2	Fam154b
EDDM3B	GIF	CHRM1	FAM155A
EDF1	GIN1	CHRM4	FAM160B2
EED	GJA4	CHRNA6	FAM19A5
EEF1E1	GJA5	CHST10	FAM209A
EEF1G	GJC2	CHST12	FAM20C
EFCAB4B	GK	CHST2	FAM217A
Efcab9	GLI3	CHST8	FAM43B
EFHB	GLIPR1L1	CIART	FAM49B
EGFR	GLIS2	CIB3	Fam71e1
EHBP1L1	GM2A	Cisd2	FASLG
EHMT2	GNA14	CITED2	FBLN7
EIF1	GNG13	CKM	FBXL17
EIF3H	GNLY	CLCN4	FBXL4
ELAVL1	GOLPH3	CLCNKB	FBXL8
ELAVL3	GOSR1	CLDN17	FBXO32
ELF1	GPN2	CLDN19	FBXO48
ELP2	GPR126	CLDND1	FBXO9
EMD	GPR133	Clec18a	FBXW5
EML2	GPR137	CLEC2L	FER1L6
EPHA3	GPR137B	CLEC3A	FFAR4
EPHB6	GPR182	CLEC4E	FGD6
EPHX3	GPR20	CLEC4M	FGF9
EPS15L1	GPR55	CLSPN	FGFR3
EPS8L3	GPR68	CLSTN2	FHAD1
ERCC5	GPS2	CLTA	FILIP1L
Ercc6l	GPX8	CLTC	FKBP15
ERGIC2	GRB7	CLVS1	FOXD4L5
Ermn	Greb1l	CMAS	FOXJ3
ERO1LB	GRP	CMTM3	FOXL2
ESM1	GRPEL2	CNDP2	FOXS1

ESRP2	GRXCR1	CNEP1R1	FRMD5
ESYT3	GSPT1	CNGB1	FRYL
ETFA	GSPT2	CNIH1	FSCN2
ETFDH	GSTM3	CNN2	FSTL1
ETNK1	GSTT2B	CNR2	FUT11
ETV4	GTPBP6	CNTN6	FYN
EVA1B	H2AFY	CNTNAP3B	G0S2
EXD1	H2BFM	COA1	G6PC3
EXT2	HAPLN1	COG2	GAD2
F2	Haus7	COG3	GALNT1
F3	Haus8	COL2A1	GAPDHS
F7	HBG1	COL3A1	GAS2L2
F8A3	HCAR1	COL7A1	GAS2L3
FAAH2	HCFC2	COL8A1	GCM1
FABP2	HEATR3	COMP	GCSAML
FABP5	HELLS	COPS2	GDAP2
FADS2	HERPUD1	CORO1B	GGN
FAF2	HES2	CORO2B	GHR
FAM101B	HIAT1	COX20	GIMAP7
FAM109A	HIBCH	COX5A	GJC3
FAM118B	HIF1AN	COX6B1	GKAP1
FAM175A	HIST1H1T	COX7A2	GLB1L3
FAM177A1	HIST2H2BE	CPA3	GLRA2
FAM19A3	HLA-DOB	CPNE5	GLRX2
FAM20C	Hm13	CPO	GLT1D1
FAM24A	HNRNPD	CPXM2	GMEB1
FAM26E	HNRNPH1	CRB1	GMNN
FAM47A	HNRNPU	CREB5	GNPNAT1
FAM50B	HOMER1	CRK	GOLGA1
FAM65A	HOXA7	CRNN	GOLGA6L4
FAM69C	HOXD8	CRTC2	GP9
FAM71E2	HPDL	CRX	GPC1
FAM72A	HPGDS	CRYAB	GPN3
FAM8A1	HS3ST6	CRYGB	GPR112
FAM90A1	HSD17B4	CRYL1	GPR162

FAM96B	HSPB8	CSAG1	GPR182
FAT2	HSPG2	CSF1	GPR34
FAT3	HTN1	CSF1R	GPR65
FBLN2	IDH3G	CSF2RB	GPR68
FBXO31	IFNL3	CSGALNACT2	GRIN2A
FCAMR	IGF2BP3	CSH2	GRINA
FCF1	IGFALS	CSN2	GRM2
FCHSD2	IKZF4	CSNK1A1	GSG1
FDCSP	IL17D	CSNK1D	GSK3B
FERD3L	IL1RL1	CT45A2	GSPT1
FGF13	IL22	CT45A3	GSTO1
FGF18	IL23A	CT45A4	GTF2B
FGF19	IL3	CT47A8	GTF2H1
FGFBP2	IL5	CT47B1	GZMM
FHL2	IMPAD1	CTAGE15	H2AFX
FIGNL1	INF2	CTAGE9	HAGHL
FIS1	INPP4A	CTBP2	HAVCR2
FITM1	INPP5D	CTBS	HBA2
FKBP14	IRS4	CTLA4	HCAR3
FKBP3	IRX5	CTNNB1	HDAC2
FLAD1	ISM2	CTPS2	HDGFRP3
FMO5	ISPD	CTSF	HDLBP
FNDC3A	ISX	CTSW	HEPHL1
FNDC9	ITPR2	CUL5	HERC5
FNIP2	JADE3	CUTA	HFM1
FOSB	JPH3	CXCL9	HGS
FOSL1	KANSL1L	CXCR4	HHLA2
FOXA1	KAT6A	CXORF23	HIC2
FOXG1	KATNAL2	CXorf58	HINFP
FOXJ1	KBTBD6	CYB561D2	HJURP
FOXK1	KCMF1	CYB5B	HLA-DOA
FOXN1	KCNA6	CYB5D1	HLA-DPB1
FOXO3	KCNB2	CYBA	HLA-DRB4
FOXR2	KCNE4	CYP1A2	HLA-F
FREM1	KCNH2	CYP1B1	HMGN2

FREM3	KCNK1	CYP24A1	HOMER1
FRS3	KCNRG	CYP26A1	HOPX
FSCN1	KCNS2	CYP27B1	HOXA7
FSD2	KCTD10	CYP2A7	HOXB2
FST	KDELC1	CYP2C18	HOXB4
FTMT	KDM5C	CYP2J2	HOXB5
FUT5	KDR	DACT1	HOXD9
FXYD6	KDSR	DAPK1	HPS6
FYN	KIAA0408	DAPK2	HRSP12
G0S2	KIAA1024L	DAXX	HS6ST2
GABARAPL1	KIAA1147	DAZ2	HSD11B1
GABBR1	KIAA1244	DAZAP2	HSD11B1L
GABPB1	KIAA1324L	DCAF12L2	HSFY1
GABRA6	KIAA1377	DCHS1	Hspa13
GABRG2	KIAA1432	DCLRE1B	HSPA14
GABRP	KIAA1462	DCUN1D2	HSPA6
GAGE4	KIAA1683	DDO	HSPBAP1
GAGE8	KIF11	Ddrgk1	HTR2C
GALC	KIF27	DDTL	HTRA1
GARS	KIFAP3	DDX11	HYAL1
GBP2	KIT	DDX26B	HYAL4
GCFC2	KLF12	DDX43	HYI
GCM1	KLHDC2	DDX51	HYPK
GDF2	KLHDC7A	DEFA1B	IDH3G
GDF6	KLHL1	DEFB104B	IDO1
GDPD1	KLHL13	DEFB107A	IFNA2
GEMIN8	KLHL26	DEFB121	IFNL1
GFRAL	KLHL29	DEFB127	IGFBP7
GIP	KLHL4	DENND6A	IGFL3
GJA4	KLHL6	DERA	IGJ
GJC1	KLRC4-KLRK1	DERL1	IL12RB2
GLRA3	KLRD1	DERL2	IL21R
GLTSCR1L	KLRG2	DESI2	IL27RA
GMEB2	KLRK1	DFFB	IL6ST
GMNN	KRAS	DGCR14	IMMT

GNA12	Krt222	DGKE	IMPAD1
GNAT1	KRT25	DGKI	INHA
GNL3	KRT35	DHCR7	INPP4B
GNL3L	KRT71	DHDH	INSIG1
GNS	KRT75	DHPS	IRAK3
GOLGA5	KRTAP10-7	DHRS3	IRX3
GOLGB1	KRTAP2-2	DHRS4L2	ITGB5
GOT2	KRTAP2-3	DIDO1	ITGB7
Gpank1	KRTAP4-11	DISP2	JAK3
GPATCH2L	KRTAP4-2	DLG3	KAAG1
GPBP1	KRTAP5-5	DLGAP1	KANSL1L
GPR113	KRTAP5-9	DLL3	KAT2B
GPR126	KRTAP7-1	DLX1	KAT6B
GPR132	KRTAP9-1	DMBT1	KAZN
GPR137B	Krtdap	DMTN	KCNA5
GPR171	LACTB2	DNAI1	KCNK2
GPR31	LAMB4	DNAJA1	Kcnu1
GPR83	LAMC2	DNAJA2	KCNV2
GPRC5B	LAT2	DNAJB1	KCTD17
GPRIN1	LCMT2	DNAJB6	KDELC2
GPT2	LDB1	DNAJB7	KIAA0226L
GRB10	LEKR1	DNAJB8	KIAA0319L
GRIA4	LEMD3	DNAJC17	KIAA0391
GRIN2B	LGALS7	DNAJC22	KIAA0513
GRIN3A	LHCGR	DNASE1L3	KIAA0586
GRK4	LHFPL3	DNASE2	KIAA0895
GRXCR1	LHPP	DNASE2B	KIAA1024
GRXCR2	LMO2	DNMT3L	KIAA1244
GS1-259H13.2	LMOD3	DOCK1	KIF18B
GSG1	LOC100130357	DOK1	KIF27
GSS	LOC100130705	DOPEY1	KIF5B
GSTM5	LOC100132731	DPF1	KIFAP3
GTF2H3	LOC100288562	DPF3	KLC2
GUCA1B	LOC100505478	DPP7	KLF2
GYG1	LOC100652807	DPPA5	KLHDC2

GYPC	LOC100996720	DRC1	KLHL6
GYS1	LOC100996906	DRD5	KLK13
H2AFJ	LOC101059976	DSC1	KNG1
HAS1	LOC101060588	DSCAM	KPRP
HAVCR1	LOC101060861	DSG2	KRAS
HCAR2	LOC101927989	DSP	KRIT1
HCN1	LOC101928291	DTWD1	KRT1
HEATR2	LOC101928892	DUOXA2	KRT6A
HEATR5B	LOC101928951	DUS4L	KRT72
HERPUD1	LOC101929264	DUSP26	KRT86
HEY2	LOC101929451	DUSP8	KRTAP11-1
HIPK2	LOC101929725	DVL2	KRTAP12-4
HIRIP3	LOC101929829	DVL3	KRTAP13-1
HIST1H1C	LOC101929950	DYDC1	LAMA1
HIST1H2BA	LOC101930085	DYNC2H1	LANCL3
HIST2H2AA4	LOC101930300	DYNLRB2	LAT2
HJURP	LOC101930400	DYNLT3	LCE5A
HK2	LOC101930432	EBF2	LCN2
HK3	LOC101930553	ECHDC2	LDHAL6A
HLA-DRA	LOC101930592	EDEM1	LDHB
HLA-F	LOC645177	EDEM2	LDHD
HMGB3	LOC645202	EEA1	LEFTY1
HMGN2	LOC651959	EFCAB13	LEMD2
HOMER1	LOC729458	EFCAB3	LGMN
HOXC13	LOC730159	EFCAB7	LHFPL5
HOXD11	LOXL1	EFHC1	LHX3
HRASLS5	LPL	EFNA3	LIMA1
Hrct1	LRCH4	EFNB2	LMAN1L
HRH1	Lrit1	EGFL6	LMBR1L
HRH3	LRP2	EIF2AK2	LMTK2
HS2ST1	LRRC10B	EIF3H	LMTK3
HSF1	LRRC14B	EIF4A1	LOC1001293<br>61
HSF4	LRRC37B	EIF4E3	LOC1001303<br>01
HSPA1A	LRRC43	ELAC2	LOC1001303<br>57
HSPB7	LRRC52	ELF3	LOC1001320<br>04

HTN3	LRRTM3	ELMO3	LOC1002929<br>52
HTR1D	LSM11	ELMOD2	LOC1002937<br>04
HTR3B	LSMEM1	EMC10	LOC1005055<br>49
HTR3D	LTB4R2	EMD	LOC1005090<br>91
HYPK	LURAP1L	EMP1	LOC1009963<br>18
ICAM1	Ly6g6f	EMR1	LOC1009963<br>50
IDH3G	LYPD4	ENKD1	LOC1009964<br>05
IDO2	MAD1L1	ENPP3	LOC1009964<br>15
IDUA	MAFF	EPHA1	LOC1009966<br>19
IFNA1	MAK	EPHB2	LOC1009968<br>44
IFNA10	MAN2B2	EPO	LOC1010599<br>76
IFNA2	MANSC1	EPRS	LOC1010600<br>22
IFNL4	MAP1A	ERAL1	LOC1010602<br>75
IGF1	MAP4K1	ERCC8	LOC1010604<br>00
IGLL5	MAPK8IP2	ERICH1	LOC1019275<br>94
IGSF5	MARS2	ERICH4	LOC1019276<br>14
IL11	MCC	ERICH6	LOC1019278<br>48
IL17B	MCCD1	Erlec1	LOC1019280<br>44
IL22RA2	MCTP1	ERLIN2	LOC1019284<br>98
IL23A	MEAF6	ESAM	LOC1019287<br>28
IL2RB	MESP2	ESPN	LOC1019297<br>02
IL31RA	METTL9	ETNK2	LOC1019297<br>25
IL34	Mex3b	EXOSC3	LOC1019298<br>03
IL36A	MFI2	EYA3	LOC1019298<br>04
IL36G	MLLT3	EYS	LOC1019300<br>75
IMPA1	MLPH	F13B	LOC1019301<br>54
IMPAD1	MLST8	F2	LOC1019304<br>00
INF2	MMP16	F9	LOC1019305<br>51
ING1	MMS19	FADS2	LOC285500
ING5	Mob1b	FADS6	LOC391722
INHBB	MOSPD1	FAIM3	LOH12CR1
INSR	MPC1	FAM110A	LOXL1
IPO4	MPHOSPH8	FAM110C	LRIG3
IQCC	MPO	FAM110D	Lrrc16a

IQCD	MRE11A	FAM133A	LRRC25
Iqcf5	MRPL10	FAM151B	LRRC30
IQGAP2	MRPL36	FAM156A	LRRC43
IRAK1	MRPL48	FAM160A1	LRRC49
IRF2	MS4A12	FAM163A	LRRC61
IRX5	Ms4a15	FAM188B	LRRC70
ISL1	MSC	FAM195B	LSM12
ISL2	MSLN	FAM196B	LSM4
ITGAD	Mst4	FAM19A1	LSMEM1
IVL	MTBP	FAM200B	LY75-CD302
Izumo2	MTMR9	FAM206A	LY9
JADE1	MTNR1B	FAM209B	LYPD1
JAG2	MTRNR2L10	FAM229A	LYPLA2
JAM3	MTRNR2L4	FAM25A	LYRM7
KCNAB3	MTX2	FAM45A	LYVE1
KCNB2	MUC13	FAM53B	MAFA
KCNE2	MUC3B	FAM65A	MAGEA10
KCNG4	MX2	FAM76B	MAN2B2
KCNH3	MYH10	FAM78A	MAP3K1
KCNH5	MYL7	FAM78B	MAP3K6
KCNJ9	MYO1C	FAM83B	MAPKAPK3
KCNK16	MYO3A	FAM98A	MARCH2
KCNS2	MYO5B	FAM9B	MARCH4
KCTD14	MYO7B	FANCL	MARK1
			MB
			MCAT
KCTD21	N6AMT1	FARP2	MCC
KDM6B	NAB1	FAT3	MCF2
KDR	NAMPT	FBN2	MCHR1
KIAA0408	NANOS1	FBN3	MCM5
KIAA0513	NCAPG	FBXO36	MCOLN1
KIAA0922	NDUFA11	FBXO46	MEAF6
KIAA0947	NDUFA13	FCER1G	MED1
KIAA1033	NDUFA4L2	FCGR1B	MEIG1
KIAA1045	NDUFV2	FCGR3A	MFAP3

KIAA1211	NECAB2	FCGR3B	MGARP
KIAA1244	NETO2	FCHSD2	MGMT
KIAA1279	NEUROG2	FCRL1	MIER2
KIAA1755	NFATC1	FERMT1	MLLT3
KIDINS220	NFE2L1	FEV	MLLT4
KIF2B	NFKB2	FEZ2	MMADHC
KIFC1	NFKBIB	FGF10	MMD
KIR3DL1	NGRN	FGF9	MMP13
KLF17	NHEJ1	FGFBP3	MMS19
KLF9	NKAPL	FGFR3	MORC2
KLHL13	NLRP14	FGL2	MPEG1
KLHL33	NLRP3	FHAD1	MPHOSPH9
KLHL6	NMUR1	FHIT	MRGPRX4
KRT5	NOM1	FILIP1L	MRPL23
KRTAP13-3	NPAS2	FKBP2	MRPL24
KRTAP5-6	NPFF	FKBP7	MRPL27
LACE1	Nprl2	FLAD1	MRPS18C
LACTB	NT5C1A	FLJ22184	MRPS36
LAGE3	Ntpcr	FLT4	MRPS7
LAMC3	NTSR2	FLVCR1	MS4A12
LARP1B	NUDT6	FLYWCH1	MS4A13
LBP	NUP37	FMN2	Ms4a15
LCAT	NUP62CL	FMNL1	MTA2
LCE2B	NUS1	FMO5	MTDH
LCE5A	NUTM2A	FNDC3A	MTERFD3
LCN12	NXN	FOXA3	MTFR1
LCN2	NXPE2	FOXF2	MTHFR
LCP2	OBSCN	FOXK1	MTIF2
LDB1	ODF3L1	FOXN2	MTMR6
LDHA	OGG1	FOXO6	MTMR7
LDLR	OLFML1	FOXQ1	MTRNR2L1
LEMD1	OPCML	FPGT	MTUS2
LETMD1	OPN1LW	FPR2	MUC5AC
LGALSL	OPTC	FRAT1	MUC5B
LGI1	OR10Z1	FRK	MVP

LGI2	OR11H12	FRMD3	MYEOV
LGSN	OR11H4	FRZB	MYL3
LIF	OR11L1	FSCB	MYO18B
LILRA2	OR12D3	FSTL3	MYO1F
LILRB5	OR13C3	FUOM	MYO3A
LIMA1	OR13D1	FUS	MYO7B
LIN28B	OR13G1	FUT6	MYOF
LIN7A	OR1J2	FUT7	MYOM3
LINC00684	OR1L6	FYB	MYOZ1
LIPI	OR2F2	G0S2	MYOZ3
LIPT2	OR2L8	G3BP2	MYT1
LITAF	OR2Z1	GAB1	NAA35
Llph	OR4B1	GAB3	NAA60
LOC100129083	OR4C6	GABRP	NAAA
LOC100130357	OR4D11	GAGE12J	NAB1
LOC100133128	OR4E2	GAGE2E	NAPG
LOC100144595	OR4F6	GAGE7	NARFL
LOC100288966	OR4M2	GAL3ST2	NAV3
LOC100294033	OR52N2	GALNT11	NBPF6
LOC100294341	OR56B4	GALNT16	NCF1
LOC100505549	OR5K4	GALNT18	NCS1
LOC100996707	OR6B2	GALNT2	NCSTN
LOC100996713	OR6C70	GALP	NDOR1
LOC100996758	OR6C74	GANAB	NDRG4
LOC101059915	OR7C2	GAS2L1	NDUFS5
LOC101060341	OR8K3	GAS6	NEFL
LOC101060521	OR9A4	GATA6	NEU2
LOC101060604	OSER1	GBP2	NFYA
LOC101927375	OTP	GCM2	NGB
LOC101927562	OTUD6B	GCNT1	NHS
LOC101927859	PADI1	GCNT3	NIPBL
LOC101927989	PAK1IP1	GCSH	NKAPL
LOC101928058	PALM	GDAP1	NKG7
LOC101928268	PALM2-AKAP2	GDE1	NOG
LOC101928291	PALMD	GDF5	NOTCH1

LOC101928380	PAM	GEMIN7	NOTCH2
LOC101928764	PAN2	Gen1	NPC1
LOC101929065	PAOX	GGT2	NR5A2
LOC101929070	PAQR6	GHSR	NRD1
LOC101929072	PARP10	GIMD1	NSUN7
LOC101929264	PARP16	GJA8	NT5C1A
LOC101929578	PARP6	GJB1	NT5DC4
LOC101929702	PBK	GKN2	NTSR2
LOC101929725	PCDH1	GLA	NUDT1
LOC101929748	PCDH19	GLIPR1L1	NUDT7
LOC101929936	PCDHA1	GLIS2	NUP210
LOC101930318	PCDHGA1	GLP2R	NXN
LOC101930355	PCDHGA11	GLT1D1	OAZ2
LOC101930512	PCDHGB4	GLTSCR1L	ODF3L1
LOC101930546	PCDHGC4	GLUD2	OFD1
LOC101930637	PCGF2	GLYCTK	OGFRL1
LOC389831	PCTP	GMEB2	OGN
LOC441239	PDCD1	GMIP	OLA1
LOC646862	PDE1A	GNA11	OLFM4
LPL	PDE4A	GNA12	OLFML1
LRFN1	PDHA1	GNAI1	OPN1SW
LRP10	PDP1	GNB4	OR10AD1
LRP4	PDXDC1	GNGT2	OR10G3
LRRC39	PEX11G	GNL1	OR10G4
LRRC4	PHF2	GOLGA2	OR11A1
LRRC48	PHIP	GOLGA4	OR12D3
LRRC58	PHKA1	GOLGA6L1	OR14I1
LRRC8D	PHKG1	GOLGA6L3	OR1F1
LRRC8E	PIP5KL1	GOLGA6L9	OR2D2
LRRN2	PITPNB	GOLGA8M	OR2F2
LSG1	PJA1	GON4L	OR2T1
LSM5	PKD1	GOT1L1	OR2T8
LST1	PKD1L2	GPA33	OR4K2
LTBP2	PKHD1	GPATCH11	OR4L1
LTK	PKN1	GPATCH2L	OR4M2

LY6E	PLA2G4E	GPBP1	OR4N4
LYG1	PLCB2	Gpn1	OR4P4
LYVE1	PLCB4	GPR116	OR5AS1
LYZ	PLEKHA4	GPR133	OR5AU1
LZTS1	PLEKHF2	GPR137C	OR5K1
MACC1	PLIN1	GPR149	OR5M9
MACF1	PLK2	GPR156	OR6C6
MAFG	PML	GPR158	OR6M1
MAGEA1	PMM2	GPR162	OR8B2
MAGEB1	PNLDC1	GPR88	OR8D1
MAGEC3	PNMA1	GPRASP1	OS9
MAL2	PNOC	GPSM3	OSBP
MAMDC2	PNPLA5	GRAMD2	OSGEPL1
MAMDC4	POC5	GRB7	OSM
MANEA	POFUT1	GRIA4	OSTM1
MANSC1	POLR2K	GRM2	OTOF
MAP1B	POM121L2	GRN	OTOP2
MAP4K3	PPFIA2	GRXCR1	OVCA2
MAP7	PPIAL4D	GRXCR2	P2RY8
MAPK15	PPM1J	GSDMA	P4HA1
MAPK3	PPP1R14D	GSG1	PA2G4
MAS1	PPP1R16B	GSG1L	PADI1
MBD3	PPP2R2A	GSG2	PAFAH1B2
MBLAC2	PPP2R5D	GSTO1	PALM
MBOAT4	PPRC1	GTF2E1	PAOX
MCEE	PQBP1	GTF2H3	PAPL
MCM2	PRAME	GTF2H4	PAPPA2
MCM5	PRC1	GTF3A	PARL
MCMBP	PRDM15	GUCD1	PATE2
MCOLN2	PREPL	GUCY2D	PATL1
MDK	PRIMA1	GYPC	PAX7
MECOM	PRNP	GYS1	PCBP3
MEGF10	PRODH	H1FNT	PCBP4
METRNL	PRR15	H1FX	PCDH1
MFAP2	PRSS21	HADHA	PCDHA3

MFI2	PRSS45	HADHB	PCDHB11
MICA	Prss53	HAO2	PCDHB12
MIEN1	PRSS56	HAUS6	PCDHGC4
MILR1	PSAP	HBD	PCSK7
MINK1	PSAPL1	HBEGF	PCTP
MIR205HG	PSMA5	Hbq1	PCYOX1
MLLT1	PTDSS1	HEATR4	PDCD2L
MME	PTGES3	HECTD3	PDCD4
MMRN2	PTGES3L	HELZ2	PDE6H
MNDA	PTGIS	HEMGN	PDHX
MOGAT2	PTGR2	HES4	PDK4
MPO	PTP4A1	HES5	PDP2
MROH5	PTP4A2	HGD	PDSS1
MRPL39	Ptpmt1	HGFAC	PDXDC1
MRPS26	PTPN12	HHLA3	PDZD3
MRPS30	PTPN18	HIF1AN	PET117
MRPS36	PTPRZ1	HIF3A	PGK1
MRPS6	PTRH2	HIGD1B	PGK2
Ms4a15	PTRHD1	HIP1R	PGM2
MS4A5	PVRL2	HIST1H2AG	PGP
MS4A6E	PXK	HIST1H2BK	PHF14
MSANTD1	RAB23	HIVEP2	PHF21A
MSH6	RAB3GAP2	HK2	PHKA1
MTCP1	RAB3IL1	HKDC1	PHOX2A
MTERFD1	RABGGTB	HMGCL	PHYHIP
MTERFD2	RAD51	HMGN4	PIANP
MTG2	RAD54L	HN1L	PIGW
MTHFD2L	RAD54L2	HNF1B	PIK3C2A
MTNR1B	RAET1E	HNF4G	PITPNA
MTR	RALGPS1	HNRNPA3	PKN1
MTRF1L	RANBP1	HNRNPCP5	Plac9
MTRNR2L10	RANBP17	HOXA1	PLAGL1
MTUS1	RANBP6	HOXA13	PLCG2
MUTYH	RAP2B	HOXB4	PLEKHA1
MX1	RAPSN	HOXC5	PLEKHA4

MXRA8	RARA	HOXC6	PLEKHF1
MYBL1	RASGRP3	HOXD9	PLEKHO1
MYH10	RAVER1	HPSE2	PLEKHS1
MYH3	RBM45	Hrct1	PLOD2
MYH9	RBMY1J	HRH2	PLS3
MYL6	RBP1	HRNR	PLXNA2
MYL7	REEP4	HS2ST1	PML
MYLPF	REM1	HSD11B1	PMP2
MYO1B	RERE	HSF2BP	PNLIPRP3
MYT1	RESP18	HSPA1B	POLD3
NAA11	REST	HSPBP1	POLD4
NAA38	RFPL4AL1	HTR2A	POLR2F
NADK2	RFT1	HUS1	POLR2J2
NAGS	RGS21	HVCN1	POU2F3
NAP1L1	RGS4	ICAM3	POU3F2
NAPRT1	RHOC	ICAM4	PPARG
NAT16	RHPN2	ICOSLG	PPL
NAT8B	RIPK1	ICT1	PPP1R14A
NBPF14	RNF123	IDNK	PPP1R35
NBPF19	RNF125	IDS	PPT2
NCALD	RNF180	IER2	PRADC1
NCBP2	RNF219	IFI27L1	PRAMEF10
NCCRP1	RNF26	IFNA10	PRAMEF6
NCEH1	RNF39	IFNA2	PRC1
NCR3	RNF7	IFNL4	PRCP
NDFIP2	RNMTL1	IFNW1	PRKAG3
NDST2	ROBO3	IFRD1	PRKD3
NDUFA4L2	ROM1	IFT20	PRMT9
NDUFB11	ROPN1L	IGFL1	PROK1
NDUFB5	RPUSD1	IGFN1	PRPH2
NDUFS3	RPUSD4	IGJ	PRRC1
NDUFV3	RRAS	IGSF23	Prrt4
NECAB2	RRM2B	IKZF1	PRSS57
NECAP2	RSPH10B	IL15RA	Prss58
NEK4	RSPH10B2	IL21	PSAT1

NEK9	RSPH4A	IL2RA	PSME4
NELFE	RUNX1T1	IL32	PSPH
NEMF	S100A11	IL4	PTBP1
NEU1	S100A14	IL5RA	PTCH1
NFATC2IP	S100A4	IL6ST	PTDSS1
NGDN	S1PR5	IMPAD1	PTDSS2
NHLRC4	SAC3D1	INADL	PTGES3
NIN	SAG	ING1	PTGIS
NKAIN2	SC5D	ING2	PTN
NKAIN3	SCAMP2	ING3	PTPLAD1
NKX2-2	SCAMP5	INMT	PTPN13
NLGN3	SCARA5	INPP5A	PTPN22
NLK	SCFD2	INPP5J	PTPRCAP
NLRP2	SCN7A	INTS1	PTPRG
NLRP7	SCO1	INTS10	PTPRR
NNAT	SDC3	INTS3	PUM2
NOB1	SDHC	INTS9	PYCR2
NOL7	SDPR	INTU	QPCT
NOMO2	SEC24A	IPO5	QTRT1
NOXA1	SEC31B	IPO7	RAB29
NPAP1	SECISBP2	IQCB1	RAB30
NPAS1	SELPLG	IQCF1	
NPBWR2	SENP2	IQGAP3	RAB34
NPM3	SEPT8	IRF2BP2	RAB9A
	SEPW1	IRF2BPL	RABGEF1
NPPB	SERPINB10	ISX	RAD23A
NPTX1	SESN2	ISY1	RAD50
NPY2R	SET	ITGA1	RARRES2
NR1H4	SETD7	ITGA4	RARS2
NR2C2	SFN	ITGA7	RASAL3
NR5A1	SFRP5	ITGA8	RASD1
NRF1	SFTA2	ITGB1BP2	RASL11A
NRG3	SFTPC	ITM2B	RB1CC1
NRIP2	SFXN3	ITPR1	RBM42
NRTN	SFXN4	Izumo2	RBM8A

NRXN2	SGPP1	JADE2	RBP3
NSG1	SH2B3	JAM3	RCE1
NSL1	SH3BGRL2	KATNA1	RCN2
NTF3	SH3PXD2B	KATNAL1	REEP5
NUDT12	SHFM1	KCMF1	REL
NUMB	SHISA4	KCNA1	REPIN1
NUP62CL	SHOC2	KCNA5	RET
NUPL1	SHPRH	KCNAB3	RGCC
NUSAP1	SHROOM3	KCNC3	RGL2
NVL	SIDT1	KCNG3	RGS17
NWD1	SIGLEC8	KCNJ16	RGS18
NXPH3	SIK3	KCNK3	RGS6
NYNRIN	SIKE1	KCNMB3	RHOBTB3
OAS1	SIN3A	KCNMB4	RHOD
OASL	SIRT5	KCNN2	RHOV
OAT	SIX6	KCTD21	RIC8A
OCM	SKI	KCTD5	RNASE2
ODAM	SLC10A1	KDM1B	RNASE6
OMA1	SLC10A5	KDM8	RNASEK
OR10A3	SLC12A4	KHDC1L	RND1
OR10A6	SLC12A7	KHK	RNF141
OR10H1	SLC16A4	KIAA0100	RNF145
OR10V1	SLC17A1	KIAA0754	RNF150
OR1G1	SLC22A24	KIAA0895	RNF186
OR1Q1	SLC22A7	KIAA1045	RNF213
OR2AG2	SLC25A40	KIAA1210	RNF7
OR2C1	SLC25A51	KIAA1244	RNPEP
OR2F2	SLC27A3	KIAA1257	ROCK2
OR2M3	SLC29A1	KIAA1328	RPA4
OR2W1	SLC2A12	KIAA1377	RPGR
OR4C16	SLC2A14	KIAA1429	RPL8
OR4C6	SLC2A2	KIAA1456	RPP25L
OR4D9	SLC34A1	KIAA1841	RPS4Y2
OR4F15	SLC35B1	KIF12	RRP1
OR4F17	SLC36A3	KIF13A	Rrp7a

OR4K13	SLC39A12	KIF18B	RSAD1
OR4K14	SLC39A2	KIF20B	RSPH1
OR4K5	SLC43A2	KIF26A	RSPO2
OR4M2	Slc46a2	KIF27	RTCB
OR4N2	SLC4A7	KIF28P	RTN4
OR51B5	SLC6A5	KIF3B	RUNDC1
OR52B4	SLC6A8	KIF5C	S100A1
OR52B6	SLC7A13	KLC3	S100Z
OR52K2	SLC9A5	KLF1	S1PR2
OR52L1	SLC9B1	KLF6	S1PR5
OR52W1	SLIT2	KLHDC9	SACM1L
OR5AP2	SLMAP	KLHL13	SAMD10
OR5AS1	SLPI	KLHL28	SCAMP2
OR5B12	SMAD2	KLHL36	SCAP
OR5D18	SMAD3	KLHL4	SCARB2
OR5M1	SMARCA2	KLHL42	SCFD2
OR6C1	SMARCD1	KLHL6	SCRN3
OR6C75	SMARCD2	KLK15	SDF2L1
OR6F1	SMC3	KLK7	SDK1
OR6K2	SMIM23	KLRC1	SEH1L
OR6K3	SMPDL3A	KLRC4-KLRK1	SENP2
OR7A10	SMTNL1	KLRF1	SENP8
OR7A5	SMYD5	KLRF2	SEPT1
OR7G1	SNAP23	KPNA3	SERINC1
OR8G2	SNAP25	KPNA6	SERPINA1
OR9G1	Snrnp25	KPRP	SERPINA6
OR9Q2	SNTG2	KRBA2	SERPINE2
ORC5	SNUPN	KRT10	SERPINH1
ORM2	SOCS1	KRT27	SETD9
OSCP1	SOCS7	KRT34	SF3A2
OTOF	SOS2	KRT6A	SGSM1
OXSR1	SP110	KRT78	SH2D1A
P2RY14	SPATA5	KRT8	SH2D6
P4HB	SPDL1	KRT84	SHC4
P4HTM	SPI1	KRTAP10-7	SHKBP1

Pabpc1l	SPINK9	KRTAP10-8	SIGIRR
Pabpc1l2b	SPRN	KRTAP12-2	Siglec15
PABPC4L	SPTLC3	KRTAP12-4	SKP2
PAFAH2	SPX	KRTAP2-3	SLAMF8
PAGE1	SRRD	Krtap21-1	SLC10A7
PAGE2B	SRSF8	Krtdap	SLC12A3
PAK4	ST14	KTI12	SLC15A4
PALLD	ST20-MTHFS	LAMA1	SLC18A3
PALMD	ST5	LAMA2	SLC1A7
PAM	STARD13	LAMA5	SLC22A2
PANX1	STK17A	LAMP2	SLC22A23
PAPL	STK17B	LAMP5	SLC22A6
PAPOLB	STK38	LAT	SLC22A9
PAQR3	STOML1	LAT2	SLC24A3
PARK2	STX10	LBX1	SLC25A2
PARP3	STXBP6	LCE2B	SLC25A22
PARS2	SUB1	LCN8	SLC25A25
PATE1	SUCLA2	LCNL1	SLC25A33
PATE3	SUDS3	LCTL	SLC26A11
Pbrm1	SUMO4	LDB1	SLC2A11
PBX1	SUSD1	LDB2	SLC31A1
PCCB	SYCE1L	LDHD	SLC35A3
PCDH19	Syce2	LDOC1L	SLC35B4
PCDHA13	Sycp2l	LEFTY1	SLC35E2
PCYT1A	SYK	LEKR1	SLC35E2B
PDCD4	SYNJ2	LETM1	SLC35F5
PDCD6IP	SYT14	LGALS7	SLC39A13
PDCD7	SYTL1	LGALS7B	SLC4A7
PDE10A	TAC4	LHX4	SLC5A4
PDE1A	TACC2	LHX5	SLC6A5
PDHB	TAF1L	LIAS	SLC6A6
PDLIM2	TAF9B	LILRB2	SLC9B2
PDP1	TAOK1	LIMCH1	SMIM11
PDSS2	TARSL2	LIN9	SMIM4
PDZRN3	TAS2R16	LINGO4	SMIM9

PEG10	TBC1D10C	LIPA	SMN2
PERP	Tbck	LITAF	SMPDL3A
PEX11B	TBX19	Llph	SMPX
PEX11G	TCEA3	LMAN2	SNAI1
PEX5L	TCEAL1	LMAN2L	SNRPE
PEX7	TCERG1L	LMBR1	SNTB1
PFAS	TCF12	LMF2	SNUPN
PFDN6	TCN2	LMOD3	SNX1
PFKFB1	TCP11L1	LMX1B	SNX25
PGC	TCP11X1	LNP1	SNX4
Phax	TCTEX1D1	LNX2	SOCS2
PHKA1	Tctex1d2	LOC100129083	SOCS5
PHLPP1	TDGF1	LOC100129361	SORCS3
PI15	TEAD1	LOC100130357	SOX18
PI16	TEDDM1	LOC100131107	SOX5
PI3	TEKT5	LOC100132731	SPATA2
PI4K2B	TENM3	LOC100506127	SPATA31A6
PIGC	TET2	LOC100506248	SPATA31A7
PIGT	TEX26	LOC100506922	SPIN2B
PIGW	TEX28	LOC100507462	SPINK5
PIGY	TGIF2LY	LOC100652824	SPON2
PIH1D1	THBS4	LOC100996318	SPRED1
PIK3C2B	THNSL1	LOC101060399	SPRY4
PIK3C3	TIMP3	LOC101060580	SPSB2
PIP5K1C	TJP2	LOC101060581	SPTBN4
PITPNB	TLK2	LOC101927160	SPTY2D1
PITX1	TLR2	LOC101927375	SRCAP
PIWIL4	TLR6	LOC101927594	SRCIN1
PJA2	TM2D2	LOC101927662	SRI
PKD1L2	TMC6	LOC101927789	SRSF8
PKLR	TMEM151B	LOC101928006	SSPN
PKN3	TMEM165	LOC101928044	SST
PKP1	TMEM170A	LOC101928093	ST7
PLA2G1B	TMEM177	LOC101928356	STAB1
PLA2G2E	TMEM198	LOC101928548	STARD7

PLA2G4F	TMEM206	LOC101928558	STAT5A
PLAGL2	TMEM216	LOC101928892	STC1
PLBD1	Tmem237	LOC101928991	STEAP4
PLCB4	TMEM255A	LOC101929571	STH
PLEKHA3	TMEM30A	LOC101929578	STK17B
PLEKHG7	TMEM51	LOC101929587	STK32A
PLEKHH2	TMEM56	LOC101929627	STK33
PLEKHO1	TMEM67	LOC101929766	STK38
PLP1	TMEM9	LOC101929792	STOM
PLXNA1	TMPRSS11F	LOC101929805	STRIP2
PMM1	TMPRSS2	LOC101929829	Stx1b
PMP22	TMPRSS6	LOC101929936	STYK1
PNKP	TMTC1	LOC101929983	SUDS3
PNLIPRP2	TNFAIP1	LOC101929989	SULT4A1
PODXL	TNFAIP6	LOC101929991	SUMO4
POLA2	TNFRSF11B	LOC101930098	SUN2
POLE3	TNFRSF13C	LOC101930295	SUPT20HL2
POLE4	TNFRSF6B	LOC101930355	SUSD5
POLL	TNFSF9	LOC101930479	SVIL
POLR2K	TNIP1	LOC199882	SYBU
POLR3G	TNKS2	LOC285500	SYN3
POMGNT1	TOM1L2	LOC388813	SYNJ2BP-COX16
POP1	TOMM7	LOC391322	TAAR9
POTEA	TOP1	LOC441155	TACSTD2
POU5F2	TOP2A	LOC441239	TAF6L
PPARA	TOR1B	LOC642441	TAGLN3
PPARD	TP53TG3D	LOC646730	TARBP2
PPIL4	TPI1	LOC646862	TARSL2
PPIP5K2	TPSD1	LOXL1	TAX1BP1
PPM1G	TRAFD1	LPA	TBC1D29
PPM1J	TRAM1	LPAR4	TBX19
PPM1N	TRIM32	LPCAT4	TBX2
PPP1R12A	TRIM39-RPP21	LPHN1	Tchh
PPP1R14D	TRIM42	LPHN3	TCP1
PPP1R17	TRIM46	LPO	TCTEX1D4

PPP1R2	TRIM54	LPPR2	TDP2
PPP1R7	TRIM62	LPPR3	TENM2
PPP2R2B	TRIM67	LRP11	TESK1
PPP2R3C	TRIML1	LRP2BP	TEX101
PPP3CB	TRIO	LRRC23	TEX11
PRAME	TRPC5OS	LRRC24	TGM4
PRAMEF25	TRPM2	LRRC27	THAP2
PRAP1	TRPM4	LRRC38	THEM5
PRDM15	TRPT1	LRRC39	THSD7A
PRDM8	TRPV1	LRRC42	TIMM10B
PREX1	TSN	LRRC57	TINF2
PRG4	TSPY3	LRRC6	TLK1
PRKCD	TSR3	LRRC70	TMEM114
PRMT9	TTC17	LRRC8B	TMEM144
PROZ	TTC34	LRRC8E	TMEM156
PRPF4	Ttc39a	LRRIQ1	TMEM165
PRR11	TTC4	LRRTM2	TMEM175
PRR32	Tti2	LSM1	TMEM178A
PRRG4	TTLL11	LSMEM2	TMEM183B
PRSS57	TUBGCP5	LTBP3	TMEM184C
PRTN3	TULP4	LYPD5	Tmem185b
PSMD10	TVP23B	LYRM9	TMEM253
PSMD12	TVP23C	LYZL1	TMEM33
PSMD9	TYMP	M6PR	TMEM41B
PSME4	TYROBP	MADD	TMEM45B
PSMG2	UBA52	MAG	TMEM63B
PSMG3	UBASH3A	MAGEA11	TMPRSS11F
Pstk	UBE2D2	MAGEA9	TMTC2
PTGES3L	UBL4B	MAGEB1	TMUB1
PTPN13	UBQLN4	Mageb17	TNFRSF1B
PTPN20B	UGT2B17	Magt1	TNFRSF21
PTPN22	ULBP1	MAK	TNFSF12
PTPN3	UNCX	MAMDC4	TNPO2
PTRH1	UPP2	MAN2A1	TOM1L1
PVALB	UQCRC2	MANSC4	Tomm20l

PVRL2	URI1	MAP1LC3B	TOP1MT
QRICH2	USF1	MAP1LC3BP1	Tox2
RAB11FIP3	USP17L15	MAP2K2	TPBGL
RAB12	USP20	MAP3K11	TPP1
RAB15	USP41	MAP3K6	TPSG1
RAB26	UXT	MAP4K5	TRAPPC2L
RAB33A	VAC14	MAPK1	TRERF1
RABEP2	VAMP1	MAPK15	TREX1
RABL2A	VAMP3	MAPK8IP1	TRIB1
RAD23A	VAMP8	MAPK8IP2	TRIB3
RAD9A	VBP1	MARCKSL1	TRIM16L
RAG1	VCAM1	MARCO	TRIM39-RPP21
RAP1GDS1	VCY	MARK4	TRIM50
RAPGEF5	VIPR2	MAS1	TRIM63
RASA2	VN1R2	MAST3	TRMT6
RASSF1	VNN1	MBD1	TRUB1
RASSF10	VPS26A	MBLAC2	TSFM
RASSF4	VPS37B	MCFD2	TSN
RBBP5	VPS4A	MCM10	TSPAN2
RBM34	VRK3	MCM2	TSPAN7
RBP4	VSTM2B	MCPH1	TSSC4
RCN2	VWA2	MED18	TSSK2
REG1A	VWA8	MED20	TSTA3
REM2	WAC	MEGF11	TSTD3
REP15	WDFY1	MEPCE	TTBK1
REXO1L1P	WDR45B	MET	TTBK2
RFESD	WDR53	METTL17	TTC13
RFPL2	WHSC1L1	METTL21B	TTC21B
RFPL4AL1	WISP3	METTL4	TTC7B
RGAG1	WRB	METTL9	TUBB2A
RGL2	WTAP	MEX3D	TUFM
RHBDL2	XPNPEP1	MFAP4	TULP2
RHOA	YBEY	MFAP5	TULP4
RHOF	ZAR1	MFI2	TVP23C-CDRT4
RHOV	ZBTB8B	MFNG	U2AF1L4

RHPN1	ZCCHC4	Mfsd10	U2AF2
RICTOR	Zdbf2	MFSD2B	UAP1L1
RILPL1	ZEB2	MFSD4	UBE2D2
RIPK2	ZFP36	MFSD6	UBE2QL1
RLTPR	ZNF131	MGAT3	UBE3A
RMND5A	ZNF148	MGAT5	UBR7
RNASE1	ZNF155	MIA3	UGT2B11
RNF133	ZNF177	MIB2	UHMK1
RNF150	ZNF185	MICAL1	ULK2
RNF169	ZNF200	MIEF1	UNC13D
RNF182	ZNF267	MIEF2	UNC79
RNF219	ZNF273	MILR1	UNK
RNF8	ZNF282	MIPEP	UPK1A
RNFT2	ZNF284	MISP	UPK3A
ROBO4	ZNF302	MKRN3	UQCRFS1
ROR2	ZNF324	MLIP	USB1
RORA	ZNF408	MLLT4	USF2
RPL36A-HNRNPH2	ZNF433	MLLT6	USP12
RPL39L	ZNF488	MLXIP	USP18
RPL3L	ZNF500	MMP10	USP22
RPL6	ZNF518B	MMP17	USP3
RPS12	ZNF606	MMP28	USP51
RPUSD1	ZNF665	MOGAT3	USP6
RSRC1	ZNF688	MORC2	USP9Y
RTFDC1	ZNF705D	MOXD1	UTP23
RTKN	ZNF727P	MPRIP	UTRN
RTL1	ZNF732	MRGPRD	VANGL1
RUFY3	ZNF737	MRGPRX1	VCPIP1
RXFP1	ZNF793	MRGPRX3	VDR
RXRA	ZNF80	MRGPRX4	VEGFC
RYR1	ZNF814	MROH2A	VIL1
S100A7	ZNF816-ZNF321P	MRPL14	VN1R5
S100B	ZNF845	MRPL36	VNN2
SAA4	ZNF880	MRPL4	VPS26A
SAFB	ZNF92	MRPL48	VPS52

SALL1	ZNRF2	MRPL49	VRK3
SARS2	ZSCAN12	MRPL51	VWA5B2
SBF2	ZSCAN23	MRPL53	WBP2
SBK1	ZSCAN4	MRRF	WBSCR27
SCAF11	ZWINT	MS4A1	WDTC1
SCAMP3		MS4A4A	WFDC6
SCML1		MS4A6A	WFIKKN2
SCML2		MS4A7	WIZ
SCN1B		MSC	WNT5A
SCRT2		MSH4	WRN
SDAD1		MSH5	XCL1
SDC4		MSI2	XKR3
SDF2		Msl2	XYLT2
SDHB		Msmp	YPEL4
SDHC		Mst4	YWHAG
SEC13		MTA1	ZBED3
SEPT1		MTAP	ZBTB14
SEPT8		MTERFD1	ZBTB2
SEPT14		MTG1	ZBTB41
SERINC4		MTHFS	ZBTB42
SERPINA6		MTMR4	ZBTB8B
SERPINB3		MTNR1B	Zcchc18
SERPINB5		MTR	ZCCHC3
SESN2		MTRNR2L3	ZDHHC1
SETDB2		MTRNR2L5	ZDHHC5
SFRP1		MTRNR2L6	ZDHHC9
SGCB		MTURN	ZFP62
SGCE		MTX2	ZFYVE20
SGSH		MUC21	Zglp1
SH2D1B		MUC7	ZMIZ2
SH3BP2		MUM1L1	ZMPSTE24
SHANK1		MX1	ZNF132
SHBG		MXI1	ZNF208
SHCBP1		MYF6	ZNF233
SHD		MYH13	ZNF248

SIK2
SIRT1
SKIV2L2
SLAMF6
SLC10A2
SLC10A4
SLC12A1
SLC13A5
SLC1A3
SLC22A2
Slc22a20
SLC22A24
SLC25A46
SLC25A53
SLC26A5
SLC2A11
SLC30A1
SLC30A9
SLC34A2
SLC35B3
SLC35G3
SLC37A2
SLC37A3
SLC38A10
SLC38A11
SLC38A9
SLC39A8
SLC6A1
SLC7A11
SLC7A14
SLC7A3
SLC8A1
SLC9B1
SLCO5A1
SLTM

MYH14	ZNF263
MYL3	ZNF280B
MYO5B	ZNF324B
MYO5C	ZNF444
MYO7B	ZNF501
MYO9B	ZNF513
MYOD1	ZNF517
MYOM3	ZNF521
MYT1	ZNF576
MYZAP	ZNF578
NAA25	ZNF579
NAA60	ZNF582
NACC2	ZNF589
NAGPA	ZNF600
NALCN	ZNF623
NAMPT	ZNF684
NANOG	ZNF710
NANS	ZNF718
NAPEPLD	ZNF747
NAT1	ZNF791
NAT2	ZNF8
NAV3	ZNF891
NBPF3	ZNRD1
NCAM2	ZP1
NCF1	ZP2
NCKAP1L	ZPBP2
NCKAP5	ZSCAN2
NCSTN	
NDST4	
NDUFA10	
NDUFA4L2	
NDUFA7	
NDUFA9	
NDUFAF3	
NDUFAF6	

SMAD1
SMARCD2
SMARCD3
SMC2
SMCO4
SMG9
SMIM13
SMIM15
SMPX
SNAI1
SNAP23
SND1
SNED1
Snrnp70
SNX1
SNX14
SNX8
SOX13
SOX30
SOX8
SP2
SPACA4
SPAG17
SPARCL1
SPATA22
SPATA31D1
SPATA33
SPATS2L
SPCS3
SPDL1
SPERT
SPIB
SPIN1
SPINK7
Spns1

NDUFB3
NDUFS3
NDUFV1
NEUROD4
NEUROD6
NFKB2
NFU1
NHP2L1
NIPAL4
NIPSNAP3A
NKG7
NKIRAS2
NKX2-4
NLGN4X
NLRP11
NME2
NMRK2
NMS
NOL10
NOL11
NOL9
NOMO1
NOP10
NOS1
NOTO
NPHP1
NPL
NPM3
NPR1
NPY4R
NPY5R
NR5A2
NRDE2
NRP1
NRSN2

SPOCD1
SPTBN1
SPTSSA
SQLE
SRCIN1
SRP68
SRP72
SRPK3
SRRD
SRRM4
SRSF8
SSBP2
SSMEM1
STARD6
STAT5B
STK17B
STK19
STPG1
STRADA
STRIP2
STX1A
STXBP6
SUDS3
SUGCT
SULT1E1
SUN2
SUN3
SUOX
SYCE1
SYN1
SYNPR
SYPL1
SYS1
SYT12
SYT13

NSRP1
NSUN7
NT5C1A
NT5C1B
NT5M
NTF4
NTNG2
NUCB2
NUMA1
NUP133
NUP210
NUSAP1
NXF1
NXNL1
NXPH2
NXT2
OAZ1
OCA2
OCM
OCSTAMP
OFCC1
OLFML2A
OLIG3
OPLAH
OPN3
OPTN
OR11G2
OR11H4
OR13C8
OR13G1
OR13H1
OR1N1
OR2A1
OR2A5
OR2F2

SYTL2
TAAR9
TAB2
TAC1
Tarm1
TAS2R13
TAS2R60
TBC1D26
TBC1D4
TBCE
TBPL2
TEP1
TEX30
TFDP3
TFE3
TFEC
TGM3
THAP3
THBS2
THEMIS
TIMM17B
TIMM44
TIMM9
TLR5
TLR6
TLX2
TLX3
TM7SF2
TMCO2
TMCO4
Tmco5a
TMED10
TMED3
TMED7
TMEM147

OR2M4
OR2M7
OR2T29
OR2Z1
OR4A15
OR4C16
OR4E2
OR4F17
OR4K5
OR4S2
OR51A7
OR51B6
OR51I1
OR51M1
OR52H1
OR52K2
OR52N2
OR56A5
OR5AN1
OR5AS1
OR5B12
OR5M3
OR5M9
OR6C74
OR6M1
OR7D4
OR8D1
OR8G2
OR8J3
ORAI1
ORAI3
ORMDL1
OSBPL7
OTOP2
OTUB1

TMEM150B	OTUB2
TMEM165	OTUD5
TMEM17	OTUD6B
TMEM173	OTX2
TMEM191B	OXSR1
TMEM240	OXT
TMEM241	P2RX4
TMEM243	P2RX7
TMEM63C	P2RY8
TMEM91	P4HTM
TMEM98	PABPC4
TMOD2	PABPN1
TMPRSS12	PACSIN2
TMTC3	PAGE2B
TMX4	PAGE4
TNFAIP8L2-SCNM1	PAN2
TNFRSF13C	PAQR6
TNK1	PARD3
TOMM6	PARD3B
TOR3A	PARD6B
TP53BP2	PARL
TP53I11	PARVA
TP53TG3D	PASK
TP73	PATE1
TPD52	PATE3
TPD52L3	PBK
TPRKB	PBX2
TRABD2B	PBXIP1
TRADD	PCBD2
TRAF3IP2	PCBP2
TRAF6	PCDH11Y
Trappc10	PCDH12
TRAPPC9	PCDH18
TRHDE	PCDH19
TRIB1	PCDH9

TRIM10
TRIM28
TRIM32
TRIM34
TRIM38
TRIM45
TRIM54
TRIM60
TRIQK
TROAP
TRPM2
TRPS1
TRUB2
TSEN34
TSFM
TSHZ3
TSPAN2
TSPAN5
TSTA3
TTC18
TTC7A
Tti2
TULP4
TWF1
TWF2
TXNDC15
TYW1
Ubash3b
UBE2N
UBE2R2
UBE2V2
UBE3C
UBQLN2
UBQLNL
UBTFL1

PCDHA6
PCDHB14
PCDHGA10
PCDHGB1
PCDHGB3
PCDHGB5
PCIF1
PCSK1
PCSK5
PDCD1
PDCD4
PDCL2
PDDC1
PDE1B
PDE1C
PDE9A
PDIA5
PDXP
PDZD3
PDZD4
PDZD8
PECR
PENK
PER1
PET117
PEX3
PFKFB2
PGAM5
PGBD3
PHKG2
PHLDA3
PHYHIP
PI16
PIGR
PIGW

UCHL5	PIK3C2A
UNC119	PIK3CA
UPRT	PIK3IP1
UQCC2	PIK3R4
UQCRC1	PIK3R5
UQCRQ	PILRB
USF2	PINX1
USP1	PIPOX
USP37	PITPNB
USP51	PITPNM3
VAMP8	PIWIL1
VGF	PKD1L3
VIMP	PKDCC
VPREB1	PKIA
VPS11	PLAC1
VPS37C	PLB1
VPS39	PLCL2
VPS41	PLD6
VPS4B	PLEKHG4B
VPS51	PLEKHJ1
VPS72	PLIN4
VSTM2B	PLK3
VSTM2L	PLS1
VWA5B1	PLVAP
WAS	PLXNA2
WBP11	PNLIPRP2
WBP2	PNMA1
WBP4	PNMAL1
WBSCR28	POLD3
WDR17	POLN
Wdr38	POLR2C
WDR55	POLR2J2
WDR62	POLR3F
WDR64	POLR3G
WDR72	POLR3GL

WDR78
WDR96
WFDC8
WFIKKN2
WNT10B
XRCC4
YBX3
ZBED3
ZBTB20
ZBTB46
ZBTB7A
ZC3H3
ZCCHC10
ZDHHC15
ZDHHC2
ZDHHC3
ZFYVE20
ZGRF1
ZMAT2
ZMIZ2
ZMPSTE24
ZMYM2
ZNF132
ZNF19
ZNF20
ZNF202
ZNF215
ZNF230
ZNF233
ZNF263
ZNF300
ZNF311
ZNF418
ZNF436
ZNF445

POMC
POMZP3
PON1
PON3
POP5
POTEC
POTEI
PPARGC1B
PPFIA3
PPIAL4C
PPL
PPM1G
PPM1H
PPM1M
PPP1CB
PPP1R35
PPP1R9B
PPP2R5A
PPP3CA
PPP3R2
PPP4R1
PPP4R2
PPP6R1
Ppp6r2
PRAME
PRAMEF19
PRAMEF5
PRAMEF6
PRAMEF7
PRDM13
PRDM15
PRDM4
PRDM5
PRDM7
PRDX4

ZNF468
ZNF517
ZNF550
ZNF551
ZNF554
ZNF555
ZNF565
ZNF569
ZNF572
ZNF583
ZNF585B
ZNF606
ZNF607
ZNF615
ZNF658
ZNF665
ZNF682
ZNF701
ZNF727P
ZNF740
ZNF747
ZNF786
ZNF8
ZSCAN16
ZSCAN31
ZSCAN32
ZYX

PRIMPOL
PRKAG2
PRKCD
PRKCI
PRKD3
PRKG2
PRKX
PRMT8
PRNP
PROK2
PROM2
PROSC
PRPF38B
PRPH
PRPSAP2
PRR14L
PRR22
PRRC2C
PRRT3
PRSS46
PRSS50
PSD2
PSD3
PSD4
PSMA1
PSMB11
PSMC1
PSMC3
PSMF1
PTBP3
PTCH1
PTGDR2
PTGDS
PTK7
PTP4A1

Ptpmt1
PTPN14
PTPN18
PTPRH
PTPRK
PTPRN
PTRH2
PTTG1IP
PTTG2
PUM2
PUS10
PUSL1
PVR
PYROXD2
QRFPR
R3HDM2
RAB17
RAB20
RAB21
RAB33A
RAB39B
RAB3B
RAB9A
RAC1
RAC2
RAD23B
RAD54L
RANBP2
RANBP6
RANBP9
RAPGEF5
RASA1
RASAL3
RASGEF1A
RASGRP1

RASL10A
RASSF4
RBKS
RBL1
RBM43
RBM44
RBM6
RBMY1J
RBP3
RBP4
RBPMS
RCBTB1
RCCD1
RCN2
RCN3
REEP3
REG3G
RELN
REPS2
RER1
RERGL
RETNLB
REV3L
RFPL4B
RFT1
RFTN1
RFX5
RFXANK
RGMB
RGPD2
RGS12
RGS13
RGS5
RHOB
RHOH

RIC3
RILPL2
RIPK2
Ripply1
RLN1
RLN3
RNASE1
RNASE12
RNASE6
RNASE7
RNASE9
RND2
RNF113A
RNF125
RNF133
RNF157
RNF181
RNF212
Rnf217
RNF4
RNFT2
Rnls
ROR1
RPA4
RPL10
RPL10L
Rpl18a
RPL23A
RPL28
RPL9
RPP40
RPS18
RPS4X
RPS6KA1
RPS6KB1

RPS6KC1
RPTOR
RRH
RRP36
Rrp7a
RSPO3
RSRC1
RTFDC1
RTN4RL1
RUNX3
RUSC1
RYBP
S100A10
S100A12
S100A2
S100A7
S100A7A
S100PBP
SAA2-SAA4
SAFB2
SAMD11
SAMD12
SAMSN1
SAPCD2
SAR1B
SASS6
SBF2
SBK3
SCAF8
SCAI
SCARA3
SCD5
SCGB3A2
SCGN
SCN2A

SCN3B
SCNN1D
SCOC
SCRT1
SCUBE2
SDR16C5
Sec16a
SEC61A2
SEH1L
SELPLG
SEMA3G
SEMA4F
SEMA4G
SEMA5A
SEMA7A
SEMG2
SENP8
SEPT1
SEPT8
SEPW1
SERBP1
SERHL2
SERPINA6
SERPINB11
SERPINB13
SERPINB2
SERPINE3
SESN2
SETD6
SETD8
SEZ6L
SEZ6L2
SFI1
SFT2D1
SFTPC

SFTPD
SFXN2
SGK2
SGSM1
SH2D4A
SH2D7
SH3GL3
SH3KBP1
SH3PXD2B
SH3RF3
SH3YL1
SHANK3
SHMT2
SHOX
Siglec15
SIN3B
SIPA1L3
SIRT7
SIT1
SKI
SLAMF6
SLC10A5
SLC11A1
SLC12A4
SLC13A5
SLC17A6
SLC17A8
SLC18A2
SLC19A2
SLC1A1
SLC1A5
SLC22A2
SLC22A6
SLC23A3
SLC25A11

SLC25A12
SLC25A16
SLC25A22
SLC25A23
SLC25A24
SLC25A26
SLC25A28
SLC25A46
SLC25A47
SLC27A6
SLC28A1
SLC29A3
SLC2A13
SLC2A14
SLC2A3
SLC30A1
SLC30A8
SLC32A1
SLC33A1
SLC35A2
SLC35D1
SLC35D3
SLC35G1
SLC35G3
SLC35G6
SLC36A2
SLC37A2
SLC38A5
SLC38A8
SLC39A12
SLC39A8
SLC44A1
SLC44A4
SLC45A2
SLC46A1

SLC4A1
SLC4A9
SLC5A4
SLC7A13
SLC7A6
SLC9A4
SLCO1A2
SLCO1B1
SLCO1B7
SLCO3A1
SLITRK2
SLITRK4
SLMAP
SMAD7
SMARCA5
SMARCAD1
SMARCC1
SMARCD2
SMCO2
SMCO4
SMG1
SMIM14
SMIM15
SMIM3
SMIM7
SMPD1
SMR3A
SMR3B
SMYD2
SNAI3
SNRNP27
SNTG2
SNURF
SOCS7
SORBS2

SOX10
SOX11
SP5
SPAG5
SPAST
SPATA21
SPATA31A6
SPATA8
SPDYE4
Spg11
SPG21
SPIDR
SPINK2
SPINK6
SPRY4
SPSB2
SRGAP2D
SRGAP3
SRPR
Srrm3
SRSF3
SRSF8
SRXN1
SSBP2
SSBP4
SSR2
SSX2
ST6GALNAC1
ST6GALNAC5
ST6GALNAC6
STAG1
STARD10
STARD4
STAT1
STAT4

STAT5A
STEAP3
STK10
STK11
STK17B
STK25
STK36
STK38L
STK4
STMN3
STOML2
STRA13
STX17
STXBP5
SUDS3
SUGP2
SULT1A2
SULT1A3
SULT1C2
SULT2A1
SULT4A1
SUMF1
SUN1
SURF1
SURF6
SVIL
SVIP
SYCP2
SYDE2
SYF2
SYN1
SYNE1
SYNGAP1
SYNJ2
SYT13

SYT2
SYT6
TAAR1
TAAR6
Tab1
TACR2
TAF10
TAF12
TAF7
TAF7L
TAOK2
TARDBP
TARP
TAS2R10
TAS2R46
TBC1D16
TBC1D2
TBC1D21
TBC1D5
TBCB
TBX18
TBX5
TC2N
TCEAL6
TCEANC2
TCEB3
TCF24
TCF7L2
TCN1
TCP11L2
TCTA
Tctex1d2
TDG
TDP2
TDRD1

TDRD5
TEAD2
TEF
TENM3
TERT
TES
TESC
TESK1
TESK2
TFB2M
TFDP3
TFPT
TGDS
TGIF1
TGM3
TGM5
TGM7
TGS1
THAP10
THAP2
THAP7
THBS2
THBS3
THEMIS
THG1L
THOC6
THRAP3
THRSP
THSD7B
TIAF1
TIFA
TIMM17A
TIMM9
TINAG
TJP1

TK2
TLE4
TLR5
TLX2
TM2D1
TMC1
TMCO4
TMED9
TMEFF1
TMEM100
TMEM102
TMEM107
TMEM109
TMEM11
TMEM117
TMEM129
TMEM144
TMEM158
TMEM161A
TMEM165
TMEM167A
TMEM183A
TMEM189-UBE2V1
TMEM200C
TMEM210
TMEM211
TMEM214
TMEM216
TMEM229A
TMEM231
Tmem237
TMEM242
TMEM247
TMEM249
TMEM251

TMEM255A
TMEM38A
TMEM38B
TMEM45B
TMEM52B
TMEM54
TMEM56
TMEM79
TMPRSS11B
TMPRSS2
TMPRSS4
TMSB10
TMX3
TNF
TNFAIP1
TNFRSF21
TNFRSF6B
TNFSF11
TNFSF15
TNIK
TNKS2
TNNC1
TNNI2
TNNI3K
TOB1
TOMM7
TOMM70A
TOPORS
TOR1AIP1
TOR3A
TP53I11
TP63
TPPP
TPTE
TRABD2B

TRAPPC12
TRAPPC13
TRDMT1
TRDN
TRIM17
TRIM21
TRIM31
TRIM32
TRIM37
TRIM43B
TRIM49D1
TRIM56
TRIM59
TRIM62
TRIM68
TRIM71
TRIML1
TRIOBP
TRMT13
TRMT2A
TRMT2B
TRMT6
TRPM7
TRPV3
TRPV4
TRUB1
TSC22D3
TSGA10IP
TSPAN12
TSPAN17
TSPAN18
TSPAN2
TSPAN31
TSPAN4
TSR2

TSSC4
TTBK2
TTC23
Ttc23l
TTC30A
TTC32
TTLL12
TTLL5
TTR
TUBGCP6
TULP2
TUSC3
TWF2
TXNDC11
TXNDC17
TXNRD1
TXNRD3
TYROBP
TYRP1
UBB
UBE2D3
UBE2E2
UBE2NL
UBE2V2
UBE3B
UBE3C
UBLCP1
UBOX5
UBR7
UBTD1
UGT1A1
UGT1A9
UGT2B10
ULK2
ULK3

UNC13D
UNC5B
UNC5D
UNCX
UPF2
UPF3A
UPK1A
UQCC1
USH1C
USP14
USP25
USP28
USP33
USP40
USP43
USP7
UTP6
UTS2B
UXS1
VAMP7
VAMP8
VAT1
VCL
VCY
VCY1B
VEGFA
VIPAS39
VIPR2
VKORC1
VMO1
VPRBP
VPREB3
VPS36
VPS45
VPS53

VRTN
VTI1B
VWA8
VWF
WASF3
WBSCR16
WBSCR28
WDR31
WDR53
WDR6
WDR72
WDR75
WDR81
WDR87
WDSUB1
Wfdc2
WFIKKN1
WIZ
WNT3A
WRB
WWC2
XCR1
XIRP1
XKR6
XKRX
XPA
XPO5
XRCC2
YIF1A
YIPF5
YKT6
ZAK
ZBED3
ZBTB20
ZBTB32

ZBTB37
ZC3H12C
Zc3h18
ZC3HC1
ZCCHC2
ZCRB1
ZDHHC8
ZFAND1
ZFP14
ZFP36
ZFY
ZFYVE16
ZFYVE19
ZIC5
ZIK1
ZKSCAN2
ZMPSTE24
ZMYND10
ZMYND11
ZMYND15
ZMYND19
ZNF146
ZNF16
ZNF165
ZNF17
ZNF174
ZNF19
ZNF200
ZNF22
ZNF248
ZNF25
ZNF250
ZNF276
ZNF292
ZNF300

ZNF304
ZNF319
ZNF32
ZNF324B
ZNF331
ZNF34
ZNF37A
ZNF385A
ZNF396
ZNF410
ZNF417
ZNF419
ZNF443
ZNF461
ZNF485
ZNF488
ZNF518A
ZNF519
ZNF526
ZNF555
ZNF557
ZNF593
ZNF600
ZNF628
ZNF668
ZNF678
ZNF705A
ZNF716
ZNF720
ZNF746
ZNF747
ZNF761
ZNF8
ZNF808
ZNF879

ZNF93
ZP2
ZRANB3
ZSCAN12
ZSCAN16
ZSCAN20
ZSCAN30
ZSCAN9
ZSWIM8
ZXDB

Supplementary Table S4: Unique/multiple shRNA hits

Control hit	# shRNA	HU hit	# shRNA	DOX hit	# shRNA	HU + DOX hit	# shRNA
ACTB	4	ACTB	3	CENPE	6	OCM	5
FGFR1	4	CTNNB1	3	CIART	6	PFDN4	5
H3F3B	4	EEF2	3	H3F3B	6	PPIB	5
PLCB4	4	GAGE12J	3	POLR1A	6	ACTB	4
ACSM2A	3	GPBP1	3	YEATS4	6	ADAMTSL1	4
CCDC121	3	KRTAP10-7	3	AURKA	5	BRSK2	4
CDK1	3	Ms4a15	3	FGFR1	5	C5orf49	4
CEP250	3	NAA30	3	LAMB1	5	Eif6	4
EREG	3	PARP10	3	NIP7	5	GRPEL1	4
FOSL1	3	PCDHGA1	3	PFDN4	5	H3F3B	4
FYN	3	PFDN4	3	RPL6	5	LOC100293704	4
HLA-F	3	PPIB	3	SNRPF	5	POLR1A	4
KCNAB3	3	PTGR2	3	TWF2	5	ACY3	3
LDLR	3	RABGGTB	3	YAP1	5	ADCK3	3
LY6E	3	SLC2A12	3	ACTB	4	AR	3
NAA30	3	TBX19	3	BAK1	4	CARD14	3
NEMF	3	TTBK1	3	COPB2	4	CCKBR	3
NIP7	3	ABCA12	2	CPA3	4	CDK1	3
NUP62CL	3	ACVR1B	2	CXCL9	4	CENPE	3
OCM	3	ADAM10	2	DENND2C	4	CIART	3
PAGE2B	3	ADD1	2	DYNC1H1	4	CMTM5	3
PHKG1	3	AGAP5	2	Eif6	4	COG4	3
PNPLA8	3	AGBL2	2	FAM185A	4	COPB2	3
PRDM15	3	AIFM2	2	FOXO6	4	DCSTAMP	3
RASIP1	3	ANKS1A	2	GAPDH	4	DDX47	3
RASSF10	3	ARFIP1	2	GJA8	4	DGKK	3
SLX4	3	ARHGAP9	2	HEATR4	4	DTX3	3
SRRD	3	ARMC8	2	HEMGN	4	DZANK1	3
ST5	3	ARSA	2	ITM2B	4	EIF3H	3
VPS72	3	ATF7IP2	2	MLLT6	4	ELL2	3
ZBTB20	3	ATP5H	2	NAA30	4	EPX	3
ZCCHC10	3	AURKA	2	NIT1	4	Fam154b	3
ZNF555	3	AXDND1	2	OCM	4	GPR65	3
ZNF565	3	BIRC7	2	OR5AN1	4	GRINA	3
ABR	2	BTNL2	2	PLK1	4	HADHB	3
ACACB	2	C1orf194	2	POLR2B	4	HIAT1	3
ACOXL	2	C1QTNF6	2	PPAN	4	HYPK	3
ACVR2B	2	C2ORF16	2	PPIB	4	KAT5	3
ADCK1	2	C9orf16	2	PRDM15	4	L3MBTL3	3
ADRBK2	2	CA5A	2	RAE1	4	LALBA	3

AGA	2	CCDC17	2	SLITRK3	4	LOC101928761	3
AIG1	2	CCDC22	2	STRA13	4	LYPD2	3
AKAP3	2	CCND2	2	UBB	4	MAGEA9	3
ALG14	2	CD276	2	URB1	4	MSANTD3	3
Apobr	2	CHAF1A	2	WBSCR28	4	NAA30	3
APOC2	2	CHRND	2	ZNF19	4	NDOR1	3
APOL5	2	CLCN6	2	A4GNT	3	NDUFS2	3
APOL6	2	CLDND1	2	ABR	3	NIPBL	3
AQP4	2	CLEC6A	2	ADAM30	3	OLFM4	3
ARHGAP15	2	CNOT7	2	ADAT2	3	PARP6	3
ARHGAP44	2	COA1	2	AGO3	3	PCCB	3
ARHGAP9	2	COL25A1	2	AKAP3	3	PHOX2A	3
ASB11	2	COL4A1	2	APAF1	3	POLB	3
ASIC4	2	CORO1B	2	ARHGEF15	3	PRKD3	3
ASMTL	2	COX6B1	2	ARMC8	3	PTRH2	3
ATE1	2	CPO	2	ATP6V0E1	3	RASGEF1C	3
ATP1B4	2	Cul9	2	ATP9A	3	RCE1	3
ATPAF1	2	DCTPP1	2	BAIAP3	3	REL	3
AURKA	2	DCUN1D1	2	BMP4	3	RGCC	3
B9D2	2	DDA1	2	BZW2	3	RMDN2	3
Bend3	2	DDX23	2	C11ORF31	3	RPGRIP1L	3
BIN3	2	DFNA5	2	C17orf70	3	SEMA4F	3
BMP6	2	DHCR24	2	C1ORF27	3	SLC25A25	3
BRINP1	2	DLG1	2	C5AR2	3	SLC5A4	3
BRINP2	2	DPYSL3	2	C9ORF72	3	SSPN	3
C12orf60	2	DRD5	2	CA5B	3	SYNJ2	3
C14orf177	2	DUOXA2	2	CAPNS1	3	TMEM259	3
C16orf90	2	EBP	2	Carkd	3	TRIM4	3
C17orf112	2	EFCAB13	2	CCDC59	3	TTBK1	3
C1orf127	2	EGFR	2	CCNY	3	UBB	3
C21ORF62	2	EGR4	2	CD164	3	YAP1	3
C5orf24	2	EHD1	2	CD248	3	ZNF132	3
C9ORF72	2	Eif6	2	CD33	3	ZNF391	3
CACNG3	2	ELAVL4	2	CD86	3	ZNF536	3
CAGE1	2	ELL2	2	CIAO1	3	MARCH2	2
CAMK2D	2	EML3	2	CLEC4F	3	SEPT9	2
CARD14	2	ENDOG	2	CMAS	3	AAED1	2
CASP7	2	EPN2	2	CNR2	3	ABCB6	2
CATSPERB	2	EVA1A	2	CRTAC1	3	ABCF2	2
CAV1	2	FABP5	2	CT47B1	3	ABHD12	2
CBFB	2	FAM127A	2	CTBS	3	Acap3	2
CBX6	2	FAM185A	2	CTNS	3	ACAT1	2
CCDC37	2	FAM83D	2	DBX1	3	ACE	2

CCDC57	2	FBLN7	2	Dcaf8	3	ACN9	2
CCDC64	2	FCN1	2	DEFB136	3	ACOT11	2
CCK	2	FRRS1L	2	DNAJA2	3	ADAM29	2
CCL24	2	FSTL1	2	DNASE1L3	3	ADHFE1	2
CCR9	2	GAL3ST1	2	DNASE2B	3	AGXT	2
CCRL2	2	GALNT1	2	DOPEY1	3	AGXT2	2
CD276	2	GAPT	2	DPF1	3	Akip1	2
CDH18	2	GCA	2	DPH1	3	AKR1C1	2
CECR2	2	GCSAML	2	DUOX1	3	AKT1S1	2
CEP76	2	GNL1	2	ECI2	3	ALDH1A2	2
CHD6	2	GP9	2	ELMOD2	3	ALDH4A1	2
Chpf2	2	GPR68	2	EMP1	3	ALPL	2
CHRND	2	GPS2	2	ERAL1	3	AMIGO2	2
CLK4	2	GRPEL2	2	EREG	3	ANKFN1	2
CLSTN1	2	GRXCR1	2	ERVMER34-1	3	ANKRD13B	2
CNTNAP4	2	GSPT2	2	Fam154b	3	ANKRD33	2
COL28A1	2	GTPBP6	2	FAM168A	3	AP3B1	2
COL4A4	2	GTSE1	2	FCHSD2	3	AP5S1	2
CPA3	2	H3F3B	2	FGFR3	3	APOL5	2
CPE	2	HBS1L	2	FLYWCH1	3	AQP6	2
CRCP	2	HELLS	2	FOXQ1	3	ARAP1	2
CSPG4	2	HLA-DRB4	2	GLIPR1L1	3	ARHGAP6	2
CYB5D2	2	HOMER1	2	GLTSCR1L	3	ARHGEF6	2
CYB5R4	2	HPGDS	2	GPA33	3	ARL14	2
CYP4F3	2	HSPB8	2	GPAA1	3	Aspg	2
DAZ2	2	IKZF4	2	GPR137C	3	ATF1	2
DCAF15	2	INF2	2	GRIA4	3	ATF7IP2	2
DDX47	2	INPP5A	2	GRXCR1	3	ATM	2
DFNA5	2	KANSL1L	2	HIC1	3	ATP10A	2
DGKI	2	KCNK16	2	HN1L	3	ATP1B3	2
DHX57	2	KCNRG	2	HOXC6	3	ATP5SL	2
DIABLO	2	KIAA1377	2	HP	3	ATP6V1B1	2
DIRC2	2	KLHDC2	2	HSPA1B	3	AURKA	2
DMC1	2	KLHDC7A	2	IAPP	3	AXIN1	2
DSC1	2	KLHL13	2	ICAM3	3	BAG5	2
DUSP21	2	KLHL6	2	IDNK	3	BCAT2	2
DYM	2	KLRK1	2	IGBP1	3	BCL3	2
EARS2	2	KRAS	2	IL11RA	3	BCOR	2
EBI3	2	KRTAP5-9	2	IL2RA	3	BNC2	2
ECI1	2	KRTAP9-1	2	IMPACT	3	BRINP1	2
EEF1G	2	LAT2	2	ING5	3	BRINP2	2
Eif6	2	LBP	2	IRF2BP2	3	BTBD11	2
ENPEP	2	LEMD3	2	ITIH4	3	BZW1	2

EPC1	2	LMBR1	2	ITPKC	3	C12orf55	2
Ermn	2	LOC1001327	2	KCNN2	3	C15orf39	2
ETNK1	2	LOC1005055	2	KIAA1244	3	C16orf74	2
F3	2	LOC1009967	2	KIF5C	3	C17orf70	2
FABP5	2	LOC1010605	2	KIF9	3	C17orf97	2
FAM101B	2	LOC646862	2	KRT10	3	C19orf33	2
FAM20C	2	LOXL1	2	LMAN2	3	C1GALT1	2
FAM24A	2	LPL	2	LNX2	3	C1orf174	2
FAM72A	2	LRRTM3	2	LOC1019270	3	C20ORF85	2
FAM76B	2	LYPD4	2	LOC1019275	3	C2ORF16	2
FASLG	2	MAP1A	2	LOC1019280	3	C3orf27	2
FBXL4	2	MAP3K6	2	LOC1019296	3	C9ORF96	2
FEZF1	2	MARS2	2	LOC1019302	3	CACNG7	2
FIS1	2	MCCD1	2	LOC285500	3	CALML3	2
FKBP14	2	MCTP1	2	LOC646862	3	CAPN12	2
FNIP2	2	MLLT3	2	LRIG3	3	CARD9	2
FOSB	2	MMGT1	2	LRRC8E	3	CASP2	2
FOXG1	2	MMS19	2	LYPD4	3	CASP3	2
FOXO3	2	MRGPRX4	2	MAD2L1	3	CCDC121	2
FRS3	2	MRPL48	2	MAGEB1	3	CCDC135	2
FUT10	2	MS4A12	2	MAPK15	3	CCDC22	2
FUT5	2	MUC13	2	MCU	3	CCDC80	2
FZR1	2	MYH10	2	MINPP1	3	CCL13	2
GAB1	2	NAMPT	2	MRPL30	3	CCNC	2
GABRE	2	NARS	2	MTHFD2L	3	CD109	2
GABRP	2	NDUFA11	2	MTHFS	3	CD300E	2
GALNTL5	2	NDUFV2	2	MTMR11	3	CD69	2
GARS	2	NECAB2	2	MXI1	3	CD79B	2
GCA	2	NETO2	2	MYOC	3	CD97	2
GDAP1	2	NEUROG2	2	NAPEPLD	3	CDC123	2
GGT2	2	NFKB2	2	Nbas	3	CDCP1	2
GIP	2	NIP7	2	NDOR1	3	CDH16	2
GLTSCR1L	2	NLRP3	2	NDUFA4L2	3	CDK3	2
GNAT1	2	NME2	2	NES	3	CDV3	2
GPBP1	2	NUP210	2	NFAT5	3	CELF6	2
GPBP1L1	2	NXN	2	NOP10	3	CEP76	2
GPR116	2	OLFML1	2	NOTO	3	CEP85L	2
GPR171	2	OPTC	2	NT5DC4	3	CEP89	2
GPR65	2	OR11H4	2	NT5M	3	Cers2	2
GPR83	2	OR12D3	2	NUP210	3	CFDP1	2
GRIN3A	2	OR1L6	2	NUP62CL	3	CHIC1	2
GRXCR1	2	OR8G2	2	NUS1	3	CHRNA4	2
GRXCR2	2	PADI1	2	OPHN1	3	CHRNG	2

GS1-259H13.	2	PAM	2	OPTN	3	Cisd2	2
GTF2B	2	PAQR6	2	OR2F2	3	CLCN6	2
GTF2H1	2	PATL1	2	OR5K1	3	CLEC2A	2
GTPBP6	2	PBK	2	OR7A17	3	CLINT1	2
GYG1	2	PCDH1	2	OSBPL7	3	CLIP1	2
GYS1	2	PCDHGB4	2	PACSIN2	3	CLPB	2
HIST1H4F	2	PDE1A	2	PAQR9	3	CLSTN1	2
HJURP	2	PGP	2	PCBP2	3	CNGA2	2
HOMER1	2	PHF2	2	PCDHGC4	3	CNTN1	2
HOXB4	2	PJA1	2	PDCD4	3	COL25A1	2
HSF1	2	PML	2	PDZD3	3	COL6A1	2
HSPB7	2	PMM2	2	PIK3AP1	3	COPRS	2
HYPK	2	POLR2K	2	PMM1	3	CORO2B	2
IDH3G	2	POU6F1	2	PNLDC1	3	COX14	2
IDUA	2	PPM1J	2	PNPLA4	3	CPAMD8	2
IFNL4	2	PQBP1	2	PPFIA4	3	Crybg3	2
IGLL5	2	PRDM15	2	PPM1G	3	CSNK1D	2
IL17B	2	PREPL	2	PPP1R11	3	CTNNB1	2
ING1	2	PTGES3	2	PPP3CA	3	CTTN	2
ITGB7	2	PTPRG	2	Ppp6r2	3	CXCR4	2
JADE3	2	RAB2A	2	RAC1	3	CXorf49	2
KANSL1L	2	RAD54L2	2	REC8	3	CYFIP1	2
KCNE2	2	RAE1	2	REPS2	3	CYP1A1	2
KCNH3	2	RANBP17	2	RGAG1	3	CYP1B1	2
KCNJ9	2	RBMY1J	2	RGCC	3	CYP39A1	2
KCNN1	2	RFPL4AL1	2	RIPK2	3	CYP46A1	2
KDM6A	2	RNF7	2	RNF41	3	CYR61	2
KIAA0753	2	RRAS	2	RSPO3	3	DCAF4	2
KIR3DL3	2	S1PR5	2	RYBP	3	DEFB103A	2
KLRF1	2	SCAP	2	SCN4B	3	DENND1C	2
KRAS	2	SDC2	2	SEMA4G	3	DENND5A	2
KRTAP13-1	2	SEC24A	2	SERBP1	3	DHX29	2
LAMP5	2	SH3TC1	2	SEZ6L	3	DHX40	2
LCA5	2	SIDT1	2	SH3RF3	3	DIAPH2	2
LCE5A	2	SLC22A2	2	SIDT1	3	DIS3L	2
LCN12	2	SLC25A3	2	SLC25A12	3	DLX5	2
LCN2	2	SLC2A14	2	SLC25A23	3	DMC1	2
LGMN	2	SLC36A3	2	SLC32A1	3	DNASE2B	2
LHX1	2	SLC6A18	2	SLC45A2	3	DNMT3B	2
LILRA2	2	SLC9A5	2	SLC6A18	3	DPEP1	2
LIN28B	2	SLIT2	2	SMAD6	3	DPP8	2
LNX2	2	SP110	2	SPAG17	3	DRAP1	2
LOC1001303	2	SPAG17	2	STYK1	3	DSCR3	2

LOC1002889	2	SPG7	2	SUPT4H1	3	DSCR4	2
LOC1005055	2	SRRD	2	THBS2	3	DSG2	2
LOC1009966	2	STAB1	2	TMED9	3	DTD1	2
LOC1009967	2	STARD3NL	2	TMEM216	3	DTL	2
LOC1019282	2	STPG2	2	TMEM45B	3	DUSP5	2
LOC1019299	2	STRBP	2	Tox2	3	DUXA	2
LOC285500	2	SYNJ2	2	TPD52L2	3	Eapp	2
LOC389831	2	TARSL2	2	TRABD2B	3	EDNRB	2
LOC646862	2	TCEA3	2	TRIM49D1	3	EEF2	2
LOC729159	2	TCN2	2	TRPM2	3	EEFSEC	2
LRFN1	2	TIMM17A	2	TSTD1	3	EGLN2	2
LRRC70	2	TIMP3	2	TTC14	3	EHD3	2
MANSC1	2	TK2	2	Ttc23l	3	EIF4G3	2
MCC	2	TMEM165	2	TTC7B	3	ELF5	2
MDK	2	TMEM216	2	UBL4A	3	ELOVL1	2
MECOM	2	Tmem237	2	UHMK1	3	EMC1	2
METRNL	2	TMEM41B	2	UNC13D	3	EPHB2	2
MFSD6	2	TMEM9	2	UXS1	3	ERBB2IP	2
MROH2B	2	TNFRSF13C	2	VTN	3	Ermn	2
MRPL39	2	TRAM1	2	WDR31	3	ERP44	2
MRPS11	2	TRH	2	ZBTB32	3	EVI2B	2
MRPS36	2	TRIM39-RPP2	2	ZC3HC1	3	EXO1	2
Ms4a15	2	Ttc39a	2	ZFYVE19	3	FAM110B	2
MTNR1B	2	TUBGCP5	2	ZNF300	3	FAM129C	2
NAT16	2	TVP23B	2	ZSCAN16	3	FAM175B	2
NBPF9	2	TYROBP	2	MARCH9	2	FAM185A	2
NCAM1	2	URB1	2	SEPT1	2	FAM209A	2
NEU2	2	USP9X	2	SEPT8	2	FAM20C	2
NFE2L1	2	VPS4A	2	A2ML1	2	FAM217B	2
NHS	2	YAP1	2	AADACL4	2	FAM25A	2
NKAIN3	2	YEATS4	2	AARSD1	2	FAM43B	2
NNAT	2	ZBTB8B	2	ABCC3	2	FAM98B	2
NPAS1	2	ZNF282	2	ABCG5	2	FASLG	2
NPL	2	ZNF500	2	ABHD2	2	FBXO48	2
NPM3	2	ZNF502	2	ACOT13	2	FBXW5	2
NPPB	2	ZNF606	2	ACSF3	2	FCGR3B	2
NR1H4	2	ZNF652	2	ACSM3	2	FER1L5	2
NRIP2	2	ZNF727P	2	ACTC1	2	FEV	2
NUAK1	2	ZNF80	2	ACTL9	2	FGD4	2
NUDT12	2			ACY1	2	FGF10	2
NUS1	2			ACYP2	2	FHAD1	2
ODF1	2			ADAM20	2	FNIP2	2
OLFML1	2			ADAMTS10	2	FOSL2	2

ONECUT3	2
OPN1LW	2
OR10A3	2
OR10H1	2
OR10Z1	2
OR2C1	2
OR51B5	2
OR52L1	2
OR5AS1	2
OR5B12	2
OR6C75	2
OR7A5	2
OR9Q2	2
ORM2	2
OSBPL9	2
P4HB	2
PALB2	2
PALMD	2
PAM	2
PAOX	2
PCDHA3	2
PCDHA5	2
PCYT1A	2
PDCD1	2
PDE1A	2
PDHB	2
PDZRN3	2
PEX11B	2
Phax	2
PHLPP1	2
PIBF1	2
PJA2	2
PLA2G1B	2
PLEKHO1	2
POLR1A	2
PPIAL4C	2
PPIB	2
PPM1J	2
PRKAG3	2
PRR11	2
Prrt4	2
PRSS12	2
PRTN3	2

Adcy10	2	FRG2C	2
ADD1	2	FRMD3	2
ADGB	2	FRMD8	2
ADPRH	2	FSCN2	2
AGBL4	2	FUT8	2
AGT	2	FYN	2
AICDA	2	GALM	2
AIF1L	2	GALNT18	2
AIFM3	2	GBP3	2
AKAP8	2	GCM2	2
AKR1B15	2	GHR	2
AKT1S1	2	GJC3	2
AKT2	2	GLT8D2	2
ALDH16A1	2	GLTSCR1L	2
ALDH1A1	2	GLYR1	2
ALDOB	2	GOLGA6L4	2
ALK	2	GPIHBP1	2
ALKBH5	2	GPR143	2
ALKBH6	2	GPR75-ASB3	2
AMACR	2	GRM2	2
AMIGO1	2	GSTP1	2
AMY1B	2	GTF2H1	2
ANKLE2	2	GUCY2F	2
ANKRD20A2	2	GYG2	2
ANKRD23	2	HACE1	2
ANKRD35	2	HDGFRP3	2
ANKS1A	2	HDLBP	2
ANO6	2	HES7	2
ANPEP	2	HFM1	2
ANXA7	2	HGD	2
AP1S1	2	HGS	2
AP2A2	2	HIST2H4B	2
AP2M1	2	HLA-F	2
AP3B1	2	Hmgn5	2
AP3D1	2	HOMER1	2
AP5S1	2	HOXB4	2
APEX1	2	HPCAL1	2
APOBEC3A	2	HSBP1L1	2
APOC2	2	HSD3B2	2
APOC3	2	HVCN1	2
APP	2	HYI	2
AR	2	ID3	2
ARF4	2	IFNA2	2

PSME4	2
PTCH1	2
PVALB	2
PVRL2	2
QRICH2	2
RAP1GDS1	2
RBL2	2
RCN2	2
REL	2
RFPL2	2
RFPL4B	2
RIPK2	2
RNASE6	2
RNF111	2
RNF115	2
RNF150	2
ROBO4	2
RTKN	2
RTN4RL1	2
RUFY3	2
RYBP	2
S100A12	2
S100A7	2
SAMD1	2
SAP130	2
SCAF11	2
SCARB1	2
SDAD1	2
SERPINA6	2
SESN2	2
SGSH	2
SH2D1B	2
SH2D7	2
SHANK1	2
SIT1	2
SLC12A1	2
SLC22A2	2
Slc22a20	2
SLC30A1	2
SLC37A3	2
SLCO5A1	2
SMPX	2
SNAI1	2

ARFIP1	2	IFRD2	2
ARHGAP19	2	IFT80	2
ARHGAP30	2	IGF2BP1	2
ARHGAP44	2	IKZF1	2
ARHGAP6	2	IPPK	2
ARHGDIA	2	ITGB1BP2	2
ARHGEF10L	2	ITGB5	2
ARHGEF38	2	ITPR1	2
ARL6IP5	2	JOSD1	2
ARMC9	2	KCNE2	2
ARNT2	2	KCNQ3	2
ARPC5L	2	KCNV2	2
ARSI	2	KCTD17	2
ASB16	2	KIAA0226L	2
ASCL1	2	KIAA0586	2
Aspg	2	KIAA0895L	2
ATF6B	2	KIF20B	2
ATF7IP2	2	KIFC2	2
ATG10	2	KIR2DL5B	2
ATG14	2	KLF12	2
ATG4D	2	KLRK1	2
ATOH7	2	KNDC1	2
ATP10A	2	KNOP1	2
ATP13A2	2	KPNA7	2
ATP2A3	2	KRIT1	2
ATP4B	2	KRT6A	2
ATP6AP1	2	KRT7	2
ATP6V1H	2	KRTAP10-7	2
ATXN1	2	L1TD1	2
BABAM1	2	LAMB1	2
BAGE	2	LAMC2	2
BAIAP2L2	2	LARGE	2
BAMBI	2	LCN2	2
BAP1	2	LEFTY1	2
BBS2	2	LEKR1	2
BCDIN3D	2	LETMD1	2
BCL7A	2	LHFPL5	2
BCL9L	2	LIG3	2
BCOR	2	LINC00684	2
BDH2	2	LMAN2	2
BGN	2	LMNB1	2
BHMT	2	LMOD3	2
BIRC6	2	LOC100132004	2

Snrnp70	2
SNRPF	2
SNUPN	2
SOCS7	2
SOX8	2
SPATA22	2
SRPK3	2
SS18	2
SSPN	2
STARD3NL	2
STOM	2
STPG1	2
SULT1B1	2
TAC1	2
TAF5	2
TCERG1L	2
TCTN3	2
TG	2
TGFBR3L	2
TICRR	2
TLE4	2
TMEM126A	2
TMEM198	2
TMEM218	2
TMEM240	2
TMEM241	2
TMTC3	2
TNFRSF17	2
TNFSF14	2
TNK1	2
TOMM34	2
TOR3A	2
TRIM16L	2
TRIM54	2
TRIM56	2
TRIM60	2
TSHZ3	2
TSPAN12	2
TSPAN5	2
Tti2	2
UBB	2
ULBP3	2
USP51	2

BLOC1S3	2	LOC100132731	2
BOK	2	LOC100287294	2
BOLA2B	2	LOC100288562	2
BPIFA3	2	LOC100509091	2
BPIFB2	2	LOC100652901	2
BRINP1	2	LOC101059914	2
BRIP1	2	LOC101060022	2
BRPF1	2	LOC101060179	2
BRSK2	2	LOC101060861	2
BTLA	2	LOC101927594	2
BTN1A1	2	LOC101927751	2
BTNL10	2	LOC101928044	2
C10ORF11	2	LOC101928638	2
C11orf82	2	LOC101928728	2
C11orf91	2	LOC101929097	2
C12orf71	2	LOC101929469	2
C14ORF169	2	LOC101929571	2
C14ORF2	2	LOC101930006	2
C15orf38-AP3	2	LOC101930125	2
C15orf54	2	LOC101930480	2
C16orf80	2	LOC101930551	2
C17orf104	2	LOC391722	2
C17orf64	2	LRFN3	2
C18orf63	2	LRIG3	2
C1GALT1	2	LRRC19	2
C1GALT1C1	2	LRRC42	2
C1orf159	2	LRRC43	2
C1orf85	2	LRRC73	2
C1orf95	2	LRRC8E	2
C1QTNF3	2	LSM4	2
C1R	2	LY6E	2
C1S	2	LY6G6C	2
C20orf196	2	LYPD1	2
C20ORF85	2	MAD2L1	2
C21ORF33	2	MAN1C1	2
C2orf81	2	MAP3K15	2
C3orf58	2	MCHR1	2
C5orf24	2	MCOLN1	2
C5orf42	2	MCPH1	2
C5orf51	2	MEA1	2
C5orf58	2	MED29	2
C6	2	METTL11B	2
C8orf37	2	MFSD4	2

VGF	2
VPS11	2
VPS52	2
VSTM5	2
WBP11	2
WDR17	2
XPNPEP1	2
YAP1	2
YARS	2
ZBED3	2
ZBTB14	2
ZBTB7C	2
ZC3H3	2
ZMYM2	2
ZNF132	2
ZNF19	2
ZNF20	2
ZNF215	2
ZNF264	2
ZNF560	2
ZNF569	2
ZNF585B	2
ZNF8	2
ZSCAN16	2
ZSCAN32	2

C8orf76	2	MIER3	2
C9orf172	2	MIXL1	2
C9ORF37	2	MKL2	2
CA6	2	MLPH	2
CAB39	2	MMADHC	2
CABP5	2	MON1B	2
CABYR	2	MRPL24	2
CACNG3	2	MRPS18C	2
CALD1	2	MS4A12	2
CALML5	2	Ms4a15	2
CAPZA1	2	MSLN	2
CARHSP1	2	MSS51	2
CARM1	2	MTFR1	2
CASC10	2	MTHFR	2
CASP5	2	MTUS2	2
CASQ1	2	MTX2	2
CATSPER4	2	NAA35	2
CBX6	2	NAAA	2
CCDC115	2	NAB1	2
CCDC12	2	NAV3	2
CCDC121	2	NCKAP1L	2
CCDC13	2	NDRG3	2
Ccdc138	2	NDST2	2
CCDC176	2	NEDD4	2
CCDC178	2	NEUROG2	2
CCDC27	2	NFE2L3	2
CCDC30	2	NKG7	2
CCDC53	2	NKPD1	2
CCDC54	2	NMRAL1	2
CCDC91	2	NMUR2	2
CCK	2	NOL4	2
CCL4L2	2	NOTCH1	2
CCM2L	2	NOTCH2	2
CCND2	2	NPAS1	2
CCNE1	2	NPNT	2
CCNE2	2	NPY4R	2
CCT8	2	NRD1	2
CCZ1B	2	NSUN3	2
Cd101	2	NT5DC4	2
CD226	2	NT5M	2
CD302	2	NUMA1	2
CD69	2	NUP210	2
CD96	2	NUP62CL	2

CDC25A	2	NXN	2
CDC42BPA	2	OLFM2	2
CDCA7L	2	ONECUT3	2
CDK1	2	OR11A1	2
CDK2AP1	2	OR12D2	2
CDK3	2	OR12D3	2
CDKN3	2	OR13C2	2
CDY2A	2	OR14J1	2
CELF6	2	OR1L3	2
CEP135	2	OR2AG2	2
CEP170B	2	OR2F2	2
CER1	2	OR4K2	2
CERKL	2	OR4M1	2
CFD	2	OR4M2	2
CHAMP1	2	OR4N4	2
CHCHD6	2	OR4P4	2
CHD2	2	OR5A2	2
CHGA	2	OR5K1	2
CHPT1	2	OR6M1	2
CHRFAM7A	2	OR8D1	2
CHST8	2	OVCA2	2
CIB3	2	OXSM	2
CIDEC	2	PACS1	2
CITED2	2	PAGE2B	2
CIZ1	2	PAK6	2
CKM	2	PARP10	2
CLCF1	2	PBX2	2
CLCNKB	2	PCCA	2
CLDN17	2	PCDH1	2
CLDN19	2	PCDHA12	2
CLDN7	2	PCDHGB3	2
CLEC6A	2	PCDHGC5	2
CLIP3	2	PCDP1	2
CLK2	2	PCYT1B	2
CLK4	2	PDCD2L	2
CLRN2	2	PDCD4	2
CNDP1	2	PDDC1	2
CNGB1	2	PDE12	2
CNIH3	2	PDZRN3	2
CNN2	2	PGP	2
CNTD1	2	PHF14	2
COA5	2	PHF2	2
COG2	2	PHKG1	2

COL24A1	2	PI3	2
COL2A1	2	PIK3C2A	2
COL4A2	2	PKD1	2
COQ7	2	PKDREJ	2
CORIN	2	PKN1	2
COX5A	2	PLA2G3	2
COX7A2	2	PLAGL1	2
COX8A	2	PLCG2	2
CP	2	PLK1	2
CPNE1	2	PLXNA2	2
CPNE5	2	PML	2
CPNE9	2	PNLIPRP2	2
CPO	2	POLA1	2
CR1	2	POLR2B	2
CRADD	2	POLR2F	2
CRISP2	2	PORCN	2
CRKL	2	POU2F3	2
CSAG1	2	POU6F2	2
CSF1	2	PPM1A	2
CSF2	2	PPP2R1A	2
CSF2RB	2	PQBP1	2
Csgalnact1	2	PRAMEF10	2
CST7	2	PRC1	2
CT83	2	PRDM15	2
CTLA4	2	PRKAR1A	2
CTNNA1	2	PRKDC	2
CTSF	2	PRSS46	2
CUL5	2	PRSS54	2
CWC27	2	Prss58	2
CYB5R4	2	PSAT1	2
CYP27B1	2	Ptpmt1	2
CYP2U1	2	PTPN20B	2
DAPK1	2	PTPN7	2
DAPK2	2	QTRT1	2
DAPL1	2	RAB11B	2
DAZ2	2	RAD23B	2
DAZAP2	2	RAPGEF6	2
DCAF15	2	RASAL1	2
DCLRE1B	2	RASAL3	2
DCUN1D5	2	RASD1	2
DDI1	2	RASIP1	2
DDO	2	RBBP6	2
DDX26B	2	RBBP9	2

DDX39A	2	RBP3	2
DDX51	2	RCAN3	2
DEFA1B	2	RDH13	2
DEFA4	2	RET	2
DEFB127	2	RGS17	2
DEGS1	2	RGS22	2
DENND5A	2	RHOBTB3	2
DERA	2	RHOT2	2
DHCR7	2	RNASE6	2
DHRS7B	2	RNF186	2
DHX33	2	RNF26	2
DICER1	2	RPGR	2
DIS3L2	2	RPL37	2
DKK1	2	RPL6	2
DLD	2	RPS6KB1	2
DLX2	2	RRH	2
DMRT1	2	RRP1	2
DMXL2	2	RTP3	2
DNAI1	2	RUFY3	2
DNAJB1	2	S100A1	2
DNAJB6	2	SCAMP2	2
DNAJC18	2	SCARB2	2
DNAJC22	2	SEC31B	2
DOK1	2	SEH1L	2
DPF3	2	SENP2	2
DRG2	2	SERINC2	2
DTX3	2	SERINC4	2
DTX4	2	SERPINB12	2
DUS4L	2	SERPINH1	2
DUSP12	2	SFTPD	2
DUSP21	2	SGCB	2
DUSP3	2	SHKBP1	2
DVL3	2	SHOX	2
DYNC2H1	2	SIK3	2
E2F2	2	SIRT1	2
ECHDC2	2	SLC12A4	2
EDC4	2	SLC16A14	2
EEA1	2	SLC1A5	2
EED	2	SLC22A3	2
EFCAB1	2	SLC26A7	2
EFCAB12	2	SLC31A1	2
EFCAB13	2	SLC4A4	2
EFCAB3	2	SLC4A7	2

EFHC1	2	SLC9C1	2
EIF2AK2	2	SLCO2B1	2
EIF4A1	2	SMN2	2
EIF4E3	2	SNAI1	2
EIF5	2	Snrnp25	2
ELF4	2	SNRNP27	2
ELMOD3	2	SNX1	2
EML4	2	SNX4	2
ENKD1	2	SOCS5	2
ENPP1	2	SOWAHD	2
ENPP3	2	SPATA31A6	2
ENTPD1	2	SPIN2B	2
EPB41L4A	2	SPRR2E	2
EPHA4	2	SRD5A1	2
EPHB2	2	SRI	2
EPHX3	2	SSTR1	2
ERAP2	2	SSX2	2
ERI1	2	ST3GAL5	2
ERICH1	2	STK24	2
ERICH4	2	STK38	2
ERICH6	2	STK4	2
Erlec1	2	STOM	2
EVA1B	2	STRIP2	2
EVX2	2	SULT2B1	2
EXD1	2	SVIL	2
EXOSC4	2	SYNJ2BP-COX1	2
EYS	2	SYT1	2
F13B	2	TAS2R19	2
FAF1	2	TBL3	2
FAM110C	2	TBX10	2
FAM120A	2	TEX11	2
FAM124A	2	TEX29	2
FAM133A	2	TFAM	2
FAM134B	2	TGFBR3L	2
FAM153B	2	TGM6	2
FAM160A1	2	THEM4	2
FAM163A	2	TIMM22	2
FAM173B	2	TMEM114	2
Fam174b	2	TMEM132E	2
FAM177A1	2	TMEM138	2
Fam181b	2	TMEM144	2
FAM195B	2	TMEM165	2
FAM19A1	2	TMEM176B	2

FAM25A	2	TMSB4Y	2
FAM43B	2	TNFAIP3	2
FAM50B	2	TNFSF12	2
FAM58A	2	TNPO3	2
FAM65A	2	Tomm20l	2
FAM76B	2	TOMM34	2
FAM78B	2	TRADD	2
FAM83A	2	TRAF5	2
FANCL	2	TRAP1	2
FAT3	2	TRAPPC9	2
FBXO4	2	TRIB1	2
FBXW2	2	TRIM24	2
FERMT1	2	TRIM51	2
FETUB	2	TRIOBP	2
FFAR2	2	TRIP10	2
FGF10	2	TRPV3	2
FGL2	2	TSPAN11	2
FHIT	2	TSSK4	2
FHL3	2	TTC5	2
FKBP2	2	TTL	2
FN3KRP	2	TVP23C-CDRT4	2
FOXN2	2	UBE2D2	2
FPR2	2	UBE2E3	2
FRA10AC1	2	UBE2QL1	2
FRAT1	2	UBR7	2
FREM2	2	UNK	2
FSTL5	2	UPF3A	2
FUT7	2	URB1	2
FUT8	2	USP3	2
GAB1	2	USP51	2
GADD45B	2	USP9Y	2
GADD45G	2	UTRN	2
GALNT11	2	VCPIP1	2
GALNT14	2	VIL1	2
GALNT18	2	VMO1	2
GAPT	2	VPS26A	2
GAS6	2	VPS45	2
GBP2	2	VPS52	2
GCM2	2	VRK3	2
GCSH	2	Vsig10	2
GET4	2	WBSCR27	2
GIMAP1	2	WRN	2
GIMAP7	2	Zc3h18	2

GJC3	2	ZC3H3	2
GKN2	2	ZC3H7A	2
GLCCI1	2	ZDHHC1	2
GLRA1	2	ZDHHC9	2
GMFG	2	ZMPSTE24	2
GMIP	2	ZNF202	2
GNB2	2	ZNF229	2
GNL1	2	ZNF319	2
GOLGA6L1	2	ZNF329	2
GON4L	2	ZNF331	2
GP6	2	ZNF445	2
GPATCH11	2	ZNF473	2
GPBP1	2	ZNF501	2
GPM6B	2	ZNF502	2
GPR12	2	ZNF521	2
GPR139	2	ZNF555	2
GPR160	2	ZNF560	2
GPR6	2	ZNF576	2
GPR68	2	ZNF585A	2
GPSM3	2	ZNF595	2
GRM2	2	ZNF600	2
GSDMA	2	ZNF606	2
GSPT2	2	ZNF615	2
GSR	2	ZNF623	2
GTF2B	2	ZNF669	2
GTF2H1	2	ZNF786	2
GTF2H3	2	ZNF8	2
GTF2H4	2	ZNF839	2
GTPBP2	2	ZNF891	2
GUCD1	2	ZRANB3	2
GUCY2D	2	ZSCAN9	2
GYPC	2		
GYS1	2		
GZMA	2		
H1FOO	2		
H1FX	2		
HAND2	2		
HAUS5	2		
Hbq1	2		
HDHD3	2		
HEATR3	2		
HEPHL1	2		
HES7	2		

HEXIM1	2
HGD	2
HHIPL1	2
HHLA2	2
HIBCH	2
HIF1A	2
HIST1H1C	2
HK2	2
HLA-DQB1	2
HMGB3	2
Hmgn5	2
HMOX1	2
HMOX2	2
HNRNPCP5	2
HOMER1	2
HPDL	2
HPR	2
HSD11B1	2
HSD17B10	2
HSF1	2
HSPA12B	2
HSPBAP1	2
HSPBP1	2
HTR3D	2
HUS1	2
HVCN1	2
HYAL2	2
HYAL3	2
IBTK	2
ICOSLG	2
IDS	2
IGFALS	2
IGJ	2
IGSF23	2
IKZF2	2
IKZF4	2
IL15RA	2
IL1B	2
IL20	2
IL21	2
IL2RG	2
IL4	2
IMPAD1	2

INADL	2
ING1	2
INTS1	2
INTS10	2
IQCF1	2
Iqcf5	2
IRF2BPL	2
IRS1	2
ISY1	2
ITGA1	2
IVL	2
IZUMO3	2
JAGN1	2
JSRP1	2
KCMF1	2
KCNAB3	2
KCNJ10	2
KCNMB4	2
KCTD21	2
KDM8	2
KIAA0319L	2
KIAA0391	2
KIAA1024L	2
KIAA1191	2
KIAA1429	2
KIAA1456	2
KIAA2013	2
KIF13A	2
KIF20B	2
KIF25	2
KIF27	2
KIR2DL5A	2
KISS1R	2
KLF12	2
KLHL13	2
KLHL28	2
KLHL36	2
KLHL4	2
KLK7	2
KLRC1	2
KLRF1	2
KNG1	2
KNSTRN	2

KPNA6	2
KPRP	2
KRT17	2
Krt222	2
KRT25	2
KRT34	2
KRT6A	2
KRT78	2
KRT83	2
KRT84	2
KRTAP12-4	2
KRTAP5-5	2
KTI12	2
L3MBTL2	2
LAMA1	2
LAMC2	2
LAMP5	2
LAMTOR3	2
LCE6A	2
LCTL	2
LDB1	2
LDB2	2
LDHB	2
LECT1	2
LENG8	2
LGI2	2
LHX3	2
LIAS	2
LIN28B	2
LIN54	2
LMAN2L	2
LMBR1	2
LMOD3	2
LMTK3	2
LNX1	2
LOC1001299	2
LOC1001303	2
LOC1001304	2
LOC1001331	2
LOC1002937	2
LOC1005054	2
LOC1005055	2
LOC1005062	2

LOC1005069	2
LOC1009963	2
LOC1009963	2
LOC1009966	2
LOC1009966	2
LOC1009967	2
LOC1010603	2
LOC1010605	2
LOC1019275	2
LOC1019280	2
LOC1019290	2
LOC1019293	2
LOC1019297	2
LOC1019299	2
LOC1019299	2
LOC1019300	2
LOC1019300	2
LOC1019303	2
LOC1019305	2
LOC1019305	2
LOC1019306	2
LOC391322	2
LOC441155	2
LOC642441	2
LOC643802	2
LOC646588	2
LONP2	2
LOXL1	2
LPAR4	2
LPHN1	2
LPO	2
LPPR2	2
LPPR3	2
LRP11	2
LRRC14B	2
LRRC23	2
LRRC25	2
LRRC56	2
LRRC57	2
LRRC70	2
LRRC73	2
LSM1	2
LSS	2

LUZP6	2
LYPD1	2
LYPD5	2
LYPLAL1	2
LYSMD3	2
LYZL6	2
M1AP	2
M6PR	2
MAGEA1	2
MAGEA10	2
MAGEB18	2
MANEA	2
MAP2K2	2
MAP2K4	2
MAP2K6	2
MAP3K6	2
MAP4K5	2
MAPK3	2
MBLAC2	2
MCAT	2
MCOLN1	2
MDGA1	2
MDP1	2
MEIS3	2
METTL23	2
MEX3D	2
MFAP5	2
MFI2	2
MFN1	2
MFSD4	2
MFSD6	2
MIF4GD	2
MIPEP	2
MKRN2	2
MLLT4	2
MMP28	2
MMS19	2
MNAT1	2
MOB2	2
MPC1	2
MPND	2
MPZ	2
MRGPRX4	2

MROH2A	2
MRPL36	2
MRPS14	2
MRPS16	2
MS4A4A	2
MSC	2
MSH3	2
MSTO1	2
MTA1	2
MTDH	2
MTNR1B	2
MTR	2
MTRNR2L3	2
MTRNR2L5	2
MTUS1	2
MUC21	2
MUC3B	2
MVP	2
MYCBP	2
MYH6	2
MYO16	2
MYO5C	2
MYO9B	2
MYOD1	2
N4BP1	2
NAA25	2
NAA35	2
NAA60	2
NAB2	2
NAGPA	2
NAMPT	2
NAP1L5	2
NAT2	2
NAV3	2
NCALD	2
NCF1	2
NCOA3	2
NCSTN	2
NDUFA5	2
NDUFA7	2
NECAB1	2
NFE4	2
NFU1	2

NFX1	2
NIPAL2	2
NKD1	2
NKIRAS2	2
NOL10	2
NOS1	2
NOS2	2
NPAT	2
NPBWR2	2
NPHS2	2
NPIPA1	2
NPL	2
NPM3	2
NPSR1	2
NPY4R	2
NPY5R	2
NR1I2	2
NSD1	2
NSMCE2	2
NT5C1A	2
NT5C1B	2
NT5DC3	2
NTN1	2
NUDT14	2
NWD1	2
NXF2B	2
NXNL1	2
OCSTAMP	2
ODF3L1	2
OFD1	2
OGDHL	2
OLFM3	2
ONECUT3	2
OR10J1	2
OR14C36	2
OR14J1	2
OR1M1	2
OR2A5	2
OR2M4	2
OR2M7	2
OR2T29	2
OR4D6	2
OR4K5	2

OR4M2	2
OR51B5	2
OR51B6	2
OR52K2	2
OR5A2	2
OR6K2	2
OR8A1	2
OR8B3	2
OR8G2	2
ORMDL1	2
OTOR	2
OTUB1	2
OXCT2	2
OXSM	2
P2RY8	2
P4HTM	2
Pabpc1l2b	2
PABPN1	2
PAGE2B	2
PAGE4	2
PAPL	2
PARD3	2
PARP10	2
PARP3	2
PATE1	2
PBK	2
PBXIP1	2
PCDH11X	2
PCDHA8	2
PCDHGA10	2
PCDHGB2	2
PCSK9	2
PDCD1	2
PDCL2	2
PDE1A	2
PDE2A	2
PDIA2	2
PDIA4	2
PEAK1	2
PELI2	2
PFDN2	2
PFKFB1	2
PFKFB2	2

PGAM5	2
PGRMC2	2
PHF14	2
PHF2	2
PHF20	2
PHYHIP	2
PI4KA	2
PIF1	2
PIGR	2
PIH1D3	2
PIK3C2A	2
PITPNB	2
PITPNC1	2
PITPNM3	2
PIWIL1	2
PJA1	2
PKN1	2
PLAGL1	2
PLCD3	2
PLEKHS1	2
PNLIPRP3	2
POC1B-GALN	2
POLN	2
POLR2C	2
POLR2G	2
POMZP3	2
PON1	2
PON3	2
POU2AF1	2
POU2F1	2
PPM1M	2
PPP1R14A	2
PPP1R27	2
PPP1R35	2
PPP2R1A	2
PPP2R3C	2
PPP2R5A	2
PPP6R1	2
PRADC1	2
PRAME	2
PRAMEF6	2
PRDM13	2
PRDM4	2

PRDM5	2
PRIMPOL	2
PRKX	2
PRMT2	2
PROM2	2
PRPF40B	2
PRR14L	2
PRR20C	2
PRR21	2
PRSS50	2
PSIP1	2
PSPC1	2
PTBP3	2
PTCD1	2
PTGES3	2
PTGS2	2
PTPDC1	2
PTPN13	2
PTPN7	2
PTPRE	2
PTPRZ1	2
PVALB	2
PVRL1	2
PVRL2	2
PYROXD2	2
QPCTL	2
QRFPR	2
QRSL1	2
RAB17	2
RAB27A	2
RAB41	2
RABGEF1	2
RABGGTB	2
RAD23B	2
RAD51	2
RANBP6	2
RANBP9	2
RASL10A	2
RBAK	2
RBL1	2
RBM34	2
RBM45	2
RBMY1J	2

RBP3	2
RC3H2	2
RCBTB2	2
RCCD1	2
RELN	2
RELT	2
REPS1	2
RETNLB	2
RFESD	2
RFPL4AL1	2
RFXANK	2
RGL2	2
RGR	2
RGS18	2
RGS20	2
RHOJ	2
RIMBP3	2
Ripply1	2
RLN1	2
RMND1	2
RNASE7	2
RNASE8	2
RNASEH2A	2
RND2	2
RNF112	2
RNF135	2
RNF145	2
RNF149	2
RNF169	2
RNF181	2
RNF208	2
RNF214	2
Rnf217	2
RNFT2	2
RNMT	2
RPN2	2
RPS18	2
RPS6KA1	2
RPS6KB1	2
RPTOR	2
RRAGC	2
RRBP1	2
RRP15	2

RRP36	2
RSPO4	2
RUSC1	2
S100A10	2
S100A2	2
S100A7	2
SAA2-SAA4	2
SAMD7	2
SAMSN1	2
SCGN	2
SCRN1	2
SCYL3	2
SDF2L1	2
SDR16C5	2
Sec16a	2
SEC31B	2
SEMA4F	2
SEPN1	2
SERGEF	2
SERPINB11	2
SERPINE3	2
SFI1	2
SFXN2	2
SGOL2	2
SGPP2	2
SGSM1	2
SH3BP5	2
SH3KBP1	2
SH3PXD2B	2
SHB	2
SHBG	2
SHCBP1	2
SHD	2
SHE	2
SHMT2	2
SIAH2	2
SIK3	2
SIRT1	2
SIRT7	2
SIT1	2
SKIL	2
SLC10A5	2
SLC13A5	2

SLC16A3	2
SLC17A6	2
SLC20A1	2
SLC20A2	2
SLC22A1	2
SLC22A2	2
SLC22A9	2
SLC24A4	2
SLC24A5	2
SLC25A28	2
SLC25A33	2
SLC25A46	2
SLC26A11	2
SLC2A3	2
SLC30A1	2
SLC30A7	2
SLC34A1	2
SLC34A3	2
SLC35C2	2
SLC35D3	2
SLC35E2	2
SLC35G1	2
SLC35G6	2
SLC43A2	2
SLC44A3	2
SLC4A9	2
SLC5A2	2
SLC5A4	2
SLC9A5	2
SLCO1B1	2
SLCO3A1	2
SLCO4C1	2
SLCO5A1	2
SMAD7	2
SMAP2	2
SMARCA5	2
SMARCD2	2
SMCO2	2
SMCO3	2
SMCO4	2
SMIM15	2
SMIM22	2
SNAI3	2

SNX32	2
SOCS6	2
SOD2	2
SOST	2
SOX10	2
SOX11	2
SPACA1	2
SPAG5	2
SPATA16	2
SPATA24	2
SPATA25	2
SPINT3	2
SPRR2E	2
SPRY4	2
SPSB2	2
SRD5A3	2
SRI	2
SRPR	2
SRPX2	2
SRSF3	2
SRSF8	2
SSBP2	2
SSR1	2
SSR2	2
SSX2	2
ST14	2
ST6GALNAC1	2
ST6GALNAC5	2
STAG1	2
STAT1	2
STC2	2
STIP1	2
STK36	2
STK4	2
STK40	2
STOML2	2
STRIP2	2
STX17	2
SUGCT	2
SUGP2	2
SULT1A2	2
SULT4A1	2
SUN1	2

SUPT20HL2	2
SYBU	2
SYCP1	2
SYDE2	2
SYF2	2
SYN1	2
SYNGAP1	2
SYNJ2	2
SYT15	2
SYT16	2
TAAR1	2
TAAR9	2
TACO1	2
TACR2	2
TAF12	2
TAF1C	2
TAF1L	2
TAF7	2
TANGO2	2
TAOK1	2
TAOK2	2
TARBP2	2
TAS2R19	2
TAS2R31	2
TAS2R41	2
TAS2R8	2
TBX5	2
TCEAL6	2
TCN1	2
TCOF1	2
TCP11X2	2
Tctex1d2	2
TDP2	2
TDRD5	2
TESC	2
TESK2	2
TEX26	2
TFB2M	2
TFDP3	2
TGIF1	2
TGM3	2
THAP5	2
THSD7B	2

TIAF1	2
TIFA	2
TIMM10	2
TIMM17A	2
TINAG	2
TJP1	2
TLL2	2
TLR5	2
TLX2	2
TM2D1	2
TM4SF5	2
TM9SF2	2
TMC3	2
TMEM144	2
TMEM14A	2
TMEM167A	2
TMEM176A	2
TMEM181	2
TMEM183A	2
TMEM200C	2
TMEM212	2
TMEM229A	2
Tmem237	2
TMEM253	2
TMEM38B	2
TMEM50B	2
TMEM56	2
TMEM67	2
TMPRSS11E	2
TNFAIP1	2
TNIK	2
TNIP2	2
TNNI2	2
TNRC6B	2
Tomm20l	2
TOMM34	2
TPPP	2
TPTE	2
TRAF3	2
TRAK2	2
TRAPPC11	2
TRAPPC2L	2
TRDMT1	2

TRDN	2
TRIB1	2
TRIM56	2
TRIM63	2
TRIML1	2
TRIOBP	2
TRMT10C	2
TRMT13	2
TRMT6	2
TRUB1	2
TSPAN12	2
TSPAN2	2
TSPYL6	2
TST	2
TSTA3	2
TTBK2	2
TTC19	2
TTF1	2
TTLL5	2
TUBB4B	2
TUSC3	2
TXNDC11	2
TXNDC17	2
TYR	2
UAP1L1	2
UBAP2	2
UBE2A	2
UBE2Q2L	2
UBE2V2	2
UBE3B	2
UBQLN3	2
UBR7	2
UGT1A1	2
UGT1A9	2
UGT2B10	2
Unc119b	2
UPF2	2
UPF3A	2
UQCC1	2
UQCRQ	2
URAD	2
USH1C	2
USP12	2

USP14	2
USP25	2
USP51	2
VIPAS39	2
VMAC	2
VPS36	2
VRTN	2
VSX2	2
Vwa3b	2
WDR12	2
WDR53	2
WDR63	2
WDSUB1	2
WFIKKN1	2
WFS1	2
WIPF3	2
WIPI2	2
WNT5A	2
XCL1	2
XKR3	2
YEATS2	2
YIF1A	2
YIPF5	2
YPEL2	2
ZBED3	2
ZBTB20	2
ZBTB37	2
ZBTB39	2
ZBTB49	2
ZC3H10	2
ZCCHC3	2
ZCCHC4	2
ZDHHC8	2
ZKSCAN2	2
ZMPSTE24	2
ZNF106	2
ZNF136	2
ZNF185	2
ZNF212	2
ZNF304	2
ZNF319	2
ZNF324B	2
ZNF331	2

ZNF33B	2
ZNF34	2
ZNF385B	2
ZNF410	2
ZNF419	2
ZNF461	2
ZNF485	2
ZNF502	2
ZNF512	2
ZNF561	2
ZNF569	2
ZNF576	2
ZNF593	2
ZNF600	2
ZNF615	2
ZNF654	2
ZNF669	2
ZNF677	2
ZNF70	2
ZNF71	2
ZNF746	2
ZNF775	2
ZNF776	2
ZNF780B	2
ZNF8	2
ZNF891	2
ZNF93	2
ZRANB3	2
ZSCAN30	2
ZSCAN9	2

radiation resistance or sensitivity

[resistant] and [control]:	[resistant] and [HU]:	[resistant] and [Dox]:	[resistant] and [HU + Dox]:	[sensitive] and [Control]:	[sensitive] and [HU]:	[sensitive] and [Dox]:
SEPT1	ABCC4	SEPT1	SEPT1	AHSG	ACE	AHSG
AP2B1	ATP6V0E1	AARS	AKT1	BAZ1B	AFP	AKAP13
APBB1	BRD2	ABHD13	ATP6V0E1	CAPN1	ASB7	ALOX5AP
ATP2B2	BRSK1	ADIPOR1	C1GALT1C1	CCR9	ASPH	ARHGAP19
BAD	C9orf16	AFTPH	C2orf42	CD302	BUB1	ATOH7
C3orf27	CASK	ALDH8A1	C3orf27	CLCN2	C1orf162	CD302
C3orf36	CDC25C	ANKK1	CA6	COPS2	CCNC	CFL1
CAPN9	CDKL3	ANXA7	CACHD1	CSF1	CHAF1A	CHCHD7
CBFB	CELSR1	ASB18	CACNG7	DARS	CITED2	CITED2
CD81	CXCL9	ASCL1	CAPN3	DGKI	COPS2	COPS2
CDK2	DNASE1L1	ASS1	CBFB	EPHB6	DNALI1	CSF1
CNTNAP4	ERBB2IP	ATP6V0E1	CDC42BPG	FIS1	FOS	CTBS
CRELD2	ESR2	BIRC6	DIDO1	GABARAPL 1	GABARAPL 1	DGCR14
CXCL1	FAM65A	BRSK1	ELL2	GEMIN8	GLI3	DGKI
CYB5D2	FBXO21	C12orf50	ERBB2IP	GNA12	ISPD	ELF3
CYB5R4	FKBP14	C5orf22	FBXL17	GRB10	ITPR2	EPRS
EPHB6	GLI3	CACNA1E	FRMD5	GRK4	KIAA1324L	FGF10
ESM1	HCFC2	CCRL2	GMNN	GYG1	MLLT3	FOXA3
FAM118B	HIF1AN	CDC25C	GTF2B	KCNJ9	MX2	GCM2
FAM65A	HOMER1	CDC42BPG	HOMER1	LCE2B	PNOC	GNA12
FKBP14	IL3	CDK2	HSPA14	LITAF	PTPN12	GNAI1
GALC	INPP5D	CRTC2	IGFBP7	LSG1	PTPRZ1	GON4L
GMNN	KCMF1	CTBP2	IL27RA	LZTS1	RESP18	HIST1H2AG
GOT2	KCNK1	CXCL9	KIAA0513	NCBP2	REST	HNF4G
GRIA4	KCTD10	CYB561D2	LMTK3	OXSR1	RNF7	HOXB4
HOMER1	LAMC2	DDO	MCHR1	PALLD	SFXN3	IPO7
KIAA0513	MTMR9	DIDO1	MED1	PGC	TCP11L1	KCNMB4
KLF9	NFKB2	DNASE1L3	MLLT4	PSMD12	TMEM9	KIAA1429
LDLR	OR10Z1	ERICH1	NKG7	RAG1	TOP1	LCE2B
LIF	OR4F6	FAM65A	OR11A1	RIPK2	UBE2D2	LINGO4
LRP4	OR6C70	FBXO46	OR2D2	RORA	WHSC1L1	LITAF

MAGEB1	PALMD	FLVCR1	PIK3C2A	SLC38A10	WRB	MED20
MS4A6E	PARP10	GAS6	POU2F3	TMEM17	ZEB2	MICAL1
NCR3	PLK2	GCM2	PPARG	TMOD2		MTA1
NDST2	PNOC	GMIP	PTCH1	TP53I11		MYH13
NEU1	PXK	GRIA4	SHKBP1	UBE2N		NALCN
NFATC2IP	RASGRP3	GSG2	SLC24A3	UBE2R2		NBPF3
NLGN3	RAVER1	HADHA	SMPDL3A	UCHL5		NHP2L1
NR5A1	RNF125	HIF1AN	STK38	USP1		NPHP1
OR4F17	SFXN3	KCMF1	TRIB1	USP51		NXPH2
OR4K5	SHOC2	KCNN2	TSPAN2	ZNF554		OXSR1
PALMD	SLMAP	LDOC1L	UHMK1	ZNF740		PABPN1
PDE10A	SMARCA2	LMX1B	UQCRFS1			PLXNA2
PFDN6	SMPDL3A	MAG	ZNF132			PPM1M
PI3	STK38	MAGEB1	ZP1			PSD3
PITX1	SYT14	MAP3K11	ZSCAN2			RAD23B
PLXNA1	TRAFD1	MLLT4				RGMB
POLE3	TRIM54	MRPL49				RIPK2
PPP1R12A	TRIM67	MSH5				RPS6KB1
RAG1	TTC4	MYF6				RYBP
RAP1GDS1	VAC14	MYO9B				S100A7A
RAPGEF5	VCAM1	NEUROD4				SASS6
RHOA	WHSC1L1	NFKB2				SCD5
RHPN1		NHP2L1				SEMA4G
SLC12A1		NKG7				SEMG2
SOX8		OR4F17				SENP8
SRPK3		OR4K5				SLC1A1
SYPL1		ORAI3				SLC38A8
TFE3		PDZD8				STX17
TLX2		PECR				TBC1D2
TMEM98		PIK3C2A				TGDS
TRIB1		PIWIL1				TK2
TRIM28		PPP3CA				TMEM183A
TRIM54		PSD3				TMPRSS11B
TRIM60		PTCH1				TOPORS
TRUB2		PTPRK				TP53I11

TSPAN2
UBE3C
USP1
WAS
WFDC8
ZNF132
ZNF418
ZNF583

RAPGEF5
REV3L
RNF125
RPL10
SGK2
SLC18A2
SLC25A28
SLMAP
SNURF
SOX11
TESK2
THAP10
TLX2
TMEM189- UBE2V1
TMPRSS4
TNF
TRPV4
TSPAN2
TYRP1
UBE3C
UGT1A1
UPF2
USP25
XPA
ZNF593

TRDMT1
TRIM21
TRIM56
WRB
ZMYND11
ZNF485

[resistant] and [control]:	[resistant] and [HU]:	[resistant] and [Dox]:	[resistant] and [HU + Dox]:	[sensitive] and [Control]:	[sensitive] and [HU]:	[sensitive] and [Dox]:
SEPT1	ABCC4	SEPT1	SEPT1	AHSG	ACE	AHSG
AP2B1	ATP6V0E1	AARS	AKT1	BAZ1B	AFP	AKAP13
APBB1	BRD2	ABHD13	ATP6V0E1	CAPN1	ASB7	ALOX5AP
ATP2B2	BRSK1	ADIPOR1	C1GALT1C1	CCR9	ASPH	ARHGAP19
BAD	C9orf16	AFTPH	C2orf42	CD302	BUB1	ATOH7
C3orf27	CASK	ALDH8A1	C3orf27	CLCN2	C1orf162	CD302
C3orf36	CDC25C	ANKK1	CA6	COPS2	CCNC	CFL1
CAPN9	CDKL3	ANXA7	CACHD1	CSF1	CHAF1A	CHCHD7
CBFB	CELSR1	ASB18	CACNG7	DARS	CITED2	CITED2
CD81	CXCL9	ASCL1	CAPN3	DGKI	COPS2	COPS2
CDK2	DNASE1L1	ASS1	CBFB	EPHB6	DNALI1	CSF1
CNTNAP4	ERBB2IP	ATP6V0E1	CDC42BPG	FIS1	FOS	CTBS
CRELD2	ESR2	BIRC6	DIDO1	GABARAPL1	GABARAPL1	DGCR14
CXCL1	FAM65A	BRSK1	ELL2	GEMIN8	GLI3	DGKI
CYB5D2	FBXO21	C12orf50	ERBB2IP	GNA12	ISPD	ELF3
CYB5R4	FKBP14	C5orf22	FBXL17	GRB10	ITPR2	EPRS
EPHB6	GLI3	CACNA1E	FRMD5	GRK4	KIAA1324L	FGF10
ESM1	HCFC2	CCRL2	GMNN	GYG1	MLLT3	FOXA3
FAM118B	HIF1AN	CDC25C	GTF2B	KCNJ9	MX2	GCM2
FAM65A	HOMER1	CDC42BPG	HOMER1	LCE2B	PNOC	GNA12
FKBP14	IL3	CDK2	HSPA14	LITAF	PTPN12	GNAI1
GALC	INPP5D	CRTC2	IGFBP7	LSG1	PTPRZ1	GON4L
GMNN	KCMF1	CTBP2	IL27RA	LZTS1	RESP18	HIST1H2AG
GOT2	KCNK1	CXCL9	KIAA0513	NCBP2	REST	HNF4G
GRIA4	KCTD10	CYB561D2	LMTK3	OXSR1	RNF7	HOXB4
HOMER1	LAMC2	DDO	MCHR1	PALLD	SFXN3	IPO7
KIAA0513	MTMR9	DIDO1	MED1	PGC	TCP11L1	KCNMB4
KLF9	NFKB2	DNASE1L3	MLLT4	PSMD12	TMEM9	KIAA1429
LDLR	OR10Z1	ERICH1	NKG7	RAG1	TOP1	LCE2B
LIF	OR4F6	FAM65A	OR11A1	RIPK2	UBE2D2	LINGO4
LRP4	OR6C70	FBXO46	OR2D2	RORA	WHSC1L1	LITAF

MAGEB1	PALMD	FLVCR1	PIK3C2A	SLC38A10	WRB	MED20
MS4A6E	PARP10	GAS6	POU2F3	TMEM17	ZEB2	MICAL1
NCR3	PLK2	GCM2	PPARG	TMOD2		MTA1
NDST2	PNOC	GMIP	PTCH1	TP53I11		MYH13
NEU1	PXK	GRIA4	SHKBP1	UBE2N		NALCN
NFATC2IP	RASGRP3	GSG2	SLC24A3	UBE2R2		NBPF3
NLGN3	RAVER1	HADHA	SMPDL3A	UCHL5		NHP2L1
NR5A1	RNF125	HIF1AN	STK38	USP1		NPHP1
OR4F17	SFXN3	KCMF1	TRIB1	USP51		NXPH2
OR4K5	SHOC2	KCNN2	TSPAN2	ZNF554		OXSR1
PALMD	SLMAP	LDOC1L	UHMK1	ZNF740		PABPN1
PDE10A	SMARCA2	LMX1B	UQCRFS1			PLXNA2
PFDN6	SMPDL3A	MAG	ZNF132			PPM1M
PI3	STK38	MAGEB1	ZP1			PSD3
PITX1	SYT14	MAP3K11	ZSCAN2			RAD23B
PLXNA1	TRAFD1	MLLT4				RGMB
POLE3	TRIM54	MRPL49				RIPK2
PPP1R12A	TRIM67	MSH5				RPS6KB1
RAG1	TTC4	MYF6				RYBP
RAP1GDS1	VAC14	MYO9B				S100A7A
RAPGEF5	VCAM1	NEUROD4				SASS6
RHOA	WHSC1L1	NFKB2				SCD5
RHPN1		NHP2L1				SEMA4G
SLC12A1		NKG7				SEMG2
SOX8		OR4F17				SENP8
SRPK3		OR4K5				SLC1A1
SYPL1		ORAI3				SLC38A8
TFE3		PDZD8				STX17
TLX2		PECR				TBC1D2
TMEM98		PIK3C2A				TGDS
TRIB1		PIWIL1				TK2
TRIM28		PPP3CA				TMEM183A
TRIM54		PSD3				TMPRSS11B
TRIM60		PTCH1				TOPORS
TRUB2		PTPRK				TP53I11

TSPAN2
UBE3C
USP1
WAS
WFDC8
ZNF132
ZNF418
ZNF583

RAPGEF5	
REV3L	
RNF125	
RPL10	
SGK2	
SLC18A2	
SLC25A28	
SLMAP	
SNURF	
SOX11	
TESK2	
THAP10	
TLX2	
TMEM189-UBE2V1	
TMPRSS4	
TNF	
TRPV4	
TSPAN2	
TYRP1	
UBE3C	
UGT1A1	
UPF2	
USP25	
XPA	
ZNF593	

TRDMT1
TRIM21
TRIM56
WRB
ZMYND11
ZNF485

Supplementary Table S6: Overlaps between shRNA hit loci/proteins and proteins bound or depleted at nascent chromatin

**[shRNA] and [Nascent Enriched]:**	**[shRNA] and [Nascent Depleted]:**	[shRNA] and [Forks]:	**[shRNA] and [Chromatin]:**
ANXA1	AAAS	ANXA1	AAAS
API5	EMD	ASF1B	EMD
ASF1B	SLTM	ATAD5	LEMD2
ATAD5	SUN2	BAZ1B	NOL10
BAZ1B	PHIP	BRD2	PRDX4
BRD2	MBD1	BTAF1	SLTM
BTAF1	NOL10	CDK2	SUN1
CBR1	PRDX4	CDK5	SUN2
CDC37	SAFB2	CFDP1	UTP23
CDK2	SUN1	CHD1	ZNF22
CDK5	ZNF22	CLSPN	
CFDP1	LEMD2	CSN2	
CHD1	UTP23	CTBP1	
CLSPN		CTBP2	
CSN2		CUL4A	
CTBP1		CUL5	
CTBP2		DHX40	
CUL4A		DNMT1	
CUL5		DUT	
DHX40		EHMT2	
DNMT1		EYA3	
DUE-B		FOXK1	
DUT		GCFC2	
EHMT2		HDAC2	
EYA3		HELLS	
FOXK1		HIC2	

GCFC2
HDAC2
HELLS
HIC2
HMGB3
IPO5
IPO7
JADE3
KIFC1
LDB1
MBD3
MCM10
MCM2
MCM5
MCMBP
MED1
MED20
MMS19
MORC2
MSH6
MTA1
MTA2
MTAP
MTBP
MUTYH
NACC2
NAMPT
NR2C2
NSL1
NXN
ORC5
OTUB1
PCBP2
PGK1
PGP

JADE3
KIFC1
LDB1
MBD3
MCM10
MCM2
MCM5
MCMBP
MED1
MED20
MORC2
MSH6
MTA1
MTA2
MTAP
MTBP
MUTYH
NACC2
NAMPT
NR2C2
NSL1
NXN
ORC5
OTUB1
PGK1
PGP
PHF14
PML
PMM2
PNKP
PPIL4
PPM1G
RAD50
RAD9A
SCAF8

PHF14
PML
PMM2
PNKP
PPIL4
PPM1G
PTBP1
RAD50
RAD9A
RTCB
SCAF8
SCML2
SET
SIN3A
SMC2
SMC3
STAT1
TBCB
THOC6
TLK1
TLK2
TOP1
TOP2A
TRUB1
TTC4
WIZ
XPO5

SCML2
SET
SIN3A
SMC2
SMC3
STAT1
TBCB
THOC6
TLK1
TLK2
TOP2A
WIZ
XPO5

Supplementary Table S7: overlapiping hits between three cell lines

DF/myc(Pu/Py)58/CMV-TK	DF/myc (CTG)100/CMV-TK	[DF/myc(CTG)100eGFP/TK] and [DF/myc (CTG)100/CMV-TK]	[DF/myc(CTG)100eGFP/TK] and [DF/myc(Pu/Py)58/CMV-TK]
ADRB1	PAH	AAAS	ABCG2
DDX20	IRAK1	ACVR2B	ACTG1
CD2BP2	NDUFB7	AGBL1	ADAMTS19
CT45A4	LGMN	AGPAT9	AFF1
G0S2	PIK3C2B	AMELY	AKTIP
UBE2C	KIR3DL1	ANKRD13D	APOA1BP
SFTPB	ADAR	APOL6	ARHGDIG
LYPD6	MS4A13	BTF3	ARID2
C7orf76	PHKG2	C22orf24	ARPC5L
BZRAP1	RPS27L	CCDC92	AURKC
COPS2	FGGY	CCK	BPIFB2
SRSF8	COPS2	CD37	C12orf45
TTLL4	IFIT1	CEP250	C12ORF5
RGS16	LNPEP	CNIH1	C1orf198
RMND5B	UBE2C	CRADD	C1orf229
SLC16A4	SRSF8	DAGLB	CA3
HBB	COMMD3-BMI1	DBH	CACNG3
GRID2IP	FHAD1	EGFR	CALCOCO2
TMEM202	NOX5	ETFA	CAPN9
CHPT1	TDP1	F8A3	Ccdc39
HDAC9	DUSP4	FGF13	CDR2
LOC101929857	SLC40A1	FGF18	CHIC2
CCDC148	CLDN4	FITM1	CRAT
UQCRHL	PCDHGA2	FNDC3A	CRTAP
PGPEP1L	C7orf76	GABBR1	DALRD3
PANK1	NEUROD4	GIP	DBR1
ADAR	TSPAN11	GRXCR2	DDX51
SHMT1	EFR3B	GYG1	DISP1
RBM34	KLF16	HIPK2	EPS15L1

LDHB	HBB	HOMER1	ERGIC2
SSPN	NT5E	Hrct1	ESM1
HNRNPA1L2	ZHX1	HTR1D	EVA1B
CABP5	LBX1	IFNA1	FAAH2
ARG2	PLET1	IQGAP2	FAM101B
PAK4	CARD6	IRAK1	FREM3
MYOG	MCOLN1	ITGAD	GAGE4
KLK9	PITPNB	JADE1	GOT2
DDX27	FA2H	JAG2	Gpank1
ERC1	DIS3L	KIR3DL1	GPATCH2L
TAGLN2	ZNF625	KLHL6	HK3
CTBP2	KRBA2	LCAT	HMGB3
VCPKMT	B4GALT4	LCN2	HRASLS5
MTRNR2L10	G0S2	LOC101928268	HSPA1A
ARSA	NCAM1	MFI2	IL2RB
SHISA6	TMEM221	MMRN2	KCNE2
MS4A12	SNRPB2	MPO	KIAA1045
SUV39H2	LIM2	MROH5	LINC00684
NPM3	CBX6	MRPS6	LOC100996707
CSNK1A1L	CPSF4L	MSH6	LOC101927989
RNF167	CNIH1	MTCP1	LRFN1
MUC3B	MANSC4	MTERFD1	LRRC48
HSF2BP	PPP1R1B	MTERFD2	MACF1
BFSP2	SPATS1	MTG2	MAP1B
SAMHD1	AMPD1	NDUFB5	Ms4a15
ECI2	OTX1	NLK	MTRNR2L10
SLCO2A1	SLC31A1	OCM	MYBL1
EMX1	ORC1	OMA1	NADK2
PC	ERVFRD-1	OR2C1	NAT16
KTN1	ZNF362	OR4F15	NAT8B
SEBOX	NOTCH2	OR4M2	NEK9
KCNK12	CD8B	OR52B6	NELFE
Cdk14	GEMIN5	OR5AP2	NPM3
C6ORF165	NOL3	OR9G1	NPY2R
UBAC2	PAX6	PAGE1	NUP62CL

NMU	TMEM30A	PATE3	OASL
FEN1	PRY	PBX1	OR4K5
ANKRD13A	PTK2	PCCB	OR5B12
CHFR	C1S	PCDH19	OR6K3
TM4SF18	Mul1	PDE10A	OR7A10
DALRD3	RAB35	PDLIM2	PAK4
HSPA4L	JAG2	PIK3C2B	PEX11B
NPY2R	LOC100652871	PITPNB	PEX5L
IL17RA	NUMBL	POLL	PFKFB1
LOXL1	RPIA	PRPF4	PKLR
ARID2	SPEF2	PSMD9	PLA2G1B
TAPT1	GPR153	RHPN1	POLR3G
MPP4	HDAC9	SH2D1B	PPARA
TMEM234	RAB26	SLC13A5	PRTN3
WDR91	EXOC3	SLC1A3	RABL2A
CACFD1	TAF7	SLC2A11	RBBP5
RCN2	MET	SMCO4	RBM34
NEURL3	OCM	SNAI1	RFESD
FOXS1	Ddrgk1	SOX8	RHOF
CACNG3	SULT4A1	STK17B	RNF150
NAE1	DGKQ	TMEM165	RNF169
AHSA1	ACOT11	TMEM98	RTFDC1
MEP1B	OFD1	TRAF3IP2	RXRA
GPR45	FGFBP3	TROAP	S100B
DEGS2	KLF3	UBE2R2	SERPINA6
C12orf73	CISD3	USP1	SLAMF6
PNOC	CSPG5	VAMP8	SLC22A2
ZNF830	C15orf53	VPS4B	SLC25A53
ENKD1	HORMAD2	WDR72	SLTM
PLEKHA6	ST3GAL6	ZMPSTE24	SMIM15
C15orf61	IL10	ZNF418	SND1
SLC2A9	LOC101929758	ZNF8	SOX30
DGAT1	H2AFX		SPTBN1
PDCD1	CCDC125		SQLE
BEX1	CBX2		SRP72

PEX5L	NR2F1
SLC33A1	NPR2
Septin 5	LRP2
IL22RA1	TTK
SLC22A2	WDR89
ZNF891	SLC16A3
NME1	FAM98A
TSGA10IP	TAGLN
LGALS16	ZSWIM8
TVP23C	EPG5
METRN	HPN
MUTYH	PAX9
OVCA2	TNNT2
IDO1	LRRC15
BDKRB2	DNPH1
KRIT1	OR2F2
TP53BP1	IGF1R
ARMC1	CYP2C19
CRYGB	ACOT7
LOC100130357	TEFM
PXDC1	ZNF862
ZNF425	POLN
FAM180B	ASCL5
TRIM49C	SH3PXD2B
C1orf168	Tpra1
OR2F2	OSBPL6
NUPR1	ADCY8
LY6G6D	LOC100996337
AHSA2	IDI2
NUDT11	LYL1
SSR4	ZCCHC4
FAM57B	AP3S1
LTB4R2	NDEL1
IRX2	TMEM134
HSPB9	LDB2

SRPK3
SSMEM1
STK19
TAAR9
TEP1
TGM3
THBS2
TIMM44
TMEM191B
TMTC3
TRIM28
TRIM38
TULP4
TWF2
TXNDC15
UBE3C
USF2
WBP4
ZMIZ2
ZNF551
ZNF555

GGCT	XYLT2
SLC4A8	KIAA1147
ETFB	ALDOB
FKBP15	PSKH1
TOB1	C3orf83
NUP35	LOC100653515
MRGPRE	SUMO1
ERBB3	OR5AN1
SEMA4F	STX4
PPP6C	VIL1
KIAA1328	ANKFY1
NKX2-1	EDNRA
PKIA	NDUFB5
ACOT12	PGM2
LENG9	HGFAC
FAM135A	MOGS
PHOX2A	PRKAR1B
KAT2B	F8A3
RPL8	SLC18B1
ZC3HAV1	MED26
NUTF2	SGIP1
KIAA1191	PDZRN3
SPARC	NFIC
NOTCH4	VN1R4
ERCC4	HIST2H4B
LOC101060236	Ankrd36
RTFDC1	ZC3H12B
SPOCK1	OR8H3
CARF	OSBPL10
CATSPER2	ANXA4
ABCA13	CUL7
Hbq1	CCNDBP1
GPALPP1	OR2L2
CXorf40A	LUZP1
Rgsl1	TROAP

C1GALT1	ETFA
ACTL8	PPP1R14A
IGSF9B	BNIP1
ICOSLG	ASB2
LYPD1	SLC41A3
PCYOX1	OR5AP2
ZNF441	S100A2
LRRC14	SLC35F4
BTC	POLL
PPIAL4D	ULBP1
KLHL3	PBX4
LOC100292952	LEP
ATG4D	RBMS3
SDHA	GALK1
AKAP5	ARL6IP6
WBP4	PDE10A
HMGB3	SPAG9
NSUN3	CT47A8
SOX30	SNIP1
NAT9	OR10Z1
IDI2	KCNK13
TAOK2	SEC14L1
SEMA6D	NDUFA5
KAZALD1	TM9SF1
IQGAP1	MRPL23
FEM1B	FGF13
CIITA	ULK2
RAB2B	CDPF1
FKBP1A	LSM3
ANKRD46	KRBOX1
HAPLN3	CHRNB2
FUK	PSAP
TEX13A	TEX13A
KRTAP13-1	JADE1
NAMPT	ODF3

MAN2A2	MRGPRF
FAM118A	FAM135A
CNOT4	CRADD
ACAT1	ZNF837
FUS	NNT
EPCAM	NFRKB
KRT78	HNRNPA1L2
UPF1	CASP5
LRRC24	KCNV1
DGKG	VANGL1
PQLC1	TRIM64
Tada2b	SLC39A14
CEACAM16	PLP2
PTPRQ	PADI6
GTSCR1	TMCC1
TMEM251	NDP
BAGE4	POLR2F
KLHDC7A	UGT3A2
RNF186	PCDHB1
OR4F5	TNP2
SLC24A3	TRAPPC1
LOC101927403	TOM1
ATP6AP1L	LOC101928268
MTMR2	RSPO4
RGS10	PBX1
IL2RB	CCDC67
EIF4EBP2	GALNT18
CCDC180	LOC101928728
TMEM92	ARHGDIB
CPA6	SERPINB4
GPR82	KIAA1328
EMC3	SLC2A7
PDXP	OR4D6
LIG3	GSDMA
MEF2D	TMEM74

PAQR9	LOC100130357
SLC22A8	C20orf196
OR8D1	TOP2A
PIWIL3	DDX25
FOLH1	SOX6
NSA2	ANKRD13B
TCEAL7	CCNC
CRYGD	GHSR
CNEP1R1	DDX18
SELPLG	MED19
TICAM2	ZNRF3
EXO1	GLRA1
ELK3	PCDHB16
NPRL3	TALDO1
RNASEK	SYNGR2
WISP3	FOXP3
CELF1	C3orf22
Jkamp	CDS2
ABCC12	FAM173A
RPL41	PLEKHO1
UBAP1	ZNF157
LOC100507462	PEX3
PDAP1	C10ORF67
NUTM2B	ARL2
POU2F3	GABBR1
PRTN3	MUC15
GNL1	TIMELESS
FBXO6	ALKBH8
TALDO1	IL17RD
PICK1	C12orf56
HS3ST2	FOLH1
RBAK-RBAKDN	VGLL1
DDX18	COL1A1
PCDHB12	SEMA6D
PFN3	Pirt

KCNJ3	KDM5B
HERC5	TMEM62
PFKM	OR51I1
SLC22A17	LOC101929490
CYP4F12	NACA2
MB	ZMPSTE24
SIGLEC1	UBN2
C10ORF128	PIF1
IKZF4	ZNF418
SSR1	MKL1
BEST1	SIGLEC11
ANKRD2	CCDC180
CDH15	CDKL1
CD5L	MAPK1
SPINT1	SMIM2
RGPD6	CAST
DEFA3	NT5DC3
FLOT2	EXOC3L1
OGN	DDX11
RAX	SHROOM4
PXK	STK17B
STXBP2	MTCP1
DKK1	EREG
IMPAD1	C9orf173
SESN1	AZU1
ZNF567	OMA1
PEX11B	SLC51A
Kdm4a	DCHS1
NGLY1	SLC34A2
PEX3	OAZ3
TSTD1	DGKK
NAT8B	CEACAM16
SLC30A2	VSNL1
EBP	MRPS6
GTF3A	CTNND1

LPPR5	ZNF101
RPP38	MORC2
CCR9	TRIM27
LMO2	CIART
CMTM2	ZNF665
CAPN9	DEFB127
APPL2	MYO1H
KIAA1244	ZNF573
SPAG6	BANF2
SMIM22	LOC101928120
C11orf21	ANKRD13D
SLC28A1	OGT
HMBS	FBXO15
LOC101930073	UST
SENP3	FAM161B
TMEM218	TM7SF2
KLF7	HAS2
KIF18B	MAGEC1
PIK3IP1	AGAP7
ASAH2B	LLGL1
ZSCAN18	BLMH
ZCWPW2	WDSUB1
TMEM55B	NPR1
PXMP2	SLC25A29
ZNF136	TASP1
KCNH6	C3orf84
NANP	CALD1
OSTCP2	FAM213B
CHST10	AFP
NOTCH2	MAGEA3
RASGEF1A	ZNF112
DMXL2	ASGR2
PFKFB1	HSD11B2
CD59	GPR65
C4orf45	LHCGR

RASGRP1	SAMD4B
IL1RAP	RPS8
TTLL12	HSPA12A
EEF1A1	LCK
TNFSF15	HS3ST6
PTGES3	WNT3
GPR160	STAT1
TMEM183B	PAXIP1
PPP1R3C	ANKRD42
LOC101930154	HPRT1
PTX3	KIF15
SPATA19	DNAJB1
IGF2	ZNF844
ARHGDIG	ZNF350
ZNF142	KLHL36
TYW1B	HMGCLL1
RFPL1	CAPN3
PTGDS	TGFB1I1
HECTD2	WBP1L
OR8J3	BMP4
ARHGAP4	OR4A16
TMEM253	UBE2U
KDELR2	TMPRSS11A
RABL2A	P2RY2
MXI1	CTC1
DCBLD2	COX16
ANGPTL6	TCL1A
SLCO4C1	FBXL17
LOC101930295	APOL6
NPIPA7	PDP2
DUSP11	CD44
TRAPPC5	CA7
ACOX3	ZNF236
UVRAG	HTR1D
CHD9	BAGE2

LCT	RNASE10
ELK4	HSPB2
ARC	AARSD1
RPS6KA2	MUC8
PAPPA2	PRKAB1
SERPINC1	ZNF595
ABCD2	WNK2
WNK2	PFKFB3
Uba5	Manf
NBN	STON1
SULF2	NALCN
MFAP5	PAX4
SLIT2	NTMT1
MGAT5B	GFPT1
TMEM59L	CD97
PPP1R3E	FNDC3A
BIRC8	XRRA1
LOC339862	IL22RA1
SLC35D2	COIL
POU3F3	IGF2BP3
SGCD	PALB2
TXNDC9	TSSK4
TRIM64C	ACAN
PRELP	NEURL1B
TMEM163	FAM53C
Rnls	C19orf33
SLC22A11	GALNT3
IRGC	LOC101930279
RND2	TDP2
NLGN4X	LOC100506374
RABGEF1	TLX1NB
XRRA1	ITGAD
TMEM51	EIF4G2
OXLD1	ASCC3
ZNF823	LXN

SLC15A4	NOTCH1
RXRA	RCN2
GRPEL2	LRRC49
HSBP1	IL1RN
NR1D1	AKNA
SLC19A2	Uba5
ALPL	OR10J5
CHD8	RAP1GDS1
FASTKD5	MTFR1L
NEDD4	IFITM10
RNASE8	AMPD2
TFF2	PDGFC
SAMD14	OR51A7
MAT2B	HRNR
PNMA6A	LHFPL5
AQP1	B4GALNT2
MID1	RUNDC3B
MLX	CD8A
MAP3K9	SLC1A3
GCN1L1	VAMP8
ATOX1	KIAA1958
IQCJ-SCHIP1	EEF1A1
ANP32E	VCPIP1
CXCR4	COL27A1
KRT84	CST2
FBXO42	PXDN
FAM83A	DPT
FAM76A	FGF14
CA3	MPHOSPH9
OLFM4	PRR20D
EQTN	CHUK
TAT	PNLIPRP1
HIST3H3	ENOPH1
DNMT1	ADI1
SLAMF6	PRKACB

CNTF	FAM102B
USP38	FXN
POLB	CACNG8
ZNF774	CIITA
SFRP5	TACR1
FAM46B	CA8
RAB30	IFNG
CAPNS1	VPS33B
FBXO28	FICD
PCK2	C12orf60
SCN1B	MRPL50
RNF113B	CHN1
TMEM170A	ACR
RHOT2	CERK
POPDC2	Prr24
PRKAR2A	JAGN1
ZNF720	PCNXL2
ADAM33	PCDHGA4
HMGCLL1	TNKS2
CCL8	POC1B
TNFRSF21	FPGS
CLDN3	NLK
FAM175B	MAFB
HSP90AB1	TG
LACC1	C7orf55-LUC7L2
XYLT1	SEMA4D
HHIPL2	KIAA1244
MICU3	CCDC142
ICA1	MCF2
LOC101928548	OR7A17
FAM170B	SRPK2
SPATA4	RS1
Ak4	NDFIP2
LOC100505478	ZNF189
CELA2B	GEMIN7

ENO2	DEDD2
PGLYRP4	DUOX1
FAM110D	RBBP4
SHC4	FUT8
SFT2D1	LAS1L
TAF3	PPP3R1
MYO1D	GSG2
ANAPC10	KLRG2
CLC	ZFAND2A
GLRA4	OR52N5
TEX12	CCDC58
C8orf33	TBCEL
SHOC2	NOL4L
CLIC5	FAM111A
LCN9	GABRB3
C3orf30	PEX13
Tet3	IL18
TTC4	GOLGA6L1
PTPN1	OR5M3
KLF10	CFH
TRIM61	PSMB5
TM7SF2	GNG7
ZNF674	RAP1A
USF2	LCN10
CDR2	ERP29
KRTAP12-4	MFRP
CASP5	LAMA4
ATP5G1	NPEPL1
LMNA	ASB12
NFE2L3	SNRPB
EPO	NPIPA7
LOC100293211	ITGAE
RNF169	SCN1B
MAS1L	SMAD6
C7orf62	LOC730183

AMPD2	SLC25A21
ADAMTS19	CACNG6
CLPB	PMVK
NR0B1	WISP2
LOC401052	UBE2R2
LCN15	CTSW
MRPL43	PCDHB3
PRRC2A	DNM1L
ZNF283	CCDC179
ABHD17C	PLCG2
DNASE1L3	CXCR4
PSMB5	IL10RB
CCDC108	PEAK1
GALK1	C19ORF26
TMEM121	RAB33B
ANKHD1	PDIA6
Plac9	COPG2
CMTM8	COX4I1
KDM5C	SOS1
MFSD9	WWP1
PTGDR	FITM1
POLR2J2	HMGB1
HAO2	ERVV-1
PCDHGB2	FBXO28
OR10J5	MCAT
RSPO2	PRR16
NLRP12	TAS2R38
OR14C36	TFCP2
RPTOR	KIF21A
SMUG1	HOMER1
PRKD1	Srsf6
FAM71F2	MCF2L
STEAP4	LOC100996721
BAG1	ASB11
CYP4A22	KIAA0141

SUSD2	LRCOL1
CASP6	PRTG
PLA2G4C	CUL4A
C10ORF129	DLG4
WNT4	ZNF274
SLC25A53	AVL9
SCRT1	CD74
NOS3	TM4SF19
LHFPL5	KHDRBS3
CCL7	MYC
Ms4a15	PCDH19
ZMIZ2	POTEF
FAM101B	CYP46A1
ARIH2	CD48
MALT1	CEP68
RAB26	LOC554223
MFRP	SFXN1
SIM1	RPL36A
NCF2	RAB30
CRYAB	ZNF90
ZNF341	PLAG1
VGLL3	MPP4
PMP2	NIFK
ITPKC	TM6SF2
GTF2H2C	NDC80
GPR89A	C7ORF33
CELA3B	OLFML2A
CEACAM20	DBF4B
ZNF793	MYH15
CGNL1	VSTM1
Sap25	LYZL1
CCDC68	CFI
MSANTD3-TMEFF1	CCDC51
NEBL	PLAC1
TOP3B	FDX1

BTN2A1	DOCK8
ZNF461	CREG2
ATG7	ARL13B
RPS10-NUDT3	LCE3E
SIN3A	KLHDC10
ATP6V0E1	SPTBN2
SND1	MTERFD1
EVI5	GLI1
ZZEF1	SEMA4G
COL23A1	TRAF3IP2
LRP5L	SPINK5
RLBP1	PRAMEF4
PDZD8	IMPAD1
PRICKLE3	KAL1
RSPH1	FGF20
PLD4	AXIN2
Dnah12	KLF12
AMY1C	POM121L2
TIMM44	CEACAM20
RNF152	COG8
LOC101927029	FCGR3B
CEP104	ATP5G1
MBD2	Minos1
ATCAY	JADE2
FBP1	SOD2
ZRANB3	SBK3
CLUAP1	ACBD6
KIAA0930	SH2D1B
SIX3	EIF5
DLGAP2	C6orf99
NCKAP5	AQPEP
CCL22	PLEKHH3
EID1	KRT13
TAAR9	GJA10
OR13C2	PSMD9

FAM122B	MMRN2
ZC3HC1	PLA2G12A
LOC149373	GNLY
SOX3	AGPS
S100B	KRT39
GPATCH1	LIPJ
FNTA	CGNL1
GCAT	LOC732265
C14ORF105	AKAP10
LRFN1	CPEB3
SMIM7	LRRN4CL
POU4F3	FOXD4L3
TEX101	ENPP4
NAV1	CTAGE4
ZNF331	RPS20
OR5H14	SIRPA
ATXN3L	SLC2A11
SHKBP1	BRE
RFTN2	AKR1B1
ZDHHC6	EFHD2
TRIM48	CWF19L2
RPLP0	PHLDB2
LMO4	KIAA1751
KCNJ2	OR6C74
PEX5	STARD10
ADCK4	RTN4RL2
CDS1	PDE6H
ZDHHC23	ZNF439
ODF3	FAM214B
PGP	GSTK1
PRDX4	SLC6A3
MAP1B	CCR10
TGM3	TBC1D7
FILIP1	RASL10A
CETN3	TXK

CTSC	USP10
MFSD5	LOC101930586
ZRANB1	GPA33
RPS19	GNPAT
PPP1R8	ERN1
ACAA1	CHST13
TMPRSS11B	PAXBP1
RFPL3	CMC2
ERCC6-PGBD3	MSH6
RHCE	REPIN1
SNAPC2	ASTL
MYH4	INSIG1
SETD5	TRIM39
OR4K5	CRYM
SGCG	TTC23
NDEL1	SLC25A22
GOT2	LRFN5
SLC7A10	C20ORF24
FAM83F	CLU
TLX1NB	GIP
ASGR1	FAM153B
TFF3	MTERFD2
HBP1	PDE7B
MRPL55	PPP4R1
C6ORF118	HS3ST1
SLC15A2	MTG2
GPR37	CD33
STK32A	TOR1AIP1
MED23	HTATIP2
LIPJ	OR4D10
PELI3	DNAL4
EGFL6	MS4A7
LOC101929972	AGPAT9
TMTC3	FAM188A
EIF3H	ZCCHC11

OR4C16	ARPP19
C17orf77	PCCB
SVOPL	CLEC12B
C14ORF79	PXT1
ADAM9	ZNF480
PHRF1	SAMD12
CSGALNACT2	LNX2
GPI	CTSS
CERS3	FBXO10
SERBP1	KCNA6
UGT1A9	TM9SF4
KIAA1045	CMKLR1
TM9SF1	MFSD2B
ASL	CTCFL
PDZD3	MS4A14
GZMA	PCGF6
Sgk1	TMEM253
AKAP10	TOB2
CARD9	VGLL3
KIRREL3	FBXW8
PHF20	ZNF180
OR6N2	CCDC22
NEDD4L	PLEKHA4
DSCR3	PLBD2
POLR3E	MPC1L
C2orf27B	AADACL4
ATP5C1	KRTAP10-11
TUBD1	PCID2
GPHA2	MEF2B
Tmx2	LCAT
C21ORF2	THUMPD1
MAGI2	TRPC4
C1orf115	RAP1B
CENPQ	NAT2
MAML1	TBCC

OR5C1	AMER3
ADCYAP1	RBL1
Vsig10	PELP1
CARD17	ZAR1L
CALCOCO2	ZBTB7B
ARPC5L	PCP4
SERPINA6	LAP3
ZNF589	RUSC2
TESK1	COL9A1
APOBEC3H	S100A4
SH3TC1	AMY2A
MEGF8	LOC100506859
GINM1	DHRS7
NCL	IRX1
CRYZ	MMAB
NFKBIE	RABEP1
RNF168	LGALS3
H2AFY2	FBXO22
COASY	TECTA
ZNF398	CLCN6
MANBA	CCK
FTH1	YIPF6
RFESD	EGF
MYOZ1	C3orf35
COX14	CCNT2
MMS22L	MX2
SLC7A4	GP1BA
MRPL36	MYO3B
SOX12	POLR3K
TRPA1	CRISP1
SLC2A5	Ubxn7
AKTIP	IL1R2
LOC100996415	CLVS1
C1orf101	SLC35A3
CREBZF	CCDC6

CBX8	MAP3K13
C19orf52	SPG7
GAMT	NR3C2
TCP10L2	IL31
B2M	LRRK1
DCLRE1C	ARFGEF2
CHIC2	TBC1D27
GATAD2B	TELO2
OR3A3	SGCA
USP16	SMCO4
FMO3	NSMF
Clvs2	TCERG1
ST8SIA6	TBC1D20
NOS1AP	RABIF
GAR1	CBR4
OR10K1	DPY19L2
HIST1H2BK	CCDC23
CSTB	TNFSF12-TNFSF13
WDR52	NME4
OR5V1	LHX1
FBXL16	OR4M2
EXOSC1	ADAT1
ARNT	FGG
SKA1	PITRM1
LOC101929220	KLK4
PACRG	SPECC1
ADPRH	ASIC5
MTX1	ZNF888
MRPL45	DHX16
PCNXL2	FGF18
KRTAP4-11	YPEL3
FER1L6	MPP2
ADM5	VSTM2A
LIPC	PATE3
OR6M1	HOXB3

TMEM168	KCNJ15
WAPAL	H2AFY
SCARB2	GDF9
ZEB1	GTF3C6
SLC31A2	ACVR1
FJX1	OR4C16
EVA1B	ADRBK2
DMRTC2	PPP2R1B
CCL3L1	PLCD3
TERF2IP	RBPJ
DYNC1LI1	SLC22A15
MTMR9	RPSA
DOLPP1	TCL1B
KCNE2	TGFBR2
PREP	STK32B
C2orf42	TNNC1
FAS	MRGPRE
AGAP5	OR52B6
CRABP1	LPAR4
PLA2G12A	KRTAP29-1
GUCY2D	AMHR2
GSTA2	MARC1
USP21	NCKAP5
SIN3B	SLC45A4
GPR162	HBEGF
DUSP18	ATG14
MIER2	OR4F15
SLC6A17	PAGE1
FRMPD1	LMNA
TOMM22	EIF3H
SMIM15	MCF2L2
MAPT	OR9G1
GNAL	DRG1
SP6	TMEM205
ERCC1	GOT1L1

CMAS	NUDT16
KAT5	HLA-DQA1
NLRX1	FAM166B
CCL18	TGM6
VAMP4	Septin15
TRIB3	FNDC4
BPIFB1	CHTF18
MTMR7	GMPR2
PHF10	Cisd2
OR51Q1	PGM3
LOC101930432	RNF149
KCNC2	SEC14L2
RIMKLA	TMEM165
RAVER1	C10ORF129
APOOL	TMPRSS6
HPS5	CD3E
TAS1R1	FAM110D
MTA2	NUAK1
PLEKHN1	UBE2M
CLDN2	STAB1
CKS2	AGPAT6
ZNF527	RGR
CELA2A	CCDC154
IZUMO4	ACAT1
PELP1	FGF7
KLF4	PROKR1
RARS	LOC101929519
LCN8	ZDHHC5
LOC100996707	TXNDC9
BSPH1	ITLN1
HSPA1A	AK6
DNAH11	LOC101929857
ENTPD8	AMICA1
DEXI	NDOR1
ADAM11	C11orf68

PRIM1	NKPD1
TAF15	ASB4
GTF2E2	MSX1
RAP1GDS1	TMCC3
ITIH6	SCIN
FCGR1B	RRNAD1
LPAR6	ADAM20
CADM4	CYP27B1
MTIF2	NOTCH4
LOC390937	ZNF408
TAF1	OR56A4
BPIFB2	IRF9
CDCP2	ZFAND4
ATF7	RDH16
Atat1	SLC2A14
NAT16	IRGM
BTAF1	TRIM61
KRTAP12-3	SLC11A1
SPAG1	C14orf182
CRTAC1	CIDEA
INHBE	ENTPD1
CNTD2	ZNF341
SLC35A1	PNPLA8
PCBP3	LRRC4B
TNFSF18	OR2A7
ARL6IP6	SUDS3
ZSCAN1	TH
ZKSCAN4	WNT9B
SKIDA1	LOC101929876
GLYATL1	THUMPD3
TP53I3	BOLL
SPACA1	FZR1
NIFK	OR1L6
THBS2	SFRP4
PSMB8	FOXF1

LOC388282	ZNF441
TRMU	CCDC126
CAPS	DNAAF1
TMEM180	LRP12
CCDC27	C1orf177
CLU	PCDHB12
ERGIC2	FAM65C
NTNG2	RNF17
KBTBD8	ALX1
OR6B3	USF1
LOC101929989	PDLIM2
CHEK2	ENOX2
PARPBP	ARL10
CTSA	WDR5B
LOC101929393	SLC13A5
ATXN2L	CYP17A1
SAYSD1	SH3GL2
GFRA4	RRAD
KLRG1	CDH4
PEX13	KRAS
RCC1	C12orf61
RGL4	OR2A2
LOC101929766	PCTP
ZNF860	MTA3
ATP10B	ZNF704
NUP62CL	TNC
ZNF721	MCUR1
SRP72	HLA-DRB5
OR4D10	SLC35B2
TCN1	LOC101927850
SLC7A9	C3orf58
PFKFB4	B3GNT8
ZNF671	HEPN1
COMT	RASAL2
LRP2BP	LGALS8

FBXL18	HIRA
AMOT	GOLGA7
KCNA5	ZXDC
MAPK8	VWA5A
FANCD2OS	AAAS
ITGB1BP1	CHORDC1
SHROOM3	DAPP1
BRD4	KLHL6
GOLGA8B	GCNT4
DNAH8	CD207
ZBTB18	MEF2BNB-MEF2B
BAIAP2L1	TUBB2B
NDUFC1	NONO
KBTBD7	SLC25A43
WFDC6	AGBL1
PTGIR	C16orf96
CSDC2	DMXL2
NDFIP2	BRD7
PANO	PDK3
SERPINB9	IQUB
DHX16	Igdcc4
RNPEP	HSPB3
NRN1L	Kncn
TDGF1	MRPL36
EPS15L1	COL4A1
LEPRE1	ZBTB14
GUCA1A	EFHC2
SUMO4	BUB1
ST3GAL5	STRN3
SLTM	PHOX2A
CTNNB1	Cela1
HOOK1	ADAT2
TIE1	FRMD4A
OR6K3	SIT1
TRPV5	ATCAY

MTCH1	FAU
HCN2	KCNK3
GAGE7	CDC25B
ICAM1	OR8K3
GSTA1	GZMA
Rsg1	CDCA3
COIL	FBXW11
EFCAB7	PIGN
DRC1	FKBP1A
LOC101927050	ACVR2B
PAK3	KCNK4
LY6D	ASB6
PHLDA3	NGFR
SLC1A6	PDE1C
SH3BGR	SERPINB3
LRRC3	TNFAIP8L3
GRAMD1C	TMEM159
LRRC2	YAP1
RHNO1	DEFB132
COL7A1	LOC100996782
ZNF546	TSPY8
RGR	LOC729458
USP17L5	CCDC171
CCDC64B	C8orf58
HOXB7	MROH5
C2CD5	MMEL1
TMEM30B	ATP6V1G3
BTBD11	CREG1
LAT2	TSSK1B
SLC37A1	TMEM47
SIRT5	PRPF4
CRLS1	TRIAP1
MSLN	ACBD5
FREM3	FAM136A
METTL13	SCAP

DBR1	ABCA8
FAM76B	MTURN
DSG1	ZP3
TANK	CHMP7
TGDS	MNS1
MTO1	IL4R
PLEKHD1	BID
Ufl1	SPCS2
DPH7	HS3ST5
GCC2	ZNF491
ABCA9	PPP6R1
ZNF551	ID4
TFF1	TBC1D22B
MATN4	C8orf86
SLC34A2	FBXL12
TMEM132A	DBH
ATP10D	DGKG
SLC17A8	SLC12A4
GRK7	DDC
C7orf69	POLR2J2
XRN1	ADCY6
C20orf202	TJAP1
C12orf61	DEPDC1
CXCL13	TBC1D10B
CEP128	RNF14
NDUFA3	MAP4K5
VTCN1	NPAT
HBG2	LOC100506127
ANO5	NPPB
SOX17	PKHD1
AQP6	IFNA1
TMEM235	ABCC10
ORC1	ABHD14A
BID	SEC23A
PDGFA	SCN11A

GAGE12B	LOC285500
MIPEP	LOC646021
HIBCH	LOC101929864
GLRA2	RPS4Y2
ZNRF2	ASNA1
TLR1	IKZF2
POLR3B	LRFN3
CRK	ZNFX1
CNN3	GTF2I
SEC31A	SLC25A12
ACTL7B	YWHAE
HRASLS2	RGS18
STYXL1	C1orf210
CD28	HSBP1L1
ZNF140	OR51T1
LOC100130880	FAM180B
Scaf4	OR5K1
Tmem219	TUT1
DUSP5	LCN2
TARS	CDC6
LOC101928805	EIF4E2
KLHL1	MICU2
TNFAIP1	AMELY
SLC25A19	UBQLN4
PLCD3	C22orf24
SLC2A4	ZCRB1
Sp9	IMMP2L
TRAPPC2L	SLMO1
APOA4	MS4A6A
TAF1C	PDIK1L
PCDHGA9	WFDC10A
AURKAIP1	TEKT3
PDZRN3	SLCO2A1
Tmem237	ADRB1
MRPL49	DDX19B

FKBP10	EID2
FBXO2	KCNIP1
TMEM79	ZMAT5
HS1BP3	SORL1
CTBS	MPO
CSAG1	ICAM1
NGFR	CCM2
PPP1R16A	GABRA5
SNAPC1	Rab44
PPM1E	MRO
USF1	NIPBL
CRTAP	MSTO1
UROS	AVEN
NUP93	CT45A4
SERF2	TMEM120B
ERI2	RASIP1
ZNF492	GSTCD
BMPR2	SATB1
KCNAB1	NSUN3
HBG1	SYNJ2
CCDC3	CCBE1
FADS6	SP100
ZDHHC1	USP5
ZNF34	AP1B1
GNB4	C1ORF27
COX7C	UCKL1
METTL4	FKBP5
LIN37	LOC149373
MON1B	HAL
PPEF2	TMEM98
ODF3L1	RBM38
ARPC4-TTLL3	ADAM22
CDYL2	IRF7
MACF1	C10ORF76
ZNF397	NRDE2

MFSD11	FAM212B
GPSM3	ZNF695
SSMEM1	ADAMTS1
LOC101930392	IFNLR1
TCEAL3	EPHA5
AFF1	CAMK2A
TRIM50	ABAT
NRCAM	LY75-CD302
TAF13	TMEM182
RPL23	STMN3
B3GAT2	SPRYD7
TRIM52	MEGF8
PNPLA6	OTC
LBX1	HTRA1
PDPK1	A3GALT2
POLR3G	ACE2
RAB21	MTIF2
TMEM211	OR4B1
TRIM38	COG3
PYGL	TGFBR1
UBE2D4	C1orf56
PDGFD	KRT71
EFHC2	TPD52L1
SPINK1	SLC25A37
LOC101930235	CCDC92
RRAGA	PCDHGA1
FBXW12	TEX12
FXYD4	CHD9
KIF1A	LOC100652901
CYP4F2	OR1I1
ARFGEF2	PTGER3
C7	STX8
DEPDC1	KIF5B
ENAM	SMS
MTFR1	OR2C1

FAM47E-STBD1	IFT22
ZYG11A	KRT74
TICRR	ABCB8
C1orf137	OLFM1
TUBAL3	PLCB3
TRIAP1	KCNJ18
SRI	SLC14A2
NADK2	CPXM2
CCNT1	LRFN2
CYC1	DAZ3
RPA3	PRB4
CCDC177	FXYD1
SETDB1	POLR2J
NRSN1	EZH1
VSTM4	USP21
HMGXB3	ZNF502
ANGPTL3	PTK6
GPR114	FAM186A
CACHD1	SPON1
PPARA	GLOD5
DYNC2H1	SUFU
APOBEC4	ADAD1
CEP57L1	MIPEP
BMP5	FAM63A
BBS2	METTL7A
NRGN	PNPLA3
TAF11	GTF2A2
C7orf61	CD1E
GLI4	ACSM4
NFAM1	SLC35E2
OR2L8	HSPH1
IFNA17	CPLX2
TMEM178A	MFI2
CMA1	C1orf115
PPM1L	HPD

LOC101930441	GRXCR2
C14orf178	ZNF219
TAS2R30	SNX5
LOC101928282	COL19A1
ACOX1	PDZRN4
ACSM4	SLC50A1
TRNP1	APOE
MRPS2	MRPL4
SPRR4	DAGLB
Ralgapa2	PDC
ZNRF3	BRINP3
TMEM175	STX1A
PTER	GPR173
LOC388780	Hrct1
LOC101060580	HIPK2
AURKC	ARPC4
THYN1	ZNF8
TOM1L2	VEZT
CCDC154	ACOT12
PTEN	NUDT19
SLC20A1	CCDC94
KCNJ14	NDUFS6
TMLHE	TP53AIP1
LYRM2	ACSF3
KPNA7	ZRANB1
CPN1	HN1
COX7B2	KRT4
DISP1	MUTYH
MAP4K1	KLHL40
RTCA	AGAP5
SLC39A10	ANKRD10
OR4A15	CSF3R
CARD10	IFI44
C17orf99	IL16
TMEM18	CNFN

NTMT1	TSSC4
POTEG	ZNF714
BRAT1	ANK2
GPR64	TMEM229A
NPAT	Speg
ARL2	CEP250
AP1S2	VPS54
HSPBP1	PHLPP2
KDM1A	LIMD2
TMEM155	Dnajc27
CTDSPL	SLC22A25
PRDM14	ATP6V1E2
PTPRA	PMCH
GALP	LOC101927050
PPP1R35	FAM171A2
CORT	Ptpmt1
PLEK	VPS4B
DNAJC2	PIGR
ARID4B	WDR72
TM4SF5	LSM12
TEP1	CD6
TMEM214	PDE1B
SAMD11	DPP4
SLMAP	MRPL42
LEP	RNASEK
SURF1	HIST1H2BK
CYTL1	C1orf50
NLGN1	ATG10
IFI44	KLF2
DLX1	ZNF43
CACNG5	SOX8
ELMSAN1	SLFN5
SMCO3	TMEM107
ADAMTS20	PHYKPL
SPPL2B	Agap1

EFCAB5	C21ORF59
SPATA25	CNTN6
RHOU	C10ORF91
RNF157	CYP26A1
ALPP	LOC101928044
FRAT2	CRX
RNF43	CCR9
MAML2	MANEAL
METTL21C	KLRC3
CALCR	SLC41A1
GPR128	RAP2C
Fam174b	TPBGL
CORO1B	SSRP1
OASL	STX11
EML3	NR2F6
UGP2	ODF2L
PLA2G1B	EGFR
DIS3L	Ect2l
GPR62	C9ORF84
FAM189A1	DRAXIN
CES3	OR1J4
LOC100130520	C5orf46
ALDH1L2	BTF3
ASCC3	MYL6B
COMTD1	GYG1
TFG	C3orf55
SALL3	LARGE
CCDC63	PLS3
DEAF1	KITLG
CRY2	FZD9
SUDS3	ABCD3
CHIA	ATP6V0E2
KIAA1841	TENM1
SHISA8	MBIP
EFNB1	TSTD1

MGEA5	GDE1
TWF2	C7orf43
KRT6A	XPNPEP1
DNAJB1	TXNRD1
GLP1R	ZNF846
LOC101930059	KCTD18
PODN	EML5
STAM2	NXT2
FAM196A	PTGR1
HOXD13	FAH
C12orf45	ARFGAP1
SLC41A3	DOLK
RBBP5	SNAI1
WDSUB1	ERC2
STK19	ZZEF1
E2F5	EXO5
NCS1	TMEM215
KIF5B	UBE2H
AARSD1	ATP13A5
KISS1	P2RY12
UGT8	SLC1A7
STX1A	BTF3L4
HSFX1	RHPN1
ZGPAT	PLA2R1
LOC101930553	IQGAP2
FKBP7	NSMCE1
CARHSP1	SNTG1
SRPK3	AMER2
TCP11X2	ULBP3
C1QL2	HOXA4
TRMT2A	C3AR1
KLF5	OR5AK2
MRPS10	Nrg1
AQP10	CD37
SLC25A13	CANT1

ANKRD45	DOCK1
PGM3	OR1A1
ZFP41	DYNC1I1
CDKL4	HGS
Ubxn6	USP1
ALG2	DMGDH
GABRE	RNF181
HIST1H2AH	MICALL1
LOC100996412	
LOC100129520	
GTF2A1L	
ADD1	
COX5A	
DAK	
SLC44A2	
WWTR1	
RAB3IP	
ERVV-1	
RLN3	
OR5H15	
ACSS1	
CCL3L3	
SHROOM2	
IL1R1	
DDX10	
GCLC	
ARAF	
DCAF12L2	
PAPPA	
GPC5	
SLITRK6	
CRH	
LYPD3	
PPP1R1C	
PCDHA7	

MORC1
MYH6
TXNDC15
TCEB3B
OR52N4
BYSL
CABP1
ZNF619
CD164L2
KMO
MALRD1
TACR3
WDR83
RAB11FIP4
MCHR1
SPANXN2
EFCAB4A
WNT9B
GCNT4
PON1
TRAK1
SNX16
HSD17B3
PCDHGA4
SEC14L6
ZNF562
TXNDC2
PITHD1
DUSP22
Gpank1
HLA-DMA
DEFB129
LEPREL2
GDF7
BCL7B

PPRC1
GMFB
QRICH1
GAGE12G
SIX1
ZBTB48
PHACTR1
CDH11
PIGP
C18ORF8
RHOF
ZC2HC1A
PTK2B
OR7A10
RBM47
TNKS1BP1
SLCO1B1
GABRG3
BATF
SLC25A36
AKNA
LOC100653225
FBXO44
ZNF597
MARVELD3
CYP2U1
GTSF1L
E2F6
RIC3
MAPK7
SERPINB3
TUFM
HBE1
ASCC1
TUSC3

DDX17
NEK9
GLI1
SPTLC1
BARHL2
CXCL6
PLLP
MS4A14
TDO2
PPAPDC2
GNE
ZNF250
UCP3
TEX15
NGFRAP1
PSKH1
AMY1B
CBX6
C11orf35
TMEM200A
KIAA1024L
GH2
GSDMA
SLC35C1
DEGS1
APOA2
FAAH2
LOC101928486
GLRB
SH2B2
IRAK2
MTFP1
MAGED2
QSOX2
TGFBR3L

ALDH7A1
OR7C2
FMR1NB
STRBP
C11orf74
CLDN1
JAKMIP1
ABCG2
Synm
ANG
AMHR2
TMEM45B
PEX26
LOC100862671
SH2D4A
HMCN1
POM121
FOXD4L4
LOC101060181
HIBADH
ZNF705A
PLEKHO1
MUC15
KPRP
TLCD2
OR5B12
CAPZA2
LUZP6
ACTG1
TCEB3CL
AGPAT1
GMNC
PUM1
ACTBL2
HCRTR2

ETV5
IDS
BHLHA15
MVB12A
C12orf29
STYX
KCTD5
SQLE
CLDN15
FGGY
CCNI2
ZMYM1
ZNF816-ZNF321P
RBFOX3
AMOTL2
PCYT2
LIME1
ODF2L
ZSWIM8
P2RY8
PLA2G15
USP17L2
ZNF555
CLEC4M
RPS6KA3
MYO18B
ACRV1
ABCC3
KLHL40
MYOD1
MYLK3
GYPE
SLC25A44
LCE1F
PDLIM7

LOC101927850
CRAT
CETP
OSGEP
CCDC172
PRSS35
NPPB
HK3
VWCE
PSEN1
OR52N5
CCNT2
ZNF180
PAXIP1
AWAT2
PFN2
KIF1B
TMEM123
CASP4
IL2RG
TULP4
CDC14B
RAB23
SACS
DNAH14
Ccdc39
CREB5
STIM1
Fam181b
ESM1
PRKAR1A
STARD10
ACSL6
RDH13
CTCFL

ZC4H2
ZFP62
DOT1L
PTGR2
FLRT2
ZCCHC3
HRASLS5
CLEC18B
TRIM28
Dcaf8
ZBTB26
CTHRC1
TMPRSS6
LRRC10B
EGLN1
PKLR
TMEM59
MYBL1
TMEM191B
LEPREL4
TLN1
Ccdc138
NELFE
GPR22
AVPR1B
LIMK2
VIP
NPTX2
LIN7C
CTSK
NT5C
REG4
UBE3C
TMEM244
GPS1

PLA2R1
MTNR1A
SMIM4
HCST
DPY19L3
ZNF70
NUDT6
MAP9
LASP1
C19orf83
RORC
DDIT3
ZNF462
Acap3
CDCA3
TMEM249
WFDC11
SLC1A4
FXN
VN1R4
ANKRD60
ACSM3
NKIRAS2
FAM160A1
NHSL1
TRIM39-RPP21
C1orf198
BRD7
FHAD1
DCAKD
BICD2
C19orf60
STK36
C2orf76
TMEM177

NONO
STRN
SIGLEC8
PRSS21
CTDSP1
NMT1
HIPK4
P2RY13
EML1
IFT74
C5orf54
HINT3
PRDM1
GNRHR
SDE2
TMEM262
TAS2R9
CNOT2
MRPL15
ARNTL
Rnf144b
MAN1A2
NXPH4
NQO1
ZNF718
PCDHB10
KCNK17
ARMC12
C1orf189
AK6
PABPC5
PRR21
VN1R2
TMEM50B
FAM107B

CTAGE6
SARS
UBE3B
Itpripl2
TOR1A
CCDC176
ATG2B
GPATCH2L
OR10J3
RPL36AL
CCDC167
APOBEC3A_B
PAX6
NUP188
TREH
SLC22A7
RHOXF1
AMIGO1
AHCYL1
EPYC
NPM1
MYO10
CCDC152
APOA1BP
SPON1
TNR
ZCCHC7
RNF150
UCN2
LTB4R
ZNF776
RHOBTB1
Pirt
STK31
KPNA2

LPPR4
SETD2
RRAGD
EMC1
CDC34
ZNF530
WDTC1
MEPCE
HSDL2
CXorf51A
UGT1A10
ZDHHC21
ARFGAP1
ACE
SH3PXD2B
NKX2-6
CAMK1
ACADVL
APOA5
LRRC48
PSMA1
LOC101060161
DMRT3
BCHE
MBL2
GHRHR
SYVN1
SARM1
FAM91A1
APC
USP17L28
PPOX
STRC
XKRX
SMIM20

ELAVL2
GPKOW
WRAP73
PIAS4
BRD1
NOX5
KIF17
OR9K2
CLCC1
PVRL1
GBGT1
TSSK1B
HHAT
TELO2
AMY2A
ADRB3
EFNA5
SLC22A9
FBXL20
LRTM1
SH3PXD2A
C1orf229
INHBA
SPTBN1
MAST2
OR5M11
ERVW-1
FBL
TRAPPC4
SLC43A2
TEC
ZNF665
LDLRAD4
STK39
EHD4

ADCY5
KDELR3
MR1
VPS13A
ZNF382
CDS2
OR51G1
ZFYVE9
DDX51
GUCY1A3
SLC22A3
RNASE2
ITPKB
FXYD5
YWHAG
LOC101930515
CNBP
LOC101927989
FBXL4
C4BPB
ERCC3
C12orf40
GRHL3
HPCA
IFNGR2
RIMS4
ZNF354B
HECTD4
CNFN
KLF13
NARG2
MCF2L
TMEM259
LRRC61
GSKIP

HDGF
SCN4B
GAGE4
SLC35G1
CSRP1
LSM14A
LAMTOR2
BCAM
TRIM14
FAM206A
MARS2
YAP1
NKX3-1
SPOCK3
SPATA5L1
TNFAIP8L1
ZC3H6
ACTN3
C12ORF5
ST6GAL2
TAAR2
GPR20
PLGLB1
MBD3L2
EFCAB14
PHYHIPL
CHADL
PRMT8
CDK4
WDFY1
LINC00684
E2F8
ALG11
PKD1L1

Supplementary Table S8: Mutation quantitation.

	siControl	siCOPS2	siCON, per bp	siCOPS2, per bp
Insertions	26,447	18,890	1.08E-03	1.05E-03
Deleted bases	23,281	20,741	9.53E-04	1.15E-03
Deletions	22,670	15,924	9.28E-04	8.85E-04
Matches . Forward	14,618,326	6,585,050		
Matches , reverse	9,809,032	11,402,716		
Mismatches	4436	3651	1.82E-04	2.03E-04
Mapped bases	24,428,301	17,988,425		
read fragments	46,792	37,428		
	siControl	siCOPS2	siCON, per bp	siCOPS2, per bp
Insertions	26,447	18,890	1.08E-03	1.05E-03
Deletions	23,281	20,741	9.53E-04	1.15E-03
Mismatches	4,436	3,651	1.82E-04	2.03E-04
Mapped bases	24,428,301	17,988,425		
